# Differential Gene Expression Involved in Bone Turnover of Mice Expressing Constitutively Active TGFβ Receptor Type I

**DOI:** 10.3390/ijms25115829

**Published:** 2024-05-27

**Authors:** Ohnmar Myint, Nithidol Sakunrangsit, Jatuphol Pholtaisong, Parichart Toejing, Pinyada Pho-on, Asada Leelahavanichkul, Somyoth Sridurongrit, Chatchawit Aporntewan, Matthew B. Greenblatt, Sutada Lotinun

**Affiliations:** 1 Center of Excellence in Skeletal Disorders and Enzyme Reaction Mechanism, Department of Physiology, Faculty of Dentistry, Chulalongkorn University, Bangkok 10330, Thailand; ohnmarmyint.rl@gmail.com (O.M.); nithidol.s@hotmail.com (N.S.); jatuphol@tu.ac.th (J.P.); lookplanoi_@hotmail.com (P.T.); pinyada.pph@gmail.com (P.P.-o.); 2 Division of Immunology, Department of Microbiology, Faculty of Medicine, Chulalongkorn University, Bangkok 10330, Thailand; aleelahavanit@gmail.com; 3 Department of Anatomy, Faculty of Science, Mahidol University, Bangkok 10400, Thailand; somyoth.sri@mahidol.ac.th; 4 Department of Mathematics and Computer Science, Faculty of Science, Chulalongkorn University, Bangkok 10330, Thailand; chatchawit.a@chula.ac.th; 5 Department of Pathology and Laboratory Medicine, Weill Cornell Medicine, New York, NY 10065, USA; mag3003@med.cornell.edu; 6 Research Division, Hospital for Special Surgery, New York, NY 10065, USA

**Keywords:** TGF-β, bone turnover, bioinformatics, siRNA, osteoclasts, osteoblasts

## Abstract

Transforming growth factor beta (TGF-β) is ubiquitously found in bone and plays a key role in bone turnover. Mice expressing constitutively active TGF-β receptor type I (*Mx1;TβRI^CA^* mice) are osteopenic. Here, we identified the candidate genes involved in bone turnover in *Mx1;TβRI^CA^* mice using RNA sequencing analysis. A total of 285 genes, including 87 upregulated and 198 downregulated genes, were differentially expressed. According to the KEGG analysis, some genes were involved in osteoclast differentiation (*Fcgr4*, *Lilrb4a*), B cell receptor signaling (*Cd72*, *Lilrb4a*), and neutrophil extracellular trap formation (*Hdac7*, *Padi4*). *Lilrb4* is related to osteoclast inhibition protein, whereas *Hdac7* is a *Runx2* corepressor that regulates osteoblast differentiation. Silencing *Lilrb4* increased the number of osteoclasts and osteoclast marker genes. The knocking down of *Hdac7* increased alkaline phosphatase activity, mineralization, and osteoblast marker genes. Therefore, our present study may provide an innovative idea for potential therapeutic targets and pathways in *TβRI*-associated bone loss.

## 1. Introduction

Transforming growth factor beta (TGF-β) is the most abundant growth factor involving in bone homeostasis. The TGF-β family contains three isoforms in mammals, TGF-β1, TGF-β2, and TGF-β3, and both osteoblasts and osteoclasts secrete these three isoforms [1]. They exist in latent form in the bone matrix and the most prevalent isoform in bone is TGF-β1. TGF-β1 performs different roles in bone formation, hematopoietic cell generation, and mineral storage. In addition, TGF-β1 interacts not only with other cytokines and hormones but also with bone component cells. In mice, TGF-β1 protein is also found in bone marrow cells, cartilaginous matrix, and chondrocytes [2].

Because of the complex role of TGF-β1 in the skeletal development, there are many contradictory reports on the exact function of TGF-β1 in bone. In the skeletal osteoblastic lineage, TGF-β1 promotes bone formation by stimulating progenitor cell proliferation and migration during the initial phases but inhibits osteoblast differentiation during the late phase [3,4]. In the case of the hematopoietic osteoclastic lineage, the effect of TGF-β1 on bone resorption is dose-dependent [5]. Low levels of TGF-β1 promote osteoclast precursor migration to the site of bone resorption and their transformation into mature osteoclasts, whereas high levels of TGF-β1 suppress osteoclast migration by altering the production of receptor activator of nuclear factor kappa-Β ligand (RANKL) and osteoprotegerin (OPG) by osteoblasts [5,6]. However, studies in transgenic mice revealed that elevated levels of TGF-β in the bone microenvironment fostered osteoclast formation and bone resorption and suppressed osteoblast differentiation and bone formation [7,8].

TGF-β is expressed as an inactive complex consisting of a latency-associated polypeptide (LAP) and a mature polypeptide that can be activated by releasing proteases from cells. TGF-β controls cellular functions by interacting with the high-affinity serine/threonine kinase TGF-β receptor type I (TβRI), TβRII, and TβRIII. Active TGF-β signals through the Smad-mediated or canonical signaling pathway. Following the activation of TβRII, subsequent activation and the phosphorylation of TβRI occurred. Type I receptor activation triggers the phosphorylation of receptor-regulated Smads (R-Smads or Smad 2/3), and activated R-Smads form a complex with common-partner Smad (Co-Smad or Smad 4) and then translocate into the nucleus for transcription. Apart from canonical pathways, TGF-βs can activate Smad-independent or non-canonical pathways, including PI3K/AKT, ERK1/2, JNK, and p38 cascades. Changes in TGF-β signaling result in altered bone mass and poor bone quality in various skeletal disorders.

In *TGF-β1^−/−^* mice, tibia length was shorter than that of wild-type (WT) or *TGF-β1^+/−^* mice, and the bone mineral content of proximal tibial metaphysis was significantly decreased with a reduction in bone elasticity [9]. *Tgfβ3^−/−^* mice display defective palatal shelf fusion [10]. *Osterix-cre;Tgfbr2^f/f^* mice exhibited osteochondral dysplasia, irregular articular cartilage, and early postnatal death around 3–4 weeks of age [11]. In subchondral bone, the numbers of osteoclasts were increased, and bone resorption was activated by a higher concentration of TGF-β1, leading to osteoarthritis [8]. Moreover, transgenic mice with an osteoblastic-specific overexpression of TGF-β2 displayed increased bone remodeling [7]. It was also reported that an imbalance in activity of osteoblasts and osteoclasts in this model caused progressive bone loss, defective bone mineralization, and clavicular hypoplasia. Therefore, identifying the potential mechanisms of bone loss associated with TGF-β is key for the prevention and treatment of osteoporosis.

Our previous study indicated that Mx1-cre mediated the overexpression of *TβRI* in hematopoietic cells and other cells in mice, inducing bone loss [12]. However, the downstream effector genes mediate this bone loss, and therefore the TGF-β-pathway-mediated mechanisms of hematopoietic to bone crosstalk remain largely unknown. In this study, we used the *TβRI* overexpression mouse model and carried out RNA sequencing (RNA-seq) from femoral bone cells to compare the gene expression profile in *Mx1;TβRI^CA^* mice with WT controls. We also performed pathway association in differentially expressed genes (DEGs) involved in *TβRI* overexpression and bone turnover. We defined the candidate genes in osteoclasts and osteoblasts which were involved in bone loss. These genes may be used as potential targets for preventing bone loss.

## 2. Results

### 2.1. RNA-Seq Shows DEGs in Mx1;TβRI^CA^ Mice

**Figure 1:**
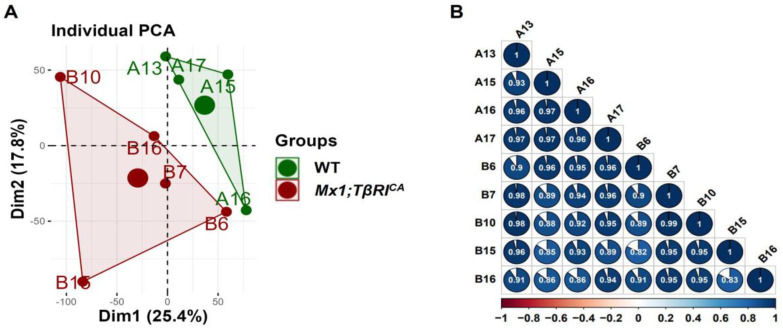
Variation in the expression data between WT and *Mx1;TβRI^CA^* mice groups. (**A**) Principal component analysis (PCA) plot between the two groups. The green dots represent WT controls (*n* = 4) and the red dots represent *Mx1;TβRI^CA^* (*n* = 5). The larger dots represent groups of samples. The smaller dots represent each sample within the groups. The percentages of total variation for dim1 and dim2 are shown on the x and y axes, respectively. (**B**) Pearson’s correlation matrix between WT controls and *Mx1;TβRI^CA^*. Positive correlations are explained by colors that lean toward dark blue, whereas negative correlations are represented by colors that lean toward dark red. The shape of the pie chart identifies the positive or negative correlation between the samples. dim = dimension.

**Figure 2:**
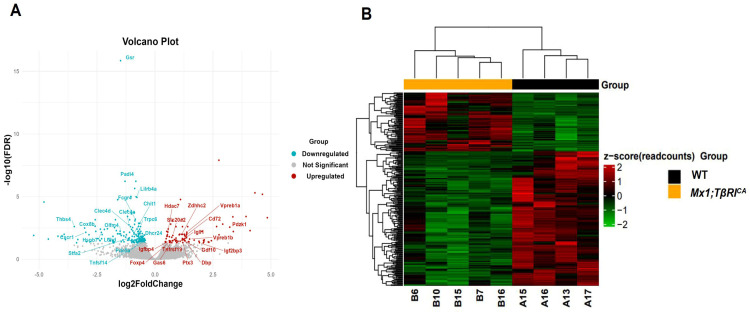
RNA-seq analysis of DEGs in WT and *Mx1;TβRI^CA^* groups. (**A**) Volcano plot of all DEGs. Grey nodes represent non-significant genes. Red nodes represent significantly upregulated genes (*n* = 87) with the threshold value of log2 fold change > 0 and FDR < 0.05 in comparison with WT controls. Blue nodes represent significantly downregulated genes (*n* = 198) with the threshold value of log2 fold change < 0 and FDR < 0.05 in *Mx1;TβRI^CA^* mice compared to WT controls. (**B**) Heatmap showing how DEGs are generally expressed in each sample. Each line represents one gene, and each column represents a distinct sample. The red color indicates upregulation, whereas the green color indicates downregulation. DEGs = differentially expressed genes.

According to bioinformatics analysis of RNA-seq in femoral bone cells of WT controls and *Mx1;TβRI^CA^* mice, 15,554 genes were expressed. Among the 15,554 genes, 285 genes were significantly differentially expressed, including 87 upregulated and 198 downregulated genes. For filtering read counts, the “filterByExpr function” in the edgeR package (version 3.4.0) was used. Figure 1A shows the principal component analysis (PCA) plot between WT and *Mx1;TβRI^CA^* groups. No overlap between these two clusters in the PCA plot indicated that there was a significant difference between WT and *Mx1;TβRI^CA^* groups. The dimensions dim1 and dim2 were 25.4% and 17.8%, respectively. The corheatmap results (Figure 1B) showed that there was a positive correlation in each sample, which was consistent with the PCA results. The volcano plot of upregulated (*n* = 87) and downregulated (*n* = 198) DEGs in *Mx1;TβRI^CA^* versus WT mice is shown in Figure 2A. The DEGs in *Mx1;TβRI^CA^* versus WT mice were further identified by heatmap analysis (Figure 2B).

### 2.2. The Validated Genes by the qPCR Data Were Consistent with the RNA-Seq Data

**Figure 3:**
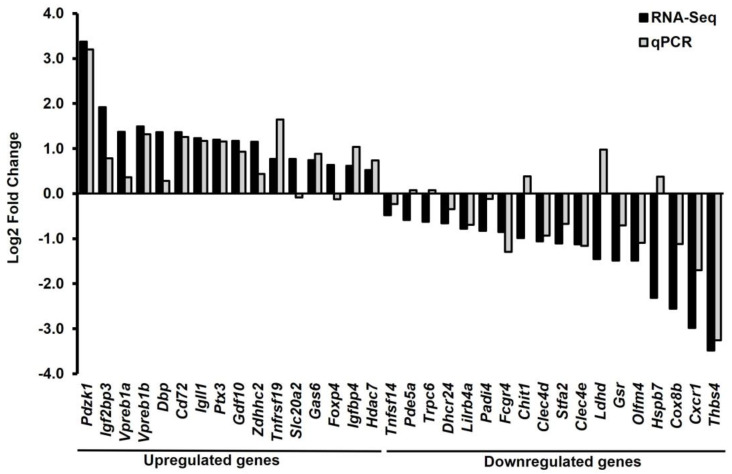
Log2 fold change of selected DEGs were compared between RNA-Seq and qPCR. Totals of 16 upregulated (*n* = 6) and 18 downregulated genes (*n* = 6) were selected in this study. About 79% of the differentially expressed genes identified by RNA-Seq could be validated by qPCR. DEGs = differentially expressed genes.

**Figure 4:**
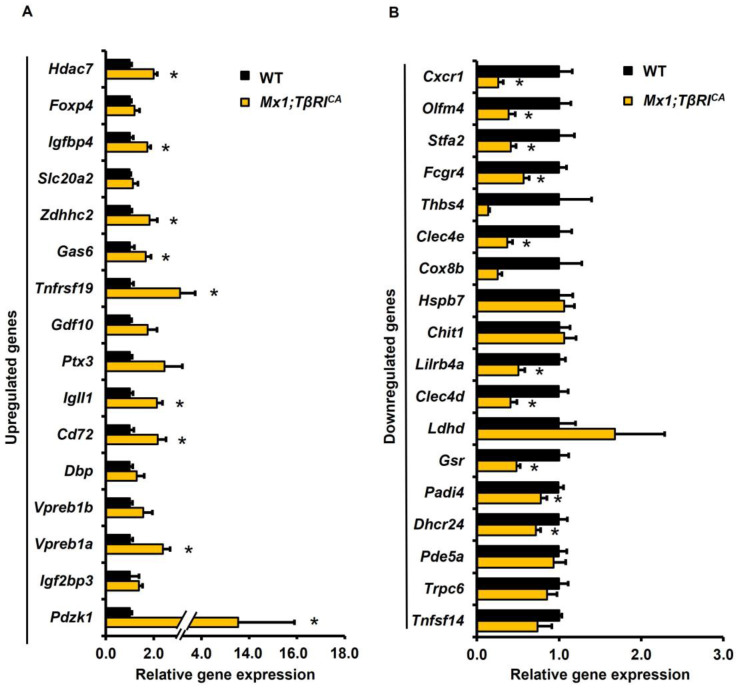
The validation of RNA-seq data by qPCR analysis of 34 DEGs. (**A**) Upregulated (16 genes) and (**B**) downregulated (18 genes) DEGs between *Mx1;TβRI^CA^* mice (*n* = 5) and WT controls (*n* = 5). Data are mean ± SEM. * *p* < 0.05 compared to WT controls. DEGs = differentially expressed genes.

Most of the validated genes by the qPCR data were consistent with about 79% (27/34) of the RNA-seq data (Figure 3). The RNA-seq data were validated by qPCR analysis based on a fold change and *p*-values. Thirty-four DEGs (16 upregulated and 18 downregulated DEGs) were selected for qPCR analysis. According to the RNA-seq data, *Pdzk1*, *Igf2bp3*, *Vpreb1a*, *Vpreb1b*, *Dbp*, *Cd72*, *Igll1*, *Ptx3*, *Gdf10*, *Zdhhc2*, *Tnfrsf19*, *Slc20a2*, *Gas6*, *Foxp4*, *Igfbp4*, and *Hdac7* were upregulated, while *Tnfsf14*, *Pde5a*, *Trpc6*, *Dhcr24*, *Lilrb4a*, *Padi4*, *Fcgr4*, *Chit1*, *Clec4d*, *Stfa2*, *Clec4e*, *Ldhd*, *Gsr*, *Olfm4*, *Hspb7*, *Cox8b*, *Cxcr1*, and *Thbs4* were downregulated. The results of qPCR analysis showed that 9 upregulated genes, *Pdzk1*, *Vpreb1a*, *Cd72*, *Igll1*, *Tnfrsf19*, *Gas6*, *Zdhhc2*, *Igfbp4*, and *Hdac7,* were significantly increased, and 10 downregulated genes, *Dhcr24*, *Padi4*, *Gsr*, *Clec4d*, *Lilrb4a*, *Clec4e*, *Fcgr4*, *Stfa2*, *Olfm4*, and *Cxcr1,* were significantly decreased in *Mx1;TβRI^CA^* compared to the WT group (Figure 4A,B). The functions of these 19 DEGs are briefly described in Supplementary Tables S1 and S2 [13,14,15,16,17,18,19,20,21,22,23,24,25,26,27,28,29,30,31,32,33,34,35].

### 2.3. DEGs Were Expressed in Osteoclast Differentiation Pathway in Mx1;TβRI^CA^ Mice

**Figure 5:**
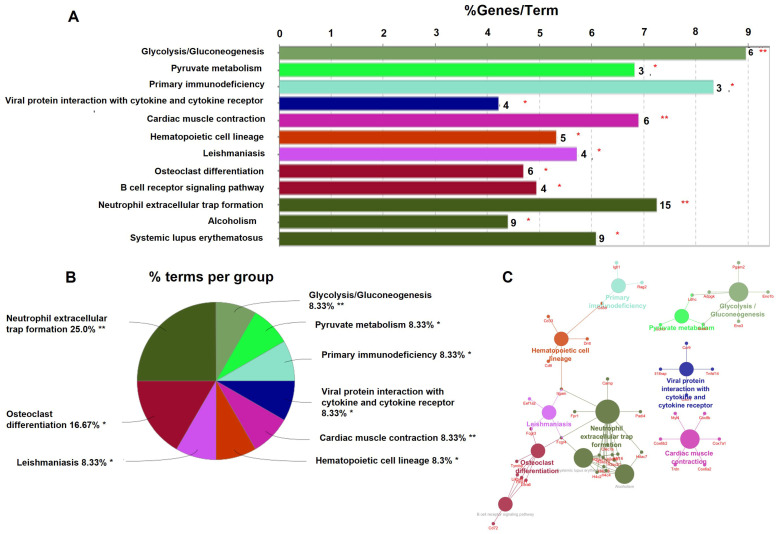
KEGG signaling pathway of significant up- and downregulated DEGs. (**A**) Significant KEGG pathways shown in a bar plot according to the adjusted *p* < 0.05. The number in each bar represents the number of genes involved in each pathway. (**B**) Pie chart representing the percentage of each pathway. (**C**) The KEGG pathway network shows the name of genes involved in each pathway. The size of circles in each pathway represents the number of genes involved in each pathway. * *p* < 0.05, ** *p* < 0.001.

To gain insight into gene functions and their interactions, pathway enrichment analysis was performed. The Cytoscape plug-in ClueGO was used for Kyoto Encyclopedia of Genes and Genomes (KEGG) enrichment analysis in 285 DEGs. The results showed that a total of 17 pathway terms were enriched with 285 DEGs. A total of 12 pathways were significant with *p* < 0.05 (Figure 5). The significantly enriched pathways were glycolysis/gluconeogenesis, pyruvate metabolism, primary immunodeficiency, viral protein interaction with cytokine and cytokine receptors, cardiac muscle contraction, the hematopoietic cell lineage, leishmaniasis, osteoclast differentiation, the B cell receptor signaling pathway, neutrophil extracellular trap (NET) formation, alcoholism, and systemic lupus erythematosus. Significant KEGG pathways are shown in Figure 5A. The percentage of genes involved in each pathway is shown in Figure 5B. The names of genes involved in each pathway are shown in Figure 5C. *Lilrb4* is involved in osteoclast differentiation and B cell receptor signaling pathways. *Hdac7* is involved in NET formation and alcoholism pathways. These two genes are involved in the regulation of osteoblast and osteoclast differentiation [25,30]. In addition, *Lilrb4* and *Hdac7* were significantly downregulated and upregulated in our qPCR analysis, respectively. Therefore, we selected these two genes to determine the effect on bone turnover in *Mx1;TβRI^CA^* mice.

### 2.4. Protein–Protein Interaction (PPI) Connection Occurred between DEGs

**Figure 6:**
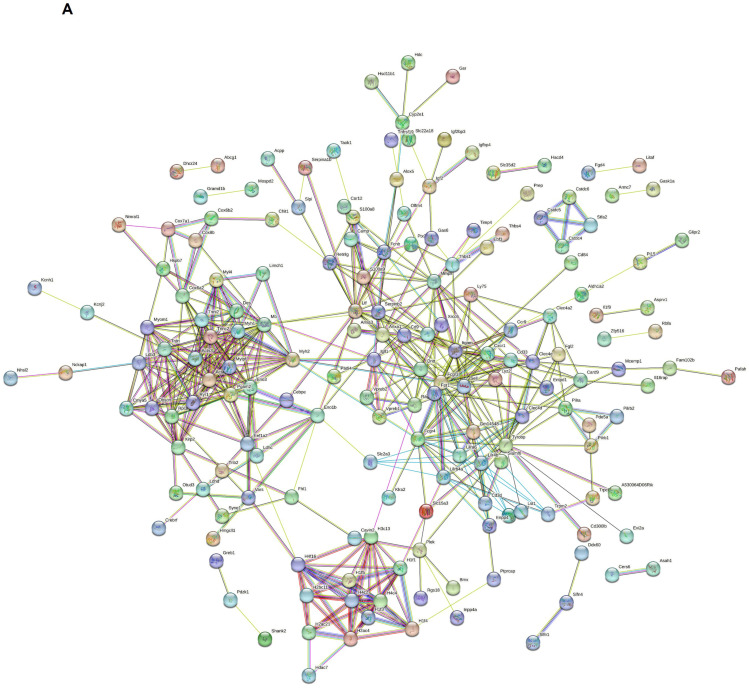

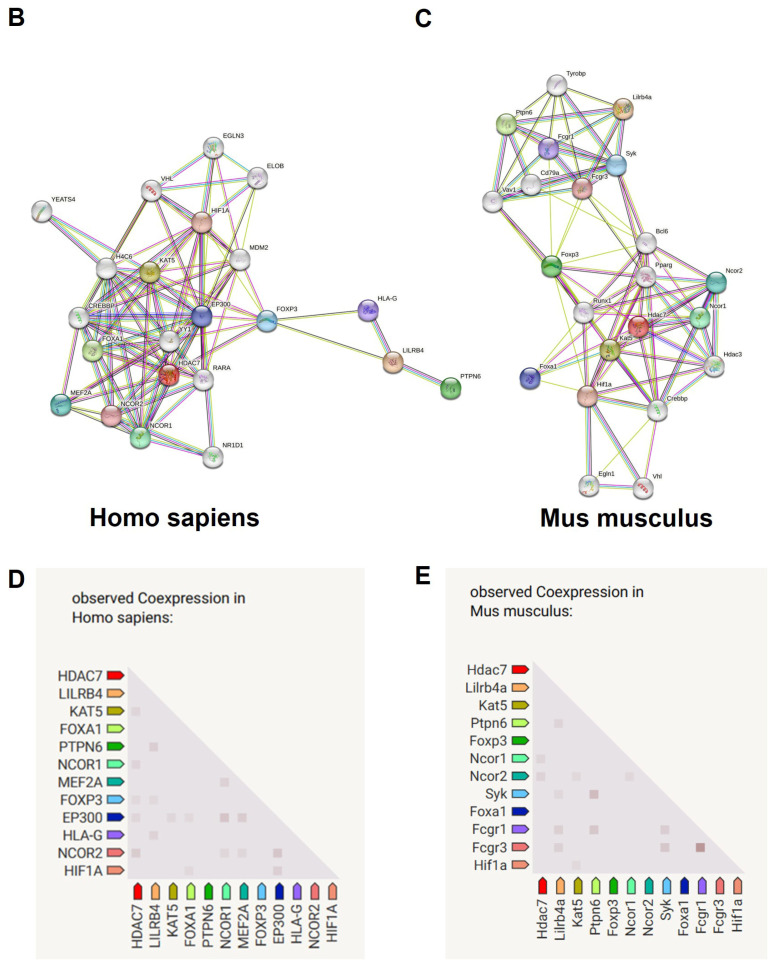
Protein–protein interaction (PPI) network of significant DEGs. STRING database was used to obtain PPI data. (**A**) PPI network of significant DEGs with 254 nodes and 510 edges. PPI network of *Hdac7* and *Lilrb4* in humans (**B**) and in mice (**C**). Co-expression between the genes in humans (**D**) and in mice (**E**).

The STRING database was used to establish the PPI network of significant DEGs after removing disconnected nodes, which is shown in Figure 6A. A minimum required interaction default score of 0.4 was used. This PPI network contained 254 nodes and 510 edges with an average local clustering coefficient of 0.425 and node degree of 4.02. The PPI enrichment value was <1.0 × 10^−16^. The PPI network of *Hdac7* and *Lilrb4* in humans and in mice is shown in Figure 6B,C. In humans, *Hdac7* showed co-expression with *NCOR2*, *EP300*, *FOXP3*, *NCOR1*, and *KAT5*, and co-expression scores were 0.097, 0.049, 0.044, 0.058, and 0.056, respectively (Figure 6D). *Lilrb4* in humans showed co-expression with *HLA-G*, *FOXP3*, and *PTPN6*, and co-expression scores were 0.062, 0.044, and 0.128, respectively (Figure 6D). In mice, *Hdac7* showed co-expression with *Ncor2* and *Ncor1,* and co-expression scores were 0.067 and 0.057, respectively (Figure 6E). *Lilrb4* in mice showed co-expression with *Fcgr3*, *Fcgr1*, *Syk*, and *Ptpn6*, and co-expression scores were 0.111, 0.108, 0.097, and 0.103, respectively (Figure 6E). Some genes that are co-expressed with *Hdac7* are involved in the Notch signaling pathway, and those co-expressed with *Lilrb4* are involved in the osteoclast differentiation signaling pathway.

### 2.5. Proinflammatory Markers Were Elevated in Mx1;TβRI^CA^ Mice

**Figure 7:**
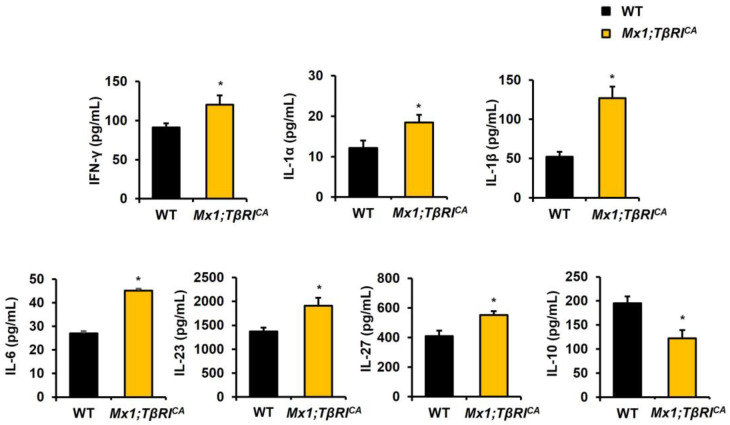
*Mx1;TβRI^CA^* mice had increased inflammatory cytokines. Serum levels of IFN-γ, IL-1α, IL-1β, IL-6, IL-23, IL-27, and IL-10 in *Mx1;TβRI^CA^* mice (*n* = 5) and WT controls (*n* = 5). Data are mean ± SEM. * *p* < 0.05 compared to WT controls.

Inflammatory-related bone loss in *Mx1;TβRI^CA^* mice was further confirmed by checking proinflammatory cytokine serum levels. *Mx1;TβRI^CA^* mice had significantly elevated IFN-γ, IL-1α, IL-1β, IL-6, IL-23, and IL-27 serum concentrations (Figure 7 and Supplementary Table S3). IL-10 serum levels were significantly decreased in *Mx1;TβRI^CA^* mice (Figure 7). These data suggested that a continuous increase in *TβRI* expression caused bone loss by increasing circulating proinflammatory cytokines levels in *Mx1;TβRI^CA^* mice.

### 2.6. Silencing of Lilrb4 and Hdac7 Promotes Osteoclast and Osteoblast Differentiation

**Figure 8:**
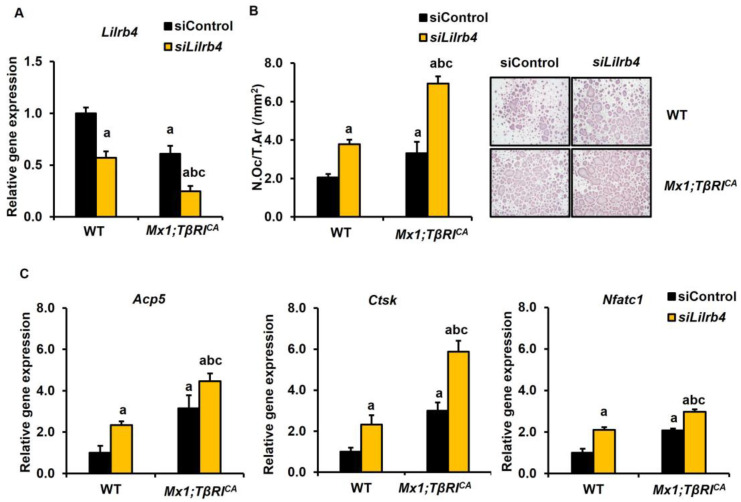
Silencing *Lilrb4* promotes osteoclastogenesis in WT and *Mx1;TβRI^CA^* bone cells. (**A**) qPCR analysis of the efficiency of *Lilrb4* knockdown in BMMs from long bones of 7-week-old *Mx1;TβRI^CA^* mice and WT controls (*n* = 3–4). (**B**) TRAP-positive osteoclasts with more than 5 nuclei were counted using OsteoMeasure software version 4.2.0.1 (Decatur, GA, USA). (**C**) The qPCR analysis of osteoclast gene expression after *siLilrb4* transfection. Data are mean ± SEM. ^a^ *p* < 0.05 compared to WT cells transfected with siControl, ^b^ *p* < 0.05 compared to WT cells transfected with *siLilrb4,* and ^c^ *p* < 0.05 compared to *Mx1;TβRI^CA^* cells transfected with siControl. N.Oc, osteoclast number; Ar, area. Scale bar: 200 μm.

To investigate the regulatory role of *Lilrb4* and *Hdac7* on bone homeostasis, the siRNA-mediated depletion of *Lilrb4* in BMMs and *Hdac7* in primary osteoblasts was performed. The mRNA levels of *Lilrb4* were significantly lower in *Mx1;TβRI^CA^* osteoclast cells compared to WT controls (Figure 8A). The efficiency of *Lilrb4* knockdown was evaluated by qPCR. The expression levels of *Lilrb4* were decreased in WT and *Mx1;TβRI^CA^* transfected with *siLilrb4*. As *Lilrb4* is a negative regulator of osteoclasts, there was an increase in TRAP-positive multinucleated osteoclasts in both WT and *Mx1;TβRI^CA^* after silencing *Lilrb4* (Figure 8B). Two-way ANOVA showed an additive effect between the overexpression of *TβRI* and *siLilrb4* in osteoclast numbers (Supplementary Table S4). *Lilrb4*-depleted cells had enhanced mRNA levels of *Acp5*, *Ctsk*, and *Nfatc1* (Figure 8C). Two-way ANOVA showed that there was no interaction between the overexpression of *TβRI* and *siLilrb4* in osteoclast marker genes.

**Figure 9:**
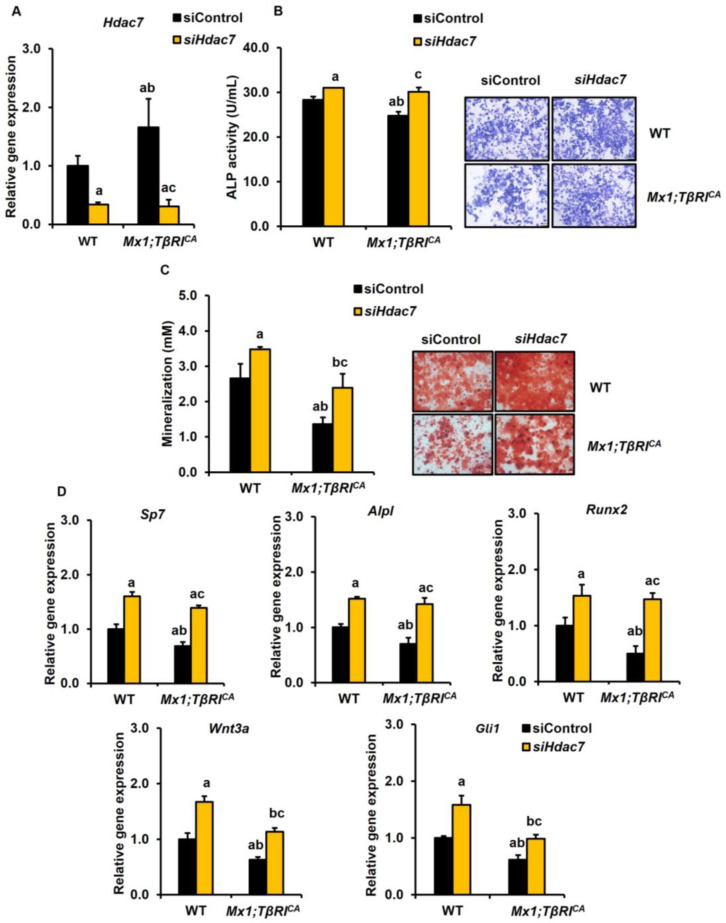
Silencing *Hdac7* promotes osteoblastogenesis in WT and *Mx1;TβRI^CA^* bone cells. (**A**) qPCR analysis of the efficiency of *Hdac7* knockdown in osteoblasts from long bones of 7-week-old *Mx1;TβRI^CA^* mice, and WT controls (*n* = 3–4). (**B**) ALP activity and (**C**) mineralization in *Mx1;TβRI^CA^* mice compared to WT controls were determined. (**D**) The qPCR analysis of osteoblast gene expression after *siHdac7* transfection. Data are mean ± SEM. ^a^ *p* < 0.05 compared to WT cells transfected with siControl, ^b^ *p* < 0.05 compared to WT cells transfected with *siHdac7,* and ^c^ *p* < 0.05 compared to *Mx1;TβRI^CA^* cells transfected with siControl. Scale bar: 200 μm.

The mRNA levels of *Hdac7* were increased in *Mx1;TβRI^CA^* osteoblasts compared to WT controls (Figure 9A). The expression levels of *Hdac7* were decreased in WT and *Mx1;TβRI^CA^* transfected with *siHdac7*. As *Hdac7* was a negative regulator of osteoblasts, silencing *Hdac7* statistically increased ALP activity in both WT and *Mx1;TβRI^CA^* osteoblasts (Figure 9B). Furthermore, a mineralization assay showed that *Hdac7* knockdown also increased mineralization in both WT and *Mx1;TβRI^CA^* osteoblasts (Figure 9C). *Mx1;TβRI^CA^* mice had decreased osteoblastic gene markers, including *Sp7*, *Alpl*, *Runx2*, *Wnt3a*, and *Gli1* (Figure 9D). These genes were increased in *Hdac7*-depleted *Mx1;TβRI^CA^* osteoblasts compared to WT littermates. Two-way ANOVA showed that there was no interaction between the overexpression of *TβRI* and *siHdac7* and any parameters (Supplementary Table S5). These findings suggested that the silencing of *Lilrb4* and *Hdac7* increased bone resorption and bone formation, respectively.

## 3. Discussion

TGF-β is involved in the regulation of bone cells, such as osteolineage progenitors, osteoblasts, osteoclasts, and chondrocytes. Dysregulation in TGF-β causes abnormal bone activity. A higher concentration of TGF-β1 caused accelerated bone resorption and osteoarthritis [8]. Our previous study indicated that conditionally activated TβRI in *Mx1;TβRI^CA^* mice induced bone loss with a decrease in bone density in femurs and mandibles compared to WT controls [12]. In addition, cortical bone hardness was also reduced. Furthermore, osteoclast marker genes were increased and osteoblast marker genes were decreased in *Mx1;TβRI^CA^* mice. However, the involvement of genes that play roles in TGF-β signaling related to bone loss needed to be verified. Based on our study, we further investigated the involvement of candidate genes in the process of bone turnover in *Mx1;TβRI^CA^* mice. RNA-seq is one of the most indispensable tools for transcriptomic analysis of the gene expression profile. Our RNA-seq results revealed that 285 DEGs were identified in femoral bones from *Mx1;TβRI^CA^* mice compared to WT controls, of which 87 were upregulated and 198 were downregulated.

Our results indicated that some downregulated genes were involved in osteoclast differentiation (*Lilrb4*, *Fcgr4*, *Olfm4*, *Cxcr1*). *Lilrb4a*, also known as *Lilrb4*, is a cell surface immunosuppressive receptor containing three immunoreceptor tyrosine-based inhibition motifs (ITIMs). It is expressed in endothelial cells, osteoclasts, macrophages, immune cells such as monocytes, and dendritic cells. It works in colligation with IgG Fc receptors. Upon activation of *Lilrb4*, Src family tyrosine kinases cause the phosphorylation of ITIM, which in turn recruits the SHPs (SHP1, SHP2, SHIP1), leading to inhibiting inflammation via the inhibition of Syk, PI3K, and calcium signaling. Because of these connections, *Lilrb4* showed co-expression with Fcgr1 and 3 and Syk proteins in our PPI results (Figure 6E). *Lilrb4* is involved in immune responses and inflammatory processes associated with autoimmune diseases, infectious diseases, inflammation disorders, and cancers and is negatively regulated in immune cell activation. *Lilrb4* deletion increased the inflammatory response by macrophages via triggering NF-κB signaling [36]. It is involved not only in the immune response of various diseases but also in bone-related immunity. Although different *Lilrb4* inhibitors for anti-tumor therapy have emerged in pre-clinical studies, there are only a few studies on the effects of *Lilrb4* on bone cells. It is reported that activated SHP-1 inhibits osteoclast differentiation by the dephosphorylation of Syk protein [37]. Mori et al. revealed that cultured osteoclast precursor cells of paired Ig-like receptor (PIR)-B from mouse and leukocyte Ig-like receptors (LILR)B from human origin showed that ITIM recruited SHP-1 in the presence of RANKL and M-CSF and suppressed the development of osteoclasts in vitro [30]. In addition, the expression of *Lilrb3* and *Lilrb4* in osteoclasts of human peripheral blood monocytes showed a downregulation of osteoclast development via association with SHP-1. In our report, we identified *Lilrb4* as a major gene involving bone loss of the *Mx1;TβRI^CA^* model via osteoclast differentiation signaling.

Our results showed that upregulated genes were associated with osteoblast differentiation (*Igll1*, *Tnfrsf19*, *Zdhhc2*, *Igfbp4*, *Hdac7*) and *Hdac7* may cause bone loss in *Mx1;TβRI^CA^* mice. *Hdac7* is one of the members of the class IIa histone deacetylase (HDAC) subfamily. It is a co-repressor of gene transcription in the skeleton, plays a role in chromatin structure regulation, and regulates bone formation and resorption. Bradley et al. stated that the deletion of *Hdac7* promotes chondrocyte proliferation, β-catenin activity, and the regeneration of cartilage by regulating the canonical Wnt signaling pathway [38]. *LysM;Hdac7* knockouts exhibited accelerated osteoclastogenesis, bone resorption, and a reduction in bone mass by changing the RANKL/NFATc1-mediated β-catenin activity [22]. Stemig et al. and Pham et al. suggested that *Hdac7* suppression in osteoclasts enhanced osteoclast formation, increased TRAP^+^ multinucleated cell numbers, and reduced bone mass by repressing the transcriptional activity of MITF in a deacetylase-independent manner [23,24]. Furthermore, the expression of osteoclast genes such as *Nfatc1, Ctsk*, and *DC-STAMP* was increased [24]. *Runx2*, located in the nuclear matrix, plays a role as a key regulator of osteoblast differentiation. *Runx2^−/−^* mice showed a lack of mineralized bone in both calvaria and long bones, indicating that *Runx2* is essential for intramembranous ossification and endochondral bone formation [39]. Several cofactors like *Hdacs* regulate the *Runx2* activity as corepressors. TGF-β cooperates with class IIa *Hdac* in the repression of *Runx2* by Smad3 in osteoblasts differentiation [40]. As *Hdac7* is also a member of class IIa *Hdacs*, *Hdac7* negatively regulates the transcription of *Runx2* in which they are co-localized in the nuclei and regarded as a *Runx2* co-repressor [25]. In *Hdac7* knockdown C2C12 cells, osteoblast maturation was enhanced and the expression of osteoblast genes such as *Alpl*, *Runx2*, *Osx*, *Col1a1*, and *ER-α* was increased, but *OPG* was downregulated by short hairpin RNAs. BMP signaling regulated *Runx2* activity by the nuclear export of *Hdac7* [41]. BMP2 stimulated protein kinase D in osteoblast-like cells. PKD1 stimulated the phosphorylation-dependent nuclear export of *Hdac7*. PKD1 suppressed the ability of *Hdac7* to repress *Runx2* transcription. BMP belongs to the TGF-β superfamily and signals via two classes of serine/threonine kinase receptors. Specifically, type II receptors mediate the phosphorylation of type I receptors. TGF-β/BMP plays a role in bone homeostasis through both Smad-dependent and Smad-independent signaling pathways. There is an interplay between TGF-β and BMP protein in bone formation. TGF-β1 markedly accelerated the BMP2-induced bone formation when compared with BMP-2 alone [42]. TGF-β1 signaling in turn prolonged BMP signaling and triggered BMP-2-induced osteoblast differentiation in ST2 mesenchymal stromal cells [43]. Two types of dominant negative type II TGF-β receptors (BMPRII and ActRII) decreased BMP-9-induced osteogenesis, resulting in decreased ALP activity and mineralization in C3H10T1/2 stem cells and decreased bone mass in vivo [44]. Therefore, TGF-β has context-dependent effects on BMP signaling in bone formation. O’Neil et al. reported that NET caused an increase in osteoclasts formation via Toll-like receptor 4 signaling and NET-associated proteins such as histones and neutrophil elastase in rheumatoid arthritis [45]. They also stated that carbamylated histones and inflammatory markers were elevated in rheumatoid arthritis patients, leading to bone resorption. Therefore, we identified *Hdac7* as a responsive gene in bone loss in the *Mx1;TβRI^CA^* model.

Another factor that regulates bone remodeling is matrix metalloproteinases (MMPs). MMPs are members of zinc-dependent endopeptidase and are responsible for extracellular matrix remodeling, bone regeneration, bone resorption, and bone formation. MMPs are expressed by both osteoclasts and osteoblasts. MMPs can influence bone turnover and remodeling via their interactions with other proteins like TGF-β. The MMP-13 expression level was upregulated by TGF-β in osteoblasts, leading to changes in osteoblast morphology, which, in turn, accelerates bone resorption by osteoclasts [46]. It is also reported that the bioavailability of TGF-β regulates bone matrix composition and that bone hardness is regulated by both MMP-2 and MMP-9 [47,48].

TGF-βs interplay between skeletal and immune systems. TGF-β dysregulation results in a disruption of the immune system as it regulates the immune responses. Gao et al. indicated that bone loss occurred in the disruption of T cell-specific TGF-β signaling [49]. In addition, our KEGG pathways showed that *TβRI* overexpression was linked to many signaling pathways such as viral protein interaction with cytokine and cytokine receptors, osteoclast differentiation, the B cell receptor signaling pathway, NET formation, and systemic lupus erythematosus. *Lilrb4* and *Hdac7* were involved in bone loss mechanisms of *Mx1;TβRI^CA^* mice. *Lilrb4* works together with IgG Fc receptors and Syk protein in the process of osteoclast differentiation. *Ptpn6*, also known as SHP-1, is involved in osteoclast differentiation via the RANK pathway which is an indirect activation by *Lilrb4*. Therefore, *Lilrb4* showed co-expression with Fcgr3, Fcgr1, Syk, and Ptpn6 proteins in mice and PTPN6 protein in humans involved in the osteoclast differentiation signaling pathway. Nuclear receptor corepressor (NCOR), as a co-repressor, recruits *Hdacs* to suppress gene expression. Even though there is no direct relation between HDAC7 and NCOR protein, our interaction results showed that HDAC7 had an association with NCOR1 and NCOR2 proteins in humans and in mice which may be involved in osteoblast differentiation.

*Lilrb4* is a negative regulator of osteoclast differentiation by inhibiting Syk proteins, leading to decreased bone resorption. In our study, silencing *Lilrb4* in osteoclast cells promoted osteoclast formation and increased osteoclast marker genes (*Acp5*, *Ctsk*, and *Nfatc1*) in both *Mx1;TβRI^CA^* and WT controls. The number of TRAP^+^ multinucleated mature osteoclasts was higher in the *Lilrb4* knockdown of *Mx1;TβRI^CA^* and WT cells. Consistent with our findings, PIR-B-deficient mice had increased bone resorption and the expression of *Lilrb4* in osteoclasts of human peripheral blood monocytes showed a downregulation of osteoclast development [30]. Therefore, *Lilrb4* was involved in the osteoclast differentiation pathway. *Hdac7* is a co-repressor of *Runx2*. *Hdac7* knockdown increased the expression of osteoblast genes such as *Alp*, *Runx2*, *Osx*, *Col1a1*, and *ER-α* [25]. Our study confirmed that silencing *Hdac7* in osteoblast cells promoted osteoblast formation, increased ALP activity and mineralization, and increased osteoblast marker genes (*Sp7*, *Alpl*, *Runx2*, *Wnt3a*, and *Gli1*) in both *Mx1;TβRI^CA^* and WT controls. The formation of NETs decreased osteoblast activity by releasing histones in rheumatoid arthritis [45,50]. Therefore, *Hdac7* may be involved in NET formation signaling by regulating osteoblast formation.

Several proinflammatory cytokines are involved in the process of bone turnover. In inflammatory diseases like osteoarthritis and rheumatoid arthritis, accelerated bone destruction occurred with the increase in production of proinflammatory cytokines levels. In our previous study, the constitutive activation of *TβRI* induced an increase in osteoclast differentiation which caused osteopenia [12]. Increasing proinflammatory cytokines may cause RANKL/OPG axis dysregulation resulting in enhanced osteoclastogenesis. IFN-γ has different roles in osteoclastogenesis. IFN-γ receptor knockout mice protected ovariectomy-induced bone loss [51]. It is also stated that IFN-γ has a promoting effect on osteoclast differentiation through T cell activation in vivo [52]. Likewise, the IFN-γ level was elevated in our study. IL-1 also plays a role in inflammatory-mediated bone loss. IL-1α^−/−^ and IL-1β^−/−^ mice inhibited osteoclast formation and bone resorption even though there was inflammation in joints [53]. Similarly, our results showed that IL-1α and IL-1β proinflammatory cytokines levels were increased in *Mx1;TβRI^CA^* mice. In osteocyte-like MLO-Y4 cells, stimulation with IL-6 promoted osteocyte-mediated osteoclastogenesis, JAK2 activation, and the formation of TRAP^+^ multinucleated cells [54]. In addition, the IL-6 overexpression mouse model showed increased osteoclasts, and skeletal development was impaired with a reduction in trabecular and cortical bone mass [55]. In our result, the IL-6 level was also elevated. IL-23 plays a pivotal role in several inflammatory conditions. Consistent with our results of the IL-23 level, ovariectomized mice had an increased IL-23 serum level and reduced trabecular bone density, leading to bone loss [56]. In the proteoglycan-induced arthritis (PGIA) mice model, IL-27R^−/−^ mice showed a delay in the development of arthritis compared to WT mice [57]. Our results also showed that the IL-27 level was elevated. IL-10 is an anti-inflammatory cytokine that limits pro-inflammatory cytokine (IFN-γ, IL-1, IL-6) secretion. In the osteoporosis model of postmenopausal mice, serum IL-10 cytokine levels were significantly reduced [58]. TGF-β1 and IL-10 interplayed with each other in the osteoarthritis rat model [59]. Serum levels of TGF-β1 were increased but those of IL-10 were decreased in osteoarthritis rats. Consistent with their findings, our IL-10 serum levels were significantly decreased in *Mx1;TβRI^CA^* mice. Therefore, our results demonstrated that alterations in the proinflammatory cytokines levels were associated with bone loss in TGF-β receptor I overexpression by increasing osteoclast formation.

Taken together, this study identified candidate genes impacting bone homeostasis and their pathways in *Mx1;TβRI^CA^* mice. Most of the upregulated genes in *Mx1;TβRI^CA^* mice were related to intracellular activities, whereas the downregulated genes were associated with immune activities. *Lilrb4* was related in the osteoclast differentiation pathway and decreased bone resorption, whereas *Hdac7* was involved in osteoblast differentiation and inhibited bone formation. These candidate genes might be used as potential biomarkers and therapeutic targets for the diagnosis or treatment of bone loss in *TβRI* overexpression.

## 4. Materials and Methods

### 4.1. Mice

Mice from Dr. Laurent Bartholin were transferred from the Department of Anatomy, Faculty of Science, Mahidol University, Bangkok, Thailand. Constitutively active *TβRI* was knocked into X-chromosome-linked hypoxanthine phosphoribosyl transferase. Female *TβRI^CA^* mice were used in this study and PCR genotyping from the tail of *TβRI^CA^* and *Mx1;TβRI^CA^* mice was carried out based on a previous study [12]. Mice were housed at the Faculty of Medicine, Chulalongkorn University, where they had free access to water, standard rodent food (C.P. Mice Feed, Perfect Companion Group Co., Ltd., Bangkok, Thailand), and a temperature-regulated environment (25 ± 2 °C). The animal research protocol was approved by the Institutional Animal Care and Use Committee (IACUC) at the Faculty of Medicine, Chulalongkorn University. This study complied with the guidelines of the Animal Research: Reporting of In Vivo Experiments (ARRIVE, Singapore). Seven- and nine-week-old *Mx1;TβRI^CA^* and WT females were used in this study. At the end of the experiment, mice were anesthetized with isoflurane. Blood samples were collected to prepare serum and kept at −80 °C. Femurs were frozen in liquid nitrogen and kept at −80 °C for RNA isolation and RNA-seq analysis. A previous publication was used as a reference for calculating sample size [12]. Sample size calculation for animal experiments was carried out by power calculation using priori. At least four animals per group were used to reach a power of 0.80 with an alpha value of 0.05. Animal groups were randomized and blinded throughout the experiment.

### 4.2. RNA Extraction, RNA-Seq Data Analysis, and qPCR Analysis

WT and *Mx1;TβRI^CA^* femurs were pulverized in liquid nitrogen. RNA was isolated by Trizol (Invitrogen, Waltham, MA, USA) and purified with the RNeasy Mini kit (Qiagen, Germantown, MD, USA).

For RNA-seq data analysis, total RNA concentration was assessed by using Nanodrop (Thermo Fisher Scientific, Waltham, MA, USA). The integrity of the total RNAs was determined to validate the quality of isolated RNAs by using Bioanalyzer (Agilent 2100, Santa, Clara, CA, USA). Approximately 500 ng of the total RNAs from each sample was used to construct RNA-seq libraries by using QIAseq Stranded mRNA Library kits (Qiagen, Germantown, MD, USA). The reactions were assessed for fragmentation and cDNA synthesis. cDNAs from the reaction mix were separated by using QIAseq Beads (Qiagen, Germantown, MD, USA). Ligation of the cDNA libraries was performed by using Indexing adapters. Subsequently, Bioanalyzer (Agilent 2100, Santa, Clara, CA, USA) and the DeNovix fluorometer (DeNovix, Wilmington, DE, USA) were used for quality and quantity assessments of all cDNA libraries. The resulting cDNA sequencing libraries were pooled in equimolar quantities and subjected to cluster generation and paired-end 150-nucleotide read sequencing on an Illumina HiSeq sequencer (Illumina Inc., San Diego, CA, USA) at the Omics Sciences and Bioinformatics Center, Bangkok, Thailand. The obtained final sequence reads were subjected to bioinformatics analyses. Raw-read RNA sequence data files were used for quality control using FASTQC software (version 0.11.9) [http://www.bioinformatics.babraham.ac.uk/projects/fastqc]. The FastP software (version 0.23.1) [https://doi.org/10.1093/bioinformatics/bty560] was used to filter raw data by removing the adapter and low-quality sequences to obtain clean reads. Afterwards, the sequence read files were mapped to reference mouse genome:mm39 (2020) using STAR software (version 2.7.10b) [https://doi.org/10.1093/bioinformatics/bts635] with the default parameters [60]. The read counts of each gene were counted by using HTseq software (version 2.0.2) [https://doi.org/10.1093/bioinformatics/btac166]. EdgeR package (version 3.4.0) [https://doi.org/10.1093/bioinformatics/btp616] was used to filter, normalize, and define DEGs between WT and *Mx1;TβRI^CA^* groups. For normalization of gene expression, the “Trimmed Mean of M-values” (TMM) normalization method in the edgeR package was used. False discovery rate (FDR) and log fold change (logFC) were used to determine significant genes. If the threshold value of FDR was less than 0.05 and logFC was more than 0, these genes can be considered upregulated genes. When the FDR value was less than 0.05 and the logFC value was less than 0, these genes are listed as downregulated genes. The resulting upregulated and downregulated genes were used for further analysis.

For qPCR analysis, RNA yields were measured with a NanoDrop 2000 machine (Thermo Fisher Scientific, Waltham, MA, USA). After that, first-strand cDNA was reversely transcribed from 1 µg of individual total RNA using the SuperScript VILO cDNA synthesis kit (Invitrogen, Carlsbad, CA, USA). The qPCR was performed in the Luna Universal qPCR master mix (New England Biolabs, Ipswich, MA, USA) using the CFX96™ Optics Module (Bio-Rad, Hercules, CA, USA). *Gapdh* expression was used as a reference for gene expression normalization. All primer sequences for qPCR analysis are listed in Supplementary Table S6. Three or four independent assays were carried out for each experiment.

### 4.3. Osteoclast Culture

Briefly, bone marrow cells from long bones were flushed out using α-MEM medium, filtered with a 40 μm filter, and centrifuged. Cells were cultured in α-MEM medium supplemented with 10% FBS and 1% penicillin–streptomycin in an incubator overnight. To generate bone marrow macrophages (BMMs), non-adherent cells were cultured in the same medium by adding 20 ng/mL of M-CSF (R&D Systems, Inc., Minneapolis, MN, USA) for 48 h. siRNA transfection was carried out followed by osteoclast differentiation and qPCR analysis. BMMs were transfected twice with *siLilrb4* and siControl. Transfected BMMs were cultured in the medium containing 20 ng/mL of M-CSF and 3.3 ng/mL of RANKL (R&D Systems, Inc., MN, USA). For tartrate-resistant acid phosphatase (TRAP) staining, cells were washed twice with PBS, fixed, and then stained using Fast Red Violet LB Salt (Sigma-Aldrich, Burlington, MA, USA). TRAP-positive osteoclasts with more than 5 nuclei were counted using OsteoMeasure software and then the osteoclast number per total area (N.Oc/Ar) was analyzed. For RNA isolation and qPCR analysis, osteoclasts were stored at −80 °C until further analysis.

### 4.4. Osteoblast Culture

Long bones without bone marrow were minced into small pieces and digested by collagenase type II (Worthington Biochemical Corporation, Lakewood, NJ, USA) for 2 h at 37 °C and centrifuged. After removing the supernatant, the bone fragments were cultured in α-MEM medium supplemented with 20% FBS and 1% penicillin–streptomycin in an incubator until the cells became confluent. Osteoblasts were plated in the same medium for 48 h. siRNA transfection was carried out followed by osteogenic differentiation and qPCR analysis. Osteoblasts were transfected twice with *siHdac7* and siControl. Transfected osteoblasts were cultured with differentiation media containing 5 mM β-glycerophosphate, 50 μg/mL of ascorbic acid, and 10 μM dexamethasone. On days 7 and 21, alkaline phosphatase (ALP) and mineralization assays were carried out, respectively. ALP activity was measured with the alkaline phosphatase assay kit (ab83369, Abcam, Cambridge, UK) and osteoblasts were stained with Fast Blue RR (Sigma, St. Louis, MO, USA). For detecting mineralization nodules, 2% alizarin red (Sigma, St. Louis, MO, USA) was used, the mineralized nodules were destained with 10% cetylpyridinium chloride in 10 mM sodium phosphate, and the mineralization was measured. For RNA isolation and qPCR analysis, osteoblasts were stored at −80 °C until further analysis.

### 4.5. siRNA Transfection

For the siRNA transfection experiment, Silencer Select *Lilrb4* and *Hdac7* siRNA (Thermo Fisher Scientific, Waltham, MA, USA) were used to transfect twice in BMMs and osteoblasts, respectively, using Lipofectamine 3000 (Invitrogen, Carlsbad, CA, USA) with serum-free Opti-MEM medium (Thermo Fisher Scientific, Waltham, MA, USA) as per the manufacturer’s instructions. The Silencer Select Negative Control No. 1 siRNA (Thermo Fisher Scientific, Waltham, MA, USA) was used as a negative control. The efficiency of gene knockdown was quantified by qPCR analysis and *Gapdh* expression was used as a reference for gene expression normalization.

### 4.6. Identification of Differentially Expressed Genes (DEGs)

After data normalization, upregulated and downregulated DEGs between WT and *Mx1;TβRI^CA^* groups were used to render a volcano plot, and a principal component analysis (PCA) plot was generated by using ggplot2 and heatmap in the pheatmap (version 1.0.12 packages of R language (version 4.2.0) [https://www.R-project.org/].

### 4.7. Functional Enrichment Analysis

Kyoto Encyclopedia of Genes and Genomes (KEGG) pathways were also performed using the Cytoscape plug-in clueGO (version 2.5.10) to determine the significant pathways of the gene set [61].

### 4.8. Protein–Protein Interaction (PPI) Network Construction

The Search Tool for the Retrieval of Interacting Genes (STRING) database (version 12) [https://doi.org/10.1093/nar/gkac1000] was used as a resource for protein–protein interaction among DEGs with a combined medium default score of 0.4. In addition, the STRING database was used for detecting the association and interaction of *Lilrb4* and *Hdac7* in humans and in mice.

### 4.9. Serum Chemistry

A multiplex bead-based assay (LEGENDplex^TM^, BioLegend, San Diego, CA, USA) was used to determine mouse serum IFN-β, IFN-γ, TNF-α, MCP-1, IL-1α, IL-1β, IL-6, IL-10, IL-12p70, IL-17A, IL-23, IL-27, and GM-CSF levels according to the manufacturer’s instructions.

### 4.10. Statistical Analysis

All statistical analyses were performed using SPSS 29 (IBM, Armonk, NY, USA). The differences between two groups were compared using an independent student’s *t*-test and more than two groups were compared using one-way ANOVA followed by Fisher’s protected least significant difference test. Two-way ANOVA was used for interactions between TβRI overexpression and *siLilrb4* or *siHdac7*. Data are expressed as mean ± SEM. *p*-values < 0.05 were considered statistically significant.

## Data Availability

All data are included in this article and its Supplementary Materials.

## Supplementary Materials

The following supporting information can be downloaded at https://www.mdpi.com/article/10.3390/ijms25115829/s1.
