# Supplementary material for: Differential Gene Expression Involved in Bone Turnover of Mice Expressing Constitutively Active TGFβ Receptor Type I
Source: Int J Mol Sci. 2024 May 27;25(11):5829. doi: 10.3390/ijms25115829 (PMC11173332; doi:10.3390/ijms25115829)
Supplement: Supplementary file 1 [file ijms-25-05829-s001.zip › ijms-2991170-supplementary.pdf]

Differential Gene Expression Involved in Bone Turnover of Mice expressing Constitutively Active TGFβ Receptor Type I

Ohnmar Myint[1], Nithidol Sakunrangsit[1], Jatuphol Pholtaisong[1,2], Parichart Toejing[1], Pinyada Pho-on[1], Asada Leelahavanichkul[3], Somyoth Sridurongrit[4], Chatchawit Aporntewan[5], Matthew B. Greenblatt[6,7], and Sutada Lotinun[1,*]

[1]Center of Excellence in Skeletal Disorders and Enzyme Reaction Mechanism, Department of Physiology, Faculty of Dentistry, Chulalongkorn University, Bangkok 10330, Thailand

[2]Department of Biotechnology, Faculty of Science and Technology, Thammasat University (Rangsit Center), Pathum Thani, 12120, Thailand
[3]Division of Immunology, Department of Microbiology, Faculty of Medicine, Chulalongkorn University, Bangkok 10330, Thailand
[4]Department of Anatomy, Faculty of Science, Mahidol University, Bangkok 10400, Thailand
[5]Department of Mathematics and Computer Science, Faculty of Science, Chulalongkorn University, Bangkok 10330, Thailand
[6]Department of Pathology and Laboratory Medicine, Weill Cornell Medicine, and New York, NY 10065, USA
[7]Research Division, Hospital for Special Surgery, New York, NY 10065, USA

*Address correspondence to:
Sutada Lotinun, Ph.D.
Department of Physiology
Faculty of Dentistry
Chulalongkorn University
Bangkok, Thailand
Tel: +662 218-8557
Fax: +662 218-8691
Email: sutada.l@chula.ac.th

Short title: RNA sequencing: TGFβI receptor overexpression induced bone turnover in mice.

Key words: TGF-β; bone turnover; bioinformatics; siRNA; osteoclasts; osteoblasts

Supplementary Table S1. Functional roles of upregulated DEGs which showed a significant increased expression in qPCR.

Gene symbol and ID	Full name	Functions
Pdzk1 (ID 59020)	PDZ domain containing 1	*Pdzk1* is a scaffold protein and can bind with various transporters and regulates their functions [13].
Vpreb1a (ID 22362)	V-set pre-B cell surrogate light chain 1A	*Vpreb1a* is a member of immunoglobulin superfamily and involves in B cell development [14].
Cd72 (ID 12517)	CD72 antigen	*Cd72* is a member of calcium-dependent C-type lectin superfamily. It functions as a co-receptor of B cell receptor and is involved in B cell development [15].
Igll1 (ID 16136)	Immunoglobulin lambda-like polypeptide 1	*Igll1* is an early B cell protein and known as λ5. It is a component of preB cell receptor and is involved in early preB cell development. It plays an important role in bone formation. $\lambda5^{-/-}$ mice had decreased trabecular bone mass [16].
Tnfrsf19 (ID 29820)	Tumor necrosis factor receptor superfamily, member 19	*Tnfrsf19* is a member of tumor necrosis factor receptor superfamily. It acts as cell membrane receptor. It plays an important role in bone formation and is involved in osteoblast differentiation by regulating *Runx2* [17].
Gas6 (ID 14456)	Growth arrest specific 6	*Gas 6* is a member of the vitamin K-dependent proteins family. It acts as a ligand for receptors such as Tyro3, Axl, MerTK. It is involved in regulation of osteoclast function by binding with the receptor Tyro3 [18].
Zdhhc2 (ID 70546)	Zinc finger, DHHC domain containing 2	*Zdhhc2* is a protein coding gene. It has a positive regulation in osteoblast differentiation [19].
Igfbp4 (ID 16010)	Insulin-like growth factor binding protein 4	*Igfbp4* is produced by osteoblast. It inhibits osteoblastic cell differentiation [20]. *Igfbp4* knockouts had increased osteoclast numbers which means that it has a negative regulation on osteoclastogenesis [21].
Hdac7 (ID 56233)	Histone deacetylase 7	*Hdac7* is a part of histone deacetylase complex. It is a negative regulator of bone resorption. Silencing *Hdac7* enhances osteoclast numbers [22-24]. It also suppresses *Runx2* expression and osteoblast formation [25].

Supplementary Table S2. Functional roles of downregulated DEGs which showed a significant decreased expression in qPCR.

**Gene symbol and ID**	**Full name**	**Function**
Dhcr24 (ID 74754)	24-dehydrocholesterol reductase	*Dhcr24* is involved in the process of cholesterol synthesis and lipid metabolism which in turn involves differentiation of osteoclasts [26].
Padi4 (ID 18602)	Peptidyl arginine deiminase, type IV	*Padi4* is an enzyme involved in protein citrullination. It functions as a binding partner of *Runx2* and is involved in osteoblast mineralization [27].
Gsr (ID 14782)	Glutathione reductase	*Gsr* is an enzyme which is a member of the class-I pyridine nucleotide-disulfide oxidoreductase family. It is involved in cellular redox homeostasis. Increasing in sirtuin 1 (*Sirt1*) expression in mice could increase *Gsr* level and activates the osteoblastic bone formation [28].
Clec4d (ID 17474)	C-type lectin domain family 4, member d	*Clec4d*, macrophage C-type lectin, is a member of C-type lectin domain family. It expresses in inflammatory condition. It acts as a working partner of *Clec4e* (Mincle) [29].
Lilrb4a (ID 14728)	Leukocyte immunoglobulin-like receptor, subfamily B, member 4A	*Lilrb4a* is a member of the leukocyte immunoglobulin-like receptor family. It is involved in immune response. It is a negative regulator of osteoclast differentiation and inhibits osteoclast differentiation [30].
Clec4e (ID 56619)	C-type lectin domain family 4, member e	*Clec4e*, also known as Mincle, is a member of the C-type lectin domain family. It is involved in immune response. It stimulates osteoclast resorption. It is the positive regulation in osteoclast formation. Deletion of *Clec4e* can cause a decrease in osteoclast numbers [31].
Fcgr4 (ID 246256)	Fc receptor, IgG, low affinity IV	*Fcgr4* is a member of the immunoglobulin superfamily. It acts as cell membrane receptor. It is a positive regulator of bone resorption. Silencing of *Fcgr4* could decrease osteoclast numbers and TRAP+ cells [32].
Stfa2 (ID 20862)	Stefin A2	*Stfa2* is an intracellular inhibitor of cysteine proteases and negative regulator of endopeptidase activity. Recombinant Stfa2 protein can inhibit osteogenic differentiation *in vitro* [33].

Olfm4 (ID 380924)	Olfactomedin 4	It acts as an antiapoptotic protein and is involved in cell adhesion. It is one of the hub genes involved in subchondral bone osteoarthritis [34].
Cxcr1 (ID 227288)	Chemokine (C-X-C motif) receptor 1	It acts as a cell surface receptor. Interlukin-8 (IL-8) is the ligand for *Cxcr1* where they work together and have pro-osteoclastic effect [35].

Supplementary Table S3. Proinflammatory cytokines serum levels in *Mx1;TβRI*^{CA} female mice and their controls.

Parameters (pg/ml)	7-weeks old	
	WT (n=5)	*Mx1;TβRI*^{CA} (n=5)
IFN-β	233.80±14.93	211.16±11.45
IFN-γ	91.29±5.38	120.32±11.91*
TNF-α	189.71±4.97	208.22±8.04
MCP-1	42.49±1.07	45.6±1.31
IL-1α	12.12±1.86	18.47±1.88*
IL-1β	52.22±6.15	126.85±14.71*
IL-6	27.00±0.87	45.09±0.82*
IL-10	195.26±14.1	122.26±17.23*
IL-12p70	20.47±1.45	30.13±4.87
IL-17A	37.58±2.52	48.00±6.54
IL-23	1368.92±82.47	1912.88±163.31*
IL-27	409.16±37.37	552.6±26.01*
GM-CSF	26.6±1.39	38.99±5.22

*p<0.05 versus WT controls, unpaired *t*-test.

Supplementary Table S4. Osteoclast marker genes and osteoclast numbers in osteoclast cells transfected with *siLilrb4*.

Parameters	WT		*Mx1;TβRI*^{CA}		Two-way ANOVA		
	siControl (n=3-4)	*siLilrb4* (n=3-4)	siControl (n=3-4)	*siLilrb4* (n=3-4)	*Mx1;TβRI*^{CA}	*siLilrb4*	Interaction
Lilrb4	1.00±0.06	0.57±0.06[a]	0.61±0.08[a]	0.25±0.05[abc]	$p < 0.05$	$p < 0.05$	NS
Acp5	1.00±0.34	2.33±0.18[a]	3.15±0.62[a]	4.46±0.37[abc]	$p < 0.05$	$p < 0.05$	NS
Ctsk	1.00±0.19	2.32±0.46[a]	2.99±0.40[a]	5.88±0.53[abc]	$p < 0.05$	$p < 0.05$	NS
Nfatc1	1.00±0.19	2.10±0.13[a]	2.07±0.08[a]	2.97±0.11[abc]	$p < 0.05$	$p < 0.05$	NS
Osteoclast numbers	2.04±0.19	3.79±0.23[a]	3.31±0.59[a]	6.94±0.37[abc]	$p < 0.05$	$p < 0.05$	$p < 0.05$

[a]p<0.05 compared with WT siControl, One-way ANOVA followed by Fisher's protected LSD test
[b]p<0.05 compared with WT *siLilrb4*
[c]p<0.05 compared with *Mx1;TβRI*^{CA} siControl

Supplementary Table S5. Osteoblast marker genes, ALP activity and mineralization in osteoblast cells transfected with *siHdac7*.

Parameters	WT		*Mx1;TβRI*^{CA}		Two-way ANOVA		
	siControl (n=3-4)	*siHdac7* (n=3-4)	siControl (n=3-4)	*siHdac7* (n=3-4)	*Mx1;TβRI*^{CA}	*siHdac7*	Interaction
Hdac7	1.00±0.17	0.34±0.04[a]	1.65±0.49[ab]	0.30±0.12[ac]	*NS*	$p < 0.05$	NS
Sp7	1.00±0.09	1.71±0.09[a]	0.73±0.08[ab]	1.48±0.05[ac]	$p < 0.05$	$p < 0.05$	NS
Alpl	1.00±0.06	1.52±0.03[a]	0.70±0.11[ab]	1.42±0.11[ac]	NS	$p < 0.05$	NS
Runx2	1.00±0.15	1.54±0.20[a]	0.50±0.13[ab]	1.47±0.11[ac]	NS	$p < 0.05$	NS
Wnt3a	1.00±0.11	1.67±0.10[a]	0.63±0.05[ab]	1.13±0.07[bc]	$p < 0.05$	$p < 0.05$	NS
Gli1	1.00±0.03	1.58±0.16[a]	0.61±0.08[ab]	0.99±0.07[bc]	$p < 0.05$	$p < 0.05$	NS
ALP activity (U/mL)	28.30±0.75	31.05±0.003[a]	24.78±0.91[ab]	30.14±0.95[c]	$p < 0.05$	$p < 0.05$	NS
Mineralization (mM)	2.66±0.41	3.48±0.07[a]	1.36±0.19[ab]	2.39±0.40[bc]	$p < 0.05$	$p < 0.05$	NS

[a]$p<0.05$ compared with WT siControl, One-way ANOVA followed by Fisher's protected LSD test
[b]$p<0.05$ compared with WT *siHdac7*
[c]$p<0.05$ compared with *Mx1;TβRI*^{CA} siControl

Supplementary Table S6. Primer sequences for qPCR.

Gene	Forward	Reverse
Pdzk1	5′-CACCTTCAACCCCAGAGAGT-3′	5′-TGATCCTGAGCACCCTGTCA-3′
Igf2bp3	5′-CTGCCCCACGTAGTGTGATA-3′	5′-ATGGAGCCGGTGGATTTTGT-3′
Vpreb1a	5′-GGGAGTGGGAAGGAGAAAAG-3′	5′-AAAACCTCACGTTCTACCCC-3′
Vpreb1b	5′-AGCTAGGCCCCCTGCTATTT-3′	5′-AAAGTCATGGGCCAGAACCC-3′
Dbp	5′-GGTCCCCAAATGCGAAAGGA-3′	5′-GCTCGCTCCCATTTTTGGTG-3′
Cd72	5′-TGTGAAAGTGCCCCTGAAG-3′	5′-GGTGAGTTCCCCATCTTCATAG-3′
Igll1	5′-TGCTGCTGTTGGGTCTAGTG-3′	5′-AGGGACCCCATCTACCTTCC-3′
Ptx3	5′-CGTGCATCCTGTGAGACCAA-3′	5′-TAGGGGTTCCACTTTGTGCC-3′
Gdf10	5′-CTGCCCACACTTTTCAAGGC-3′	5′-TGGGATCGACTCTCTCCGTT-3′
Tnfrsf19	5′-TCTGGTAGGGACTTCGTGCT-3′	5′-GTTCGCTACCGTCTGGTTCT-3′
Gas6	5′-ATGAAGATCGCGGTAGCTGG-3′	5′-CCAACTCCTCATGCACCCAT-3′
Zdhhc2	5′-CCAGCTGTGCATAGTGTCCA-3′	5′-TCTCCAGCAATTCTTTCTCTGC-3′
Slc20a2	5′-GAGGGAACGAGAAGCCAGAA-3′	5′-TGAGTCTGCTCTGGAAAGCG-3′
Igfbp4	5′-TGTGGGAAAGGAATGGGGTG-3′	5′-GGAAATATGGGGACGGAGGC-3′
Foxp4	5′-GATCGGAAGCTGGAGAGAGC-3′	5′-GCCTGTTGCTGTTGGAAGTG-3′
Hdac7	5′-ACAGAAACCCAACCTCAGTG-3′	5′-ATGCAGCCCCAGTATTTCC-3′
Tnfsf14	5′-CCATCCGCTGCATTGGTTTC-3′	5′-ATTGTGCCTTCCCACGATCC-3′
Trpc6	5′-GCTCTCATATACTGGTGTGCTC-3′	5′-GCTTGGTGCCTTCAAATCTG-3′
Pde5a	5′-GGCAAGCACCATGGAACGAG-3′	5′-GAGCACTGGTCCCCTTCATC-3′
Dhcr24	5′-GATGAATGGTCAACGCGAGC-3′	5′-TTTGGGTGACACAGGTTGCT-3′
Padi4	5′-CCTACAGGTGAAAGCAGCCA-3′	5′-TCAAAGTCCATTCCGGAGGC-3′
Gsr	5′-TGGCACTTGCGTGAATGTTG-3′	5′-CGAATGTTGCATAGCCGTGG-3′
Ldhd	5′-GAGGCTCTGAAGGCAGTTGT-3′	5′-CTGTCACGCAGGTGGGTATT-3′
Clec4d	5′-CGACATCCCCAACTGATCCC-3′	5′-CGGATGCACGTTACTCTCGT-3′
Lilrb4a	5′-GCTACTTTAGAAATGAACCACAGG-3′	5′-AATGACACCAGGACTCCAATC-3′
Chit1	5′-CCCGTCAGAGGAGAGCAGAT-3′	5′-GTACCAAAGGTCCAGCCTCC-3′
Hspb7	5′-ACTAACAGTGCCAGGTGGTG-3′	5′-CCCCTTGTCTGCTGGCTAAA-3′

Cox8b	5′-AGAATCATGCCAAGGCTCCC-3′	5′-TGGAACCATGAAGCCAACGA-3′
Clec4e	5′-ATGGAAAGGCCGGTCAAACT-3′	5′-GAAGTGCTCGTAATGAGTGCTA-3′
Thbs4	5′-TGTGCGCTGTGTGAATTTGG-3′	5′-CATGGGTTCTGCTCTGGGTT-3′
Fcgr4	5′-CACCATGCTTTGAGCAGCAG-3′	5′-GAATCGCTTCTTGGGAGGGT-3′
Stfa2	5′-AATTGGAGGTTTGTCAGAGGC-3′	5′-CTCTTTACAATGGGGGTTAGGG-3′
Olfm4	5′-GGCACGATGAGTTACAGCCT-3′	5′-GGTGATGTTGGAGGTGTGGT-3′
Cxcr1	5′-CTATGCTGGTCTGCTACGGG-3′	5′-TTCTCTTTGAGGGCAAGCGA-3′
Acp5(TRAP)	5′-GATCCCTCTGTGCGACATCA-3′	5′-CCAGGGAGTCCTCAGATCCA-3′
Ctsk	5′-AGGCATTGACTCTGAAGATGCT-3′	5′-TCCCCACAGGAATCTCTCTG-3′
Nfatc1	5′-AGGCTGGTCTTCCGAGTTCA-3′	5′-ACCGCTGGGAACACTCGAT-3′
Sp7(Osx)	5′-CCCTTCTCAAGCACCAATGG-3′	5′-AAGGGTGGGTAGTCATTTGCATA-3′
Alpl	5′-CTTGACTGTGGTTACTGCTGATCA-3′	5′-GTATCCACCGAATGTGAAAACGT-3′
Runx2	5′-AGTCCCAACTTCCTGTGCTCC-3′	5′-CGGTAACCACAGTCCCATCTG-3′
Wnt3a	5′-TGGCAGAATGAGGCATGGAG-3′	5′-TCAGGAAAGCTCTGCCAAGG-3′
Gli1	5′-ACCCGGGATACAACCCAAAT-3′	5′-AGACCATTGCCCATCACAGA-3′
Gapdh	5′-TGCACCACCAACTGCTTAG-3′	5′-GGATGCAGGGATGATGTTC-3′

References

13. Higashino, T.; Matsuo, H.; Sakiyama, M.; Nakayama, A.; Nakamura, T.; Takada, T.; Ogata, H.; Kawamura, Y.; Kawaguchi, M.; Naito, M.; et al. Common variant of PDZ domain containing 1 (PDZK1) gene is associated with gout susceptibility: A replication study and meta-analysis in Japanese population. *Drug Metab Pharmacokinet* **2016**, *31*, doi:10.1016/j.dmpk.2016.07.004.
14. Mårtensson, I.-L.; Ceredig, R. Role of the surrogate light chain and the pre-B-cell receptor in mouse B-cell development. *Front Immunol* **2000**, *101*, 435-441, doi:10.1046/j.1365-2567.2000.00151.x.
15. Shen, Y.; Ma, Y.; Xie, J.; Lin, L.; Shi, Y.; Li, X.; Shen, P.; Pan, X.; Ren, H. A regulatory role for CD72 expression on B cells and increased soluble CD72 in primary Sjogren's syndrome. *BMC Immunol* **2020**, *21*, 21, doi:10.1186/s12865-020-00351-2.
16. Khass, M.; Rashid, H.; Burrows, P.D.; Javed, A.; Schroeder, H.W. Loss of early B cell protein λ5 decreases bone mass and accelerates skeletal aging. *Front Immunol* **2022**, *13*, 906649, doi:10.3389/fimmu.2022.906649.
17. Wu, H.; Whitfield, T.W.; Gordon, J.A.; Dobson, J.R.; Tai, P.W.; van Wijnen, A.J.; Stein, J.L.; Stein, G.S.; Lian, J.B. Genomic occupancy of Runx2 with global expression profiling identifies a novel dimension to control of osteoblastogenesis. *Genome Biol* **2014**, *15*, R52, doi:10.1186/gb-2014-15-3-r52.
18. Xue, J.; Xu, L.; Zhu, H.; Bai, M.; Li, X.; Zhao, Z.; Zhong, H.; Cheng, G.; Li, X.; Hu, F.; et al. CD14(+)CD16(-) monocytes are the main precursors of osteoclasts in rheumatoid arthritis via expressing Tyro3TK. *Arthritis Res Ther* **2020**, *22*, 221, doi:10.1186/s13075-020-02308-7.
19. Al Saedi, A.; Myers, D.E.; Stupka, N.; Duque, G. 1,25(OH)(2)D(3) ameliorates palmitate-induced lipotoxicity in human primary osteoblasts leading to improved viability and function. *Bone* **2020**, *141*, 115672, doi:10.1016/j.bone.2020.115672.
20. Mohan, S.; Nakao, Y.; Honda, Y.; Landale, E.; Leser, U.; Dony, C.; Lang, K.; Baylink, D.J. Studies on the mechanisms by which insulin-like growth factor (IGF) binding protein-4 (IGFBP-4) and IGFBP-5 modulate IGF actions in bone cells (∗). *J Biol Chem* **1995**, *270*, 20424-20431, doi:10.1074/jbc.270.35.20424.
21. Maridas, D.E.; DeMambro, V.E.; Le, P.T.; Nagano, K.; Baron, R.; Mohan, S.; Rosen, C.J. IGFBP-4 regulates adult skeletal growth in a sex-specific manner. *J Endocrinol* **2017**, *233*, 131-144, doi:10.1530/joe-16-0673.
22. Jin, Z.; Wei, W.; Dechow, P.C.; Wan, Y. HDAC7 inhibits osteoclastogenesis by reversing RANKL-triggered β-catenin switch. *Mol Endocrinol* **2013**, *27*, 325-335, doi:10.1210/me.2012-1302.
23. Stemig, M.; Astelford, K.; Emery, A.; Cho, J.J.; Allen, B.; Huang, T.H.; Gopalakrishnan, R.; Mansky, K.C.; Jensen, E.D. Deletion of histone deacetylase 7 in osteoclasts decreases bone mass in mice by interactions with MITF. *PLoS One* **2015**, *10*, e0123843, doi:10.1371/journal.pone.0123843.
24. Pham, L.; Kaiser, B.; Romsa, A.; Schwarz, T.; Gopalakrishnan, R.; Jensen, E.D.; Mansky, K.C. HDAC3 and HDAC7 have opposite effects on osteoclast differentiation. *J Biol Chem* **2011**, *286*, 12056-12065, doi:10.1074/jbc.M110.216853.

25. Jensen, E.D.; Schroeder, T.M.; Bailey, J.; Gopalakrishnan, R.; Westendorf, J.J. Histone deacetylase 7 associates with Runx2 and represses its activity during osteoblast maturation in a deacetylation-independent manner. *J Bone Miner Res* **2008**, *23*, 361-372, doi:10.1359/jbmr.071104.
26. Kim, H.; Oh, B.; Park-Min, K.H. Regulation of osteoclast differentiation and activity by lipid metabolism. *Cells* **2021**, *10*, doi:10.3390/cells10010089.
27. Zhai, Q.; Zhao, Y.; Wang, L.; Dai, Y.; Zhao, P.; Xiang, X.; Liu, K.; Du, W.; Tian, W.; Yang, B.; et al. CircRNA hsa_circ_0008500 acts as a miR-1301-3p sponge to promote osteoblast mineralization by upregulating PADI4. *Front Cell Dev Biol* **2020**, *8*, doi:10.3389/fcell.2020.602731.
28. Sun, W.; Qiao, W.; Zhou, B.; Hu, Z.; Yan, Q.; Wu, J.; Wang, R.; Zhang, Q.; Miao, D. Overexpression of Sirt1 in mesenchymal stem cells protects against bone loss in mice by FOXO3a deacetylation and oxidative stress inhibition. *Metabolism* **2018**, *88*, 61-71, doi:10.1016/j.metabol.2018.06.006.
29. Kerscher, B.; Wilson, G.J.; Reid, D.M.; Mori, D.; Taylor, J.A.; Besra, G.S.; Yamasaki, S.; Willment, J.A.; Brown, G.D. Mycobacterial receptor, Clec4d (CLECSF8, MCL), is coregulated with Mincle and upregulated on mouse myeloid cells following microbial challenge. *Eur J Immunol* **2015**, *46*, 381 - 389.
30. Mori, Y.; Tsuji, S.; Inui, M.; Sakamoto, Y.; Endo, S.; Ito, Y.; Fujimura, S.; Koga, T.; Nakamura, A.; Takayanagi, H.; et al. Inhibitory immunoglobulin-like receptors LILRB and PIR-B negatively regulate osteoclast development. *J Immunol* **2008**, *181*, 4742-4751, doi:10.4049/jimmunol.181.7.4742.
31. Andreev, D.; Liu, M.; Weidner, D.; Kachler, K.; Faas, M.; Grüneboom, A.; Schlötzer-Schrehardt, U.; Muñoz, L.E.; Steffen, U.; Grötsch, B.; et al. Osteocyte necrosis triggers osteoclast-mediated bone loss through macrophage-inducible C-type lectin. *J Clin Invest* **2020**, *130*, 4811-4830, doi:10.1172/jci134214.
32. Negishi-Koga, T.; Gober, H.J.; Sumiya, E.; Komatsu, N.; Okamoto, K.; Sawa, S.; Suematsu, A.; Suda, T.; Sato, K.; Takai, T.; et al. Immune complexes regulate bone metabolism through FcRγ signalling. *Nat Commun* **2015**, *6*, 6637, doi:10.1038/ncomms7637.
33. Patra, D.; Kim, J.; Zhang, Q.; Tycksen, E.; Sandell, L.J. Site-1 protease ablation in the osterix-lineage in mice results in bone marrow neutrophilia and hematopoietic stem cell alterations. *Biol Open* **2020**, *9*, doi:10.1242/bio.052993.
34. Yang, Z.; Ni, J.; Kuang, L.; Gao, Y.; Tao, S. Identification of genes and pathways associated with subchondral bone in osteoarthritis via bioinformatic analysis. *Medicine (Baltimore)* **2020**, *99*, e22142, doi:10.1097/md.0000000000022142.
35. Humbert, P.; Brennan, M.; De Lima, J.; Brion, R.; Adrait, A.; Charrier, C.; Brulin, B.; Trichet, V.; Couté, Y.; Blanchard, F.; et al. Apoptotic mesenchymal stromal cells support osteoclastogenesis while inhibiting multinucleated giant cells formation in vitro. *Sci Rep* **2021**, *11*, 12144, doi:10.1038/s41598-021-91258-4.

STAR edgeR results

gene	logFC	logCPM	PValue	FDR
Gsr	-1.4843419	7.9382221	9.12E-21	1.42E-16
LOC118568312	2.7549136	3.9394045	1.61E-12	1.25E-08
Rnf144a	-1.2921309	5.7530134	1.45E-10	6.00E-07
Padi4	-0.826089	6.554952	1.54E-10	6.00E-07
Ankrd22	-0.8749041	4.7068037	7.16E-10	2.23E-06
H4c18	4.3093645	-0.4453594	1.93E-09	4.99E-06
5430403N17Rik	4.6327517	-0.5503611	2.94E-09	6.54E-06
Cd300lb	-1.0327733	5.6833124	5.28E-09	1.03E-05
Asah1	-0.8151864	6.4449185	5.97E-09	1.03E-05
Lilrb4a	-0.7780084	6.949586	7.39E-09	1.15E-05
Trib2	1.0991548	5.5162643	1.20E-08	1.70E-05
Igkv10-94	-1.6110943	3.4450333	1.37E-08	1.77E-05
Xirp2	-4.7872004	1.595017	2.26E-08	2.71E-05
A530064D06Rik	-1.1818625	4.0370669	1.19E-07	0.0001324
Ly6g	-0.8875625	6.8341963	1.89E-07	0.0001961
Lilr4b	-1.0210496	5.0556487	2.42E-07	0.0002355
Retnlg	-1.1827903	9.7520073	4.36E-07	0.0003877
H1f5	3.9270337	0.9724864	4.49E-07	0.0003877
H1f1	3.3589536	-0.6125142	5.02E-07	0.0004113
H2ac21	4.8442967	-1.1221693	6.20E-07	0.0004819
Dntt	1.1692525	5.1965538	8.76E-07	0.0006387
Fcgr4	-0.8501588	4.1931175	9.03E-07	0.0006387
H2af-ps2	-1.0920998	3.4704023	1.05E-06	0.0007011
Cstdc6	-1.7054002	2.3655746	1.11E-06	0.0007011
Des	-2.5761255	2.5614642	1.13E-06	0.0007011
9830107B12Rik	-0.8650868	4.2444984	1.44E-06	0.0008592
Chchd10	0.7526558	5.8345892	1.52E-06	0.0008736
Hacd4	-0.6532668	5.6095197	2.38E-06	0.0013193
Anxa3	-0.7038011	6.9614772	2.52E-06	0.0013521
H4f16	2.9213395	-0.1490057	3.07E-06	0.0015674
Plpbp	0.6767062	4.7119432	3.12E-06	0.0015674
Gm38525	-0.8807321	3.5838625	3.36E-06	0.001631
Clec4e	-1.1236512	4.8755001	3.81E-06	0.0017954
Greb1	-2.5792627	1.1401312	5.05E-06	0.0023119
Rap1gap2	-0.708652	4.8904093	5.42E-06	0.002409
Thbs4	-3.4807057	1.9268796	5.72E-06	0.0024552
Trpc6	-0.6221587	4.6588155	5.84E-06	0.0024552
H1f3	2.6560963	1.6749332	6.00E-06	0.0024572
Limch1	1.1843573	5.9999879	6.49E-06	0.0024976
Clec4d	-1.0573038	5.3987966	6.52E-06	0.0024976

Olfml3	1.4100844	5.5497882	6.65E-06	0.0024976
Arvcf	0.905407	3.6813837	6.74E-06	0.0024976
Gm11847	3.2233086	3.1687893	7.59E-06	0.0027461
Hdc	-0.964332	6.4323361	8.32E-06	0.0029394
Igkv12-89	-1.746128	1.5650733	8.57E-06	0.0029638
Myo5c	-3.033897	-0.7409406	9.57E-06	0.0032355
Gm17803	-2.0518523	0.9059905	1.11E-05	0.0036831
Tifa	0.5694018	6.5024003	1.19E-05	0.0038406
Pilrb1	-0.773517	4.3216167	1.23E-05	0.0038938
B430306N03Rik	-0.6636668	5.9800729	1.29E-05	0.0039867
Sh3bgr	-2.3539578	-0.3626946	1.31E-05	0.0039867
Shank2	-2.1846773	1.3806004	1.40E-05	0.004141
Acpp	-1.0124085	4.6079098	1.41E-05	0.004141
Gm16793	-1.6679654	2.6815519	1.80E-05	0.0051588
Eno1b	4.1004904	2.7909527	1.82E-05	0.0051588
Asprv1	-0.9578287	4.7891887	1.95E-05	0.0054064
Ipcef1	-0.7002242	5.8061553	2.10E-05	0.005732
Cox6a2	-1.5057535	1.9981823	2.14E-05	0.0057506
Cox8b	-2.5532078	0.3237701	2.25E-05	0.0059218
Xrcc6	0.5074848	5.9632184	2.35E-05	0.0059588
Kcnh1	2.0195853	-0.2782865	2.37E-05	0.0059588
Pbx1	-0.6105417	6.168367	2.38E-05	0.0059588
Pdzk1	3.3776059	-0.4515199	2.48E-05	0.0061321
Tmem154	-0.7385803	5.8293837	2.53E-05	0.00614
Cstdc5	-1.3399342	4.2195402	2.81E-05	0.0067093
Pkig	0.554984	5.8843695	2.85E-05	0.0067093
Vpreb1	1.3739783	4.4093836	2.98E-05	0.0069156
Ryr1	-1.0974394	2.2697274	3.13E-05	0.0070543
Cxcr1	-2.9848427	0.4755521	3.13E-05	0.0070543
Hspb7	-2.3132136	0.0334851	3.23E-05	0.0071751
Fgd4	-0.908722	6.0716079	3.43E-05	0.0075101
Armc7	-0.651759	4.1367847	3.62E-05	0.0078122
Mctp1	-0.7219426	5.6131377	4.06E-05	0.00865
Mospd2	-0.5497034	5.0552307	4.27E-05	0.0089836
Fhl1	-0.6454084	5.2605287	4.37E-05	0.0090591
Zfp941	-1.6152087	-0.0518811	4.54E-05	0.0091821
Pilrb2	-0.6702491	4.6607045	4.55E-05	0.0091821
Olfm4	-1.4872576	6.7851145	4.67E-05	0.0093038
Mylpf	-1.5447924	4.5071888	4.79E-05	0.0093359
Cldn10	1.3713923	2.4131048	4.80E-05	0.0093359
Slc7a2	-0.9002056	3.1593666	4.98E-05	0.0095582
Srpk3	1.0697237	2.2350279	5.29E-05	0.0100278
Gm6197	1.2743949	5.7836528	5.45E-05	0.01019

Zdhhc2	1.1522206	2.1583815	5.60E-05	0.01019
Myh1	-3.9177752	2.6752369	5.60E-05	0.01019
Gm12057	1.1970404	1.691677	5.63E-05	0.01019
Thsd4	-0.8238572	3.5620206	5.87E-05	0.0105019
Obscn	-2.2773443	0.8524028	6.05E-05	0.0106981
H19	-2.8459541	1.8207998	6.31E-05	0.0110256
Nmral1	0.534792	5.2615664	6.57E-05	0.0111566
Gm38863	-1.2320587	0.8004399	6.63E-05	0.0111566
Smim41	2.7612932	-0.7385395	6.66E-05	0.0111566
Cd72	1.3630942	5.9429168	6.68E-05	0.0111566
Sapcd1	1.0428211	1.3368715	6.74E-05	0.0111566
Slfn4	-0.7098823	7.5865011	6.84E-05	0.0111921
Lilra6	-0.8085707	4.3112705	7.00E-05	0.01134
Pilra	-0.4523116	6.0329554	7.37E-05	0.0118142
Cyp2e1	-5.2294775	1.4252479	7.88E-05	0.0125137
Slc20a2	0.7689519	5.0113845	8.05E-05	0.01265
Ldhd	-1.4560556	0.1715357	8.17E-05	0.0127033
Trabd2b	1.2838305	1.2558031	8.62E-05	0.0132725
Prep	0.5188702	6.0334596	8.78E-05	0.0133815
Clec2f	-1.3483465	0.8999084	8.93E-05	0.0134808
Serpina1b	-4.1837615	1.9873069	9.76E-05	0.0144836
Il18rap	-0.7018187	5.9709045	9.78E-05	0.0144836
Ankrd33b	-0.7714708	4.2714418	0.0001007	0.0146074
Hdac7	0.5219813	5.4875527	0.0001022	0.0146074
Litaf	-0.5352994	6.5412967	0.0001027	0.0146074
Cd33	-0.5934184	6.1797501	0.0001031	0.0146074
Chit1	-0.9829662	4.2236876	0.0001033	0.0146074
Gm51802	1.3317904	1.7406345	0.0001073	0.0148475
Slc22a18	-1.8130534	-0.2782262	0.0001079	0.0148475
Fcgr3	-0.5357573	7.1035061	0.0001087	0.0148475
Acss1	0.5129826	6.4549842	0.0001091	0.0148475
Cd3d	-0.7315945	4.8595504	0.0001104	0.0148475
Eef1a2	-4.0046331	0.5512502	0.0001114	0.0148475
Tyrobp	-0.6166845	7.9693911	0.0001117	0.0148475
F630028O10Rik	-0.5098153	8.3084714	0.0001145	0.0150985
Gm36551	-0.8889318	3.0801927	0.0001191	0.0155634
D17H6S56E-5	0.6174535	8.7091756	0.0001217	0.0157742
Cstdc4	-1.2644018	4.5008119	0.0001325	0.0170336
Apobr	-0.5836477	6.6366269	0.000136	0.0173438
Tbc1d23	-0.5951435	5.7843695	0.0001405	0.0177012
Ptprcap	0.7225678	5.5186175	0.0001416	0.0177012
Thbs1	-0.5336493	9.3521489	0.0001423	0.0177012
Gask1a	1.3603732	0.8455068	0.0001534	0.0189331

Fgl2	-0.6919161	6.02534	0.0001587	0.0194333
Stfa2	-1.1049618	2.7434039	0.0001638	0.0199025
Bmx	-0.6688653	4.8121446	0.0001695	0.0203895
AU020206	0.9059489	5.0419449	0.0001704	0.0203895
Igkv12-46	-1.1402431	3.9009033	0.0001828	0.0217037
Celsr2	1.29137	1.1645945	0.0001857	0.0218809
Taok1	-0.4623346	7.2375247	0.0001877	0.0219457
Il1f9	-0.5608062	5.2391489	0.0001926	0.0222732
Myl4	1.1809964	4.1963191	0.0001933	0.0222732
Ldhc	-2.0262853	0.3353705	0.0001974	0.0225786
Itgam	-0.6354751	8.89838	0.0002025	0.0226984
Fnbp1l	-0.4569279	5.036012	0.0002035	0.0226984
Ldb3	-2.4556911	0.8830436	0.000204	0.0226984
Cers6	-0.5377689	6.1761521	0.0002043	0.0226984
Gm36741	-2.3015486	-0.7517428	0.0002081	0.0228168
Gramd4	0.4811776	5.4048187	0.0002083	0.0228168
Klf7	-0.484196	5.8429509	0.000213	0.0231672
Aldh1a2	-0.7607159	4.6648492	0.000221	0.0238524
Lyz2	-0.6906243	11.407412	0.0002224	0.0238524
Vpreb2	1.4925971	1.4919326	0.0002286	0.0243581
Myh2	-4.5987099	1.9957272	0.0002318	0.0245226
Alox5	-0.4827636	6.335118	0.0002445	0.0254451
Slc15a3	-0.633654	4.8776773	0.0002447	0.0254451
Igll1	1.2322984	4.6535478	0.0002454	0.0254451
Rag2	1.1908474	2.5118154	0.0002524	0.0259768
Dhcr24	-0.6558901	5.6581651	0.0002539	0.0259768
Chd7	-0.4364886	5.932499	0.000264	0.0265628
Mylf-ps	-3.3284191	2.8507535	0.0002646	0.0265628
Slfn1	-0.6399555	6.1168676	0.0002647	0.0265628
Mb	-3.8611052	3.1951165	0.0002692	0.0267883
Lrrk2	-0.5231378	6.6349862	0.000273	0.0267883
Glipr2	-0.5648381	6.4570365	0.000274	0.0267883
Mical2	-0.9931951	3.233707	0.0002747	0.0267883
Islr	0.8383865	5.6264632	0.0002756	0.0267883
Mmp8	-0.8218176	8.8358454	0.0002866	0.0276911
Trpm2	-0.6190776	5.3044723	0.0002907	0.0279146
Gdpd2	-0.5097109	5.6751115	0.0002931	0.0279689
Pard3b	-0.5972169	4.1282524	0.0002982	0.0282775
H2bc11	2.1265167	0.8785284	0.0003045	0.0286515
Enpp4	-0.5702335	4.9511487	0.000306	0.0286515
Ccr9	-1.1216666	1.412018	0.0003076	0.0286515
Slc2a3	-0.4754994	7.4390984	0.0003125	0.028815
S100a8	-0.6881812	12.675013	0.0003131	0.028815

Pi15	-1.3813087	3.5538599	0.0003192	0.0292023
Clec4b2	-0.8958472	2.3741297	0.0003233	0.0294045
2810408B13Rik	1.688654	1.8840089	0.0003264	0.0294917
Mocos	-0.6363492	4.3437649	0.0003284	0.0294917
Gdf10	1.1704604	3.1437434	0.0003299	0.0294917
Adpgk	-0.5821747	8.0917419	0.0003416	0.0301072
Bcl7a	0.8935281	6.263308	0.0003439	0.0301072
Cebpe	-0.4510407	6.7615854	0.0003446	0.0301072
Gm30996	1.2658045	2.2281811	0.0003452	0.0301072
Gm14548	-1.1777205	3.9429396	0.0003465	0.0301072
Pgam2	-2.3819375	1.3432318	0.0003561	0.0306055
Pde5a	-0.5840157	6.7979852	0.0003581	0.0306055
Eno3	-0.8056731	4.266364	0.0003599	0.0306055
Atp11a	-0.416862	6.2402936	0.0003601	0.0306055
Anxa1	-0.5709836	10.125217	0.0003644	0.0308053
2610005L07Rik	0.9257465	3.2233526	0.0003683	0.0309688
H3c13	2.0938787	-0.3936893	0.0003705	0.0309784
Camp	-0.6916446	10.874905	0.0003725	0.0309824
Fam160a2	-0.4638588	6.0117877	0.0003788	0.0313376
Fpr1	-0.5622492	5.4809808	0.0003828	0.0315026
Plek	-0.5823749	9.0487691	0.0004011	0.0325815
Sell	-0.7889606	7.4190202	0.0004018	0.0325815
Adgrg7	-1.0329227	3.3624271	0.0004026	0.0325815
Tnfrsf19	0.7712261	5.6027932	0.0004043	0.0325815
Tnni2	-2.3685366	3.4465529	0.0004082	0.0327249
Timp4	2.4295625	-0.6159192	0.000412	0.0328623
Entpd1	-0.5528758	5.4689877	0.0004191	0.0332585
Serpinb10	-0.8257577	5.4192418	0.0004276	0.0334846
Gas6	0.7462028	6.8827499	0.0004277	0.0334846
Igfbp4	0.6179874	8.5793571	0.0004284	0.0334846
Bfsp2	1.2951226	1.7521715	0.0004326	0.0334902
Cox7a1	-1.8333862	0.7279546	0.0004328	0.0334902
Nbeal2	-0.553745	6.5448612	0.0004375	0.0336888
Gm13453	-1.9279427	-0.0471195	0.0004407	0.0337671
Dmxl2	-0.4960826	6.0011166	0.000447	0.034083
Crebrf	-0.4610984	5.8825963	0.0004585	0.034785
Angptl1	-0.9179375	2.1788746	0.0004733	0.0357337
Kcnj2	-0.651886	4.4894718	0.000476	0.0357681
Evi2a	-0.4904552	4.9581034	0.0004787	0.0357991
Rhoh	0.6629129	4.3738338	0.0004818	0.0358575
Slamf6	0.8953996	4.1593597	0.0004906	0.0363364
AI429214	1.0851749	1.3367228	0.0004994	0.0364693
Aff2	-0.9503776	2.7837116	0.0004994	0.0364693

Pafah1b3	0.9909505	4.6179441	0.0005	0.0364693
H4c4	1.896944	0.5425778	0.0005018	0.0364693
S100a9	-0.6644731	13.844382	0.0005155	0.0372668
5830432E09Rik	-0.9840158	1.3624728	0.0005175	0.0372668
Rpl3l	-1.8049937	1.0839375	0.0005225	0.0374505
Mcemp1	-0.5351652	6.8171688	0.000538	0.0383847
Clec7a	-0.7194031	5.9557231	0.0005443	0.0386542
Vars	0.4603499	7.1787141	0.0005563	0.0392088
Acta1	-2.9520663	4.5824765	0.0005588	0.0392088
Car12	1.4438908	2.4411807	0.0005628	0.0392088
Tnfsf14	-0.4802634	4.655988	0.0005633	0.0392088
Tnnc2	-2.7683087	3.5123962	0.0005647	0.0392088
Cmya5	-1.4570225	2.003412	0.0005685	0.0392983
Actn3	-1.7701573	2.1972429	0.0005742	0.0395178
Slc35d2	-0.7907934	2.8308897	0.0005918	0.0403856
Zfp516	-0.4906667	5.4764859	0.0005946	0.0403856
Hsd11b1	-0.6195033	5.7447934	0.0005953	0.0403856
Gm30569	-1.3371027	1.7784278	0.0005972	0.0403856
H4c2	2.1073778	-0.0772923	0.0006007	0.0404479
Cd84	-0.4695121	6.9206868	0.0006036	0.0404668
Foxp4	0.6351933	5.0674142	0.0006096	0.0406969
E030007A22Rik	1.4655223	0.950167	0.0006178	0.0408154
Fcnb	-0.7017241	7.5437795	0.0006193	0.0408154
Ifi30	0.4409472	6.8673899	0.0006193	0.0408154
Cox6b2	0.6611875	4.6545205	0.0006317	0.0413157
Otud3	-0.4965074	4.4665529	0.000633	0.0413157
Ly75	-0.5606482	6.0679454	0.0006371	0.0413157
Ebf3	0.6351631	4.8507023	0.0006387	0.0413157
Clec4a2	-0.5650939	6.5274478	0.0006418	0.0413157
Ltf	-0.6157675	11.510474	0.0006439	0.0413157
H1f4	2.2975582	0.6509259	0.0006455	0.0413157
Lst1	-0.6164859	4.6686949	0.0006755	0.0430571
Myom1	-0.7371819	4.4668209	0.0006922	0.0437192
Zfp410	0.4666338	5.2939249	0.0006922	0.0437192
Ptpre	-0.508543	6.1210094	0.0006967	0.0437192
H2ac22	2.3255306	-0.5777618	0.0006971	0.0437192
Syne1	-0.5639734	8.4686891	0.0007131	0.0442289
Abcg1	0.4581139	5.1661467	0.0007139	0.0442289
Cyyr1	-0.7898486	2.3894597	0.0007161	0.0442289
Map3k15	-0.6980504	3.9301442	0.0007166	0.0442289
Acp1	-0.5728974	6.1349258	0.0007287	0.0447981
LOC118567623	-0.9240577	1.7617105	0.000734	0.0449457
Rbfa	-0.4864235	5.7767109	0.0007394	0.045103

Rsph9	0.9431348	1.7460772	0.0007487	0.0453177
Serpinb2	-0.6751486	3.6869581	0.0007488	0.0453177
Pou2f2	0.6280769	4.9629192	0.0007519	0.0453268
Ppp1r3b	-0.6060625	4.7361735	0.0007603	0.0456607
Ptx3	1.19779	2.5065945	0.0007793	0.0464484
Klra2	-1.0002406	1.4350946	0.0007794	0.0464484
Gramd1b	-0.5006572	5.4374078	0.0007848	0.0465926
9330154J02Rik	-1.8082976	-0.1036434	0.0007919	0.0468339
Igf2bp3	1.9170733	-0.16315	0.0008034	0.0472003
Dbp	1.3684142	3.9902551	0.0008042	0.0472003
Tex2	-0.4958265	5.8582084	0.0008083	0.047264
Apcdd1	0.9368987	3.6170989	0.0008186	0.0474938
Ddx60	-0.8806021	3.7176241	0.000825	0.0474938
Fam102b	-0.4817834	5.8190762	0.0008265	0.0474938
Cpne3	-0.6278073	7.4450157	0.0008289	0.0474938
Cavin2	-0.5422401	6.7869488	0.0008323	0.0474938
Syt6	2.0894347	1.167205	0.0008357	0.0474938
6430548M08Rik	-0.6045207	6.3858275	0.0008366	0.0474938
Slpi	-0.580593	6.3016202	0.0008367	0.0474938
Trdn	-3.3807836	-0.365772	0.0008444	0.0477585
Rgs18	-0.5815784	7.0650589	0.00085	0.0478977
Epb41l4b	0.7279799	3.8802234	0.000853	0.0478977
Cd9	-0.4307094	7.7740633	0.00086	0.0479552
Inpp4a	-0.4878387	5.7022217	0.0008602	0.0479552
Sqor	-0.5627127	5.7135924	0.0008692	0.0481141
Nhsl2	-0.7532581	6.5107409	0.0008692	0.0481141
Dhrs7	-0.4289991	6.1925692	0.0008766	0.048352
Igf2	-1.773869	2.1500548	0.0008802	0.0483783
Ccpg1	-0.483732	7.418578	0.000886	0.0485259
Hmgcll1	0.9365433	2.933081	0.000895	0.0488467
Dgkh	-0.6509266	4.7274471	0.0009271	0.0504172
Psmd9	0.5098296	5.5012981	0.0009338	0.0506047
Tagln3	3.1556134	-1.4574694	0.00094	0.0507673
Ly6a2	-0.6302683	7.4540925	0.0009526	0.0512467
Clec1b	-0.4988127	5.8527016	0.0009555	0.0512467
Itgb2l	-0.5521816	5.101819	0.0009676	0.0517164
Grem1	-0.6873421	2.8167017	0.0009728	0.0518203
Plxnc1	-0.5957399	6.2072566	0.0009802	0.0520325
Plbd1	-0.5129854	7.649181	0.0009856	0.0521417
Thrsp	1.3729667	2.9819958	0.0009923	0.0523131
BC021767	1.0193653	1.529727	0.0009955	0.0523131
Aoah	-0.6861768	4.4792289	0.0010066	0.0527145
Gucy1a1	-0.4387626	5.188421	0.0010137	0.0529087

Wfdc21	-0.6557061	8.0671306	0.0010414	0.0541728
Gpr27	-0.649456	3.7319962	0.0010474	0.0543055
I830077J02Rik	-0.4543805	5.8215432	0.0010539	0.0544597
Olfr289-ps1	1.4747971	0.073654	0.0010606	0.0546229
Cep19	-0.5176485	4.8154834	0.0010675	0.0548
Cacna1s	-2.2842791	-0.0754807	0.0010894	0.0557379
Rbm17	0.3381677	6.6189987	0.0011194	0.0568655
Tcap	-2.6464966	1.7097926	0.0011197	0.0568655
Cd300a	-0.5794655	5.137351	0.0011235	0.0568655
Ppp2cb	-0.3683309	6.7141581	0.0011261	0.0568655
Fn3krp	1.0347269	5.7319548	0.0011347	0.0569315
Eif2ak2	-0.3943019	5.6622221	0.0011347	0.0569315
BC016579	-1.1671	-0.0070654	0.00114	0.0570134
Glipr1	-0.450617	6.2040121	0.0011506	0.0573598
Otulinl	0.4164259	5.0395017	0.0011565	0.057397
Tcf3	0.4930287	7.4086572	0.0011592	0.057397
Dnajb14	-0.406376	5.1578339	0.0011624	0.057397
Neb	-2.4392579	2.1148869	0.0011748	0.0577663
Nabp1	-0.4710424	4.6491668	0.0011773	0.0577663
Plppr5	-1.9666612	1.4846034	0.0011855	0.057985
Atp2a1	-2.7694012	3.4576825	0.0011924	0.0581416
Id4	0.9098853	3.7988022	0.0012209	0.0593427
Vpreb3	1.2205857	5.3582306	0.0012298	0.0595877
Dennd2d	-0.5305611	4.9099847	0.001242	0.0599397
Dhrs9	-1.1603636	4.4853815	0.0012447	0.0599397
Ckm	-2.4635413	3.8517234	0.0012509	0.0600491
Nrap	-1.5313137	0.4042757	0.0012583	0.0602188
Pwp2	0.4792134	4.2036514	0.0012863	0.0612162
Slc12a3	1.6913644	1.3128121	0.001287	0.0612162
Dach1	-0.7886527	5.041398	0.001367	0.0648247
Rpp25l	0.5401551	3.6278784	0.0013756	0.0650334
Kcna2	-0.9458614	2.9000687	0.0013878	0.0654127
Erdr1	0.6343104	4.7476453	0.0014195	0.0666582
Spata13	0.4459584	6.3542681	0.0014228	0.0666582
Matn4	1.5657423	-0.3952631	0.0014486	0.067663
Slc7a11	-1.5193007	1.9866029	0.0014728	0.0685851
Braf	-0.4387731	5.9077392	0.0014822	0.0687918
Abca13	-0.8670074	6.1890365	0.0014861	0.0687918
Fpr2	-0.5901964	6.7910378	0.0015079	0.0695969
Xdh	-0.6221093	7.1284446	0.0015143	0.0696473
Lhx2	1.6602137	-0.5694832	0.0015188	0.0696473
Trnp1	2.0181742	-0.5632425	0.0015224	0.0696473
Gpam	0.6451253	5.1979163	0.0015366	0.0700878

Phb2	0.3544433	7.0937448	0.0015654	0.0710541
Zbtb7b	-0.4222425	6.2681359	0.0015669	0.0710541
4933439C10Rik	0.9494892	3.3736432	0.0016184	0.0731741
Mettl9	-0.4406682	6.80452	0.0016469	0.0742473
Clec12a	-0.4912351	6.9899362	0.0016605	0.0746448
Tiparp	-0.4640492	4.7278444	0.0016729	0.0749454
Gm38865	1.5194991	-0.0049929	0.0016806	0.0749454
Dok2	-0.7364747	5.9385534	0.0016816	0.0749454
Gm15411	1.3232736	3.0357712	0.0017323	0.0769821
Arpc5l	0.4512366	5.9837396	0.0017501	0.0772061
Lxn	0.6892094	3.630671	0.0017521	0.0772061
Phlda3	0.6974766	3.3666271	0.0017572	0.0772061
Cbx3-ps6	1.8355298	0.2559503	0.0017686	0.0772061
Tmem254a	0.9129684	3.9111288	0.0017722	0.0772061
Septin5	-0.5251662	5.6032881	0.0017765	0.0772061
Igsf6	-0.5087153	7.61311	0.001786	0.0772061
Mmrn1	-0.6399931	6.4015653	0.0017899	0.0772061
Tmem38a	-0.9731655	2.3009836	0.0017912	0.0772061
Ripply3	0.9931982	0.9880956	0.0017924	0.0772061
Actn2	-3.4801643	0.4372723	0.0017959	0.0772061
Angpt1	-0.456766	5.6512884	0.0017969	0.0772061
Oscp1	-0.6681839	2.9303532	0.0018145	0.077749
Sertad4	1.2446462	1.7966686	0.0018211	0.0778189
Creb3l2	-0.5670006	4.1819283	0.001837	0.0781921
Gm41914	-0.8873975	2.5145565	0.0018399	0.0781921
Cd79a	0.9006009	6.9842523	0.001853	0.0785343
Cd200r1	-0.6245244	3.296773	0.0018765	0.0793118
Gm10175	1.4587109	-0.4810328	0.0018925	0.0797711
Tbc1d8	-0.4335374	5.972076	0.0019319	0.080577
Cds2	-0.429324	7.1961566	0.0019408	0.080577
Tnnt3	-2.6043112	4.3486287	0.0019423	0.080577
Gapt	-0.7316218	5.2889962	0.0019432	0.080577
Sphk1	0.5778375	4.9689775	0.0019472	0.080577
Ebf1	0.8816004	6.281905	0.0019513	0.080577
Ltb4r1	-0.5357271	5.8190468	0.0019534	0.080577
Ptpn3	-0.6145242	3.0373265	0.0019562	0.080577
Ston2	-0.60491	4.9701252	0.0019582	0.080577
Prdx5	-0.4671056	8.549149	0.0019714	0.0809046
Cd300ld	-0.6933141	4.2655425	0.0019861	0.0811422
Ncf2	-0.4254475	7.5291977	0.0019876	0.0811422
Gca	-0.6561472	6.0347108	0.0020105	0.0816829
Fasn	0.4144345	5.5462276	0.0020134	0.0816829
Akap17a	0.4038769	5.906915	0.0020166	0.0816829

Gbp6	-1.2671461	1.6203345	0.0020257	0.081839
Ptafr	-0.4219043	5.2299887	0.0020509	0.0826334
Actn1	-0.4297228	7.9940155	0.002056	0.0826334
Cpd	-0.3573644	7.3617356	0.002066	0.0828211
Pira1	-1.1953414	2.9217975	0.0020788	0.0831191
Adam8	-0.6325711	5.8113698	0.002119	0.0845119
Sntb1	-0.5080769	4.6805834	0.0021358	0.0846312
Gm32900	1.2699104	0.7021394	0.0021367	0.0846312
Bmf	0.8907555	4.4760748	0.0021384	0.0846312
Ly6c2	-0.5744377	8.4628858	0.0021553	0.0850862
Zpbp2	1.151273	-0.0345721	0.0022038	0.0864873
Stat4	-0.605855	4.1225601	0.0022093	0.0864873
Snap23	-0.3427836	6.4872589	0.0022129	0.0864873
Zfp94	0.876732	1.3839297	0.002214	0.0864873
Lcn2	-0.5620131	10.874762	0.0022186	0.0864873
Cx3cr1	0.529522	4.4432256	0.002252	0.0875694
Dpp4	0.6320716	5.6248683	0.0022784	0.0879115
Pstpip2	-0.4473477	5.5007284	0.0022788	0.0879115
Hprt	-1.7961108	4.4686034	0.0022795	0.0879115
Samd9l	-0.3583858	7.4004	0.0022858	0.0879115
Slc16a3	-0.5764545	5.9572557	0.0022891	0.0879115
Rasgrp4	-0.4868581	6.2782953	0.0022974	0.0880153
Epcam	-1.0450366	1.2887175	0.0023085	0.0882222
Myot	-3.1221598	0.6289786	0.0023195	0.0882382
Lef1	0.9665476	4.3332227	0.0023254	0.0882382
LOC115487194	-0.7694966	2.2796017	0.0023288	0.0882382
Svil	-0.434165	6.9481773	0.0023372	0.0882382
Plxdc2	0.5120532	4.8280997	0.0023373	0.0882382
Panct2	-0.7105913	3.2953514	0.0023542	0.0886607
Ccl6	-1.0464815	7.0994439	0.0023618	0.088732
Bend4	-1.0759021	2.3266755	0.0023763	0.0890624
Serpina1d	-3.3261596	1.1185483	0.00239	0.0892121
Coq7	0.4561627	4.2477329	0.0023918	0.0892121
Gm5150	-0.9541734	2.4132752	0.0024279	0.0898148
Il7r	1.1266029	5.4098975	0.0024281	0.0898148
Tmem86b	0.6277069	3.8078408	0.0024305	0.0898148
Myo18b	-1.3648131	0.3579004	0.002431	0.0898148
Pi16	-0.5993941	5.7401742	0.0024546	0.0901549
Ro60	-0.417928	5.287194	0.0024559	0.0901549
Ifitm6	-0.5787354	7.4217971	0.0024576	0.0901549
Fcer1a	-0.9095682	1.9742122	0.0024718	0.0904621
Niban1	-0.611882	6.5719233	0.0024835	0.0906774
Inhba	-0.7056148	4.7090379	0.0025187	0.091748

Tmem170b	-0.4742491	5.6674245	0.0025265	0.0918153
Scnn1a	-0.698053	4.2163031	0.0025704	0.0931932
Ankrd28	-0.4231529	7.5348731	0.0025866	0.0933561
Pygm	-0.9270443	3.2563264	0.0025945	0.0933561
Fam83g	1.340336	0.4779534	0.0026002	0.0933561
Mmp9	-0.5244059	9.8452571	0.0026037	0.0933561
Stx11	-0.402945	6.7104247	0.0026049	0.0933561
Rnf125	-0.5570775	4.7371883	0.0026286	0.0939357
Peli2	-0.5474554	4.864821	0.0026332	0.0939357
Lcp2	-0.3631619	6.8208919	0.0026687	0.0949873
Lyst	-0.4738915	7.5477385	0.0026937	0.0956564
Gm36753	-0.8777797	2.2437866	0.0027013	0.0957095
Itfg2	0.5905463	2.8970786	0.0027105	0.0958166
Lin28a	-0.5245331	5.3127024	0.0027476	0.0969069
Pnpo	0.6564523	6.579384	0.0027924	0.0982641
Ngp	-0.5160477	12.710861	0.0028088	0.0986196
Il1rap	-0.4192159	4.4653773	0.0028153	0.0986254
Scp2	-0.4164752	7.6740592	0.0028265	0.0986305
Dock5	-0.5023715	6.3683093	0.0028282	0.0986305
Chil1	-0.4915105	8.4378471	0.002835	0.0986473
Calcrl	-0.4468099	4.822602	0.0028424	0.0986835
Sbk1	0.8428309	5.2736257	0.002892	0.0999869
Ntng2	-0.5431049	3.4994126	0.0028928	0.0999869
Bcas2	0.3823646	6.0553725	0.0029002	0.100022
Clcf1	1.0414276	2.2347026	0.0029218	0.1005433
Rpgrip1	1.002711	1.5635808	0.0029368	0.1008348
Rab27b	-0.4975735	6.4205328	0.0029856	0.1019522
Slco4c1	-0.8711886	4.8976851	0.0029978	0.1019522
Usp53	-0.5881921	3.4056588	0.0029997	0.1019522
Lgr5	0.9030447	2.3924756	0.0030135	0.1019522
Itgb2	-0.5102577	8.6190834	0.0030144	0.1019522
Blk	0.8430826	4.4112722	0.0030147	0.1019522
Lims1	-0.4575967	8.3769953	0.0030152	0.1019522
Pla2g4a	-0.4129118	5.2711825	0.0030305	0.1022477
Smarcd1	0.3712195	4.6866109	0.0030411	0.1023822
Ddah2	0.6258365	3.3197061	0.0030672	0.1028366
Ppbp	-0.4994442	10.040422	0.0030678	0.1028366
Srm	0.4510647	5.2429689	0.0030946	0.1035122
Hook3	-0.3883267	6.7907596	0.003123	0.1041713
Cplx2	0.9615621	5.0038091	0.0031325	0.1041713
Fnip1	-0.4035577	6.2167541	0.0031344	0.1041713
Ifit1bl2	-0.8828409	3.1344288	0.0031506	0.1044872
Stard10	0.8215321	6.2911666	0.003173	0.1050056

Ism1	0.8132991	3.0564117	0.0032177	0.1061332
Gm4735	1.4280977	2.7608218	0.0032207	0.1061332
Stfa3	-1.2956869	1.9023104	0.0032505	0.1067376
Tef	0.8777102	4.6757367	0.0032528	0.1067376
Itih5	-0.5390727	4.8850693	0.0032657	0.1067476
Cybb	-0.6707102	9.4538289	0.0032713	0.1067476
Endou	0.8152475	3.0797437	0.0032737	0.1067476
Snrpb	0.3054115	6.9825337	0.0033072	0.1076164
Hdgf	0.3855348	8.7839924	0.0033375	0.1083756
P2rx1	-0.520769	5.1178994	0.0033489	0.1085173
Slc25a3	0.3369231	7.9332653	0.0033624	0.1085901
Cd177	-0.5040092	9.3451915	0.0033651	0.1085901
Gm3942	-0.9718198	0.8519803	0.003384	0.1089751
Enpp5	-0.4203183	4.7311371	0.0033913	0.1089852
Nlrp3	-0.6765801	3.867701	0.0034057	0.1092206
Sema4b	0.3427675	5.7608756	0.0034699	0.1110503
Mdp1	0.4520182	5.058927	0.0034844	0.1111321
Tnfaip8l2	-0.4780639	5.0367936	0.0034905	0.1111321
Cnp	0.5943789	6.6088194	0.0034973	0.1111321
Lypd6	-1.2517762	-0.8067941	0.0035148	0.1111321
Rcc1	0.3922964	5.9183877	0.003518	0.1111321
Mybpc1	-3.3324106	0.6895926	0.0035341	0.1111321
Ptprc	-0.4357194	8.6416793	0.0035342	0.1111321
Sorl1	-0.4837533	7.9201732	0.0035378	0.1111321
Fam160b1	-0.4198682	5.9562123	0.0035397	0.1111321
Tmod4	-0.8293309	1.7216464	0.0035569	0.1111321
Cish	-0.8625829	2.3191756	0.003561	0.1111321
Ttn	-2.2182526	2.6781449	0.0035664	0.1111321
Cped1	-0.5030717	3.6708551	0.0035824	0.1111321
Chil3	-0.5430367	10.927525	0.0035874	0.1111321
Ern1	-0.5322571	5.3888651	0.0035957	0.1111321
Ms4a4a	0.7793366	3.2318736	0.0035996	0.1111321
Gm35113	-1.9380452	-0.2702999	0.0036018	0.1111321
Gpatch4	0.4303383	4.2172255	0.0036029	0.1111321
Mcam	-0.5958266	3.5614397	0.0036117	0.1111321
Disp2	1.3661186	-0.0123284	0.0036153	0.1111321
Rab37	-0.550424	4.0095022	0.0036492	0.111659
Ppcs	0.6248792	2.7219709	0.0036502	0.111659
Lsm4	0.3104277	6.3372106	0.003654	0.111659
Alpk3	-1.6011443	-0.6159986	0.0036993	0.1128201
Desi1	0.3703047	5.7816581	0.0037132	0.1129836
Prr5l	-0.5500528	3.5692433	0.0037197	0.1129836
Selenbp2	-1.2991672	0.3654359	0.0037307	0.1129836

LOC118568543	1.3143762	-0.3635526	0.0037337	0.1129836
Pde3a	-0.6002268	5.0781791	0.0037773	0.1140823
Tirap	-0.5941441	5.24418	0.0038031	0.1146371
Plec	-0.4629248	6.8204575	0.0038141	0.1147394
Tcf24	-1.4366889	-0.2256255	0.0038212	0.1147394
H4c8	1.7444987	-0.1484371	0.0038351	0.1149104
Snrpa1	0.4201133	5.4076025	0.0038421	0.1149104
Rpl30	0.5535326	8.8012945	0.0038491	0.1149104
Gm16619	1.6813834	-0.7688432	0.0038575	0.1149422
LOC118568370	-0.9794527	1.1020686	0.003872	0.1151532
Usp13	-1.2685682	0.2476003	0.0039092	0.115779
Gm7887	-0.8704884	2.9793525	0.0039137	0.115779
Cdkl2	-1.4312586	0.8994357	0.0039154	0.115779
Ecrg4	0.9610775	2.2595264	0.0039471	0.1162009
C1qbp	0.3702398	6.3757429	0.0039523	0.1162009
Mxd1	-0.4165391	8.4473514	0.0039553	0.1162009
Adgrg6	-0.8811066	2.8674752	0.0039647	0.1162009
Rcc2	0.3605396	8.1284723	0.003967	0.1162009
Slc30a10	0.6759175	3.9561088	0.0039791	0.116335
Bmyc	0.5692536	2.5885424	0.0040103	0.1169639
H3c8	1.4707385	0.0151488	0.0040156	0.1169639
Gm30963	-1.6732282	-0.8264284	0.0040323	0.1172296
Il7	0.6180067	2.6095462	0.0040416	0.1172812
Gm6713	-1.6493452	-0.0435274	0.0040998	0.1185422
Cd209f	-0.8359065	2.0437683	0.0041003	0.1185422
Pgls	0.385597	6.6034206	0.0041166	0.1187919
LOC115490354	0.9607717	1.4593573	0.0041376	0.1191787
Pygl	-0.3996455	8.5155053	0.0041502	0.1193188
Ampd1	-2.3994561	-0.2782662	0.0041686	0.1194156
Gm33370	1.3353157	0.7888582	0.0041764	0.1194156
Fcrla	0.9145485	5.4473173	0.0041797	0.1194156
Igkv19-93	-0.7293437	4.0492864	0.0041842	0.1194156
Myo9a	-0.3801119	5.7164215	0.0042067	0.1198359
Gm37065	1.3374034	1.2342894	0.004265	0.1212754
P2ry14	-0.8285645	2.231263	0.0042829	0.121563
P2ry6	0.7041408	3.2149304	0.0043398	0.1228778
Mgl2	-0.6197162	3.7614059	0.004345	0.1228778
Mmp25	-0.5898142	5.8966129	0.0043915	0.123966
Mdk	1.0419424	3.3635521	0.0044049	0.1241188
Tbc1d2	-0.4491305	4.2920519	0.0044219	0.1243742
Plaur	-0.5216595	5.945782	0.0044981	0.1262888
Rps7-ps3	0.7136027	6.0963706	0.004524	0.1267859
Tpm2	-0.4660071	5.407331	0.0045403	0.1269297

Blnk	0.8239725	5.4122996	0.0045454	0.1269297
Tshr	-0.6321051	2.4812325	0.0045642	0.127225
Samd4	-0.588976	4.0816818	0.0046016	0.1278805
Card10	-0.4941793	4.4266803	0.0046046	0.1278805
Ptgs1	-0.3953655	5.9183874	0.0046124	0.1278805
Gfra1	0.798766	3.7576705	0.0046604	0.1289809
Pym1	0.391894	4.5845748	0.0047153	0.1299215
Mfsd6	-0.3646465	5.1083079	0.0047174	0.1299215
Glrx	-0.495799	6.3385733	0.0047253	0.1299215
Tnfrsf13c	0.8892192	4.8311173	0.0047335	0.1299215
Utrn	-0.4056243	6.2383683	0.0047361	0.1299215
Mkx	1.1699324	1.0351041	0.0047582	0.1302972
Syt9	1.2501683	1.1343313	0.0047934	0.1310302
LOC105246083	1.3776407	-0.3838588	0.0048155	0.1314042
Ttyh2	-0.7664919	3.2740663	0.004824	0.1314051
Ccno	-0.8077822	2.4651038	0.0048381	0.131447
Spef1	0.7320745	2.6482351	0.0048502	0.131447
Osgep	0.4090196	4.9308196	0.0048509	0.131447
Galnt18	-0.6522303	2.4412976	0.0048611	0.1314954
Nlrp12	-0.5799435	4.0383544	0.0048783	0.1315081
Gm40935	2.0497018	0.6335377	0.0048785	0.1315081
F5	-0.6504184	7.473353	0.0049013	0.1318952
Ifrd2	0.5249864	7.1281254	0.0049277	0.1321976
Igl	0.7041837	6.8589383	0.0049296	0.1321976
Gbp8	-0.8011921	2.5523703	0.0049616	0.1326561
Slc9a9	-0.3966416	4.2702982	0.0049637	0.1326561
Csrp3	-1.2933233	0.5202757	0.0049904	0.1331401
Steap4	-0.4518513	5.8369192	0.0050176	0.133467
Stambpl1	0.5694949	4.8978474	0.0050308	0.133467
Dnajc7	0.3604498	7.3371258	0.0050309	0.133467
Med19	0.3670914	5.0214497	0.005037	0.133467
Gm6916	1.9883808	0.0059324	0.0050758	0.134216
Kcnj5	-0.5715586	4.4523524	0.0050841	0.134216
Zfp991	-0.7481342	2.816333	0.0051007	0.134216
F830208F22Rik	-1.5445815	-0.1668843	0.0051069	0.134216
Pglyrp1	-0.485199	8.5646503	0.0051084	0.134216
Gm15433	-0.9793002	0.7865161	0.0051247	0.1344185
4930438A08Rik	-0.4868928	5.3865406	0.0052165	0.1363825
Lnpep	-0.6382589	6.6472676	0.0052421	0.1363825
Gm4617	0.5777245	4.788996	0.0052488	0.1363825
Tbc1d30	-0.8653133	1.0391082	0.0052528	0.1363825
Zfp518a	-0.355594	5.4243239	0.005255	0.1363825
Zfp654	-0.3810127	5.3621695	0.0052606	0.1363825

Rhou	-0.4019095	5.4174204	0.0052617	0.1363825
Col14a1	-0.7296539	4.4723451	0.0052698	0.1363825
Mrpl43	0.3694106	4.8096605	0.005293	0.1367554
Aatk	-0.4525387	4.1940883	0.0053239	0.1368491
Irak1bp1	0.8647031	0.8050151	0.0053259	0.1368491
Gm8459	-1.0292581	0.8755998	0.0053333	0.1368491
Lysmd1	0.8379382	1.4881646	0.0053408	0.1368491
Dgat1	-0.4693603	6.0469809	0.0053461	0.1368491
Hemk1	0.9106733	2.3950686	0.0053494	0.1368491
Hk3	-0.5385441	7.3143932	0.0054179	0.1380246
Ero1a	-0.4621798	6.3829637	0.0054199	0.1380246
4930426D05Rik	1.1468158	2.2394472	0.005422	0.1380246
Srpk2	-0.3440695	6.1916912	0.0054402	0.1381227
Kdm6b	-0.4223149	6.3050162	0.0054472	0.1381227
Prss50	0.7640324	1.8672713	0.0054525	0.1381227
Gsdme	-0.5252726	4.3992054	0.0054938	0.1387865
Sirpb1c	-0.7529737	3.313476	0.0054965	0.1387865
Nlrp1a	-0.9505426	2.3409264	0.005509	0.1388765
Kcne4	-1.3981189	0.1168959	0.0055567	0.1397948
Xpc	-0.4420929	5.3958482	0.0055646	0.1397948
Cmtm6	-0.370957	6.9791982	0.0055823	0.1397948
Mylk3	0.4698147	5.5748433	0.0055977	0.1397948
Arg2	-0.8662126	3.2650215	0.0055981	0.1397948
Nos1ap	-0.5851402	2.7299838	0.0055993	0.1397948
Acad11	-0.5130126	4.6684105	0.0056152	0.1399652
Zbed6	-0.4659234	5.7401626	0.0056477	0.1405512
Sfrp1	0.6805061	3.6025338	0.0056986	0.1415655
Snx13	-0.3443136	6.0279738	0.0057168	0.1415655
Elovl7	-0.5470116	3.8220133	0.0057208	0.1415655
2010016I18Rik	-1.4028067	1.7418974	0.0057249	0.1415655
Lat2	0.4173526	4.3360088	0.0057712	0.1424855
Igf1r	-0.4596384	6.6152691	0.0057825	0.1425379
F8	-0.5448758	4.1721788	0.0058051	0.142847
Mro	1.5309316	-0.8930848	0.0058134	0.142847
Lmbrd2	-0.38283	5.1066598	0.0058447	0.1433877
4833407H14Rik	-0.9562639	2.4335002	0.0058542	0.1433963
Atp1b1	0.9313633	6.1041657	0.00587	0.1435557
Trem3	-0.4686762	5.9056229	0.0059125	0.1443697
Dolpp1	0.5087672	4.0635037	0.0059233	0.1444047
Atp7a	-0.3567457	5.0442052	0.0059371	0.1445149
Shc3	-1.5234589	-0.4808567	0.0059655	0.1448287
Snn	0.6476364	5.6336587	0.0059832	0.1448287
Ccdc86	0.4479381	4.0053591	0.0060068	0.1448287

Gsn	-0.3929714	8.0124314	0.0060088	0.1448287
Unc119	-0.4299704	5.9642295	0.0060114	0.1448287
Ube3a	-0.3064731	6.9433865	0.0060149	0.1448287
Cd40	0.6980263	2.5636944	0.0060151	0.1448287
Megf9	-0.6779676	5.9121537	0.0060795	0.1461516
Trip11	-0.334012	5.9822791	0.0061376	0.1470653
Pim2	0.5647049	3.9345409	0.0061448	0.1470653
Nlgn3	1.2783637	-0.4910278	0.0061458	0.1470653
Gm16252	1.5457348	-0.6780948	0.0061601	0.1471793
Otud1	0.4052168	4.2337431	0.0061706	0.1472047
Gm36393	-1.2748011	-0.7944845	0.0061862	0.1473506
Prpf38b	0.342829	7.4876509	0.0061962	0.1473632
Ifitm1	-0.6871156	5.2093454	0.0062275	0.1478826
Arl5c	0.8494258	4.8405211	0.0062621	0.1479884
Tg	-0.7230905	2.542946	0.0062686	0.1479884
Adgre4	-1.0021378	3.7684658	0.0062715	0.1479884
Tomm40	0.3414829	5.3684033	0.0062835	0.1479884
Fmnl2	-0.5207763	4.8636781	0.0062849	0.1479884
Eif4ebp1	0.3281522	5.4691929	0.0062933	0.1479884
Pfdn6	0.3473595	5.7129741	0.0062986	0.1479884
Scube3	-1.1251607	0.6498042	0.0063216	0.1483047
Pou2af1	0.9171984	6.6218848	0.0063387	0.148482
Trmt61a	0.5482549	3.118991	0.0063853	0.1493492
Cd79b	0.7813567	6.7629814	0.0063952	0.1493562
Gm10693	-2.2444653	-0.6352773	0.0064207	0.1497266
Mov10	-0.3838853	4.6379906	0.0064552	0.150306
Pik3c2a	-0.4709765	5.3089809	0.0065126	0.1511511
Ndn	1.318844	0.0233599	0.0065165	0.1511511
Gm30211	0.9394404	0.2891381	0.0065207	0.1511511
AI467606	-0.3518793	5.8837137	0.0065337	0.151228
Gnaq	-0.3199132	6.5372386	0.0065436	0.1512312
Gm33583	-0.9123014	1.9579274	0.0065655	0.1515126
Syvn1	0.4435231	6.1958817	0.0065867	0.1516057
Pck2	0.362235	6.0953966	0.006589	0.1516057
Rinl	-0.344732	5.7878522	0.0067203	0.1543979
Nadk	-0.3633109	7.5506334	0.0067652	0.1550427
Mfhas1	-0.4121045	5.337479	0.0067683	0.1550427
Gm51657	-1.1121434	1.3966389	0.0067985	0.1555048
Acp6	0.4324486	3.6471404	0.0068333	0.1559546
Phf5a	0.382444	6.2041119	0.0068382	0.1559546
Slc2a6	-0.5470444	3.3392873	0.0068692	0.1564319
Atp6v0e2	1.2575747	0.6732398	0.0068986	0.1568713
Prr3	0.604371	3.0973494	0.0069255	0.1572545

Prkg1	-0.5520048	3.2524058	0.0069438	0.1574392
Igkv5-48	-0.8849938	3.2301014	0.0069579	0.1575307
Lpcat2	-0.3757318	5.5069634	0.0069765	0.1577218
Havcr1	-0.9518802	0.4055174	0.0069897	0.1577909
Psmb10	0.3903088	4.9395211	0.00703	0.1584698
Fcho1	0.3377627	6.2313916	0.0070545	0.1585271
Clec9a	-0.9271298	1.706297	0.0070572	0.1585271
Atp10d	-0.7292761	4.7522202	0.0070631	0.1585271
Rapgef6	-0.3680057	6.9818982	0.0071032	0.1591975
Tcn2	-0.3893812	6.7594632	0.0071458	0.1599229
Gm8995	-0.4601708	7.5143439	0.0071576	0.1599562
Nefh	0.7348594	2.0385519	0.0071697	0.1599966
Gm5637	-1.159178	0.081215	0.0071814	0.1600281
Cxcr2	-0.421355	7.7906241	0.0071941	0.1600813
Tnnc1	-1.7303486	0.3802983	0.0072238	0.1604021
Pram1	-0.4565139	6.5846815	0.0072387	0.1604021
5830416I19Rik	-1.2309951	-0.6492203	0.0072394	0.1604021
Fmo2	0.9524721	3.6023375	0.0072839	0.1611174
Zfand4	-0.4839107	4.0894332	0.0072924	0.1611174
Cnn2	-0.3609243	8.2326113	0.007335	0.1618275
Ccn5	-0.7212897	3.8289882	0.0073471	0.1618657
Gm32917	-1.0464054	1.2901434	0.0073792	0.1623424
Amigo1	0.5974002	2.6064864	0.0074239	0.1630956
Ugdh	-0.3299545	6.0801609	0.0074651	0.1637682
Ripor2	-0.4209411	6.5372156	0.0074792	0.1638479
Fam126b	-0.4244979	4.9527061	0.0075247	0.1646119
Ifnlr1	-0.5335355	4.2273216	0.0075414	0.1647461
Nfam1	-0.4050891	7.2154359	0.0076022	0.1658401
H4c14	2.4329893	-0.5252895	0.0076236	0.166075
Dgki	-0.9407191	3.4081143	0.0076999	0.1673256
Cbx6	0.3230808	5.4026111	0.0077025	0.1673256
Gm19343	-1.3484816	-0.1960083	0.0077168	0.1674012
Igkv12-44	-1.2484032	4.3802878	0.007736	0.1674182
C5ar1	-0.438595	6.3747796	0.0077391	0.1674182
Dap3	0.3243967	6.0012878	0.0077927	0.1682295
Agap1	-0.3394125	6.0212691	0.0077982	0.1682295
Cysltr2	-0.6275306	3.3375178	0.0078176	0.1684134
Gm19240	1.2066094	-0.6201612	0.0078813	0.169551
Ppp1r14b	0.3531418	5.9037336	0.0079068	0.1698656
Fstl1	0.567295	6.994268	0.0079258	0.170039
Gpat3	0.6492269	3.8256505	0.0079455	0.1702254
Igkv4-69	-2.6049828	0.1558362	0.0079931	0.1710115
Itga2	-0.5137555	5.5353373	0.0080106	0.1711499

Fgr	-0.4020075	6.6211063	0.0080601	0.1719708
Dstn	-0.3623356	8.3772262	0.0080759	0.1719725
Pgam5	0.3235614	4.8374026	0.0080935	0.1719725
Dlg2	-0.69534	3.6883526	0.0080979	0.1719725
Zfand2a	-0.3824935	4.0998459	0.0081044	0.1719725
Gm13092	1.0082328	0.5985237	0.008118	0.172026
Nfat5	-0.3277655	6.4557981	0.0081614	0.1727111
Zfyve16	-0.3827474	4.3285834	0.0081759	0.1727822
Pak6	-0.7922917	1.0150559	0.0082092	0.1732499
AU041133	0.7451459	1.4839009	0.0082344	0.1734845
Dusp3	-0.357259	5.1837856	0.0082426	0.1734845
Fem1c	-0.3662164	5.1825922	0.008266	0.1737432
Perp	-1.0684623	1.3285541	0.008291	0.1740328
Gm10819	1.2554491	-0.4078483	0.0083027	0.174044
Niban3	1.1667279	5.9293498	0.0083209	0.1741896
Ccr1	-0.5197798	6.1346337	0.0083619	0.1747556
Gm39746	1.2050322	0.0534905	0.0083704	0.1747556
Gm32374	-1.3261287	0.1337793	0.0083829	0.1747812
Rab3d	-0.4243738	6.3558626	0.0084715	0.1763934
Raver1	0.309792	5.6228495	0.0085094	0.1769449
Gp6	-0.4787914	5.3689056	0.0085291	0.1771182
Fam78b	-0.884714	3.9702421	0.0085504	0.1773247
Cep135	-0.4018042	5.4952505	0.0086238	0.1786079
Cacnb1	0.8011593	1.6358143	0.0086479	0.1788687
Tmem140	-0.4594064	3.5362809	0.0087682	0.1811165
Wif1	0.6857472	5.6109565	0.0087875	0.1812731
Arhgap15	-0.3854895	5.8918675	0.0087998	0.1812865
Vegfa	-0.3735078	5.4343019	0.0088313	0.1814714
H2-Q5	0.7951736	2.4030534	0.0088321	0.1814714
Clns1a	0.3225862	5.6400601	0.0088546	0.1816938
Prss16	0.782168	2.1435842	0.0088757	0.1818866
Vps26b	-0.3394411	6.3844633	0.0089765	0.183711
Gnai3	-0.3157647	7.6551471	0.0090022	0.1837741
Aff4	-0.3555795	6.6581744	0.0090032	0.1837741
Dock11	-0.4153125	6.6624779	0.0090405	0.1842941
Gpr155	0.4378261	4.2287684	0.0090689	0.1844594
Slc1a5	0.4684319	7.193138	0.0090785	0.1844594
Msto1	0.5154156	2.8880709	0.0090842	0.1844594
Ica1	-0.4232203	4.5040538	0.0090993	0.1845243
Sp140	-0.3653632	5.8776152	0.0091976	0.1862743
Igkv6-20	-1.4494473	1.8944536	0.0092672	0.1874406
Ceacam1	-0.8346643	6.2923145	0.0093362	0.1883839
Nampt	-0.3070039	6.0887106	0.0093381	0.1883839

Cd226	-0.4657066	5.7961816	0.009391	0.1892067
Tnks	-0.4215205	5.6322677	0.0094063	0.1892703
Tnrc6b	-0.370548	7.5918025	0.009432	0.1895412
Mboat7	-0.3415166	5.9367444	0.0094477	0.1896128
Ostf1	-0.3265289	7.555985	0.0094601	0.1896172
Cln8	0.6503862	6.1076038	0.0096009	0.1921918
Snrpa	0.2891089	5.9295693	0.0096259	0.1924443
Ms4a6d	0.7258372	3.6328197	0.0097472	0.1944133
Rnf26	0.3654565	5.5509326	0.0097494	0.1944133
Cd163	-1.0760552	4.9194328	0.0097632	0.1944391
Atp8b1	-0.5680637	2.5472996	0.0098064	0.1950502
Hnmt	-0.5160308	4.234232	0.0098229	0.1951275
Cd19	0.9426812	6.346384	0.0098538	0.1952911
G6pdx	-0.4558911	8.0030775	0.0098628	0.1952911
Lcp1	-0.3467172	9.9057897	0.0098688	0.1952911
Camk1d	-0.442446	4.4520822	0.0098991	0.1956368
Gzma	-1.1086485	1.9382347	0.0099114	0.1956368
Prune2	-0.7827499	1.7684373	0.0099275	0.1957068
Nipsnap1	0.6251878	2.4432035	0.0099499	0.1958298
Kcne3	-0.9380494	1.8967114	0.0099628	0.1958298
Slmap	-0.3402576	6.793836	0.0099715	0.1958298
Rara	-0.3598013	4.967136	0.0100261	0.1964581
Wdfy3	-0.403285	6.2659639	0.0100288	0.1964581
Vcl	-0.464769	8.4131581	0.0100907	0.1972113
Cd53	-0.3700158	7.7050715	0.0100926	0.1972113
Stub1	0.2988953	5.9061104	0.0101056	0.1972167
Gm36161	-0.5418057	4.4134678	0.0101444	0.1976278
Ffar2	-0.4984533	4.518651	0.0101606	0.1976278
Tarbp1	0.52358	4.4856996	0.0101647	0.1976278
Exosc3	0.3214819	5.1373407	0.0102392	0.1980799
Tmem108	1.2392763	0.9637346	0.0102486	0.1980799
Rps6ka2	-0.5528007	4.4569215	0.0102625	0.1980799
Syne2	-0.6143699	5.2699594	0.0102683	0.1980799
Ccdc88b	-0.3664172	6.4570273	0.0102721	0.1980799
Rftn1	0.430828	4.6762367	0.0103025	0.1980799
Igkv8-21	-1.0453546	3.108451	0.0103032	0.1980799
Gm4258	0.9201754	2.380539	0.010316	0.1980799
Tmx4	-0.3926781	7.2384568	0.01032	0.1980799
Rap2c	-0.3205707	6.424339	0.0103333	0.1980799
Ccdc3	1.2594006	2.0010765	0.010373	0.1980799
Tgm3	-1.2037852	-0.7071961	0.0103814	0.1980799
Apobec2	-2.7154731	0.1510436	0.0103864	0.1980799
Arfgef1	-0.3747491	7.5450141	0.010391	0.1980799

Scrg1	-1.0473836	1.995936	0.0103912	0.1980799
Ankrd23	-1.0037767	2.5767962	0.0104013	0.1980799
Bora	0.4430557	4.9353556	0.0104045	0.1980799
Gm39673	-1.3147674	-0.5066164	0.010523	0.200092
Stxbp6	-0.8621033	2.250269	0.010561	0.200568
Gm15931	-1.7120497	0.3333488	0.0106038	0.2009249
Lgi2	-1.5968821	1.5814461	0.0106183	0.2009249
Cdkl4	-1.0318985	-0.1467998	0.0106185	0.2009249
Nkd2	0.8491034	2.8681457	0.0106451	0.2009967
Chp2	-0.8237293	3.3106816	0.0106568	0.2009967
Ltk	1.4107687	0.7467454	0.0106611	0.2009967
Slc35e1	-0.3663014	6.5482443	0.0107224	0.2019087
Mlf1	-1.5126114	0.4113053	0.0108265	0.2034826
Stap1	-0.3999886	4.6682604	0.0108322	0.2034826
Pank3	-0.3833595	6.0166031	0.0108619	0.203693
Sncg	-1.2488353	-0.8549713	0.0108696	0.203693
Igkv6-23	-0.4439885	4.9730847	0.0109255	0.2044954
Gm35813	1.4398722	1.1341066	0.0110261	0.2060722
Medag	-1.4227872	-0.8203581	0.0110584	0.2060722
Rbmxl1	0.2991379	6.1500181	0.0110679	0.2060722
Gm35891	-1.1609169	-0.4472725	0.0110727	0.2060722
Stxbp5	-0.3552292	6.5258986	0.011076	0.2060722
Gpd2	-0.3878779	5.7512317	0.0111074	0.2064099
Gm10687	-1.661872	-1.1882073	0.0111321	0.2066206
Gimap9	0.3812647	4.4536738	0.0111469	0.2066497
Stk26	-0.4223064	6.5754132	0.0111708	0.2068461
Arl16	0.4494913	3.0934646	0.0112176	0.2071984
Gm4786	1.331245	-0.804523	0.0112253	0.2071984
Fam161a	0.7155601	2.3040894	0.0112298	0.2071984
Sost	-1.8203191	5.6919114	0.0112985	0.207748
Mrps26	0.3236862	4.6593911	0.0112991	0.207748
H2ac20	2.0400158	-0.538694	0.0113015	0.207748
Uchl1	0.9830396	2.7901921	0.011313	0.207748
Pvrig-ps	-0.878313	0.0423139	0.0113405	0.2080078
Ifit1	-1.067601	2.6612078	0.0113966	0.2086153
Ppp1r12a	-0.346933	7.8782542	0.0114005	0.2086153
Pacc1	0.4504184	3.9031929	0.011429	0.2088916
6030468B19Rik	0.7579204	3.0270026	0.0114914	0.2094471
Adgrg3	-0.3528022	6.2866334	0.0114999	0.2094471
Emc10	0.3156201	5.7451249	0.0115256	0.2094471
Ptp4a3	0.3803753	7.6581623	0.0115332	0.2094471
Rflnb	-0.3950472	7.1204249	0.0115455	0.2094471
Dram2	-0.328055	5.2815819	0.0115657	0.2094471

Phyhip	0.509066	4.5856391	0.0115728	0.2094471
Cgn	-1.2033915	-0.2204712	0.0115781	0.2094471
Magef1	0.6754763	2.5099861	0.0115806	0.2094471
Ints12	-0.31094	5.4366603	0.0116179	0.2098769
Rp9	0.4447882	5.8239527	0.0116432	0.2100405
Rtn4ip1	0.3855982	3.6628794	0.0116539	0.2100405
B230208H11Rik	-0.454332	3.7883845	0.0117096	0.2108
Mturn	0.364017	4.590394	0.0117369	0.2110464
Upp1	-0.766855	2.3895474	0.0117553	0.2111341
Gm40632	1.6530761	-1.1855995	0.0117993	0.2116792
Serpina3c	1.1073137	0.2186124	0.0118278	0.2119469
Ppp1r3c	-1.0094368	0.974942	0.0118783	0.212606
Ctu1	0.5628571	2.749506	0.0118997	0.2127451
Arf5	0.5188719	7.7379367	0.0119735	0.2138174
Mypn	-1.295033	-0.8352365	0.011992	0.2139027
Slc28a2	-0.5441247	4.6546395	0.0120386	0.2144887
Fndc3a	-0.352274	6.278506	0.0120733	0.2148605
Mgst1	-0.4026685	6.7708119	0.0121622	0.2161944
Lamc3	-1.2141257	1.3747467	0.0122025	0.2166642
Ubc	0.8167926	6.114359	0.0122429	0.2171341
Lima1	-0.4272705	5.2250783	0.0123066	0.218014
Mtarc2	0.3472939	5.6901836	0.01236	0.2185901
Sort1	-0.4366348	7.678123	0.012368	0.2185901
Gad1-ps	-0.6611949	3.4940539	0.0123812	0.2185901
Txnrd3	0.506103	2.9045952	0.0124241	0.2188873
B3gnt5	0.5273909	4.7094912	0.0124262	0.2188873
Pvalb	-2.4534414	2.9322776	0.0124497	0.2190534
Aldh1a7	-1.8104395	0.8490413	0.0124748	0.2192461
Pklr	0.5432823	5.6538439	0.0124894	0.219256
Perm1	-1.4513189	-0.0857851	0.0125128	0.219419
Carmil2	0.5845694	4.3849807	0.0125636	0.2200498
Cenps	0.4427543	4.0497116	0.0125771	0.2200498
Gm10557	0.7671415	1.7352039	0.0125981	0.2201703
Omd	1.0793952	4.3412668	0.0127158	0.2216128
Ppp1r3e	1.559933	0.0197215	0.0127184	0.2216128
Fmo1	0.789711	4.2541133	0.0127277	0.2216128
Glrx5	0.489538	7.8263991	0.0127377	0.2216128
Gm19639	2.0457802	-1.0206662	0.0127809	0.2221154
Ptma	0.304594	10.784616	0.0128063	0.2223088
Gm5879	0.8106061	0.8133468	0.0128247	0.2223812
Phkb	-0.3494292	5.7251634	0.0128483	0.2225419
Rbp7	1.8172893	-0.1140519	0.0129246	0.2233309
Bnip5	-1.3188292	-0.50299	0.0129368	0.2233309

Prkcq	-0.4413964	4.7400939	0.0129369	0.2233309
6530402F18Rik	-0.4621324	4.5370171	0.0129679	0.2236177
Csf3r	-0.3927752	8.1529305	0.0130145	0.2239237
Gps2	0.2623557	6.1625758	0.0130184	0.2239237
P3h2	-0.4482339	3.6147727	0.0130537	0.2239237
Fgfrl1	0.5223018	3.9413721	0.0130646	0.2239237
Ank3	-0.5492748	3.8753113	0.0130647	0.2239237
Tiam2	-0.5101767	4.007849	0.0130721	0.2239237
Golim4	-0.3087427	7.3580043	0.0131059	0.2242556
Hmgn3	0.4782896	4.1431475	0.0132087	0.2257679
Cecr2	0.7857703	6.8208432	0.0132751	0.2266529
Rnf213	-0.3528035	6.9624771	0.013314	0.2270673
Jund	0.3063525	6.5540116	0.0133346	0.2271698
Vnn3	-1.1130976	1.8339657	0.0133898	0.2278609
Gpr50	-1.4072514	0.3996119	0.013416	0.2280572
Tnik	-0.4591288	5.1822877	0.0134418	0.2280595
D130017N08Rik	-0.9619255	0.1336072	0.0134542	0.2280595
Vsir	-0.3550822	8.18397	0.0134601	0.2280595
LOC102643083	1.5834078	0.6139577	0.0134843	0.2282208
Mgp	-0.8509413	4.9336438	0.0135437	0.2289766
Gm11714	-0.8325943	1.611878	0.0135847	0.2294202
C3	-0.4501444	9.0712528	0.0136513	0.2299695
Myh9	-0.4205947	10.088771	0.0136572	0.2299695
9130227L01Rik	1.8028359	-1.1103474	0.0136616	0.2299695
Mettl23	0.4975667	3.6075	0.0136893	0.230188
Tnfrsf26	-0.4564873	3.338216	0.0137339	0.2303193
Sema3a	0.4930395	3.6312113	0.0137465	0.2303193
Sypl	-0.3148136	6.6575612	0.0137758	0.2303193
Ogfrl1	-0.364965	7.6326208	0.013781	0.2303193
Fndc3b	-0.4009266	5.9163923	0.0138001	0.2303193
Cxcl14	0.5005324	5.9877518	0.0138183	0.2303193
LOC115488029	-3.0290807	3.6617102	0.0138259	0.2303193
Asah2	-0.464048	3.701087	0.0138279	0.2303193
Agl	-0.3339494	5.5893964	0.0138373	0.2303193
Zfyve27	-0.3094677	5.4442116	0.0138452	0.2303193
Il1r2	-0.9358072	3.7564744	0.0139002	0.2309872
Ada	-0.4169968	4.269315	0.0139231	0.2311206
Ceacam10	-0.6315132	4.4620836	0.0139912	0.2319368
Hmgn2	-0.4054335	9.7997945	0.0140021	0.2319368
Emp1	0.4732336	5.9331615	0.0140279	0.2321175
Phactr2	-0.4655424	5.791099	0.0140725	0.2326068
Anxa11	-0.4071486	6.9954966	0.0141143	0.2330504
Cklf	-0.3855701	4.6870223	0.0141686	0.2335355

Myo1f	-0.444939	7.6481561	0.0141737	0.2335355
Tek	-0.4150705	4.358292	0.0142209	0.2338819
Hectd2	-0.7622145	1.3211947	0.0142386	0.2338819
Adipoq	0.5939833	6.7213269	0.0142398	0.2338819
Vav3	-0.5099473	5.3607235	0.0142583	0.233939
Tmem50b	-0.2951031	6.0248598	0.0142961	0.2343116
Plekha2	0.484965	7.2478418	0.0143311	0.23437
Ppan	0.4145942	4.8608568	0.0143358	0.23437
Rplp2	0.4389177	8.9627224	0.014379	0.23437
9030619P08Rik	-0.9575753	0.6909954	0.0143794	0.23437
Carm1	0.3546266	6.1553402	0.0143816	0.23437
LOC118568531	-0.8352796	0.9383485	0.0143901	0.23437
Ly6g6f	-0.6281419	3.1211261	0.0144344	0.2346056
Srsf4	0.2640873	7.1258107	0.0144456	0.2346056
Mpzl3	-0.8063902	3.1186117	0.0144498	0.2346056
Dtx3	0.4172467	4.881678	0.0144651	0.2346083
Smarca4	0.4513514	7.8002304	0.0145291	0.2348445
Agpat1	0.2868317	5.8748833	0.0145344	0.2348445
Ndst1	-0.3233446	6.2580944	0.0145389	0.2348445
Gadd45gip1	0.4129814	5.1930146	0.01454	0.2348445
Arid3a	-0.3841645	6.6178444	0.0145953	0.2354928
Ebi3	-0.5415539	4.2706609	0.0146223	0.2355906
Sv2a	1.1245024	-0.5541972	0.0146316	0.2355906
Fmo5	-0.6366109	4.8085801	0.0146506	0.2356524
Clic5	-0.6175341	2.0909745	0.0147543	0.2370748
Nin	-0.4925151	8.0219128	0.0148136	0.2377821
Igkv6-32	-0.9270188	2.7284872	0.0148392	0.2379481
Igkv4-57-1	-1.0969862	2.1428967	0.0149645	0.2397093
Pls1	-0.5485903	4.7240607	0.0149962	0.2397152
Itgal	-0.3712152	7.0103561	0.0149994	0.2397152
Ppp1r16a	0.4147476	3.4035117	0.0150184	0.2397152
Aldh2	-0.4324935	9.0838916	0.0150265	0.2397152
Ddit4l	-0.7080036	2.280015	0.015096	0.2404827
Tmem151b	-0.9836578	1.0306847	0.01513	0.2404827
Kalrn	-0.4066071	5.8251189	0.0151368	0.2404827
Fstl4	1.3817816	-0.8073381	0.0151658	0.2404827
Rad23a	0.4605581	7.8189594	0.015171	0.2404827
Ckmt2	-2.3280849	-0.0415399	0.0151976	0.2404827
Bcl11a	0.3447019	6.1159215	0.015205	0.2404827
Srrd	0.5340155	3.1365381	0.0152097	0.2404827
Polm	0.6239636	4.2631185	0.0152138	0.2404827
Klrd1	-0.7559806	1.4446667	0.0152506	0.2405913
S100a1	-0.3422858	5.4743055	0.0152548	0.2405913

Erp29	0.2966789	7.2930749	0.015267	0.2405913
Pmf1	0.2875482	5.9218577	0.0152913	0.240729
Nek3	0.7971335	0.9918579	0.0153141	0.2408447
LOC118568732	-1.8377297	0.1761154	0.0153439	0.2410195
Cela1	0.4185289	4.5851065	0.0153702	0.2410195
Arhgef6	-0.3416499	6.490444	0.0153717	0.2410195
Ints10	0.3870157	5.3807352	0.0154141	0.2414411
Ifitm3	-0.3513504	7.1344538	0.0154346	0.2415183
N4bp1	-0.3463279	6.0700712	0.0155232	0.2425674
Msra	-0.3749503	5.4605932	0.0155328	0.2425674
Jakmip1	0.7229273	3.704816	0.0155815	0.2430835
Sntb2	-0.4625665	3.8720559	0.0156836	0.2444096
Cdk9	0.2900038	6.3831823	0.0156979	0.2444096
Rnf130	-0.352806	7.0546218	0.0157901	0.2453776
Slc25a38	0.417489	6.6371549	0.0157916	0.2453776
Ecsit	0.4289511	3.4736299	0.0159328	0.2469765
Plcb2	-0.3984505	6.7863237	0.0159385	0.2469765
Cd52	-0.3573795	8.3838689	0.0159471	0.2469765
Gm30294	-1.2255064	0.757535	0.015958	0.2469765
Mfap2	0.8890039	2.4844297	0.0159943	0.247291
Nr4a1	-0.6025446	2.5978343	0.0160642	0.248066
Caskin2	0.391693	4.2562188	0.0160763	0.248066
Cfb	0.5323948	3.2880128	0.0161274	0.2486081
Lrg1	-0.3978334	7.1634636	0.0161709	0.2490318
Gng11	-0.4096076	4.7259569	0.0162234	0.2495937
Map3k9	-0.544219	4.8759573	0.0162443	0.2496673
LOC118568505	1.0401077	0.6107937	0.0163106	0.2504398
Emc8	0.3112282	4.9034451	0.0163501	0.2504638
Lrrc25	-0.3867093	3.8772629	0.0163599	0.2504638
Lrrfip1	-0.3209009	7.1865446	0.0163728	0.2504638
Mcf2l	-0.4257159	4.1259916	0.0164166	0.2504638
Tbc1d8b	-0.3770153	4.872951	0.0164329	0.2504638
Zfp428	0.8070206	1.2205142	0.0164589	0.2504638
Rtp4	-0.6359002	3.6379868	0.0164598	0.2504638
Rc3h1	-0.2634396	6.6198725	0.0164687	0.2504638
Zmpste24	-0.3320837	7.1748177	0.0164707	0.2504638
Tspo2	0.6111764	6.3400672	0.0164783	0.2504638
Ndel1	-0.2780791	5.836067	0.0164893	0.2504638
Ptgir	-0.4691784	4.8056298	0.0165242	0.2505142
Myl3	-2.3188677	-0.5728917	0.0165249	0.2505142
Kcnq3	1.1112876	1.0193722	0.0165781	0.2510771
Snrk	-0.3804854	5.631573	0.0166332	0.2516663
Mettl16	0.3028329	4.9085341	0.0166936	0.2522436

Adgrl4	-0.5573423	4.4550944	0.0167038	0.2522436
Klri2	-1.0725116	0.7197159	0.0167434	0.2525963
Ralgapa1	-0.3163501	6.3934641	0.0167936	0.2530524
B9d2	0.4028473	4.1095228	0.0168073	0.2530524
Gm31639	1.155048	0.6498035	0.0168224	0.2530524
Uhrf1	0.2966251	8.2311793	0.016842	0.2531014
Dennd5a	-0.3256412	7.1156927	0.0168782	0.2534017
1700020L24Rik	-0.4437218	4.7135225	0.0169254	0.2536587
Cd209d	-1.5254912	-0.7597402	0.016928	0.2536587
Prkcg	0.7382159	1.9338441	0.0169524	0.2537809
Hrob	0.6197505	3.5727196	0.0170171	0.2540093
Trp53i11	0.6013166	4.9431499	0.0170426	0.2540093
Trmt1	0.3095699	4.8274125	0.0170643	0.2540093
H4c12	1.2338	-0.0356524	0.0170691	0.2540093
Usp2	0.6537492	2.8671221	0.0170694	0.2540093
Stk25	0.3428394	5.695616	0.0170728	0.2540093
Rpusd4	0.3769338	3.6840495	0.017082	0.2540093
Gtf2h2	0.3371889	4.0593634	0.0171608	0.2549374
Il1a	-0.603108	3.2494825	0.0171885	0.2551054
Olr1	-0.4500828	4.8397385	0.0172109	0.2551938
Snx27	-0.3363386	6.2582546	0.0172391	0.2553688
Plcb4	-0.4911957	3.9205119	0.0172732	0.2554908
Pnpla8	-0.2645235	6.4077328	0.0172802	0.2554908
Rap1gap	0.9999848	2.4854519	0.0172976	0.2555055
Aldh3b3	-0.7021252	1.4525902	0.0173554	0.2560716
4930597A21Rik	1.235022	0.7232354	0.0173689	0.2560716
Dut	0.3208599	7.2908091	0.0174205	0.2563825
Ncr1	-1.0191505	0.9279966	0.0174332	0.2563825
Tm6sf1	-0.3869694	6.154886	0.0174399	0.2563825
Rap1b	-0.2972697	9.0350276	0.0174559	0.2563825
Gar1	0.28094	5.3467265	0.0174984	0.2565349
Sun2	-0.3665605	8.5462031	0.0174993	0.2565349
Tmem40	-0.3172211	5.1383994	0.0175205	0.2565418
Bicd1	-0.598051	3.0387701	0.0175887	0.2565418
Hook2	0.6094288	2.101368	0.0175955	0.2565418
Tars2	0.3203997	4.7402934	0.0175996	0.2565418
Olfm1	0.401866	4.4648929	0.0176012	0.2565418
Ptprb	-0.423225	4.9981449	0.0176037	0.2565418
Scnm1	0.3565693	4.0030671	0.0176152	0.2565418
Clip1	-0.2975597	6.1326307	0.017642	0.2566918
Nod1	-0.3583447	4.5910296	0.0176644	0.2566953
Chst3	0.8784685	6.189026	0.0176752	0.2566953
Igkv13-85	-1.105078	1.8283326	0.0176948	0.2567142

Slc39a11	-0.4120495	5.1010733	0.0177096	0.2567142
Agpat2	-0.3751825	5.3745907	0.0177386	0.2567488
Cnn3	0.3786743	5.880552	0.017745	0.2567488
Lamp2	-0.3175566	8.1510613	0.0177957	0.2570649
H3c3	1.5715993	0.0593817	0.0177999	0.2570649
Setd7	-0.3295299	6.4095686	0.0179217	0.2585844
Pign	-0.3632538	5.6621539	0.0179639	0.2586501
Map3k1	0.2889824	6.5542244	0.0179685	0.2586501
Igkv10-96	-0.6924926	5.5877157	0.0179876	0.2586501
Reln	0.7372965	4.4382427	0.0179928	0.2586501
Stk17b	-0.3641514	7.7892539	0.0180218	0.258761
Cln5	-0.289414	5.0450194	0.018037	0.258761
Pkd2l2	0.4172185	3.9517018	0.0180504	0.258761
D830031N03Rik	-0.753989	3.0962112	0.0180734	0.2588517
Chst11	-0.3289409	6.2808567	0.0181845	0.260204
Plekho1	0.3791249	5.489632	0.0182083	0.2603049
Gm49668	-0.6366139	1.9033006	0.0182322	0.2604068
Foxo1	0.5335371	5.2191159	0.0183173	0.2613825
Cdt1	0.2962967	6.3509202	0.01834	0.2614661
Stmn1	0.3007905	8.1672786	0.0183771	0.2617565
Apoe	0.4169135	9.7814377	0.0184067	0.2619373
Atf5	0.5182383	4.1321779	0.0184593	0.2623201
Scara3	0.7122358	5.2074108	0.0184673	0.2623201
Slc11a1	-0.470902	4.4552119	0.0185095	0.2626791
Chst12	-0.3583804	6.0983991	0.018566	0.2632406
Slc25a24	-0.282749	6.5236216	0.0186642	0.2643922
Pgbd1	1.082108	-0.0276168	0.0186989	0.2646432
Slc22a15	-0.4071755	4.3875457	0.0187413	0.2650019
Trem1	-0.488699	5.0962759	0.0187627	0.2650634
Tmem178	0.8178395	1.822762	0.0187881	0.2651818
Tlr1	0.5273546	3.7430966	0.0188502	0.2654247
Ap4b1	0.3647486	5.028461	0.0188511	0.2654247
Mefv	-0.4318525	4.6134714	0.0188565	0.2654247
Aldh6a1	-0.430565	3.8613631	0.0188768	0.2654699
Akap5	-0.659018	2.7709509	0.0189445	0.2661814
Gm32652	1.2712457	1.907858	0.0189732	0.2663434
Shmt2	0.3293881	6.1462775	0.0190795	0.2675631
Ap5s1	0.4563317	3.3002417	0.0191187	0.2675631
Gk	-0.3402232	4.5749147	0.0191245	0.2675631
Copg2	-0.3607588	5.7432762	0.0191477	0.2675631
Tmprss3	0.9361171	2.4604798	0.0191494	0.2675631
Spib	0.9117864	6.8449741	0.0191633	0.2675631
Rplp0	0.301372	10.300129	0.019181	0.2675707

Mpeg1	-0.3631266	8.6678419	0.0192009	0.2676085
Stx3	-0.6933052	2.9533656	0.0192281	0.267625
Nacc2	-0.3463919	4.8782127	0.0192365	0.267625
Btla	0.5530519	4.936083	0.0192599	0.2677105
Lta4h	-0.3695193	8.1434759	0.0192812	0.2677674
Acadsb	-0.3451814	5.1538765	0.0193819	0.2689265
Hgf	-0.5159045	3.7835914	0.0194049	0.2689481
LOC118568257	0.8016474	1.2475019	0.0194181	0.2689481
Chil5	-0.8548203	2.5343788	0.0194484	0.2691277
Acy1	0.5366547	2.6295632	0.0195412	0.2701723
Fosl2	-0.4576206	6.9197507	0.0195975	0.2705957
Gde1	0.3609246	5.1258096	0.0196066	0.2705957
Msantd3	-0.3658008	4.4332929	0.0196442	0.2708733
Traf3	-0.3674037	5.484507	0.0196676	0.2709564
Abhd2	-0.3047025	6.0065864	0.0197092	0.271192
Il27	-0.9997699	-0.4838845	0.0197196	0.271192
Prps2	0.2836694	5.2670527	0.0197646	0.2715711
Gm10282	-0.3872206	6.9501434	0.0197873	0.2716437
Grap	0.5882621	3.9069944	0.0198822	0.2727047
Sec22c	-0.3579959	4.4674424	0.0199161	0.2727538
Mamdc2	1.0002525	3.005789	0.0199208	0.2727538
Mfsd14b	-0.3061344	7.4187477	0.0200049	0.2729168
Tjap1	0.3193273	4.3016823	0.0200067	0.2729168
Akap6	-0.7606987	2.3435083	0.020018	0.2729168
Gm32196	0.7267719	2.9524064	0.0200338	0.2729168
Jsrp1	-2.2982012	-0.6539722	0.0200455	0.2729168
Wdr72	1.4987334	-0.1950336	0.0200493	0.2729168
Ankrd44	-0.3554126	6.7031434	0.0200555	0.2729168
Has2	-1.1456255	0.896342	0.0201174	0.2735197
Fer	-0.3557794	4.2268265	0.020158	0.273771
Myh4	-2.2301568	4.0352464	0.0201711	0.273771
Serpinb1a	-0.4127031	8.3698787	0.0202078	0.2739091
Zc3h7b	0.24952	5.8341377	0.0202165	0.2739091
Samd1	0.3429908	6.1421069	0.0202384	0.2739666
Casq1	-2.2442989	1.2114423	0.0202667	0.274112
Hk2	-0.3804462	5.3398362	0.0203013	0.2743404
Psmb4	0.3180319	6.8468885	0.0203324	0.2745232
Hoxa6	-0.7806735	0.5658894	0.020417	0.2750905
1700037H04Rik	0.3371638	5.9574921	0.0204351	0.2750905
Aldh1l1	-1.0086246	1.7519035	0.0204368	0.2750905
Slc7a4	0.7693712	0.8011357	0.0204452	0.2750905
Gm32620	-1.426923	-0.6270881	0.0204802	0.2752627
Acer3	-0.3166294	5.7922173	0.0204934	0.2752627

Prom1	-0.4907093	5.3853938	0.0205274	0.275481
Pgm1	-0.3652146	4.5817199	0.0205718	0.27584
Avl9	-0.2776742	5.3528389	0.0206658	0.2768618
Rmdn3	0.3812718	5.6230146	0.0206889	0.2769316
Med12l	-0.449248	4.610861	0.0207449	0.2774427
Manbal	0.4300733	3.759892	0.0208069	0.2778099
Slain2	-0.312139	6.7555946	0.0208191	0.2778099
Acd	0.2881502	4.784482	0.0208376	0.2778099
Pfkfb4	-0.259097	5.7958746	0.0208438	0.2778099
Mob1b	-0.3690161	5.4505631	0.0208924	0.2782191
Stxbp2	-0.3285684	6.8481678	0.0209304	0.2784867
Zfp871	-0.2839437	7.2272783	0.0209881	0.2788042
Capza1	-0.3135184	7.8629691	0.0209901	0.2788042
Gnb1l	0.6384681	1.5974868	0.0210397	0.2792254
Ccdc134	0.459622	3.6947582	0.0210636	0.2793039
Raph1	-0.4618653	5.2497517	0.0211241	0.2798678
Tnfrsf13b	0.36548	5.4563543	0.0212308	0.2809583
Casp4	-0.4707402	3.6847317	0.0212426	0.2809583
Siglech	-0.6717821	3.7961444	0.0212678	0.281041
AB010352	-0.44582	3.5804363	0.0212874	0.281041
Gm32249	0.702898	2.8665963	0.021303	0.281041
Tusc3	0.3489138	4.7352123	0.0214242	0.2824
Uba6	-0.3493556	4.7812705	0.0215095	0.2832839
Inpp1	-0.3858154	4.5993143	0.0215573	0.2836741
Tmem216	-0.3056542	4.6421171	0.0215958	0.2839396
Celsr3	-0.5588871	2.8569336	0.0216269	0.2841089
9930111J21Rik1	-0.4860709	3.5336498	0.0216801	0.2845667
Ccdc125	-0.3894162	5.2987937	0.0219535	0.2878809
Ddx56	0.3156724	4.3882647	0.0219696	0.2878809
Eps8	0.7315835	4.0342793	0.021998	0.2880104
5530601H04Rik	-0.4673849	3.4913077	0.0221034	0.2890364
Mif	0.3452601	6.3025412	0.0221135	0.2890364
Mrpl28	0.290854	5.1603583	0.0221395	0.2891331
Zyx	-0.4000703	8.170315	0.0221777	0.2893894
Mfsd14a	-0.2503406	6.2354358	0.0222487	0.2900718
Rtn1	-1.2325838	-0.273161	0.0222765	0.2901921
Rybp-ps	-0.5625353	3.2031586	0.0223415	0.2903161
Cytl1	-1.5388373	1.3006533	0.0223534	0.2903161
Ccdc74a	0.9421357	0.6760615	0.0223559	0.2903161
Pdlim4	0.717842	1.5284937	0.0223607	0.2903161
Dnajc21	0.2747851	5.2928046	0.0224256	0.2909158
Cacna1a	-0.7957652	0.9734011	0.0224926	0.2915418
Trem2	0.6768238	3.4537722	0.0225496	0.2916447

Il18r1	-0.5350706	3.4787249	0.022552	0.2916447
Sh2d3c	-0.3045706	5.6921728	0.0225682	0.2916447
Muc13	-0.3975146	4.3273307	0.0225756	0.2916447
Slc52a2	0.4367318	2.8279061	0.0225977	0.2916882
Dennd1b	-0.3034426	5.2431949	0.022684	0.2925593
Plac9a	1.2694909	2.3249081	0.0228562	0.2943658
Nabp2	0.2823049	5.1315807	0.0228748	0.2943658
4931406C07Rik	-0.3008972	4.5628778	0.0228808	0.2943658
Ppm1l	0.3132245	4.6075251	0.0229151	0.2945636
Hspbp1	0.2995124	4.6244605	0.0229564	0.2948497
Tes	-0.3533166	4.9920115	0.0230164	0.295197
Vegfc	0.590775	3.0250001	0.0230337	0.295197
Sp6	0.9020007	2.2230612	0.0230444	0.295197
Tubb2b	0.847464	2.3300044	0.0230873	0.295197
Svip	-0.3565275	5.7707329	0.0230955	0.295197
Ankle1	0.4619024	5.4561733	0.0230973	0.295197
Pf4	-0.3545782	8.2530697	0.023186	0.2957383
F11r	0.3525803	4.6274403	0.0231887	0.2957383
Gng5	-0.2837376	6.4313174	0.0231967	0.2957383
Kcna3	-0.5951281	3.43304	0.0232337	0.2959684
LOC118567469	0.4105726	3.1923225	0.0233195	0.2965517
Tceanc2	-0.3026947	4.7162201	0.023326	0.2965517
Ces2e	-0.9782607	0.5040976	0.0233367	0.2965517
Cxadr	-0.4671527	3.180008	0.0233722	0.2967605
Snx2	0.250571	7.1644064	0.023474	0.2978091
Tmem132e	0.8733622	1.6472676	0.0234954	0.2978376
Rpl7-ps9	1.2514753	-0.3276068	0.0236026	0.298953
Ppcdc	0.3773919	3.91077	0.0236583	0.2994
Atp2c1	-0.2640191	7.1920907	0.0237202	0.2994
Adora2a	-0.5701782	2.3267346	0.0237274	0.2994
Insr	-0.2659805	6.3244082	0.0237669	0.2994
Cystm1	-0.8647156	1.3225448	0.0237963	0.2994
Cpt1c	0.8536654	0.9693192	0.0238027	0.2994
Itgad	-0.6008243	4.4158833	0.0238037	0.2994
Ptpn4	-0.3344853	5.1213505	0.0238162	0.2994
St6gal1	0.3795827	5.3299522	0.0238272	0.2994
Btrc	0.3237991	6.0465893	0.0238304	0.2994
Igkv4-79	-0.9970248	1.0652151	0.0238664	0.2996107
Kctd12b	-0.4582815	4.3128558	0.0239363	0.3002463
0610030E20Rik	-0.2668193	6.4869753	0.0239597	0.3002974
Adat3	0.7341317	1.0613536	0.0239888	0.3004199
Gm10614	-1.1554031	-1.0141423	0.0240252	0.3006338
A930009A15Rik	-0.9238868	0.8229422	0.0241381	0.3012842

Tmsb10	0.3416038	9.0913481	0.0241662	0.3012842
Lipa	-0.3955191	6.1640977	0.0241875	0.3012842
Zfp971	-0.3795015	4.8995176	0.0241883	0.3012842
Rnd1	-0.5073684	3.9256677	0.0241992	0.3012842
Gnaz	-0.442796	5.2950072	0.0242027	0.3012842
Sema4f	-0.5529179	2.0944024	0.0242177	0.3012842
Sfxn5	-0.3904667	3.9498263	0.0242321	0.3012842
Kif3b	-0.2746464	5.5935988	0.024293	0.3018003
Nelfcd	0.279338	5.8525467	0.024357	0.3020106
Etl4	-0.6259838	3.7484339	0.024366	0.3020106
Mzb1	0.513035	4.5742998	0.0243682	0.3020106
Aldh3b1	-0.3524554	5.8773113	0.0243918	0.3020621
Cd44	-0.2597851	8.4519735	0.0244341	0.3023449
Nbeal1	-0.4249825	4.7177254	0.0244848	0.3027314
Apod	-1.1646925	1.4781511	0.0245184	0.3028708
Rrp1	0.2407079	6.9110242	0.0245463	0.3028708
Gm38641	1.2771191	-1.0937978	0.0245545	0.3028708
Ifitm10	-0.9121017	0.8120286	0.0246034	0.3029964
Pdlim3	-2.0429245	-0.7551782	0.0246036	0.3029964
Ncf1	-0.3245398	9.1834811	0.0246287	0.3030659
Mib1	-0.2835286	6.4369935	0.0246929	0.3034799
Rab5b	-0.3096599	6.7334351	0.0247014	0.3034799
Tmem45a2	-1.1094661	2.1124671	0.0247508	0.3037126
AA986860	0.6186246	2.7164716	0.0247594	0.3037126
Dync2li1	-0.6114438	1.5878119	0.0248209	0.3039544
Fam71b	-0.7332251	2.8242813	0.0248325	0.3039544
Nsmce3	0.3290772	4.4333704	0.0248488	0.3039544
Osbpl3	-0.4341559	3.2499929	0.0248831	0.3039544
Flna	-0.4022956	9.9943415	0.0249039	0.3039544
Nucb2	-0.4172393	5.4231474	0.0249171	0.3039544
Acvr2a	0.4152639	3.4947905	0.0249322	0.3039544
Rpl35a	0.5715696	7.9411865	0.0249354	0.3039544
Rell1	-0.3138247	5.6854574	0.0250395	0.3049839
Sft2d2	-0.3253616	6.4111669	0.0250771	0.3052026
Anp32b	0.2955431	9.503256	0.0251481	0.3058272
Gm35035	0.7366667	1.5047234	0.0251743	0.3059068
Selenoo	-0.3365741	4.4738974	0.0252695	0.3068242
Alcam	-0.4833554	4.8479567	0.0253166	0.3068773
Igkv4-59	-0.7288409	2.9173202	0.02533	0.3068773
Itprip	-0.3551782	5.6143879	0.0253331	0.3068773
Fam53b	0.3879921	7.4706713	0.0255345	0.3088539
Ethe1	-0.3485878	5.3614736	0.0255424	0.3088539
Gm12191	-1.2098859	3.4745389	0.0255558	0.3088539

Psrc1	0.4633422	3.7557702	0.0256434	0.309672
Aph1b	-0.4441676	4.1364176	0.0256969	0.3100585
Gdf15	-1.2570641	-0.2192157	0.0257205	0.3100585
Mrpl2	0.3583037	4.7503583	0.0257352	0.3100585
Brix1	0.2914788	5.833683	0.0258698	0.3111219
Tnfsf13os	-1.2071378	0.2501795	0.025902	0.3111219
Cep85l	-0.4439876	4.7528607	0.0259137	0.3111219
Atf4	0.3458993	7.8024064	0.0259282	0.3111219
Clec4g	-0.6127972	1.1790015	0.025953	0.3111219
Cask	-0.3295133	4.4988337	0.0259625	0.3111219
Tpm4	-0.2959554	8.7489151	0.0259745	0.3111219
Psmc5	0.264323	6.2561299	0.0259835	0.3111219
Krt18	-1.0606831	-0.9124688	0.0260085	0.3111816
Pla2g12a	0.4222591	5.1084965	0.0260458	0.3113735
Cdc42ep2	-0.4966404	3.167771	0.0260646	0.3113735
H2-Eb1	0.6872293	6.8051911	0.02614	0.311928
Dclk1	0.6147532	2.6715778	0.0261545	0.311928
Dnah1	0.9051166	-0.1503573	0.0261712	0.311928
Gm13212	1.0333491	3.6831693	0.0261996	0.3120276
Naa10	0.2857468	5.2091063	0.0262538	0.3122905
Tbcd	0.3294418	5.4952528	0.0262618	0.3122905
Clec5a	-0.3278388	6.8930396	0.0262941	0.3124353
Tmcc1	-0.3237706	5.7242835	0.0263275	0.312594
Gpr153	0.7736201	3.7250279	0.0264481	0.3136144
Eif4ebp3	0.6550313	3.0137758	0.0264538	0.3136144
Jhy	0.6801981	2.5106953	0.0265396	0.3143594
Smim6	-0.8289601	1.7325116	0.0265571	0.3143594
Gm34084	-0.5371538	3.1958143	0.0266485	0.3152025
Cwf19l2	-0.3063097	4.8429891	0.0267619	0.3155539
Grpel2	0.2930278	4.5293613	0.0267833	0.3155539
Myo5a	-0.345195	6.9846626	0.0267946	0.3155539
Hypk	0.3325226	6.7984393	0.0268568	0.3155539
Gm10603	0.7708444	3.8050925	0.0268579	0.3155539
Angptl2	0.5877434	3.4905572	0.026865	0.3155539
Ppox	0.4675549	6.8044584	0.0268733	0.3155539
Ptprn	-1.0281602	0.2146132	0.0268789	0.3155539
Elavl1	0.2545492	6.5836797	0.026887	0.3155539
Eri1	0.2616018	6.8926625	0.0269166	0.3155539
Gm1866	0.7680972	1.4921928	0.0269456	0.3155539
Gm46781	-0.9232778	-0.354494	0.0269474	0.3155539
Agrn	0.5479726	5.2429676	0.0269574	0.3155539
Mrpl34	0.4047733	5.0247081	0.0269623	0.3155539
Ngef	-1.0410498	1.4561057	0.0270527	0.3159306

Lgalsl	-0.2720631	5.9158195	0.0270728	0.3159306
Pkia	-1.1365689	0.0754939	0.0270815	0.3159306
Dhx58	-0.5140994	3.4194667	0.0270822	0.3159306
Ccbe1	0.5092451	2.7028444	0.0271009	0.3159306
Irak3	-0.306807	5.2080173	0.027119	0.3159306
Usp9x	-0.3834889	7.304065	0.0271366	0.3159306
Mpp5	-0.2809494	5.057351	0.027284	0.3174091
Nxt2	-0.375744	3.9660228	0.0273111	0.3174868
Klra4	-1.043301	-0.1138638	0.0273753	0.3179394
Flot1	-0.2762666	6.1959576	0.0274076	0.3179394
Serpinf1	0.8649465	7.6696644	0.0274235	0.3179394
Arhgap27	0.3408904	5.2894064	0.0274318	0.3179394
Rapgefl1	0.8994438	2.8458764	0.0274549	0.3179695
Bglap2	1.1524177	7.9350598	0.0275759	0.318979
C1qtnf12	0.36478	6.0991573	0.0276176	0.318979
Lpcat1	0.3448503	7.1745348	0.0276316	0.318979
Kif16b	-0.3214898	4.2871619	0.0276323	0.318979
Add3	-0.3363948	7.2335018	0.0276599	0.318979
Gm35853	-0.7886831	2.6547265	0.0276651	0.318979
Cyria	-0.360698	4.4783151	0.0277694	0.3199441
Znhit1	-0.249861	5.348037	0.0278898	0.320673
Pstpip1	-0.2665059	6.2238464	0.0278914	0.320673
Ddx31	0.4171051	3.1861411	0.0278945	0.320673
Gm11223	0.6014397	1.7536116	0.0280041	0.3210428
Trim28	0.2490508	7.3485124	0.028036	0.3210428
Plekhg3	-0.3591391	5.2127496	0.0280472	0.3210428
Hdac10	0.4951352	3.3657209	0.0280681	0.3210428
Gm34405	-0.9552838	-0.2330653	0.0280692	0.3210428
Rgs14	-0.3797781	4.9661423	0.0280814	0.3210428
1600010M07Rik	-0.6572205	2.5864804	0.028097	0.3210428
Gm39138	-2.0078754	0.1069092	0.0281013	0.3210428
AA467197	-0.5697356	4.5588912	0.0281351	0.3210428
B4galt5	-0.3912795	4.457146	0.0281486	0.3210428
E2f1	0.3021471	5.7138135	0.0281537	0.3210428
Slamf8	0.6082011	1.06187	0.0282003	0.3213388
Mgst2	-0.3914676	5.0986613	0.0282721	0.3219209
Ankrd12	-0.3018814	6.3171646	0.0283214	0.3222464
Ankrd46	-0.2724502	4.8362557	0.0284187	0.3231175
Fam217b	-0.3106316	4.5245073	0.0285022	0.3238063
Nfia	0.4048511	4.7324099	0.0285252	0.3238063
Ddx28	0.3660147	3.6345504	0.028543	0.3238063
B4galt6	-0.4635043	5.9448235	0.0285626	0.3238063
Fancf	0.4463664	2.7711572	0.0286244	0.3242705

Shf	0.4360057	3.3471703	0.0286646	0.3243246
Maz	0.2614146	8.14652	0.0286971	0.3243246
Hnrnph3	0.269077	5.5523977	0.0287047	0.3243246
Triobp	-0.2587589	6.5807865	0.0287136	0.3243246
Cflar	-0.3084885	6.67778	0.0287334	0.3243246
Kin	0.4096648	4.9965217	0.0287645	0.3243484
Nrp2	-0.53279	7.6193063	0.0287772	0.3243484
Ice1	-0.2398675	5.7192156	0.0288362	0.324648
Mbnl1	-0.3102783	9.0707416	0.0288455	0.324648
Myo10	-0.4770366	6.0237117	0.028886	0.3247871
Cars	0.3028479	5.5113336	0.0289079	0.3247871
Atp2b1	-0.2957244	7.0388441	0.0289206	0.3247871
Tmx3	-0.2687096	6.5729923	0.0289782	0.3251036
Ablim1	0.5881009	5.4747959	0.0290113	0.3251036
Thsd7b	1.1974164	-0.9112061	0.0290215	0.3251036
Sema5a	-0.6286054	4.9464108	0.0290323	0.3251036
Gpr141	-0.5265354	5.4752465	0.0291199	0.3255098
Atp5g3	0.277827	7.7226768	0.0291296	0.3255098
Gtf3a	0.2935444	4.7743825	0.0291314	0.3255098
Rab8b	-0.2694367	7.8183984	0.0292108	0.3260201
Lig4	0.4972509	4.0699707	0.029219	0.3260201
Rubcnl	0.4789633	3.4884574	0.029253	0.3261659
Fyb	-0.2778333	7.3280334	0.0292796	0.3262289
Adam33	0.9029797	3.0676617	0.0293087	0.3263193
Gm8181	-1.1559192	-0.8539884	0.0293669	0.3265753
Bax	0.2858122	5.1338612	0.0293737	0.3265753
Gm50599	1.0864066	-0.5670633	0.0294542	0.3272358
Gm40181	1.1373998	-0.3994648	0.0295253	0.3275402
Gm42281	-0.6882615	0.9435286	0.0295337	0.3275402
Cntrob	0.3059328	4.806045	0.0295574	0.3275402
Gm40309	0.8364421	1.369671	0.0295791	0.3275402
Rbm8a	0.2872333	6.8497606	0.0296026	0.3275402
Lrrfip2	-0.2481561	6.171899	0.0296079	0.3275402
Snhg8	0.4830242	5.9208297	0.029752	0.3288215
Lair1	-0.4767902	4.8363574	0.0297872	0.3288215
Fam160b2	0.374127	4.7652225	0.0298067	0.3288215
Samd8	-0.3666899	5.3943798	0.0298271	0.3288215
Rsl24d1	0.264429	5.6251963	0.0298294	0.3288215
Trim12c	-0.3331116	5.8941702	0.0298666	0.3289977
Phgdh	0.3037364	5.0722053	0.0298994	0.3291068
Usp36	0.321858	4.3649622	0.0299246	0.3291068
Tet2	-0.3080699	5.2320751	0.02996	0.3291068
Eef1g	0.2604745	8.8470278	0.0299699	0.3291068

Hspbap1	0.4132762	3.8382073	0.0299823	0.3291068
Gm12758	1.2930483	-0.8508281	0.0300187	0.3292741
Gm9733	-0.6314636	3.2239206	0.0301218	0.3301728
Gm22009	-1.4478303	-0.7499849	0.0301746	0.3305184
Myl1	-1.8878347	3.2101547	0.0302261	0.3308496
Lepr	-0.2956777	6.724533	0.0302822	0.3312298
Zc3h12d	0.4648001	2.9569634	0.0304886	0.3330519
Thnsl1	-0.7482791	1.0228956	0.0304932	0.3330519
Nt5dc1	-0.3649548	4.6049059	0.030513	0.3330519
Brwd3	-0.2816245	5.2628924	0.0305534	0.3330822
9430015G10Rik	0.3817206	4.2514634	0.0305639	0.3330822
Exoc1	-0.2772441	5.2450788	0.03058	0.3330822
Apol6	-0.8283318	2.0250575	0.0307652	0.3348652
Smco3	1.078725	-0.7539518	0.0308514	0.3355683
Pde10a	-0.5651618	3.8623211	0.0308968	0.3358271
Prcc	0.2648056	5.3759772	0.0309336	0.3359926
Adamts4	-0.7706006	2.1973026	0.0309809	0.3362715
Rapgef2	-0.3224371	4.5988914	0.031077	0.3367369
Gm10184	-0.596786	1.3390061	0.0311099	0.3367369
Tanc2	-0.4089184	3.5249426	0.0311591	0.3367369
Otub1	0.2441261	5.5812757	0.0311645	0.3367369
Mef2a	-0.2716795	6.931122	0.0311741	0.3367369
Gm41659	-0.694862	2.4125438	0.0311792	0.3367369
Smpdl3a	-0.3121667	6.7866598	0.0311941	0.3367369
LOC115488162	0.5498626	2.4823858	0.031197	0.3367369
Thy1	0.5359125	3.1723291	0.0312935	0.3373514
Arap2	-0.4156942	4.4577065	0.0312973	0.3373514
Myzap	0.6397343	2.6042716	0.0313199	0.3373611
Dusp6	0.4079511	5.6307677	0.0313464	0.3374133
Adamts1	-0.5053263	3.0756323	0.0314334	0.3381153
Limd1	0.2648969	6.068557	0.0314873	0.3382932
Hgsnat	-0.3813047	4.4143683	0.0314934	0.3382932
Myl12a	-0.286631	7.7242234	0.0315239	0.3383331
Kctd3	0.2741731	4.9823664	0.0315406	0.3383331
Tor4a	-0.2712831	5.7679745	0.0316074	0.3383788
Rnf13	-0.286683	5.9267395	0.0316089	0.3383788
H2az2	0.2813656	6.5693209	0.0316102	0.3383788
Armc10	0.2741724	4.7179415	0.0317328	0.3394581
Bcl2l1	0.3995773	7.0602306	0.031762	0.3395365
Numb	-0.3600673	5.7359951	0.0318201	0.3398103
Serinc5	0.3627478	7.0934813	0.0318313	0.3398103
Klf5	-0.5468494	3.8706919	0.0319028	0.3403403
Gm9949	-0.6162031	1.9486911	0.0319261	0.3403552

Thumpd1	0.2986362	5.049579	0.0319954	0.3407073
Pnkp	-0.2883527	6.6665215	0.0320029	0.3407073
Gm38424	-0.784127	1.2065432	0.0320599	0.3409323
Adcy9	-0.3652666	4.4960459	0.0320679	0.3409323
Tgoln1	-0.2846001	6.8517621	0.0320973	0.3410116
C330026H20Rik	1.3067017	0.0316819	0.0323212	0.3431561
Pnck	-0.8456998	-0.4012045	0.0323604	0.3433377
Il2ra	1.1941206	3.0978545	0.0324052	0.3435791
Hjv	-2.0670554	-1.1462172	0.0324682	0.3440125
Cd274	-0.5153746	2.6212696	0.0325145	0.3442681
Hes6	0.4137795	4.7371195	0.0325465	0.3443728
Banf1	0.2613783	6.4185361	0.0326047	0.3447538
H4c9	0.5253901	3.2428194	0.0327831	0.3464056
Dennd11	-0.3676442	5.0276417	0.0328371	0.3467397
Exd1	-0.7848297	0.2996806	0.0329034	0.3472044
Serpina3f	0.7322523	2.7643187	0.0329742	0.347623
Setbp1	-0.563756	2.4454887	0.0329878	0.347623
Otub2	0.3812934	4.4311335	0.0330641	0.3481918
Nubp1	0.2859084	4.816664	0.0331565	0.3484163
Ctsk	0.7423386	8.8753729	0.0331646	0.3484163
Dele1	0.2604639	4.9427186	0.033171	0.3484163
Zbtb37	-0.2759571	4.9306596	0.033175	0.3484163
Rab27a	-0.3405033	6.9799685	0.0331996	0.3484384
Tmem156	-0.2932345	4.336574	0.0332534	0.3486149
Paqr3	-0.6392561	1.4711413	0.0332656	0.3486149
Ppm1d	0.28368	5.6302623	0.0333005	0.3486149
F2rl2	-0.3990298	5.523059	0.033306	0.3486149
Rsph1	0.5540179	2.4740587	0.033404	0.3494053
Prpsap1	0.3141963	4.8056217	0.0335394	0.3505864
Prelid2	0.6464102	1.4023929	0.0335828	0.350628
Mfap3l	-0.3348301	4.8889218	0.0335885	0.350628
Psat1	0.2612618	5.641376	0.0336739	0.3512834
Sms	0.2897959	5.368703	0.0337115	0.3513064
Faap24	0.3667564	3.8629177	0.0337213	0.3513064
H2-Ab1	0.4935719	6.9271885	0.0337465	0.3513342
Sgta	0.2603921	5.6576439	0.03378	0.3514476
Utp3	0.2574975	5.9721697	0.0338304	0.351525
Gbp4	-0.5127214	3.2735946	0.0338327	0.351525
Nqo2	-0.2918907	5.362529	0.0339177	0.3521737
Mgam	-0.6114122	5.4579991	0.0339905	0.3526943
Pold1	0.2997919	6.2856859	0.0340169	0.3527063
Gm8518	1.6026416	2.1927455	0.034037	0.3527063
Timm13	0.2791301	5.2459612	0.034097	0.3529237

Ier5l	0.7883681	0.8598491	0.0341034	0.3529237
Cbr2	-0.8988574	3.9179349	0.034149	0.3529367
Akap12	0.6998791	6.248081	0.03415	0.3529367
Zfp653	0.4565049	3.1207617	0.0342155	0.3532151
Sos2	-0.3568584	6.0188238	0.0342224	0.3532151
1700012D01Rik	-1.1024984	-1.0841077	0.0342457	0.3532213
Rtcb	0.2171752	6.7114495	0.034271	0.3532478
Rsbn1	-0.2599577	6.2133882	0.0344506	0.354864
Xrcc1	0.2631511	5.1680744	0.0344845	0.3549776
Hdac4	-0.3944499	4.8281371	0.0345894	0.3556862
Dubr	0.7177542	1.2116241	0.034599	0.3556862
Sae1	0.2431096	6.3404489	0.034656	0.3558096
Kmt5c	0.2298429	5.9814877	0.0346568	0.3558096
Drc7	0.8401545	1.3038544	0.0347156	0.3558544
Stx1a	0.8604983	0.4412033	0.0347273	0.3558544
Myh7	-2.5233244	0.4894182	0.0347298	0.3558544
Paqr7	-0.2538755	4.9917691	0.0347951	0.356275
Gm15610	1.0599679	0.4737846	0.0348373	0.356275
Alox12	-0.3858332	7.0146836	0.0348396	0.356275
Tmem201	0.3624682	3.5097553	0.0349094	0.3564638
Suox	0.4289082	4.9065135	0.0349112	0.3564638
Wbp1l	-0.2489356	6.1373506	0.0349268	0.3564638
Hmbox1	-0.3214446	6.2809657	0.0349586	0.356555
Slc15a5	1.60241	0.8869478	0.0350098	0.3568432
Ndor1	0.2746525	4.4295632	0.0350686	0.3571365
G0s2	-0.4453139	4.7999516	0.0351017	0.3571365
Mapk8ip1	0.9678802	0.6668772	0.0351075	0.3571365
Lca5	0.856049	1.0618548	0.0351801	0.3573192
Zcchc7	0.2982216	4.9539078	0.0351849	0.3573192
Cyp2s1	1.4404447	0.1280317	0.0352121	0.3573192
Cdc25b	0.3784838	7.5491235	0.0352456	0.3573192
Dmac2	0.3865307	3.8299855	0.0352468	0.3573192
Set	0.245658	8.3519066	0.0352633	0.3573192
Hba-a2	0.5594939	11.870109	0.0353024	0.3574028
Ryk	0.363245	5.4453171	0.035337	0.3574028
Hcar2	-0.6239193	1.9253175	0.0353405	0.3574028
Meis1	-0.4082519	3.6946308	0.0354326	0.3580824
Ifi204	-0.3945629	4.4852623	0.0354537	0.3580824
Lilra5	-1.0321784	0.8344257	0.0355737	0.3583078
Gm35107	1.4583987	0.0229988	0.035578	0.3583078
Stim2	-0.2603271	5.3492285	0.0356094	0.3583078
Vars2	0.3389547	3.6080809	0.035613	0.3583078
Pcp4l1	-0.5611591	3.3126399	0.0356343	0.3583078

Samd11	0.5350612	5.5825549	0.0356655	0.3583078
Vps13a	-0.3493656	6.1863009	0.0356829	0.3583078
Xcl1	-0.6269238	1.2722475	0.0356925	0.3583078
Ifit2	-0.3736687	4.3765059	0.0357037	0.3583078
Diablo	0.2926347	4.9316261	0.0357064	0.3583078
Trir	0.2853591	6.8616568	0.0357574	0.358457
Cmpk2	-0.317641	5.5881721	0.0357693	0.358457
Pwwp3a	0.2691675	5.3390128	0.0358077	0.358457
Gm5977	0.9732638	-0.7133977	0.0358493	0.358457
Hspa4l	-0.2895653	6.2906214	0.0358584	0.358457
Stfa2l1	-0.5165579	5.1773273	0.0358595	0.358457
Sh3tc2	0.4613393	5.1931443	0.0359274	0.358905
Vdr	-0.5381861	4.9735298	0.0359539	0.3589393
Gm9493	-0.5791137	1.6842258	0.0360965	0.3600138
Haus4	0.3627294	5.1118309	0.0361079	0.3600138
Il15	-0.5895349	2.98505	0.036153	0.3600766
Kcnk6	-0.3157056	5.0362147	0.0361605	0.3600766
LOC115489886	0.6893219	0.7597562	0.0361988	0.3602114
Rb1cc1	-0.2437077	6.7308646	0.0362427	0.3602114
Pdgfrl	1.0259115	2.5303888	0.0362435	0.3602114
Dcun1d3	-0.4005264	3.5400365	0.036271	0.3602548
Usf1	0.2609039	5.4583113	0.03632	0.3605109
Rpl28	0.2318823	9.6388173	0.0363828	0.3609042
Golga3	-0.2322032	5.7816025	0.0364443	0.3611778
Nwd1	-0.7589119	1.3283736	0.0364568	0.3611778
Dhrs13	0.4550588	2.7185304	0.0365031	0.361343
Gtf2ird2	0.5123024	2.7202487	0.0365199	0.361343
Emid1	0.5822752	3.0595214	0.0365529	0.3614387
Rps24-ps3	0.4428479	3.777339	0.0366207	0.3618797
R3hdm1	0.2292737	6.4750951	0.0366851	0.3622857
Sema3c	-1.3915203	-1.0765824	0.0367644	0.3624599
Srl	-0.5279724	2.6811716	0.0367655	0.3624599
LOC105246914	0.9590594	1.4362592	0.0367727	0.3624599
Cdo1	0.701177	5.6764453	0.0368536	0.3630276
Ropn1l	-0.8793304	-0.3443487	0.036922	0.3633819
Myoz1	-2.1016044	0.9319143	0.0369363	0.3633819
Gpnmb	-1.2801337	2.1713218	0.0369596	0.3633819
Arpc2	-0.3280676	8.9788339	0.0370284	0.3638281
Impg2	-1.2177418	0.4211387	0.0371267	0.3644939
Prr13	-0.2601787	7.3828664	0.0371648	0.3644939
Jmjd7	0.8399485	0.3738529	0.037177	0.3644939
Mrto4	0.3429363	4.8490537	0.0371978	0.3644939
Hhip	-1.1256893	-0.1895653	0.0372153	0.3644939

6430590A07Rik	-0.7511145	0.2670705	0.0372753	0.3644939
Mt1	0.3719836	6.3481454	0.037303	0.3644939
Eef2k	0.2902818	6.3104341	0.0373177	0.3644939
Ak8	-0.9231358	-0.34129	0.0373231	0.3644939
Tmtc1	-0.5491506	1.881576	0.0373305	0.3644939
Elane	-0.3893746	9.311824	0.0373912	0.3646843
Ptprj	-0.3457844	7.1412325	0.0374097	0.3646843
Cebpd	-0.3309321	6.4271395	0.0374204	0.3646843
Gm40372	1.0597644	1.3682202	0.0374849	0.3650848
Imp3	0.2797728	5.1033717	0.0375084	0.3650848
Rbm24	1.1885997	1.0997399	0.0375429	0.3650882
Sccpdh	0.5061554	3.0066203	0.0375557	0.3650882
Mbd3	0.2211638	6.1944643	0.0376224	0.3652343
Ccdc186	-0.2609459	5.5750035	0.0376305	0.3652343
Zfp951	1.1453872	0.378687	0.0377324	0.3652343
2700003A03Rik	0.9424947	-0.174633	0.0377411	0.3652343
Acnat1	-1.0148795	-0.832764	0.0377486	0.3652343
Tnfrsf1a	-0.2922185	6.4282392	0.0377498	0.3652343
4932438A13Rik	-0.3416019	6.7455227	0.0377588	0.3652343
LOC68395	0.6865569	1.0779435	0.0377618	0.3652343
Gm40150	1.0656336	-0.5065371	0.0377821	0.3652343
Polr2f	0.3205594	4.9061609	0.0378227	0.3654001
H2bc18	0.7303152	1.4982669	0.037923	0.3661421
Gm9816	-1.0005271	1.403466	0.0379609	0.3662584
Kpna4	-0.2291359	7.0504186	0.0380279	0.3662584
Gm38427	-0.7439496	1.3325958	0.0380309	0.3662584
Gm46657	-0.9776814	-0.066159	0.0380336	0.3662584
Ppil1	0.290161	5.5992695	0.0380551	0.3662584
Bccip	0.2943521	5.9369095	0.0380764	0.3662584
Zswim3	-0.4259278	2.4946054	0.0381026	0.366284
Rsu1	-0.2765774	7.2693487	0.0381343	0.3663629
Lclat1	-0.3159375	4.1790287	0.0382379	0.3669589
Mospd3	0.2857339	5.4629718	0.0382436	0.3669589
Igfbp3	0.6105861	3.9380192	0.0383136	0.3672051
Efcab14	-0.2637849	5.8962058	0.0383334	0.3672051
Gm6552	1.0146311	-0.48559	0.03834	0.3672051
Hey1	0.9415288	2.606244	0.0383815	0.3673761
Nup85	0.2909533	6.1617223	0.03841	0.3674227
Zfp608	-0.3201073	5.3227383	0.0384758	0.3676775
Cldn15	-0.4458565	4.4410271	0.0384839	0.3676775
Gm5884	-0.8028551	0.0896661	0.0385399	0.3677814
Znf41-ps	-0.7091903	2.7441747	0.0385421	0.3677814
Wwp1	-0.2574442	5.7581078	0.0386048	0.3681535

Nck1	-0.2434332	5.5119141	0.038677	0.3686165
B430319F04Rik	-1.0190783	-0.3107145	0.0387169	0.3687712
Clgn	0.6938264	2.093657	0.038871	0.3700119
Il34	0.5993296	2.1272518	0.0389097	0.3701536
LOC115487419	-0.6616598	0.8237941	0.0390319	0.3709216
Abcc5	-0.2283811	6.3948912	0.0390544	0.3709216
Ube2c	0.2904375	7.8637385	0.0390661	0.3709216
Tram2	-0.5339685	2.337028	0.039096	0.3709216
Dgcr6	0.4089987	3.4441983	0.0391097	0.3709216
Erbin	-0.3053276	7.7212037	0.0391471	0.3710501
Srsf2	0.2897963	8.899022	0.0393168	0.3724325
Atg14	-0.3000734	3.9666279	0.0393693	0.3726577
Jph2	-1.6104419	-0.0435137	0.0393885	0.3726577
Dnajc12	-0.5462747	1.6162795	0.0394186	0.3727158
Arsb	-0.3490691	7.2472062	0.0394904	0.3731674
Ttc21a	-0.5066258	3.3110015	0.0395173	0.3731946
Lrrcc1	0.2961254	4.9311781	0.0395875	0.3736314
Upf2	-0.2402073	6.2419572	0.039721	0.3746632
Diaph2	-0.3802619	5.1332027	0.0398638	0.3757824
Pax5	0.7064957	6.4924096	0.0399388	0.3759413
Ccdc137	0.3254959	4.2952963	0.0399487	0.3759413
Pirb	-0.3325629	6.5868645	0.0399531	0.3759413
Ash1l	-0.2872382	6.3369044	0.0400329	0.3764639
Pan2	0.2671775	5.6118313	0.040252	0.3782962
Brwd1	-0.2769522	5.614327	0.040342	0.3789128
Gm10516	0.759194	0.4952122	0.0404244	0.3793592
Treml4	-0.4060285	3.6813743	0.0404452	0.3793592
Anapc7	0.2450576	5.3176226	0.0404627	0.3793592
Slco3a1	-0.2998849	4.6218	0.040508	0.3795553
Rpl29	-1.0855431	7.9473782	0.040571	0.3799165
Ddx39b	0.2322545	7.6301643	0.0405966	0.3799278
Wdr83	0.297261	3.8249266	0.0407852	0.3812018
Pdcd2	0.3223399	4.0627847	0.0407887	0.3812018
Igkv5-37	2.791143	0.4842419	0.0408063	0.3812018
Gm30455	-1.0787738	-0.1378645	0.040942	0.3818007
Tspan4	0.3995062	4.6434044	0.040954	0.3818007
Loxl3	0.5101297	3.6014749	0.0409685	0.3818007
Wiz	0.2854639	5.0924344	0.0409686	0.3818007
Elof1	0.2116377	5.6876474	0.041032	0.3821625
Alg6	-0.3469267	3.7127871	0.041064	0.3822321
Xpr1	-0.2855701	6.941277	0.041095	0.3822793
Snhg12	0.4922099	3.4256704	0.0411183	0.3822793
Nat8l	-0.5845072	3.6228862	0.0411604	0.3823118

Gm39326	1.0167852	0.4230309	0.0412005	0.3823118
Pag1	-0.2582464	6.4942372	0.0412369	0.3823118
Ap5b1	-0.4059501	4.5265764	0.0412496	0.3823118
Fgf13	0.7625942	1.57332	0.0412621	0.3823118
Tmem268	0.3232956	4.1183992	0.0412692	0.3823118
Myo1c	0.3413884	6.7579087	0.0413968	0.3832652
Cox4i2	0.9323129	1.1209118	0.0414984	0.3836874
Cd22	0.5789907	5.3722188	0.0415331	0.3836874
Ppp6r2	0.2801873	5.0862984	0.0415368	0.3836874
Mrc1	-0.3774523	7.1655245	0.0415411	0.3836874
Bin1	0.2767477	5.5394529	0.0416136	0.3841295
Pigq	0.3706044	8.1203568	0.0417027	0.3847233
A430035B10Rik	0.6145658	1.1290289	0.0417543	0.3847671
Dusp4	-0.7951486	1.8622969	0.0417569	0.3847671
Apc-ps1	-1.119374	-0.9248764	0.0418667	0.3855504
Map3k2	-0.3052715	5.5959295	0.0419204	0.3858167
1700017B05Rik	0.3383472	5.475661	0.0419794	0.3859589
Tmem38b	-0.3653152	4.9483731	0.0419855	0.3859589
Mndal	0.2617994	6.1520035	0.0421129	0.3866457
Aldh18a1	0.2817863	5.5876542	0.0421213	0.3866457
Brinp2	0.8871134	0.5934116	0.0421502	0.3866457
Guk1	0.4330066	4.3431596	0.0421754	0.3866457
Dok4	0.5424123	2.5420919	0.0421845	0.3866457
Zfp61	0.4670846	3.0190034	0.0422106	0.386657
E230016K23Rik	-1.2485994	-0.6172462	0.0423419	0.3876318
Gm52290	0.8845308	-0.0253508	0.0424153	0.3878571
Lratd1	1.2163333	0.9240095	0.0424325	0.3878571
Hars	0.2742253	6.9778148	0.0424414	0.3878571
Arpc1b	-0.2802154	8.4986395	0.0424717	0.3879063
Trib1	-0.4443456	4.1135907	0.0425057	0.3879892
Ccl3	0.8983196	-0.0151842	0.0426308	0.3887837
Maob	0.4922476	2.3603296	0.0426606	0.3887837
Gm33454	1.0149235	-0.2355028	0.0426677	0.3887837
Atp8b4	-0.4206113	7.3630855	0.0427127	0.3888225
Aph1c	-0.5383354	2.9835797	0.0427751	0.3888225
Jak1	-0.2307753	8.1910002	0.0427884	0.3888225
5730552O08Rik	-0.4556132	2.8100327	0.0427889	0.3888225
Fam111a	-0.2631735	8.1779779	0.0428198	0.3888225
Panx1	0.3735355	3.6838231	0.042822	0.3888225
Dennd4c	-0.315331	5.5070247	0.043011	0.3903106
Sc5d	-0.403062	4.4201417	0.0430784	0.3904983
Arhgap33	0.3393171	4.0822852	0.0430818	0.3904983
Birc5	0.3449588	7.8561412	0.0431643	0.391018

Atp13a1	0.2808578	5.5893169	0.0432837	0.3913455
Snf8	0.2766931	5.5248594	0.0432922	0.3913455
Fry	-0.4076897	4.9223184	0.0433111	0.3913455
Gm11685	0.5406308	2.3068582	0.0433137	0.3913455
Arhgap29	-0.3320047	4.3611307	0.0433472	0.3913455
Gsap	-0.2334402	5.2763785	0.0433616	0.3913455
Yars	0.2744327	5.6350046	0.0433766	0.3913455
Gm20075	-0.7801196	2.2651502	0.0434262	0.3915654
Fzr1	0.3085395	7.0072708	0.0435319	0.3922911
Tasp1	0.3958337	3.452593	0.0436189	0.3927057
Mob3a	-0.2578398	6.2575883	0.0436284	0.3927057
Dnmbp	-0.3875821	4.024757	0.0437	0.3931234
Ccdc107	0.3808835	3.5902126	0.0437488	0.3933343
Melk	0.2464931	5.0681929	0.0438286	0.3938248
Hsd17b7	-0.5949339	3.2680696	0.0438816	0.393849
Zc3h12c	-0.3521694	3.5904135	0.043882	0.393849
Gbe1	-0.3349379	5.1338767	0.0439222	0.3938549
Ets2	-0.4066346	5.1282772	0.0439454	0.3938549
Rab6b	-1.1045894	1.8559231	0.043962	0.3938549
H2-T24	-0.2895849	6.0312644	0.0439839	0.3938549
Uhrf1bp1l	-0.268025	6.190835	0.0440812	0.3944568
B3gnt3	0.4183526	2.7585129	0.0441019	0.3944568
Gas1	0.6915973	4.0619071	0.04416	0.3947496
Agtrap	-0.2762598	5.9860288	0.0442002	0.3948824
Dffb	0.3697102	3.2731733	0.0442433	0.3950399
Timeless	0.2550184	6.0234558	0.0443133	0.3954202
Mapkapk3	-0.3552305	6.1056373	0.0443367	0.3954202
H2-Ob	0.6224192	4.9271017	0.0443854	0.3955761
Rbck1	0.2121026	5.9960153	0.0444294	0.3955761
Sema3f	0.5306642	1.9997789	0.0444305	0.3955761
Chad	-1.1355218	4.6463849	0.044477	0.3957639
Supt7l	0.287742	3.8942585	0.0445354	0.3960571
B3glct	0.3063563	4.6506619	0.0445728	0.3961627
Tmem71	-0.5899945	4.7288811	0.0446121	0.396286
Pomt1	0.3109278	3.9763573	0.0446881	0.396734
Egfl6	1.3840359	0.7251711	0.0447849	0.3968033
Sema6d	-0.3335733	5.5990198	0.044799	0.3968033
Fbxo40	-1.2616003	-0.6728816	0.044821	0.3968033
Gm26782	1.305164	0.9944576	0.0448336	0.3968033
Rps6kb2	0.2944993	4.7733489	0.0448925	0.3968033
Lgals9	0.2516872	7.1036727	0.0448928	0.3968033
E330020D12Rik	0.8051136	0.7352306	0.0448937	0.3968033
C130026I21Rik	1.3457646	1.5028448	0.0448999	0.3968033

Sox17	0.7423842	1.1049247	0.0449434	0.3969029
Tspan11	-1.0377565	0.9459683	0.0449623	0.3969029
Slc7a7	-0.3571825	4.6470803	0.0449952	0.396968
Orm1	-0.5716552	4.30132	0.045065	0.3969946
Snrnp40	0.2439454	5.8565529	0.0450668	0.3969946
Zfp799	-0.4714373	2.8183029	0.0450822	0.3969946
Prr7	0.667166	1.6800829	0.045117	0.3969946
Rbm44	0.9919859	1.9253933	0.0451399	0.3969946
2310040G24Rik	-0.876436	0.5548617	0.0451513	0.3969946
Hbs1l	0.2619408	5.8797385	0.0452854	0.3979103
Fbxl20	-0.2854473	5.7215483	0.04535	0.3979103
Ubiad1	0.3402358	3.4141866	0.0453556	0.3979103
Atoh8	0.9479136	1.4077879	0.0453578	0.3979103
Lurap1l	0.9872391	2.269911	0.0454141	0.3981793
Tuba1c	0.2943443	7.154759	0.0454521	0.3982885
Pld1	-0.3693323	4.2047919	0.0456434	0.3997396
Gm34589	-0.7408714	1.1631005	0.0456933	0.3999515
Fbn1	-0.2815051	6.1584574	0.0457775	0.400463
Rac2	-0.3298189	8.948519	0.0458425	0.4006151
Myom2	-1.517775	-0.0565405	0.0458464	0.4006151
Nrm	0.2773578	5.0571391	0.0458794	0.4006788
Tspan33	0.3920201	7.7090575	0.0459076	0.4006996
Mindy1	-0.2171727	5.6446407	0.0460044	0.4013192
F10	-0.4480599	5.1476677	0.0460402	0.4014069
Spsb3	0.3791717	4.9526608	0.0461068	0.401409
Pqlc3	-0.2748486	5.4587652	0.0461122	0.401409
Slamf7	0.6042767	4.1534178	0.0461208	0.401409
Mrpl9	0.2724747	5.230166	0.0461519	0.401409
Fgd3	-0.2654134	6.0564356	0.0461695	0.401409
Deptor	-0.3857877	4.2774646	0.0462907	0.4017528
Slc30a7	-0.2618273	5.3404971	0.0463023	0.4017528
Kctd10	-0.2416698	6.3641201	0.04632	0.4017528
Gp5	-0.3786337	6.3854218	0.0463674	0.4017528
Cxcl13	1.4485544	2.891215	0.0463817	0.4017528
Ring1	0.298332	4.2269309	0.0464176	0.4017528
Ldb1	0.2323523	6.9161914	0.0464256	0.4017528
Rps6ka3	-0.2712612	6.9775293	0.0464374	0.4017528
Chp1	-0.2340937	7.1338786	0.0464415	0.4017528
Simc1	0.4223658	2.7301005	0.0465263	0.4022628
Brd7	0.2297096	6.9703996	0.0466071	0.4027367
Gzmb	-0.8223402	0.3493789	0.0467435	0.4036299
Tnfsf12	0.4522759	2.4432918	0.0467741	0.4036299
Dio2	-0.4826863	4.0387813	0.0468292	0.4036299

B3gnt8	0.4396234	3.7131134	0.0468385	0.4036299
Scaper	-0.4210979	3.8464939	0.0468833	0.4036299
Gm38500	-0.6588973	1.4070529	0.0468883	0.4036299
Npas2	-1.2989886	0.5693687	0.0468921	0.4036299
Notch3	0.5930128	4.0345508	0.0469243	0.4036839
4632428C04Rik	0.9225819	-0.4273172	0.0469511	0.4036916
Atp5g1	0.4531491	5.9645698	0.0469781	0.4037003
Zfp984	-0.4921418	4.9291361	0.0470732	0.4042942
Gm10509	-0.3666812	3.0587715	0.0471174	0.4044074
Tpd52	-0.2749471	6.6000694	0.0471384	0.4044074
Kank1	-0.5844259	4.3405278	0.0472078	0.4045108
Arhgef40	0.4012629	4.1113449	0.0472087	0.4045108
Ino80e	0.2495808	5.032787	0.0472588	0.4045108
Gtse1	0.282644	5.2273951	0.0472949	0.4045108
Mbnl2	-0.2626967	6.8247995	0.0473058	0.4045108
Ccdc63	-0.8696053	-0.4156097	0.0473065	0.4045108
Nebl	0.6950959	1.1091983	0.0473829	0.4047821
Polr2c	0.2419061	5.1824394	0.047403	0.4047821
Epas1	-0.2794959	6.6374283	0.0474163	0.4047821
Myl2	-2.198506	0.6148521	0.0474682	0.4050029
Gp1bb	-0.3186437	6.1943695	0.0475367	0.4053653
Ctc1	0.2510383	5.1253192	0.0476712	0.4060079
Itpr1	-0.2819485	5.8086244	0.0476758	0.4060079
Zfp106	-0.2668399	7.3721397	0.0476904	0.4060079
Rab6a	-0.2247091	7.566384	0.0477582	0.406363
Gstt2	0.3759492	3.0580393	0.0478828	0.4069534
Zfp462	-0.5816623	2.7436115	0.0479059	0.4069534
Septin9	-0.2910061	7.2276718	0.0479061	0.4069534
Ptpn12	-0.2243591	6.9466378	0.0480431	0.4077658
Nek7	-0.2044089	6.485978	0.0480757	0.4077658
Tlr4	-0.2789957	5.4777389	0.0480804	0.4077658
Ggh	-0.312169	4.6435384	0.0481085	0.4077815
Ppp1r14a	-0.445868	2.0295923	0.0484068	0.4100866
Sfxn1	0.2134219	6.2896165	0.048494	0.4103446
Zdhhc16	0.2986347	4.6548113	0.0485012	0.4103446
Rdh12	-0.4099426	4.7601981	0.0485164	0.4103446
Snx15	0.3818971	4.8148833	0.0485866	0.4104051
Hadh	0.2404228	5.3852088	0.0485941	0.4104051
Scamp1	-0.2471053	5.4642028	0.0486027	0.4104051
Lsm2	0.2909273	5.2607821	0.0486456	0.4105449
Cxxc5	0.4021567	4.8065257	0.0487427	0.4110176
Zbtb34	-0.3412321	5.0784489	0.0487926	0.4110176
Mtus1	-0.2833987	7.2906926	0.0487966	0.4110176

Gm36827	1.0111087	2.3972499	0.0488074	0.4110176
Ctu2	0.345178	3.2440828	0.0488504	0.4111572
Cep97	0.2838425	4.1403096	0.0488868	0.4112416
Diaph1	-0.2938713	7.5826763	0.048936	0.4112558
Tjp1	0.4449215	5.9268577	0.0489414	0.4112558
Gypc	0.3882933	7.5467946	0.0489751	0.4113166
Trappc8	-0.2512564	6.4972705	0.04903	0.4115558
Srsf1	0.2432271	9.2494277	0.0490835	0.4115665
Gm8828	1.3877641	0.4069611	0.0491315	0.4115665
Dysf	-0.4639889	3.3862285	0.0491335	0.4115665
Adamtsl1	-1.2619226	-0.6223706	0.0491371	0.4115665
Cd2bp2	0.198414	6.1913072	0.0491917	0.4116182
BC037704	-0.4946878	1.682327	0.0491962	0.4116182
F2rl3	-0.4741713	3.402359	0.049301	0.4122728
Gemin4	0.3874782	3.4728156	0.0493365	0.4123485
Gm9917	-0.7339601	0.5400987	0.049415	0.4127822
R3hdm4	-0.2344491	7.2686186	0.0495078	0.413064
Hrc	-0.7554089	2.651771	0.0495221	0.413064
Pank2	0.2387868	6.0938965	0.0495636	0.413064
Wdr83os	0.2535722	4.9594839	0.0495741	0.413064
Slc27a6	-0.7543074	0.6436857	0.0495815	0.413064
Tcp1	0.2542903	7.9769662	0.0496433	0.4133575
Fam167b	0.3897933	2.7877634	0.0497306	0.4137084
Pnp	0.7679528	6.9509427	0.0497386	0.4137084
Prss23	-0.7694191	2.4306616	0.0499695	0.4150896
E130317F20Rik	-0.871625	0.4175335	0.0499886	0.4150896
Rapsn	0.7249138	0.6008085	0.0500465	0.4150896
AW209491	-0.3568095	3.4045286	0.0501443	0.4150896
Txlnb	-0.9558218	0.0748789	0.0501652	0.4150896
Fn3k	0.4444348	5.3225981	0.0502087	0.4150896
Gm40443	-0.6564815	2.1057011	0.050226	0.4150896
Galk1	0.3390145	4.1997616	0.0502505	0.4150896
Gm9844	0.389448	3.9748339	0.0502622	0.4150896
Mcu	-0.2885277	5.2326468	0.0502759	0.4150896
Hspa1b	-0.9290759	1.4577513	0.0502915	0.4150896
Ssbp3	0.2823319	6.6964897	0.0502922	0.4150896
Hnrnpab	0.2299787	9.3990525	0.0503113	0.4150896
Camk2d	0.3539752	5.4182835	0.0503547	0.4150896
Mpv17l2	0.3365378	3.7407551	0.0504049	0.4150896
Gm34939	-0.9181163	-0.1724605	0.0504094	0.4150896
Asb13	0.4503836	2.8325475	0.0504262	0.4150896
Skap2	-0.2629549	6.9427862	0.0504714	0.4150896
Trim30b	-0.5177089	2.2402945	0.0504775	0.4150896

Septin8	0.2914732	7.0957115	0.0504928	0.4150896
Gstt1	0.4860292	3.5417905	0.0505074	0.4150896
Tmem265	0.7036577	2.3906549	0.0505416	0.4150896
Dus3l	0.2889224	4.5756832	0.0505497	0.4150896
Mapk4	-0.6504352	1.3952902	0.050562	0.4150896
Esm1	0.3103608	5.8278032	0.0506118	0.4150896
Fbxl15	0.4920701	2.2805098	0.0506206	0.4150896
Gnpda2	-0.3278115	3.9633205	0.0506252	0.4150896
Fam13a	0.4719944	2.3501961	0.0506647	0.415194
Rps12-ps5	0.6935571	0.7108127	0.05076	0.4157383
Mira	0.6124213	0.7224226	0.0508803	0.4157383
Akirin2	0.2340712	6.0229103	0.0508809	0.4157383
Phf20l1	-0.2529516	7.1206549	0.0508812	0.4157383
Flot2	-0.2912086	6.4942944	0.0508949	0.4157383
Etv6	-0.2656376	6.0859106	0.0508988	0.4157383
Loxl4	-0.6229624	4.1106035	0.0509182	0.4157383
Mrtfa	-0.3499257	6.2098685	0.05103	0.416433
Gys1	-0.3623239	5.5634764	0.0511891	0.4173589
Ndufa10	0.2054126	6.2292336	0.0512328	0.4173589
Lrfn4	0.6770565	1.4650781	0.0512492	0.4173589
Zfp300	-0.6216763	0.8009199	0.051265	0.4173589
Pdf	0.3123311	4.2185518	0.0512956	0.4173589
Fpgt	-0.3800166	2.8104571	0.0513045	0.4173589
Rfc3	0.2458002	5.1258195	0.051532	0.4189901
Bbx	-0.3242393	5.0931833	0.0516049	0.4193638
Mcm3	0.2473454	7.8506777	0.0516589	0.4195838
Man2b1	-0.3219799	7.458332	0.0516863	0.4195871
Hdac2	0.2600709	6.6822833	0.0517458	0.4198506
Ube2s	0.2570982	6.9891424	0.0518518	0.4204919
Tmem184a	-1.849576	-0.2461515	0.0519284	0.4205803
Cpne2	-0.2668319	6.0776449	0.0519575	0.4205803
Nab1	-0.2331522	5.8989269	0.051988	0.4205803
Mxd3	0.2898692	5.1162094	0.0519897	0.4205803
Prkaa2	-0.5521413	2.1714011	0.0520094	0.4205803
Fcrl6	1.2773065	-0.7728603	0.0520372	0.4205803
Neil1	0.4982119	3.1173834	0.052052	0.4205803
Zbtb1	0.22182	6.229216	0.0521871	0.4210272
Gm32483	0.723648	0.9912498	0.0521962	0.4210272
Tmem200b	0.6594675	1.6794263	0.0522413	0.4210272
Ube2r2	0.2985543	7.0938116	0.0523063	0.4210272
Mta3	0.2296543	5.8515117	0.052336	0.4210272
LOC115490435	0.8197843	0.2743332	0.0523572	0.4210272
Slc16a2	-0.5167632	1.8925105	0.0523694	0.4210272

Mapk12	0.6973275	1.6123506	0.0523696	0.4210272
Cmtr1	-0.4151371	3.7868624	0.0524255	0.4210272
Gda	-0.3523059	8.5408107	0.0524364	0.4210272
Tdp2	0.2547482	5.2711676	0.0524383	0.4210272
Zfp683	-1.0004974	-0.8975221	0.0524422	0.4210272
Rbfaos	-0.7985794	1.9240345	0.0524787	0.4210272
Gm46116	1.2312776	-0.6823608	0.0525121	0.4210272
Ms4a1	0.5259344	6.0095601	0.0525134	0.4210272
Nfkbiz	-0.3754229	3.6219397	0.052546	0.4210717
Fscn1	0.4604974	4.7221334	0.0526004	0.4211663
Gm39667	-1.0573781	-1.1857613	0.0526515	0.4211663
Gm12020	-1.2353619	0.3744851	0.0526615	0.4211663
Gpr83	-1.0428883	-0.1087279	0.0526661	0.4211663
Pdzd8	-0.2259638	7.0621806	0.0527487	0.4214088
Rn18s-rs5	0.3062262	8.0443529	0.0527538	0.4214088
Spout1	0.3007812	3.9548111	0.0527777	0.4214088
Maip1	0.3322081	3.6966521	0.0528093	0.4214449
Rpa2	0.2903116	6.0908643	0.0528675	0.4216762
Flnc	-1.2669706	0.3690006	0.0529153	0.4216762
Ltbp2	0.5360502	5.7805247	0.0529383	0.4216762
Steap3	0.3334605	6.8273831	0.0529467	0.4216762
Arhgap9	-0.267372	6.2246822	0.0529859	0.4217724
Cipc	0.2378773	5.0361774	0.0530979	0.4224475
Gm20619	0.8055387	-0.6153731	0.053167	0.4227256
Chm	-0.2920469	4.847663	0.0531872	0.4227256
LOC118568712	-1.6899388	-1.48172	0.0532866	0.4232439
Gm36723	-2.3231593	0.1484316	0.0533271	0.4232439
Pcnp	0.2195709	6.5793059	0.0533341	0.4232439
H2-DMa	0.2792853	4.8622008	0.0533987	0.4235407
Eefsec	0.3612893	3.7860157	0.0534934	0.4240756
Fn1	-0.4281136	8.8369701	0.0535445	0.4242648
Zfp69	0.560457	1.9343512	0.0535873	0.4243877
Map3k3	-0.2681925	6.4510083	0.0536457	0.4246338
Psmc4	0.2150722	6.1892349	0.0538232	0.4258216
Vamp3	-0.2183167	5.9935072	0.0539944	0.4268927
Atp11c	-0.2529183	6.1060307	0.0540134	0.4268927
Kcnn4	0.3637011	6.7115379	0.0540783	0.4271887
Idi1	-0.3608889	3.6928611	0.0542202	0.4280915
Arhgap18	-0.2870291	6.2104382	0.0542734	0.4282944
Ostm1	-0.222026	6.1710227	0.0543064	0.4283171
Usf3	-0.2292718	6.4515827	0.0543459	0.4283171
Cetn4	0.6645433	1.2849964	0.054405	0.4283171
Zbtb46	0.3833873	4.1347762	0.054407	0.4283171

Oaf	0.3603657	4.6927689	0.054414	0.4283171
Rnase2a	-1.3026278	0.9768152	0.0545486	0.4291597
Cntn3	1.074247	-0.0575123	0.05458	0.4291899
Apoc1	-1.4353216	2.2486726	0.054629	0.4293576
Txnip	-0.3298915	8.0329988	0.0547163	0.4298267
Ufd1	0.2429883	5.7267451	0.0548236	0.4304521
Edem1	-0.2670984	7.3819109	0.0548769	0.4305383
Gm7665	-0.8956284	0.8839764	0.0548899	0.4305383
4930467E23Rik	1.079577	-0.4771641	0.0549545	0.4307757
Slc27a4	-0.247817	5.866272	0.0549756	0.4307757
Il1b	-0.6961954	5.1738684	0.0550116	0.4308413
Epha7	-1.0202017	-0.1488745	0.0551341	0.4315834
Abtb2	-0.4446599	3.1601331	0.0551868	0.4317784
Ate1	-0.2692893	5.8014441	0.0552927	0.4323898
Pck1	-1.7234703	1.2521149	0.0554341	0.4330344
Cks1b	0.265915	6.6783929	0.0554527	0.4330344
Zfp979	1.4543728	2.4863834	0.0555144	0.4330344
Far1	-0.2380837	7.7586083	0.0555434	0.4330344
Pcnx	-0.3109897	5.5089682	0.0555535	0.4330344
Mylip	0.2432812	6.2958168	0.055555	0.4330344
Rnaseh2b	0.3741064	5.5186678	0.0555751	0.4330344
Mon1b	-0.252833	5.2663065	0.0555979	0.4330344
Slc35a5	-0.3164046	5.2206469	0.0556339	0.4330975
Reep3	-0.27462	6.6261818	0.0556617	0.4330975
Gm7334	0.6917563	0.9992158	0.055883	0.4346022
Cep89	0.2560805	4.5068246	0.055919	0.4346306
Hsd17b1	-0.7453796	0.5632985	0.0559426	0.4346306
Ndrg1	-0.2840741	6.1223578	0.0561355	0.4359121
Gm17619	-0.9225757	0.7543082	0.0562364	0.4360634
Trub2	0.3662351	4.3341111	0.0562583	0.4360634
Cadm1	-0.4890689	6.5146243	0.056262	0.4360634
Nme1	0.3642655	6.619639	0.0562672	0.4360634
Peg13	-0.275436	5.0966353	0.0562959	0.4360692
Bbof1	-0.6099346	0.7740102	0.0563413	0.4360726
Gramd3	0.4040792	3.7080135	0.0563852	0.4360726
Colgalt1	0.2420145	6.7244442	0.0563934	0.4360726
S100a4	0.4507395	6.4454932	0.0565058	0.4360726
Ptrh1	0.8492991	0.3873269	0.0565066	0.4360726
Rin3	-0.3696013	6.8890794	0.0565172	0.4360726
Smpd5	0.8564353	0.8305733	0.056562	0.4360726
Ttll3	-0.5739424	2.0523961	0.0565712	0.4360726
Tnk1	0.6234324	1.6206263	0.0566377	0.4360726
Slc25a45	-0.2840445	4.9648524	0.0566461	0.4360726

Gm867	0.5474083	5.205835	0.0566543	0.4360726
Nrcam	-0.5298892	2.1518555	0.0566626	0.4360726
Nkg7	-0.4020477	5.3703536	0.0566985	0.4360726
Morrbid	-0.3891141	3.2781243	0.0567035	0.4360726
Zfp652	-0.3203341	4.9158767	0.0567169	0.4360726
Gm11560	0.6752456	0.3106014	0.0568482	0.4361962
Rbp1	0.4853122	2.4591478	0.0568689	0.4361962
B230219D22Rik	-0.2571378	6.8232322	0.056882	0.4361962
Adamts3	-0.4505708	2.3715772	0.0569847	0.4361962
Nars2	0.450713	3.4715439	0.0569856	0.4361962
Dram1	-0.2732796	5.1550865	0.0570172	0.4361962
Col6a5	0.8571222	3.4638521	0.0570513	0.4361962
Foxn2	-0.2385616	5.7746648	0.0570978	0.4361962
Atp11b	-0.3100051	7.7078342	0.0571307	0.4361962
Kdm4c	-0.2236276	5.2119289	0.0571481	0.4361962
Fem1al	-1.01909	-0.6970317	0.0571735	0.4361962
Cbl	-0.3416615	7.6279897	0.0571835	0.4361962
Cfap20	0.2659963	4.2502158	0.0572388	0.4361962
Itsn2	-0.2664358	7.5876761	0.0572452	0.4361962
Zswim6	-0.3057663	4.8627975	0.0572683	0.4361962
Pop4	0.3984428	4.1801803	0.0572835	0.4361962
Gm3227	1.3381579	-0.4110291	0.0572895	0.4361962
Inka2	-0.9585656	1.3769466	0.057295	0.4361962
Abhd1	-0.6702056	1.190079	0.0573047	0.4361962
Tubb5	0.2345046	9.9574354	0.0573065	0.4361962
Homer1	-0.3416954	3.5370551	0.0573466	0.4361962
Arrb2	-0.2316568	7.8156646	0.05735	0.4361962
Pxylp1	-0.285298	5.8433062	0.0574148	0.436476
Epn1	0.2153859	7.5883776	0.0575127	0.4370069
H2ac6	1.7898047	-1.1208772	0.0576716	0.4380003
Maml1	-0.2793333	5.4411425	0.0579029	0.439542
Mks1	0.5469426	1.2427815	0.0579809	0.4399192
Vegfb	0.3031915	3.6745041	0.0580874	0.4405129
Nckap1l	-0.247494	7.7858505	0.0581525	0.4405997
Fcer2a	0.7390973	3.974871	0.0581617	0.4405997
Poli	0.3556404	3.8752233	0.0581934	0.4405997
2610318N02Rik	0.4151975	2.6402341	0.0582359	0.4405997
Pacs1	-0.2894133	5.7902052	0.0582405	0.4405997
Ifitm2	-0.2408922	7.2410232	0.0583644	0.4413225
Gm51534	0.9479856	-0.5157115	0.0584147	0.4414878
Arhgdib	-0.2948582	10.004724	0.0584905	0.4415669
LOC102634078	0.4771474	3.6328398	0.0585101	0.4415669
Pde1c	-0.7368618	0.4351985	0.0585103	0.4415669

Ccdc12	-0.261994	5.293421	0.0586008	0.441591
Emc1	0.261728	5.0042885	0.0586219	0.441591
Magi1	0.7964103	3.3396933	0.058626	0.441591
Mblac2	-0.3913677	2.4505651	0.0586383	0.441591
Dpep1	0.4116074	3.7844665	0.0586555	0.441591
LOC118568498	-0.8989211	0.2062356	0.0586903	0.4416398
Klre1	-0.9837723	0.41369	0.0588314	0.4424869
Snhg5	0.4084522	5.2682598	0.0588848	0.442512
Rab11fip4	-0.406484	4.6556821	0.0588971	0.442512
Cxcl12	0.3128377	10.383016	0.0589508	0.442512
Slc35d3	-0.3743756	3.8783299	0.0589625	0.442512
Dapk3	0.2699399	4.5170334	0.058977	0.442512
Clock	-0.2835406	4.7357284	0.0590535	0.4426332
Dusp2	0.5994231	3.6957864	0.0590748	0.4426332
Susd6	-0.1999159	6.5185963	0.0590785	0.4426332
Mef2c	-0.3018187	6.9846333	0.0592064	0.4433778
Sat1	-0.3213124	7.348982	0.0592469	0.4433926
Psmf1	0.3260049	6.225854	0.0592653	0.4433926
Atg7	-0.3628444	5.0589205	0.0594396	0.44426
Agpat4	0.4261484	4.7459131	0.059464	0.44426
Tppp	-0.6050798	0.7242648	0.059467	0.44426
Nsg1	0.5867415	2.1715399	0.0596433	0.4450611
AI463229	-0.5235143	1.4370555	0.0596672	0.4450611
Gm38426	0.6987626	0.5077024	0.0597124	0.4450611
Ucp2	0.2570768	9.9564366	0.0597298	0.4450611
Gm6430	0.8980906	-0.6454843	0.0597518	0.4450611
Gyg	-0.2841818	6.2945602	0.0598567	0.4450611
Gm10275	0.3574809	4.5958475	0.0598855	0.4450611
Cemip2	-0.2395084	5.8764707	0.0598905	0.4450611
Acap2	-0.2861638	7.2826878	0.0599002	0.4450611
Galnt3	-0.2962303	4.332398	0.0599238	0.4450611
Gm34095	1.0450909	1.5350138	0.0599318	0.4450611
LOC102631979	-0.6895942	0.8636246	0.0599327	0.4450611
Gm10146	0.7958689	0.0526226	0.0599473	0.4450611
Plagl1	0.5284424	4.7654318	0.0599748	0.4450611
Ulk4	1.1133463	-0.1777838	0.0600716	0.4454342
Emb	-0.3217875	7.5098302	0.0600824	0.4454342
Gstp2	0.9170583	-0.2860966	0.0601278	0.4455005
Stk40	-0.2404102	5.1538404	0.0601486	0.4455005
Stag2	-0.2244913	8.4434885	0.0602307	0.4458961
2610528J11Rik	-0.8805532	0.6278755	0.0602777	0.4460323
Rps4x-ps	1.0300132	-1.0308274	0.0605626	0.4474017
Gpatch2	0.3136586	4.3651231	0.0605753	0.4474017

Tigit	-0.8436496	-0.1808791	0.0606039	0.4474017
G3bp1	0.2387652	7.6120249	0.0606066	0.4474017
Mrvi1	-0.4373887	6.2964839	0.0606213	0.4474017
Mrpl48-ps	-1.7481826	1.2203612	0.0606469	0.4474017
H2bc21	0.3958172	4.151635	0.0606642	0.4474017
Gucy1b1	-0.4461587	5.0418909	0.0607682	0.4479569
Dnph1	0.3694895	2.8839984	0.0608236	0.4481525
Tmem43	-0.2475079	5.7275273	0.0609269	0.4486561
Cnot9	0.2091701	6.111099	0.0610118	0.4486561
Prkx	-0.2887277	4.7244885	0.0610121	0.4486561
1110038B12Rik	0.3659877	4.1331875	0.0610171	0.4486561
Gm46828	-0.7510667	0.5653425	0.0610433	0.4486561
5730408K05Rik	0.899039	2.1068233	0.061065	0.4486561
Flnb	-0.2657096	5.4750856	0.0611394	0.4489903
Slc38a5	0.3486924	6.3171075	0.0611816	0.4490667
2700038G22Rik	0.4743568	2.3787806	0.0612146	0.4490667
Prpf31	0.228806	5.644801	0.0612605	0.4490667
Recql4	0.3555098	4.376849	0.0612652	0.4490667
Mt2	0.4853822	5.8653643	0.0613459	0.449446
Herpud1	0.3708352	6.4863375	0.0614141	0.449734
Hsh2d	-0.4691336	1.8260779	0.0614673	0.4498574
Rnaseh2a	0.2636479	5.1676202	0.0614888	0.4498574
Aff3	0.5439196	5.0704979	0.0615917	0.4503983
Csgalnact2	-0.2683425	6.9588622	0.061805	0.4514695
Trim30a	-0.241185	5.8608865	0.0618426	0.4514695
Yrdc	0.3248517	4.1188191	0.0618612	0.4514695
Mthfd1l	0.2413832	5.8294725	0.0618689	0.4514695
Ugp2	-0.217494	6.1887382	0.0618833	0.4514695
Rps26	0.2684533	9.7882532	0.0619394	0.4515862
AW146154	0.4133848	2.3038907	0.0619574	0.4515862
Tmed3	-0.3007129	6.7514899	0.0620244	0.45173
Ncoa4	-0.2822601	7.620794	0.0620997	0.45173
Agpat5	-0.2105729	5.7307162	0.0621368	0.45173
Alkbh1	0.3347904	3.4678628	0.0621446	0.45173
Rab44	-0.4190863	6.7432504	0.0621507	0.45173
Ubtf	0.2249523	7.783216	0.0621514	0.45173
Fes	-0.2550866	7.113018	0.0622072	0.451925
E130307A14Rik	0.5020562	1.8420711	0.0622909	0.4521967
Gm8532	-0.9720315	-0.1134147	0.0623193	0.4521967
Cfp	-0.2942938	6.4099658	0.0623332	0.4521967
Vps16	0.2250322	5.5562636	0.0623609	0.4521967
Eif4e2	0.1971047	6.1675274	0.0624358	0.4523456
Plekho2	-0.261182	6.762636	0.0624396	0.4523456

Mblac1	0.681171	1.5232905	0.0625568	0.4529835
Ear2	-1.3284857	6.8336822	0.0626317	0.4532079
Lmbrd1	-0.2247658	5.5225418	0.0626461	0.4532079
Ehd2	0.4678263	4.7345307	0.0627118	0.4532675
Lamtor4	-0.3039049	6.0255951	0.0627126	0.4532675
Lifr	-0.5269753	7.5339114	0.0627599	0.4533194
Pgk1	-0.2644956	7.4231944	0.0627881	0.4533194
Gm21927	0.7167298	1.4288895	0.0628072	0.4533194
Ptpdc1	0.6084937	1.2333583	0.0628418	0.4533368
Hif3a	0.7943603	1.0642343	0.0628863	0.4533368
Stk10	-0.3346501	6.5875732	0.0628971	0.4533368
Nrp1	-0.197402	6.1950284	0.062945	0.4534722
Dnm3os	-0.7307895	3.8074445	0.0630034	0.4535191
Mbd5	-0.3846857	4.3199588	0.0630098	0.4535191
Selp	-0.4804002	5.1486901	0.0630604	0.4536735
Lrp4	-0.3941614	5.6437445	0.0631073	0.453795
Xylt1	-0.6226885	3.5837887	0.0631357	0.453795
Slc25a10	0.2375184	4.4913549	0.0632373	0.4541776
Ap3s1	-0.2836399	7.1761421	0.0632473	0.4541776
Gm8719	1.4851752	-0.6507373	0.0632954	0.4543133
Fam117b	-0.2920225	5.8853137	0.0634451	0.4551776
Eea1	-0.21979	5.9747002	0.0635051	0.4553763
Grb7	1.1390842	0.7678494	0.0635633	0.4553763
Eftud2	0.2667131	6.8294716	0.0635682	0.4553763
Exoc6	-0.2443567	6.7799007	0.0635899	0.4553763
Itgb3	-0.3021972	8.7674929	0.0636324	0.4554709
Osbpl2	-0.2461469	5.7082655	0.0636649	0.4554942
Mir17hg	0.3521652	4.726	0.0638158	0.4563638
Ccnd3	0.2349897	9.0469722	0.0638936	0.4564647
Dhrs11	0.3686048	7.1735682	0.0638954	0.4564647
Marchf6	-0.217873	7.0832098	0.063918	0.4564647
Zbed5	0.679602	0.1837668	0.0640182	0.4569447
Bzw2	0.2141541	5.5989143	0.0640439	0.4569447
Tedc2	0.3406091	4.2848188	0.0641317	0.4573607
P2ry1	-0.2601733	5.6153087	0.0641801	0.4574966
Ppp1r15a	0.415249	7.8951383	0.0642883	0.4578739
Gm15290	0.7457528	0.3824101	0.0643133	0.4578739
Rarres2	0.5786613	3.8203892	0.0643214	0.4578739
Cadm4	1.0811761	-0.7573028	0.0644507	0.4583549
Zfp956	0.4962944	2.8143823	0.0644961	0.4583549
Btf3-ps18	-0.9132626	-0.3466431	0.0644968	0.4583549
Snrpf	0.2504629	6.7764651	0.0645068	0.4583549
Cd300ld3	-1.2748381	-0.4699919	0.0646076	0.4585904

Sdhaf2	0.2123417	5.4400768	0.064655	0.4585904
2700099C18Rik	0.4275718	3.4963535	0.0646568	0.4585904
Xlr	-0.9023427	1.0717372	0.0646579	0.4585904
Idh2	0.2262129	6.0681193	0.0647039	0.4587076
Il6st	-0.2937891	6.595432	0.0649019	0.4599018
Map4k1	0.2583915	5.4939168	0.0649718	0.4601873
Cacna1b	1.0498188	-1.0958995	0.0651781	0.4614386
Ube2j2	0.2138082	5.6880964	0.0652198	0.4615237
Ptpa	0.244106	7.7218356	0.0653626	0.4623235
Pcbp2	0.231018	7.9660537	0.0654295	0.4625867
Rnf220	0.2118246	5.7493352	0.0655446	0.4631898
Asb11	-2.3728833	-1.429631	0.0655861	0.4632723
Csnk1e	-0.2404637	5.4045863	0.0657586	0.4642801
Colq	0.7704267	0.9219508	0.065842	0.464532
Sult1a1	0.5034947	3.7981167	0.0658763	0.464532
Gm21188	-0.4451646	3.4937118	0.0658839	0.464532
AU021092	-0.9835655	1.0492862	0.0659197	0.4645737
Cd74	0.3797736	8.7041813	0.0660492	0.4649535
Gm12816	0.9825322	-0.6405568	0.066069	0.4649535
Fbxo7	0.2868329	6.0004136	0.0660765	0.4649535
Kiz	-0.2697688	4.4003115	0.0661026	0.4649535
Ndnf	0.6360357	3.368561	0.0661352	0.4649535
Tuba1b	0.3242986	8.4212746	0.0661529	0.4649535
Fem1a	0.239622	5.4848457	0.066231	0.4651244
Mvb12b	-0.2606102	5.9527968	0.066237	0.4651244
Itga2b	-0.3171725	7.609525	0.0662787	0.465159
Bmpr1a	-0.3938077	4.652602	0.0664235	0.465159
Serinc4	0.8748437	-0.6986414	0.0664317	0.465159
Pctp	0.6479458	3.236171	0.0664884	0.465159
Mtfp1	0.4274245	3.213439	0.0664942	0.465159
Gart	0.2480122	6.2453289	0.0665079	0.465159
Gstp1	0.249832	5.0197242	0.066519	0.465159
Dhdh	-0.355989	3.3193828	0.066538	0.465159
E4f1	0.3372705	4.719476	0.0665508	0.465159
P2ry2	-0.5248484	2.2758241	0.0665585	0.465159
Cyp4b1-ps2	0.620312	2.4300258	0.0665709	0.465159
Cyp1b1	0.2965684	5.8578044	0.0666619	0.4654275
Bcl2a1b	-0.4720668	3.4772256	0.0666692	0.4654275
Stard4	-0.2990356	5.467435	0.0667455	0.4655229
Ddc	0.8840668	0.5729099	0.0667642	0.4655229
Nfe2l2	-0.2056223	7.0141633	0.0667726	0.4655229
Lsp1	-0.3216423	8.0085381	0.0668057	0.4655444
Abcf1	0.2049508	6.8999307	0.0669357	0.4660315

Lck	0.4399276	3.456626	0.0669771	0.4660315
5730488B01Rik	1.4082434	-1.1267426	0.0669869	0.4660315
Pdcd10	-0.2274018	6.0226719	0.0669954	0.4660315
Ksr1	-0.2871929	4.7212119	0.0670787	0.466402
Serpinb6a	-0.2657622	5.6964279	0.0671555	0.4667276
Pibf1	-0.3028326	3.7536638	0.0673199	0.467661
Selenbp1	0.2725917	5.1175649	0.0673879	0.4678988
Gm10138	0.4709104	1.7509478	0.067418	0.4678988
Snhg16	0.4529867	2.7793653	0.0674616	0.4678988
Grwd1	0.3437906	3.4315876	0.0674744	0.4678988
Eef1d	0.2650833	7.4567096	0.0675215	0.4679527
Hint2	0.3925964	3.0158891	0.0675424	0.4679527
Kdr	-0.2753373	5.5386386	0.0676967	0.4680556
Paqr4	0.4201916	3.3003503	0.0677369	0.4680556
Gm30603	-1.0726462	-0.3808459	0.0677423	0.4680556
Rnf144b	-0.2904537	5.3747926	0.0677854	0.4680556
Tlr13	-0.3135968	5.6146624	0.0678266	0.4680556
Igkv8-24	-0.6826121	3.130021	0.0678281	0.4680556
S100a6	-0.3610878	8.1015083	0.0678425	0.4680556
Pclaf	0.2171449	7.3369604	0.0678449	0.4680556
Magee2	1.2701296	-0.129476	0.0678468	0.4680556
Cept1	-0.2358919	5.8555389	0.0678719	0.4680556
Cd300lf	-0.285383	5.7923784	0.0678882	0.4680556
Exosc5	0.2761629	4.5934318	0.0679375	0.4681875
Galc	-0.2821067	4.8242907	0.0680229	0.4684188
Ung	0.2557192	4.1369151	0.0680737	0.4684188
Gna11	0.3047945	4.712054	0.0680921	0.4684188
Rpl8	0.251717	9.8120884	0.0681305	0.4684188
Angpt2	-0.5478695	2.7028632	0.068135	0.4684188
Cdca4	0.2335122	6.085984	0.0681517	0.4684188
Atpaf2	0.3004889	4.2221597	0.0681981	0.4685303
Slc25a53	0.5710058	2.3969561	0.0682667	0.4687947
Mrm1	0.3986533	4.4879908	0.0683764	0.4690689
Fam193b	0.2505244	5.6622447	0.0684528	0.4690689
Suv39h1	0.2397127	6.1415395	0.0684932	0.4690689
Nlgn2	0.3175382	3.9720574	0.0685134	0.4690689
Bmper	0.6828862	0.5660044	0.0685261	0.4690689
Vwf	-0.387715	6.9452201	0.0685825	0.4690689
Abhd17a	0.1837436	6.0283284	0.068588	0.4690689
Adgre5	-0.2848004	7.2205225	0.0686088	0.4690689
Notum	1.1227585	-0.3757655	0.0686179	0.4690689
Syap1	-0.2114392	5.7057993	0.0686334	0.4690689
Usp10	0.28935	5.60007	0.0686384	0.4690689

Dtwd2	0.3630179	2.8545501	0.0687556	0.4693489
Trerf1	-0.4886766	3.2202869	0.0687767	0.4693489
Gatad1	-0.2239268	6.1573939	0.0688037	0.4693489
Sptlc2	-0.2453796	6.4083574	0.0688287	0.4693489
Ubl4a	0.2818887	4.3574615	0.0689051	0.4693489
Bank1	0.3792285	4.5269955	0.0689307	0.4693489
Gm4489	-0.7630275	-0.8925424	0.068932	0.4693489
5430401H09Rik	0.4296177	5.2308733	0.0689481	0.4693489
Neurl1a	-0.5073537	1.8113616	0.0689509	0.4693489
Lrp8	-0.2815183	4.5936513	0.0689829	0.4693614
Pacrgl	0.372176	2.4610509	0.0690459	0.4695845
Slfn3	-0.434822	3.6703765	0.069191	0.4701778
Vrk3	0.2177747	5.2832443	0.0691936	0.4701778
Phkg1	-0.9311773	-0.5284137	0.0692486	0.4702027
Il22ra2	-0.7142813	0.1876748	0.0692577	0.4702027
Hp	-0.3120525	9.0858232	0.0693427	0.4705743
Fcna	-0.6077711	5.9876838	0.0694292	0.470924
Asb8	0.2593823	4.5030862	0.069459	0.470924
Stim1	-0.2512599	6.0547611	0.0694854	0.470924
Gm40652	-1.6062336	-0.7963226	0.0695153	0.470924
Ndufa4l2	0.8559938	0.8198837	0.0695581	0.4709914
Tnfrsf21	-0.2387622	6.2372351	0.0696313	0.4709914
Ubxn8	-0.2398436	4.8850131	0.0696464	0.4709914
Adgrg1	0.2519843	6.5286657	0.069687	0.4709914
Igsf5	-0.9238782	-0.4461111	0.0697058	0.4709914
Cntln	-0.2777474	4.8249633	0.069707	0.4709914
Slc12a5	-0.7886592	0.7022127	0.0697473	0.4710592
Trim30d	-0.493299	3.8771044	0.069819	0.4713391
5830417I10Rik	0.2790896	4.4043851	0.0698511	0.4713506
Ccdc34	0.2496781	5.8529099	0.0699591	0.4717567
Rab31	-0.2561637	6.3386872	0.0699719	0.4717567
Tbxas1	-0.3453202	5.2539252	0.0700358	0.471914
Wipi1	-0.2250256	5.4846175	0.0700916	0.471914
Zkscan17	0.3450124	4.371113	0.0701275	0.471914
R3hcc1	0.3130767	3.6490581	0.0701333	0.471914
Clasp2	0.2159258	5.7369315	0.0701469	0.471914
Insig1	-0.3826396	4.6841211	0.070283	0.4722768
Srgn	-0.3088811	9.0047932	0.0703093	0.4722768
Lrp6	-0.2203973	5.7534419	0.0703309	0.4722768
Lama3	-0.8502187	0.8110869	0.0703645	0.4722768
Ccs	0.2749609	4.3665511	0.0703798	0.4722768
Snhg15	0.4400175	1.9671246	0.070383	0.4722768
Jun	0.4610161	4.2390593	0.0704426	0.4724729

Tfr2	0.4030874	4.7869418	0.0706904	0.4739304
Psmb5	0.4100956	5.1851519	0.0707308	0.4739968
Igkv8-30	-0.5686629	4.2353207	0.0708441	0.474334
Pa2g4	0.2163134	7.8780492	0.0708837	0.474334
Sptan1	-0.2905088	7.3601468	0.0709185	0.474334
Tmem47	0.4413696	2.9749869	0.0709229	0.474334
Homez	-0.3568914	3.0273517	0.0709336	0.474334
Nat10	0.2687221	4.8468528	0.0710092	0.4746358
Kdm7a	-0.2432486	8.1622656	0.071085	0.4749384
Gm6789	0.4884097	1.5321696	0.071125	0.4749435
Mpg	0.3618693	3.7604112	0.0711469	0.4749435
Rnf150	-0.5194593	3.5402322	0.0714038	0.476142
Tamm41	0.2857797	3.7140798	0.0714144	0.476142
Gga2	0.2362007	5.5680907	0.0714182	0.476142
Gm15448	-0.5832099	1.4756811	0.0715429	0.4767686
Wdr90	0.2600136	4.5698895	0.0715905	0.4768815
Hps3	-0.2383237	5.3802578	0.0716704	0.477183
Ttc28	-0.3066566	4.321513	0.0716971	0.477183
Ibsp	-0.5777997	9.7433362	0.0717362	0.4771899
Hltf	-0.2301204	5.755432	0.0717881	0.4771899
Nop2	0.2858136	5.1100329	0.0718388	0.4771899
Mrps17	0.2658745	4.9075563	0.0718409	0.4771899
Tonsl	0.2647217	4.759758	0.0718515	0.4771899
Tacc3	0.242879	7.0121932	0.0719452	0.4776081
Dusp7	-0.3259645	4.5649769	0.0720296	0.4779641
Akap8	0.2312696	6.8671007	0.0721234	0.4782491
Ndufab1	0.2901499	5.7073859	0.072157	0.4782491
Nop9	0.2109301	5.0430999	0.0722053	0.4782491
Nrep	0.4758698	4.2936306	0.0722176	0.4782491
Fcer1g	-0.286476	7.8316629	0.0722542	0.4782491
Ppm1m	-0.2606634	5.7898418	0.0722742	0.4782491
Xndc1	0.3677087	3.5420542	0.0722967	0.4782491
Ccdc126	-0.3426757	3.4062659	0.0723185	0.4782491
Usp42	0.2472531	4.302317	0.0724176	0.4784562
Phtf1	0.2517636	4.3521111	0.0724396	0.4784562
P2ry12	-0.2927605	3.9618302	0.0724421	0.4784562
Gm15991	0.8983191	0.5138903	0.0724746	0.4784676
Ramp1	-0.2687306	5.391485	0.0725094	0.4784942
Bin2	-0.2965343	7.352842	0.0725474	0.4785419
Wdr86	0.5328293	2.2828601	0.0726829	0.4792328
Il27ra	0.5987593	2.3485015	0.0727853	0.4797048
Nudt19	0.3324954	5.4080438	0.0732858	0.482344
Nkd1	0.8115501	0.6585547	0.0733395	0.482344

Ovgp1	-0.9328074	2.8163555	0.0733858	0.482344
Nrip2	0.6583755	0.6505051	0.073386	0.482344
Prmt5	0.2391258	5.3366922	0.073389	0.482344
Tesk2	-0.3587184	4.0758073	0.0733892	0.482344
Zfp184	0.7832811	0.0578552	0.0734333	0.482344
Coq6	0.3867608	3.0969609	0.0734574	0.482344
Taf7	0.2350955	4.6240319	0.0734649	0.482344
Kat14	0.2780581	5.0799667	0.0736246	0.4830017
Upf3a	0.2240947	5.248308	0.0736272	0.4830017
Kcnk13	0.6293609	0.8571471	0.0736582	0.4830017
Prodh	-0.3288803	3.5625955	0.0737253	0.483129
Sdhaf1	0.3756944	3.263769	0.0737459	0.483129
Ndufs3	0.2496801	5.1512293	0.0737784	0.483129
Prkrip1	0.2777871	4.1863027	0.0738323	0.483129
H2ac11	0.7397077	-0.0180169	0.073841	0.483129
Tdrd7	-0.2266595	4.6052352	0.073864	0.483129
Jph1	-0.9221679	1.2511319	0.0739361	0.4832258
Utp18	0.2207959	5.3258709	0.073941	0.4832258
Ino80d	-0.2806375	6.42571	0.073975	0.4832451
Minpp1	0.2872362	7.0745717	0.0740414	0.4833269
Fam3c	0.2393943	5.2968228	0.0740496	0.4833269
Rc3h2	-0.2054226	5.9728455	0.0742276	0.4842852
Kcnj10	-0.4314606	4.3104811	0.0742947	0.4844158
Zfyve1	-0.2300029	4.7530304	0.0743099	0.4844158
Aldh3a2	-0.2458219	5.5529055	0.07437	0.4846043
Eef1b2	0.3081008	8.7367854	0.0744596	0.4849854
Nek2	0.2500762	6.157288	0.0745138	0.4851353
Pgbd5	0.6874227	1.8026848	0.0745818	0.4853744
Sytl1	-0.4972222	2.5968247	0.0746821	0.485673
Eef1aknmt	0.3202961	3.2499443	0.0746901	0.485673
Snrnp70	0.2454799	8.3271031	0.0747419	0.4857198
Slamf9	0.5554479	1.7918711	0.0747686	0.4857198
Coro2a	0.2852643	4.7882853	0.074791	0.4857198
Rwdd2a	-0.7681469	0.6563905	0.074882	0.4859504
Lonrf3	-0.3641407	3.8486307	0.074889	0.4859504
Efcab5	-0.4982427	1.5505387	0.0750912	0.4870119
Pcyox1l	0.3068885	3.8934559	0.0751152	0.4870119
Akap1	0.2814802	3.8841619	0.0751907	0.4872985
Gm52709	-0.7020692	1.9820613	0.0752408	0.4873734
Nol10	0.2651244	4.4805187	0.0752649	0.4873734
Sema6c	-0.6954835	-0.4242506	0.0753289	0.4873816
Gm4875	-1.9099803	2.3323797	0.0753289	0.4873816
Cbx1	0.1947361	6.705109	0.0753837	0.4875335

Gm33682	-0.5373356	1.2278981	0.0754966	0.4880471
Nrxn1	1.201233	0.553979	0.0755369	0.4880471
Zfp931	-0.4100934	2.4617203	0.0755573	0.4880471
Ppt2	-0.2854207	4.7928035	0.075641	0.4883854
Rps19	0.282614	8.746386	0.0756788	0.4884263
Ahnak2	-0.879474	0.2108511	0.0757408	0.4886237
Eif2b5	0.216467	5.4617554	0.0759049	0.4890624
Gm46558	-0.8627178	-0.0316689	0.0759064	0.4890624
Dpy19l4	-0.2703069	5.2136463	0.0759408	0.4890624
Rassf3	-0.2670814	7.0885735	0.0759443	0.4890624
Pkm	-0.2672856	9.5479118	0.075966	0.4890624
Grhpr	0.4076177	3.2670121	0.0760047	0.4891092
Nfe2l1	-0.1997266	6.7450021	0.0760899	0.4892561
Fkbp1a	0.2265656	6.2681337	0.0761606	0.4892561
Zbtb16	-0.4313416	3.2689556	0.0761656	0.4892561
Sdc4	0.2970967	5.15211	0.0761702	0.4892561
Bnip1	0.29891	3.5336204	0.0761886	0.4892561
Zfp689	0.5411566	2.1198289	0.0762162	0.4892561
Ccnd2	0.2367768	6.0892724	0.0762909	0.4895331
Crtc2	0.2654841	5.1374305	0.076419	0.4896331
Trim11	0.2549931	5.601761	0.076436	0.4896331
Sf3b5	0.2480037	5.6840422	0.07644	0.4896331
Prxl2c	-0.2383822	4.9375449	0.0764517	0.4896331
Bend3	0.3471962	3.3093985	0.0764798	0.4896331
Strn	-0.2524239	6.3679365	0.0765601	0.4896331
Myl10	-1.0462311	0.1934466	0.0765606	0.4896331
Eif4a3	0.1884748	6.4001325	0.0765728	0.4896331
Ppargc1a	0.7692116	-0.7350967	0.0765898	0.4896331
Tmem222	0.2531836	4.3976459	0.0767232	0.4902845
Itga6	-0.3371131	7.124452	0.0768143	0.49045
Dact3	0.6044311	1.4316413	0.0768696	0.49045
Gm13611	0.8573357	-0.5289086	0.0769376	0.49045
Phex	-0.7279868	7.1826748	0.076945	0.49045
Nceh1	-0.2900222	5.1983011	0.076994	0.49045
H2ac15	0.9725347	-0.3829658	0.0770211	0.49045
Palm	-0.3028011	5.5714185	0.0770342	0.49045
Tob1	-0.2957279	4.9301161	0.0770795	0.49045
Mrip-ps	0.8397134	-0.4023505	0.0771096	0.49045
Msc	0.9618482	-0.0070176	0.077142	0.49045
Isyna1	0.2245939	5.0905787	0.0771464	0.49045
Ces1d	-0.8338762	2.9484416	0.0771532	0.49045
Lrpprc	0.2030808	6.1074414	0.077159	0.49045
Gcc2	-0.2947725	5.3309064	0.0772012	0.4905179

Fzd6	0.5778885	1.3926718	0.0772751	0.4907865
Plxna4	-0.3316453	4.3545541	0.077348	0.491049
Cdk4	0.1959527	7.0600071	0.0774352	0.4914021
Gm15860	0.7981931	1.4377718	0.0775071	0.4915836
Atxn7l1	-0.1918272	6.2757439	0.0775463	0.4915836
Gm11827	-0.8342848	-0.172846	0.0776159	0.4915836
Elac1	-0.3097599	3.7326051	0.077659	0.4915836
Gm32528	-0.7578694	0.5092628	0.0776718	0.4915836
Ankrd66	1.0615678	-0.7697792	0.0776974	0.4915836
Gm8129	0.8686233	0.2759976	0.0777059	0.4915836
Fut7	-0.4228054	2.5445243	0.0777464	0.4915836
Nudt9	0.3155246	5.8261617	0.0777498	0.4915836
Trit1	0.3290711	3.2002695	0.0777798	0.4915836
Gm20161	0.434982	4.5195301	0.0778615	0.4918997
Cygb	0.7694133	2.6529172	0.0779133	0.4920271
Rhobtb3	-0.3746651	3.4407004	0.078187	0.4930606
Btbd7	-0.1946944	5.8012892	0.0781924	0.4930606
Glce	-0.2781686	4.0235854	0.0782122	0.4930606
Ddx27	0.2141186	5.8909177	0.0782133	0.4930606
Tubb4b	0.2679898	8.866522	0.0782354	0.4930606
Wdr81	0.2402638	6.1610526	0.0783238	0.4933833
Pfas	0.2949437	5.4957034	0.0783501	0.4933833
Ncam1	-0.2583968	5.1811294	0.0784529	0.4938309
Cisd1	0.3470807	4.4986142	0.0785467	0.4941914
Gm4366	0.3699099	2.7672871	0.0786032	0.4941914
Socs4	-0.2211109	6.677272	0.0786055	0.4941914
4930431P19Rik	0.8134605	-0.6576093	0.0786391	0.494203
Zxdc	-0.2700779	4.1299198	0.0786715	0.4942072
Rpp40	0.4954406	1.793254	0.0787861	0.4945311
Gxylt1	-0.2433001	5.9485976	0.0788164	0.4945311
Cdc20	0.2572899	6.7265113	0.0788457	0.4945311
Unc93b1	0.2207305	7.4798919	0.0788503	0.4945311
Rab3gap2	-0.2117058	5.9910145	0.0789448	0.4949245
Rps2-ps10	1.0251061	0.6162519	0.0790074	0.4950204
Gvin3	-0.5183109	6.6602087	0.0790238	0.4950204
Bcdin3d	0.5283349	1.890495	0.0790587	0.4950401
Zfpm2	-0.7927116	-0.6017069	0.0791263	0.495101
Rnf187	0.196173	6.873958	0.0791321	0.495101
Wdr1	-0.2488955	8.2409889	0.0791711	0.4951456
Nudt21	0.2429222	5.9194657	0.0792619	0.4955145
Ccndbp1	0.2838834	6.5619989	0.0794179	0.4959796
Sult4a1	-0.9405296	-0.2079814	0.0794345	0.4959796
Ccn6	-0.7917562	-0.6295172	0.0794347	0.4959796

Ercc2	0.3337464	4.0200362	0.0794639	0.4959796
Tiam1	-0.3270245	5.9802921	0.0795526	0.496096
Hspb11	-0.352495	2.482812	0.0795784	0.496096
Gja5	0.5651072	1.5517424	0.0795965	0.496096
Dus4l	0.4949328	2.370034	0.0796514	0.496096
Rassf9	-1.3421127	-1.1017219	0.0797078	0.496096
Tlr8	-0.3372007	5.0828797	0.0797101	0.496096
Mthfs	-0.4877119	3.3717942	0.0797136	0.496096
Ppm1j	-0.9965461	-0.0438856	0.0797377	0.496096
Serpina3i	-0.9158258	-0.8218015	0.0798487	0.4963224
Ptpn22	-0.2690881	4.7830074	0.0798729	0.4963224
Hmgcs2	-0.9771468	1.5683922	0.0799217	0.4963224
Slamf1	-0.3883838	4.3938994	0.0799314	0.4963224
Scd1	0.8044904	7.8882786	0.0799595	0.4963224
Slc4a8	-0.5388358	0.9142713	0.0799655	0.4963224
Plac8	-0.3113183	8.0984297	0.0800013	0.4963465
Smtnl2	0.535629	1.8770763	0.0800514	0.4964592
Rrp12	0.3255526	4.3100713	0.0801337	0.4965759
St8sia6	-0.5048493	2.8207079	0.080146	0.4965759
Wdr74	0.2700623	4.5263894	0.0801995	0.4965759
Mrps34	0.278265	4.642372	0.0802179	0.4965759
Pxn	-0.2861988	7.2908456	0.080235	0.4965759
B3galt2	-0.6197479	1.7486353	0.0802618	0.4965759
Cdk14	-0.452669	3.2646885	0.080322	0.4967506
Snx14	-0.2372031	4.7229494	0.0803776	0.4968972
Ctsd	-0.2147963	9.2622744	0.0804496	0.4969888
Dhx30	0.2117615	5.2129545	0.0804654	0.4969888
Eef2kmt	0.3287175	3.0352342	0.0805026	0.4969888
Mettl24	1.0577442	-0.4754522	0.0805364	0.4969888
Dock7	-0.253367	5.1061794	0.0806671	0.4969888
Gm32798	0.7784449	1.4595426	0.0806877	0.4969888
Ifi209	0.3364419	6.5766126	0.0807196	0.4969888
Mob1a	-0.2522307	7.8648241	0.0807342	0.4969888
Rab1b	-0.2520571	6.5862694	0.0807424	0.4969888
Lct	-0.6025595	0.8124855	0.0807514	0.4969888
Msmo1	-0.3839232	4.8323038	0.0807923	0.4969888
Atp1b3	0.2458813	7.6219583	0.0808039	0.4969888
Hmgn2-ps1	-0.4612114	2.8705485	0.0808078	0.4969888
Eid2b	0.3508038	2.657145	0.0809769	0.4978321
Krt10	-0.6806963	0.5377018	0.0810553	0.4981168
LOC115490200	0.3466764	3.0430134	0.0812345	0.4990209
Cacna1g	0.40417	3.8243138	0.0813689	0.4996495
Pomgnt1	0.4021537	3.2623671	0.0815172	0.5002698

Rbm10	0.23006	6.188828	0.0816245	0.5002698
Rps3a2	0.3936589	3.2262458	0.0816304	0.5002698
Tenm4	-0.5153311	3.3581514	0.0816415	0.5002698
Stat1	-0.2142853	6.7709377	0.0816476	0.5002698
Kitl	0.250634	6.258755	0.0816629	0.5002698
4931428F04Rik	0.5154168	1.7732665	0.0817011	0.5003065
Hpgds	-0.3542531	3.8490032	0.0817632	0.5004898
Bach2	0.7516287	6.2123421	0.0818413	0.5006911
Idh1	-0.2260354	6.3336127	0.0818813	0.5006911
Bmpr2	-0.2250427	6.1795944	0.0818926	0.5006911
9530077C05Rik	0.3908571	2.6273413	0.0820071	0.5011006
Fam222b	-0.2476735	4.8508449	0.0820241	0.5011006
Cpm	0.8929276	4.274582	0.0820657	0.5011582
Osgin1	-0.3828215	3.2550655	0.0823616	0.5026585
Tln1	-0.3230068	9.8774662	0.082376	0.5026585
Casp8ap2	-0.2347921	6.4141271	0.0824486	0.5027368
Tut1	0.2326979	4.3124362	0.0824535	0.5027368
Slc40a1	-0.2319601	7.8710277	0.0825942	0.5032894
Dnajc13	-0.2842067	6.8303893	0.0826088	0.5032894
Mettl3	0.2236858	4.5627985	0.0827703	0.5038833
D630044L22Rik	0.7366232	0.6563738	0.0827975	0.5038833
Diras2	-0.5003452	4.0078086	0.0828124	0.5038833
Ndufs7	0.2706065	5.4293961	0.0828609	0.5038833
Nhsl1	-0.5123336	3.869578	0.0828698	0.5038833
Klf8	-0.4116207	2.3257594	0.0829095	0.5038833
Slc11a2	0.2458626	5.8481167	0.0829331	0.5038833
Ikbke	-0.3122263	4.9050095	0.0829877	0.5040184
Tmem109	0.2824609	4.5936565	0.083169	0.5048077
Lancl2	0.2635047	5.3218726	0.0831826	0.5048077
Creb5	-0.6131694	0.1767315	0.0832728	0.5051581
Srsf10	0.2226521	7.7966754	0.0833813	0.5052349
Kif21b	-0.3453384	6.7425801	0.0833971	0.5052349
Mctp2	-0.4068564	4.4145923	0.083424	0.5052349
Dnmt3a	-0.263609	6.5604572	0.0834707	0.5052349
Tbrg4	0.2115422	5.5622683	0.0834916	0.5052349
Slc7a5	0.3503935	6.6628691	0.0835098	0.5052349
Igkv9-124	-0.7322339	2.7480674	0.0835325	0.5052349
Zfas1	0.3354443	5.0718087	0.0835673	0.5052349
Adtrp	-0.5637459	1.8983644	0.0835778	0.5052349
Klf6	-0.2810852	6.9767498	0.0836134	0.5052539
Gcsam	-0.3807701	4.2735189	0.0836915	0.5054655
Sars2	0.3271295	3.396519	0.0837352	0.5054655
Tmsb4x	-0.2983092	12.031929	0.0837729	0.5054655

Fahd1	0.3891076	5.3102343	0.0838398	0.5054655
Cracd	-0.3004157	6.3278717	0.0838904	0.5054655
Hspa1a	-1.3645254	-0.1531743	0.0839174	0.5054655
Arl6ip4	0.1950266	5.5912874	0.0839262	0.5054655
Mbnl3	-0.2333211	5.906646	0.0839464	0.5054655
Csrnp1	0.3947705	3.7665296	0.0839919	0.5054655
Zfp281	-0.242892	5.4280183	0.0840024	0.5054655
Hsd17b11	-0.2608509	6.1373524	0.0840059	0.5054655
Eif3j2	0.2531303	5.9958529	0.0840631	0.5054955
Mustn1	-0.7161492	1.4205123	0.0840759	0.5054955
2310033P09Rik	0.3347581	4.4197861	0.0841235	0.5055382
Gm2666	0.7181375	1.1949932	0.084148	0.5055382
Fmn1	-0.5485436	2.7684597	0.0842264	0.5058138
Timm9	0.3641389	3.2004273	0.0842696	0.5058285
Tbkbp1	-0.2446269	5.2537065	0.0842939	0.5058285
Gm26908	-0.9166343	-0.6445205	0.0843464	0.5058601
Spry1	0.3783997	4.1244591	0.0843733	0.5058601
Edaradd	0.713243	2.473853	0.0843968	0.5058601
Hbq1b	0.5321645	4.3742628	0.0844736	0.5061259
Naip3-ps1	-0.9016193	-0.0589645	0.0845578	0.5063434
Cap1	-0.2810194	9.1864319	0.084575	0.5063434
Oaz1	0.2337273	8.5503167	0.0846589	0.5066504
Gm30881	-0.8517538	-0.8996445	0.0847018	0.5067121
Sh2d1a	-0.8481881	-0.5805967	0.0848127	0.5070722
Myef2	0.279598	6.2188731	0.0848535	0.5070722
Mtch2	0.2437106	6.250347	0.0848598	0.5070722
Cdon	-0.4875129	2.0804192	0.085	0.507663
Ccr7	0.6278869	2.5011995	0.0850298	0.507663
Chst2	0.2953324	3.772585	0.0850566	0.507663
Fam229b	1.1403736	-0.7210185	0.0850914	0.5076763
Runx1	0.2242589	6.4756804	0.085183	0.5080279
Zfp446	0.420341	2.1306426	0.0852792	0.5084067
Atad3a	0.2233083	4.9094101	0.0853269	0.5084758
Cdc14a	-0.2410245	5.0034607	0.0853627	0.5084758
Rasa2	-0.2619831	5.5030359	0.0853889	0.5084758
Gm15501	0.4201486	2.8096132	0.0854315	0.5085347
Gm6525	1.3192921	0.3620661	0.0855403	0.5089878
Rex1bd	0.2911624	4.8130995	0.0856496	0.5093007
Mapk1ip1	0.3996989	3.1267498	0.0856584	0.5093007
Utp6	0.182254	5.748037	0.0857127	0.5094288
Gm41464	0.621252	0.3582471	0.0857688	0.5095674
Elapor2	-0.4449754	1.4995393	0.0859516	0.5104586
Phyhd1	0.4515308	2.4754362	0.0860178	0.5105795

Stam2	-0.2463058	4.676968	0.0860376	0.5105795
Nhlrc3	-0.4708103	2.3966644	0.0863024	0.5118208
Zfp1	0.3951493	3.0169129	0.0863126	0.5118208
Cln3	-0.2381569	4.9406664	0.0863802	0.5119377
Mtss1	0.2342904	6.4896246	0.0863981	0.5119377
Eif3g	0.1854655	6.19077	0.0865535	0.5126628
Gata1	0.2880426	6.7485567	0.0866158	0.5127867
Lonrf2	0.5047196	1.5255012	0.0866529	0.5127867
Stk39	-0.4348631	3.4777628	0.0866733	0.5127867
Fst	0.6848343	2.8438816	0.0867869	0.5128575
Ldhb	-0.473193	3.9510507	0.0867877	0.5128575
Cpeb2	-0.2144883	4.9468716	0.0868515	0.5128575
Smim1	0.297265	5.2238933	0.0868533	0.5128575
Ypel3	0.2305921	6.6678778	0.0868634	0.5128575
9530068E07Rik	-0.2050596	6.9987664	0.0868831	0.5128575
Tm4sf19	-0.5833352	2.7376609	0.0870536	0.5130948
Triqk	-0.6514099	1.0312904	0.0870543	0.5130948
Pgap1	-0.2964099	3.8106396	0.0870576	0.5130948
Iqgap1	-0.3301978	8.7185873	0.0871101	0.5130948
Ybx3	0.2343967	8.0002146	0.0871271	0.5130948
Mrps6	0.4052516	3.4228249	0.0871319	0.5130948
Rfng	0.3085024	3.91635	0.0871814	0.5130948
Adamtsl4	0.5642745	2.0008203	0.0871872	0.5130948
Lrrc45	0.2696555	4.2829061	0.0872591	0.5133237
Tmie	-0.8260433	1.4739661	0.0873463	0.5136423
Crot	-0.2445304	5.461232	0.0873936	0.5137262
Dis3l	0.2413875	4.3093501	0.0874633	0.513942
Nr1d2	0.5752858	5.2219032	0.0875655	0.5143479
Mrps18a	0.2700928	4.2978162	0.0876451	0.5146213
Rps15-ps3	0.7581151	0.0661009	0.0876824	0.5146462
Oma1	-0.2590136	3.8193313	0.0878185	0.5151784
Spsb1	0.4772554	3.2954557	0.0878423	0.5151784
Pde8a	-0.2730538	4.7035626	0.0878764	0.5151784
Ube2d-ps	0.5272599	2.1278321	0.0879706	0.5151784
Tmem63a	-0.22058	5.7657019	0.0879718	0.5151784
Dixdc1	-0.4463518	1.6641716	0.0879718	0.5151784
Dsc2	0.9809465	2.5518731	0.0880629	0.5155174
Zfp407	-0.2740429	4.6271606	0.0881351	0.5156101
Ano10	-0.2936202	5.1298507	0.0881607	0.5156101
Inpp5b	-0.1897835	6.1146695	0.0881781	0.5156101
Gm16174	-0.7642711	0.1212082	0.0883366	0.516225
Tom1	-0.3242432	4.3547483	0.0883497	0.516225
Fras1	0.7875454	0.856774	0.0883995	0.5162879

Plscr2	-0.6439241	1.0206348	0.0884479	0.5162879
Srsf6	0.2090549	8.0380072	0.08846	0.5162879
Gm16378	-0.6907729	-0.0102297	0.0885281	0.5164914
Pkd1l3	-0.5298886	2.7108632	0.0885641	0.5165077
Ccl25	0.6128518	1.0024362	0.0887069	0.5169209
Kmt2c	-0.2391423	7.0436925	0.0887258	0.5169209
Ythdf1	0.1866411	6.0432688	0.0888299	0.5169209
Atrx	-0.2390802	7.9216471	0.0888399	0.5169209
Rnf128	-0.4286213	2.0112794	0.0888471	0.5169209
Fbl	0.179591	6.775135	0.0888499	0.5169209
Zdhhc20	-0.2306445	6.1430826	0.0889038	0.5169209
LOC115487771	-1.1281722	-0.9556302	0.088926	0.5169209
Abcg3	-0.3441094	4.1411841	0.0889341	0.5169209
Zfhx3	-0.2801907	4.0893255	0.0890507	0.5173193
Fads3	0.2726056	4.6589634	0.0890691	0.5173193
Fcrlb	1.055353	-0.7446504	0.0891815	0.5176554
6030458C11Rik	0.2449029	5.6915344	0.089229	0.5176554
Id3	0.5789701	5.9769126	0.0893097	0.5176554
Smarcd2	0.1922236	6.9524876	0.0893114	0.5176554
Znrf2	-0.219956	5.2090175	0.0893262	0.5176554
Hoxb4	-0.3755304	3.0422357	0.0893267	0.5176554
Pik3r6	-0.3655906	4.1058894	0.0894438	0.5176758
Rab29	-0.2486973	4.1514427	0.0894755	0.5176758
Jchain	-0.5090447	9.4073584	0.0894759	0.5176758
Casz1	-0.4261435	3.0100401	0.0894885	0.5176758
Polr3gl	0.260968	4.6199227	0.0895098	0.5176758
Gm16675	-0.5051119	0.9605307	0.0895599	0.5176758
Dst	-0.301399	5.5530145	0.0896032	0.5176758
Srrt	0.223988	7.6271899	0.0896276	0.5176758
Ptgfrn	0.4467099	4.6995876	0.0896297	0.5176758
Zcchc9	0.2371984	4.2789726	0.0897006	0.5177714
Rabif	-0.2089139	4.8308365	0.089747	0.5177714
Prss53	0.8649186	-0.0782668	0.0897695	0.5177714
Pfkp	-0.2459231	5.5089036	0.0897794	0.5177714
Lonp2	-0.2086545	5.7505824	0.0898664	0.5180808
Igsf11	-1.2218833	-0.6827486	0.0899219	0.5182086
Ccdc24	0.7500882	-0.4439095	0.090063	0.5188298
G2e3	-0.2199766	6.108115	0.0901066	0.5188583
Ccdc92b	0.9091533	3.7569123	0.0901834	0.5188583
Med10	0.2334409	4.8472006	0.0901891	0.5188583
Lrrn4cl	0.843901	-0.7996579	0.090231	0.5188583
Slk	-0.2131778	8.0499793	0.0902497	0.5188583
Arf6	0.1999961	8.1219737	0.0903328	0.5188583

Gstp3	0.5482613	2.2322139	0.0903341	0.5188583
Arap3	-0.2211489	6.4731075	0.0903419	0.5188583
Bricd5	0.80258	-0.9861124	0.0904153	0.5188583
Fli1	-0.2503182	6.541309	0.0904506	0.5188583
Zcchc10	0.2901775	3.4663419	0.090456	0.5188583
Zdhhc14	0.2945817	4.6644366	0.0904683	0.5188583
Nfatc2ip	0.2215034	4.9140016	0.0905661	0.5192277
Atp13a2	0.2370642	5.9370268	0.0906894	0.519743
Pon2	0.1910969	6.2828074	0.0907327	0.5197995
Gimap7	-0.5071045	1.4612501	0.0907698	0.5198206
Ap1g1	-0.2007989	6.7559479	0.0908069	0.5198421
Tbx2	0.5881778	3.8787351	0.0910226	0.5207038
Fcho2	-0.1945828	6.8915151	0.0910369	0.5207038
Mepe	-1.0835794	5.4554763	0.0910579	0.5207038
Klf16	0.2631506	4.8896524	0.0912236	0.5213882
Eml2	-0.2317815	5.0363626	0.0912505	0.5213882
Zfp317	0.244401	4.6077054	0.0912781	0.5213882
Clec4b1	-0.663962	0.9643543	0.0913413	0.5215577
Fut8	-0.2242148	5.8816107	0.091441	0.5219349
Cenph	0.3088438	5.6526616	0.0914754	0.5219399
Bloc1s2	0.2596882	5.087051	0.091525	0.5220316
LOC102639428	0.8818868	-0.3359223	0.09157	0.5220713
Rnf149	-0.1843592	6.1952018	0.0915991	0.5220713
Ggct	0.4495777	2.3275992	0.091673	0.5223008
Hnrnpm	0.1932702	8.7815271	0.0917424	0.5224489
AY036118	0.5183076	1.7976892	0.0917661	0.5224489
LOC118568325	-0.6214448	0.4597996	0.0919154	0.5231072
Naip2	-0.3728358	5.2042793	0.0920356	0.523405
Parp8	-0.2912746	5.3930196	0.0920407	0.523405
Zfp276	-0.2315575	5.0911658	0.0920687	0.523405
Dnajc3	-0.195071	7.1323129	0.0921064	0.5234283
Rbm33	-0.1993988	6.8007341	0.0924423	0.5248003
Rhoj	-0.2495391	4.1065099	0.092448	0.5248003
Eml5	-0.4849158	2.9217145	0.0924491	0.5248003
Rpl9-ps4	0.5930515	0.0843724	0.0925219	0.5248752
Pisd-ps3	0.2556139	5.8765718	0.0925526	0.5248752
Rnf126	0.2104369	5.5358341	0.0925635	0.5248752
Smtn	-0.2537194	5.6300263	0.0926071	0.5249131
Tyw5	0.3961293	3.1221668	0.0926377	0.5249131
Zscan29	-0.2132443	5.7548203	0.092673	0.524922
Vipr2	-0.7479303	-0.0084132	0.0927342	0.5250354
Nde1	0.2227593	6.5881747	0.0927605	0.5250354
Xiap	-0.2365622	7.0963396	0.0928562	0.5252881

Ypel4	0.3928924	6.9392709	0.0928727	0.5252881
Gdap10	-0.5492622	0.6097621	0.0929586	0.5254048
Poc5	0.3004753	4.3605451	0.0929609	0.5254048
Septin4	0.3218178	3.579362	0.0930844	0.5258192
Map2k2	0.1935591	6.2184598	0.0931018	0.5258192
Aif1	0.3630137	4.2918522	0.0932397	0.5264064
Exoc6b	-0.3102575	3.728884	0.0933511	0.5268444
Mtmr6	-0.207359	6.6476407	0.0935763	0.5276637
Gm29718	0.6175777	0.9785987	0.0935854	0.5276637
Pgam1	-0.3165519	6.1802688	0.0935981	0.5276637
Kti12	0.2587679	4.6203043	0.0937149	0.5281307
Smpd4	0.2358091	5.7317396	0.0937717	0.5281307
Clcn6	0.3543796	3.3149648	0.0937828	0.5281307
Pink1	-0.4340855	6.3515769	0.0938187	0.5281421
Gm42151	0.7066395	1.2927181	0.0938835	0.5283153
Tsen15	0.2942375	3.5822396	0.0939642	0.5285782
Selenow	0.2679851	6.2563731	0.0941019	0.5289787
Angptl4	-0.4732955	4.6292095	0.0941423	0.5289787
Sirpb1a	-0.8094975	0.4650438	0.0941928	0.5289787
Gm32164	0.6026863	4.2294261	0.0942063	0.5289787
Tnfsf13	-0.5597311	1.6399076	0.0942678	0.5289787
Ttc39aos1	0.7291356	2.8886321	0.0942987	0.5289787
Srgap2	-0.2147401	6.0826355	0.0943202	0.5289787
Gm5518	0.5053148	2.3084307	0.0943404	0.5289787
Trim39	0.266553	4.9544855	0.0943545	0.5289787
Capsl	1.1467967	0.9364897	0.0944031	0.5289787
Rspo3	0.540505	2.9159815	0.094438	0.5289787
Gk5	-0.4519764	3.3821867	0.0944509	0.5289787
Tab1	0.2955101	4.685948	0.0945039	0.5289787
Arsg	-0.3214978	3.7928999	0.0945433	0.5289787
Il18bp	-0.2519447	3.7595216	0.0945733	0.5289787
Ptdss2	0.3162397	6.4823532	0.0946224	0.5289787
Tomm40l	0.243248	4.1457233	0.0946396	0.5289787
Mgat5	-0.3808663	5.1846264	0.0946475	0.5289787
Wwc1	-1.0709573	-0.5019389	0.0946973	0.5290666
Mrpl52	0.2883877	5.3440472	0.094785	0.5292026
Gm28530	-0.7512436	-0.7831133	0.0948032	0.5292026
Sinhcaf	0.2559064	4.1717165	0.0948237	0.5292026
Nop53	0.1744339	7.2806453	0.0949562	0.5295353
Impa2	-0.2688495	5.2193168	0.0949987	0.5295353
Tmem30a	-0.1990199	7.4053106	0.0950062	0.5295353
Fat3	-0.6136945	4.7387919	0.0950759	0.5295353
Tra2a	0.2616749	7.4064063	0.0950859	0.5295353

Fdxr	0.42124	2.7360831	0.0950989	0.5295353
Crtc3	-0.2649589	4.4597053	0.0951216	0.5295353
Mul1	-0.250961	3.801694	0.0952917	0.530103
Sytl2	-0.4725027	3.0841295	0.095359	0.530103
Bcl2l12	0.3419022	3.5387914	0.0953665	0.530103
Kcnab3	0.7871227	-0.4072505	0.0954118	0.530103
Eid3	0.9572433	-0.9570132	0.095469	0.530103
Znrd2	0.2970541	3.3556334	0.095574	0.530103
Gm27252	0.9702308	-0.2055089	0.0956363	0.530103
Rps21	0.3156019	9.093688	0.0956408	0.530103
Pycr2	0.252289	5.2783006	0.0957284	0.530103
Heca	-0.1955019	6.086872	0.0957743	0.530103
Ddx58	-0.2270633	5.1563777	0.0958803	0.530103
Tmem33	-0.2630372	6.7623733	0.0959152	0.530103
Pip4p1	0.2339741	4.7765949	0.0959156	0.530103
Gm32819	0.6830968	2.6700453	0.0959372	0.530103
Spring1	-0.3825608	2.2720534	0.0959444	0.530103
Tomm6	0.1856878	6.4260558	0.0960609	0.530103
Arap1	-0.2223488	6.8089075	0.0960682	0.530103
Rev3l	-0.2277975	5.8798899	0.0960842	0.530103
Fcmr	0.5581273	4.7495655	0.0960849	0.530103
Ap5m1	-0.25995	5.2551075	0.096121	0.530103
Apobec3	0.2099944	5.1022223	0.0961508	0.530103
Osbpl8	-0.1856398	7.7390458	0.0961834	0.530103
Zdhhc12	0.3445206	2.8112906	0.0961909	0.530103
Zfp444	0.3693193	3.0847141	0.0962104	0.530103
Shb	0.3402954	2.4384722	0.0962128	0.530103
H2-M3	0.3560201	3.1537667	0.0962435	0.530103
Arid4a	-0.2065045	6.6668203	0.0962554	0.530103
Heg1	-0.2509386	5.6404562	0.0962647	0.530103
Tmem234	0.211497	6.6868749	0.0963128	0.530103
Zfp182	-0.2711723	4.0024824	0.0963191	0.530103
Parp3	-0.3336836	3.652989	0.0963643	0.530103
Itprid2	-0.2265928	5.2265801	0.0964152	0.530103
Igkv4-53	-0.832259	2.1794321	0.0964415	0.530103
Gm36595	1.1731472	-0.2176145	0.0964434	0.530103
Tal2	-0.9439503	-1.0861544	0.0964499	0.530103
Hmgb1-ps7	0.586211	1.5494119	0.0964758	0.530103
Bhlhe40	-0.2582499	4.9782227	0.0964846	0.530103
Rhobtb1	-0.402259	3.314625	0.0965542	0.530167
Eno1	-0.2362694	8.2802151	0.0965644	0.530167
Alas1	-0.2897889	6.8321963	0.0966562	0.5303051
Rbm45	0.2022043	4.8822278	0.0966578	0.5303051

Scn4b	0.8152267	1.334395	0.0967259	0.5303176
Hdhd5	0.3543885	3.1938998	0.0967283	0.5303176
Dcaf15	0.2844264	4.628485	0.0968708	0.5309119
Gpn1	0.2796559	3.563585	0.096948	0.5309858
Tomm22	0.2193135	6.2304016	0.0969525	0.5309858
Emg1	0.2240698	6.1483605	0.0970008	0.5310633
Atp7b	0.3801507	3.9743571	0.0970932	0.5313822
Trmt1l	0.233936	5.1558021	0.0972216	0.5317744
Gm20324	-0.6498551	0.1882706	0.0972333	0.5317744
Tmem100	0.6379413	0.8858313	0.0973356	0.5318256
Rgs4	-0.3187221	3.3598836	0.0973655	0.5318256
Cenpt	0.3007991	3.9984105	0.0973926	0.5318256
Rangap1	0.2062351	7.2088742	0.0974197	0.5318256
Draxin	-0.5590849	2.1115144	0.0975024	0.5318256
Snx18	-0.2044951	6.6324123	0.0975248	0.5318256
Dnpep	-0.2556801	4.4295909	0.0975773	0.5318256
Hyls1	0.2643919	3.8590169	0.0975823	0.5318256
Dtd1	-0.2859539	3.4171975	0.0975918	0.5318256
Elk4	-0.2885976	5.5662024	0.0976049	0.5318256
Cox4i1	0.2218893	8.2377163	0.0976401	0.5318256
Btf3	0.2564274	8.2813749	0.097684	0.5318256
Pwp1	-0.2077064	5.3662662	0.09769	0.5318256
Oas3	-0.3256477	5.0189438	0.0977213	0.5318256
Cdadc1	-0.229245	5.6518576	0.097852	0.5323507
Glul	0.2065674	8.1326227	0.0979133	0.5323698
Cthrc1	0.7101029	4.3121063	0.097924	0.5323698
Narf	-0.243911	7.4142768	0.09799	0.5325052
Sik2	-0.2128563	5.7142994	0.0980174	0.5325052
Map1a	-0.3230711	3.9824826	0.0980538	0.5325171
Setdb2	0.6425004	1.0342645	0.0981039	0.5326029
Park7	0.1745805	6.5001997	0.0982644	0.5326529
Zbed4	0.2089354	4.9826277	0.098273	0.5326529
Traf2	0.2394798	4.7469044	0.0982737	0.5326529
Hmgn1	0.1808642	7.2172579	0.0982816	0.5326529
Nufip1	0.2515768	3.7871754	0.0982843	0.5326529
Gfm1	0.216175	5.3081217	0.0984074	0.5327943
Thoc2	-0.2102084	6.9898736	0.0984112	0.5327943
Tlr2	-0.2588978	5.0653954	0.0984131	0.5327943
Gm7931	-0.4852734	2.132656	0.0984747	0.532942
Chl1	0.6758237	1.8943545	0.09851	0.5329475
Lsm6	0.220806	6.3469998	0.0985772	0.5331259
Zbtb20	-0.2996996	4.8215381	0.0987117	0.5336678
Igkv12-41	-0.8475208	3.1492398	0.0987734	0.5338159

Plscr1	-0.2476069	5.3044367	0.0988689	0.534065
Ciita	0.5024069	3.8580355	0.0988882	0.534065
Foxl3	-1.2211765	-1.0068677	0.0990079	0.5344589
Gm34908	0.9991108	-0.691858	0.0990711	0.5344589
Hibch	-0.2380682	4.5741789	0.0990819	0.5344589
Angptl3	-1.2511049	-0.2306907	0.0991104	0.5344589
Mrpl12	0.2728532	5.8040247	0.0991605	0.5344589
Rpl14	0.2201315	9.7061776	0.0991673	0.5344589
Cuedc1	-0.3267576	4.5004825	0.0992052	0.5344777
Hunk	0.73417	0.4483816	0.0992542	0.5345566
Capn15	0.2363171	4.2293608	0.0994109	0.5350915
Armc9	0.2995621	3.5505142	0.0994223	0.5350915
Kif5b	-0.2002423	7.7639742	0.0995557	0.5352201
Rubcn	0.2849881	4.6704097	0.0995886	0.5352201
Pxdc1	0.4399098	3.2844459	0.0995927	0.5352201
Nr1d1	0.4776276	3.7913497	0.0996038	0.5352201
Nr2f2	0.4560598	3.0175232	0.0996457	0.5352201
Zfp111	-0.3234791	3.0941589	0.0996527	0.5352201
Eif2ak1	0.2376047	7.8442214	0.0997329	0.5352916
Mcfd2	-0.2007345	5.4820488	0.0998058	0.5352916
Eif3i	0.2232961	6.8106605	0.0998144	0.5352916
Pola2	0.2513301	6.0531058	0.0998316	0.5352916
LOC118568764	-0.5641988	1.5179196	0.0998797	0.5352916
Ttpa	-0.8970869	0.2501701	0.0998989	0.5352916
Tipin	0.3263508	6.8984951	0.0999069	0.5352916
Hpf1	0.2451208	6.2652414	0.0999467	0.5353206
Abcb6	0.3014217	5.7572637	0.1000082	0.5353829
Aida	0.1772632	5.8919978	0.1000272	0.5353829
F730016J06Rik	-0.3304522	3.1671987	0.100089	0.5354126
E2f2	0.2525441	8.4280311	0.1001298	0.5354126
Col6a4	1.0383053	1.1329749	0.1001566	0.5354126
Tmem11	0.242341	4.573261	0.1001704	0.5354126
Gfap	0.3893897	3.9590302	0.1002232	0.5355107
Prkd3	-0.2672547	6.2367056	0.100323	0.5355126
Amotl2	-0.2855688	4.4802887	0.1003241	0.5355126
Osbpl11	-0.1910726	5.2991259	0.1003268	0.5355126
Cuta	0.2267822	5.210716	0.1004737	0.5359589
Irf4	0.5877225	5.4227365	0.1004794	0.5359589
Amot	-0.3195861	3.2137884	0.1005843	0.5363344
Dpysl3	0.4161246	6.0893064	0.1006292	0.5363531
Sh3bp2	-0.2194851	5.2742379	0.1006567	0.5363531
Rnf157	0.3629429	3.1441825	0.1007659	0.5366432
Cotl1	-0.2361255	8.6052646	0.1007978	0.5366432

Ubqln4	0.2003409	5.5048376	0.1008378	0.5366432
Gm15658	-0.6749613	0.2801934	0.1008527	0.5366432
Dtx1	0.6252586	1.8485818	0.1009362	0.5366432
1500002F19Rik	-0.8329504	-0.1952955	0.100966	0.5366432
LOC118568062	-1.0088654	-0.4846732	0.1010183	0.5366432
Unc13d	-0.2190692	6.1117924	0.1010728	0.5366432
Gm15500	0.2492673	4.1727366	0.1010804	0.5366432
Gm35144	0.7001902	0.1253606	0.1010841	0.5366432
Atxn7l2	0.5271334	2.3558137	0.1010907	0.5366432
Gm15708	-0.8268278	0.1188537	0.1011587	0.5366514
Adgrf5	-0.2575765	5.3637518	0.1012216	0.5366514
Map7	0.335949	4.1138672	0.1012379	0.5366514
Pdlim7	-0.2219137	5.5061451	0.1012612	0.5366514
Snrpe	0.3434719	7.1974637	0.1013146	0.5366514
Pcdh7	-0.4121069	4.9532444	0.1013187	0.5366514
Gm9243	0.6267686	0.9075464	0.1013337	0.5366514
Abca2	-0.23576	5.088908	0.1014459	0.536802
Plppr4	-0.8640362	1.2242782	0.101458	0.536802
Mterf2	0.3730346	2.4415978	0.1014657	0.536802
Trmt12	0.3282194	3.8120095	0.1015707	0.5371667
Gm9895	0.573306	1.7642427	0.1016037	0.5371667
Gskip	-0.2427284	4.6455673	0.1016436	0.537195
Klrb1f	-0.5033934	2.6884512	0.1017551	0.5375406
Gm13841	1.5637615	3.5199587	0.1017781	0.5375406
Mcm2	0.2382444	8.0816636	0.1019463	0.5379497
Atp1a3	-0.3263909	5.5608477	0.1019824	0.5379497
Cyp2j9	-0.6656312	-0.0874716	0.101988	0.5379497
LOC118568581	-0.5414955	1.5389734	0.1019939	0.5379497
Rps4l	0.5927683	2.270243	0.1022628	0.5391265
Mxra7	0.6987777	2.0628626	0.1023204	0.5391265
Gm26847	0.6208932	0.5463559	0.1023427	0.5391265
Angel1	0.5910791	1.6285631	0.1023557	0.5391265
Tcof1	0.2260861	5.7951423	0.1025311	0.5397636
Npl	-0.3387367	3.0865729	0.1025792	0.5397636
Gm46659	0.5466478	0.7401092	0.1025808	0.5397636
Pim3	0.4416332	3.6900496	0.1026523	0.5398057
Rap2a	-0.2196739	5.2282247	0.1026582	0.5398057
Chtop	0.171364	7.2735668	0.1027466	0.5398848
Abcd2	-0.2632328	5.7919302	0.1027574	0.5398848
Kif1b	-0.2251009	6.1811001	0.1029043	0.5398848
Pde9a	0.6173662	0.6768084	0.1029168	0.5398848
Rwdd2b	0.3233032	2.8034387	0.1029606	0.5398848
Toe1	0.2324343	4.2162746	0.1029834	0.5398848

Fbxl6	0.2457419	4.0357291	0.1029993	0.5398848
Alox5ap	-0.254824	8.0463898	0.1030087	0.5398848
Nexn	-0.8269249	0.8859287	0.1030291	0.5398848
Pros1	-0.2255155	6.1563977	0.103094	0.5398848
Rhoq	0.2810897	5.1758357	0.1030998	0.5398848
Sigmar1	0.2813658	4.0367009	0.103106	0.5398848
Slc38a3	-1.5313207	0.0277507	0.1031245	0.5398848
Asb2	-0.6039148	1.9182072	0.1032327	0.5402236
Rlf	-0.1717709	6.161777	0.1032586	0.5402236
Kel	0.3583842	7.6974379	0.1033572	0.5405272
Tmem60	0.349957	4.1245105	0.1033862	0.5405272
Snhg1	0.343778	5.1202725	0.1034645	0.5407354
Tmem147	0.2273629	4.4727403	0.1035105	0.5407354
Gm43380	0.6904145	1.202776	0.103549	0.5407354
Gm14322	0.5612416	0.56598	0.1035994	0.5407354
Usp12	-0.2662062	5.7416039	0.1036181	0.5407354
Cerk	0.2964192	6.5180727	0.1036508	0.5407354
Celf4	0.8073748	0.4323099	0.1037001	0.5407354
Hspd1-ps3	0.4544143	1.3516123	0.1037232	0.5407354
Gm14200	0.6862775	1.2263427	0.1037439	0.5407354
Rbm14	0.2261279	5.7959088	0.1037823	0.5407354
Mug1	-1.3591914	1.378499	0.1038084	0.5407354
A730036I17Rik	0.3853499	2.6349405	0.1038606	0.5408263
Rnft1	-0.2260904	4.5680456	0.1039207	0.5408752
Ddx11	0.2643852	4.2609489	0.1039673	0.5408752
Tlcd1	0.3890316	3.970651	0.1039763	0.5408752
Dnttip1	0.2180923	5.2732918	0.1040091	0.5408752
Lyz1	-0.543644	1.7965595	0.1040636	0.5409777
Hnrnph1	0.2008072	8.8011016	0.1041439	0.541214
Stag3	0.9253868	1.4484094	0.1042356	0.5415098
Ppp1r16b	0.4757184	3.8061567	0.1043058	0.5416938
Bmt2	-0.2152142	4.9249405	0.1043582	0.541785
Il2rb	-0.3064815	3.4702949	0.1044933	0.542245
Gm32234	-0.8669813	0.3764881	0.1045166	0.542245
Orai1	-0.2196886	5.1796573	0.1046569	0.542302
Pebp1	0.1975081	6.4434259	0.10469	0.542302
Chpf	0.4620145	3.6731388	0.1047426	0.542302
Tmem44	0.6462408	0.6584164	0.1047568	0.542302
Tmem41a	0.3813351	2.9112084	0.1047652	0.542302
Gm7694	-0.2362674	3.9913479	0.1047736	0.542302
Romo1	0.2483701	5.4929133	0.1048053	0.542302
Pdzrn3	0.5953261	2.2695266	0.1048301	0.542302
LOC118567599	1.0807368	-0.897945	0.1048413	0.542302

Ercc5	0.2299831	4.0219603	0.1049529	0.5426777
Ccl8	1.1670932	-0.7306146	0.1049994	0.5426777
Crat	0.2910833	6.0710153	0.1050186	0.5426777
Rnf103	-0.2233805	4.4633968	0.1051295	0.5430701
Foxk2	0.2284178	5.9217658	0.1051687	0.5430922
Elmo1	-0.2292415	6.3514612	0.1052651	0.543114
Cttn	-0.2012275	5.76895	0.105279	0.543114
Prrx1	0.4397518	5.0597336	0.1053275	0.543114
Fbxo5	0.2138505	6.8487553	0.1053612	0.543114
Mthfd2	0.2903875	5.577008	0.1053654	0.543114
Ms4a3	-0.35308	7.0335945	0.1053824	0.543114
Fam189a1	0.9593837	-0.6511221	0.1054545	0.5431301
Rabgap1l	-0.2023977	6.937602	0.1054763	0.5431301
Rab5a	-0.1675798	5.8449455	0.1054903	0.5431301
Zfr	-0.1887649	6.9596392	0.1056394	0.5437176
Myl9	-0.2199882	6.6182292	0.1058078	0.5443776
Dock1	-0.3063415	5.1376154	0.1058376	0.5443776
Gpr137c	-0.6434341	1.8313043	0.1060746	0.5448605
LOC118568705	1.4525407	0.8303465	0.1060885	0.5448605
Gm16907	0.7052164	0.0774475	0.1061817	0.5448605
Gpr157	-0.333946	3.1901517	0.1062088	0.5448605
Sash1	-0.2887528	5.318321	0.1062928	0.5448605
Sptbn1	-0.2314295	8.0038884	0.1062958	0.5448605
Tfpi	-0.1906308	6.7399796	0.106296	0.5448605
Gm46877	0.689285	-0.0720148	0.1063108	0.5448605
H2bc12	0.9766526	-0.6809948	0.1063191	0.5448605
Pak2	-0.2434662	7.4475211	0.1063349	0.5448605
Frg1	0.2033214	6.1493114	0.1063441	0.5448605
Ndufs5	-0.3481403	4.522596	0.1063992	0.5448605
Gpaa1	0.2255389	4.9063575	0.1064211	0.5448605
Ybx1-ps2	1.0003945	0.2640011	0.1064613	0.5448605
Acadl	0.2002181	6.5522936	0.1064657	0.5448605
Iqcd	0.5101087	1.5792554	0.1065259	0.5448605
Ankrd54	0.2496609	4.7615755	0.106527	0.5448605
Gm21092	-1.1419521	-0.7060804	0.1065767	0.5448793
Slc25a51	0.2635482	8.355213	0.1066007	0.5448793
Ptpn21	-0.3306089	2.819159	0.1066804	0.5451074
Mmp11	0.3736853	2.3648187	0.1067662	0.5451192
Stoml1	0.3412305	3.0562294	0.1068164	0.5451192
Mcrs1	0.189374	5.0369741	0.1068259	0.5451192
Sgcd	0.4792212	3.1838038	0.1068473	0.5451192
Dnajc9	0.1991833	7.2730236	0.1068763	0.5451192
Igkv4-86	-0.7922097	1.7720339	0.106893	0.5451192

Rps2	0.5419152	8.7091066	0.1069985	0.5451764
Plk3	0.4069781	2.4147006	0.1070092	0.5451764
Gm31166	-0.3023545	3.5959341	0.1070094	0.5451764
Akap13	-0.2519067	8.1308117	0.1071524	0.545443
Akap11	-0.1722286	6.2052488	0.1071768	0.545443
Rgs12	0.2656988	5.5094025	0.1071972	0.545443
Kif13b	-0.1900002	5.4680982	0.1072078	0.545443
Kdm5b	-0.2020282	5.4940934	0.107237	0.545443
Gm8210	1.7535491	1.9273121	0.1074114	0.5458796
Pcf11	-0.2056532	6.8050844	0.107449	0.5458796
Ramp2	0.4673823	2.4531916	0.1074699	0.5458796
Marchf1	-0.2711732	5.4614967	0.1075301	0.5458796
Dmtn	0.3314502	7.7020974	0.1075643	0.5458796
Sspn	0.422426	3.2695666	0.1075665	0.5458796
Mcrip1	0.2222618	5.1690965	0.1075755	0.5458796
Mta2	0.1935368	7.6129372	0.1076036	0.5458796
Cherp	0.1935812	6.5437605	0.1077041	0.5460577
Igkv6-15	-0.6349501	4.9653733	0.1077201	0.5460577
Dynlt1a	-0.4253479	3.2977898	0.1077593	0.5460577
Rpl17-ps5	0.5906623	0.3252908	0.1077792	0.5460577
Eri2	0.3660384	4.2988697	0.1078579	0.5462785
Dyrk1b	0.291001	4.6454852	0.1078994	0.5463109
Rnf24	-0.2839761	3.8029428	0.1080484	0.5468873
Meak7	0.3906653	2.231789	0.1080941	0.5469407
Slc43a1	0.3351171	7.2714697	0.1083031	0.5471159
Gpr37	-0.9436951	-0.3914347	0.108408	0.5471159
Rp2	-0.2305228	6.5759673	0.1084179	0.5471159
Deaf1	0.195874	4.6497019	0.1084181	0.5471159
Prkca	-0.3260618	6.0410827	0.1084795	0.5471159
Gm39469	-0.3007631	2.9671489	0.1084802	0.5471159
Timp3	-0.244279	7.2129576	0.1085081	0.5471159
Tent5c	0.4197091	8.8620783	0.1085209	0.5471159
Washc2	-0.2141547	6.5544163	0.1085402	0.5471159
Ruvbl1	0.275523	5.2253743	0.1085621	0.5471159
Zfp993	-0.6355886	-0.0484372	0.1085923	0.5471159
Gm39876	0.5792543	0.8265073	0.1085967	0.5471159
Slc25a42	0.3257965	3.4737501	0.1085975	0.5471159
Chd9	-0.2124955	6.2449199	0.1086908	0.5471159
Ndufs2	0.1699843	6.5685792	0.1086934	0.5471159
Ppp2r5b	0.3194019	5.39527	0.1087488	0.5471159
Mid1ip1	-0.188393	6.3358423	0.1087834	0.5471159
Map7d3	-0.5803183	1.5569724	0.1088124	0.5471159
Dtymk	0.2023382	6.6357494	0.1088273	0.5471159

Agps	-0.2601626	7.4143547	0.1088462	0.5471159
Ppp1r35	0.2951181	4.477111	0.1089001	0.5471159
Sgpl1	-0.168244	6.5401333	0.1089026	0.5471159
Pdcd11	0.2188684	5.2915472	0.108944	0.5471473
Ly96	-0.3465414	2.2725305	0.10921	0.5478967
Golga4	-0.2050307	6.441451	0.1092628	0.5478967
Ttc3	-0.1912552	5.9074592	0.1093256	0.5478967
Gm32462	-0.6362027	-0.1158584	0.1093904	0.5478967
Bysl	0.2299887	5.0796693	0.1094416	0.5478967
LOC118568687	0.8586249	-0.0283624	0.109472	0.5478967
Ddx54	0.2456628	5.9182113	0.1094955	0.5478967
Got2	0.1822899	6.8644823	0.1095013	0.5478967
Fntb	0.3002799	4.5267093	0.109514	0.5478967
Pde4dip	-0.2858863	5.0304665	0.1095258	0.5478967
Zfp808	0.3120129	4.4770659	0.1095474	0.5478967
Plin1	0.9575789	2.7197827	0.1095537	0.5478967
Slc16a1	0.2881141	8.4485605	0.1095905	0.5478967
Tfrc	0.3106525	10.0299	0.1096568	0.5478967
Adssl1	-0.3106952	5.7573228	0.109712	0.5478967
Tubg1	0.2338079	5.0802904	0.1097176	0.5478967
Pcdhgc3	-0.4512923	4.5084827	0.1097245	0.5478967
Mrm2	0.3844283	2.5095795	0.1097273	0.5478967
Gm12715	-0.3179843	4.4365056	0.1097856	0.5480121
Zfp108	-0.5761722	0.0280504	0.1098285	0.54805
Mycn	-0.5099508	1.4154402	0.1099298	0.5483798
Wdr43	0.1800946	6.2097984	0.109986	0.548484
Abcb10	0.3175039	8.2015514	0.1101971	0.5491375
Pnpla1	-0.8343733	0.982994	0.1102461	0.5491375
Hacd2	-0.2353984	4.9600784	0.1102902	0.5491375
Papolg	-0.1911892	4.9584375	0.1102986	0.5491375
Mrps23	0.1917212	5.2186516	0.110339	0.5491375
Dcaf6	0.2387786	6.6380734	0.110366	0.5491375
Gpr35	-0.3347209	2.9662594	0.1104066	0.5491375
Cnnm4	-0.3210105	3.9764747	0.1104128	0.5491375
Trpc3	-0.8094107	-0.0040963	0.1104348	0.5491375
Pla2g5	0.7219609	3.0175701	0.110551	0.54954
Rcc1l	0.2942133	3.2136212	0.1106212	0.549713
Ssu72	-0.1805609	5.8254522	0.1106689	0.5497744
Slc15a4	0.2410825	4.920817	0.1108694	0.5505317
Rusf1	0.2520517	4.5566644	0.1108921	0.5505317
Begain	1.0367821	-0.4486777	0.11094	0.5505938
Prg3	-1.6096928	5.3551293	0.1111261	0.5513413
Rrm2	0.2270285	9.0615572	0.1111959	0.5515118

Gars	0.1842631	6.6517931	0.1112505	0.5516066
Zcchc18	0.7058154	0.7709013	0.1113163	0.5517569
Phlda1	-0.306711	3.9105145	0.1114212	0.551934
Gm10767	0.7971823	-0.8368942	0.111423	0.551934
Psen2	0.2173927	4.9219088	0.1115292	0.5520075
Snai2	0.4270311	3.5984209	0.1115346	0.5520075
Slc10a3	-0.3122532	3.1275298	0.1115443	0.5520075
Vangl1	0.3134345	4.6013244	0.111639	0.5522543
Eloa	-0.1774934	6.8165095	0.1116825	0.5522543
Wdr6	0.205373	5.1300571	0.1117007	0.5522543
Sncaip	-0.5882021	1.5080947	0.1118082	0.552366
AB124611	-0.2175585	6.6093013	0.1118133	0.552366
Thap3	0.4814415	2.5407137	0.1118298	0.552366
Slc16a13	0.4434119	1.3099385	0.1118774	0.5524259
Mybbp1a	0.2057579	6.7793263	0.1120093	0.5527344
Layn	-0.3543037	2.837551	0.1120776	0.5527344
Exosc1	0.2789811	3.9624409	0.1120887	0.5527344
BC024063	0.7961418	0.5974206	0.1121593	0.5527344
Abhd16a	0.2069112	5.8630514	0.1121764	0.5527344
Gtf2ird1	-0.3272765	3.5996649	0.1121874	0.5527344
Rce1	0.2957859	3.6198445	0.1121887	0.5527344
Ttc5	0.2483235	4.5843448	0.112278	0.5528581
5033406O09Rik	-0.4236551	2.0116685	0.1123116	0.5528581
Lrrc47	0.1936336	5.9876333	0.1123338	0.5528581
Angptl7	0.9710489	0.1207974	0.1123559	0.5528581
Gid4	0.1800712	5.4338719	0.1124449	0.5530691
Gm33148	-0.6015148	2.31867	0.11247	0.5530691
Pde3b	0.5384063	4.5090214	0.1125676	0.5532924
Psmd7	0.2029956	6.5259853	0.1125992	0.5532924
Pik3cb	-0.1922827	6.1327079	0.1126221	0.5532924
Hmces	0.258671	3.6325945	0.1127956	0.5535324
Pank4	0.2333919	5.1082791	0.1128065	0.5535324
Skiv2l	0.1799492	6.0254373	0.1128081	0.5535324
Nrg4	-0.495888	1.1503431	0.1128366	0.5535324
Ssrp1	0.1956259	8.2723441	0.1128556	0.5535324
Dlst	0.1877004	7.3906585	0.1128845	0.5535324
2210016F16Rik	0.2232702	4.6552649	0.1129227	0.5535453
P2rx3	0.5796966	2.2310691	0.1132248	0.5547796
Plekhg1	-0.2981297	4.1834559	0.1132599	0.5547796
Map2k3os	0.813818	0.4063939	0.1132815	0.5547796
Rpain	0.2657382	4.4397734	0.113503	0.5556893
Rnf227	0.474694	1.6799952	0.1136179	0.5559835
Hpse	-0.3445124	5.0503422	0.1136345	0.5559835

Mrps35	0.2381276	4.3657384	0.1137177	0.5561058
Rai1	0.2331923	5.966006	0.113735	0.5561058
Mapk13	-0.3253124	5.0875117	0.1137668	0.5561058
Zfp784	0.4539271	1.2185194	0.1138519	0.5561622
Slc35g1	0.3154524	4.3756253	0.1138865	0.5561622
Pycrl	0.2597834	3.7884279	0.113898	0.5561622
Marveld2	1.0242576	-0.008923	0.1139407	0.5561622
Mri1	0.2675347	3.4710776	0.1139571	0.5561622
4930432K21Rik	-0.7510759	-0.8479186	0.1141406	0.556883
Eif2d	0.1984748	5.0287514	0.1143854	0.5579025
Ncf4	-0.2515643	7.6065337	0.1144666	0.558099
Kdm3a	-0.1849071	5.7163132	0.1145135	0.558099
Rpl6l	0.3612464	3.0808831	0.1145334	0.558099
Hdac11	0.4206636	2.2089171	0.1146356	0.5582396
Acox3	-0.2132536	5.7512145	0.1146641	0.5582396
Marchf5	0.2717152	7.4185224	0.1146875	0.5582396
Cactin	0.1851196	5.7635621	0.1147058	0.5582396
Prg2	-1.6048207	9.2306629	0.1148603	0.5588168
Apbb1ip	-0.2394285	6.9372225	0.1149968	0.5593058
Slc2a4	0.4055362	4.1762834	0.1151165	0.5594545
Ap1s2	-0.2544218	6.3133815	0.1151464	0.5594545
Igkv4-91	0.845258	2.6145018	0.11516	0.5594545
Hoxb2	0.7730866	1.6298297	0.1152026	0.5594545
Rbm41	-0.3032575	4.3340487	0.1152209	0.5594545
Pja2	-0.176608	6.2780469	0.115267	0.5594545
Zc3h12a	-0.3339576	3.1037332	0.1152791	0.5594545
Jazf1	-0.6015307	0.5444375	0.1153229	0.5594925
Srf	-0.2337118	5.9961799	0.1154255	0.5598154
Themis	-0.7146814	-0.2191165	0.1156523	0.5607406
Naip6	-0.5556232	2.5420672	0.1157309	0.5609469
Afap1l2	-0.3694948	3.4111834	0.1158694	0.561443
Ifih1	-0.2924231	4.778346	0.1159809	0.5615938
E230032D23Rik	-0.5460366	0.7321267	0.1159842	0.5615938
Impdh2	0.1856717	6.7445707	0.1160372	0.5615938
Rasgrp1	0.392607	4.6020513	0.1160649	0.5615938
Gsk3b	-0.1774347	6.671917	0.116081	0.5615938
Igsf3	0.3273213	4.8482303	0.1161713	0.561856
Mrpl15	0.2460124	5.1807616	0.1163121	0.562362
Rnf141	-0.1684735	6.1101461	0.1164668	0.562935
Jmjd1c	-0.1978276	7.0592823	0.1165661	0.5630171
Far2	-0.4136195	3.701529	0.1165782	0.5630171
Patj	0.5219035	1.2980906	0.1165924	0.5630171
Jpt1	0.1789187	6.7153255	0.1166351	0.5630486

Gm51785	-0.6219735	0.5364839	0.1167824	0.5635622
Rdm1	0.3060738	4.1621433	0.116814	0.5635622
Zyg11b	-0.2168295	5.9746264	0.1171611	0.5650618
Rbm3-ps	0.3929265	1.8471379	0.1172403	0.5652681
Ncor1	-0.255518	8.1153931	0.117383	0.5657812
Gm16464	-0.6014171	0.5149744	0.1174781	0.5658522
Txndc15	0.2444611	4.50923	0.1174987	0.5658522
Sbno2	-0.1863181	6.503336	0.1175174	0.5658522
Apaf1	-0.2552969	6.7392582	0.1176062	0.5658522
Fhl3	-0.2346931	4.3685261	0.1176186	0.5658522
Marchf3	0.3690752	5.2901494	0.1176431	0.5658522
Mad2l1bp	0.2762775	4.7814718	0.1176718	0.5658522
Ppp3cc	0.3208383	3.7264159	0.1177487	0.5658522
Cxcl16	0.5140778	2.5489198	0.1178168	0.5658522
Gemin6	0.3385876	3.4032343	0.1178332	0.5658522
Khnyn	-0.2188423	6.2810361	0.1178367	0.5658522
Akr1b3	0.1844652	5.4814615	0.1178512	0.5658522
Letmd1	0.2103282	4.5090996	0.1178707	0.5658522
Meltf	-1.0788677	-0.1403845	0.1179557	0.5660137
Nr2f6	0.2996692	3.5096965	0.1179771	0.5660137
Uros	0.351662	6.6959974	0.1180311	0.566098
1700097N02Rik	0.8145732	2.0367272	0.1180773	0.5661146
Klrk1	-0.5288654	2.15768	0.1181074	0.5661146
4930451G09Rik	-0.7105165	0.2721859	0.1182297	0.5662163
Gdf11	0.4404591	3.4415591	0.1183091	0.5662163
Myo15b	-0.7148997	-0.0018721	0.1183096	0.5662163
Tnfsf13b	-0.2856966	4.1178069	0.1183382	0.5662163
Rlim	-0.1677749	7.0946438	0.1183682	0.5662163
Tnfsf8	-0.6362486	1.4051318	0.1183742	0.5662163
Rhag	0.302514	7.9100964	0.118398	0.5662163
Ntn1	-0.6367055	0.9173517	0.1184206	0.5662163
Rae1	0.2014134	5.5369499	0.118463	0.5662163
Ascc3	-0.2097257	5.7514796	0.1184926	0.5662163
Ifi203	0.2046797	6.2000795	0.1185983	0.5664858
Pabpn1	0.1762651	5.4825092	0.1186219	0.5664858
Spink2	0.9953621	-0.5457848	0.1187066	0.5665901
Tgds	-0.2730627	3.9995803	0.1187166	0.5665901
Ttc39b	-0.2125625	5.1131989	0.1187859	0.5667063
Calhm5	-0.7073576	0.4435012	0.1188359	0.5667063
Klhl28	-0.2110622	5.0152593	0.1188502	0.5667063
Rbms3	0.368221	3.499575	0.1189215	0.5667137
Pcgf5	-0.1867535	5.5700588	0.1189246	0.5667137
Ap1ar	0.1906508	5.7367397	0.1189759	0.5667186

Ctnnd1	-0.2203807	6.6251182	0.1189985	0.5667186
Asf1b	0.1918486	6.8067104	0.1190444	0.5667637
Irf1	-0.2035271	5.6104324	0.1191034	0.5668711
Tex9	0.3510124	3.8951158	0.1192264	0.5672825
Dlgap2	1.0913386	-0.3068805	0.1193116	0.5674109
Ccnh	0.204216	5.2017412	0.1193263	0.5674109
Runx2	-0.3289908	5.9750996	0.1193984	0.5674757
Gm33168	-0.4228604	1.6678638	0.1194129	0.5674757
Esyt2	-0.1741877	6.9685859	0.1195152	0.567601
Rfx5	0.2315131	4.1875438	0.1195458	0.567601
Alg3	0.3252758	3.1613785	0.1195487	0.567601
Dstyk	-0.1921756	5.0776482	0.1196079	0.5676016
Ctsg	-0.3697236	8.3986975	0.1196462	0.5676016
Gm15760	0.7103335	-0.2801271	0.1196583	0.5676016
Gpd1	0.3991231	3.9451118	0.1197017	0.5676341
Gm15816	0.7397452	-0.2083714	0.1198816	0.5680438
Micall2	0.2977909	5.0227382	0.1199106	0.5680438
Rgs19	-0.1990826	6.1973386	0.1199179	0.5680438
Gm13991	-0.8059103	-0.4681719	0.1199954	0.5680438
Pdgfrb	0.2547021	5.5363332	0.1200059	0.5680438
Lnpk	-0.1966441	5.9978953	0.1200132	0.5680438
Plxna2	0.3590671	4.4121871	0.1200442	0.5680438
Gria3	-0.3304422	4.6297934	0.1200802	0.5680438
Brpf3	0.2488006	7.4747549	0.1202064	0.5681779
Kng1	-0.3231125	4.3707748	0.1202509	0.5681779
P2ry13	-0.3036446	4.1505875	0.1202523	0.5681779
Orc6	0.2943556	6.2056041	0.1203026	0.5681779
Abcc9	-0.3214554	5.8450587	0.1203544	0.5681779
Mrpl4	0.1966032	4.7810541	0.1203593	0.5681779
Gm10768	-0.8060073	0.1328944	0.120401	0.5681779
Pira2	-0.5132445	3.1180692	0.1204193	0.5681779
Hmga2-ps1	-0.2900904	3.9043694	0.1204373	0.5681779
Tma7-ps	1.1290379	3.3238414	0.1204964	0.5682839
Dgkg	-0.2776975	4.701927	0.1205851	0.5683906
Itfg1	-0.1750111	6.1428559	0.1205947	0.5683906
Mpig6b	-0.3023245	4.9957531	0.1206286	0.5683906
Pcyox1	-0.2469005	6.3403556	0.1207469	0.5687227
Rpl37rt	0.2708989	3.3860763	0.1207722	0.5687227
Gcdh	0.2462226	4.6403656	0.1208327	0.5688352
Acat1	0.1738069	6.320119	0.1208703	0.5688401
Kifap3	0.2795309	3.6443534	0.1209658	0.5689996
Dnajc11	0.2414416	5.819325	0.1209773	0.5689996
Vamp2	0.2502984	5.4066973	0.1210427	0.5690832

Abi3	0.2347165	4.4954596	0.1210702	0.5690832
Hspa9	0.1857852	7.5927119	0.1211049	0.5690832
Tuft1	-0.5533769	1.8811131	0.1212045	0.5693417
Tssc4	0.2489235	4.9445297	0.1213012	0.5693417
Gfpt1	-0.1865851	5.7425546	0.1213312	0.5693417
Sart1	0.1678644	6.4438682	0.1213594	0.5693417
Vit	0.4783201	3.2583816	0.1214395	0.5693417
Tmem14a	-0.8301239	0.0016378	0.1214521	0.5693417
Carns1	0.3787318	3.8175707	0.1214707	0.5693417
Mif4gd	0.2688425	4.9693599	0.121538	0.5693417
Grin2d	0.9199617	0.4090997	0.121553	0.5693417
Pabpc4l	0.7591285	0.0730199	0.1215867	0.5693417
Mrpl51	0.2170409	6.1143605	0.1216502	0.5693417
Aoc3	0.7112721	3.1966492	0.1216831	0.5693417
B4gat1	0.2958405	3.1369378	0.1216874	0.5693417
Acad8	-0.2812189	3.7660089	0.1216978	0.5693417
Mcoln2	0.5744009	1.958643	0.1217543	0.5693417
Prss36	0.7780726	0.0168272	0.1217814	0.5693417
Capn3	-0.3429061	2.5928959	0.1218259	0.5693417
Ints7	0.1976031	6.2043135	0.1218482	0.5693417
Klra17	-0.4068602	3.9241546	0.1219766	0.5693417
Zfp639	0.2455162	4.781363	0.1219768	0.5693417
Lrrc8c	0.2032141	6.0033355	0.1220075	0.5693417
Phf10	0.2119046	6.9604316	0.122042	0.5693417
Clec4a3	-0.326411	3.9625692	0.1221458	0.5693417
Agap2	-0.2508171	4.55089	0.1221598	0.5693417
Rps16-ps2	0.2962711	3.9251514	0.1222451	0.5693417
Gm12543	0.6627745	0.0584023	0.1222981	0.5693417
Gm15972	-0.7496552	-0.3420098	0.1223109	0.5693417
Edc4	0.1904689	5.3496649	0.1223869	0.5693417
Map3k6	0.362862	2.2552719	0.1224384	0.5693417
Xpo4	-0.1882926	5.9597875	0.1224435	0.5693417
Clp1	0.2482332	5.0102779	0.122518	0.5693417
Polr3e	0.2607777	4.4122107	0.1225198	0.5693417
Gna13	-0.1912553	7.0930255	0.1226125	0.5693417
Tarm1	-0.3527768	3.2180899	0.1226177	0.5693417
Gm14494	0.4937369	0.4927377	0.1227176	0.5693417
Rag1	0.8058179	6.4719383	0.1227985	0.5693417
Spaca9	-0.7190317	0.2263123	0.1228276	0.5693417
Wscd2	-0.69519	-0.385781	0.1228391	0.5693417
Fdps	-0.3230824	5.1415206	0.1228497	0.5693417
D130040H23Rik	0.4300981	2.7989557	0.1228799	0.5693417
H2bc15	1.0032766	-0.8936648	0.1229727	0.5693417

Chtf18	0.2527896	4.8718229	0.1229895	0.5693417
Prkci	-0.2353243	5.0220482	0.122997	0.5693417
Cdc42bpb	-0.2812983	5.1439857	0.1229972	0.5693417
Crybg3	-0.3070945	4.9759709	0.1230234	0.5693417
Pdrg1	0.2190598	4.8200623	0.123067	0.5693417
Myd88	-0.2006832	5.6235466	0.1230926	0.5693417
LOC118568244	1.0097226	-0.8917609	0.1231038	0.5693417
Kctd20	-0.1965888	7.3667057	0.1231488	0.5693417
Net1	0.1888528	6.7829946	0.1231493	0.5693417
Hipk1	-0.2380803	9.2781799	0.1231647	0.5693417
Serpinb9	-0.2581951	3.8012235	0.1231706	0.5693417
Prok2	-0.7351084	2.1755062	0.1231846	0.5693417
Znhit2	0.2662013	3.6846789	0.1231887	0.5693417
Rbbp7	0.1849311	7.6490218	0.123192	0.5693417
Ran	0.2190496	8.5499971	0.1232349	0.5693417
Atg4c	-0.2813409	3.4557049	0.1232572	0.5693417
5031434O11Rik	0.6548051	-0.0500204	0.1232829	0.5693417
Dnajc4	-0.3142603	3.3785039	0.123336	0.5693471
Stat6	-0.1648183	6.7150578	0.1233573	0.5693471
Llgl1	0.1907065	4.8760018	0.1234105	0.5694234
Prdm16	-0.5240253	0.5660352	0.1234837	0.5695887
Mipep	0.2908344	4.1705212	0.1235234	0.5695887
Tgfb3	-0.4132876	4.3439126	0.1235562	0.5695887
Gm36587	-0.6459877	-0.2208387	0.1237099	0.5700628
Plekha7	-0.6349348	0.6383557	0.1237323	0.5700628
Nf1	-0.226829	4.8256898	0.1238113	0.5702517
Gm41308	-0.7609683	-0.3452033	0.1238466	0.5702517
Itgae	0.9796678	-0.6109681	0.1239307	0.5702735
Spcs3	-0.1926225	7.2840726	0.1239733	0.5702735
Igf2r	-0.1850277	5.9514588	0.1239912	0.5702735
Tsfm	0.2905892	3.8772516	0.123998	0.5702735
D730003I15Rik	0.5415658	0.96511	0.124055	0.570367
Il18	-0.365333	2.8426108	0.1241753	0.5707516
Chaserr	0.2390486	5.4466087	0.1242278	0.5708173
Cdan1	0.2240181	5.2625186	0.1242937	0.5708173
Cpeb1	0.8103773	0.4017374	0.1243261	0.5708173
Epha3	-0.7335741	0.3493598	0.1244139	0.5708173
Gm39427	-0.5721867	1.453025	0.1244341	0.5708173
Prdx2	0.339863	9.7524064	0.1244352	0.5708173
Zfp202	0.5567159	1.0384711	0.1244465	0.5708173
Gm16477	0.947999	-1.0550605	0.1245152	0.5709637
Ccna2	0.197487	8.5019547	0.1245927	0.5710714
Rab4a	0.3694773	2.8015335	0.1246121	0.5710714

Cacna1e	0.602416	4.4849972	0.124789	0.5717032
Mcee	-0.2465804	4.2430845	0.1248375	0.5717032
Dnmt3b-ps1	0.5747914	0.7818743	0.1248602	0.5717032
0610012G03Rik	0.2234927	4.7080181	0.1251303	0.5723558
Stx17	-0.2095156	5.6746955	0.1251308	0.5723558
Klf2	0.2493185	6.0904809	0.1251352	0.5723558
Gja4	-0.5858227	0.4090701	0.1251718	0.5723558
Hspa13	-0.1981822	5.3440914	0.1251867	0.5723558
Gpr137	-0.2604671	3.856233	0.1253316	0.5728419
Gm2991	1.0082598	-0.6060509	0.1253667	0.5728419
Rpl13a	0.3085737	10.449547	0.1254082	0.5728633
Tspan18	0.3632932	2.7568026	0.1254863	0.5729849
Strbp	0.246138	5.8837375	0.1255085	0.5729849
Pidd1	0.2253918	4.7697565	0.1256065	0.5732594
Ccdc82	-0.1844248	6.3636784	0.1256559	0.5732594
Cstf1	0.2076271	4.9662299	0.1257026	0.5732594
Crebl2	-0.2415253	3.838979	0.1257161	0.5732594
Otud7b	-0.190245	6.3368962	0.1258318	0.5736189
Rsf1	-0.1897725	6.1176651	0.1259213	0.5736933
Cd63-ps	-0.4341316	1.3411199	0.1259612	0.5736933
Urm1	0.2116022	4.5335391	0.1259638	0.5736933
Gm9620	-0.7304362	-0.4622109	0.1260262	0.5736933
Fam32a	-0.2008402	6.9549718	0.1260369	0.5736933
Smarce1-ps1	-0.9290851	-0.58918	0.1260694	0.5736933
Denn2b	0.2194782	5.2980741	0.1261709	0.5739872
Cib2	-0.3714665	3.2731029	0.1263062	0.5744346
Rfk	0.169873	6.0059506	0.126447	0.5747667
Rpl27	0.2199276	8.2834096	0.1264817	0.5747667
Parvg	-0.2416536	6.9665422	0.1264901	0.5747667
Map3k8	-0.3121997	2.6548871	0.1265513	0.574877
Fnbp1	-0.2311937	6.7340244	0.1266554	0.5750043
Prmt7	0.2710039	3.7698907	0.1266869	0.5750043
Psmd4	0.1855696	6.9091078	0.1266902	0.5750043
Syn1	-0.8756882	0.1463494	0.1267579	0.5750563
Rps26-ps1	0.3278467	3.2000254	0.1267756	0.5750563
Camk1	-0.2580731	5.3647425	0.1268696	0.5751919
Ly86	0.2575469	5.211438	0.1268795	0.5751919
Ttbk2	-0.2194105	4.9666608	0.1269792	0.5752167
Pbxip1	-0.1846478	6.8373098	0.1269874	0.5752167
Rpl5-ps2	0.8040648	-0.9949644	0.1270251	0.5752167
Ell	-0.2015776	4.6497703	0.1270329	0.5752167
Samm50	0.1737301	6.3768327	0.1271541	0.5754345
Tmem259	0.1756928	6.2021907	0.1271903	0.5754345

5830444B04Rik	-1.7078787	-0.01622	0.127225	0.5754345
Htatsf1	0.1768658	5.9717904	0.1272347	0.5754345
Gm19897	-0.4047339	1.2876002	0.127266	0.5754345
Cd160	-0.8882828	-0.1372749	0.1273711	0.575625
Parp14	-0.2127609	5.7114008	0.1273821	0.575625
Tctn1	0.2820831	3.8018311	0.1274195	0.5756265
Ripk1	-0.1921274	5.8890866	0.1275733	0.5760221
Exosc2	0.213873	4.8818273	0.1275811	0.5760221
Mfap3	-0.1870345	5.6062835	0.1277228	0.5764944
Prpf40b	0.4499817	2.4160091	0.1277721	0.5765496
3830408C21Rik	0.7048754	-0.3239318	0.128013	0.5773076
Sox12	0.2685278	3.8863102	0.1280143	0.5773076
Cybc1	0.2217844	5.9327682	0.1281492	0.5777487
Rad51	0.1972891	5.4765474	0.1283711	0.5785069
Tmem79	-0.6263557	-0.3534454	0.1283918	0.5785069
Cdca8	0.1946336	6.8715046	0.1284599	0.5785535
Cubn	-0.7961621	-0.4177944	0.1284765	0.5785535
S1pr4	-0.2898629	5.1566131	0.1287599	0.5791629
Rpa1	0.2054979	7.3100335	0.1287904	0.5791629
Epx	-1.506444	7.1418455	0.1287981	0.5791629
Aven	0.2531575	3.8082389	0.1288421	0.5791629
Ccdc180	-0.6407718	1.2271577	0.1288456	0.5791629
Stx7	-0.1835596	6.6865876	0.1288555	0.5791629
Gm33310	0.6492315	0.0503806	0.1288725	0.5791629
Ski	0.2615117	6.3955785	0.1289114	0.5791703
Fkbp8	0.165	7.1945997	0.1292451	0.5804294
Dhps	0.2464787	3.5932867	0.1292663	0.5804294
Akr1b8	0.45151	1.4678349	0.1293578	0.5806728
Eepd1	-0.2821335	3.3930562	0.1294348	0.5807507
Gm8941	1.1846	-1.3949941	0.1294498	0.5807507
Rpl28-ps1	0.2900072	2.7965684	0.1296023	0.581113
Faah	-0.6014818	1.1683053	0.1296425	0.581113
Tfap4	0.2632927	3.748031	0.1296578	0.581113
Nfic	-0.2056431	6.1761434	0.12968	0.581113
Pcolce2	-0.630059	2.2083094	0.1297437	0.5811565
Vps13c	-0.2510279	6.2455607	0.1297981	0.5811565
Apeh	0.2624483	6.6007801	0.1298018	0.5811565
Klk1	-1.2742478	0.5617161	0.1299594	0.5816945
Hook1	-0.2656948	3.353186	0.1301611	0.5824295
Dab2ip	-0.2382146	5.3437739	0.1302893	0.5825927
Mamld1	0.5076024	0.509322	0.130343	0.5825927
Gm52182	0.3408086	3.1091967	0.1303513	0.5825927
Gp1ba	-0.3541533	6.221133	0.1303655	0.5825927

Col6a3	-0.4820803	5.9637892	0.1303848	0.5825927
Rps10	0.2222184	8.755757	0.1304796	0.5827793
Gm10371	0.5110306	2.1886533	0.1305015	0.5827793
Fam227a	-0.4681452	0.8664654	0.1306218	0.5831492
Aarsd1	0.2228357	4.8763276	0.1307125	0.5832948
Tmem256	0.3423549	6.2594131	0.1307499	0.5832948
Fam234b	0.2344637	5.4138592	0.130767	0.5832948
Sidt2	-0.1786033	6.2642451	0.1308828	0.5836443
Igsf8	0.216724	4.7021489	0.1309693	0.5838627
Bglap	0.7012524	9.3327239	0.1310267	0.5839509
Klhl14	0.8292481	2.7040257	0.1312532	0.5846736
Hivep1	-0.2597676	5.655083	0.131264	0.5846736
Nav1	-0.2040942	5.5496723	0.1313374	0.5848331
Txnl4a	0.2317347	4.6020026	0.1314666	0.5852408
Zfp638	-0.1856578	6.7918365	0.1315714	0.5853187
Adh1	-0.6533476	2.6325481	0.1316071	0.5853187
Grk3	-0.2639475	5.4110862	0.1316297	0.5853187
Gm46536	-1.0624186	-0.0842562	0.1316346	0.5853187
Npepps	-0.1980598	6.4967774	0.1318194	0.5859518
Sh3d21	-0.7008677	-0.5978375	0.1318766	0.5859518
Gm34254	-1.1107749	-0.6445156	0.1318925	0.5859518
Sec14l2	0.2924731	5.611663	0.1319277	0.5859518
Pak1ip1	0.2438814	5.7264831	0.1320013	0.5861113
LOC100861749	0.5786521	0.063893	0.1321145	0.586331
Ubtd1	-0.2780304	4.1596673	0.1321571	0.586331
Vldlr	-0.5103474	5.3056683	0.1321639	0.586331
Gm30604	-0.5406211	0.5358379	0.132254	0.5864738
Tanc1	-0.2574038	4.9268704	0.1322714	0.5864738
Brd8dc	-0.5378627	1.0269292	0.1323771	0.586775
Zdhhc9	-0.236574	4.6021169	0.1325569	0.5873045
Psmg3	0.2938464	3.5102183	0.1325721	0.5873045
Aqp9	0.247529	4.8145089	0.1327479	0.5877122
Wfdc17	-0.434804	4.6973221	0.1327648	0.5877122
Cuedc2	0.1995245	5.7427193	0.1328312	0.5877122
Fam199x	-0.2599412	3.8177139	0.1328331	0.5877122
Prr14l	-0.19452	6.0023617	0.1329455	0.5877122
Tmem165	-0.1770502	6.3120661	0.1329456	0.5877122
Depp1	0.5303872	2.4728919	0.1329623	0.5877122
Ncoa3	-0.2166614	6.0940828	0.1329797	0.5877122
Plin3	-0.1878138	5.560192	0.1330042	0.5877122
1110006O24Rik	0.4923431	0.8064142	0.1330815	0.5877608
Lasp1	-0.1861164	7.9625322	0.1331381	0.5877608
Bco1	-0.7420675	-0.4134611	0.1331779	0.5877608

Tada2a	0.3172969	3.6400999	0.1331982	0.5877608
Znfx1	-0.1871367	6.214873	0.1332041	0.5877608
Slc29a1	0.1933147	6.7766887	0.1333	0.5880169
Dmap1	0.2645432	3.9365095	0.1333707	0.5881557
Rhog	-0.1790725	7.3327775	0.1334195	0.5881557
Gm45916	-0.5128133	0.8595494	0.133477	0.5881557
Ddx47	0.1670977	5.7240872	0.1334881	0.5881557
Fxyd6	0.6947771	2.5342726	0.1335415	0.5881557
Evl	0.2159537	5.3935845	0.1335956	0.5881557
Gm5837	-0.8033923	-0.4302007	0.1335961	0.5881557
C1rl	-0.3468447	4.0212796	0.1336698	0.5883134
Cpxm1	0.4431612	3.4066418	0.1337147	0.5883448
Siglec1	-0.5208464	4.5480607	0.1337986	0.5885471
Memo1	0.2534452	7.0894116	0.1339472	0.5888137
Prkg2	-0.901292	1.0162192	0.1339708	0.5888137
Car2	0.3486706	11.000333	0.1339727	0.5888137
Polr1d	0.2621527	6.0022887	0.1340486	0.5889806
Gm6377	-0.6263628	0.2099878	0.1342774	0.5895146
Gon7	0.365001	2.6231959	0.1342867	0.5895146
Eef2	0.1924577	10.694839	0.1343229	0.5895146
Osbpl9	-0.1853273	6.8232814	0.1343388	0.5895146
Kctd5	0.2074218	4.243309	0.134389	0.5895146
Prepl	-0.1968342	4.9150969	0.1343975	0.5895146
Hs3st3b1	-0.4960221	1.5524749	0.1344498	0.5895779
Nudt1	0.3184644	3.6649311	0.1345016	0.5896386
Zpr1	0.1990215	4.7517059	0.1346436	0.5900913
Sdf2l1	-0.231214	5.0687663	0.1347537	0.5900913
Cahm	-0.6593173	-0.0082661	0.1347822	0.5900913
Aldob	-0.6453459	2.5255129	0.1347864	0.5900913
Dnah6	-0.6200822	0.9408115	0.1347945	0.5900913
Lca5l	1.040253	-1.0789327	0.1348856	0.5903237
Pspc1	0.1920813	5.6128499	0.1350041	0.5904392
Msrb1	-0.2672888	7.8837705	0.1350216	0.5904392
Gm16685	0.6976263	-0.2715653	0.1350853	0.5904392
Sh3gl1	-0.2132533	5.4672583	0.1350958	0.5904392
Rab36	-0.4780591	2.1646335	0.1351018	0.5904392
Kidins220	-0.2045843	7.0829317	0.1351725	0.5905007
Rasal1	0.6440657	0.787915	0.1351918	0.5905007
Srsf3	0.2248115	8.7103831	0.1353606	0.5908321
App	-0.2019461	8.0688839	0.1354118	0.5908321
Lats2	-0.159642	6.1020558	0.1354572	0.5908321
Srgap1	-0.4172292	3.1729901	0.1354947	0.5908321
Mme	-0.2034653	5.7302973	0.1355297	0.5908321

Pycr1	0.5112158	2.7821492	0.1356161	0.5908321
Yaf2	-0.1992464	5.1795637	0.1356319	0.5908321
Gm20337	0.7573337	-0.1390959	0.1356933	0.5908321
Gm7993	1.0998023	6.1097	0.1357048	0.5908321
Mrpl41	0.2824503	5.2498804	0.135733	0.5908321
Sec14l1	-0.1635865	6.4458638	0.1357798	0.5908321
Bmp5	0.3494172	3.7392616	0.1358232	0.5908321
Gm16386	-0.4714354	1.1444501	0.1358245	0.5908321
Gm5909	0.6769643	0.9109703	0.1358322	0.5908321
Rit1	-0.1769317	5.2256898	0.1358471	0.5908321
Cdcp1	-0.8542019	-0.56814	0.1358754	0.5908321
Vasp	-0.1790174	7.981994	0.1360397	0.5913659
Pitpnm2	0.2799042	4.3004165	0.1360742	0.5913659
Mcur1	-0.1737787	5.4864298	0.1361686	0.591394
Colgalt2	-0.6380246	1.6548256	0.1361851	0.591394
Ddx39a	0.2057254	6.6308359	0.1362075	0.591394
Slc23a1	0.6891983	-0.2149535	0.1362328	0.591394
LOC108167922	0.7409354	-0.7590967	0.1363191	0.591457
Abr	-0.1908792	6.3966867	0.1363247	0.591457
Bag1	0.2450168	7.3078957	0.1363614	0.591457
Myl6	-0.2247844	9.2407901	0.136414	0.5915065
Rmc1	0.2064544	5.3680407	0.136451	0.5915065
Gngt2	0.2625029	4.0896656	0.1364869	0.5915065
Wrap73	0.2595807	3.4927218	0.136531	0.591533
Ntmt1	0.3612431	2.8134606	0.1365874	0.5916125
Arnt	0.1568142	5.9108441	0.1367288	0.5919806
Degs1	-0.1652625	6.840108	0.1367485	0.5919806
Dhtkd1	-0.604623	0.0289696	0.1367983	0.5920313
Obsl1	0.359369	2.6931445	0.1369399	0.5921105
Mrpl11	0.2676524	4.5483077	0.1370055	0.5921105
Spen	-0.2656149	6.9655715	0.137016	0.5921105
Gm41423	0.7641242	-0.8524292	0.1370289	0.5921105
Ankrd39	0.4769063	1.6818644	0.1370356	0.5921105
Bod1l	-0.2278762	6.5779751	0.137045	0.5921105
Taf1d	0.2246361	6.0146866	0.1370859	0.5921228
Ckap4	-0.1944527	7.9041985	0.1371638	0.5922948
Nap1l1	0.1841673	8.5629603	0.1372453	0.5924823
Ces2g	0.3321974	7.4766223	0.1373351	0.5926834
Atn1	-0.2172289	5.5453918	0.1373681	0.5926834
Mef2d	0.2268338	6.7209836	0.1374559	0.5927456
Ttc39a	0.5423441	2.263494	0.1374945	0.5927456
Als2cl	-0.4203983	2.6292374	0.1375141	0.5927456
Gm7912	-0.9236913	-0.9668191	0.137535	0.5927456

Fhit	0.5741904	0.0863621	0.1376148	0.5927998
Ptger2	-0.431411	2.593711	0.137636	0.5927998
Hebp2	-0.6475404	0.8757657	0.1377473	0.5927998
Tspyl2	0.2435145	4.1193786	0.1377483	0.5927998
Snx8	0.2767772	4.4419027	0.1377744	0.5927998
Hbegf	0.5663932	0.6637288	0.1377765	0.5927998
Gm39860	0.5525131	0.5034498	0.1378143	0.5927998
Syne3	-0.2743019	4.8876131	0.1379326	0.5931445
Lgals3	-0.2451749	7.3434386	0.1379856	0.5932083
Nlk	-0.224512	5.5626804	0.1380433	0.5932211
Hmmr	-0.2065709	7.2466381	0.1381414	0.5932211
Hax1	0.2018962	5.2790513	0.138146	0.5932211
Ddx10	0.1985266	5.2778844	0.1381571	0.5932211
Slc18a1	-0.8358076	-0.1056433	0.1382417	0.5932211
Drg2	0.206938	4.3741593	0.1382564	0.5932211
Tbc1d24	-0.195451	5.1388402	0.138293	0.5932211
Fgfr1op2	0.2028541	7.3716422	0.1382937	0.5932211
Slc18a2	-0.3189395	3.1724609	0.1383507	0.5933021
Sgf29	0.2403398	3.8556291	0.1384419	0.5933757
Bag2	0.2521233	4.1401261	0.1384442	0.5933757
BC028528	-0.2853079	3.7297283	0.1385625	0.5934345
Rrp36	0.2291156	3.9754889	0.1385761	0.5934345
Gramd2	-0.441759	2.1642924	0.1386033	0.5934345
Sox4	0.321313	6.3536578	0.1386176	0.5934345
Osgin2	0.2626542	3.4308899	0.1386974	0.5934345
Gm5487	0.5146903	0.94694	0.1387497	0.5934345
Dock8	-0.2590156	7.7621481	0.1387525	0.5934345
Pik3r2	0.2181926	5.065577	0.1387631	0.5934345
Mpdu1	0.2149789	4.6845433	0.138829	0.5935449
Got2-ps1	0.6765411	0.1519406	0.138885	0.5935449
Nr6a1	0.4910385	1.000781	0.1389034	0.5935449
Cage1	0.6408908	0.2007104	0.1392279	0.5945701
Greb1l	-0.8389344	0.1116531	0.1393233	0.5945701
Slfn2	-0.2861525	7.4363233	0.1393288	0.5945701
Gm3788	0.3009815	2.9929567	0.1393692	0.5945701
Slc12a7	0.2447424	4.2469443	0.1393707	0.5945701
E2f4	0.2148221	7.4086831	0.1393966	0.5945701
Ncmap	-0.946453	-0.6208027	0.1394645	0.5945701
Slc25a22	0.2283497	3.6201993	0.1395003	0.5945701
Gm9861	0.8623795	1.1314067	0.1395214	0.5945701
Atp8a1	-0.2055752	7.4625563	0.1395601	0.5945701
Gm8730	-1.2922159	5.5534294	0.1396068	0.5945701
Snhg7	0.513335	2.1149111	0.1396341	0.5945701

Hipk3	-0.1647435	7.1133579	0.1396403	0.5945701
Dxo	0.2309806	3.6793566	0.1398037	0.5947842
Dexi	0.3767699	2.7695371	0.1398058	0.5947842
Elob	0.2025235	6.675665	0.1398104	0.5947842
Samd14	0.2292988	7.0067051	0.1398435	0.5947842
Sh2d4a	0.3015474	4.1792807	0.1399289	0.5949849
Smg1	-0.2215021	7.2689801	0.1399981	0.5951162
Rpl7a	0.2143906	9.6233523	0.1400377	0.5951217
Mrpl38	0.2033362	4.6833962	0.1401297	0.5953503
Ankrd13d	0.4255391	2.4889826	0.1402646	0.5956142
Notch1	-0.2660872	6.231568	0.1402842	0.5956142
Kcnk2	0.4852103	3.3878026	0.1403067	0.5956142
Cep350	-0.2474053	7.0747607	0.1403516	0.5956421
B3gat3	0.2008612	5.2870578	0.140429	0.5958083
Rps23	0.2360346	9.8045617	0.1406394	0.5965253
Bach1	-0.1599156	6.4037229	0.1406747	0.5965253
Ten1	0.2920645	3.9320898	0.1407418	0.596647
Micos10	0.2345753	6.9168026	0.1409998	0.5971178
Vps25	0.1545983	5.9936204	0.1410285	0.5971178
Doc2g	0.6780188	0.4308526	0.1410556	0.5971178
Clca3a1	-0.2294332	5.1025552	0.1410644	0.5971178
Mrpl47	0.2777018	3.7868055	0.1410662	0.5971178
Ibtk	0.2289362	6.9646376	0.1410849	0.5971178
Rtraf	0.2431289	6.4790032	0.1411216	0.5971178
Ccdc22	0.2011929	4.6546527	0.1412122	0.5973388
Tmem163	0.4228844	1.8418173	0.1413211	0.5975372
Tkt	-0.2207549	9.047783	0.1413457	0.5975372
Ptprr	-0.5788889	0.3721643	0.1413744	0.5975372
Hspb6	-0.4121757	3.4877764	0.1415402	0.5980756
Atic	0.1972202	6.167922	0.1415927	0.5980832
Shroom3	0.4900173	1.825412	0.1416476	0.5980832
Ptpn14	-0.2851588	3.9315222	0.1416765	0.5980832
Snx17	0.1648954	5.6437843	0.1417295	0.5980832
2310061I04Rik	0.22708	4.0701919	0.1417343	0.5980832
Coasy	0.2214135	4.1508246	0.1417942	0.5981282
Cpa3	-0.2854536	3.6039092	0.1418435	0.5981282
Arhgap17	0.2169589	5.8242035	0.1419891	0.5981282
Irs2	-0.2013574	5.4522089	0.1419946	0.5981282
Ophn1	-0.2937961	3.9221248	0.1420021	0.5981282
Armc5	0.2413266	4.2924301	0.1420089	0.5981282
Ap2a1	0.2261977	6.3692365	0.1420141	0.5981282
Calhm6	0.5364485	1.0429325	0.142148	0.5985302
H3f3b	0.2035622	9.4850877	0.1422535	0.5988121

Ctps	0.2174579	5.3171362	0.1423126	0.598899
Ctr9	-0.1578961	5.8837952	0.1423724	0.5989724
Rplp1	0.2544514	10.413981	0.1424367	0.5989724
Galnt6	0.2864403	4.6374923	0.1424737	0.5989724
Nfil3	-0.3656201	3.8427923	0.1424841	0.5989724
Zfp418	-0.5256126	0.5251227	0.1426474	0.599489
Lbp	-0.2743737	5.7967352	0.1427152	0.599489
Cd38	0.2576728	5.2533734	0.1427226	0.599489
Gm33786	0.4431273	0.7897369	0.142895	0.6000509
Prf1	-0.7061614	0.0957736	0.1429343	0.600054
Dhx57	0.2539284	4.4191786	0.1430149	0.6002099
Dhodh	0.2795011	3.1999981	0.1430734	0.6002099
Gm32026	-0.458647	2.9487119	0.1430872	0.6002099
Rpl24	0.2195647	8.6526018	0.1432537	0.6007465
Slfn8	-0.2948847	4.0414503	0.1434045	0.6012166
Tet1	-0.4100748	3.0146619	0.143533	0.6015932
Gm6035	0.8225234	-0.0511389	0.1436587	0.6019502
Mtmr12	-0.1910742	5.434032	0.1437545	0.6019502
Hoxa11	0.7592164	-0.5037205	0.1437562	0.6019502
Mtmr1	-0.1979864	5.231659	0.143794	0.6019502
Kctd14	0.3942549	3.8812393	0.1438117	0.6019502
Iars2	0.1886277	5.9360652	0.1439577	0.6023991
Fmr1os	-1.1637813	-1.0016261	0.1442728	0.6033249
Trio	-0.2340052	5.0096342	0.1443366	0.6033249
Trip4	-0.2184126	5.156388	0.1443419	0.6033249
Atxn1	-0.2410947	4.0385471	0.1444875	0.6033249
Zcrb1	0.2210578	5.3360708	0.1445244	0.6033249
Ncaph	0.2084783	6.5272324	0.1445264	0.6033249
Rasa1	-0.181157	5.9818169	0.144535	0.6033249
Rpl17	0.2579674	9.8352623	0.1445508	0.6033249
Ccdc88a	-0.1843528	6.1430615	0.1445587	0.6033249
Sapcd2	0.2810314	3.3144752	0.1445668	0.6033249
Ceacam2	-0.4076971	5.2672928	0.1446326	0.6033256
Med18	0.2876965	3.0080865	0.1446623	0.6033256
Rps3a1	0.2661824	10.571076	0.1446833	0.6033256
Ccrl2	0.2910752	3.9938407	0.1447629	0.6034955
Gm16214	0.8658776	-0.7574509	0.144831	0.6035285
Clasrp	0.2262482	5.0241628	0.1448484	0.6035285
Sytl4	-0.3345028	3.9695261	0.145026	0.6037909
Klc3	0.9533909	-0.7015585	0.1450651	0.6037909
H2bc4	0.3782465	4.5692529	0.1450778	0.6037909
Susd3	0.3126205	3.0242732	0.1451237	0.6037909
Psmb2	0.1645818	6.2889282	0.1451647	0.6037909

Gm5620	0.3903283	1.4958167	0.1451874	0.6037909
Vcan	0.5593651	4.1916012	0.1453145	0.6037909
Cat	0.2008008	7.5893474	0.1453458	0.6037909
Sowahc	-0.2485891	4.138849	0.145362	0.6037909
Pmm1	0.2578999	4.0416121	0.1454085	0.6037909
Cyba	-0.2123323	7.6956833	0.14542	0.6037909
Ddx6	-0.1725819	8.600083	0.1455727	0.6037909
Bok	0.5047324	1.5307365	0.1455787	0.6037909
Eif3f	0.1726966	7.4579317	0.1456203	0.6037909
Ndufb8	0.1669912	6.6570164	0.1456741	0.6037909
Kif20b	-0.2145227	6.5873528	0.145676	0.6037909
Leng9	0.5654219	1.6203592	0.1456862	0.6037909
Fam222a	1.0611512	-0.6533999	0.1457454	0.6037909
Ldlr	-0.4661961	4.4031093	0.1458061	0.6037909
Ssc4d	-0.5676908	0.9882941	0.1458795	0.6037909
Mcm4	0.2037037	8.0635603	0.1458911	0.6037909
Zfp955a	-0.2220239	3.9769667	0.1459174	0.6037909
Prkaa1	-0.1530257	6.3573042	0.1459245	0.6037909
Cldn13	0.2949701	6.9121855	0.1459254	0.6037909
Serpina3h	-1.5130958	0.8015605	0.1459984	0.6037909
Sync	-0.8452583	-1.0338683	0.1460042	0.6037909
Chfr	0.1686954	6.1309587	0.1460282	0.6037909
Mogat2	-0.3855112	3.5222062	0.1460454	0.6037909
Mpc1	-0.2558376	4.7386967	0.1460651	0.6037909
Pot1a	-0.2169345	5.0221565	0.1460816	0.6037909
Tnfrsf4	-0.5961837	-0.5228425	0.1461317	0.6037909
Tbl3	0.2384829	4.666828	0.1461536	0.6037909
Rell2	0.7847929	-0.667359	0.1462265	0.6038743
Setd5	-0.1924303	6.9360277	0.1462514	0.6038743
Gm3219	0.627951	0.0613704	0.1463011	0.6038822
Wrap53	0.2501557	3.962799	0.146331	0.6038822
Krt80	-0.5408866	2.9476192	0.1465046	0.6043078
Gm14853	-0.4166343	1.8845589	0.1466076	0.6043078
Nsdhl	-0.2805772	3.8160683	0.1466095	0.6043078
Vat1	-0.2115729	5.9858628	0.1466198	0.6043078
Srebf2	-0.193513	6.4353127	0.1466305	0.6043078
Mapk14	-0.2096879	7.4113398	0.1467047	0.6043078
Qsox1	-0.1852736	6.1415418	0.1467061	0.6043078
Gm32029	-0.6449452	-0.0759603	0.1467473	0.6043176
Nat8f1	-0.4523714	0.9047576	0.1468061	0.604355
Pprc1	0.2181308	5.6564589	0.1468425	0.604355
Stradb	0.2426448	6.2023243	0.1468869	0.604355
Actr3b	0.6637171	0.251749	0.1469118	0.604355

Rhno1	0.1844398	5.7624734	0.1470327	0.6046924
Gmeb2	0.206683	5.063775	0.1472662	0.6054488
Fbxo11	-0.2132678	6.1451588	0.1473199	0.6054488
Zfp758	-0.1841536	4.5863892	0.1474354	0.6054488
Rest	-0.1984064	6.0603528	0.1474635	0.6054488
Cenpn	0.2212945	5.1552627	0.1475397	0.6054488
Psme2	0.2472379	6.1969545	0.1475723	0.6054488
Gimap1	0.3459259	4.3953691	0.1475877	0.6054488
Asb16	-0.8340706	-0.7263402	0.1476948	0.6054488
Plin5	-0.8565234	-0.6172029	0.1477031	0.6054488
Oxa1l	0.1785442	5.6796033	0.1477417	0.6054488
Clpp	0.2471618	3.9934093	0.1477631	0.6054488
Gm14276	0.4701277	1.1399817	0.147781	0.6054488
LOC118567733	-0.4875631	2.4692529	0.1478449	0.6054488
Gstp-ps	-0.6250466	-0.2154686	0.1478693	0.6054488
H2-DMb2	0.4619202	4.2018594	0.1479142	0.6054488
Zfp251	0.2485035	3.6161161	0.1479268	0.6054488
Ptpn11	-0.1988338	7.6440423	0.1479282	0.6054488
Ccdc40	-0.4802194	0.5868055	0.1479775	0.6054488
Dancr	0.40367	2.3223882	0.1479806	0.6054488
Pelo	0.2250931	4.3921814	0.1479951	0.6054488
C1qtnf7	0.8923085	-0.3758266	0.1480667	0.6055821
Ilkap	0.1935423	5.727752	0.1481356	0.6056835
Snrpd1	0.2710024	7.0464497	0.1481693	0.6056835
Trmt11	0.323102	3.6979217	0.148261	0.6058991
Dcn	0.5492228	8.1495909	0.148391	0.606271
Bcat1	0.4850059	2.5731183	0.148561	0.606806
Gm31493	0.4925312	1.2553571	0.1486442	0.6069028
Spty2d1	-0.1752516	5.9427108	0.1486743	0.6069028
St8sia4	-0.2411446	6.29704	0.1487029	0.6069028
Gtf3c5	0.2227056	4.4529754	0.1487407	0.6069028
Spg11	-0.2287984	4.4986095	0.1489664	0.6073664
Foxred2	-0.3880057	3.2780934	0.1489853	0.6073664
Peak1	-0.1998262	5.0129322	0.1490028	0.6073664
Tmem184c	0.1982772	4.2010768	0.1490389	0.6073664
Dennd3	-0.2361669	4.2674013	0.1490734	0.6073664
Zkscan16	0.9894937	0.6812341	0.1490887	0.6073664
Dnajc18	0.2239677	3.8213994	0.1491345	0.6073941
B4galt2	0.5845594	2.1335416	0.1492179	0.6075747
Seh1l	0.1605177	6.3094047	0.1494247	0.6081195
LOC118567336	0.6218104	0.1450691	0.1494558	0.6081195
Chchd7	0.2837284	4.1244968	0.1495003	0.6081195
Abcc3	-0.3945008	4.4568055	0.1495224	0.6081195

Gm9776	-0.4807342	0.7559125	0.1495649	0.6081195
Flt3	-0.3465894	3.4264673	0.1495863	0.6081195
Gmppb	0.2533152	3.7342646	0.1496958	0.6084055
Antxr2	-0.2456736	6.1775538	0.1497545	0.6084854
Jtb	0.2064064	5.6271011	0.1498222	0.6085948
Zfp160	0.2018066	4.772987	0.1498912	0.6085948
Svbp	0.3072278	5.1647202	0.1498989	0.6085948
Rab3ip	0.2804966	3.1584216	0.1500471	0.6085958
A930013F10Rik	-0.5154843	1.1249812	0.1500651	0.6085958
Eif1ad	0.1891362	5.546714	0.1500668	0.6085958
Trim33	-0.1831575	6.2704635	0.1501031	0.6085958
Zmiz2	0.1539087	6.2898166	0.1501555	0.6085958
Mrps10	0.2729042	3.9547577	0.150178	0.6085958
Itpkb	-0.2059705	6.1151035	0.1501911	0.6085958
Nes	-0.5215771	2.3780451	0.1502121	0.6085958
Scyl2	-0.1994297	5.9026096	0.1502912	0.6086637
Serf1	0.3884418	3.4351028	0.1503071	0.6086637
Bcas3	0.2045897	4.6664657	0.1503603	0.6087205
Gm33887	-0.4324203	3.6336116	0.1504135	0.6087774
Elmod3	0.2950414	3.1073351	0.1505807	0.6088889
Ptprz1	-0.5582224	4.2564053	0.1506149	0.6088889
Milr1	-0.2336228	4.7210156	0.1506472	0.6088889
Col2a1	-0.6160136	9.020842	0.1506874	0.6088889
Zswim5	-0.5929575	0.7635052	0.1507027	0.6088889
Gm26740	-0.2441271	4.6868481	0.1507064	0.6088889
Adora3	-0.6957059	0.1128177	0.1507151	0.6088889
Zmynd15	0.7542093	-0.7494982	0.150817	0.6091424
Tmem199	0.2495979	3.3013655	0.1508945	0.6092368
Slc14a2	-0.5697607	-0.1835466	0.1509187	0.6092368
Pakap	0.2639251	4.3521038	0.1510337	0.6093427
Prdm1	-0.3283014	3.3957245	0.1510937	0.6093427
Smarcb1	0.1753691	5.4796721	0.1510986	0.6093427
Rccd1	0.4002821	2.2852192	0.1511016	0.6093427
Klf12	-0.6422214	2.805481	0.1512168	0.6094828
Adrb2	0.3226679	5.2996081	0.1512209	0.6094828
Rps15	0.2209845	9.6688812	0.1512539	0.6094828
Eif4a-ps4	0.2759503	3.799553	0.1514263	0.610004
Card19	-0.2068262	4.5307209	0.1514679	0.610004
Gm38852	0.9085484	-0.31876	0.1515009	0.610004
Zfp112	0.6089931	-0.1557333	0.1515541	0.6100603
Gm34223	-0.7919983	-0.9100898	0.1516405	0.6102502
Spata2l	-0.3278353	1.8267104	0.151794	0.6103846
Ccnyl1	-0.2630323	3.6469944	0.1518404	0.6103846

Als2	-0.2261146	4.0565964	0.151852	0.6103846
Cebpa	0.2004351	6.2390066	0.1518541	0.6103846
Camk4	-0.8014015	0.3202858	0.1519051	0.6103846
Cpz	0.5577852	4.853165	0.1519268	0.6103846
Ccdc93	-0.1916167	5.1325498	0.1519486	0.6103846
Ipo11-lrrc70	-0.6066982	0.7048545	0.1521078	0.6108663
Rps19-ps6	0.3737828	4.6209573	0.1521766	0.6109848
Hacd3	0.1957934	5.5636729	0.152346	0.6111945
Crispld1	-0.4562342	2.9561063	0.1523794	0.6111945
Dtx3l	-0.2350218	5.3912718	0.1523841	0.6111945
Gm42067	0.5643712	0.8085983	0.152386	0.6111945
Otud5	0.2259281	7.5911003	0.152454	0.6113095
Slc25a37	0.3193582	10.282877	0.1525052	0.6113349
Epb41l4aos	0.3989812	3.1069188	0.1525389	0.6113349
Nprl3	0.2541408	4.3036568	0.1526193	0.6113363
Slc26a2	-0.3271461	3.6405845	0.1526611	0.6113363
Gm13889	0.4298885	1.6340588	0.1527077	0.6113363
Gnl3	0.2192696	6.0664052	0.15271	0.6113363
Pip4p2	0.2335727	4.5964432	0.152791	0.6113363
Mgat4b	0.1806592	5.0566962	0.1528261	0.6113363
Il6ra	-0.3755265	5.4947998	0.1528503	0.6113363
Itga8	-0.4496714	1.462082	0.1528742	0.6113363
Tlcd4	0.2575261	6.2459591	0.1529716	0.6113363
Copb1	-0.1643053	6.7851684	0.1530275	0.6113363
Rack1	0.1977709	9.4873142	0.153028	0.6113363
Samsn1	-0.2188415	5.855788	0.153082	0.6113363
Isg20l2	0.1711987	5.8761038	0.153092	0.6113363
Sufu	0.2538229	4.5765675	0.1531021	0.6113363
Pacsin2	0.1857513	7.2351366	0.1531558	0.6113363
Wdr48	-0.170638	6.1894752	0.1531998	0.6113363
Capns2	-0.7578583	-0.6307536	0.1532367	0.6113363
Tmsb15l	-0.612231	0.4709801	0.1532468	0.6113363
Lpgat1	-0.2575591	6.6042521	0.153331	0.6113898
Cdk10	0.2490058	3.3437493	0.1533388	0.6113898
Ust	-0.4568727	3.4753649	0.1534579	0.6116162
Rps12	0.2953106	10.295089	0.1534742	0.6116162
Pth1r	-0.4221496	5.6979341	0.1537117	0.6121001
Gpsm2	0.2208254	6.5257802	0.153729	0.6121001
Bcl10	-0.1976766	5.5166278	0.153731	0.6121001
Ube2o	0.3097413	7.5544459	0.1537579	0.6121001
Zbtb40	0.2492291	3.7376205	0.1538691	0.6121001
Gm4204	0.2124556	5.0058002	0.1538693	0.6121001
Arhgap5	-0.2440347	4.8625485	0.1538711	0.6121001

Pcdhb20	-0.6022565	0.5274099	0.1540585	0.6125624
Ngf	0.6341148	-0.1538439	0.1540697	0.6125624
Ap3m1	-0.1597201	6.2236706	0.1541055	0.6125624
Gm11696	0.4263084	1.4808991	0.1543985	0.6135373
Slc22a13b	0.7771807	-0.86985	0.1544425	0.6135373
Timp2	-0.2676407	7.1159375	0.1544691	0.6135373
Snx5	0.1781344	7.415193	0.1545264	0.6136083
Tsnax	0.1481862	5.7580453	0.1546458	0.6138929
Clec3a	-1.1464313	3.0022722	0.154677	0.6138929
Camk2g	-0.2073462	4.8182343	0.1548116	0.6142703
Notch2	-0.1613986	6.8430827	0.1549239	0.6144306
Rpl3	0.2009807	8.9567271	0.1549338	0.6144306
Lyrm4	0.3499198	3.8657392	0.1549705	0.6144306
F3	0.736394	0.3998872	0.1550637	0.6146434
Ppp1r8	0.1634839	5.5487053	0.1551697	0.614907
Gm7367	-0.4471907	0.8308239	0.155357	0.615062
4833447I15Rik	-0.5913834	0.3071824	0.1553665	0.615062
Mrps25	0.2085349	4.6419278	0.1554063	0.615062
Tmem250-ps	0.1734741	5.4688875	0.1554742	0.615062
Wdr55	0.2529174	4.4645813	0.1554752	0.615062
Pop1	0.3527118	3.0663223	0.1555098	0.615062
Prrt1	0.7590693	0.0193657	0.1555167	0.615062
Ear-ps9	-1.841691	2.3454089	0.1555252	0.615062
Zfp146	0.1889435	6.0494341	0.1556354	0.6152292
Poglut3	0.3142649	3.7301778	0.1556466	0.6152292
Tspan5	-0.2997824	4.4438035	0.1557832	0.6152876
Fpgs	0.2898906	4.0403557	0.155858	0.6152876
Gm6560	-0.3449825	1.8867486	0.1558614	0.6152876
Ttc4	0.2185053	4.9033432	0.1558875	0.6152876
Bloc1s4	0.2295695	4.055458	0.1559146	0.6152876
Glt28d2	0.6480588	0.0581345	0.1559321	0.6152876
Ctdspl2	-0.1621284	6.311939	0.1559549	0.6152876
Gm32908	-0.4138518	2.1882335	0.1559778	0.6152876
Arhgap32	-0.2919428	3.0674682	0.1560814	0.6153701
Slc30a5	0.1572717	6.4020702	0.1560946	0.6153701
Lats1	-0.150589	5.6668853	0.1561174	0.6153701
Bicc1	-0.2943855	4.9693728	0.1562505	0.615485
Pcyt2	0.2527384	4.4547769	0.1562738	0.615485
Pcdhb11	-0.7642729	-0.5361733	0.1563034	0.615485
Postn	-0.5907983	6.634762	0.1563174	0.615485
C3ar1	0.443928	2.4575862	0.1563793	0.615485
B3gat2	0.6722457	0.2019461	0.156384	0.615485
Capn5	0.3317541	5.2172147	0.1564728	0.6156788

Eif2b3	0.2792558	4.2674429	0.1566644	0.6157455
Mpl	-0.2999789	5.2615138	0.156681	0.6157455
Lingo3	-0.806129	0.0115455	0.1566926	0.6157455
Ppwd1	0.2250391	5.3700405	0.1567139	0.6157455
Igkv5-39	-0.6578909	2.9143064	0.1567248	0.6157455
Tmem9b	-0.1530392	5.5454671	0.1567273	0.6157455
Srsf7	0.1825908	7.5079746	0.1567821	0.6158051
Olfml2a	-0.4807256	2.6671575	0.157021	0.6160903
Cracr2a	-0.2069086	4.3864866	0.157041	0.6160903
Tmem129	0.1978777	4.4465601	0.1570437	0.6160903
Spry2	0.3049641	2.9385992	0.1570606	0.6160903
Homer2	-0.5442945	1.977116	0.1571136	0.6160903
Micu2	-0.1719604	6.330501	0.157117	0.6160903
Tlr12	-0.3931224	1.5903686	0.157132	0.6160903
Arl4c	0.2369614	4.3948203	0.1572424	0.616274
Smim11	0.2456064	4.7464149	0.157258	0.616274
Mboat2	0.3866433	5.2642226	0.1573383	0.6164331
Tgif2	0.3505578	2.3951903	0.1575428	0.6168403
Nop56	0.2295023	6.9185542	0.1575467	0.6168403
Cs	0.1582878	7.5161768	0.1575612	0.6168403
Uhrf1bp1	0.2547815	4.4891682	0.1576469	0.6168553
Slc25a1	0.1656342	4.9319012	0.15767	0.6168553
Klhdc10	-0.1700194	5.9679988	0.157684	0.6168553
Zbtb39	-0.2163682	4.1009661	0.1577522	0.6169495
Setd1b	-0.2308405	6.727373	0.1578091	0.6169495
Naa40	0.1718854	5.7734529	0.1578441	0.6169495
Dus2	0.287072	3.2702845	0.1579514	0.6169495
Ddx20	0.2157522	4.8840546	0.1579677	0.6169495
Telo2	0.2349744	3.3626051	0.1579787	0.6169495
Srsf3-ps	-0.9442499	-0.291197	0.1579857	0.6169495
Actr3	-0.198913	9.5729771	0.1580769	0.6169681
Parp4	-0.2360581	5.4098702	0.1581198	0.6169681
Mei4	1.0042029	-0.5456462	0.1581367	0.6169681
Ermap	0.2672765	9.0381203	0.1581492	0.6169681
Dcaf8	0.1617498	7.0237695	0.1582378	0.6171592
Cp	-0.2521526	6.8165516	0.1582856	0.6171908
Frg2f1	-0.744367	-0.3663585	0.1583839	0.617383
Scand1	0.218262	6.0202067	0.1584212	0.617383
Mrps30	0.1880987	4.7688798	0.158454	0.617383
Snrnp48	0.2440624	4.7427382	0.1585133	0.617452
Rps28	0.2384003	8.7634705	0.1585897	0.617452
Sox13	-0.2830031	4.2171877	0.1586341	0.617452
Babam1	0.1634109	5.7776617	0.1586683	0.617452

Surf6	0.1924465	4.6047594	0.1587063	0.617452
Gpr12	1.53252	-0.6823816	0.1587389	0.617452
Myb	0.2309885	8.0543092	0.1587816	0.617452
Gpc6	0.3778067	3.5182948	0.1587893	0.617452
Arhgap25	-0.190064	5.9633787	0.1589263	0.6176615
Zfp382	-0.2288495	4.1488892	0.1589622	0.6176615
Foxm1	0.188152	6.3488527	0.1589623	0.6176615
Sorcs2	0.3839217	2.7137346	0.1590599	0.6178864
Cenpx	0.2455143	5.1487538	0.1591081	0.6179194
BC003965	0.2546234	4.4217051	0.1592902	0.6183315
9430038I01Rik	0.2967002	2.3133182	0.1592937	0.6183315
Bvht	0.6760125	0.7604803	0.1594627	0.6183406
Actg1	-0.2164697	9.6627812	0.1594742	0.6183406
Rhov	0.4892039	1.2805368	0.1594884	0.6183406
Dusp16	-0.2847474	2.9545313	0.1595676	0.6183406
Abat	0.2611498	3.0928834	0.1595737	0.6183406
Abcb4	0.2453359	7.0577269	0.1595852	0.6183406
Dnajb2	0.2941076	6.3500948	0.159651	0.6183406
Taf8	0.1596708	5.3506092	0.1596531	0.6183406
Cgas	-0.2814537	3.7640602	0.1596589	0.6183406
Ago2	-0.287737	7.4532766	0.1597066	0.6183406
Neurl4	0.1730433	5.4155263	0.159751	0.6183406
Wdr19	-0.4027118	1.8913568	0.1597731	0.6183406
Rpl26	1.1005204	8.0930532	0.1598149	0.6183483
Adi1	0.2101107	4.656612	0.1598847	0.618438
Hras	0.2042399	5.3907448	0.1599175	0.618438
Smg5	0.1708881	6.4316378	0.1600631	0.6188471
2410002F23Rik	0.2429513	4.5576248	0.1602183	0.6192467
Pabpc1l	0.4774403	1.3543835	0.1602794	0.6192467
Rps8	0.2237161	9.7562022	0.1603223	0.6192467
Zfp580	0.6078458	0.9519378	0.1603393	0.6192467
Gm20506	0.5675569	1.6757747	0.1603949	0.6192467
Abcb1b	-0.3217445	2.7209555	0.1604336	0.6192467
Mmp2	0.4884657	6.3375336	0.1604452	0.6192467
Sfmbt2	-0.522779	1.6175717	0.1605223	0.6192786
Gm5805	0.5746521	1.7190442	0.1605331	0.6192786
Rbpj-ps3	-0.9342328	-0.6393631	0.1606012	0.6193879
Myc	0.1951936	6.1738191	0.1606481	0.6194151
Ric3	-0.7523039	0.4402537	0.1607535	0.6194558
Nfyb	0.1727906	5.9123075	0.1608427	0.6194558
Gm12250	-0.281777	2.7984279	0.1609105	0.6194558
Tbxa2r	-0.4114449	2.6123976	0.1609257	0.6194558
Txnrd2	0.2922309	5.4896394	0.1609315	0.6194558

Tmem14c	0.2549833	7.9781024	0.1609722	0.6194558
Ssbp1	0.2096586	5.2838132	0.160985	0.6194558
5830428M24Rik	-0.5981967	0.7230991	0.1609858	0.6194558
Cald1	-0.1795055	6.7667679	0.1610171	0.6194558
Lhx1	-0.5501241	1.5570526	0.1612099	0.6195342
Pif1	0.2176525	4.9570792	0.1612121	0.6195342
Zfp229	0.2986255	2.500708	0.1612336	0.6195342
Psd	0.7148274	0.530505	0.1612436	0.6195342
Pop5	0.2546023	4.383577	0.1613616	0.6195342
Hsd17b12	-0.1666684	5.4048512	0.161373	0.6195342
2600006K01Rik	0.3529997	2.1928219	0.1614652	0.6195342
Apoc2	0.5859106	-0.1804961	0.1614967	0.6195342
Tap1	0.1671389	5.5873499	0.1615039	0.6195342
Rab28	-0.2007875	5.2377585	0.1615205	0.6195342
Slx1b	0.3213281	2.651117	0.1616457	0.6195342
Tcf12	0.2022991	6.4292916	0.1616934	0.6195342
Mta1	0.186652	5.8703278	0.1617026	0.6195342
Lnx1	-0.872484	-0.2494241	0.1617079	0.6195342
Map2	0.7306364	-0.7853817	0.1617286	0.6195342
Emx2	0.8487104	-1.3076641	0.1617477	0.6195342
Tmem26	-0.35133	3.3537024	0.1617491	0.6195342
Gm12906	0.398575	2.5249976	0.1617551	0.6195342
Iqgap2	-0.3311747	6.6593729	0.1618196	0.6195342
Gm41658	0.7042197	-0.0244903	0.1618341	0.6195342
3425401B19Rik	-1.0137191	-0.436035	0.1618849	0.6195761
9330175E14Rik	0.7084336	-0.2040063	0.1619937	0.6198403
Gpr173	0.8707621	-0.8394131	0.1620689	0.619917
Ilk	-0.1723419	7.7126748	0.1620935	0.619917
Gja1	0.4296801	8.1864879	0.1622026	0.6200327
Gm13461	0.4855553	1.1681377	0.1622213	0.6200327
Setdb1	0.1822404	5.4805725	0.1622434	0.6200327
Tbc1d15	-0.1592236	6.4108916	0.1625301	0.6209758
Drosha	0.1822907	5.6282452	0.1625703	0.620977
Med1	-0.2423127	6.5472695	0.1627073	0.6212105
Kcnq1ot1	0.3165656	3.1879021	0.1627113	0.6212105
Hspa2	0.3503428	3.5288123	0.1628322	0.6215194
Phax	0.1944074	5.0442339	0.162966	0.6216281
Appl1	-0.1953118	5.5193524	0.1629717	0.6216281
Rasa3	-0.1844089	7.7881015	0.1629865	0.6216281
Zwilch	0.2016274	5.3427882	0.1630205	0.6216281
Nt5c	0.2067906	5.2799817	0.1630642	0.6216385
Anxa5	-0.195889	7.1290591	0.1631032	0.6216385
Hebp1	0.2839497	7.3606881	0.163145	0.6216457

Mlec	-0.1915021	8.4791853	0.1632697	0.6219462
Gstm2	0.2860073	3.2784056	0.1633361	0.6219462
Fmn2	0.7725046	1.1213311	0.163395	0.6219462
2010204K13Rik	0.6428984	0.4635562	0.1634575	0.6219462
Arhgef2	-0.1988279	6.6505026	0.1635063	0.6219462
Gstm7	-0.6935957	0.1649794	0.1635106	0.6219462
Cpsf7	0.1739495	6.5750197	0.1635529	0.6219462
Tgfbr1	-0.2186728	6.7052966	0.1635629	0.6219462
Cpne8	-0.4786276	1.6713059	0.1635838	0.6219462
Tmem119	0.4546275	5.55222	0.1639159	0.6230089
Ppm1h	-0.2035336	4.6001785	0.1639434	0.6230089
Cd81	-0.2198726	6.263075	0.1640393	0.623221
F8a	0.3181185	2.7407016	0.1641364	0.6234376
Aspa	-0.9278148	-0.8280311	0.1643652	0.6241542
9430037G07Rik	-0.928483	-0.148787	0.1644501	0.6242386
Gm5560	0.5983154	-0.1407043	0.1644721	0.6242386
Xrcc3	0.2615571	2.971936	0.1645078	0.6242386
Lat	-0.2374135	4.3902714	0.1645551	0.6242657
Rcbtb1	0.1993158	4.7534267	0.164733	0.6247884
Kdm5a	-0.1724196	6.6447279	0.1649201	0.6251943
Serpina11	-1.31179	-0.8676716	0.1649204	0.6251943
Spi1	-0.1834445	7.4065314	0.1649693	0.6252273
Tcp11l1	-0.3196571	2.9482999	0.1650282	0.6252979
BE692007	0.5630951	3.2891173	0.1651681	0.6255793
Sbds	0.1733604	5.9945317	0.1651829	0.6255793
Ephb1	-0.7323761	-0.9211392	0.1653656	0.6261188
Gla	-0.2767575	3.5312936	0.1654157	0.6261217
Gmpr	-0.3214512	3.952295	0.1654524	0.6261217
Ctcf	0.1595552	7.7118325	0.1654871	0.6261217
Gm9836	-0.7134965	-0.423681	0.1655608	0.6262484
Gm31852	-0.7086306	-0.4326338	0.1656455	0.6263881
Prps1	0.189901	5.5548638	0.1656783	0.6263881
Cnot11	0.1609964	5.1733064	0.1657728	0.6264471
Rida	-0.2573611	5.2682159	0.1657745	0.6264471
Puf60	0.1410081	6.6379052	0.1659231	0.6268565
Tsn	0.1498314	6.7031828	0.166029	0.626985
Tceal3	0.6787453	0.6718217	0.1660378	0.626985
Ppp5c	0.1642273	5.4139742	0.166188	0.6273375
Zfp36l1	0.1960792	7.0372194	0.1662682	0.6273375
Alyref2	0.2972297	2.6990807	0.1662965	0.6273375
Cfap54	0.5210822	0.3915838	0.1663285	0.6273375
Gm16867	1.7681094	2.218557	0.16636	0.6273375
Dll1	-0.3699842	1.4002247	0.1663873	0.6273375

Cep57l1	0.2486009	3.873687	0.1664134	0.6273375
Bsdc1	0.2437348	7.1018299	0.1664816	0.6274291
L3mbtl2	0.2034049	4.6329479	0.1665215	0.6274291
Gm11690	0.7619843	1.5142911	0.1665588	0.6274291
Rras2	0.3548478	4.1792629	0.1666863	0.6274742
Cdkn2c	0.2124173	6.530837	0.1666886	0.6274742
6820431F20Rik	0.3970208	2.1954414	0.1666918	0.6274742
Cnih1	0.202773	5.6515659	0.1668695	0.6279167
Nphp1	0.3085252	2.8950751	0.1669005	0.6279167
Icam4	0.3313846	4.623156	0.1669304	0.6279167
Dctd	0.3422144	2.7499027	0.1670718	0.6282966
Heatr5a	-0.1671672	6.0816713	0.167213	0.6285144
Ascc2	0.2212512	5.1361779	0.1672265	0.6285144
Noc4l	0.2165509	4.5504534	0.167251	0.6285144
Ift81	-0.4770459	1.6804261	0.1673186	0.6286167
Nacad	0.8256624	0.0660038	0.1673622	0.6286287
Nomo1	-0.1943355	6.8826884	0.1674495	0.6288048
Secisbp2l	-0.1537475	6.0213305	0.1676399	0.6293678
Tmem88	0.5301399	0.3471206	0.1677216	0.6295228
Arhgef11	-0.2418144	4.9984644	0.1677793	0.6295505
Dok3	0.2077731	6.9962092	0.1678556	0.6295505
Prxl2a	0.2995788	6.9100462	0.1678755	0.6295505
Rtkn	-0.4100645	1.3145741	0.1679217	0.6295505
Rpap3	0.2588417	3.8430979	0.1679498	0.6295505
Plp2	-0.2208095	6.7005858	0.1679719	0.6295505
Hsf1	0.1774255	4.5314703	0.1681473	0.6298402
Gm10698	-0.4785426	3.583059	0.1681645	0.6298402
Pyurf	0.2304395	4.1312744	0.168188	0.6298402
Eif5al3-ps	0.5459374	0.3846866	0.1682112	0.6298402
Palld	0.2694447	5.4852526	0.16837	0.6298443
Ap2s1	0.1568909	6.5655405	0.1683712	0.6298443
B230369F24Rik	0.4560277	0.6931524	0.1683807	0.6298443
Plaa	0.1528392	6.2858981	0.1684489	0.6298443
Vwa5a	-0.163265	5.5246137	0.1684949	0.6298443
Gmip	-0.1848888	6.2974166	0.1685135	0.6298443
Tomm20	0.1718158	6.3987524	0.168526	0.6298443
Kdelr1	0.169281	6.2790913	0.1685727	0.6298443
Crip2	0.2476945	4.6769154	0.168585	0.6298443
Matk	0.2501962	3.2565639	0.1686172	0.6298443
Becn1	0.1695207	7.1743804	0.1686916	0.62987
Adra2a	-0.4156427	1.2631571	0.1687051	0.62987
Siae	-0.2841598	4.0202802	0.1689129	0.630297
Lum	0.596916	7.4227	0.1689147	0.630297

Man1c1	-0.213991	5.1283273	0.168941	0.630297
Gm41408	0.5471887	0.7264718	0.1691524	0.6309343
9630013D21Rik	0.719441	0.5416045	0.1693097	0.6313696
Fam53a	0.2141582	4.1012522	0.169362	0.6314135
Cdc123	0.1629101	6.3970769	0.169467	0.6316082
Dhrs1	-0.1841447	4.9761211	0.1695624	0.6316082
Rps6-ps1	0.712583	-0.6769748	0.1695942	0.6316082
Bud23	0.1780649	4.6111877	0.1696528	0.6316082
Plekhj1	0.2199742	5.3929221	0.1696585	0.6316082
Hpn	0.3678715	2.9335104	0.1696921	0.6316082
Rps5	0.2233303	9.3267808	0.1697158	0.6316082
Dok1	0.1720738	5.5034224	0.1697391	0.6316082
Rps23-ps2	0.7752302	-0.5884705	0.1698483	0.6318381
Immp2l	-0.4028763	0.86344	0.1698821	0.6318381
Eif5a	0.163451	9.2652653	0.1699365	0.6318892
Dock10	-0.213692	6.8458896	0.1700903	0.632261
Mpp7	-0.2241925	4.331983	0.1702813	0.632261
Marchf2	0.3346314	7.815779	0.1702828	0.632261
Anxa9	1.3854792	1.3836497	0.1703082	0.632261
Chst14	0.3392729	2.4895645	0.170327	0.632261
Nlgn4l	0.2857803	2.7725757	0.1703282	0.632261
Clec16a	-0.212419	5.0077063	0.1703612	0.632261
Ddhd1	0.2956257	5.1788229	0.1703617	0.632261
Klhl30	-1.0414633	-0.3291536	0.1704632	0.6324549
Enpp1	-0.3078203	4.6520956	0.1704953	0.6324549
Rps19bp1	0.2107964	3.7858207	0.1706795	0.6329872
Syngr4	-0.6588401	-0.8883284	0.1707675	0.6331626
Gm10297	-0.6101275	-0.6229996	0.170844	0.6332953
Arrdc2	0.3447505	4.1086776	0.1709913	0.6336258
Nipal3	-0.2297084	5.4366643	0.171017	0.6336258
Plk1	0.1797388	7.0058472	0.1710554	0.6336258
St8sia2	0.7635221	0.6059301	0.1711099	0.6336771
Gm20590	0.2399237	3.9797717	0.1711952	0.6338419
Tmem267	-0.3984323	1.7633771	0.1712777	0.6338622
Tmem158	-0.2592323	2.9400862	0.1713359	0.6338622
Micu1	-0.1726429	5.3188055	0.1713601	0.6338622
Foxj3	0.1726458	5.1749988	0.1714843	0.6338622
Il33	0.9065654	-0.5009652	0.1715052	0.6338622
Lysmd3	-0.1714	5.5471439	0.1715081	0.6338622
Chchd2	0.349458	7.7517067	0.1715226	0.6338622
Sesn1	0.2206529	6.0532153	0.1715267	0.6338622
Chd2	-0.1765253	6.4869154	0.1716568	0.6340584
Emp2	-0.327168	3.2837541	0.171703	0.6340584

Galnt2	-0.1802855	6.3241379	0.1717329	0.6340584
Znrf3	-0.3094708	2.0917831	0.1717693	0.6340584
Ccdc38	0.6433693	-0.4674954	0.1718162	0.6340584
A330023F24Rik	-0.7051901	1.183001	0.1718509	0.6340584
Gm46430	0.2912081	3.6702283	0.1719003	0.6340584
Kcnf1	-0.9742313	1.0959412	0.1719059	0.6340584
Prxl2b	-0.2740742	3.1044517	0.1720312	0.6343702
Ankrd27	0.1780702	5.3645721	0.1720754	0.6343828
Psmd2	0.1635057	7.6452918	0.1721447	0.6344877
Tmpo	0.1838832	9.0471302	0.1722567	0.6347503
Enho	0.5414736	1.1172607	0.1722998	0.6347513
1700010H22Rik	-0.7255068	0.16019	0.1723394	0.6347513
Pear1	-0.269816	3.8651865	0.1723794	0.6347513
2510003B16Rik	0.7006951	-0.9781028	0.1724886	0.6350031
Rpl10a	0.1853476	8.9666357	0.1725542	0.6350564
Aldh3b2	0.372693	1.9000042	0.1726013	0.6350564
Jaml	-0.376767	5.1066309	0.1726551	0.6350564
Gnai2	-0.1911012	9.5306978	0.1726664	0.6350564
Csf2rb2	-0.2578914	4.6416019	0.1727717	0.6351693
Elovl6	0.2272196	4.0739242	0.1728504	0.6351693
Ttf2	0.2231972	4.7275643	0.172857	0.6351693
Gpn2	0.2415064	3.1441428	0.172917	0.6351693
Pde4d	-0.1856862	5.0020758	0.1729301	0.6351693
Gm19585	-0.8021354	0.1748855	0.1729598	0.6351693
Platr25	0.2261358	3.598924	0.1730185	0.6351693
Gm6733	-1.027546	-0.31054	0.1730238	0.6351693
Ccnb2	0.1904196	7.2673637	0.173247	0.6357303
Ttll12	0.257319	5.9209406	0.1732584	0.6357303
Gng12	-0.1721951	6.0505967	0.1734014	0.635951
Xrn1	-0.1566794	6.1294884	0.1734566	0.635951
Il17rc	0.5617695	0.8100387	0.173472	0.635951
Med8	0.1955919	5.7224861	0.1735338	0.635951
Rnd2	0.5671588	1.0857318	0.173559	0.635951
Dctn5	0.1576798	5.8695264	0.1736706	0.635951
Tsr3	0.2592873	3.5535886	0.1736999	0.635951
Gfra2	0.2893712	3.5301586	0.1737161	0.635951
Setd6	0.2996189	3.4417311	0.1737319	0.635951
Ino80b	0.2398781	4.3185399	0.173733	0.635951
Rab38	-0.2382169	3.4171835	0.1738128	0.635951
Anp32e	0.1678587	8.8188572	0.1739385	0.635951
H2-K1	0.1852738	8.7946444	0.1739403	0.635951
Acot12	-0.7659267	-0.1210877	0.1739419	0.635951
Kcnk12	-0.2899806	3.605592	0.1739973	0.635951

4931440P22Rik	-0.5956213	0.2227489	0.174037	0.635951
Tsacc	-0.6009157	0.5518519	0.1740778	0.635951
Gsk3a	0.1465683	6.8235198	0.1741601	0.635951
Tdp1	0.1934684	4.5814543	0.1741792	0.635951
Psen1	-0.2079002	6.2422663	0.1742103	0.635951
Zfp296	0.425411	1.9159522	0.174212	0.635951
Slc8b1	-0.2146179	5.1823488	0.1742925	0.635951
Fabp4	0.4237863	7.6134501	0.1743533	0.635951
Ptprm	-0.3414164	3.9273986	0.1743572	0.635951
Usp21	0.2023891	4.636463	0.174374	0.635951
Pln	-0.5462046	0.3080413	0.1743816	0.635951
Lrrc8b	-0.2129566	4.9057081	0.1745442	0.636165
Ube2m	0.1530374	6.7673369	0.1745572	0.636165
Mrpl39	0.160237	4.9813507	0.1745629	0.636165
Ermp1	-0.1599006	6.4639428	0.1746916	0.6362843
Csgalnact1	-0.2622962	3.3819065	0.1747341	0.6362843
Ranbp10	0.2716767	8.235761	0.1747599	0.6362843
Plekha8	-0.2449212	3.996913	0.1747754	0.6362843
Zfp961	0.1831418	4.5075162	0.1748002	0.6362843
Mtres1	0.2929481	3.7645433	0.1749992	0.6367194
Gm41604	0.4121318	2.7047845	0.1750498	0.6367194
Acsl5	0.1630516	7.3771992	0.175076	0.6367194
Rhd	0.2740863	8.0326107	0.1750895	0.6367194
Apol11b	0.5424114	4.180943	0.1751779	0.6367194
Amigo3	0.3786936	1.1760538	0.1751886	0.6367194
Gm40827	0.6205508	-0.0750671	0.1752063	0.6367194
Nit1	0.2113679	4.1702767	0.1753108	0.6369504
Mrps2	0.1938661	4.4024529	0.1754035	0.6371383
Isy1	0.1732901	5.3712836	0.1755134	0.6373888
Gm26512	0.5352021	-0.0204471	0.1758518	0.6382768
Lsm7	0.2540146	4.8047893	0.1758565	0.6382768
A730008H23Rik	-0.2354906	4.4752191	0.1759074	0.6382768
Bace2	-0.5472305	1.0305714	0.1759566	0.6382768
Gm33582	-0.9853461	-0.4572035	0.1759871	0.6382768
Wnt5a	0.4386901	1.7249862	0.1760042	0.6382768
AI839979	-0.3649585	3.7574361	0.1760846	0.6384194
Snip1	0.1711791	4.8880386	0.1761723	0.6384667
Rims3	-0.4783469	2.2754446	0.1761841	0.6384667
Dalrd3	0.2065508	4.8666011	0.1762715	0.6384667
Arhgef3	-0.1649452	6.0811183	0.1762999	0.6384667
Myo6	-0.2482807	4.6315723	0.1763028	0.6384667
Mettl27	0.3467591	4.0074377	0.1763691	0.638558
Ovca2	0.2709328	3.2264482	0.1765511	0.6390144

LOC118568281	0.8965666	-0.6076494	0.1765773	0.6390144
Ptar1	-0.1420561	5.88031	0.1768101	0.6397079
Cd151	-0.1824756	5.3529653	0.1768688	0.6397716
Hbp1	-0.152304	6.617725	0.1770769	0.6401493
Usp7	0.199364	8.6400601	0.177077	0.6401493
Pde7b	-0.3137585	3.8837052	0.1771576	0.6401493
Rspry1	-0.157677	5.5309243	0.1771807	0.6401493
D16Ertd472e	-0.2059146	5.1061755	0.177215	0.6401493
Klf1	0.2627395	7.6129088	0.1772202	0.6401493
Cyp27a1	-0.3664633	3.0354351	0.1772632	0.6401561
Spred2	-0.1938451	4.957414	0.1773075	0.6401674
9930021J03Rik	-0.1807965	5.3443906	0.1774376	0.6404885
LOC118567910	0.9552224	0.0386557	0.1775282	0.6406666
Gm46597	0.5740044	0.3145034	0.1776375	0.6409124
Znhit3	0.2863167	3.2500521	0.1777579	0.6411737
Purg	-0.3068953	3.2404868	0.1778103	0.6411737
Gle1	0.1634474	5.4773416	0.1778483	0.6411737
Pdia2	0.3080865	2.5392243	0.1778748	0.6411737
Gm29941	0.479796	0.2621337	0.1779639	0.6413461
Dbf4	0.1889666	6.430249	0.1780861	0.641552
Elp6	0.3910795	2.0682403	0.1781035	0.641552
Prrt2	0.6773835	0.4330225	0.1781909	0.6417181
Gm1968	-0.3967052	0.6969353	0.1782926	0.6417636
Mterf1b	0.3130966	1.9456606	0.1783501	0.6417636
Apmap	-0.1868823	5.6154522	0.1783654	0.6417636
Fat1	-0.3639521	6.4170912	0.1783685	0.6417636
S1pr2	-0.2910645	3.322248	0.1785119	0.6421308
Calm2	-0.168099	8.7951978	0.1785816	0.6421512
Tm2d1	-0.2220612	3.6167802	0.1786001	0.6421512
Mrps7	0.1955488	4.9681797	0.1787085	0.6423845
Nr3c1	-0.1635374	6.9259762	0.1787476	0.6423845
Mrpl48	0.2021796	5.3024953	0.1787962	0.6424107
Armh4	0.747838	-0.4245129	0.1788888	0.6424875
Zfp109	-0.3461066	2.1910795	0.1789236	0.6424875
Retn	0.6880756	0.3968053	0.1789542	0.6424875
Il17rb	0.556512	0.9763044	0.1790351	0.6424875
Ncoa1	-0.2601219	5.3985706	0.1790642	0.6424875
Pald1	0.3526992	3.2939639	0.1790676	0.6424875
S100pbp	0.2132309	5.0208896	0.1791597	0.6424875
Tmem86a	-0.2426954	4.7241523	0.1791702	0.6424875
Frmd7	-0.6156786	0.0531661	0.1792018	0.6424875
Polr2m	0.1421481	6.9829944	0.1792306	0.6424875
Tia1	0.1756109	6.0570494	0.17937	0.6425074

Klf9	0.2741395	5.2207818	0.1793972	0.6425074
Gm6204	0.3994982	1.9551391	0.1794494	0.6425074
Lctl	0.7033808	-0.4723764	0.1794638	0.6425074
Ube2d2a	0.1515917	7.0846301	0.1794814	0.6425074
Rpl36	0.1850601	7.8381604	0.1795091	0.6425074
Wrnip1	0.1729186	4.9978735	0.1795253	0.6425074
Wdr75	0.21128	5.1198821	0.1795932	0.6426024
Cdca5	0.1902541	5.2633277	0.1797862	0.6427276
Artn	0.6253927	0.9644649	0.179796	0.6427276
Armc8	-0.1694531	5.5757245	0.1797987	0.6427276
Spsb4	-0.6710495	-0.0898499	0.1798704	0.6427276
Pla2g4c	0.2376235	3.9101147	0.1798826	0.6427276
Ctbp1	0.1502446	8.0367386	0.1798926	0.6427276
Wdr73	0.2256614	3.6834733	0.1799238	0.6427276
Aco1	0.1652266	5.1661905	0.1799588	0.6427276
6330537M06Rik	0.5961511	-0.4657819	0.1800497	0.6429047
Cacng7	0.5754829	-0.2638665	0.1800964	0.642924
Polr2d	0.1778907	4.616538	0.1801385	0.6429268
Zbtb42	-0.3442843	2.9428093	0.1802123	0.6430424
Gm38999	-0.6440839	0.3942447	0.1803102	0.6431463
Tmem131l	0.1852117	7.0900043	0.1803324	0.6431463
Atl3	-0.160903	7.0011347	0.1804018	0.6431463
Ear6	-1.6190442	6.6155196	0.1804079	0.6431463
Sfmbt1	0.1625579	5.6770864	0.1804581	0.6431463
Omp	0.7422504	-0.1830107	0.180512	0.6431463
Ifit3b	-0.5776181	1.2768435	0.1806014	0.6431463
Dnm3	-0.7556669	-0.2858078	0.1806362	0.6431463
S100a11	-0.2401364	8.8368747	0.1806754	0.6431463
Lamc2	-0.6904907	-0.2087874	0.1806823	0.6431463
Tlr6	-0.2745577	3.1748766	0.1806963	0.6431463
Gm52329	-0.9095599	-0.6586081	0.1808476	0.6435246
Ocel1	0.2694496	2.8553916	0.1808853	0.6435246
Pcgf6	0.2942105	3.5188121	0.1809459	0.6435932
Tns2	0.3514965	4.2228263	0.1811308	0.6441034
Akap9	-0.2097728	5.9419966	0.1812059	0.644223
Ackr3	-0.6615774	3.2264112	0.1812694	0.6443016
Fbxw4	0.2355803	4.3251332	0.1813902	0.6445837
Ilf2	0.1465582	6.3379684	0.1814576	0.6445889
Tas1r3	-0.7505569	-0.4126259	0.1814745	0.6445889
Tfcp2l1	1.0041426	-1.2905037	0.1815687	0.6447762
Mtmr14	-0.1843264	5.5169323	0.1821112	0.646555
Appbp2	-0.2545855	5.0312582	0.1821773	0.6466421
Mtch1	0.1716867	6.4682568	0.1822217	0.6466522

Lpp	-0.1959194	5.1035823	0.1822856	0.6466612
Mpo	-0.2826946	10.865262	0.1823645	0.6466612
Ripk2	0.2588297	3.4361707	0.1824	0.6466612
Sulf1	-0.3499009	3.8492361	0.1824457	0.6466612
Zfp57	0.6945381	-0.1748336	0.1824478	0.6466612
Gm20559	0.218813	5.143147	0.1825139	0.6466612
Dtna	-0.4098416	3.5543062	0.1825153	0.6466612
Gm35039	-0.5445178	-0.1052622	0.1826169	0.6467406
Slc39a7	0.1518146	6.4552963	0.1826208	0.6467406
Spag1	0.3472149	1.9397798	0.1827192	0.6469417
Fhdc1	0.2695954	7.0872729	0.1828721	0.6473355
Cracdl	0.4516483	1.9596154	0.1829783	0.6475641
Trf	-0.1729642	7.7483984	0.1830681	0.647686
Dtx4	-0.1659321	5.2866452	0.183096	0.647686
Qser1	-0.2309981	5.2398476	0.1832079	0.6478938
Snu13	0.1723836	6.3477635	0.1832381	0.6478938
Irgm1	-0.1788588	4.9484989	0.1832883	0.6479242
Gm11837	0.4982812	2.3464666	0.1834384	0.648287
Ccdc15	-0.2573087	4.2514748	0.1834822	0.648287
Wdfy2	-0.1980566	5.8048425	0.1835226	0.648287
Efcab11	-0.291742	2.5640218	0.1835577	0.648287
Gm14198	0.5218571	2.3900021	0.1836415	0.6483056
Sec16b	-0.4093825	2.9813397	0.18367	0.6483056
Bcl7c	0.1721911	4.802997	0.183694	0.6483056
Cox6a1	0.1570449	7.8175912	0.1837297	0.6483056
Itgb5	-0.1819916	5.7415137	0.1838102	0.648306
Smap1	0.1929506	6.9394639	0.1838373	0.648306
Dgcr8	0.1708532	6.0297936	0.1838548	0.648306
Tubb1	-0.2922834	7.369735	0.1839491	0.6484392
Ier3	-0.2616869	2.5768085	0.183976	0.6484392
Arhgap4	-0.173519	6.6708267	0.1841585	0.6489355
Tmem200a	0.83027	0.1688622	0.1842804	0.6492181
Pmm2	0.1637404	5.3838364	0.1843266	0.6492336
Lpin1	-0.321994	4.2096932	0.1843839	0.6492779
Ggn	0.52016	-0.0536343	0.1844226	0.6492779
Dynll1	0.179006	7.4691484	0.1844984	0.6492859
Pitpna	0.1583094	7.7658983	0.1845084	0.6492859
Zfp637	0.2673559	3.2768141	0.1846213	0.649525
Paip1	0.1554706	5.5319614	0.184672	0.649525
Fam104a	0.1638125	5.971274	0.1847204	0.649525
Plcg1	0.2295474	4.2525415	0.1847734	0.649525
Gab2	-0.2009286	5.055992	0.1848056	0.649525
Synm	-0.3287265	3.1425663	0.1848631	0.649525

Twf2	-0.1436006	6.0563199	0.184981	0.649525
Isca1	0.2916343	7.9841416	0.1850155	0.649525
Hdac1-ps	0.4439539	4.6483284	0.1850317	0.649525
B3galnt2	0.1411969	5.4827059	0.1850465	0.649525
Xylt2	0.2741686	3.2398022	0.185075	0.649525
Adprhl2	0.2354806	3.3275491	0.1851438	0.649525
Ftx	0.2925281	4.155662	0.185157	0.649525
Gm32569	1.0800795	-0.6864525	0.185161	0.649525
Bst1	-0.2588457	4.8556715	0.1852413	0.6496105
Rbm6	0.14602	6.516203	0.1852694	0.6496105
Rps18-ps5	0.3295817	1.906272	0.1853473	0.6496105
Anxa2	-0.1815223	7.9440271	0.1853803	0.6496105
Ggta1	-0.1702794	5.6663169	0.1854282	0.6496105
Phip	-0.1847185	7.2001057	0.1855024	0.6496105
Pgd	-0.206941	8.643929	0.1855702	0.6496105
Gbp2	-0.2662461	3.5023654	0.185595	0.6496105
Gm5160	-0.5710363	-0.1357505	0.1856182	0.6496105
Frmd8	-0.1688616	5.305991	0.1856836	0.6496105
Slc1a4	0.398888	1.5254119	0.1857383	0.6496105
Tnf	-0.3712677	2.0278719	0.1857795	0.6496105
5930430L01Rik	0.4801757	1.6999927	0.1857959	0.6496105
Timm29	0.148647	5.6436033	0.1858404	0.6496105
Slc35a3	-0.1554479	6.060029	0.1859533	0.6496105
Bid	-0.1830082	5.3147707	0.1859576	0.6496105
Hic2	-0.3364121	1.8629402	0.1859847	0.6496105
Thoc3	0.1765624	5.0044906	0.1859988	0.6496105
Msh5	0.3899338	2.1598741	0.1860272	0.6496105
Tnfrsf14	0.2192201	8.1236915	0.1861286	0.6496105
LOC115489488	0.6156109	-0.486508	0.1862295	0.6496105
U2af2	0.1516153	7.7034644	0.1862513	0.6496105
Gpsm3	-0.1998467	6.6026835	0.1862589	0.6496105
Hapln1	-0.9179847	4.8101979	0.1862644	0.6496105
Necap2	0.1798525	6.8938903	0.186268	0.6496105
Opn3	-0.5694619	0.6661548	0.1862713	0.6496105
Nom1	0.1964544	5.1180013	0.1863751	0.6496453
Ddx42	0.143652	6.4898257	0.1864381	0.6496453
Epm2aip1	-0.1640195	5.9402121	0.1864543	0.6496453
Qrsl1	0.2827268	2.8706272	0.1864744	0.6496453
Kcnt2	-0.6820345	0.4907246	0.18649	0.6496453
Adamts7	0.6129265	2.1662287	0.1866995	0.650203
Ctnnd2	-0.5538247	0.1057153	0.1867733	0.650203
2810021J22Rik	0.324297	3.2803077	0.1868322	0.650203
Chst15	-0.235452	5.7415879	0.1868598	0.650203

Por	-0.1937783	5.7658854	0.1868977	0.650203
Hvcn1	0.1880567	6.9378416	0.186934	0.650203
Fnbp4	0.1689946	6.7920501	0.1869772	0.650203
Gm11687	-0.7145625	-0.9441721	0.1869846	0.650203
Epor	0.2780301	6.3091576	0.1871093	0.6504912
Tspan15	0.2620976	2.7436664	0.1872323	0.6506747
Gfi1	-0.2453379	5.0291657	0.1872457	0.6506747
Gm39556	-1.0095156	0.7754943	0.1872945	0.6506756
Htt	-0.1600415	5.9289205	0.1873296	0.6506756
Hfe	0.2450389	3.7255293	0.1873984	0.6507691
Sik3	-0.2004603	5.6360728	0.1875469	0.6511394
Col19a1	-0.689794	-0.8878041	0.1876269	0.6512718
Sirt4	0.3549335	2.709562	0.1876754	0.6512948
Manea	-0.1858783	5.2568637	0.1877313	0.6513433
Eif1	0.1816505	8.6242555	0.1878016	0.6514421
Gm13680	0.3924323	1.2133495	0.1878514	0.6514695
Fen1	0.2083816	7.4259074	0.1878944	0.6514733
Gm32645	0.8391318	-0.7980494	0.1879754	0.6515479
Higd1a	0.2360526	4.9865242	0.1879997	0.6515479
Smad7	0.2439163	4.4824253	0.1880825	0.6516896
Ndufa8	0.1517622	5.6796069	0.1881694	0.6518458
Ubac1	0.296306	7.890792	0.188233	0.6519208
Trim30c	0.5389998	0.0540564	0.1883041	0.6520218
2610507I01Rik	-0.6779857	5.6732474	0.1884152	0.6522614
Adamts5	-0.17345	5.456735	0.1888917	0.6532969
Rpsa-ps10	0.2682534	3.8597986	0.188903	0.6532969
Utp14b	-0.3675559	1.9652964	0.1889482	0.6532969
Psme3ip1	0.1519436	5.6625201	0.1890286	0.6532969
Zfp772	0.4148107	1.1074795	0.189048	0.6532969
Lrrc70	-0.2548304	3.0156639	0.1890898	0.6532969
Rragb	-0.4494156	1.167652	0.189113	0.6532969
Coro1a	-0.173947	9.359358	0.1891166	0.6532969
Impdh2-ps	0.3672673	1.3540306	0.1891215	0.6532969
Hnrnpa0	0.1552656	8.4677782	0.1891344	0.6532969
Sfpq	0.1634641	9.2796206	0.1891922	0.6533515
Polr2l	0.2175357	4.7862859	0.1893872	0.6537266
LOC118568699	0.6931612	-0.9818382	0.1894134	0.6537266
D330041H03Rik	-0.3042913	2.1154635	0.1894269	0.6537266
Tmed5	-0.1743701	6.0042262	0.1895504	0.6540079
Ctdspl	-0.2139022	5.2241841	0.1896458	0.6541918
Gm14681	0.6096714	2.0814388	0.1899897	0.6552328
Vegfd	0.5723172	-0.0519957	0.1901615	0.6555076
Ablim2	-0.6695308	0.04786	0.1901815	0.6555076

Stk11ip	0.2011919	4.4742536	0.1901958	0.6555076
2810006K23Rik	0.2415243	3.3141132	0.1902978	0.6557138
Slit2	-0.3474611	5.0379221	0.1904294	0.6559402
Tmem176b	0.2201527	6.7371958	0.1905443	0.6559402
Rnf114	-0.1459483	5.9661409	0.1905487	0.6559402
Mocs1	0.1677347	5.9544367	0.1905742	0.6559402
Zfp719	-0.2497972	3.3264357	0.1905744	0.6559402
Gm10288	0.2894147	3.1379739	0.1907674	0.6563006
Tspyl3	0.2360751	3.0635844	0.1907687	0.6563006
Nfix	0.1616448	6.2019356	0.1908136	0.6563006
Itgb1	-0.1519849	8.7020373	0.1908479	0.6563006
Cyhr1	0.1503475	5.6660358	0.1909195	0.6563526
Gm15441	0.417853	0.7578969	0.190965	0.6563526
Trim26	0.1858183	5.2029353	0.1909896	0.6563526
Cenpa	0.1783957	6.9319677	0.1911315	0.6566952
Zfp706	0.1687044	6.7845527	0.1912262	0.6568755
Prnp	0.2196997	6.0944302	0.1914376	0.6572939
Gm13986	-0.7340647	-0.55564	0.1915923	0.6572939
Htra1	-0.3834208	4.2420753	0.1916217	0.6572939
Rps11	0.1546538	8.9688465	0.1916706	0.6572939
E130309D02Rik	0.1824618	4.9280392	0.1916728	0.6572939
Gm8848	0.8436947	-0.0492674	0.1916809	0.6572939
Cd164	-0.1468367	8.0072412	0.1917573	0.6572939
Lrwd1	0.1518659	5.487035	0.1917707	0.6572939
5830448L01Rik	0.2902352	4.0966631	0.1918429	0.6572939
Rph3al	0.3002725	2.0802572	0.1918478	0.6572939
Rhbdf2	-0.164829	5.1373712	0.1918527	0.6572939
Lyrm1	-0.4403275	0.9913264	0.191857	0.6572939
AW549877	-0.1773922	5.5078032	0.1918974	0.6572939
Gm3704	0.9318752	-0.9894055	0.1920958	0.6576998
Six4	-0.6064934	0.6078252	0.1921005	0.6576998
Abcg4	0.2766793	5.6824531	0.1922859	0.6581898
Prkcb	-0.2232546	7.4361483	0.1924049	0.6584524
Tlr5	-0.3104397	2.2273015	0.1925524	0.6588121
Alg8	0.3425167	3.9922032	0.1926226	0.6589075
Cideb	-0.5415198	-0.021969	0.192665	0.6589076
Gm5903	-0.3887844	1.0682686	0.1927277	0.6589614
Fam136a	0.2257552	3.9706339	0.1928393	0.6589614
Gm39590	0.6447366	-0.5955258	0.1928812	0.6589614
Abt1	0.1976877	3.616669	0.1928902	0.6589614
Abhd13	-0.1581092	5.4407881	0.1928926	0.6589614
Gnl1	0.1792249	4.8398119	0.1931073	0.6595302
Amfr	-0.148736	7.2911576	0.193176	0.6595302

Ddrgk1	0.1606956	5.3800241	0.1931863	0.6595302
Dnajb3	0.4973844	2.5721411	0.1934364	0.66008
Notch4	0.2971261	2.996747	0.1934446	0.66008
Zfp511	0.2413804	3.5173841	0.1934957	0.66008
Neto2	-0.2828805	3.4515738	0.1935171	0.66008
Ube3b	0.1483268	6.00483	0.1936474	0.6603797
Fam117a	0.2048686	7.7580645	0.1937801	0.6604089
Paf1	0.1473999	5.9114712	0.1937902	0.6604089
Kctd11	-0.2570889	3.2954994	0.1939007	0.6604089
Aip	-0.1432736	6.3203229	0.1939294	0.6604089
Armc3	-0.4436788	0.7015334	0.1939467	0.6604089
Tyw3	0.3386417	2.0906699	0.1939633	0.6604089
Dlat	0.1519978	5.1168081	0.1940746	0.6604089
Lhfpl2	0.1829986	4.8142037	0.1940832	0.6604089
H2-Aa	0.3633586	7.1489893	0.1940986	0.6604089
Wnt10a	1.2348498	-0.4740473	0.1941172	0.6604089
Gm32450	-0.6404981	-0.8102073	0.194123	0.6604089
Isg20	0.3027756	7.3474778	0.1942876	0.6608243
Coa6	-0.263322	3.6908798	0.1943721	0.6608738
Rpl3-ps1	0.1711551	6.0507827	0.1944019	0.6608738
Rad51b	-0.292569	2.1607718	0.1944296	0.6608738
Tgtp1	-0.4612857	1.8716189	0.1946063	0.6610876
Ell3	0.5978196	1.0462592	0.1946718	0.6610876
Zfp934	0.3316676	2.189796	0.1947369	0.6610876
Gm32051	-0.5857056	1.1228273	0.1947435	0.6610876
Rab3il1	0.2607617	6.9458328	0.1947807	0.6610876
Fam184a	-0.541372	0.3521785	0.1947861	0.6610876
Cyb561d1	-0.2098015	4.3899951	0.1948469	0.6610876
Rbpms	-0.3377103	3.2289891	0.1948715	0.6610876
Ess2	0.2130725	3.9243562	0.1948824	0.6610876
Tslp	-0.4350211	0.3572882	0.1949547	0.6610876
Cdca7	0.1554738	6.4468062	0.1949601	0.6610876
Adamts2	0.3081547	5.9379169	0.1950502	0.6612092
Gfra4	0.4081743	0.4711985	0.1951175	0.6612092
Rasl10a	0.7403399	-0.7507674	0.1951235	0.6612092
Nscme3l	0.7449229	-0.7170371	0.1952508	0.6614965
Hmgb1	0.223459	9.4049311	0.1953492	0.6616536
Slc22a17	0.4897858	2.4415093	0.1953822	0.6616536
LOC115487949	0.5908092	1.3590648	0.1954979	0.6619014
1110038F14Rik	0.2027493	4.1897425	0.1957342	0.6625572
Cit	0.2383554	6.5258416	0.1959808	0.6629845
Neurl3	-0.2008721	5.9076358	0.1960178	0.6629845
Htr2a	-0.428425	1.6997553	0.1960189	0.6629845

Git1	-0.1633246	5.3489074	0.196037	0.6629845
Dpf3	0.3674102	2.5838938	0.1960736	0.6629845
Gpsm1	0.27673	3.3604593	0.1964267	0.6640339
Itm2b	-0.1642327	9.9425454	0.1965926	0.6644253
Slc25a20	-0.2212552	4.6040592	0.1966279	0.6644253
Slc37a1	-0.32445	3.4836024	0.1967371	0.6646499
0610010F05Rik	-0.1827611	4.3714394	0.1969843	0.6653148
Aen	0.2307756	4.0425256	0.1970483	0.6653148
Kirrel	-0.3185173	4.9961128	0.1970622	0.6653148
Art1	-0.8798582	-0.8325416	0.1971327	0.6654085
Rhbdd1	-0.1773652	4.5838188	0.1972211	0.6654957
Gm41753	1.0638126	-1.2790193	0.1972794	0.6654957
Il1rn	-0.2363208	5.7487809	0.1972869	0.6654957
Mrpl37	0.1665169	4.7466929	0.197395	0.6656597
Itch	-0.155001	6.6645026	0.1974211	0.6656597
Irx3	-0.3122255	3.2338582	0.1975338	0.6657627
Ldlrap1	-0.1657247	6.0173333	0.1975596	0.6657627
Fxyd5	-0.1888914	7.5201444	0.1975801	0.6657627
Rarg	-0.2362777	4.8003358	0.1977005	0.6659462
Tmem182	-0.5836646	2.6651838	0.1977266	0.6659462
Gm32707	0.810599	-0.6353148	0.1977754	0.6659462
Gm32287	-0.4639808	0.9666929	0.1978058	0.6659462
Lonrf1	-0.2318548	3.9088882	0.1979042	0.6661061
Nkain1	-0.4663483	2.1851462	0.1979783	0.6661061
Hcn2	0.6014406	-0.5864902	0.1980334	0.6661061
Smad3	-0.2358954	4.9921595	0.1980938	0.6661061
Ago3	-0.2188293	4.6086934	0.1982213	0.6661061
Cep170	-0.1789534	6.3391481	0.1983017	0.6661061
Cdk2ap1	0.1838197	5.3057504	0.1983293	0.6661061
Hpcal1	-0.1877926	5.2567825	0.1983818	0.6661061
Acot9	-0.1764478	4.9406399	0.1983819	0.6661061
As3mt	0.2009621	3.8295311	0.1984398	0.6661061
Zbtb41	-0.1716536	4.8534629	0.1984979	0.6661061
Fancg	0.1725867	4.1959035	0.1984984	0.6661061
Zdhhc1	0.3570065	2.1703236	0.1985072	0.6661061
Nmnat3	0.2932381	4.4326617	0.1985112	0.6661061
Espl1	0.2077703	6.2538862	0.1985303	0.6661061
Cd37	0.2085971	7.1514067	0.1985882	0.6661061
Adgrg5	-0.9328162	0.553508	0.1985973	0.6661061
Vps29	-0.1505357	5.511827	0.1986484	0.6661061
Syngr2	0.1558943	6.4572353	0.1987395	0.6661061
Selplg	-0.2109418	7.7386082	0.198762	0.6661061
Igkv4-68	-0.5675518	2.8069243	0.1988275	0.6661061

Scimp	-0.5750843	0.4390569	0.1988741	0.6661061
Eif2b4	0.1863496	4.733038	0.1989006	0.6661061
Cc2d2b	-0.4598442	2.0327762	0.1989264	0.6661061
Pcdhgb5	-0.797473	-0.8685725	0.1989572	0.6661061
Gm25432	0.5677721	0.8523132	0.1990436	0.6661061
Srebf1	0.1712865	5.4049067	0.1990506	0.6661061
Nrbf2	0.2069076	4.1274035	0.1990524	0.6661061
Kat6b	-0.2396005	4.5049371	0.1991199	0.6661649
U2af1l4	0.2249962	3.9312178	0.199189	0.6661649
Nat8f4	-0.491629	0.6808215	0.1992276	0.6661649
Klhl12	0.2115982	6.1596802	0.1992413	0.6661649
Ank	0.2214011	6.7513142	0.1994002	0.6664304
Mga	-0.1761568	6.5465164	0.1994384	0.6664304
Polrmt	0.2169123	3.8086793	0.1994492	0.6664304
Tardbp	0.1510207	8.5485174	0.1995364	0.6665783
Zfp974	0.3687301	1.67331	0.1995918	0.6666202
Gm10505	0.4637438	0.7819889	0.1996657	0.6667241
Dennd5b	0.3277241	4.1790225	0.1997127	0.6667379
Atp6v1a	-0.1519986	7.3519107	0.1998012	0.66689
Cant1	0.1545027	5.4790161	0.1998958	0.6670627
Gm2000	0.2621238	3.9246199	0.1999623	0.6671413
Kmo	0.2479807	3.5612358	0.2000503	0.6672853
Pcnx4	-0.3497319	2.2678832	0.2001063	0.6672853
Kyat1	0.3138004	1.9152707	0.2001341	0.6672853
Ppie	0.1781013	4.5357455	0.2002459	0.6674071
Ccdc122	0.6112909	-0.2476098	0.2002868	0.6674071
Hlx	-0.2798113	4.3693818	0.2002994	0.6674071
Slc9a1	-0.2125561	5.6155149	0.2004933	0.6679103
Arntl	0.2606306	4.5183246	0.2006406	0.6680888
Uqcrc1	0.1433283	7.1026305	0.200711	0.6680888
Fbh1	-0.1482698	5.7224576	0.2007551	0.6680888
Plag1	0.3809314	1.960248	0.2007631	0.6680888
Gp9	-0.1758364	5.9782488	0.200813	0.6680888
2810414N06Rik	-0.6137912	-0.5301243	0.200855	0.6680888
Arpc5	-0.1634292	8.7911181	0.2008755	0.6680888
Dazap1	0.1431873	7.0278046	0.2008905	0.6680888
Nupr1	0.4432841	5.6462457	0.2010143	0.6681383
Tnfrsf23	-0.2810458	2.6193122	0.2010186	0.6681383
Cabcoco1	-0.7063461	-0.4474553	0.2010343	0.6681383
Csnk1g3	0.1616857	5.7096629	0.201274	0.6684062
Siglecg	0.3159081	5.3622905	0.2013397	0.6684062
Ehbp1l1	0.1836161	8.1070509	0.2013619	0.6684062
Racgap1	0.1620461	7.0535045	0.2014535	0.6684062

Cct2	0.1433191	8.0079567	0.2014595	0.6684062
Slc25a39	0.2467727	7.3926883	0.2014813	0.6684062
Ncaph2	0.1664035	7.2757133	0.2015113	0.6684062
2610306M01Rik	0.6138713	0.8438125	0.2015665	0.6684062
H2-M9	-0.8325595	-0.4349217	0.2015844	0.6684062
Psmd8	0.1350158	6.932382	0.2015909	0.6684062
A630072M18Rik	-0.2232921	3.7680254	0.2016437	0.6684062
Fam174c	0.293146	2.2419329	0.2017004	0.6684062
Slc25a19	0.3058041	3.729255	0.2017556	0.6684062
Zfp438	0.3040341	1.745446	0.2017619	0.6684062
1810053B23Rik	0.5001667	0.986715	0.2018227	0.6684062
Lyve1	-0.5328957	1.3320787	0.2018403	0.6684062
Ttpal	0.1989908	5.2489729	0.2018861	0.6684062
Paxbp1	0.1845234	6.2939877	0.2019346	0.6684062
Sf3a2	0.17149	5.794078	0.2019471	0.6684062
Elk3	0.1902018	5.4888293	0.2019743	0.6684062
Usp8	-0.1503101	6.5515252	0.2020524	0.6684351
Arfgef3	-0.5909026	0.0168799	0.2020982	0.6684351
Lrp5	-0.188198	5.9011047	0.2021232	0.6684351
St8sia1	0.9157036	3.1844353	0.2022217	0.6684351
Sugp1	0.1581788	4.8296416	0.2022711	0.6684351
Il21r	0.2369802	4.5172182	0.2022737	0.6684351
Borcs5	0.338962	1.9046703	0.2022839	0.6684351
Ankrd9	0.2968757	4.2135976	0.2023483	0.66845
Gm32067	0.8597865	-1.2693188	0.2023744	0.66845
Rps27a-ps3	0.4256436	1.4702335	0.2025323	0.6685175
Inpp5k	-0.1330531	5.7678068	0.2026488	0.6685175
Snhg6	0.3084	3.5306551	0.2027031	0.6685175
Nbl1	0.5188016	3.499638	0.2027397	0.6685175
Plcb1	-0.390843	2.6385285	0.2027618	0.6685175
Aars2	0.2445479	3.0020973	0.2027672	0.6685175
Tnnt1	-0.4705131	2.268543	0.2028694	0.6685175
Gm4045	0.6656982	0.0802438	0.2029307	0.6685175
Uqcc3	0.1984317	4.4521533	0.2029461	0.6685175
Grk5	-0.2031361	4.4594158	0.2029641	0.6685175
Bmp8a	-0.6607914	1.4840187	0.2029744	0.6685175
Mrgpre	0.4793492	1.2526698	0.2030199	0.6685175
Mark4	-0.2086881	4.7279951	0.2030369	0.6685175
Srrm1	0.1600831	8.0129784	0.2030555	0.6685175
Alg9	0.2239341	3.6451801	0.2030693	0.6685175
Vipr1	-0.414493	0.8489601	0.2030825	0.6685175
Ckap2	0.2006555	5.8340761	0.2032903	0.6690599
Hmgb3	0.1819874	7.3000109	0.2036736	0.6700464

Sipa1l1	-0.2337202	5.1071963	0.2036762	0.6700464
Arhgap10	-0.2567507	4.6256088	0.2038481	0.6702465
Csnk1a1	0.1415125	8.38855	0.2038523	0.6702465
Rasip1	0.2779974	3.2219756	0.203879	0.6702465
Noa1	0.192558	4.7437241	0.2040389	0.6702465
Cpeb4	-0.1908102	8.3218966	0.2040536	0.6702465
Ctf1	-0.6682818	-1.0508447	0.2041033	0.6702465
Gm45734	0.5866204	-0.8917933	0.2041747	0.6702465
Adnp	-0.3525545	3.1433175	0.2042265	0.6702465
Psme3	0.208138	8.3012275	0.2043655	0.6702465
Iqce	0.2176002	3.2032401	0.2044634	0.6702465
Zfp91	0.1562992	7.1295853	0.2045067	0.6702465
Gvin1	-0.3855426	2.1214962	0.2045296	0.6702465
Abraxas2	-0.1496977	5.4462466	0.2045931	0.6702465
Vps35	-0.1617559	7.6847375	0.2045935	0.6702465
C8g	-0.9508549	-0.3439305	0.2045942	0.6702465
Gm51779	-0.475693	0.5903395	0.204612	0.6702465
Srsf9	0.1444622	6.0307592	0.2046242	0.6702465
Nsmce4a	0.1654219	6.6427069	0.2046505	0.6702465
Atp6ap1	0.1653578	7.8370108	0.2046812	0.6702465
4930522L14Rik	0.2203589	3.296658	0.2047099	0.6702465
Pla2g7	-0.2260549	6.2752732	0.2047126	0.6702465
Timd4	-0.3573745	3.9251669	0.2047238	0.6702465
Capg	-0.175296	6.7750718	0.2047281	0.6702465
F2rl1	-0.6994278	-0.6736425	0.2048955	0.6706534
Prag1	0.3540451	2.5630247	0.2050188	0.6709157
H4c17	0.6737905	-0.4004578	0.205201	0.6713708
Hspb1	-0.6775762	0.0548172	0.2053246	0.6713993
Rpl36a-ps2	0.3293578	3.1761365	0.2053371	0.6713993
Vta1	-0.1534082	5.2309924	0.2053392	0.6713993
C77080	-0.4668404	2.4913059	0.2054612	0.671609
Cmc2	0.1848429	5.1802763	0.2054897	0.671609
Arl11	-0.1801348	4.7261713	0.205666	0.6720437
Galnt14	0.9446884	-0.7887826	0.2058256	0.6723015
Cisd2	0.137773	5.8111513	0.2060215	0.6723015
Traf1	0.4827106	2.0227023	0.2060355	0.6723015
Rnf145	0.1660459	6.824372	0.2060525	0.6723015
Brat1	0.2374801	3.6303567	0.2060766	0.6723015
Comt	0.1714649	6.1394635	0.2061092	0.6723015
Rhod	0.3830844	1.9398617	0.2061187	0.6723015
Slc38a4	-0.7738438	0.7679013	0.2061223	0.6723015
Fasl	-0.5224375	-0.5864931	0.2061629	0.6723015
Inpp5j	-0.4914035	2.4952414	0.2061771	0.6723015

4430402I18Rik	0.8153309	-1.0720301	0.2062551	0.6724149
Cdc23	0.1419056	5.9799842	0.2063136	0.6724646
Nme6	-0.261285	3.1385442	0.2065194	0.6729946
Rarb	0.8703605	-0.9760903	0.2065681	0.6730122
Nr4a2	0.5334967	2.082704	0.2067798	0.6734314
Son	-0.1563402	8.7576391	0.2068081	0.6734314
Rock2	-0.1817035	7.3827568	0.2068516	0.6734314
Dars	-0.1488786	7.059086	0.20687	0.6734314
Atxn1l	-0.1717603	5.5812668	0.2070154	0.6737639
Dnd1	0.4336855	1.0444097	0.207101	0.6739015
Bbs12	0.3423435	2.3500454	0.2071639	0.6739092
Mad2l2	0.2212841	2.9944034	0.2071984	0.6739092
Gm8276	0.6911483	-0.8056212	0.2072334	0.6739092
Morn4	-0.512168	0.166568	0.2073483	0.6740528
Mtrf1	0.2823612	2.4809263	0.2073745	0.6740528
Gm36317	-0.6646512	0.7339343	0.2074075	0.6740528
Map6	-0.69819	-0.1615826	0.2075423	0.6743173
Gm33989	-0.2054545	5.2312049	0.2076461	0.6743173
Gm2423	0.7326323	3.8952399	0.2076579	0.6743173
Insig2	-0.2276203	4.9039602	0.2076631	0.6743173
Tysnd1	0.1801484	4.3070198	0.2077057	0.6743173
Rilpl2	-0.1962796	4.1532062	0.2079575	0.6749377
Zfp954	0.2770332	3.2763044	0.2079846	0.6749377
Ccdc84	0.2722037	3.7401179	0.208027	0.6749377
Treml1	-0.2362708	5.7889772	0.2081478	0.6751455
Gm33111	0.3417233	1.5528256	0.2081778	0.6751455
Slc4a1	0.2745017	11.427204	0.2082627	0.6752799
Mypop	0.5342479	0.5497827	0.2083842	0.6755259
Ndufa2	0.2088771	5.8162468	0.2084254	0.6755259
Tbk1	-0.159202	5.6744695	0.2085249	0.6757075
Zc3h15	0.1352768	6.3954954	0.2086502	0.6757374
Yeats4	0.164018	6.1897494	0.2086608	0.6757374
Cenpv	0.2309898	3.5904346	0.2086644	0.6757374
Tapt1	0.1417214	7.1801139	0.2089263	0.6761792
Zfp763	0.3025868	2.6601929	0.2090188	0.6761792
Wars2	-0.2278144	3.1497404	0.2091299	0.6761792
Naca	0.1978912	8.7741374	0.209156	0.6761792
Mapk9	0.1481419	5.7257899	0.2091587	0.6761792
Selenon	-0.1838991	5.0321185	0.209173	0.6761792
Nmnat1	0.2799749	2.3068954	0.2091731	0.6761792
Tmem243	0.2767341	3.9637775	0.2092719	0.6761792
Hmbs	0.2762594	9.0324309	0.2092762	0.6761792
Iqcg	-0.6304388	-0.2153901	0.2093459	0.6761792

Nmt1	0.1390183	6.8028718	0.2093843	0.6761792
Sec23a	-0.1962127	6.0106692	0.2093895	0.6761792
Gm33504	-0.7632139	0.0032399	0.2093955	0.6761792
Slc7a6os	0.1814224	4.365688	0.209605	0.6761792
Stat3	-0.1988871	7.0419055	0.2096719	0.6761792
Hnrnpr	0.1556901	7.1252108	0.209723	0.6761792
Trim2	0.2486014	4.0247951	0.2097239	0.6761792
Cox6b1	0.1950355	7.6851417	0.2098393	0.6761792
Gm12586	-1.2303816	0.9685925	0.2098871	0.6761792
Trim27	0.152904	5.9669833	0.2100016	0.6761792
Rabac1	-0.1638892	5.4802698	0.2100294	0.6761792
Idh3a	0.1517261	6.0591602	0.2100311	0.6761792
Aak1	-0.1516657	6.0139576	0.2100711	0.6761792
Map3k14	-0.2150465	3.6934943	0.2102406	0.6761792
N4bp3	0.3158399	2.8197405	0.2102489	0.6761792
Gm11539	-0.5588562	-0.9747654	0.2102617	0.6761792
Samhd1	-0.1677246	7.8903901	0.2102947	0.6761792
Zfp597	-0.1697585	4.9331241	0.2104256	0.6761792
Sec24a	-0.1979458	4.9220689	0.2104709	0.6761792
Zfp995	0.3666638	1.9286742	0.2105239	0.6761792
Swsap1	0.2373029	2.944698	0.2106095	0.6761792
Elp5	0.2372284	4.7854716	0.2106101	0.6761792
Mob2	0.2084189	5.3160167	0.21066	0.6761792
Nob1	0.1942759	4.2809102	0.2106673	0.6761792
Dnajc5	-0.1629834	6.9926908	0.2106787	0.6761792
Tshz3	0.4640673	1.3997402	0.2106825	0.6761792
Rps18	0.243286	9.3744489	0.2106975	0.6761792
Gcfc2	0.229889	3.7862651	0.210754	0.6761792
Aars	0.1926199	7.1328801	0.2107792	0.6761792
Skap1	-0.5442093	0.8187419	0.2108166	0.6761792
Pcsk4	0.421637	1.0902473	0.2108229	0.6761792
Sp1	-0.15709	7.4653192	0.2108323	0.6761792
Gm12279	0.6040141	-0.4922323	0.2108391	0.6761792
Inpp5f	-0.2103237	3.8305906	0.210873	0.6761792
Nedd9	-0.1908083	6.5502919	0.2108922	0.6761792
Smarce1	0.1336113	6.9927832	0.2109161	0.6761792
Gm40095	-0.8086101	-0.589481	0.2109576	0.6761792
Csf2rb	-0.2290761	6.7775296	0.2110891	0.6761792
Myo1h	-0.542517	0.4530012	0.2110897	0.6761792
Spata6	0.2384155	3.3139532	0.2111088	0.6761792
Gm32856	0.2788093	2.3232338	0.2111333	0.6761792
H2-T22	0.2418033	4.8432861	0.2111692	0.6761792
Plekha6	-0.3839142	1.5600603	0.2112248	0.6761792

Gm17025	0.6642934	-0.3246219	0.2113546	0.6761792
Tcaim	0.267301	2.452335	0.211412	0.6761792
Hpgd	-0.2800335	5.8671812	0.2114873	0.6761792
Baz2b	-0.1450513	6.7553834	0.2115342	0.6761792
Chn2	-0.2680862	2.7524451	0.2115364	0.6761792
Angptl6	0.5787692	-0.4083388	0.211563	0.6761792
Napa	0.1437902	6.6729476	0.2116805	0.6761792
Hyi	0.3476463	2.153005	0.2116813	0.6761792
Oip5os1	-0.1597988	6.3090897	0.2116993	0.6761792
Fam210a	-0.1678399	5.3535182	0.2117054	0.6761792
Gm30948	1.5351456	0.082124	0.2117143	0.6761792
Tm7sf2	-0.3675024	1.5175757	0.2118155	0.6761792
Rpia	0.1374567	6.7973151	0.2118568	0.6761792
Pigx	-0.216207	4.4682669	0.2118705	0.6761792
Jup	-0.2359514	4.7896003	0.2119337	0.6761792
Arhgap28	-0.3218007	1.9344989	0.2119513	0.6761792
Jag2	-0.5212202	1.302708	0.2120136	0.6761792
Atg3	-0.1674956	6.2761375	0.2120695	0.6761792
Gm39792	-0.4703633	0.6512586	0.2120899	0.6761792
Helq	0.2136117	4.3307676	0.2121095	0.6761792
Slc7a1	0.215306	5.9915758	0.2121111	0.6761792
Smim4	-0.3127661	3.4563687	0.2121135	0.6761792
Ulk1	-0.1447628	5.8086634	0.2121196	0.6761792
Trps1	-0.1763587	5.0593811	0.2121483	0.6761792
Matn3	-1.0644089	2.4717624	0.2122003	0.6762063
LOC118567450	0.8485315	-0.2938778	0.2122506	0.6762281
Tgfb2	-0.4095009	2.1589554	0.2122978	0.6762401
Lars2	0.1669073	5.0706205	0.2130751	0.6785166
AU040320	-0.1992791	5.0294687	0.2131432	0.6785166
Atp1b2	0.2529767	5.3997774	0.2131553	0.6785166
Fermt3	-0.2162298	8.2084588	0.2132092	0.6785166
Gm6809	-0.5445673	0.5354407	0.2132342	0.6785166
Rel	-0.2144274	5.4625327	0.2132742	0.6785166
Flad1	-0.2235431	3.8206967	0.2134422	0.6787215
Fbxl19	0.1754077	4.6486204	0.2134598	0.6787215
Gm6652	-0.6081346	-0.0183003	0.2135182	0.6787215
D030055H07Rik	-0.6130039	-0.3767637	0.2135423	0.6787215
Ndufa11	0.1788686	5.3281294	0.2135569	0.6787215
Cers4	0.3108788	2.7683263	0.2136757	0.6789604
Ttll7	-0.4264727	1.339568	0.2138646	0.6792557
Herc4	-0.1279551	6.5822082	0.213888	0.6792557
Endog	0.31219	2.1145622	0.2139667	0.6792557
Srprb	0.1858049	4.8220528	0.2139882	0.6792557

Kifc2	0.413071	1.7521983	0.214	0.6792557
Nrarp	0.3835256	2.9888625	0.2140306	0.6792557
Sharpin	0.1496026	5.1191547	0.2140956	0.6792852
Prdm11	0.2985926	3.1622981	0.2141273	0.6792852
Gm13436	0.2552767	2.8597494	0.2141744	0.6792961
Anxa8	-0.5955933	1.7547992	0.214352	0.6793429
Gm7730	0.5816988	-0.7955466	0.2143594	0.6793429
Gm10130	0.5020385	1.2726538	0.2143892	0.6793429
Tarbp2	0.2016001	3.6264065	0.2144374	0.6793429
Pak3	-0.6424992	0.2427937	0.2144699	0.6793429
Ndufv3	-0.1496667	6.1717886	0.214526	0.6793429
Blvrb	0.2432374	8.0602429	0.2145302	0.6793429
Mapk1ip1l	0.1377293	6.7460879	0.2146418	0.6793429
COX2	0.2207851	5.056745	0.2146424	0.6793429
Snx19	0.1568621	4.6816127	0.2147022	0.6793429
Bet1l	-0.2040929	4.3089148	0.2147707	0.6793429
Zfp180	0.1759477	5.0276558	0.2147855	0.6793429
Rrp9	0.2332476	3.7443605	0.2147872	0.6793429
Crbn	-0.1526721	5.7162757	0.2148006	0.6793429
Mss51	0.3819515	1.64192	0.2149638	0.6797207
Ftsj3	0.1611564	6.0606683	0.2150086	0.6797244
Scaf4	0.1649283	6.0277157	0.215143	0.6800109
Smim10l2a	0.7023049	-0.8431684	0.2152163	0.680079
S100a16	0.3209348	3.3511092	0.2152677	0.680079
Isoc2b	0.7188011	0.2567147	0.215354	0.680079
Proca1	-0.6528035	0.8057064	0.2153734	0.680079
Slc12a4	0.1817724	5.2007772	0.2154817	0.680079
Bambi	0.4569615	4.9273383	0.2155328	0.680079
Gm34020	-0.4857533	1.0984297	0.2155333	0.680079
Nhlrc4	0.6719323	0.4189087	0.2155347	0.680079
Tpr	-0.1825933	8.1955737	0.215558	0.680079
Tigd5	0.3858871	1.1952226	0.2156451	0.6800992
Supt20	-0.1413964	6.2575421	0.2156519	0.6800992
Tfam	0.1467774	5.3291865	0.2157957	0.6802208
Gm10257	0.3439194	1.7419546	0.2158123	0.6802208
Prkce	0.3360554	4.1497018	0.2158514	0.6802208
Me2	-0.1425943	6.8821286	0.2158654	0.6802208
Ear1	-1.5213767	7.1402831	0.2160943	0.6808042
Abca1	-0.2191442	5.9845245	0.2161559	0.6808605
Mrpl13	0.1771131	5.1465914	0.2165983	0.6820954
Tmed4	-0.1645168	4.6570556	0.2166357	0.6820954
Brd8	0.1564581	6.6723597	0.2166835	0.6821078
Gm51604	0.7398662	0.157566	0.2167374	0.6821396

Ppp3cb	0.1422636	7.0382747	0.2167884	0.6821619
Grsf1	0.14324	5.9089426	0.2169385	0.6824749
Rnf122	0.3217872	2.5333934	0.2170018	0.6824749
Xylb	0.3525429	1.3831249	0.2170195	0.6824749
Ghr	-0.276464	4.9160999	0.217069	0.6824928
4930509E16Rik	1.0484712	-0.7934244	0.2171612	0.682602
Spryd7	0.3179823	2.8500757	0.217192	0.682602
Rps6kc1	-0.1787999	4.1751864	0.2172354	0.682602
Dbndd2	0.3872474	2.8339958	0.2173926	0.6827703
Pdpn	0.5996789	2.7128209	0.2174057	0.6827703
Nup93	0.1493444	5.5275429	0.2174446	0.6827703
Zdhhc18	-0.1479124	5.9377334	0.2174844	0.6827703
Ikzf3	0.3974577	5.6393128	0.2175231	0.6827703
Abcc2	-0.6470218	1.473984	0.2175524	0.6827703
Slc35b1	0.1715676	4.9383198	0.2178601	0.6835982
Hnrnpd	0.1525952	8.7815716	0.2180032	0.6836799
Hcar1	0.9634269	0.7351233	0.2180459	0.6836799
Gm16150	-0.5778777	0.1831099	0.2180625	0.6836799
Fsd2	-0.7345696	-0.2114594	0.218076	0.6836799
D030056L22Rik	0.2139705	5.0344202	0.2181059	0.6836799
Exosc4	0.211553	3.8093812	0.2182302	0.6839316
Gtpbp2	0.196844	6.9717936	0.2183402	0.6840775
Asb6	0.2278366	4.7889867	0.2184687	0.6840775
Riox1	-0.1826454	4.9340792	0.2185515	0.6840775
Isg15	-0.3739393	5.2358469	0.2185586	0.6840775
Dmxl1	-0.181807	6.799238	0.2185631	0.6840775
Mogs	0.151761	6.1690569	0.2185933	0.6840775
Gtpbp6	0.2999731	3.3739937	0.2186209	0.6840775
Rbm3	0.1853532	8.7139925	0.2186286	0.6840775
Gsg1l	0.7549448	-0.03695	0.2187356	0.6842406
Gm40356	0.4539065	2.1710459	0.2187737	0.6842406
St3gal4	0.1425095	5.5506653	0.2189701	0.6842406
Gpatch2l	-0.1461292	5.1928859	0.2189867	0.6842406
Lgals12	0.964014	0.1159687	0.2189915	0.6842406
Pop7	0.2339026	3.5542985	0.21905	0.6842406
Mzt1	0.212547	5.834916	0.2190657	0.6842406
Supv3l1	0.2553717	4.111242	0.219078	0.6842406
Pitpnc1	-0.1544576	5.7491461	0.2190808	0.6842406
Nup62	0.1545584	6.4506461	0.2191515	0.6842406
Nudcd3	0.1514958	5.5706675	0.2191646	0.6842406
Arrdc3	-0.2342091	6.3451055	0.2192614	0.6843187
Arhgap26	-0.2273994	4.7810088	0.2192776	0.6843187
Gm9958	0.3805678	1.0419674	0.2194198	0.684625

Tmem231	0.3897219	2.0336993	0.2195386	0.6847521
Cbln3	-0.6337908	-0.809162	0.2195486	0.6847521
Grk6	-0.1991414	7.0839675	0.2196558	0.6849493
Gulp1	-0.4447995	2.0429489	0.2198199	0.6853233
Gm14165	-1.3561147	1.854892	0.2198808	0.6853759
Usp37	-0.1633175	5.6215189	0.2200587	0.6856944
Tmf1	-0.146284	5.6970542	0.2200846	0.6856944
Wdr91	0.1804937	4.9834474	0.2201306	0.6856944
Aup1	0.1286123	6.8546124	0.2202198	0.6856944
Plekhg2	0.2763572	4.6328867	0.2202763	0.6856944
Thg1l	0.2131346	3.9653299	0.2203111	0.6856944
Plaat3	0.2817713	6.4529542	0.2203337	0.6856944
4933424M12Rik	0.8922773	0.5348028	0.220453	0.6856944
Gm36572	0.7159419	-1.0838111	0.2204692	0.6856944
Txk	-0.6336522	0.3146966	0.2204767	0.6856944
E230013L22Rik	-0.6656056	-0.8909545	0.2205086	0.6856944
Pparg	0.2285053	4.0918665	0.220512	0.6856944
Slc12a2	-0.1697605	4.4582104	0.2206606	0.6860193
Cog8	0.1667642	4.5781102	0.220726	0.6860536
Gm52438	-0.6930876	-0.5393687	0.2207598	0.6860536
Abhd6	0.4314266	1.1439167	0.220824	0.6861159
Tpt1-ps3	0.2304625	5.5969667	0.2208836	0.6861642
Nudc-ps1	0.6891403	-0.6304223	0.2209584	0.6862592
Paxip1	0.1690723	6.2476496	0.2211326	0.6865515
F830016B08Rik	-0.3593582	2.6432054	0.2211769	0.6865515
Eif2b1	0.167678	4.9586996	0.2212092	0.6865515
Poglut1	0.1995343	4.3055361	0.221229	0.6865515
Galt	0.247437	3.4536559	0.2212963	0.6866234
Thap6	0.2819385	3.2664712	0.2213754	0.6867319
Taf15	0.1651256	7.2544302	0.2214438	0.6868069
Usp5	0.1288579	6.404825	0.2215175	0.6868674
Rer1	-0.1371765	6.4471719	0.2215882	0.6868674
Tmem37	0.4252534	0.9716854	0.2216368	0.6868674
Ugt1a7c	-0.3489428	3.7841296	0.2216484	0.6868674
Ppm1a	-0.1301657	6.310882	0.2216841	0.6868674
Abhd15	-0.2503555	3.1831614	0.2217821	0.6869992
Emc6	0.1778663	5.3968561	0.221815	0.6869992
Nedd4	-0.1515885	8.6686973	0.2219704	0.687239
Naprt	0.3170501	2.8526726	0.2219808	0.687239
Trim59	0.2219577	7.6237665	0.2220319	0.6872606
Sp3	-0.1500829	8.192878	0.2221154	0.6873822
Asb7	-0.1726103	5.0254097	0.2222291	0.687469
Arl5b	-0.189065	4.5398665	0.2222602	0.687469

Kctd7	0.2012134	4.0269559	0.2223073	0.687469
Zdhhc8	0.2785037	4.0632692	0.2223203	0.687469
C1ra	0.2436734	4.6047582	0.2223788	0.6875135
Serf2	-0.1589315	7.4418028	0.2224505	0.6875983
Llph	0.17449	4.6574195	0.2225987	0.6878051
Cry1	-0.2249663	4.2331879	0.2226058	0.6878051
F420014N23Rik	-0.5131249	-0.7584845	0.2229161	0.6886269
Ube2e3	0.1696815	6.4162405	0.2230769	0.6889868
D130037M23Rik	-0.265574	2.6290915	0.2232388	0.6892789
Zfp711	0.5051359	-0.2374668	0.2232601	0.6892789
Med26	0.1989189	3.6719581	0.2235684	0.6899511
Plxna4os1	0.6424113	-0.9500459	0.2235845	0.6899511
Itpripl1	0.2006845	4.0914226	0.2236109	0.6899511
P4ha2	0.459222	3.1663101	0.2237153	0.6901365
Ccdc117	-0.1982627	5.4213191	0.2238345	0.6903671
Gm10254	-0.3434827	1.0673213	0.2239417	0.6905609
Wasl	-0.1862283	5.3711973	0.2241547	0.6908291
Zfp280d	0.1526623	5.2349572	0.2241581	0.6908291
Mdc1	0.162213	6.7141134	0.2241668	0.6908291
Ager	-0.3640105	1.7730284	0.2242091	0.6908291
Irf9	-0.1718297	5.6748413	0.2242938	0.6908291
Ganab	0.1257535	7.1994264	0.2243778	0.6908291
Stard6	-0.6035737	0.6052754	0.2243824	0.6908291
P2ry10b	0.2662951	2.8495708	0.2243931	0.6908291
Frmd4b	-0.2041832	5.2801554	0.2244284	0.6908291
Abca4	-0.5578502	-0.5160985	0.2245525	0.6910202
Cd209g	-0.6101574	-0.2362939	0.2245794	0.6910202
Padi2	-0.2360201	4.506453	0.2246871	0.6910698
Cry2	0.2488264	3.2530868	0.2246985	0.6910698
Elp2	0.1513247	5.7209219	0.2247288	0.6910698
Spire1	0.2098077	7.0843405	0.2248074	0.6911515
Anapc5	0.1357823	8.0644341	0.2248819	0.6911515
Clint1	-0.1808786	7.8862537	0.2249722	0.6911515
Smco4	0.2827411	3.0711409	0.225019	0.6911515
H2ac13	1.0480226	-0.6079737	0.225032	0.6911515
Lrrc42	0.2189439	3.7026492	0.2250337	0.6911515
Gm36298	0.4003222	0.801741	0.2251095	0.6911515
Zfp791	-0.5812964	-1.0503303	0.2251108	0.6911515
Cep131	0.2545906	3.1068977	0.2252185	0.6913458
Snapc4	0.2007598	3.9409269	0.2253354	0.6914563
Ccdc8	-0.2299509	3.1858318	0.2253465	0.6914563
Gm32208	0.2822975	4.2249762	0.2253879	0.6914563
Fam3a	0.2189172	3.6625143	0.2254373	0.6914714

Washc4	-0.1758962	5.943957	0.2255926	0.6918115
Alkbh7	0.347741	2.6870314	0.2256753	0.6919284
Setd1a	0.1377402	6.1042051	0.2257509	0.6919895
Gm39499	0.6594118	-0.8839099	0.2257842	0.6919895
Tesk1	0.2201335	4.1062435	0.2259657	0.6922829
Slu7	-0.1263599	6.079504	0.2260539	0.6922829
Insl6	0.5348654	0.219004	0.2260713	0.6922829
Slc2a8	0.2836695	2.5585725	0.2261865	0.6922829
Ctnnbip1	0.2459756	3.7081746	0.2261925	0.6922829
Adipor2	-0.1408707	6.908924	0.2262369	0.6922829
Cited2	-0.2649242	6.7790967	0.2262435	0.6922829
Abraxas1	0.2664441	3.6355284	0.2263005	0.6922829
Ormdl3	0.1647067	5.917424	0.2263044	0.6922829
Tuba8	-0.1831006	4.8695921	0.226325	0.6922829
Gm15446	0.5182813	-0.1573151	0.2264963	0.6925071
Gchfr	-0.4711189	0.6849528	0.2265773	0.6925071
Ddx3x	-0.1654451	9.0307944	0.2266413	0.6925071
Acta2	-0.3496459	2.8334273	0.2266547	0.6925071
Dkkl1	0.3148559	2.2276319	0.2266702	0.6925071
Dnaja2	0.1279519	7.1764282	0.2266838	0.6925071
Ap1g2	0.1862798	4.4986454	0.2267616	0.6925071
Ubp1	0.1613057	7.6267227	0.2267733	0.6925071
Gm14176	0.4895549	0.1456542	0.226799	0.6925071
Exoc3l	0.2834108	2.4656448	0.2268816	0.6926234
Cab39	-0.1354515	7.610702	0.2269856	0.6928048
Siah1b	0.2755189	3.793874	0.2272207	0.6933865
Gclc	0.1785943	7.2982974	0.2273117	0.6934946
Scn4a	0.513867	1.7296886	0.2274112	0.6934946
Batf3	0.5788853	-0.9500807	0.2274719	0.6934946
Pikfyve	-0.1587205	5.7494763	0.2274772	0.6934946
Sh2b1	-0.1801934	5.9200883	0.2274791	0.6934946
Tent2	0.1716767	5.4859778	0.2275486	0.6935675
Polg	0.1262868	6.1550567	0.2275922	0.6935675
Pgm2	-0.1396759	5.3188964	0.2276448	0.693592
Tmcc3	-0.2118643	3.6558119	0.2277327	0.693724
Hmg20b	0.1495086	5.1732198	0.2279897	0.6943707
Pabpc1	0.1637362	10.845664	0.2280386	0.6943837
D6Wsu163e	-0.1761099	4.8080979	0.228116	0.6943988
Cyp2u1	-0.7139021	-0.9427897	0.2281328	0.6943988
Gpat4	0.1371997	5.5226871	0.2282111	0.694405
Pik3r3	0.386317	2.981056	0.2282608	0.694405
Trp53	0.1520413	6.3419336	0.2282757	0.694405
Tnpo2	0.133819	6.2267723	0.2283134	0.694405

Asb4	-0.5656937	1.2677136	0.2287255	0.6946045
0610009B22Rik	-0.2344707	3.6685671	0.2287845	0.6946045
LOC118567675	1.0129869	-0.6294724	0.2288055	0.6946045
Chrdl1	0.2048614	5.5130474	0.2288694	0.6946045
Irf3	0.1417812	5.4011141	0.2289046	0.6946045
Frmd3	0.722572	0.2955677	0.2289623	0.6946045
Apbb2	-0.2258106	4.1350663	0.2289767	0.6946045
Vezf1	-0.1544259	6.5348097	0.2289835	0.6946045
Ppm1g	0.1512703	7.4357643	0.2290627	0.6946045
2900005J15Rik	0.8043142	-0.3095341	0.2291618	0.6946045
Msh6	0.1594552	6.6478237	0.2291632	0.6946045
Shd	0.869311	-0.8522515	0.2291664	0.6946045
Fcor	-0.4084668	1.3489717	0.2292087	0.6946045
LOC118568353	1.2032404	-0.2180329	0.2292274	0.6946045
Rpsa	0.2064432	10.554041	0.229341	0.6946045
Mrgpra2b	-0.2610993	4.1266049	0.2293743	0.6946045
Ralb	-0.1446826	5.9767587	0.229395	0.6946045
Rab20	-0.3886358	2.5699757	0.2294617	0.6946045
Siglec15	-0.4091935	3.3350149	0.2294797	0.6946045
Klrb1b	0.6587898	0.7402252	0.2294802	0.6946045
Eif6	0.154544	5.4619593	0.2295209	0.6946045
Polr2e	0.1292756	5.3836256	0.229655	0.6946045
Znrd1as	0.3825711	1.5923246	0.2296592	0.6946045
Nudt13	-0.239091	2.9638113	0.2297783	0.6946045
1110032F04Rik	0.7388295	-0.9901484	0.2298358	0.6946045
Ms4a2	-0.2473862	3.4016439	0.2298911	0.6946045
Krt83	0.4621996	2.0602314	0.2298929	0.6946045
Pld2	0.311157	2.6035779	0.2299474	0.6946045
1700086O06Rik	0.362859	2.3715797	0.2299634	0.6946045
Csad	0.2720485	3.6918044	0.2299897	0.6946045
Gtpbp3	0.1944836	3.5441644	0.2300211	0.6946045
Mars1	0.1568228	5.7809066	0.2300579	0.6946045
LOC118568653	0.5578912	0.5938746	0.2300967	0.6946045
Was	-0.227111	6.4530329	0.2301382	0.6946045
Gm29933	0.4339447	1.3947725	0.2301613	0.6946045
Slc22a3	0.3620879	2.3005999	0.2301643	0.6946045
Msantd2	0.2368752	4.5623705	0.2302392	0.6946045
Gm5539	0.3637926	1.9797392	0.2302492	0.6946045
Zbtb8a	0.444147	1.5783165	0.2302696	0.6946045
Popdc2	0.2852627	2.5821956	0.2303736	0.6946045
Bin3	-0.1839736	5.9082769	0.2303917	0.6946045
Gm34632	0.4751227	1.4388556	0.2304116	0.6946045
Zfp729a	-0.1851601	4.6439848	0.2304253	0.6946045

Ms4a4d	0.3184523	2.0483062	0.2304289	0.6946045
Lpin2	0.1888478	6.9241039	0.2304358	0.6946045
Htra2	0.1598091	6.1199832	0.2304522	0.6946045
Themis2	-0.1751497	5.5146882	0.230478	0.6946045
Mrpl10	0.1512441	4.8612676	0.2306531	0.6949978
Slc28a2b	-0.3237676	3.1226749	0.2307137	0.6950299
6720473M11Rik	1.4319191	-0.2952775	0.2307883	0.6950299
Chn1	0.4616635	4.4055177	0.2308751	0.6950299
Mkks	0.1872418	4.2498489	0.2308826	0.6950299
Iars	0.167525	6.6175889	0.2308872	0.6950299
Gm8797	-0.5064305	0.508091	0.2309389	0.6950511
Gins2	0.2014165	4.524359	0.2310667	0.6953012
Podn	0.4294799	0.8140696	0.2312507	0.6956741
Art2b	-0.6396379	-0.6198074	0.2312801	0.6956741
Nectin1	0.3236657	2.2509969	0.2314098	0.6959296
Tbl1xr1	-0.1538365	6.9193074	0.2315954	0.696353
Pdp2	-0.2382458	3.21889	0.2317519	0.6966771
Pold4	0.3269644	2.5561602	0.2317927	0.6966771
Tmem168	-0.1371463	5.4597786	0.2318523	0.6967215
Cox17	0.2025254	6.2089791	0.2319359	0.6967384
Nsa2-ps1	0.322614	1.2990959	0.2319475	0.6967384
2810410L24Rik	0.5483763	-0.5207358	0.2320033	0.6967714
Npm1	0.1999144	9.6370693	0.2320887	0.6968116
Ppt1	-0.1442065	6.2340767	0.2321063	0.6968116
Rbm48	0.1841514	3.8384639	0.2323808	0.6974406
Tnfrsf1b	-0.1971767	5.8725483	0.2324055	0.6974406
Anapc4	0.1407274	5.5382767	0.2325111	0.6976088
LOC118567660	0.5470081	-0.2356607	0.2326131	0.6976088
Aim2	0.200697	5.245759	0.2326308	0.6976088
Fam171a1	0.2242772	3.7816857	0.2326409	0.6976088
LOC102637806	-0.5690683	-0.2713642	0.2329112	0.6977549
Tor1aip1	-0.1575541	6.5991579	0.2329155	0.6977549
Kif26b	-0.6012269	-0.6580916	0.2329332	0.6977549
Lsm14b	0.1536088	5.1603984	0.2329498	0.6977549
Gm12522	1.0235051	-0.7342317	0.2329978	0.6977549
Vps37b	-0.2002611	5.0565109	0.2329988	0.6977549
Cert1	-0.1673875	6.3409293	0.2330037	0.6977549
Relb	0.3171742	3.2789916	0.2331499	0.6980585
Aco2	0.1320049	7.3077112	0.23326	0.6981981
Mettl17	0.256983	2.9794743	0.2332863	0.6981981
2200002D01Rik	0.4518612	0.6736653	0.233369	0.6983113
Nub1	-0.1274308	6.1329674	0.2334378	0.6983405
Fam98b	0.1509726	5.1850426	0.2335122	0.6983405

Spata5l1	0.2482033	2.8549423	0.2335135	0.6983405
Irx2	0.5384272	-0.1327235	0.2335604	0.6983464
Tcam1	-0.5621364	-0.4944237	0.2336987	0.6986256
Dcp2	-0.1722544	6.0925236	0.2337939	0.6986763
Stard13	-0.3109213	2.4247546	0.2338055	0.6986763
Sipa1	0.1360355	7.2977905	0.2338519	0.6986807
Dolk	0.2058032	3.2539798	0.2339507	0.6988401
Xrcc4	-0.2535818	2.990165	0.2340899	0.6988401
Fbxl12os	-0.6095852	-0.774692	0.2341177	0.6988401
Gm1976	0.7021799	-0.7824958	0.2341386	0.6988401
Rps13	0.1522343	8.2293685	0.2341866	0.6988401
Stox2	-0.2881403	2.9815114	0.2342081	0.6988401
Meaf6	0.164285	5.3047732	0.2342197	0.6988401
Usp28	-0.2194057	3.7295119	0.234293	0.6989245
Casd1	-0.1423386	5.7953783	0.2343496	0.698953
Rnf113a1	-0.3035986	2.0079712	0.234429	0.698953
Mn1	-0.467458	0.3105824	0.2344373	0.698953
Gm46358	0.5066853	1.1722764	0.2345941	0.6989853
Rpl10-ps5	0.500679	-0.4734373	0.2346566	0.6989853
Emc4	-0.1838594	3.9542619	0.2347343	0.6989853
Gprin3	-0.2639596	3.3271793	0.2347382	0.6989853
Ttll1	-0.2574946	3.1574088	0.2348156	0.6989853
Cenpm	0.1893182	4.309697	0.2348368	0.6989853
Agbl5	0.2406416	3.1959083	0.2348462	0.6989853
Rab19	0.3945646	1.0115156	0.2348622	0.6989853
Dok6	-0.751622	-0.7230269	0.2349303	0.6989853
Ptpn1	-0.1762225	6.7305957	0.2349527	0.6989853
Map3k5	-0.1479854	5.7520387	0.2349947	0.6989853
Bri3	-0.1598062	5.1369151	0.2350172	0.6989853
Itm2c	0.1750767	6.450857	0.2350324	0.6989853
Nhej1	-0.2672606	2.6064229	0.2351011	0.699056
Ppp1r18	-0.1729892	7.2271015	0.2352792	0.6994519
Sp100	-0.1692762	7.2634513	0.2353316	0.6994742
Rps24	0.2457673	10.784361	0.2355045	0.6998542
Snx4	-0.1484273	6.2817996	0.2356349	0.7001081
Zfp141	-0.1969071	5.0529471	0.2357107	0.7001451
Zfp873	0.29855	2.3354202	0.2357564	0.7001451
Mlx	-0.1550849	4.9119407	0.2357824	0.7001451
Bpgm	0.3046563	8.9660126	0.2358472	0.7002037
Taco1	0.1909613	3.6225974	0.2361742	0.700444
Zkscan3	0.1524674	5.0731045	0.2361926	0.700444
Vdac1	0.1334075	7.4076731	0.2362151	0.700444
Tbx6	0.4043071	2.1540262	0.2362168	0.700444

Abtb1	-0.1490633	6.2683489	0.23623	0.700444
AA465934	0.4437604	0.4863318	0.2362384	0.700444
Dhx33	0.1568111	4.7460794	0.236276	0.700444
Ep300	-0.1837447	7.6320051	0.2363466	0.700444
Midn	-0.1548628	6.6245206	0.2363734	0.700444
Ogfod3	0.2217833	2.9361774	0.2363785	0.700444
Gm10033	0.3370735	1.5964507	0.2364354	0.7004794
Gtpbp4	0.1364883	5.8604224	0.2365584	0.700638
Gm45700	-0.4177519	1.4622545	0.2365791	0.700638
Txndc16	-0.1813995	4.85629	0.236745	0.7009959
Cables2	0.172168	4.4224085	0.236947	0.7014606
Fam135a	-0.2728524	3.1718315	0.2370412	0.7016058
Gm6863	0.2466501	3.1404574	0.2370958	0.7016338
Scube1	0.5373147	1.3633075	0.2372233	0.7018776
4930480K23Rik	0.3635607	0.6978908	0.237301	0.7019164
2810025M15Rik	0.209351	3.4694825	0.2374107	0.7019164
LOC118567927	0.3893675	0.5931954	0.2374147	0.7019164
Pmaip1	-0.3514473	2.9091847	0.2375007	0.7019164
Prelid3b	0.181031	6.2317862	0.2375436	0.7019164
Echdc2	0.5304636	0.7922213	0.2375844	0.7019164
Med4	0.1613294	4.8548162	0.2377062	0.7019164
Tymp	0.5599402	-0.7014822	0.237749	0.7019164
Gm35535	0.7598896	-1.0595361	0.2377678	0.7019164
Grina	0.1846705	8.4321236	0.2378536	0.7019164
Srd5a3	-0.1806661	4.9441059	0.2379125	0.7019164
Fabp3	-0.7186066	1.5052681	0.2379149	0.7019164
Bcat2	0.146842	5.0675076	0.2379699	0.7019164
Ccdc142	0.2627526	2.8095105	0.2379945	0.7019164
Pin4	0.229972	4.9837524	0.2380974	0.7019164
Dph1	0.3651712	2.0278454	0.2381957	0.7019164
Tmem208	0.190086	4.854338	0.2383131	0.7019164
Sdhc	0.1472142	6.2609868	0.2383922	0.7019164
Havcr2	0.3840938	2.5581931	0.2384476	0.7019164
Tspan2	-0.2085288	4.9870388	0.2384573	0.7019164
Gm11427	0.247778	4.4420724	0.2385303	0.7019164
Trmt13	0.2468001	3.1432038	0.2385514	0.7019164
Fau	0.1947512	9.1005539	0.238619	0.7019164
Stx4a	0.1441308	5.6737048	0.2386483	0.7019164
Meis3	0.3838352	1.8932254	0.2386824	0.7019164
Shc4	0.4607399	1.4231178	0.2386901	0.7019164
Rpap2	0.2571543	3.4136309	0.238694	0.7019164
Sod3	0.4267154	2.7020278	0.238794	0.7019164
B330016D10Rik	0.2697348	2.3371838	0.2388789	0.7019164

Ptk7	0.2749333	3.4771186	0.238894	0.7019164
Ptges3	0.1546825	7.3788009	0.2389479	0.7019164
Klhdc2	0.1629989	5.8694319	0.2389584	0.7019164
Gm26590	0.585967	0.0032201	0.2389871	0.7019164
Gm2629	0.3822893	1.6357534	0.2389989	0.7019164
A230045G11Rik	-0.218867	2.9539598	0.2390749	0.7019164
Zfp729b	-0.1792909	4.3474593	0.2390886	0.7019164
C1galt1c1	-0.1891852	5.0656444	0.2390905	0.7019164
Kdm1b	0.22808	4.5232277	0.2390909	0.7019164
Nt5dc2	0.1500937	5.5918032	0.2391398	0.7019164
Lrrc8d	-0.1706522	5.6618412	0.2391502	0.7019164
Saraf	-0.1461277	5.7547965	0.2391826	0.7019164
Emilin1	-0.1426727	6.5606013	0.2392144	0.7019164
Foxp1	0.2724078	6.4925856	0.2392178	0.7019164
Tlnrd1	0.142143	6.4547272	0.239222	0.7019164
H3f3a-ps1	0.2043159	4.9703633	0.2393183	0.7019912
Tmem35b	-0.2442745	2.3081829	0.2393387	0.7019912
Aplf	0.20897	4.9846959	0.2394035	0.7019912
Nup210	0.1886883	7.6828927	0.239428	0.7019912
Trim10	0.279193	8.081038	0.2395716	0.7020283
Ncapd2	0.1770075	8.0596123	0.2395872	0.7020283
Gm30605	0.3647152	2.6290553	0.2395936	0.7020283
Alg12	0.2069117	3.2820644	0.2396212	0.7020283
Zdhhc4	0.1716737	4.4796525	0.2397991	0.7024172
Gm30679	0.5350555	0.3352354	0.2399221	0.7026453
Clnk	-0.3932402	0.725343	0.2401029	0.7028417
Emc2	-0.1449941	5.7049097	0.2401909	0.7028417
Smim12	0.1998797	3.6739441	0.2402159	0.7028417
Metap2	0.2212528	9.4252543	0.2402584	0.7028417
Gm31657	-0.6510472	-0.3015339	0.2402605	0.7028417
Sez6l2	0.6061809	-0.4181327	0.240324	0.7028417
1700010I14Rik	-0.3621154	2.4827332	0.2403308	0.7028417
Zfp119a	-0.3204268	1.9641723	0.2403737	0.7028417
Pura	-0.1368362	6.2276656	0.240439	0.7028417
Zfp335os	0.3593911	0.6806083	0.2404411	0.7028417
Gm15774	0.5250824	1.0271459	0.2406792	0.7034055
Zfp960	0.7040338	-0.5582465	0.2407958	0.7036142
Abca8b	-0.417585	0.6634917	0.2408962	0.7037668
Csnk1g2	0.1316604	7.0135554	0.2409513	0.7037668
Prox2	-0.3627115	2.4167008	0.2409838	0.7037668
Acbd4	-0.2710869	3.4204884	0.2411857	0.7042243
Gm6767	0.5035032	0.0172702	0.2412317	0.7042263
Tnk2	0.1611418	6.4956991	0.2413596	0.7043804

Eif3d	0.1301462	6.7153866	0.2413751	0.7043804
Fuca1	0.1451743	5.5297251	0.2414882	0.7045783
Hipk2	-0.1642635	7.6763071	0.2415385	0.7045931
C130050O18Rik	0.2436282	3.3230351	0.2418697	0.7050649
1700023D09Rik	0.5530893	-0.3458013	0.2418809	0.7050649
Cd47	-0.142943	8.5597126	0.2418918	0.7050649
Srpk1	0.1313922	6.0371475	0.241901	0.7050649
Dnajc16	0.1868712	4.507433	0.2419269	0.7050649
Dus1l	0.1641393	5.1360619	0.2420219	0.7050721
Med13l	-0.1357028	7.0289873	0.2420387	0.7050721
Hnrnpa2b1	0.1558594	10.555666	0.2420654	0.7050721
Tmem123	-0.1438855	7.5720753	0.2422715	0.7053077
Smpd2	0.2912316	2.8348746	0.242286	0.7053077
Ubash3b	-0.17939	5.8956547	0.2423315	0.7053077
Csrp2	0.2541204	3.7585119	0.2424752	0.7053077
Ndst2	0.167251	5.0772924	0.242491	0.7053077
Garre1	-0.1623343	5.5134233	0.2425283	0.7053077
Il10rb	0.1574583	5.5500434	0.2426277	0.7053077
Rreb1	-0.1384754	6.1430981	0.2426676	0.7053077
Ugt8a	-0.4124022	1.901569	0.2426814	0.7053077
Efcab7	-0.3709758	1.8901638	0.2426868	0.7053077
Golga7	0.128266	6.4159127	0.2427245	0.7053077
Dennd1c	-0.17936	4.9739419	0.2427306	0.7053077
Echs1	-0.1842377	5.1376761	0.2427446	0.7053077
Usb1	-0.1748953	4.0793389	0.2428501	0.7053077
Lipg	-0.414336	3.7623493	0.2430144	0.7053077
Gm10051	0.4009074	0.5146544	0.2430982	0.7053077
Gm10384	-0.531309	1.4486677	0.2431164	0.7053077
Cd300ld4	-0.6080689	-0.2814965	0.2431221	0.7053077
Sppl2b	0.2112391	6.3149592	0.2431415	0.7053077
F13a1	-0.2743836	7.9273469	0.243208	0.7053077
Lmx1b	0.7997421	-1.2369132	0.243242	0.7053077
Panx3	-0.4867945	3.5239211	0.2432519	0.7053077
Uqcrh	0.2300481	7.5606989	0.2432885	0.7053077
9130401M01Rik	0.2308989	3.433828	0.2433217	0.7053077
Gfer	0.2067897	3.9309715	0.2433811	0.7053077
Ccdc138	0.2704933	2.6703317	0.2433815	0.7053077
Flvcr2	-0.451224	1.5849283	0.2434149	0.7053077
LOC118568120	0.5472076	-0.3371228	0.2434492	0.7053077
Gm11675	0.7632774	-1.309905	0.2434737	0.7053077
Igkv6-13	0.5778447	2.4081269	0.2435067	0.7053077
2210016L21Rik	0.1829622	3.9512772	0.24363	0.7055335
Klra8	-0.5643211	0.6454123	0.2436815	0.7055514

Txn2	0.139714	6.4139258	0.2439578	0.7061248
Reep5	0.1349536	6.9658409	0.2439703	0.7061248
Foxd4	-0.4471923	1.0889524	0.2440699	0.7062814
Slc2a4rg-ps	0.3297719	2.4858995	0.244249	0.7063629
Gm6147	-0.6417649	-0.4565956	0.2442539	0.7063629
Gnb4	0.1689748	4.012863	0.2442644	0.7063629
Abhd5	-0.1573782	6.1462166	0.2442797	0.7063629
Pcdhb15	-0.796598	-1.0762739	0.2443529	0.7064434
Mgst3	0.2183777	6.6749554	0.2444522	0.706595
Ankrd6	0.3032352	2.5505951	0.2444962	0.706595
Tbc1d2b	-0.1489255	6.6214599	0.2446091	0.7067899
Proser3	0.3070344	2.6391513	0.2448658	0.7072557
Miga2	0.2633853	3.8648146	0.2449243	0.7072557
Gm15854	-0.5655647	0.1214441	0.2449365	0.7072557
2310015A10Rik	0.2380052	3.4002932	0.244957	0.7072557
Cnbp	0.1861431	8.6178758	0.2449977	0.7072557
AI987944	-0.1726058	3.8828831	0.2450437	0.7072572
Prim1	0.1689259	6.1312637	0.2450893	0.7072577
Dcaf1	0.1576139	5.6768526	0.2451372	0.7072648
Kdsr	-0.178841	4.3581671	0.2452197	0.7073716
Calhm2	0.2316207	3.085387	0.245447	0.7074149
Entpd2	0.5652248	-0.3686133	0.2454474	0.7074149
Serinc1	-0.1565358	7.3998287	0.2454961	0.7074149
Mtf1	-0.1592777	5.395609	0.2455451	0.7074149
Apbb3	0.2733795	2.6275839	0.2455654	0.7074149
Csk	0.1726946	7.4182777	0.245593	0.7074149
Txndc11	0.1781894	5.1312877	0.2457048	0.7074149
Serinc3	0.1452812	10.739902	0.2457761	0.7074149
Tmem198	0.6094902	-0.5786921	0.2457935	0.7074149
Bag5	0.1509526	4.8423431	0.2457968	0.7074149
Agtr1a	0.2946497	4.178103	0.2459225	0.7074149
Rps20	0.1961762	9.181931	0.2459749	0.7074149
Lyar	0.1604187	5.7803353	0.2460972	0.7074149
Sp4	-0.1539867	5.7727463	0.2461209	0.7074149
A630089N07Rik	-0.3545093	2.5091706	0.2461437	0.7074149
Mapre2	-0.172233	6.3914898	0.2461473	0.7074149
Nufip2	-0.1404125	7.2110859	0.2461648	0.7074149
Trmt10c	0.1595187	4.9489252	0.2462374	0.7074149
Kbtbd3	-0.2992202	2.5999992	0.2462389	0.7074149
Chd3	0.2214966	5.8907477	0.2462395	0.7074149
Casp1	-0.173784	4.8010368	0.2464189	0.7074149
Dclk2	0.2171639	4.7765274	0.2464636	0.7074149
Gm30054	0.4897685	1.9105278	0.2464661	0.7074149

Pbdc1	0.1761694	5.9596115	0.2464738	0.7074149
Csnk1g1	-0.167742	4.9188207	0.2464781	0.7074149
Tacc2	-0.2967479	2.8288614	0.2464825	0.7074149
Ap1b1	0.1914924	7.113171	0.2465383	0.7074149
Fam43a	0.1873122	4.8770249	0.246544	0.7074149
Fosb	0.7987119	-0.0250415	0.2465537	0.7074149
Nfrkb	0.1484375	4.6541694	0.2466532	0.7075699
Gm9805	-0.6917343	-0.9034389	0.2468102	0.7078027
Zfp12	-0.2080271	3.8118332	0.2468254	0.7078027
Ccdc50	-0.1440852	6.6195859	0.2468948	0.7078712
Pbx4	0.4894606	0.9934888	0.2470794	0.7081163
Zswim1	0.2453071	2.7164381	0.247104	0.7081163
Tulp4	-0.3430838	4.2212458	0.2471168	0.7081163
Erg	-0.1664749	4.9949934	0.2471646	0.7081227
Smarca2	-0.1483583	6.1469678	0.2472903	0.7083041
BC048403	-0.2479174	2.9900424	0.247319	0.7083041
Trim56	-0.2343812	5.3279354	0.247476	0.7085272
Slc16a12	-0.4181763	0.882485	0.2475582	0.7085272
Ackr4	0.3175875	4.8700718	0.2476018	0.7085272
Chac2	0.4344919	5.3726014	0.2476149	0.7085272
Etnk1	-0.1659147	7.0419834	0.2476566	0.7085272
Dkk1	-0.5841551	3.4991169	0.2476831	0.7085272
4930526I15Rik	-0.3691593	1.5840979	0.2477175	0.7085272
Tbl2	0.1664733	4.9849529	0.2477613	0.7085272
Dhdds	-0.1352568	5.491473	0.2479852	0.7089657
Rmdn1	-0.3174291	2.5860995	0.2480396	0.7089657
Ptges3-ps	0.226607	3.3402279	0.2480514	0.7089657
Srgap3	-0.2683406	4.2145148	0.2481276	0.7089783
Mllt6	0.2545552	5.0431672	0.248147	0.7089783
Alas2	0.3136684	10.388083	0.2483699	0.7093687
Ipo4	0.1917511	4.3098747	0.2484425	0.7093687
Zbtb43	-0.1587068	4.2781522	0.2484479	0.7093687
Crk	-0.131984	6.7271656	0.2485003	0.7093687
Ralgps1	-0.2337657	3.3596297	0.248548	0.7093687
Ndufb10	0.1411489	6.5657168	0.2485572	0.7093687
LOC118567635	0.5064846	-0.2548634	0.2487333	0.7096341
Med7	0.2139654	5.5274396	0.2487415	0.7096341
Ssbp4	0.1395859	5.6754636	0.2488294	0.7097546
Ulbp1	-0.3844211	2.3547726	0.24888	0.709769
Elmo3	0.3387827	2.5872421	0.248968	0.7098898
Odf2	0.1553683	7.0414821	0.2491083	0.710116
Fstl3	-0.351659	1.657094	0.2491595	0.710116
Rpl18a	0.2070591	9.9758961	0.2491843	0.710116

Rassf1	0.1617492	4.6191849	0.2493175	0.7102416
Trmt10b	0.2980411	2.4154353	0.2493197	0.7102416
Gm4332	0.3416704	2.3071667	0.2495151	0.7104483
Pnpla6	0.1786894	5.1623547	0.249571	0.7104483
Btf3l4	0.1719161	4.8572357	0.2496117	0.7104483
Txndc12	-0.1731438	5.0431361	0.2496175	0.7104483
Pcgf1	0.3049861	2.6905383	0.2496207	0.7104483
Uqcrfs1	0.1602536	6.3245416	0.2497738	0.7105684
Dynlt1b	-0.2547168	2.8192438	0.2497748	0.7105684
Edil3	-0.3567592	3.3175545	0.2498167	0.7105684
Macf1	-0.1489735	8.0784841	0.2498456	0.7105684
Arhgef25	0.2603123	3.1871088	0.2498947	0.710578
Dlgap4	-0.1433584	5.3710119	0.250209	0.7113417
Muc6	-0.6694172	-0.253135	0.2505037	0.7119668
Zcchc24	0.2018249	5.3395066	0.2505888	0.7119668
Fbxo17	0.3676672	1.3816353	0.2506251	0.7119668
Gm42266	0.3216247	1.8784946	0.2506271	0.7119668
Psmg2	0.1947483	4.6550991	0.2507566	0.7119668
Snx29	0.2313275	4.3573581	0.2507873	0.7119668
AW046200	-0.7331015	-1.0950969	0.2508672	0.7119668
Gm7785	0.4086022	0.8799194	0.2508926	0.7119668
Mtap	0.1582586	4.2077349	0.250938	0.7119668
Rptor	0.1670265	4.8487267	0.2509895	0.7119668
Vav2	0.1740829	4.9859603	0.2510173	0.7119668
Gm31024	0.8393072	-1.0542531	0.2510317	0.7119668
Kansl1	-0.1894435	6.303221	0.251044	0.7119668
Ccl5	-0.3342544	2.715318	0.251109	0.7119668
Itga9	0.1874846	4.3337722	0.2511155	0.7119668
Taf5	0.1844172	4.5471036	0.2511631	0.711972
Plin2	-0.1744599	6.5737531	0.2513146	0.7122718
Pde2a	0.2014127	7.1367324	0.2514588	0.7124655
Mcub	0.27424	2.3797145	0.2515398	0.7124655
Mcph1	0.1901585	5.2179515	0.2517003	0.7124655
Tspyl5	0.5819124	-0.9084474	0.251712	0.7124655
ND3	-0.443762	0.8220271	0.2517219	0.7124655
Lypd10	-0.6879372	-0.4095938	0.2517779	0.7124655
Ctsc	0.1394402	7.102619	0.2518005	0.7124655
Timm10	0.2680936	3.3861752	0.2518279	0.7124655
Nolc1	0.1681562	6.8614791	0.2518389	0.7124655
Cdk16	0.1357869	6.0980024	0.251841	0.7124655
Ccni	0.1380089	7.8567951	0.2519714	0.7125789
Ndufaf4	0.1961842	4.2807648	0.2519824	0.7125789
Chchd4	0.2424928	3.7407571	0.2520186	0.7125789

LOC118567463	-0.5782751	-1.0366322	0.2522828	0.7131965
9330151L19Rik	0.2968991	2.9712848	0.2524701	0.7135962
Lacc1	0.3360861	1.8530529	0.252531	0.7136387
Fibin	0.361269	3.6000443	0.2526361	0.7137498
Prickle1	-0.2605615	3.4246395	0.2526621	0.7137498
Trim45	-0.2728198	2.6404662	0.25286	0.7141791
Adar	-0.1405516	5.9388735	0.2529426	0.7141976
Gm45972	-0.6193197	-0.8504105	0.2529584	0.7141976
Zfp668	0.182019	3.8026712	0.2530095	0.7142124
Zfp445	-0.1335101	6.8946247	0.2531143	0.7143501
Redrum	0.2496064	4.8265236	0.2531502	0.7143501
Abcc4	-0.1993242	5.5493797	0.253207	0.714381
C920006O11Rik	0.9172766	-0.4506947	0.253432	0.7147646
Shox2	-0.3683658	4.5455315	0.2534395	0.7147646
Mrgpra2a	-0.322024	2.078594	0.2534822	0.7147646
Nek9	-0.1418056	6.4586281	0.253635	0.7147646
Lockd	0.257303	4.9389973	0.2537271	0.7147646
Nlrc5	0.1662155	4.2952148	0.2537324	0.7147646
Plk2	-0.3453072	2.5296842	0.2537344	0.7147646
Ube2n-ps1	0.326095	1.1791668	0.2537537	0.7147646
Map4k3	-0.2176136	4.8391931	0.2537566	0.7147646
2810408I11Rik	0.3055788	3.0375118	0.2538325	0.714849
Nfs1	0.1669745	5.0950112	0.2539283	0.7148956
Gm3052	0.5039527	1.0027803	0.2539964	0.7148956
Ddit4	0.5252797	4.8499688	0.2540502	0.7148956
Oasl2	-0.2638143	4.5771194	0.2540672	0.7148956
Slc5a11	-0.4902096	-0.1756286	0.2541572	0.7148956
Rab11b-ps2	-0.5733401	0.5357403	0.2543251	0.7148956
Wdr70	0.1708153	4.3042965	0.254354	0.7148956
Rnf44	-0.1303782	6.7066123	0.254377	0.7148956
Ar	-0.2163702	3.0790926	0.254443	0.7148956
Col10a1	-0.9800157	7.0621591	0.2544581	0.7148956
Dynll2	-0.1397944	6.2268354	0.2545357	0.7148956
Bcl7b	0.1330922	5.0804431	0.2545419	0.7148956
Pigc	0.1623296	4.3258293	0.2545836	0.7148956
Cnr2	-0.1941437	5.1447723	0.2546002	0.7148956
9130019P16Rik	-0.5487687	0.622725	0.2546174	0.7148956
Sema4c	-0.2466653	2.8354982	0.2546178	0.7148956
Brap	0.1320129	5.9375208	0.2546501	0.7148956
Stat5b	-0.1550422	6.4452675	0.254697	0.7148956
Tpst2	-0.1353375	5.5623137	0.2547224	0.7148956
Ergic2	0.1507182	6.3813027	0.2549629	0.7149411
Nphp3	-0.2764172	2.2884665	0.2549629	0.7149411

Mrps9	0.1566767	5.3110003	0.255	0.7149411
Car13	0.222115	2.8297979	0.2550104	0.7149411
Tedc1	0.2153425	3.9102959	0.255012	0.7149411
Snca	0.3233594	8.3497698	0.2550143	0.7149411
Wdr78	-0.3527149	1.221494	0.2552914	0.7155356
Oas2	-0.2631249	3.1689452	0.2553184	0.7155356
Edc3	0.1414431	5.2323712	0.2553805	0.7155806
1700028E10Rik	0.6275905	-0.2707647	0.2554528	0.7155812
Slfn9	-0.2078657	4.6895389	0.2554753	0.7155812
Gm46139	-0.3780252	2.1835798	0.2555667	0.7155812
B4galt7	0.1827473	3.5916293	0.2556386	0.7155812
Gm14418	0.6273213	-0.132119	0.2556643	0.7155812
Amer2	0.3812676	3.2703261	0.2556849	0.7155812
Polr1a	-0.1865684	5.507002	0.2557027	0.7155812
Gjb3	0.3820417	1.5408544	0.2560141	0.7163238
Naif1	-0.3619477	1.9496271	0.2561307	0.7164978
Slc36a2	0.3661034	4.8013614	0.2561932	0.7164978
Tbcel	0.1837723	6.6586888	0.256243	0.7164978
Atpaf1	-0.2384512	3.202498	0.2562619	0.7164978
Il17ra	0.1556331	7.2290753	0.2563167	0.7164978
Pik3cg	-0.1660603	6.9862969	0.2564228	0.7164978
Hcls1	-0.1807055	7.504427	0.2564267	0.7164978
Gm7600	0.5393952	0.3832374	0.2564449	0.7164978
Lig1	0.1649372	7.6182964	0.2566571	0.7169619
Fbxl5	-0.1685055	6.640306	0.2568261	0.7172789
Rnf123	0.2121612	7.6104518	0.2568628	0.7172789
Cyld	-0.1291449	5.9889841	0.2569779	0.7174717
Slc9a5	0.2968082	2.9424972	0.2570477	0.7175085
Psmg1	0.2058124	3.8003338	0.2570899	0.7175085
Myo15	0.5901777	-0.2313897	0.2571295	0.7175085
Them6	0.3421701	3.0799487	0.257202	0.717582
Degs2	0.6174697	-0.9711737	0.2576122	0.7185482
Ciao2a	0.1667639	6.834631	0.2576407	0.7185482
Abi1	-0.130658	7.1769955	0.2576871	0.7185488
Klhl18	-0.1528023	4.6045331	0.2578785	0.7189535
Mkrn1	0.2400141	9.0361122	0.2580674	0.719182
1700025G04Rik	-0.1890322	4.2016107	0.2580938	0.719182
Tmem273	-0.2104147	3.4917533	0.2580991	0.719182
2900026A02Rik	0.2236045	4.0164059	0.2582673	0.7193997
Sgms1	-0.1546871	5.2363905	0.2583666	0.7193997
Gm47814	0.4383547	0.6731676	0.2583974	0.7193997
Arhgef37	-0.4321934	3.5064008	0.2584477	0.7193997
Acly	-0.1707514	7.147895	0.2584508	0.7193997

BC031361	-0.551703	0.5893751	0.2584548	0.7193997
Gm35365	-0.7287498	-1.2569345	0.2585476	0.7195002
Cyp4f16	0.2343402	2.9363851	0.2585834	0.7195002
Larp4b	-0.1354795	7.7992265	0.258632	0.7195068
Ppp2r3a	-0.2307507	3.1529254	0.2587193	0.7195874
Npas4	-0.4550229	1.9433995	0.2587535	0.7195874
Scamp4	0.1837947	4.0452655	0.2590875	0.7199774
Tec	-0.1956119	4.8542712	0.2591008	0.7199774
Col5a3	0.3280961	4.2768601	0.2591458	0.7199774
Frmd8os	-0.6255012	-0.6890053	0.2591508	0.7199774
Zfand6	0.2162671	6.8607665	0.2591522	0.7199774
Tepsin	0.221836	3.3312633	0.2591715	0.7199774
Dph5	0.2495302	3.4714589	0.2593034	0.7199941
Ccdc190	0.8442126	-0.9069564	0.2593517	0.7199941
Naa30	0.1318984	5.5325528	0.2593628	0.7199941
Ranbp3	0.1318342	5.9776584	0.2594055	0.7199941
Rgma	0.4349996	-0.1007631	0.259409	0.7199941
Dhrsx	0.1554212	4.551316	0.2596145	0.720436
Gm52391	0.5345608	0.6167981	0.2597674	0.7206308
Gpx7	0.4369965	2.7903657	0.2598053	0.7206308
P4htm	-0.6126809	-0.2609743	0.2598424	0.7206308
Setd2	-0.1667561	7.2065576	0.2598981	0.7206308
Rasgrp3	0.237585	4.0492368	0.2599585	0.7206308
Eln	0.393478	2.4176329	0.2599627	0.7206308
Pip4k2c	0.1317805	6.034513	0.260061	0.7207749
A430005L14Rik	0.174779	5.2005103	0.2601145	0.7207946
Stxbp1	0.1980684	3.565241	0.2601786	0.7208439
Cxcr5	0.4744491	3.3905103	0.2602593	0.7209104
Hint1	0.2244475	7.0523859	0.2603093	0.7209104
Eef1a1	0.1362342	12.233413	0.2603416	0.7209104
Nme2	0.162237	6.5079397	0.2603944	0.7209281
Asxl1	-0.1291269	6.5562978	0.2605211	0.7211506
LOC118568401	-0.5729253	-0.7319596	0.2606091	0.7211681
Ago4	-0.2391411	3.564418	0.2606609	0.7211681
Rnd3	0.1746843	4.7225352	0.2606665	0.7211681
AA414768	0.5504279	0.6842294	0.2609357	0.7217265
Tmed8	-0.1609652	4.955628	0.2610034	0.7217265
Gm33804	0.5636812	-0.5218874	0.2611324	0.7217265
Lgals1	0.1693283	7.5369489	0.2611558	0.7217265
Rpl7-ps7	0.4449817	0.8210973	0.2611629	0.7217265
Pam	-0.1455233	5.5584592	0.2612103	0.7217265
Dapp1	-0.1568176	6.1194171	0.2612543	0.7217265
Gm50217	-0.4856033	-0.1510776	0.2613	0.7217265

Btnl9	-0.3157459	2.7288251	0.2613256	0.7217265
Ndufaf7	0.1968994	3.7807556	0.2613324	0.7217265
Cgref1	0.5305166	5.0835374	0.2614567	0.7219415
Gm13077	0.683029	-0.5545296	0.2615258	0.7219561
Gcat	0.4893735	0.6646577	0.2615634	0.7219561
Kdm4b	0.2027018	4.3818412	0.2616076	0.7219561
Bdp1	-0.149695	6.4127209	0.2616815	0.7219561
Psmb1	0.1630723	7.3757728	0.2616975	0.7219561
Cdh5	-0.2203318	5.6582328	0.2617444	0.7219561
Cluap1	0.1849947	4.0217891	0.2617868	0.7219561
Ccr6	0.4319421	1.9487229	0.261928	0.7219909
Cd3eap	0.1931174	3.5820238	0.261931	0.7219909
Atg5	-0.1311887	5.158358	0.2619387	0.7219909
Iqsec1	-0.1671601	6.7447544	0.26201	0.7220595
Brsk1	0.5057977	0.8764594	0.2621653	0.7223362
Stc2	-0.662829	0.6740687	0.262279	0.7223362
Bend5	0.3885809	1.7173897	0.2622925	0.7223362
Mrpl21	0.1420094	4.7795683	0.2623439	0.7223362
Morc3	-0.1294923	6.548541	0.2623687	0.7223362
Flcn	-0.1586542	4.9764175	0.2623891	0.7223362
Sh2d2a	0.3461982	1.1933753	0.2625233	0.722426
Cct8	0.1557288	8.1215726	0.2625281	0.722426
Ccnt1	-0.12814	6.4063517	0.262561	0.722426
Trappc2	-0.1771307	4.1726017	0.2626893	0.7226512
Ak3	0.1128458	5.9643851	0.2627871	0.7227195
Ivns1abp	0.1419118	8.0632257	0.2628071	0.7227195
Arhgef39	0.1639292	4.8254325	0.2629349	0.7229431
Plau	-0.1563548	4.185171	0.2630756	0.7231051
Bet1	-0.1711476	5.1374933	0.2630891	0.7231051
Rai14	0.217625	5.1261699	0.2631333	0.7231051
Chchd5	0.244799	3.0736621	0.2632787	0.7233768
Zswim7	0.2810668	1.7453202	0.2634099	0.7236095
Comp	-0.4146273	5.3987303	0.26357	0.7238264
Haus3	0.1289231	5.7667563	0.2635866	0.7238264
Lsg1	0.139757	4.9633825	0.2637317	0.7238264
H2-Q10	-0.4719613	3.9947329	0.2637388	0.7238264
Necab3	-0.4676392	0.0636837	0.2637783	0.7238264
Adcy1	0.6535411	-0.7871095	0.263832	0.7238264
Mlh3	-0.1826183	3.7098095	0.2638665	0.7238264
Arhgef28	-0.356246	0.9227676	0.263901	0.7238264
Sardh	-0.2164423	3.2833089	0.2640082	0.7238264
Tjp2	-0.1856274	3.9081612	0.2640818	0.7238264
Rtn4	-0.1330499	7.1119226	0.2640871	0.7238264

Rnasel	-0.1472409	6.4889842	0.2640922	0.7238264
Ddi2	-0.2390505	4.9305733	0.2641891	0.7238264
Gm32006	-0.5133331	-0.7545945	0.2642165	0.7238264
Mtx1	0.1526366	4.3614387	0.2642323	0.7238264
Tox2	-0.5643479	-0.5752746	0.2642334	0.7238264
Usp31	-0.184617	4.7644694	0.2644113	0.7241862
Cd300c	-0.6376934	-0.7038734	0.2645112	0.7242754
Emc7	-0.1350979	5.5755475	0.2645855	0.7242754
Nenf	0.311607	4.5003194	0.2646695	0.7242754
Gm15710	0.4092887	0.4299891	0.264675	0.7242754
Grasp	0.3464992	2.0456823	0.2646767	0.7242754
Rab9	0.159155	4.8655412	0.2652124	0.7255033
Egr2	-0.4938146	0.6280188	0.2652514	0.7255033
Tfb1m	0.2369879	2.8669288	0.2652653	0.7255033
Wdr34	0.2781883	2.375407	0.2653236	0.7255134
Pdpk1	-0.1334896	6.7673278	0.2654095	0.7255134
Usp24	-0.1494246	6.9699975	0.2655025	0.7255134
Smad6	0.3515509	3.4645561	0.2655739	0.7255134
Krt7	0.906593	-0.8453937	0.2656693	0.7255134
Mib2	-0.1637649	4.2088665	0.2656871	0.7255134
Cpox	0.2364884	9.0862607	0.2656951	0.7255134
Snrpb2	0.1743487	6.4180114	0.2657638	0.7255134
Eif1b	0.1434503	5.6199863	0.2658054	0.7255134
Bgn	0.2805591	9.2600664	0.2659322	0.7255134
Apip	0.1759709	4.7887814	0.2659796	0.7255134
Traf5	0.1952009	3.9752339	0.266026	0.7255134
Usp49	0.2808134	2.620433	0.2660696	0.7255134
Dnajb6-ps	0.5531499	0.0571529	0.2660795	0.7255134
Lpl	0.3084969	7.60253	0.2660981	0.7255134
Dcc	-0.7388541	-0.2356958	0.2661335	0.7255134
Gm7809	-0.5708609	0.2124917	0.2661645	0.7255134
Rassf6	0.5235875	-0.8907529	0.266196	0.7255134
Rpl38-ps2	0.3673918	2.4699696	0.2662426	0.7255134
Ripor3	-0.2368318	2.8737645	0.2662516	0.7255134
Rad23b	0.1195091	7.2793702	0.2662653	0.7255134
Pcdhac2	-0.8147686	0.2586847	0.2662952	0.7255134
Nxt1	0.1685017	5.0348873	0.266454	0.7257169
Gstm6	0.9379938	-1.3102552	0.2664632	0.7257169
Pih1d1	0.1829298	4.8083736	0.2666112	0.7257845
Spc24	0.1377432	5.7419734	0.2666268	0.7257845
Oard1	0.2128559	5.4073507	0.2666725	0.7257845
Septin11	-0.175827	6.6987124	0.2666784	0.7257845
Mak16	0.1601601	5.1845846	0.2667214	0.7257845

Gm9843	0.2256719	4.2807554	0.2669178	0.7259912
Crip1	0.2292766	5.785787	0.2669274	0.7259912
Coq2	0.1396701	4.7629314	0.2669826	0.7259912
Bcam	-0.3278282	1.679746	0.266984	0.7259912
Ubxn1	0.1244454	6.6377292	0.2670753	0.7261124
Fip1l1	0.1249228	6.7192108	0.2671643	0.7262276
Trim62	0.3073778	1.0354561	0.267253	0.7263417
Bcas1	-0.4497367	-0.0984413	0.2673546	0.7264909
Zfp708	-0.3345808	1.7340626	0.2675416	0.7265675
Col1a1	0.4066506	13.758602	0.2675647	0.7265675
Pcdhga7	-0.6068377	-0.4169331	0.2675732	0.7265675
1600020E01Rik	0.3943287	2.499433	0.2676139	0.7265675
Gm6467	-0.5796393	-0.5946465	0.2676164	0.7265675
Adamts6	0.3095377	2.184196	0.2677075	0.7265677
Lgmn	0.1450988	6.5273584	0.2677099	0.7265677
Topors	0.1258059	5.860146	0.2678085	0.7267087
Cidec	0.6788317	2.8903504	0.2678983	0.7267138
Gm46272	-0.5163376	0.0338525	0.2679302	0.7267138
Brf1	0.1509917	6.0259397	0.2680474	0.7267138
Tcta	-0.234165	3.1742683	0.2680685	0.7267138
Pheta2	0.2134717	5.4839159	0.2680825	0.7267138
Sod1	0.141441	8.009159	0.2680908	0.7267138
Gm11335	0.619921	-0.4465649	0.2682221	0.7268196
Zfp667	0.3091343	1.9949552	0.2682233	0.7268196
Klhl2	-0.154011	4.8767881	0.2683023	0.7269072
Abhd10	0.1813992	4.0665621	0.2684777	0.7272557
Magt1	-0.134716	6.4817719	0.2685291	0.7272683
Trap1	0.1390706	6.1809562	0.2686082	0.7273559
Prrg4	0.5397361	0.9420703	0.2686828	0.7273695
Ctsh	-0.1395248	6.9889176	0.2687467	0.7273695
Nr3c2	-0.4121169	0.6922954	0.2687536	0.7273695
Fam189b	0.2195035	2.7767199	0.2690872	0.7280929
Hspa12b	0.3744587	1.8389824	0.2691144	0.7280929
Atg4b	0.1573768	5.8709133	0.2692979	0.7282971
Fam78a	0.149549	5.6889467	0.2693571	0.7282971
Pkp3	0.3553068	1.525469	0.2693704	0.7282971
Gm8524	0.2904411	2.0028264	0.2694078	0.7282971
Gm5124	-0.6277889	-0.491782	0.2694241	0.7282971
Acaa2	0.149358	5.5001022	0.2695171	0.728422
Calu	-0.1709825	7.709472	0.2696533	0.7286636
Naa80	0.1774321	4.1246511	0.2697654	0.7288399
Spn	-0.1838419	7.7071751	0.2698883	0.7290454
Cby1	0.187224	4.4429795	0.270035	0.7291295

Apln	-0.6562841	0.0410748	0.2700532	0.7291295
Zfpm1	0.2060509	8.0325602	0.2700601	0.7291295
Zfc3h1	-0.142976	6.6009894	0.2702695	0.7295042
Gm8369	0.4551318	0.6519435	0.2702927	0.7295042
Fxn	0.1610128	4.6483861	0.2703779	0.729536
Serpinb6b	0.1854528	3.7470203	0.2704209	0.729536
Ace	-0.353125	3.2139559	0.2704452	0.729536
Exosc8	0.1646747	5.9156459	0.2706399	0.7296035
Bsg	0.1948715	8.9247902	0.2706437	0.7296035
Llgl2	-0.2972938	1.7597651	0.2706586	0.7296035
Lama1	0.4985557	1.1818981	0.2706845	0.7296035
Gm6061	0.3671171	1.1560192	0.2707141	0.7296035
Tmed1	0.2003776	3.4117799	0.2707517	0.7296035
Sla	-0.2221296	7.5160027	0.2709228	0.7297427
Cmah	-0.1605989	6.3255186	0.2709555	0.7297427
Rps15a-ps7	0.3687628	1.5961052	0.270956	0.7297427
Ikzf1	0.1618644	8.3361975	0.2710345	0.7297427
Spic	-0.2702357	4.0319683	0.2710489	0.7297427
C5ar2	-0.2435312	3.6900879	0.2712	0.7297427
Slc29a2	0.6559884	0.1439222	0.2713103	0.7297427
Dlx6	0.4546328	0.6908763	0.2713345	0.7297427
Cytip	-0.2176189	6.6774306	0.2713607	0.7297427
Acan	-0.7575492	5.0833952	0.2714151	0.7297427
Tpra1	0.1650561	4.8519083	0.2714291	0.7297427
Psmd3	0.14205	6.7231212	0.2715028	0.7297427
Ywhaq-ps3	-0.3414236	1.5485153	0.2715075	0.7297427
Tank	-0.1237754	6.0540599	0.2715126	0.7297427
Txnl4b	-0.1830779	4.015323	0.2715331	0.7297427
AI504432	-0.1951142	5.7530663	0.2717155	0.7297427
Gm14295	0.2408672	2.6489828	0.2717836	0.7297427
Ubxn7	-0.1238376	6.4532386	0.2717964	0.7297427
Slc6a9	0.2104571	6.5555375	0.2718867	0.7297427
Bmi1	0.1183559	5.579919	0.2719222	0.7297427
Nckap1	-0.1440505	5.588848	0.2719976	0.7297427
Cnst	-0.1523239	5.6898871	0.2720434	0.7297427
Irf2bp1	0.1377321	5.2753336	0.2720478	0.7297427
Cxcl9	-0.3429017	3.0567149	0.2720684	0.7297427
Stamos	-0.517016	-0.7529257	0.2720798	0.7297427
Prpf19	0.1256631	6.8091742	0.2720994	0.7297427
Harbi1	-0.2272317	2.6005334	0.2721503	0.7297427
Dcaf13	0.1658046	5.3760807	0.272233	0.7297427
Zbtb4	0.1721085	4.5990058	0.2722973	0.7297427
Parl	0.1562796	5.4520503	0.2723055	0.7297427

Zbtb38	-0.1296917	5.2184588	0.272351	0.7297427
Zmat2	0.1595139	6.8859726	0.2724071	0.7297427
Cd8a	0.5537646	1.1983306	0.2724685	0.7297427
Trappc4	0.1550487	4.7937655	0.2724797	0.7297427
Nrip3	0.3074775	3.4506679	0.2726773	0.7297427
Pex16	0.184869	3.6541244	0.2727131	0.7297427
Cog2	-0.156126	4.4626994	0.2727893	0.7297427
Ube2f	0.1455184	5.9540062	0.2728226	0.7297427
Niban2	0.1716563	5.0801354	0.272826	0.7297427
Rpl17-ps10	0.2432128	2.5118851	0.2728647	0.7297427
Clcn3	0.2321462	7.4116805	0.272887	0.7297427
Champ1	0.1548856	4.7453967	0.2728883	0.7297427
Trpc1	-0.4598798	0.779842	0.2729204	0.7297427
Med25	0.1440412	5.1342629	0.2729549	0.7297427
Nmt2	0.153742	4.6499127	0.2729804	0.7297427
Med16	0.1427747	4.8826388	0.2731504	0.7297427
Zfp7	0.2178201	2.8677233	0.2731885	0.7297427
Keap1	0.1115313	5.8808935	0.2732017	0.7297427
Arfgap2	0.1242641	6.109235	0.2732168	0.7297427
Phkg2	0.1560051	5.11332	0.2732961	0.7297427
Cd101	-0.2207479	5.2805208	0.2733052	0.7297427
Tmem255a	0.4964898	0.8205781	0.2733316	0.7297427
Dctn6	0.138455	5.4421455	0.2734285	0.7297427
Inpp4b	-0.1805781	4.0245362	0.2734654	0.7297427
Prr5	0.2730578	2.8690121	0.2735341	0.7297427
Fanca	0.1479171	4.7910576	0.2735461	0.7297427
Fbrsl1	0.1505927	5.0026452	0.2735462	0.7297427
Stxbp3	-0.1180691	5.8029934	0.2735641	0.7297427
Prkn	-0.566252	0.1828335	0.2735714	0.7297427
Ywhae	0.1303568	8.9490275	0.2736286	0.72977
C1s2	0.4448383	1.1550351	0.2738241	0.7301662
1700027J07Rik	0.7600639	-0.1003807	0.2739	0.7302436
S1pr1	0.2043913	5.092635	0.2739838	0.7303416
Pts	-0.1946055	4.9929995	0.2741181	0.7304822
Dzank1	0.7536687	-1.0749194	0.2741304	0.7304822
Hmgcr	-0.1709669	5.9377514	0.2741847	0.7305016
Osr1	-0.5135235	0.7819665	0.274238	0.7305186
Psme2b	-0.2871533	2.3197006	0.2746943	0.7314311
Gm28857	0.4983843	1.5402975	0.2747352	0.7314311
Ahr	-0.2781645	2.7659045	0.2747775	0.7314311
Sirpa	-0.1340893	8.2257905	0.2748425	0.7314311
Clec10a	0.4809082	2.6690251	0.2748554	0.7314311
Gm12918	0.2654704	2.297637	0.2748627	0.7314311

Blcap	0.1609792	4.1152396	0.2749475	0.7315317
Ssb	0.1734873	8.1728383	0.2750284	0.7316216
Susd5	-0.5125465	2.7276138	0.2751046	0.7316992
Tsg101	0.1193333	5.5431011	0.2752223	0.7318718
Adcy5	-0.286459	3.0125615	0.2752636	0.7318718
Unc13b	0.4664055	0.9593776	0.2754085	0.7320501
Sqle	-0.228468	4.2864389	0.2754248	0.7320501
Hoxa3	-0.3791006	1.2700317	0.2755804	0.732229
Rbm5	0.1374418	7.0272932	0.2755863	0.732229
LOC115488525	-0.4177276	0.190827	0.2757205	0.7324604
Chmp5	-0.1313986	6.1765145	0.2758735	0.7327417
Trmt61b	0.4728856	1.3792852	0.275991	0.7329235
Pcolce	0.3482996	5.6558026	0.2760361	0.7329235
Cfh	-0.2746771	8.3293465	0.2761199	0.7329835
Tcerg1	0.1459375	6.8164662	0.2761822	0.7329835
Urod	0.2179868	7.545363	0.2762001	0.7329835
5330438D12Rik	-0.3977361	0.6426503	0.2764622	0.7333862
Tgs1	-0.1429934	5.5524279	0.2765085	0.7333862
Egr1	0.3298512	3.8211208	0.2765739	0.7333862
Zfp286	0.4981614	0.3370613	0.2765743	0.7333862
Kctd15	0.5157439	1.4863349	0.2765876	0.7333862
Golga2	-0.1259963	6.1930085	0.2766834	0.7335151
Scn7a	0.4397221	-0.127808	0.2768249	0.7336737
Nod2	-0.3893168	1.1317977	0.2768375	0.7336737
Prps1l1	-0.6708279	-1.0335409	0.2769908	0.7338685
Ncbp2	0.126098	5.6138746	0.2770259	0.7338685
Tacr1	0.828029	-1.0001884	0.2770776	0.7338685
Slc13a3	0.4379833	0.7256867	0.2770998	0.7338685
Rcn1	0.2417959	5.8572663	0.277183	0.7339641
Zfp26	-0.1449048	5.2056599	0.2772859	0.7341116
Dmpk	0.2522935	4.2785819	0.2775442	0.7344554
Gm38503	0.7307853	-0.814097	0.2775624	0.7344554
Pigu	0.147381	4.4675272	0.2775686	0.7344554
Tradd	0.1699789	3.9532813	0.2776817	0.7344554
Ube2a	0.1273187	5.2203196	0.2777007	0.7344554
Micall1	-0.1558969	5.004582	0.2778517	0.7344554
Synj1	-0.1254051	6.5025473	0.2779597	0.7344554
Gpr180	0.2360081	3.4861429	0.2780916	0.7344554
Gtpbp8	-0.2438673	3.1445161	0.2781043	0.7344554
Zeb2os	0.1900332	3.7630847	0.2781258	0.7344554
AI597479	-0.16186	4.276343	0.278135	0.7344554
Srp19	0.1747018	5.9836559	0.2781449	0.7344554
Ddah1	-0.6829937	-0.7394502	0.2781485	0.7344554

Gprc5b	0.3875704	2.1086853	0.278235	0.7344554
Mrs2	0.1298372	5.9778613	0.2782484	0.7344554
Vgll3	-0.7125646	-0.2857688	0.2782874	0.7344554
Ccdc85b	0.2188661	3.9869797	0.278311	0.7344554
Lgr6	-0.5175424	1.5718105	0.27843	0.7344554
Vps13b	-0.1733223	6.6105838	0.278434	0.7344554
Ptprd	-0.2634757	6.3392669	0.27844	0.7344554
Mrfap1	0.1256115	7.693541	0.2784423	0.7344554
Pfkm	0.182731	5.3411738	0.2784546	0.7344554
Akt3	-0.172884	5.9477008	0.2786012	0.7347174
Fastk	0.1679522	5.0887381	0.2787999	0.7349064
Gm10353	-0.2774755	1.8910377	0.2788486	0.7349064
4930556M19Rik	-0.4442461	0.3328166	0.2788766	0.7349064
Foxk1	-0.1623823	5.9834742	0.2789046	0.7349064
Flvcr1	-0.1478973	4.8528232	0.2789257	0.7349064
Pcdhb19	-0.4569642	-0.3156658	0.2789992	0.7349064
Aqp11	0.3649304	0.7394944	0.2790036	0.7349064
Add2	0.2376658	7.5146669	0.2791292	0.7349949
1110051M20Rik	0.2780863	2.479595	0.2792099	0.7349949
Fgfr2	0.2426233	5.6275477	0.2792163	0.7349949
Tor1a	-0.1518786	5.0845115	0.2792262	0.7349949
Plpp3	-0.1304673	5.9072336	0.2793035	0.735074
Smdt1	0.1367109	6.4507078	0.2795822	0.7356222
Abcb1a	-0.4577201	0.8742955	0.2796065	0.7356222
Nosip	0.1310987	5.3749518	0.2796617	0.7356432
Ipo13	0.1509054	4.561649	0.2798211	0.7359275
Atp5g2	0.1172755	6.2246239	0.2798644	0.7359275
Tbpl1	0.1227995	5.3875882	0.2800612	0.7362859
1700096K18Rik	-0.3088649	1.8344543	0.2803354	0.7362859
Eif2a	0.1396998	6.0591099	0.2803607	0.7362859
Map2k1	-0.1518609	5.786232	0.2803956	0.7362859
Prex2	-0.3254467	2.6825765	0.2804525	0.7362859
Cdca3	0.1444215	6.3434103	0.2804578	0.7362859
Galnt12	0.2777521	2.1164292	0.2804601	0.7362859
Ly6g6c	0.5424597	0.2607279	0.2805462	0.7362859
Apol8	0.4294582	5.3031574	0.280548	0.7362859
Fignl1	0.1846677	6.9873357	0.2806885	0.7362859
Lage3	0.1610697	4.4472109	0.2806885	0.7362859
Bub3	0.1255637	7.0582696	0.2806978	0.7362859
Grb2	-0.1367044	7.3641034	0.2807129	0.7362859
Eef1e1	0.2360327	4.9767824	0.2807183	0.7362859
Gm31692	-0.5525973	-0.5284616	0.280788	0.7362859
Gm52889	0.2785338	1.938165	0.2808195	0.7362859

Slco4a1	0.2367776	3.4829579	0.2808273	0.7362859
Gmfb	-0.1156716	6.9441099	0.2808877	0.7362859
Ganc	-0.2182452	4.112181	0.2809012	0.7362859
Edar	0.4878863	0.4582944	0.2809863	0.7362859
Hoxb3	-0.3451508	1.4979784	0.2810475	0.7362859
Pten	-0.1530629	7.4681728	0.2811241	0.7362859
Gm13073	0.6414598	-0.897495	0.2811315	0.7362859
Mus81	0.1703233	3.6823113	0.2811798	0.7362859
Ints5	0.1409005	5.088232	0.2811842	0.7362859
Zfp579	0.3014477	2.7622388	0.2812379	0.7363027
1810044D09Rik	0.4448136	-0.2521145	0.2813558	0.7363965
Snrpg	0.1800976	6.2455489	0.2813684	0.7363965
Pabpc4	0.1760115	8.3137013	0.2814966	0.7366081
Hadhb-ps	0.4302723	0.6768829	0.2817785	0.7372217
Tspan10	-0.6046392	0.689431	0.2820436	0.7374326
Rnf17	0.731376	0.233416	0.2820636	0.7374326
Dhx16	0.1173738	6.0052008	0.2820754	0.7374326
Prpf40a	-0.1326545	7.5726118	0.2821574	0.7374326
Chmp4b	-0.1167589	7.35265	0.2822351	0.7374326
Rnf166	-0.1661573	5.2858895	0.2822417	0.7374326
Lsr	0.4745056	0.5865617	0.282247	0.7374326
Tpgs1	0.1667001	4.1502536	0.2822571	0.7374326
Fam174b	-0.3184108	2.4995343	0.2822858	0.7374326
Btg1	0.2029115	7.963788	0.2823897	0.7375801
Riok2	0.1746475	4.5433477	0.2825623	0.737845
Rapgef1	0.1220723	6.548658	0.282586	0.737845
Upf1	0.1270607	6.9053199	0.2827266	0.7380883
4921524J17Rik	0.171235	4.0311781	0.2828416	0.7381802
Actr5	0.1937354	3.5757611	0.2829271	0.7381802
Gramd1c	-0.2480461	3.5905344	0.2830497	0.7381802
Flt3l	0.2272312	3.4729648	0.2830866	0.7381802
Ezr	0.1277231	7.7324447	0.2830895	0.7381802
Gm38604	0.5137215	-0.6490117	0.2831927	0.7381802
C130036L24Rik	0.3588504	0.6043596	0.2832282	0.7381802
Gm8894	-0.4497919	-0.2450961	0.283379	0.7381802
Get4	0.1408226	5.5690658	0.2833962	0.7381802
Gm39714	-0.3637417	2.3373681	0.2834235	0.7381802
Mrpl49	0.1251955	5.3289472	0.2834436	0.7381802
Atp5d	0.1165619	6.6591395	0.2834641	0.7381802
Fam107b	-0.1931588	8.3256856	0.2835443	0.7381802
Catspere2	-0.3648722	1.6716323	0.2835544	0.7381802
Cyc1	0.113204	6.3079736	0.2835672	0.7381802
Pex1	0.1792353	3.4032911	0.2836031	0.7381802

Hexim2	-0.2906704	2.3383872	0.2836084	0.7381802
Gm15326	0.6370705	-0.9819749	0.2836161	0.7381802
Tead2	0.4983743	2.1039454	0.2836715	0.7382007
Satb2	-0.3374875	6.2661363	0.2837342	0.7382217
Adam1a	0.3483378	1.0095137	0.2837744	0.7382217
Dynlt1f	-0.191985	3.7631597	0.2838825	0.7383793
Galnt9	-0.4504659	1.4228969	0.2840168	0.7386052
Itga11	-0.2620701	3.7983592	0.2841585	0.7388501
Slc35f2	-0.3907764	-0.0471844	0.284238	0.7389333
Mical1	-0.1285219	5.6728173	0.2843202	0.7390233
Mapkbp1	-0.2094476	3.3815114	0.2844536	0.7392466
Parp1	0.1695105	7.0229649	0.2846267	0.739573
Snupn	0.2520522	2.9213163	0.2848043	0.7396376
Mrpl19	-0.1452686	4.5594716	0.2848444	0.7396376
Chst5	-0.4678408	0.194292	0.2848559	0.7396376
Tmem198b	0.3167754	2.4592174	0.284876	0.7396376
Taldo1	-0.1384898	8.8498014	0.284895	0.7396376
Chdh	-0.2400245	4.7211852	0.2849369	0.7396376
Ngdn	0.1722544	5.0980227	0.2850469	0.7397765
Peg10	-0.3164163	2.5402132	0.2850855	0.7397765
Fbxl12	0.1991535	4.3212943	0.2852326	0.7399269
Rpl41	0.2081448	10.405208	0.2852386	0.7399269
E2f7	-0.1709347	4.6539205	0.2853582	0.7399955
Gm38485	0.401692	0.8935328	0.2853602	0.7399955
Timm17a	0.1881995	4.8449503	0.2855579	0.740287
Qars	0.1230323	6.350741	0.285634	0.740287
Hmox1	-0.2655858	7.2130188	0.285654	0.740287
Gm32618	0.5471968	0.1717743	0.2857359	0.740287
Ehbp1	0.3022281	2.1817219	0.2857775	0.740287
Bola3	0.1955315	5.9703499	0.2857928	0.740287
Phykpl	0.2232286	3.5309919	0.2858646	0.740287
Atp2b4	0.1841503	7.4161234	0.2859101	0.740287
Lin37	0.1737413	4.0569165	0.2859167	0.740287
Prdx3	0.2086119	6.9526526	0.2859486	0.740287
Rnf6	-0.1276014	5.8240331	0.2861543	0.7403406
Cd3g	-0.3226459	2.1221616	0.2861685	0.7403406
Ddx55	0.1887266	3.8580183	0.2861807	0.7403406
Art4	0.2266388	4.6649957	0.2861979	0.7403406
Dnaaf3	-0.495177	0.3560225	0.2862073	0.7403406
Prkacb	-0.1296633	6.4137535	0.2863527	0.7405936
Zfp697	-0.3324004	1.2345848	0.2865267	0.7408001
Gm14230	0.2678272	2.2198998	0.286545	0.7408001
Mtcl1	-0.5718541	0.4217191	0.286642	0.7408001

Gssos2	0.414535	1.1492959	0.2867481	0.7408001
H2-T-ps	-0.5229276	1.6873286	0.2868064	0.7408001
Wdr18	0.1683336	4.8303018	0.2868072	0.7408001
Ptp4a1	-0.1479008	5.0308082	0.2868769	0.7408001
Knstrn	0.1466251	6.5972621	0.2868792	0.7408001
Phospho1	-0.249218	7.1850499	0.2869038	0.7408001
Fars2	0.1713477	3.767848	0.2869978	0.7408001
Slc16a10	0.1863964	7.5459039	0.2870614	0.7408001
1700094D03Rik	0.3527799	2.2074552	0.2871143	0.7408001
Rbm43	0.1447969	5.7705487	0.2871998	0.7408001
Nsd3	-0.1339186	7.3079555	0.2872054	0.7408001
Ednra	-0.2430742	3.8053279	0.2872075	0.7408001
Cdc40	-0.1657485	6.0181243	0.2872957	0.7408001
Tmem97	0.1630441	4.2787089	0.2873629	0.7408001
Iqgap3	-0.2035818	5.3539904	0.2874099	0.7408001
Ybey	0.2653354	2.1967779	0.2874549	0.7408001
Mansc1	-0.4158755	1.6871118	0.2874825	0.7408001
Inip	0.1294302	5.1373738	0.2874863	0.7408001
Actr2	-0.1362472	8.9356172	0.287522	0.7408001
Il12a	0.2512286	2.9843647	0.287623	0.7408001
Sel1l	-0.1183138	6.6222838	0.2876533	0.7408001
Mapk11	0.37783	1.8265604	0.2876629	0.7408001
Cpne5	0.5793389	-0.29469	0.2876744	0.7408001
Tmc8	0.1501465	5.3915257	0.2877687	0.7408001
Afap1l1	-0.2133048	2.8099239	0.2878079	0.7408001
Tmem185b	-0.17199	4.0939577	0.2878138	0.7408001
Vopp1	0.1915292	6.8157616	0.2881588	0.7414463
Fchsd1	-0.2549825	2.913591	0.2881622	0.7414463
Copz1	0.1210263	7.1214379	0.2882078	0.7414463
A830049F12Rik	-0.3628074	0.5188587	0.2884081	0.7417289
Plxna1	-0.1922802	4.9570007	0.2884583	0.7417289
Gm31872	0.4046686	0.9343353	0.2884607	0.7417289
Aimp1	0.1489623	5.7951766	0.288596	0.741899
Etv3	-0.1531685	5.0139545	0.2886223	0.741899
Chd6	-0.1619637	5.773312	0.2887127	0.7420089
Rps14	0.2191564	9.1125793	0.288931	0.7424471
Gm38396	0.2528644	2.7281567	0.289053	0.7425751
4930524J08Rik	0.4364176	0.0736783	0.2890763	0.7425751
Rbfox1	0.761921	-0.6683678	0.2891683	0.7426889
Klhdc3	0.1289976	5.9932883	0.2892536	0.7427854
Ushbp1	0.2142159	3.4052432	0.2894923	0.7429283
Ybx1	0.1245063	9.3568038	0.2895115	0.7429283
Fam50a	0.1291307	5.4956395	0.2895339	0.7429283

Slc17a5	-0.1699699	3.7783269	0.2896066	0.7429283
Pusl1	0.2384984	2.7790519	0.2896121	0.7429283
Thoc6	0.1401222	4.2409704	0.2896283	0.7429283
Tubgcp6	0.1586028	5.0365399	0.2896742	0.7429283
Ctsz	0.1304167	7.4245382	0.2896914	0.7429283
D430020J02Rik	-0.197907	3.3424616	0.2897635	0.7429907
Ift22	0.2723984	3.0225262	0.2898888	0.7431896
Rrs1	0.1528481	4.928812	0.2902219	0.7435547
Nsun7	0.635425	0.0737389	0.2902283	0.7435547
Nptn	-0.1134464	7.7844938	0.2902344	0.7435547
Slc38a7	0.1957759	3.1877899	0.2902769	0.7435547
Sertad2	0.1784179	6.1782048	0.2902881	0.7435547
Pccb	0.1313485	5.0716031	0.2903295	0.7435547
Sod2	0.1203058	5.9754764	0.2903659	0.7435547
Atp2a3	-0.1956622	8.3383115	0.2904551	0.7435762
Kmt2e	-0.1547457	7.4122452	0.2904844	0.7435762
Mex3c	-0.1166585	5.8378504	0.2905605	0.7435762
Gm39269	-0.579201	-0.5090778	0.2905655	0.7435762
Xpo7	0.220711	9.1546373	0.2907332	0.7438432
Slc36a4	-0.1923675	4.7411514	0.2907768	0.7438432
Tpgs2	0.1747206	3.5611661	0.2908133	0.7438432
Zmynd8	0.1140674	6.6801297	0.2911472	0.7445746
Elf4	-0.2025656	6.3601887	0.2912107	0.7446146
Ift74	-0.2204736	2.6109038	0.2913181	0.7447392
Tmem9	0.1949877	5.0588634	0.2913827	0.7447392
Nt5e	0.3699848	4.6285678	0.2914249	0.7447392
Nckap5l	0.2640051	2.8535517	0.291497	0.7447392
Hif1a	-0.1288892	6.4859386	0.2914988	0.7447392
Hoxc5	0.6220477	-0.767285	0.291581	0.7448268
Swi5	0.1451437	6.682312	0.2917178	0.7448417
Cd8b1	0.4866581	0.5457837	0.2918097	0.7448417
Cd320	0.2611473	2.0498503	0.291843	0.7448417
Gm31665	-0.6015781	-0.7474655	0.2918628	0.7448417
Hdac3	0.124073	5.3710984	0.2918847	0.7448417
Mterf3	0.144969	4.4276756	0.2919624	0.7448417
Tfec	-0.1786787	4.7110666	0.2919658	0.7448417
Kng2	0.211012	4.047991	0.2919949	0.7448417
Llph-ps2	0.3735098	0.173611	0.2920178	0.7448417
Syt15	0.5815297	-0.0991854	0.2921628	0.7449891
Nfyc	0.1238356	5.3955092	0.2921714	0.7449891
Frat2	-0.1725274	4.5940988	0.2922811	0.7450168
Pam16	0.2252199	3.3502229	0.2923202	0.7450168
Tmem240	0.5055601	-0.3288874	0.2923259	0.7450168

Atrn	-0.1469705	6.4913422	0.2923748	0.7450192
Arl8a	-0.1518278	5.3324645	0.2924495	0.7450876
Reep6	0.3178487	3.6937888	0.2925964	0.7451376
Rgs2	-0.1324168	6.9538536	0.2925968	0.7451376
Crnde	0.4097569	1.5032406	0.2926419	0.7451376
Dpyd	-0.4905051	0.6867265	0.2926693	0.7451376
Smarcad1	-0.1230029	5.2881294	0.2927448	0.7451376
Rpl38	0.212644	8.669264	0.2927566	0.7451376
Nrg1	-0.3089125	2.9860413	0.2929652	0.7455466
Gm6477	0.2671634	2.1863526	0.2932391	0.7458113
Mideas	-0.1249673	6.1553795	0.2932554	0.7458113
Dnajb9	-0.1480715	5.3749757	0.2932843	0.7458113
Rhbdf1	0.3073018	2.6808241	0.2933082	0.7458113
Gm7072	0.1690395	4.6212821	0.293309	0.7458113
Apobec1	0.1582854	5.6890669	0.2935744	0.7463643
Kctd13	0.2292572	2.9735164	0.2938651	0.7469539
Pglyrp2	-0.4005105	0.4492208	0.2939024	0.7469539
Gm40245	-0.3525652	0.0905351	0.293964	0.7469882
Cabin1	0.1392736	6.5245553	0.2940366	0.7469882
Stk4	-0.1738332	7.8729189	0.2941024	0.7469882
Dnhd1	0.3031165	1.5309814	0.294108	0.7469882
Rsad1	0.421442	1.4106649	0.2941728	0.7470307
Exoc3l4	-0.1881487	3.4978664	0.2942235	0.7470376
Rnft2	0.7541565	-0.546752	0.294365	0.7471311
Plekha1	0.1810177	4.6950359	0.2944155	0.7471311
Zfp469	-0.4028208	3.5887408	0.2944351	0.7471311
Sh3bgrl	-0.1299099	7.9243047	0.2944525	0.7471311
Zfp354b	0.5218759	0.3621504	0.2945418	0.7471333
Atp6v1c2	-0.873253	-1.355035	0.2945494	0.7471333
Ppp2r5a	-0.1565651	7.9314193	0.2946595	0.7471608
Maml3	-0.2040698	3.434654	0.2946816	0.7471608
Mier1	-0.1291129	7.0738475	0.2947044	0.7471608
Abracl	-0.1611396	6.7915725	0.2949178	0.7474298
Pcdhga5	-0.7349532	-0.1720674	0.2949509	0.7474298
Glt8d1	0.1934886	4.4168429	0.2949546	0.7474298
Fam20a	0.2929423	2.3571967	0.2950407	0.7475262
Zfp940	0.3708795	1.2881069	0.2951376	0.7476498
Bahd1	0.1357596	5.1027217	0.295432	0.7482739
Zfp830	0.1414957	4.9431001	0.2954964	0.748315
Gm21814	-0.5769436	-0.2252066	0.2955879	0.748425
Polr3c	0.1322154	5.1729186	0.2957041	0.7485971
Uimc1	0.1171033	5.953508	0.2958313	0.7487195
Kbtbd12	0.4337952	0.9742754	0.2958487	0.7487195

Setx	-0.1672507	6.652545	0.2959778	0.7488403
Dffa	0.1756012	4.1661294	0.2960077	0.7488403
Castor2	-0.4377664	2.1913395	0.2960408	0.7488403
Hmga1	0.18524	6.2112154	0.2961578	0.7490052
Dnajb1	-0.1272995	6.7021252	0.2962217	0.7490052
Abca5	-0.3417581	1.742931	0.2962605	0.7490052
Prss57	-0.2318924	4.7330823	0.2963719	0.7490052
Col8a2	-0.9051306	0.1264651	0.2963874	0.7490052
Fbxw11	-0.1274164	6.1325065	0.2963949	0.7490052
Utp11	0.1252997	5.0873241	0.2964611	0.7490099
Prmt1	0.1227035	5.9416649	0.2964931	0.7490099
Sparc	0.3747938	12.092263	0.2966139	0.7491933
Plekhs1	-0.6415412	-1.0101027	0.2968261	0.7496025
Apc	-0.1202894	6.1850319	0.2968723	0.7496025
Jak2	-0.1364253	7.3369956	0.2969852	0.749766
Cep78	0.2009417	4.0089682	0.2974582	0.7508381
Fer1l6	0.3914865	0.7191537	0.2975888	0.7510267
Dph3	0.1862905	4.9864817	0.2976295	0.7510267
Zscan21	0.213112	4.0017847	0.2977687	0.7511582
Rbm26	0.1189777	6.2531673	0.2977782	0.7511582
LOC118568759	0.3895517	0.2804158	0.297828	0.7511621
Rad9b	0.3137027	1.3686767	0.2979175	0.7512661
Fam214b	0.1761342	6.415597	0.2980088	0.7513745
Ttc7b	-0.1846371	4.8877784	0.2980647	0.7513936
Yme1l1	-0.1190487	7.2130177	0.2981241	0.7514216
Snx7	-0.3375018	3.4774388	0.2983123	0.7516175
Nono	0.1127271	8.5036794	0.2983527	0.7516175
Rbpj	-0.1293994	5.997072	0.2983656	0.7516175
Lonp1	0.1291946	5.8696681	0.2984666	0.7516175
LOC108167377	0.4575573	1.0184579	0.2984726	0.7516175
Rfxap	0.1446356	4.4494198	0.298522	0.7516175
Vps72	0.1258935	4.9954164	0.2985401	0.7516175
Pkmyt1	0.165445	4.9299981	0.2985884	0.7516175
Mbp	-0.1306456	6.1480889	0.2986534	0.7516594
Rictor	-0.1129357	5.9204174	0.2987894	0.7517199
Gm7390	-0.3379322	0.743377	0.2988318	0.7517199
Ppp2r1a	0.1215543	6.8035821	0.2989599	0.7517199
Cldn1	-0.1909605	4.5284831	0.2989824	0.7517199
Gm7536	0.1668229	5.1448426	0.2989897	0.7517199
Sp2	0.1542802	5.6499959	0.2989924	0.7517199
Mcm7	0.1505649	8.3918015	0.2990158	0.7517199
Hivep2	0.2520579	4.4382915	0.2991743	0.7519295
Iigp1	-0.1815548	3.8870894	0.2991958	0.7519295

Gsdmd	-0.1287517	5.481668	0.2992836	0.7520286
Tmem64	0.1173993	5.7067483	0.2997372	0.7527669
Gzf1	-0.1247813	4.9539223	0.2997612	0.7527669
Ruvbl2	0.1299059	5.5925883	0.2997766	0.7527669
Dnajc28	-0.2192922	2.4504209	0.2998148	0.7527669
Nfkbia	0.2407707	6.1951841	0.2998194	0.7527669
Tgm2	-0.1365543	6.3279108	0.2999623	0.7528296
D10Wsu102e	0.1248153	5.0918567	0.2999804	0.7528296
Atp6v0c-ps2	-0.282585	4.6295932	0.2999896	0.7528296
Acod1	-0.4808871	1.2453927	0.3001406	0.7529839
Mrps31	0.1953229	3.7003572	0.3001847	0.7529839
Tchh	-0.5169815	-0.1331009	0.3001963	0.7529839
Tmem205	-0.239432	3.1998411	0.3004506	0.7534288
ND5	-0.1471977	11.028188	0.3005076	0.7534288
Smad4	-0.1365484	5.9000697	0.300519	0.7534288
Pgrmc2	0.1196806	6.0680127	0.3006551	0.7534579
Il1rl1	0.1609223	4.8646729	0.3007467	0.7534579
Clcn7	0.1360547	5.0514945	0.3007718	0.7534579
Eif4e3	-0.1921445	5.0664943	0.3007868	0.7534579
Rraga	0.1180385	5.5851698	0.3008024	0.7534579
Tigar	0.2363011	2.6276127	0.3008355	0.7534579
Gm41949	-0.3081086	1.2898947	0.3008697	0.7534579
Myl12b	-0.1428664	8.256736	0.3010078	0.7536824
Oasl1	-0.319241	1.8430279	0.3010708	0.7537189
Gm46287	0.5383378	-0.1051668	0.3011795	0.7538209
Gm32220	0.6800339	-0.687667	0.3012085	0.7538209
Pkp4	-0.1919374	4.5116786	0.3012842	0.7538891
Mrps16	0.1632318	4.8144694	0.3016241	0.7546077
Zkscan5	0.1878828	3.4599671	0.3017166	0.7546077
Gm5547	0.5885052	-0.316236	0.3017169	0.7546077
Gvin2	0.3466169	2.1078961	0.3020944	0.7554304
Spon1	-0.2216278	4.6706972	0.302144	0.755433
Ets1	0.1558993	7.7270774	0.3023748	0.7558885
Usp14	0.1576206	6.5999947	0.3025112	0.7561078
Jade1	-0.1245601	5.4993822	0.302797	0.7563117
Kcnq1	-0.5429741	-0.1746929	0.3028124	0.7563117
Trim21	-0.1674927	4.0693901	0.3028453	0.7563117
Tmcc2	0.2527056	8.9321784	0.3028857	0.7563117
Wdr5b	0.1885657	3.1235002	0.3029015	0.7563117
Mtfr1	0.208199	6.669115	0.3029523	0.7563117
Zc3h6	0.2673751	2.3473119	0.3029626	0.7563117
Med12	0.1397423	6.7307518	0.3029818	0.7563117
Taf6l	0.2298064	3.5068927	0.30305	0.7563277

Mia2	-0.1291631	6.8156486	0.3031358	0.7563277
Nxpe2	0.2414225	7.8811956	0.3031689	0.7563277
Nxpe5	0.5093515	-0.0095076	0.3032046	0.7563277
4930555A03Rik	0.6107998	-0.2683809	0.3032313	0.7563277
4831440E17Rik	0.2675523	1.3881338	0.3033298	0.756452
Edrf1	0.1284678	5.1740255	0.303387	0.7564733
Fam167a	0.3540026	2.7860538	0.3034916	0.7566129
Dhrs4	0.1860577	4.9840141	0.3035402	0.7566129
Gpd1l	0.1830451	6.1943449	0.3037583	0.7570352
Spock2	-0.4561693	0.6262619	0.3038937	0.7570696
Gm31090	-0.7175789	-1.0769107	0.3039016	0.7570696
Xpa	0.1935112	3.6007505	0.3039181	0.7570696
Plpp1	0.1939997	5.1946817	0.3042396	0.757623
Sparcl1	-0.3770021	2.7239082	0.3042734	0.757623
Snai1	-0.3651007	1.6119955	0.3042864	0.757623
Zfp346	0.1480597	4.2286028	0.3044028	0.7577253
Fam210b	0.2034328	6.5252689	0.3044542	0.7577253
Wipf2	-0.1286213	5.4810335	0.3044879	0.7577253
Terf1	0.1538125	5.3017393	0.3045328	0.7577253
Gm17249	-0.6042728	-0.9337229	0.3045711	0.7577253
Myh11	-0.318108	1.8788098	0.3048425	0.758055
Gm4532	0.5955728	-0.0304989	0.3048602	0.758055
Cr2	0.4528813	3.5666321	0.3048969	0.758055
Abca9	-0.172844	4.5338004	0.3050316	0.758055
Paip2b	-0.1751623	3.7913149	0.3050629	0.758055
Porcn	-0.3701802	1.4962042	0.3050821	0.758055
Tnrc18	-0.1668906	6.7350266	0.30509	0.758055
Clu	-0.1502784	6.6630849	0.3050935	0.758055
Ppdpf	0.1752193	4.0344655	0.3052046	0.75821
Efr3b	-0.4699596	0.3342509	0.3053064	0.7583151
Cacna1h	0.3768816	0.754524	0.3053636	0.7583151
Slc25a12	-0.1357073	5.6014166	0.3054011	0.7583151
Rpl4	0.137424	10.777971	0.305442	0.7583151
Pdgfa	-0.2882588	3.7409092	0.3054993	0.7583364
Ccn3	-0.8924516	-0.9979788	0.3056228	0.7583454
Fbxo32	-0.2713837	3.1042325	0.3056488	0.7583454
Tspan8	0.2532898	5.6462924	0.3056492	0.7583454
Bhlhe41	0.3524879	2.3257744	0.3057354	0.7584384
Myom3	-0.5436559	-0.5557758	0.3059193	0.7586745
Qtrt1	0.2520734	2.7900863	0.3059507	0.7586745
Mrpl24	0.1469279	5.4223514	0.3059769	0.7586745
Sema4a	-0.1529979	6.7124773	0.3064947	0.7597557
BC025920	0.3914667	1.142584	0.3065107	0.7597557

Zranb2	0.1264771	6.9755564	0.3066573	0.7597936
Retreg2	0.1239334	6.5393422	0.3066594	0.7597936
Slc38a9	0.1551992	5.9818862	0.3066725	0.7597936
Polr2i	0.169326	4.1571793	0.3067895	0.7599387
Zdhhc17	-0.1358856	5.0405483	0.3068332	0.7599387
Aqp1	0.1806736	9.4007065	0.3068777	0.7599387
Eif4ebp2	-0.1936601	6.1177645	0.3069475	0.7599842
Glcci1	0.2574793	5.8345945	0.3069937	0.7599842
Cisd3	0.4173463	1.8418267	0.3070932	0.7601093
Aasdh	0.2216408	3.248412	0.3073424	0.7601966
Foxn3	-0.1425697	6.4867789	0.3073628	0.7601966
Mecp2	-0.1153222	5.9252748	0.3073904	0.7601966
Kif5c	0.6500871	-0.8107426	0.3073985	0.7601966
Gadd45a	0.2012134	6.5262811	0.3074094	0.7601966
Mospd1	-0.1480587	5.3355799	0.3074612	0.7601966
Rps6	0.1313575	9.6573312	0.3074721	0.7601966
Ren1	-1.0233332	-0.1956503	0.3075194	0.7601966
Plxnd1	0.1328218	6.6067271	0.3076096	0.7602988
Ddhd2	0.1310726	4.8993282	0.3076987	0.7603584
A430105J06Rik	-0.5178829	0.2044852	0.307887	0.7603584
Dnlz	-0.1384662	4.2676819	0.3079104	0.7603584
Cyp4f18	0.1638883	5.9561943	0.3079128	0.7603584
Slc30a1	-0.1348499	5.5276593	0.3079226	0.7603584
Pggt1b	-0.133139	5.709436	0.307928	0.7603584
Gm11585	-0.432324	1.7254106	0.3080155	0.7603584
Stab2	-0.2270036	5.6459152	0.3080736	0.7603584
Ccne2	-0.1485559	7.2125968	0.3080737	0.7603584
Msr1	-0.2131114	3.8703961	0.3082369	0.7606025
Baiap2l2	-0.4731028	0.2760319	0.30831	0.7606025
Slc44a2	0.1182614	6.7357295	0.3083193	0.7606025
Mfsd8	0.1864943	3.1948786	0.3084006	0.7606825
Gm12258	0.3294425	3.3587908	0.3084617	0.7607125
Ndufb6	0.1498868	5.2567489	0.3086744	0.7610236
Eif4b	0.1149269	8.5855148	0.3086857	0.7610236
Lgr4	-0.3018243	3.7835788	0.3087413	0.7610399
Ermard	0.1947851	3.3886557	0.308892	0.7612908
Pknox2	-0.3315648	1.0922695	0.3090139	0.7613929
Pdxp	0.2848432	2.2289737	0.3091038	0.7613929
Rela	0.1313602	5.9236012	0.3091103	0.7613929
Oplah	0.3227375	2.0119062	0.3092204	0.7613929
Rpl12	0.1856772	9.7513451	0.3092261	0.7613929
Gm9522	1.4697228	-0.0389799	0.3092271	0.7613929
Uba3	-0.1197395	5.6297258	0.3092775	0.7613964

Cbll1	0.1481385	4.744613	0.3093444	0.7614405
Rnf14	0.1212422	6.3667203	0.3094763	0.7616447
Akap8l	0.1309166	5.4977928	0.3096063	0.7616622
Twnk	0.1649723	4.0199451	0.3097463	0.7616622
Hibadh	0.126173	5.4793165	0.3097623	0.7616622
1700048O20Rik	0.3440602	1.4607124	0.309864	0.7616622
Rnf165	-0.4836521	-0.384482	0.3098761	0.7616622
Krit1	-0.1101683	5.7256647	0.3099045	0.7616622
2410006H16Rik	0.1878787	4.7631559	0.3100392	0.7616622
Hapln4	0.5716242	1.0550849	0.3101107	0.7616622
Gpx4-ps2	0.272432	3.0190111	0.3101345	0.7616622
Psmb6	0.1182232	6.5026726	0.310188	0.7616622
Spidr	-0.1420407	4.908256	0.3102359	0.7616622
Slc27a3	-0.35693	1.782304	0.3102493	0.7616622
Ipo9	0.1296658	6.2649924	0.3102767	0.7616622
Slc25a16	-0.1542482	4.6094634	0.3103193	0.7616622
Fbp1	-0.3768102	1.5756662	0.3103946	0.7616622
Tmc7	-0.3783198	1.2045303	0.31046	0.7616622
Troap	0.1863725	4.496935	0.3105664	0.7616622
Prmt6	0.1579664	3.8783261	0.3105845	0.7616622
Rgs7bp	0.4511554	0.9512144	0.3106231	0.7616622
Ccdc90b	-0.1755416	3.9547839	0.31064	0.7616622
Ctdp1	0.1462111	5.0008404	0.310674	0.7616622
Syt13	-0.6204567	-0.6524946	0.3107037	0.7616622
Bloc1s2-ps	0.6524449	-0.6245507	0.3108892	0.7616622
Mast3	-0.1408344	5.9120037	0.3109041	0.7616622
Uhmk1	-0.115578	7.4235751	0.3110156	0.7616622
Nudc	0.1306959	6.7909186	0.3111837	0.7616622
Cfap57	-0.4254592	-0.0819942	0.3112075	0.7616622
Usp47	-0.1224046	6.9344863	0.3112304	0.7616622
Amigo2	0.3051572	1.7617063	0.311262	0.7616622
Trim9	-0.6976536	-0.7302201	0.3112712	0.7616622
Gm35339	0.293996	1.6544269	0.311288	0.7616622
Gfi1b	0.1677116	5.8990285	0.3113383	0.7616622
LOC118567527	0.2491866	3.4105202	0.3113814	0.7616622
Cyp2j6	0.3460577	1.7465496	0.3114794	0.7616622
Col25a1	-0.3884965	2.354992	0.3114969	0.7616622
Sulf2	-0.177628	4.4362045	0.3114992	0.7616622
Ndufa5	0.2123469	5.7582428	0.3115292	0.7616622
Sirt6	0.1951873	3.329404	0.3115656	0.7616622
Nlrp10	-0.4177205	0.0864065	0.3115972	0.7616622
Pdcd5-ps	0.3017875	0.8320009	0.3116285	0.7616622
Mfsd1	-0.1259102	6.356514	0.3116502	0.7616622

Ewsr1	0.1338914	8.5235131	0.3116554	0.7616622
Nudt15	0.2354892	1.9201303	0.3116708	0.7616622
Gm42365	-0.4582532	0.0854388	0.3117406	0.7616622
Ubqln2	0.1238363	5.0519198	0.3118821	0.7616622
Dock2	-0.1991652	7.2723388	0.3119097	0.7616622
Leprotl1	-0.1446313	5.444564	0.3119332	0.7616622
Slc5a6	0.309437	1.7225874	0.3119716	0.7616622
Gm5555	0.6406192	-0.6909344	0.3120404	0.7616622
Mdm1	-0.1830843	4.7207268	0.3120783	0.7616622
Rasl11b	0.8065789	1.9826211	0.3121209	0.7616622
Zfp503	0.2960538	2.175421	0.3121339	0.7616622
Rab10os	0.2435235	2.50012	0.3121448	0.7616622
Rps25-ps1	0.2929182	2.3040947	0.3121466	0.7616622
Col20a1	-0.3637829	1.6474267	0.3121802	0.7616622
Gm13665	0.7978122	-0.7462597	0.3122257	0.7616622
Gdi2	0.1183203	8.4111329	0.3122802	0.7616757
Lrrc20	0.1829086	4.3379751	0.3123915	0.7618215
Arhgap6	-0.186876	4.6749033	0.3124379	0.7618215
Sdk2	-0.2727608	4.0634599	0.3125694	0.7620227
Ehmt2	0.1343022	6.2653599	0.312873	0.7625984
Treml2	-0.1374883	6.7069211	0.3129463	0.7625984
Dnaja3	0.1152506	5.2483153	0.312968	0.7625984
Tie1	0.1798686	4.478025	0.3130037	0.7625984
Atp6v1b2	-0.1162608	7.7442962	0.3130507	0.7625984
Neu1	-0.1711954	5.5846065	0.3131356	0.7626856
Map7d1	-0.1317689	6.7736666	0.3133335	0.7628704
Gm42149	0.4439999	0.1839824	0.3133722	0.7628704
Amd-ps4	0.3725959	0.6545996	0.3134013	0.7628704
Chrm3	-0.3526499	1.1811529	0.3134076	0.7628704
Cc2d2a	-0.2446411	2.416932	0.3134723	0.7629086
Gpr174	-0.3694547	2.4491856	0.3136623	0.7631328
Ntn3	0.4123381	0.4753256	0.3136626	0.7631328
Gm35545	0.4983586	0.7374601	0.3138417	0.7634492
Vcp-rs	-0.2183628	2.676031	0.3141063	0.7638931
Gm40346	-0.5669065	0.1945	0.3141224	0.7638931
Adgrl2	-0.1741172	4.200226	0.3142228	0.7640178
Ift140	0.4869384	5.638698	0.3143369	0.7640816
Idnk	0.1486485	3.9883021	0.3143715	0.7640816
Tfeb	0.1520398	4.5381098	0.3143964	0.7640816
Gm2962	0.5283134	-0.1497352	0.3145343	0.7642973
Entpd6	0.2003418	3.3625635	0.3146547	0.7644703
Ccnf	0.1400911	6.8467354	0.3147774	0.7646266
Uso1	-0.105164	6.6398512	0.3148173	0.7646266

Cyfip1	-0.1224275	6.3451105	0.3150335	0.764644
Slain1	0.2760338	3.3282418	0.315101	0.764644
Galr2	0.6496651	-0.9436948	0.315135	0.764644
Mlst8	-0.1203711	5.3226783	0.315136	0.764644
A730063M14Rik	0.3615731	1.010524	0.3151703	0.764644
H2-Ke6	0.1573001	4.4437841	0.31518	0.764644
Bmp3	-0.3306757	5.4827122	0.3152035	0.764644
Pcdhga8	-0.8060987	-1.3869978	0.3152178	0.764644
BC055402	-0.4627672	2.6346466	0.315325	0.7647849
Afmid	0.2065113	5.0275141	0.3153763	0.76479
Pip5k1a	0.1296249	5.1780815	0.3155022	0.7648994
Gm6750	-0.3287902	0.9750999	0.3155583	0.7648994
Smoc1	0.2410764	3.3404892	0.3156036	0.7648994
Syt11	0.2333582	2.2038245	0.3156181	0.7648994
Zfp932	0.1870144	4.2551383	0.3156916	0.7649324
Zdhhc21	-0.1339662	5.4381334	0.3157409	0.7649324
LOC118567481	-1.0846782	1.0190793	0.3157793	0.7649324
Nktr	-0.116049	7.1993402	0.3159093	0.7651282
9230114K14Rik	-0.2559041	1.9936525	0.3160938	0.7654558
Ankrd2	0.5776694	0.906361	0.3162015	0.7655609
Frrs1	-0.1344684	5.6826386	0.3162356	0.7655609
Slco2b1	-0.224583	4.2685526	0.3163833	0.7656852
Gm12693	-0.6237801	-0.7302965	0.3163854	0.7656852
Arl15	-0.1627424	4.2786434	0.3165188	0.7658887
Gm15927	-0.3896894	0.3303607	0.3166688	0.7660705
Krr1	0.1329	5.6159575	0.3166924	0.7660705
Mpst	0.131751	5.4676884	0.31676	0.766115
Tbc1d10a	0.1824311	3.2328621	0.3168741	0.766137
Sec23ip	0.1047766	6.2957289	0.3169461	0.766137
Dnajc15	0.2221241	3.5945868	0.317041	0.766137
Ptger1	-0.3115621	1.2223282	0.3170495	0.766137
Coq8b	0.2110614	3.2781842	0.3170881	0.766137
Oas1a	-0.2624335	2.8793191	0.3171717	0.766137
Gm7027	0.3928927	-0.6005495	0.3171826	0.766137
Rtkn2	-0.2077875	3.3238941	0.3172528	0.766137
Aig1	0.2163423	2.9578522	0.3172606	0.766137
Ing2	0.1824671	4.2884141	0.3173212	0.766137
Kif13a	-0.1520852	5.1987003	0.3173538	0.766137
Gm13710	-0.2685477	1.023866	0.3173602	0.766137
Vezt	0.206986	4.3902808	0.3174196	0.7661614
Gm9774	0.4100382	0.2811629	0.3174983	0.7662325
Tmem167b	-0.1087768	5.8505475	0.3178213	0.766893
2610307P16Rik	0.4419217	0.3099737	0.3180076	0.7672235

Ptbp3	-0.1239787	9.1993991	0.3181696	0.7672698
Txnl1	0.1353213	6.9797694	0.3181756	0.7672698
Satb1	-0.161005	5.6236849	0.3182217	0.7672698
Mien1	0.1651582	4.4778896	0.3183255	0.7672698
Gas5	0.2122444	7.3513846	0.31839	0.7672698
Arhgap45	-0.128756	8.2421648	0.3183902	0.7672698
Dagla	0.4673499	-0.0542072	0.3184284	0.7672698
Magoh	0.1726767	6.260333	0.318459	0.7672698
Pnpla7	-0.1304818	5.7722809	0.3185176	0.7672698
Vcp	0.1130747	8.4368957	0.3185457	0.7672698
Gm13136	-0.4801086	-0.9811051	0.3185772	0.7672698
Ndufb9	0.137342	6.924913	0.3186574	0.7672698
Lpxn	0.2222147	3.0130085	0.3186681	0.7672698
Ifnar2	-0.1295599	6.5462877	0.3187706	0.7673979
Bptf	-0.1917875	7.1580827	0.318878	0.7674774
Khsrp	0.1201733	7.3996506	0.3189124	0.7674774
Zfp664	-0.1222807	5.9111084	0.3189516	0.7674774
Lrig1	0.2860533	2.6360571	0.3191269	0.7677804
Dusp22	0.2008195	3.4676233	0.3191863	0.7677962
Flrt3	0.5872424	1.3372103	0.319267	0.7677962
Hs6st1	0.1864367	6.9782519	0.3192816	0.7677962
Pex6	0.1612571	4.4049803	0.3193571	0.7678009
Ube2q2	0.1233604	5.3843347	0.3193823	0.7678009
Lig3	0.1232463	4.9733603	0.3194754	0.767906
Etv5	0.2516881	3.2448976	0.3195938	0.7680719
Tpmt	-0.2472331	1.5199482	0.3196557	0.7681021
Cryab	-0.3841728	3.2149911	0.319732	0.7681069
Xbp1	-0.1252995	6.8705026	0.3197565	0.7681069
Impact	-0.1673049	4.7407393	0.3199374	0.7683351
Gmppa	0.139973	4.5895385	0.3200064	0.7683351
Gm32725	-0.4703007	0.055628	0.3200104	0.7683351
Atrnl1	-0.1819249	4.9517675	0.3200749	0.7683351
Fbxl2	-0.2652944	3.7214537	0.3200984	0.7683351
Col9a2	-0.6738717	4.1471875	0.3201723	0.7683938
Lrrc69	-0.4382896	-0.4107338	0.3203296	0.7685679
Serpind1	-0.7807667	0.292567	0.3203437	0.7685679
Tenm3	-0.4596631	0.3038195	0.3204219	0.7686356
Atrip-trex1	0.2979544	1.3445592	0.3205079	0.7686356
Ptbp1	0.1218879	8.2433114	0.3205201	0.7686356
4930404H24Rik	0.4604393	-0.4932655	0.3207099	0.7688963
Gab1	0.134718	4.9897723	0.3207334	0.7688963
Lrp3	0.3202209	1.7993866	0.3208495	0.7688963
Tmem220	0.3730031	0.685351	0.3208771	0.7688963

Iscu	0.1449045	6.3206411	0.3209261	0.7688963
Usp30	0.1854934	3.3150556	0.3209416	0.7688963
Rbl2	-0.125021	5.9432782	0.3209749	0.7688963
Snrpd2	0.1368794	6.7029318	0.3211502	0.7691978
Daam1	-0.1490806	6.9946039	0.321389	0.7696511
Itgb4	-0.372183	0.0058731	0.3215205	0.7698475
Fbln1	-0.3084178	2.2464595	0.3216538	0.7700482
Smox	0.2042604	7.0765203	0.3217608	0.7701858
Gm34403	-0.4359927	0.663378	0.3218344	0.7702435
Lamp1	-0.1171441	8.6396539	0.3220211	0.7705113
Gm5931	0.4259552	-0.6792232	0.3221851	0.7705113
Gm51556	-0.2739267	2.0698092	0.3221975	0.7705113
6030442K20Rik	-0.481916	-0.727991	0.3222165	0.7705113
Nags	0.8914411	-0.1932547	0.3222181	0.7705113
Donson	0.14369	5.2423076	0.3222906	0.7705113
Isoc2a	0.1788067	3.692025	0.3222931	0.7705113
C1qtnf6	0.3537136	5.5700047	0.3224313	0.7706563
Suz12	0.1307416	7.7035813	0.3226082	0.7706563
Gpr34	0.2429501	2.3063165	0.3226406	0.7706563
Pappa	0.1876722	4.4198921	0.3226606	0.7706563
Lemd2	0.118095	5.0467789	0.3226959	0.7706563
Orai2	-0.2149453	5.5596125	0.3227391	0.7706563
Rufy2	0.2634776	3.2874624	0.322769	0.7706563
Hdac8	0.2322594	3.4212404	0.3227864	0.7706563
Mier2	0.2242276	2.5968418	0.3227997	0.7706563
Gm32178	-0.5497597	-0.6836179	0.323067	0.7711717
Natd1	0.1628264	5.4073121	0.3231212	0.7711717
Slc35f6	0.1497214	3.9965792	0.3232096	0.7711717
Gtf3c2	0.1024555	6.3553757	0.3232217	0.7711717
Crtam	0.4047884	0.2103861	0.323304	0.7711717
Gab3	-0.2280681	2.6199726	0.3233327	0.7711717
Rbm38	0.1512788	8.3144057	0.3234965	0.7711717
Tcim	0.2390088	2.9839844	0.3234994	0.7711717
Pip4k2b	-0.1509413	6.1630558	0.3235746	0.7711717
Map2k5	0.1554808	4.3085308	0.3235755	0.7711717
Suclg1	-0.1074865	5.7392126	0.3236746	0.7711717
Mlf2	0.1066844	6.330771	0.3237479	0.7711717
Pde4a	0.1966507	3.4992269	0.3238408	0.7711717
Gtf2a2	0.160752	6.2168454	0.3238467	0.7711717
Dpy30	0.147954	5.8575513	0.3238942	0.7711717
Gm5844	0.3693509	1.0217841	0.3239018	0.7711717
Gnas	0.1121095	9.4453963	0.3239322	0.7711717
R3hdm2	-0.1174875	6.1085658	0.323968	0.7711717

Zxdb	0.2239278	2.0211562	0.3239936	0.7711717
Gm9392	0.3437561	0.4585701	0.3240071	0.7711717
Fech	0.2228769	9.1917838	0.324118	0.7713176
Gm5596	-0.4581848	-0.2861156	0.3242318	0.7713996
Gm38293	0.4551251	-0.6500494	0.3242905	0.7713996
Klhdc8b	0.2401136	2.6008341	0.3243431	0.7713996
Slc9a7	0.1863178	4.4851891	0.3243509	0.7713996
Glud1	-0.1498765	7.1844683	0.3244856	0.7715946
Gtf2f1	0.1272296	6.631487	0.3245321	0.7715946
Igsf9	0.6145335	0.7211742	0.3248568	0.7720258
Tspan17	-0.2171478	3.1809012	0.3249407	0.7720258
Ndufaf8	0.2394435	3.8518042	0.3249798	0.7720258
Plpp2	0.3241151	1.5301717	0.3249876	0.7720258
Nsg2	0.4562593	-0.0874749	0.3249984	0.7720258
Evpl	-0.4311359	0.0940837	0.3250113	0.7720258
Aopep	0.158537	3.8543682	0.3251081	0.7720942
Tmem218	0.3186803	1.8448558	0.3251623	0.7720942
Golm2	0.2216805	3.7682776	0.3251889	0.7720942
Gm38882	0.3879092	0.6563537	0.3252769	0.7721241
Scrn2	0.2692477	1.6136883	0.3253009	0.7721241
Hmgn5	0.1193481	6.2297872	0.3254721	0.7724128
Rnh1	0.1142192	5.905412	0.3256721	0.7726671
Uchl3	0.1734831	4.7492696	0.3257093	0.7726671
Gm10080	-0.4342273	0.0303245	0.3257283	0.7726671
Shoc2	-0.1004235	6.3037764	0.3257923	0.7727011
Crim1	-0.1936108	4.0530655	0.3258817	0.7727953
Slc7a8	-0.2188942	4.8329025	0.326136	0.773243
Zc3h4	0.1218199	6.9605405	0.3261699	0.773243
Kbtbd6	0.1997267	3.4450433	0.326317	0.7734238
Crlf1	0.5635973	-0.7425349	0.3263896	0.7734238
Mbip	0.1908818	3.4728423	0.3263954	0.7734238
Nsl1	0.1227778	5.007145	0.3264464	0.7734268
Stk11	0.1248353	7.5787353	0.3265613	0.7735751
Gm19680	-0.687392	1.3764647	0.3266084	0.7735751
Zfp949	0.1965043	4.2935257	0.3268142	0.7738299
Sprtn	0.1195619	5.5128076	0.3268288	0.7738299
Tasor2	-0.1262919	5.3247517	0.3268716	0.7738299
Gm10232	0.2849004	1.291546	0.326915	0.7738299
Slc8a1	-0.1676193	4.1662802	0.3270562	0.7739701
Chkb	0.2681598	2.1480563	0.3270738	0.7739701
Rbm12b1	0.2965933	2.7878158	0.3271563	0.7740475
Nelfa	0.1280776	5.2343661	0.3273756	0.7743063
Ubr3	-0.1159205	6.4601346	0.3273809	0.7743063

Pcbd1	-0.4157342	0.2711366	0.327415	0.7743063
Fzd1	0.2519857	3.1003655	0.3275754	0.7744248
Agfg2	0.1456136	5.9186499	0.3277157	0.7744248
Gm52629	-0.4542213	-0.1297622	0.3277189	0.7744248
Gm35596	0.3286495	1.8890664	0.3277285	0.7744248
Egr3	-0.3019383	2.5436713	0.3278236	0.7744248
Ppp1r12b	-0.1321082	4.8233247	0.3278632	0.7744248
Acvrl1	-0.3427348	4.113049	0.3278678	0.7744248
Proser2	-0.1777996	3.537921	0.3279011	0.7744248
Ube2n	0.1164822	6.2435208	0.3279209	0.7744248
Phyh	-0.1564961	4.9667743	0.3280428	0.7744248
Large1	-0.278078	2.6673896	0.3280622	0.7744248
Pdgfra	-0.2139001	5.7937834	0.3280625	0.7744248
Sowaha	0.2433904	5.8629986	0.328138	0.7744854
Slc25a21	0.2637369	5.3938818	0.3281982	0.7745099
Gne	0.1287369	5.2269995	0.3282831	0.7745874
Rnaseh1	0.1582945	3.4080865	0.32852	0.7745874
Iftap	-0.1941424	3.6012776	0.3285353	0.7745874
Arhgef10	0.2337143	3.11118	0.3285382	0.7745874
Pcdhb16	-0.3673958	0.380748	0.3286092	0.7745874
Dynlt3	-0.1160481	6.1140528	0.3286142	0.7745874
Zfp90	0.2208537	3.2206125	0.3286461	0.7745874
Ltbr	0.1403793	5.1597762	0.3286483	0.7745874
Tmem62	0.242806	2.6197138	0.3286793	0.7745874
Rpl27a	0.1477469	9.751726	0.3287356	0.7746029
Mrpl58	0.1435546	4.4963799	0.3288202	0.774635
Npy	0.2315285	4.6631587	0.3288499	0.774635
Khdc3	-0.3442581	0.9844697	0.3289561	0.774635
Chadl	-0.4554695	0.9809015	0.328958	0.774635
Fam149a	0.3664077	2.2102253	0.3289983	0.774635
Uxt	0.1605868	3.9955477	0.3290782	0.774706
Cul4a	0.1624912	7.3957883	0.3291636	0.7747115
Psme1	0.1181533	6.6368008	0.3291802	0.7747115
Rab11fip1	-0.1735684	4.9104216	0.3292939	0.7748619
Lenep	0.3570183	0.5186053	0.329644	0.7755684
Orc2	0.1527535	6.2180988	0.3298335	0.775784
Acrbp	0.282526	2.5221011	0.3298641	0.775784
2510002D24Rik	0.1582442	3.4625102	0.3299652	0.775784
Maf	0.1592751	5.6392159	0.3299707	0.775784
Fam185a	0.2114716	2.6769145	0.3300074	0.775784
Pde1a	-0.3314081	3.1252952	0.3300869	0.775784
Rpl10-ps3	0.4287531	-0.1302119	0.3302641	0.775784
Zzef1	-0.1337056	6.1760228	0.330285	0.775784

Ggt1	0.4458184	1.7214991	0.3302994	0.775784
Slc5a3	0.2596252	3.8957089	0.3303794	0.775784
Heatr6	-0.1866179	4.8769742	0.3303926	0.775784
Tbc1d9b	-0.1303557	6.1504964	0.3304125	0.775784
Sppl3	0.1261699	6.1643652	0.3304159	0.775784
Usp34	-0.1375058	7.2349238	0.3304375	0.775784
Baiap2	-0.2203435	3.2251088	0.3305477	0.775784
Wbp4	0.114921	5.2733434	0.3305677	0.775784
Rock1	-0.1108554	7.8960882	0.3305835	0.775784
2510022D24Rik	0.2255904	3.5868964	0.3306666	0.7758618
6030443J06Rik	-0.3829303	-0.3768296	0.330737	0.7758875
Ndufb3	0.1658887	6.0713687	0.3307773	0.7758875
Tjp3	-0.2579068	2.2101659	0.3309504	0.7760624
Fra10ac1	0.2148828	3.2229347	0.3309516	0.7760624
Ccdc152	0.5209913	-0.0623394	0.3310498	0.7761755
Cdk2ap2	-0.1367808	6.2005287	0.3313095	0.7763327
Meis2	0.5113287	0.8284107	0.331348	0.7763327
Kansl1l	0.1881661	5.2670873	0.331415	0.7763327
Castor1	0.3854181	0.8754427	0.331465	0.7763327
Vamp7	-0.1706808	4.8649653	0.3314824	0.7763327
Slc35a1	0.1476886	4.1108299	0.3314975	0.7763327
Tmed2	0.111365	7.3090686	0.3314975	0.7763327
Dnajc1	-0.1250366	5.1283946	0.3315177	0.7763327
Prdm5	0.3195636	1.6004467	0.331566	0.7763327
Snx25	-0.1487793	4.6424673	0.3317234	0.7765582
Glb1l	-0.1464769	3.9285998	0.3317847	0.7765582
Nap1l3	-0.4849828	-0.4042869	0.3318121	0.7765582
Manf	0.1335809	7.0405518	0.3319977	0.7768538
Cyp4b1	0.2676003	2.4121905	0.3320536	0.7768538
Gm51716	-0.7754101	1.1724249	0.332187	0.7768538
Urb2	0.1674328	4.5595323	0.3322057	0.7768538
Gm13840	-0.4606267	0.0293465	0.3322112	0.7768538
Zfp715	-0.1447018	5.2897286	0.3322381	0.7768538
Gm38957	0.5516472	0.1192879	0.3323341	0.7769278
Aurkb	0.1293938	6.3064505	0.3324456	0.7769278
Mccc2	0.1825894	2.8993467	0.3325108	0.7769278
Churc1	0.1789633	3.9249366	0.332511	0.7769278
2900052L18Rik	-0.4280851	0.7755942	0.3326207	0.7769278
Nol7	0.1157083	6.7155339	0.3326525	0.7769278
6330418K02Rik	0.2925394	1.5454425	0.3326733	0.7769278
E130102H24Rik	-0.2771055	1.8530277	0.332702	0.7769278
Dgat2	-0.2245436	5.1303548	0.3327703	0.7769278
Amd2	0.4272822	1.7771147	0.332772	0.7769278

Ciao1	0.1229799	4.9822612	0.3328926	0.7769278
Lad1	0.9542094	-0.3681648	0.3329369	0.7769278
Rcan3	-0.1429119	3.9773983	0.3329395	0.7769278
Gm34121	0.7009621	-1.1326478	0.3329828	0.7769278
Zfp292	-0.1201898	6.3364811	0.3331564	0.7769278
Stk19	0.1708605	3.5288842	0.3332308	0.7769278
Uggt1	-0.1364815	6.7458154	0.333287	0.7769278
Ctso	-0.136393	4.3336183	0.3333118	0.7769278
Cdc14b	-0.2141219	2.3211337	0.3333893	0.7769278
Rrm2b	0.2117551	5.1329513	0.3333898	0.7769278
Bub1	0.1372867	6.559281	0.3334216	0.7769278
Trak1	0.1660537	5.1684392	0.3335266	0.7769278
Zfp821	0.1478803	3.937787	0.3335452	0.7769278
Gm45914	0.66244	-0.1696294	0.3335469	0.7769278
Gm41523	0.5606532	-0.3645074	0.3335861	0.7769278
Nudt7	-0.1910689	3.512216	0.3335974	0.7769278
Rabgap1	-0.1404631	5.1760033	0.3336879	0.7769278
Mthfd2l	0.3195104	1.3817245	0.3338222	0.7769278
Sugp2	0.1428115	4.8131592	0.3338682	0.7769278
Zfp748	-0.1550077	4.0770771	0.3338705	0.7769278
Bms1	0.1250701	6.1666892	0.3339467	0.7769278
Rab3gap1	-0.1065313	5.6116526	0.333996	0.7769278
Rpe	-0.1277691	6.27863	0.3340102	0.7769278
Ppp1r37	0.1267634	5.7989147	0.3340635	0.7769278
Trpm7	-0.1306575	7.0035206	0.3341436	0.7769278
Ccnq	0.1773565	3.2560619	0.3342195	0.7769278
Selenoi	-0.124937	5.1774703	0.3342206	0.7769278
Gm10073	0.2689464	2.3370249	0.3342687	0.7769278
Kansl3	0.1113744	5.7040082	0.3343406	0.7769278
Gm30368	0.3144096	0.6842273	0.334357	0.7769278
Klhl17	0.1710238	4.3210374	0.3343626	0.7769278
Ubr2	-0.1289589	6.8368847	0.3343677	0.7769278
Nyap2	-0.3357498	1.649721	0.3346091	0.7771276
Gch1	0.175469	6.4090385	0.3346246	0.7771276
Pepd	0.1608598	4.0657735	0.334631	0.7771276
Ppp6r3	-0.1243046	7.4596857	0.3346706	0.7771276
Picalm	0.1411149	9.2263614	0.3347035	0.7771276
Gm5422	0.658352	-0.8179094	0.3348433	0.7773247
Prr14	0.116127	6.0855171	0.3349023	0.7773247
LOC118568058	-0.5214611	-1.008719	0.3349383	0.7773247
Aurka	0.1400577	6.4176568	0.3350466	0.77746
Gm36681	-0.3621521	0.4241045	0.3352742	0.7777019
Dck	0.1153813	7.6099967	0.335286	0.7777019

Cops8	0.1122477	5.3423088	0.3353414	0.7777019
Glis3	-0.3136736	2.2744585	0.3353508	0.7777019
H2-D1	-0.1355938	9.6192367	0.3354303	0.7777364
Mrpl35	0.1882092	4.81491	0.3354988	0.7777364
Actr3-ps	-0.2223126	2.0294158	0.3355157	0.7777364
Gm30541	0.4461924	0.2623089	0.3356896	0.778018
Pigv	-0.1557063	3.9776651	0.3357373	0.778018
Rwdd4a	0.1148922	4.8725222	0.3359458	0.7782106
Oxct1	-0.1077093	6.8172324	0.335978	0.7782106
Setd3	0.1039338	6.7198444	0.3359802	0.7782106
Robo2	-0.3604926	3.5935045	0.336049	0.7782106
Stard3	-0.1428368	4.4980889	0.3360705	0.7782106
Tax1bp1	-0.1105496	8.1914062	0.3362263	0.7783087
Ncald	0.2932232	2.2320155	0.3362306	0.7783087
Kat6a	-0.1571275	7.3225878	0.336263	0.7783087
Tmem161a	0.1683702	4.3707336	0.3363776	0.7784179
Tacc1	-0.1137272	7.5775977	0.3364723	0.7784179
Unc5b	0.3111899	5.2148229	0.3364978	0.7784179
Gm35315	-0.4619325	0.0410514	0.3365174	0.7784179
Pafah1b1	-0.1014068	7.9697111	0.3365604	0.7784179
Gm7488	0.3605846	0.8033022	0.3367067	0.7786405
Ddit3	0.1924227	4.2445942	0.3368307	0.7788115
B4galnt4	-0.5067984	-0.8847527	0.3369593	0.778993
Tube1	-0.1920825	3.3412801	0.3371196	0.7792478
Nip7	0.1666837	5.0012462	0.3371706	0.7792499
Furin	-0.1172848	6.3353163	0.337339	0.7795233
Arfip2	0.1755208	4.3965416	0.337569	0.7798424
Atxn3	-0.1159196	5.9614901	0.3376374	0.7798424
Iqcb1	0.1922792	3.0023866	0.3376807	0.7798424
Gm6563	0.1810313	3.9400804	0.3376972	0.7798424
Lrrc15	-0.732505	-0.4951818	0.3377299	0.7798424
Pygb	-0.1454265	5.2667176	0.3377817	0.7798424
Zfp959	0.1597162	3.5480352	0.3378738	0.7798424
Cryl1	0.1721023	3.6150596	0.3379703	0.7798424
Dhfr	0.1601408	6.0143345	0.3380006	0.7798424
Arsi	-0.5953551	-0.2452634	0.3380912	0.7798424
Elp3	0.1231971	5.1515592	0.3380954	0.7798424
Adamts10	0.1633114	5.0567346	0.3382603	0.7798424
Rpl37a	0.1687045	8.7370525	0.3383137	0.7798424
Ufc1	0.1098093	5.6226363	0.3383724	0.7798424
3110070M22Rik	-0.7749375	-0.9633444	0.3384062	0.7798424
Traf4	0.1884462	5.0278323	0.3384552	0.7798424
LOC118568094	-0.275152	3.3122265	0.3384667	0.7798424

Raver2	0.5326518	-0.9534971	0.3384974	0.7798424
Ube2d1	0.1647719	4.416569	0.3385223	0.7798424
Emc9	0.2075242	2.8825544	0.3385846	0.7798424
Zswim4	-0.2000086	4.0099883	0.3385915	0.7798424
Tlr9	0.3067271	3.3970901	0.3386522	0.7798424
Cstb	0.1741821	5.2510107	0.338654	0.7798424
Slc30a4	0.2073848	4.2263822	0.3386804	0.7798424
Per1	0.4484297	5.2984364	0.3387698	0.7799255
Nifk	0.1188398	5.649866	0.338822	0.7799255
Extl2	-0.1666144	3.5818624	0.3389001	0.7799255
Nop16	0.140299	4.5749727	0.3389689	0.7799255
Gtf2h4	0.1812594	3.5112935	0.3390246	0.7799255
Ints9	0.1256661	4.8958488	0.339045	0.7799255
Lmo1	0.2488975	2.7157937	0.3391319	0.7799255
Faap100	0.2090598	3.0443372	0.339134	0.7799255
Ppip5k2	-0.1297776	6.2326041	0.3391759	0.7799255
Sbno1	-0.1295469	7.2707001	0.339218	0.7799255
Ikzf2	-0.2344312	3.0596754	0.3393319	0.7799693
Xkr5	0.2449211	2.384699	0.3394115	0.7799693
Gm39547	-0.4731646	0.0519601	0.3394762	0.7799693
Dis3l2	0.1547807	4.029713	0.3394864	0.7799693
Hmgcl	-0.141338	5.312271	0.3394877	0.7799693
Rtn2	-0.4292501	0.8236932	0.3397399	0.780364
Gpr108	-0.1246258	5.1430055	0.339773	0.780364
Gm5900	-1.0034036	1.6134992	0.3398248	0.780364
Tdrkh	0.4149602	1.0890338	0.3398602	0.780364
Slc3a2	0.137896	7.0288361	0.3399842	0.7804741
Chchd6	0.2551272	2.2864814	0.3400308	0.7804741
Acsl3	0.1886695	3.6886632	0.3400891	0.7804741
Ywhaq	-0.1065802	7.0487808	0.3401252	0.7804741
Chsy1	-0.1332862	6.3541405	0.3402403	0.7804741
Tm9sf3	-0.1125252	8.2014327	0.3402797	0.7804741
Unc13a	0.6135678	-0.7459252	0.3402825	0.7804741
Psma5-ps	-0.4574963	-0.5453291	0.3403096	0.7804741
Srsf5	0.1611188	8.3371337	0.3403616	0.7804783
Eomes	0.3168132	0.9759858	0.3404473	0.780513
Il13ra1	-0.1596251	4.8069135	0.3404771	0.780513
Ptger3	-0.2416726	2.1415327	0.3407304	0.7809785
Ccn1	0.5550764	4.8354862	0.3408002	0.7810236
Zfp938	-0.273832	1.4608086	0.3410009	0.7812875
Gm2093	-0.5406565	-0.686822	0.3410159	0.7812875
Wdr53	0.1739092	3.5577455	0.341107	0.7813812
Carhsp1	0.1382204	7.6289712	0.3412138	0.7815108

B3gntl1	-0.2262211	2.430845	0.3413265	0.7815853
Rnase4	-0.1532256	4.9995245	0.3413468	0.7815853
Rasgrp2	-0.1236784	7.3791989	0.3416024	0.7818297
Dusp12	0.1859797	2.8821948	0.34161	0.7818297
Podxl	-0.2093869	3.5251229	0.341665	0.7818297
Ttk	0.1419819	5.3750342	0.3417665	0.7818297
Cngb1	-0.4626137	-0.2084682	0.3417698	0.7818297
Abca6	-0.4064697	1.5715332	0.3418604	0.7818297
Enc1	0.1884983	3.3499464	0.3418703	0.7818297
Lrrn3	0.2745764	2.9369595	0.3418994	0.7818297
Rassf5	-0.1712389	6.2558281	0.341906	0.7818297
Suco	-0.1389687	6.5871181	0.3420675	0.7819204
Klhl9	-0.1011032	6.680509	0.3420814	0.7819204
Gpalpp1	-0.1209855	5.6925856	0.342136	0.7819204
Gm5499	0.6043418	-0.68636	0.3421602	0.7819204
Adgra2	0.2345207	3.5505562	0.342197	0.7819204
Fbxo27	-0.4466501	-0.6824758	0.3422517	0.7819306
Sdc1	0.1936319	5.2312361	0.3424109	0.782144
Anapc16	0.1183732	5.4053513	0.3424943	0.782144
Hus1	0.133807	4.825865	0.342496	0.782144
Pnn	0.1348797	7.447453	0.342571	0.7822005
Tuba5-ps	0.5399206	-0.2426068	0.3426536	0.7822741
Smurf2	-0.1344107	5.9553881	0.3429913	0.7828644
Lamb3	0.5960569	-0.8380815	0.3430128	0.7828644
Fndc5	0.7508332	-0.5711414	0.3432509	0.7832929
9830166K06Rik	-0.2372074	2.8532613	0.343435	0.7835758
Gpx3	-0.2374546	7.868297	0.3434887	0.7835758
Gm38733	0.2350334	3.5084059	0.3435407	0.7835758
Cd200r3	0.533821	0.0790835	0.3436461	0.7835758
Gipc1	-0.1337936	5.3183618	0.3436464	0.7835758
Shmt1	0.1952703	3.345783	0.3436772	0.7835758
Kmt2a	-0.1715899	6.9241912	0.3439571	0.7836424
Tmco1	-0.097182	6.0084373	0.3439971	0.7836424
Thoc5	0.148114	4.6172224	0.3440285	0.7836424
Chst13	-0.2812666	2.8285498	0.3440512	0.7836424
Eri3	0.1369821	4.4669044	0.3440634	0.7836424
Ccdc59	0.1691219	4.609598	0.3440754	0.7836424
Hdgfl2	0.1135988	5.7505022	0.3441055	0.7836424
Mcts1	0.1461256	4.8812987	0.3441094	0.7836424
Gm16433	0.6282879	-1.0054395	0.3442797	0.7837301
Kcnmb4	0.4160501	-0.4454084	0.3443072	0.7837301
She	-0.2762562	2.3104921	0.3443696	0.7837301
Arhgap30	-0.1588973	7.4274232	0.344459	0.7837301

Gins3	0.146182	4.3859242	0.3444823	0.7837301
Igfals	-0.4679497	-0.5538663	0.3444948	0.7837301
4833422C13Rik	0.5103785	1.8024638	0.3445006	0.7837301
Gm10561	-0.5459338	-0.0132592	0.3446433	0.7838051
Pdlim5	-0.1232311	5.5120304	0.3446659	0.7838051
Il17rd	-0.4382033	0.3927361	0.3447537	0.7838051
Cemip	-0.3233945	3.6347244	0.3447569	0.7838051
Wdfy4	0.2042742	6.7135382	0.3448234	0.7838051
Rbfox3	0.4111226	-0.3043218	0.344836	0.7838051
Gm7514	-0.3971127	-0.5964577	0.3448956	0.7838159
Atp6v0b	0.1363973	6.6329356	0.3450742	0.7838159
Nsun2	0.1130546	6.5872503	0.3451103	0.7838159
Gpr55	-0.3290053	0.9536366	0.3451347	0.7838159
Grb14	0.2710148	2.7892735	0.3451579	0.7838159
Gm36268	-0.521355	-0.0985823	0.3453334	0.7838159
B230354K17Rik	0.1382699	4.0467407	0.3453368	0.7838159
Mfn2	-0.1112965	5.6819593	0.3455266	0.7838159
4930402H24Rik	-0.1421628	4.1232773	0.3455288	0.7838159
Gm33862	-0.5523465	0.3717411	0.345584	0.7838159
Papln	-0.4056929	0.1706865	0.3455886	0.7838159
Med9	0.1312954	4.32509	0.3456205	0.7838159
L1cam	-0.2176092	3.1222325	0.3456702	0.7838159
Scrn1	0.5413102	1.4425132	0.3456914	0.7838159
Gm26944	-0.2187066	3.3976127	0.3457168	0.7838159
Ankib1	-0.1142948	5.4758925	0.3458407	0.7838159
Foxj2	-0.130659	4.7651574	0.3458915	0.7838159
Pmepa1	0.2744763	4.8001012	0.3458928	0.7838159
Ift20	0.138359	5.6046417	0.3459229	0.7838159
Dqx1	0.3134379	1.9144061	0.3459278	0.7838159
Mvp	0.112289	5.168142	0.3459873	0.7838159
Trip6	0.1979239	3.6303007	0.3460122	0.7838159
Pdlim1	0.1214667	5.5542497	0.3460404	0.7838159
Heatr5b	-0.171132	4.7265788	0.3460607	0.7838159
Anks1	-0.1343357	5.2758721	0.3461005	0.7838159
Cttnbp2	0.3296281	1.0780808	0.3462311	0.7839975
Gm15428	-0.5361023	0.4327795	0.3463324	0.7841126
Ltc4s	0.3097924	1.4450173	0.3464006	0.7841504
Nfatc1	-0.1380191	6.5257999	0.3464499	0.7841504
3830403N18Rik	-0.4818724	0.9760541	0.3468063	0.7846479
Dlc1	-0.1771535	4.9927331	0.3468088	0.7846479
Tmem150a	0.2956926	1.7797015	0.3469108	0.7846479
Soga1	-0.1563143	5.1205531	0.3469378	0.7846479
Gm21992	0.3030242	1.1664861	0.3469644	0.7846479

Afdn	-0.1687904	4.6994188	0.3469724	0.7846479
Rpl18	0.1267104	8.9041449	0.3471756	0.7847958
Washc1	0.1333786	4.8016657	0.3472048	0.7847958
Cyb5r1	-0.111353	5.5432525	0.3472584	0.7847958
Psma4	0.1395568	7.0891855	0.3473178	0.7847958
Zzz3	-0.1162052	5.8131595	0.3473535	0.7847958
Pla1a	-0.5080494	0.3054842	0.3474701	0.7847958
Sumo2	-0.1119804	6.5064925	0.3474757	0.7847958
Asrgl1	0.1292179	4.6652395	0.3475521	0.7847958
Esco1	-0.0977343	5.82301	0.3475793	0.7847958
Ift80	0.1406898	5.6276629	0.347641	0.7847958
Terf2	0.1356487	5.3615783	0.3477587	0.7847958
Slc36a1	-0.1435842	5.1121956	0.3477643	0.7847958
Ip6k1	0.119432	7.2057427	0.3477824	0.7847958
Bap1	0.1051398	5.7250176	0.3477971	0.7847958
Spr	0.1731672	3.9155492	0.3478206	0.7847958
Pex11b	0.1531992	3.7947234	0.3478838	0.7847958
Csmd1	-0.2946359	1.8318788	0.3478955	0.7847958
Irak4	-0.1485799	4.5128774	0.3479971	0.7848635
Ulk3	0.2206229	3.1054559	0.3480265	0.7848635
Bdh1	0.2012777	3.1188824	0.3482154	0.7851381
Ddost	0.1320877	7.2614147	0.3482492	0.7851381
Aggf1	-0.1122443	6.0863093	0.3483971	0.7852543
Myo1d	-0.1736023	5.9818546	0.3484418	0.7852543
Gm51425	0.203055	2.7893735	0.3484522	0.7852543
Pwwp2a	0.1233991	5.8697852	0.3485862	0.7854411
Cdh1	-0.295284	1.9766844	0.348636	0.7854411
Kmt5b	-0.1149235	6.1306382	0.3487471	0.7855407
Pigbos1	0.2080569	4.0425305	0.3487813	0.7855407
Btnl10	0.1978892	7.4904151	0.3490577	0.7855795
Map3k12	0.3206192	1.4918564	0.3491006	0.7855795
Gpc2	0.3124476	2.2214909	0.3491405	0.7855795
Sit1	0.397329	0.6525569	0.3491504	0.7855795
Pik3ap1	0.2117752	6.7023399	0.3492698	0.7855795
Cgnl1	-0.2657873	3.5709884	0.3493035	0.7855795
1810058I24Rik	0.1286948	6.6001796	0.3493038	0.7855795
Mapk8ip3	0.120451	5.6410911	0.3493246	0.7855795
Sars	0.118925	6.6395409	0.3493729	0.7855795
Gm33934	0.5154596	-0.2042175	0.3493846	0.7855795
Slfn10-ps	-0.3621606	0.8893578	0.3494663	0.7855795
Clta	0.1125688	7.6228278	0.3494732	0.7855795
Phc3	-0.1011582	5.9640573	0.3495178	0.7855795
Gm31814	-0.2612961	2.0405156	0.3495253	0.7855795

Klf10	-0.125344	5.5059394	0.3495791	0.7855795
Mycbp2	-0.1259663	6.8587612	0.3496066	0.7855795
Pofut2	0.1550986	4.9193061	0.3499303	0.7857773
Pik3r5	-0.1550432	5.6020898	0.3499482	0.7857773
Rps6ka4	0.1357112	4.6696079	0.3500159	0.7857773
Gm12366	-0.5868639	-1.0450913	0.3500384	0.7857773
Arhgef26	0.4274147	1.0083333	0.3500678	0.7857773
2210408I21Rik	-0.3174406	1.5086013	0.3500728	0.7857773
Eva1b	0.2581098	2.7546645	0.3502313	0.7857773
Isoc1	-0.1190495	5.5074128	0.3502414	0.7857773
Mrm3	0.231767	2.8599975	0.350262	0.7857773
Btg2	0.1248724	7.5047711	0.3503043	0.7857773
Acads	0.1347553	4.3712025	0.3503888	0.7857773
Bloc1s1	-0.1451027	5.2579391	0.3504128	0.7857773
Gm40194	-0.4730004	-0.2352212	0.3504921	0.7857773
Gas2l1	-0.1508465	5.8077849	0.3504949	0.7857773
Azin2	0.3628466	0.3136997	0.3505157	0.7857773
Rpl23a-ps3	0.2156959	1.9849599	0.3505651	0.7857773
Akr1e1	-0.2251371	3.1776263	0.3506044	0.7857773
Gm38515	-0.371158	0.2848665	0.3506239	0.7857773
Efemp2	0.2792579	4.2984089	0.3506646	0.7857773
Slc20a1	0.1624928	7.0913812	0.350705	0.7857773
Trib3	0.4660672	-0.0024844	0.3510306	0.7863835
Rpl18-ps2	0.2717693	1.7786456	0.3510767	0.7863835
Arsk	-0.1745952	3.5018354	0.3511457	0.7864248
Thnsl2	-0.4215534	-0.0231041	0.3513158	0.7866926
Zdhhc6	0.1086339	5.551225	0.351526	0.7870499
Med11	-0.1380726	4.2544966	0.3516794	0.7871833
Mto1	0.1415516	4.3472997	0.3516868	0.7871833
Prpf39	0.1119493	6.0651401	0.3517409	0.787191
Faim2	-0.5949198	-0.5402392	0.3518234	0.7871958
Rab42	-0.5872252	-0.831388	0.3518442	0.7871958
Rpl10a-ps1	0.1523753	4.8090828	0.3519852	0.7873341
Chd1l	0.1348889	4.8611483	0.3520418	0.7873341
Kif22	0.1425712	6.9120649	0.3520579	0.7873341
Lmna	0.1442146	7.2617329	0.3522585	0.7876371
Atp8b5	-0.5866062	-1.246629	0.3522947	0.7876371
Gpi1	-0.1583962	8.8961942	0.3524329	0.7876542
Tert	0.4684508	-0.1226509	0.3524989	0.7876542
Snrpc	0.1068689	5.4940338	0.3525028	0.7876542
Scml4	0.3130481	2.1258435	0.3525049	0.7876542
Pom121	0.1335625	6.3143801	0.35257	0.7876865
Gid8	0.1578181	7.4242239	0.352718	0.7879039

D930003E18Rik	-0.2854333	1.3258133	0.3528668	0.7881231
Pex11a	-0.2767111	1.7280372	0.3529562	0.7882098
Hgh1	0.2402435	2.9919258	0.3532323	0.7885798
Acy3	0.242612	1.5458385	0.3532616	0.7885798
Tnip3	0.6493914	-0.9652962	0.353274	0.7885798
Sephs1	0.1022362	6.0583415	0.3534557	0.7888721
Vipas39	-0.121988	4.6739036	0.3536111	0.7890086
Slc2a12	-0.4640135	-0.8613348	0.3536183	0.7890086
Ankrd13b	0.1960924	2.9739409	0.3537217	0.7891261
Irf5	0.1448977	5.2690175	0.3538934	0.789396
2310030G06Rik	-0.4065076	0.469256	0.3539482	0.7894049
Tmem181a	-0.2041356	3.5346226	0.3540657	0.7894756
Col13a1	-0.3445641	5.2426437	0.3540965	0.7894756
Mtm1	-0.1790864	4.0901967	0.3541483	0.7894756
LOC118567627	0.3914976	0.5805093	0.3541829	0.7894756
Ly6g6d	-0.2958311	1.5189218	0.3543802	0.7896481
Fam220a	0.2288919	6.8248433	0.3543855	0.7896481
Ppfibp1	-0.1979985	5.0663499	0.3544869	0.7896481
St3gal6	0.1413387	6.2429037	0.354538	0.7896481
Nr4a3	-0.5201264	-0.8782826	0.3545775	0.7896481
Xlr4a	0.3201492	1.598579	0.3545805	0.7896481
Mettl5	0.1675979	3.6413796	0.3546157	0.7896481
1810013L24Rik	-0.1021762	6.0904837	0.3547506	0.7898355
Tmem134	0.1145206	5.7605003	0.35484	0.7898589
Lmf2	0.1104925	5.5190543	0.3548637	0.7898589
Zfp831	-0.1639145	3.8054338	0.3549316	0.7898589
Cwc22	0.1210149	5.5189049	0.3549738	0.7898589
Clspn	0.1553421	6.9684943	0.3550718	0.7898589
Kynu	0.3741529	1.5263917	0.355171	0.7898589
Tcf20	-0.1215877	6.3893467	0.3551866	0.7898589
Gm8680	0.4762005	-0.2452417	0.3552911	0.7898589
Lhpp	0.1833309	2.9880148	0.3552985	0.7898589
Vps50	0.1453775	4.6430031	0.3553063	0.7898589
Frs2	-0.1151538	5.6520389	0.3553416	0.7898589
Myct1	-0.1572754	4.1105442	0.3553705	0.7898589
Lrrc32	-0.1836969	4.8038044	0.3555233	0.7899209
Erlec1	0.1390928	5.6745529	0.3555477	0.7899209
Gm16548	-0.498418	-0.0250312	0.3556398	0.7899209
Shld1	0.2564379	2.5700832	0.3556489	0.7899209
Cox5a	0.1249197	6.3257191	0.3556523	0.7899209
Gosr2	-0.105926	6.4559297	0.355887	0.7902204
Dpy19l1	0.1117667	6.5459568	0.3558888	0.7902204
Ints6	-0.1280731	5.7179127	0.3559599	0.7902655

Gm8666	0.4281729	0.2198941	0.3560665	0.7903894
Fbln7	-0.4352473	1.1542508	0.3561647	0.7904946
Dyrk3	0.2087867	4.9619815	0.3564179	0.7907594
Impdh1	0.1193945	5.4589378	0.356443	0.7907594
Rfc2	0.1113844	6.4321863	0.3564862	0.7907594
Cdk5r1	-0.1542455	3.8006339	0.3564874	0.7907594
Ndufb11	-0.1313598	6.4984436	0.3566529	0.7908381
Hnrnpc	0.1153667	8.3486483	0.3566711	0.7908381
Gm17669	-0.4064607	0.1561085	0.3567376	0.7908381
Aldoc	-0.3815851	1.4906146	0.3567647	0.7908381
Ascc1	0.1354497	4.3201049	0.3568425	0.7908381
Rbm19	0.1568346	3.8058082	0.3569781	0.7908381
Kif18b	0.1476176	6.2028165	0.3569875	0.7908381
Slc23a2	0.1286181	4.3382222	0.3570052	0.7908381
Crebbp	-0.1242324	6.4011814	0.3570227	0.7908381
Ccnl1	0.1257133	7.222306	0.3570958	0.7908381
Gm41958	0.4884338	-0.6768361	0.3571523	0.7908381
Ssh2	-0.1644082	7.118349	0.357173	0.7908381
Rnls	-0.3845557	0.6307554	0.3571839	0.7908381
Rpf2	0.1692561	4.7260529	0.3572658	0.7908491
Sfswap	0.1041389	6.1372597	0.3573206	0.7908491
D230025D16Rik	-0.1354855	4.6509819	0.3573414	0.7908491
Slc4a3	0.5291954	0.1789674	0.357529	0.7909161
Gm8034	-0.3887592	0.2778506	0.3575521	0.7909161
Nfkbil1	0.1389428	3.6474776	0.3575956	0.7909161
Spast	0.0951564	5.8804037	0.3576062	0.7909161
Dkc1	0.1236329	6.367872	0.357686	0.7909161
Gm30968	0.7791395	-0.8199127	0.3577078	0.7909161
Zfp703	-0.1296639	4.659837	0.3577895	0.7909161
Cntrl	-0.1447948	7.07237	0.357861	0.7909161
Erh	0.1154782	6.2806134	0.3579028	0.7909161
Gm10157	-0.3645685	0.6647224	0.3579169	0.7909161
Tent5a	-0.1643598	6.5441541	0.3579587	0.7909161
Tmem192	0.1499998	3.6116774	0.358003	0.7909161
Brip1	-0.1439849	4.9457106	0.3580327	0.7909161
Rrp1b	0.1445637	4.408146	0.3581024	0.7909389
Nxf1	0.1162702	7.2362752	0.3581447	0.7909389
Epdr1	0.1374237	5.2437832	0.3582516	0.7910626
Nfkbie	0.1773477	4.1885924	0.358694	0.7915397
Cdc34	0.1174009	6.3013755	0.358705	0.7915397
1700030K09Rik	0.2353654	2.1305209	0.3587419	0.7915397
3110009E18Rik	-0.2450762	1.5236507	0.358818	0.7915397
Gm41442	0.6365981	-0.3974104	0.3589029	0.7915397

Brms1l	0.1565419	4.5604503	0.3589049	0.7915397
C130074G19Rik	0.2283933	3.4749225	0.3589132	0.7915397
Zfp704	-0.148973	4.791056	0.3589252	0.7915397
Lmo2	0.1540303	7.9573419	0.3589256	0.7915397
Man2a1	-0.1410784	6.7642138	0.3591626	0.7919168
Tctex1d1	0.8332546	-0.696925	0.3591985	0.7919168
Rnf41	-0.1398123	5.0875631	0.3594413	0.7923398
Crebzf	0.1188327	6.8131715	0.3595321	0.7924277
Snorc	-0.481411	2.0377183	0.3597055	0.7926548
Ghdc	-0.184887	2.4847765	0.3597371	0.7926548
Plcb3	-0.1350712	4.7572121	0.3599497	0.7928835
Rnf138	0.1274145	4.410811	0.3599858	0.7928835
Zbtb8os	0.1659512	4.8632613	0.3599938	0.7928835
Bcor	0.1197756	5.9222467	0.3602367	0.7933062
Kbtbd8	-0.1738508	3.7290249	0.3603289	0.7933171
Taf10	0.1054545	6.2067133	0.3603436	0.7933171
Icam1	0.1639155	4.0497211	0.3604515	0.7934422
Pitrm1	0.1472032	6.723294	0.36055	0.7935467
Cerkl	0.4815418	0.4088094	0.3608527	0.7939162
Actl6a	0.12213	6.0421035	0.3608573	0.7939162
Adamdec1	0.619113	0.4455911	0.360899	0.7939162
Zfand3	-0.1222573	6.1233518	0.3609221	0.7939162
Hinfp	-0.1158648	5.2549098	0.3611434	0.7939977
Nbdy	0.1623969	3.4844296	0.3611537	0.7939977
Gm36445	0.3345904	1.9951701	0.3611609	0.7939977
Vps8	-0.1386944	4.1872682	0.3611788	0.7939977
Tada1	0.1163449	4.6649609	0.3612259	0.7939977
Gm52945	-0.2043412	3.4667692	0.3612743	0.7939977
Gm39913	0.6415561	-0.9784721	0.3614156	0.7939977
Uqcrq	0.1493466	6.3819541	0.3614172	0.7939977
Rps2-ps6	0.3942742	0.0241584	0.3614185	0.7939977
Pomp	0.1429091	6.8328545	0.3615063	0.7940783
Dlg5	-0.2081765	4.2360747	0.3616814	0.7942042
Hnrnpk	0.1047698	9.4238939	0.3617483	0.7942042
Sdr42e1	-0.2750057	1.8095536	0.3618173	0.7942042
Bloc1s5	0.1645192	4.0855337	0.3618283	0.7942042
Thsd1	0.3148881	1.0906154	0.3618399	0.7942042
Hspa14	0.0997749	6.3939338	0.3618923	0.7942042
Ankrd11	-0.1346161	7.2504622	0.3619629	0.7942042
Plrg1	0.1101932	5.6564366	0.3619721	0.7942042
Rnf215	0.1757171	3.6668547	0.3621621	0.7945092
Pcsk7	-0.1159894	5.245711	0.3624386	0.7948712
Klhl3	-0.3286351	0.1505348	0.3624943	0.7948712

Ms4a7	0.2585909	3.2939259	0.3625442	0.7948712
Ubtd2	0.244149	3.4578451	0.3625484	0.7948712
Msi1	0.3875662	1.8974739	0.3626166	0.7948712
Iqcc	0.1890789	3.1066291	0.3626338	0.7948712
Rfesd	-0.2417264	4.497569	0.3627977	0.7950671
Ppp1r3d	-0.1411109	4.4408783	0.3628848	0.7950671
Mmadhc	0.1047811	5.713448	0.3629216	0.7950671
Zfp710	-0.158133	6.3780392	0.363056	0.7950671
Acer2	0.2399859	2.2622083	0.3630678	0.7950671
Ids	-0.1259181	5.1685112	0.3630799	0.7950671
Cox11	0.1815258	4.3889051	0.363081	0.7950671
Hba-a1	0.3083046	14.309324	0.3632794	0.7953897
Hck	-0.1377664	6.4248662	0.3634186	0.7955029
Fam169b	0.3115606	2.3525433	0.363445	0.7955029
Rabggtb	0.2188703	5.960907	0.3635343	0.7955029
Commd1	-0.1363758	5.3256669	0.3635357	0.7955029
Senp3	0.1040733	5.8337784	0.3636518	0.7955222
Fgd2	0.1834569	4.1259714	0.3636578	0.7955222
Mtif2	0.1236597	4.9203933	0.3637706	0.7955222
Gm5431	-0.3309391	0.790986	0.3639812	0.7955222
Calml3	0.4840574	1.8440268	0.3640664	0.7955222
Iah1	0.1474485	3.889274	0.3641667	0.7955222
LOC115488470	0.3697758	0.1319626	0.3641986	0.7955222
Itgav	-0.2049621	6.7385695	0.3642287	0.7955222
Fus	0.1208029	8.5008118	0.3642655	0.7955222
Ccdc77	0.1549821	4.6591073	0.3643367	0.7955222
Tstd3	0.1437134	4.3103622	0.364435	0.7955222
Faf2	0.1013395	5.464461	0.364457	0.7955222
Tmem30b	0.6183098	0.0634618	0.3644734	0.7955222
Tpt1	0.1678071	10.938309	0.3644785	0.7955222
Tnfaip8	-0.1213704	6.7060816	0.3645419	0.7955222
Gins4	0.1187717	5.7707591	0.3645651	0.7955222
Lmnb1	0.1261692	8.4230688	0.3645733	0.7955222
Rbx1	0.1526892	6.3773932	0.3645888	0.7955222
Zfyve21	0.1900506	3.3712185	0.3646013	0.7955222
Capn1	-0.1155615	7.0883061	0.3646103	0.7955222
Pld4	0.1310602	6.0257179	0.3646577	0.7955222
Lmntd2	0.4728559	-0.3028883	0.3649592	0.7955222
Ltbp4	-0.2371745	2.8912742	0.3650012	0.7955222
Dcun1d4	-0.2404997	2.8496486	0.3650099	0.7955222
Whrn	0.2856174	2.6643253	0.3650151	0.7955222
Ap3m2	-0.1522148	3.3512631	0.3650307	0.7955222
Msh3	0.1722028	4.7989278	0.3650626	0.7955222

Pex26	-0.2177577	3.0068509	0.3651028	0.7955222
Gm13394	-0.1582159	6.5889991	0.3651237	0.7955222
Rpl36a	0.2084443	9.0847096	0.3651725	0.7955222
4631405J19Rik	-0.6104112	-1.0547329	0.3652294	0.7955222
Slc9a3r2	-0.1742412	3.2124944	0.3652861	0.7955222
LOC100042019	-0.6371804	-0.5609968	0.3653011	0.7955222
Rps18-ps3	0.5171855	-0.896915	0.3653652	0.7955222
Atp23	0.2405127	2.0386758	0.3654065	0.7955222
Sele	0.1792017	3.7158778	0.3654193	0.7955222
Gm21399	0.4841805	-0.7657939	0.3655089	0.7955222
Mapk6	-0.1094999	6.8736079	0.365626	0.7955222
Larp7	0.1225196	6.1970476	0.3656781	0.7955222
Tinf2	0.1057414	5.2182743	0.3656836	0.7955222
Zkscan8	-0.1369833	4.5270572	0.3657163	0.7955222
Pdcl3	-0.1075659	5.4056214	0.3657209	0.7955222
Marveld1	-0.1374969	5.216795	0.3657438	0.7955222
Gm6710	0.5613538	-1.0482424	0.3658786	0.795543
Hdlbp	-0.1142116	8.2087437	0.3660048	0.795543
Rtl8b	0.2658045	1.8386846	0.3661238	0.795543
Incenp	0.1169108	7.6628415	0.3661327	0.795543
Lrfn1	-0.326659	0.2217901	0.3661416	0.795543
Nr2c2ap	0.2035572	3.5078611	0.3661628	0.795543
Rrp8	0.1639629	4.3850023	0.3661765	0.795543
Col9a1	-0.6693675	4.5638001	0.3662554	0.795543
H3c6	0.6363208	-0.8233442	0.3662728	0.795543
Tbc1d5	-0.1747585	5.022176	0.3663338	0.795543
Zfp148	-0.1055211	6.4782091	0.3663625	0.795543
Gm4221	-0.3053227	0.6654032	0.3663671	0.795543
Tst	-0.1752097	3.8253275	0.3664507	0.7956133
Ifi35	0.1368789	4.0896697	0.3665668	0.7957543
Aspscr1	0.1212613	4.9584847	0.3667178	0.7959711
Lrrc17	-0.4101786	1.208848	0.3668471	0.7960785
Nup107	-0.1251463	6.1557802	0.3668697	0.7960785
Tcea3	-0.3933138	1.0574224	0.3670342	0.7962986
Banp	0.240317	4.8405945	0.3671815	0.7962986
Nqo1	0.1896071	3.9265436	0.3672331	0.7962986
Igf2bp2	-0.3157276	1.3488807	0.3672487	0.7962986
9230116N13Rik	0.3042911	1.0744844	0.3672703	0.7962986
Gm40185	-0.5875233	-0.235983	0.3673346	0.7962986
Arhgap1	-0.1187071	6.0319755	0.367412	0.7962986
LOC118568289	-0.5260116	0.1846347	0.3674384	0.7962986
Gstk1	-0.2365586	1.8614442	0.3674765	0.7962986
Bcl6	-0.1509614	5.4736654	0.3674831	0.7962986

Ppp2r5d	0.1139395	5.5524729	0.367676	0.796341
Pml	0.1279075	6.6632708	0.3676979	0.796341
Gm18860	0.5318704	-0.7112589	0.3677753	0.796341
Smagp	0.2455404	2.7223969	0.3678185	0.796341
Zmym1	0.149294	4.2887743	0.3678203	0.796341
Alg1	0.1759309	3.4225329	0.3679213	0.796341
Epop	-0.2917712	1.6458896	0.3679241	0.796341
Gm35154	-0.626886	-1.1888123	0.3679367	0.796341
Naa15	0.1053687	6.9489681	0.3680031	0.796341
Tom1l2	-0.1265077	4.1663232	0.3680539	0.796341
Tnfrsf10b	0.3538836	0.9697727	0.3680658	0.796341
Irf8	0.1287729	6.6229092	0.3682875	0.7967099
Hsp90b1	-0.1091374	9.3519479	0.3684673	0.7969879
Psph	0.2106548	3.355743	0.3685611	0.7970801
Dnaja4	0.1707735	5.6662143	0.3686205	0.7970977
Nol6	0.1422125	5.1390936	0.3687353	0.7971876
Gm13205	-0.4868036	-0.7153734	0.3687646	0.7971876
Ppp2r3d	0.151865	4.4191239	0.368887	0.7973415
Tdg-ps2	0.1411492	4.2364729	0.369011	0.7974986
Bambi-ps1	0.316367	0.5219404	0.3691115	0.797605
Cnbd2	-0.2082481	2.3523824	0.3691906	0.797665
Sarnp	0.1270349	6.5542841	0.3693179	0.797793
Rfc5	0.1200677	5.547633	0.3694385	0.797793
Gm7935	0.4021027	-0.7100706	0.369456	0.797793
Mcpt8	-0.2154879	4.8099646	0.3695185	0.797793
Mark3	0.10864	6.2102562	0.3695436	0.797793
Cfap298	0.1746541	3.5715262	0.3695576	0.797793
Itga1	-0.1770484	4.7299888	0.3696867	0.7979487
Ctbp2	-0.1164263	4.7616358	0.3697842	0.7979487
Stx18	0.1233401	4.2008664	0.3698531	0.7979487
Slc7a6	0.160547	3.9670291	0.3698746	0.7979487
Gm19299	-0.537477	0.4859223	0.3699219	0.7979487
Gm2296	0.2327629	1.9608896	0.3699375	0.7979487
Srr	0.1328004	4.0239711	0.3700201	0.7980161
Rffl	-0.1142894	6.3149022	0.3701241	0.7981298
Cenpq	-0.1662594	5.526764	0.3703136	0.7984276
Trim7	0.3211029	2.0249799	0.3704197	0.7984403
Pex19	0.1274055	5.2099664	0.3705116	0.7984403
Pafah2	-0.1645933	3.7758651	0.3705784	0.7984403
Fam71e1	0.5587881	-0.6489389	0.3706073	0.7984403
Lrrc75a	0.2551905	1.9136837	0.3706538	0.7984403
Uqcr11	0.1432365	6.4390596	0.3707117	0.7984403
Wee1	0.1242505	5.4282883	0.3707138	0.7984403

Syce2	0.1393405	5.0109767	0.3708526	0.7984403
Txndc17	-0.1357032	5.6015181	0.3708829	0.7984403
Rab39	-0.2797328	1.133605	0.3709457	0.7984403
Col23a1	0.3078266	0.6304815	0.3709894	0.7984403
Ap1s3	0.218108	3.7809054	0.3710512	0.7984403
Sf3a1	0.1122774	6.5725501	0.37109	0.7984403
LOC115488151	-0.3895457	0.1782968	0.3711251	0.7984403
Vcpip1	-0.095653	6.6136474	0.3711493	0.7984403
Gm36279	-0.2547728	1.4518388	0.3712402	0.7984403
Gpr171	0.249059	3.4362234	0.3712847	0.7984403
Pcdhga6	-0.479628	-0.3495235	0.3712863	0.7984403
Rbsn	0.130657	4.3406936	0.3712985	0.7984403
Lypla1	-0.1441753	5.794948	0.3713461	0.7984403
Slc6a1	-0.5882156	-0.447156	0.3714077	0.7984624
Cavin1	-0.1133782	5.7738447	0.3714758	0.7984985
Bcar3	-0.1950344	4.3354146	0.3715448	0.7985356
Nlrp6	-0.3415216	0.9716295	0.3715958	0.7985356
Ralgapb	-0.1043531	6.3947546	0.3720671	0.799438
Gm41248	0.671744	-0.7465966	0.3721989	0.7995074
Icam2	0.1786307	3.8906281	0.3722022	0.7995074
Tdrp	0.3837768	1.970879	0.3722873	0.7995217
Gm7729	0.3388657	0.6344333	0.3723117	0.7995217
Gm36809	0.572184	0.1636203	0.3724716	0.7997103
Rundc1	0.1320059	3.7623436	0.3725514	0.7997103
Olfml1	0.4158221	0.484571	0.3725537	0.7997103
Armc1	0.1020756	5.9782547	0.3726136	0.7997284
Gan	-0.2699501	2.3225243	0.3727718	0.7999576
Ms4a6c	-0.1250422	6.6131862	0.3728303	0.7999728
Cep72	0.1517771	3.6288512	0.3729795	0.8001452
Trmt2a	0.1127273	5.4377082	0.3731042	0.8001452
Fcrl5	0.8130557	-1.263193	0.3731474	0.8001452
Gm266	-0.3988168	2.3013623	0.3732093	0.8001452
Trp53inp1	-0.1255973	7.4144041	0.3732419	0.8001452
Terf2ip	0.1331686	4.3005733	0.3732715	0.8001452
Leng8	0.1271139	7.3166176	0.3733056	0.8001452
Gm45918	-0.6940776	-0.9370475	0.3733222	0.8001452
Man2c1	0.1254348	4.5570806	0.373382	0.800163
Zfp451	-0.1165697	4.8696335	0.3735404	0.8003923
Myo1e	0.2059124	5.4898626	0.3736098	0.8004307
Epsti1	0.2899769	3.9001755	0.3737139	0.8004769
Gemin2	0.1545335	3.6254448	0.3737343	0.8004769
Slfn14	0.1867428	6.6371352	0.3737899	0.8004783
AI413582	-0.1702817	3.4384531	0.3738379	0.8004783

Gm11942	0.8318056	4.1384077	0.3739411	0.8005891
Ttll4	0.1173475	4.9692139	0.3740334	0.8006765
Emilin2	-0.1156918	7.1887663	0.37423	0.8008276
Zfp120	-0.1358623	4.760059	0.3742825	0.8008276
C1qa	0.1544543	6.7208731	0.3742942	0.8008276
Trpv2	0.1202692	5.1235459	0.3743677	0.8008276
Vps45	0.1447413	3.8102677	0.3743685	0.8008276
Pipox	0.6284827	0.3189948	0.3744382	0.8008276
Gypa	0.219034	9.7077748	0.3744644	0.8008276
Celf2	-0.1508026	7.3081151	0.3745747	0.8009534
Wnk3	-0.580354	-0.8798321	0.3746392	0.8009812
Pde4c	0.7352943	-0.3878216	0.3747216	0.8010472
Tcirg1	0.1186225	6.7130333	0.3748122	0.801051
Eml1	0.300674	2.8654854	0.3748784	0.801051
Utp4	0.1402576	4.5516386	0.3749205	0.801051
Sptbn2	0.3877897	0.1186407	0.3749294	0.801051
Mexis	-0.3088137	0.9930416	0.375021	0.8011368
Fam110a	0.18299	3.4978916	0.3751936	0.8011942
Ahsp	0.2391897	10.328334	0.3752847	0.8011942
Cwf19l1	0.1420488	3.9542987	0.3753651	0.8011942
Gorasp2	0.102147	6.2915023	0.3754401	0.8011942
Gm5914	-0.237946	1.1802297	0.3754706	0.8011942
Nnmt	0.3781915	0.7155398	0.3754831	0.8011942
Aldh1a1	0.1937687	7.0232505	0.3754874	0.8011942
Adarb1	-0.2021206	3.8649462	0.3755453	0.8011942
Eif2s1	0.1261521	6.8404748	0.3755887	0.8011942
Sap18b	0.2568008	1.5006347	0.3756783	0.8011942
Eif4e	0.0993285	6.9652985	0.375705	0.8011942
Arl10	-0.1346704	4.129895	0.3757423	0.8011942
Smyd2	0.1337562	3.9557494	0.3758189	0.8011942
St13	0.0991805	6.8637916	0.3758279	0.8011942
Afap1	-0.1768703	4.3748741	0.3759275	0.8011942
Iba57	0.1542727	4.9546889	0.3760017	0.8011942
Fam20b	0.0969618	6.341864	0.3760912	0.8011942
Rnf5	0.1075289	5.2082569	0.3761071	0.8011942
Thumpd2	0.2251826	2.8438483	0.3761444	0.8011942
Hey2	0.5283359	-0.0574783	0.3761851	0.8011942
Rmdn2	-0.1941124	2.3741679	0.3762029	0.8011942
Gprasp1	0.1586909	4.1102683	0.3762471	0.8011942
Caprin2	0.1709185	2.9058224	0.3762554	0.8011942
F930017D23Rik	0.2825441	3.3621504	0.376307	0.8011942
9830132P13Rik	0.2858576	1.7570067	0.3763357	0.8011942
Gm16104	-0.4642898	0.1523911	0.3763907	0.8012017

Lias	0.1445689	4.7966258	0.3765486	0.8012453
Igkv4-90	-0.444559	1.7536716	0.3765635	0.8012453
D2hgdh	-0.1572914	3.5608012	0.3765657	0.8012453
Gm31828	0.3095638	1.9723306	0.3767617	0.8013988
Gba2	0.1041475	5.4085141	0.3768215	0.8013988
Cyrib	-0.1302247	7.6599474	0.3768366	0.8013988
Mertk	-0.1404412	4.5832272	0.3768832	0.8013988
BC029722	0.1566391	3.825628	0.3768991	0.8013988
Grpel1	-0.1126031	5.2953911	0.376947	0.8013988
Nmi	-0.1388035	4.0573064	0.3770016	0.8014054
Tmem245	-0.1464174	5.3051192	0.3771742	0.8016628
Atf3	-0.3658681	0.8306401	0.3774444	0.8019092
Paox	0.1932529	3.0208384	0.3774564	0.8019092
Gm11963	-0.4219645	-0.5723268	0.3775193	0.8019092
Tmod1	0.1952335	6.1647748	0.3777057	0.8019092
Camta1	-0.1568733	4.4269781	0.3777832	0.8019092
Zfp467	0.2177218	3.1136942	0.3778142	0.8019092
Tmem241	0.2439765	2.6495433	0.3778676	0.8019092
Rasal3	0.1374404	4.8212453	0.3779263	0.8019092
Per2	0.1996081	3.8800476	0.3780197	0.8019092
Gpm6a	-0.1255237	4.0897137	0.3780212	0.8019092
4931414P19Rik	0.1985488	2.7111779	0.3780578	0.8019092
Nrros	-0.1161892	6.3812269	0.378104	0.8019092
Exosc9	0.167616	4.9968727	0.378122	0.8019092
LOC118568621	0.3568106	1.5132469	0.3783131	0.8019092
2310039H08Rik	0.22273	2.7376187	0.3783217	0.8019092
Acin1	0.1122694	8.1085786	0.3783719	0.8019092
Zkscan1	-0.1012784	5.1306756	0.3783829	0.8019092
Lmln	0.2121763	2.456997	0.3784542	0.8019092
Tprn	0.1623123	3.3167487	0.378538	0.8019092
Pcdhb17	-0.3494835	0.86251	0.3785636	0.8019092
Sh3bp5l	0.1169944	4.8465977	0.3785714	0.8019092
Cnot1	-0.1461483	7.8941111	0.3786145	0.8019092
2810001G20Rik	-0.239613	2.8361368	0.3786413	0.8019092
Ppp2r1b	0.1337054	6.5259262	0.3786585	0.8019092
Dync1li1	-0.1177062	5.4864745	0.3786631	0.8019092
Nup35	-0.1412026	4.3461023	0.3787013	0.8019092
Nectin2	0.2254809	2.7873835	0.378711	0.8019092
Mfge8	0.1433994	4.6739861	0.378739	0.8019092
Sem1	-0.1354443	7.7673379	0.3787882	0.8019092
Arhgap24	0.1912179	4.0653197	0.3788534	0.8019092
Alox15	-0.8118208	5.3105051	0.3788884	0.8019092
Higd2a	0.1135006	5.0761769	0.3789795	0.8019929

Eng	0.1041198	5.9557366	0.3790509	0.8020044
Adal	0.1357808	4.0984097	0.3790881	0.8020044
Nfx1	0.0991088	6.2135352	0.3794052	0.8021195
Klrc3	-0.3414832	0.7286299	0.3794461	0.8021195
Rbms2	0.1354853	4.5303429	0.3795009	0.8021195
Asap2	-0.1763092	4.8793484	0.3795552	0.8021195
Slc9a6	-0.1176734	4.6547539	0.3795638	0.8021195
Tars	0.1107698	5.9712849	0.3797514	0.8021195
Ndufaf6	0.2666751	1.8180978	0.379768	0.8021195
Usp35	0.2885318	2.0027391	0.3798005	0.8021195
Chrac1	-0.142462	4.485882	0.3798018	0.8021195
Arhgef17	-0.2029477	3.8352645	0.379811	0.8021195
Creg2	-0.414959	-0.2500707	0.3798217	0.8021195
Arhgap35	-0.1499592	4.5506081	0.3798709	0.8021195
Dclre1b	-0.1194092	5.6297773	0.3798738	0.8021195
Zfp384	0.0985873	5.8322624	0.3799087	0.8021195
Ralgps2	0.1167615	5.6170551	0.3799377	0.8021195
Gatad2b	-0.1142145	6.3738807	0.3800481	0.8021195
Mdm2	-0.0932593	6.3367277	0.3800577	0.8021195
Mief2	-0.2113404	2.6952713	0.3800929	0.8021195
Ncor2	-0.1243152	6.1410278	0.3802363	0.8021195
Tmem80	0.1529638	3.239227	0.3803142	0.8021195
Ddx24	0.130664	6.1079389	0.3803398	0.8021195
Atp6v0e	-0.149182	6.8455703	0.3803651	0.8021195
Csrnp3	-0.4308027	2.0586704	0.3803758	0.8021195
Clk2	0.1972028	3.3982446	0.3803802	0.8021195
A630026N12Rik	-0.169805	3.3821276	0.3805459	0.8023145
Utp15	0.129254	4.7740398	0.3805758	0.8023145
Esrra	0.1403447	4.5154898	0.3807028	0.8024735
Gm19765	0.4753918	-0.446764	0.3809755	0.8026957
Brms1	0.1203026	4.9544086	0.3809802	0.8026957
Faim	0.1503038	3.7058841	0.3811385	0.8026957
Polr3h	0.1719906	4.4176794	0.3811477	0.8026957
Senp8	-0.1993268	2.7454516	0.3812118	0.8026957
Gm39173	0.3821485	0.7484724	0.3812663	0.8026957
Gm6257	0.4987205	-0.6463814	0.3813051	0.8026957
Mindy2	-0.1154075	7.0900101	0.3813265	0.8026957
Dohh	0.1721352	3.6447352	0.3813355	0.8026957
Mrgbp	0.1519294	4.1678536	0.3813395	0.8026957
6330549D23Rik	0.4520124	-0.8135965	0.381376	0.8026957
Rcsd1	-0.1420693	7.2723841	0.3815117	0.8027521
Pcdhgb7	-0.421079	-0.1128394	0.3815261	0.8027521
Gm20056	-0.2328875	3.9469415	0.3816038	0.8027521

Sf3b3	0.1249144	7.4049029	0.3816254	0.8027521
Cdyl	0.1277693	5.3389302	0.3816608	0.8027521
Akap17b	-0.1789458	2.9884036	0.3819291	0.8030364
Tm4sf1	-0.1973371	3.8750099	0.3819404	0.8030364
Ginm1	0.1225717	5.2925167	0.3819508	0.8030364
Unk	0.1131953	4.7945206	0.3820605	0.8030759
Dhx29	-0.1284312	4.8118169	0.3821034	0.8030759
Zfp36	0.1868477	6.086287	0.3821245	0.8030759
Coq8a	-0.1747344	3.6100388	0.3822772	0.8032882
Akna	-0.1526678	7.2579729	0.3824929	0.8036329
Slc39a4	-0.339524	1.1792921	0.3826363	0.8038256
Cog1	0.1093288	5.1237119	0.3827933	0.8040468
Exoc7	0.1077192	4.7377692	0.3829668	0.8043027
Cyb5rl	0.2959527	1.8035838	0.3831876	0.8045591
Fbxo38	-0.1003652	5.9477123	0.3832121	0.8045591
H2-T10	0.3041965	1.9530441	0.3832441	0.8045591
Ipo5	0.1098614	7.5988791	0.3834258	0.8046247
Gm13456	0.1491027	4.7253891	0.3834905	0.8046247
Cpsf6	0.1075532	7.5478598	0.3834916	0.8046247
Scai	-0.1348676	4.7262769	0.3834944	0.8046247
6720489N17Rik	-0.2155526	2.3243004	0.3835648	0.8046247
Def6	-0.1161753	5.9258383	0.383603	0.8046247
Sfr1	0.100362	6.6258167	0.38365	0.8046247
Kirrel2	-0.444161	-0.5941787	0.3837168	0.8046247
Rpl23	0.1323034	10.174975	0.3838402	0.8046247
5430416N02Rik	0.2526015	1.7451445	0.3838742	0.8046247
Gm6634	0.4065428	0.2664735	0.3839401	0.8046247
Trpm3	0.5984145	-0.9625233	0.3839405	0.8046247
H2-Oa	0.2226179	2.551744	0.3839857	0.8046247
Pdhb	0.0957069	6.4099138	0.3839996	0.8046247
Fundc1	-0.1344799	4.847488	0.3840843	0.8046854
Rbak	0.1654423	3.1536091	0.3841349	0.8046854
Slc25a14	-0.1996497	2.039129	0.3841837	0.8046854
Raf1	-0.0993668	6.3591805	0.384265	0.804734
Tmem214	0.1184456	5.426707	0.3843553	0.804734
Axl	-0.1795435	6.9477554	0.3843863	0.804734
Psmc3	0.1099832	7.4060897	0.3845087	0.804734
Trrap	-0.1459219	6.5165114	0.3845963	0.804734
Itgb1bp2	-0.3076453	1.5645766	0.3847501	0.804734
Pkdcc	0.2617929	3.2948176	0.3849	0.804734
A930006K02Rik	0.3193117	2.2414738	0.38491	0.804734
Rnf7	0.1094648	5.8606357	0.38492	0.804734
Gm10443	0.2293177	1.6853084	0.3849429	0.804734

Ttc23	-0.2625182	1.9049904	0.3850311	0.804734
Dlx6os1	0.4198526	0.3386505	0.3850432	0.804734
Gemin7	0.1292252	4.3761542	0.385064	0.804734
Sirpb1b	0.3574782	2.2415138	0.3850919	0.804734
Aptx	-0.1479766	3.6619978	0.3851354	0.804734
Ebf2	0.4383124	0.2306759	0.3851403	0.804734
Kit	-0.1174394	5.6372361	0.3851513	0.804734
Amer1	0.144513	3.6744735	0.3851942	0.804734
Gm16217	0.5426785	-0.6827104	0.385202	0.804734
Pogz	0.1227256	5.2246215	0.3852418	0.804734
Serpine2	-0.1935206	8.1882271	0.3853267	0.804734
Acp2	-0.1050798	5.3764204	0.3854035	0.804734
Diaph3	0.1480156	6.278858	0.3854686	0.804734
Sec31b	-0.3221232	0.4358444	0.3854953	0.804734
Gm52902	0.4304682	-0.7651805	0.3855004	0.804734
Cfap300	-0.4438174	-0.2374292	0.3856008	0.8047465
Poll	0.1689475	2.8858043	0.3857025	0.8047465
Mpv17	-0.1457791	3.5873682	0.3857163	0.8047465
Gimap5	-0.2196457	2.2198965	0.3857286	0.8047465
Asb17os	0.2925536	3.4803959	0.3857651	0.8047465
Scp2-ps2	-0.3096837	1.0839013	0.3858561	0.8048283
Zfp113	-0.1626888	3.4048822	0.3860512	0.8051274
Uvrag	0.1177534	5.3385551	0.386252	0.8053797
Gm16576	-0.2461847	2.5443643	0.3862757	0.8053797
Ncoa6	-0.1271663	6.3703753	0.3863784	0.8054858
Cd2	0.25771	3.6339834	0.3865048	0.8055948
Zc2hc1a	0.2832443	2.2635501	0.3865343	0.8055948
LOC118568032	0.1452193	5.1024825	0.3866771	0.8057845
Cops7a	0.1138918	5.1373401	0.3868322	0.8059912
Trappc11	-0.1184007	5.6143054	0.3868799	0.8059912
Col1a2	0.3175973	13.407939	0.3869625	0.8060552
Wwp2	0.1100329	6.5204817	0.3870822	0.8061357
St7l	-0.1636319	3.7105826	0.3871048	0.8061357
Cep112	-0.4250692	-0.208172	0.3872628	0.8061414
Cct5	0.1015726	7.8402236	0.3872704	0.8061414
Flrt2	-0.2789102	3.5226087	0.3872896	0.8061414
Prdm2	-0.129016	6.3577087	0.3873148	0.8061414
AA543186	0.464009	-1.0378414	0.3874266	0.8061846
Igkv4-70	0.7214166	1.8924693	0.3874841	0.8061846
Ccnl2	0.137834	7.5409788	0.3875597	0.8061846
Dhrs3	0.1197723	5.8642044	0.3875746	0.8061846
Erlin1	-0.1269956	5.5552946	0.3875947	0.8061846
2700081O15Rik	0.2048615	4.534861	0.3876768	0.8062475

Dusp14	0.3234226	1.1639967	0.3884002	0.807486
Arih2	0.0976802	5.927586	0.3884371	0.807486
Adrb1	-0.4655411	-0.7679806	0.3885532	0.807486
Nop14	0.1103739	5.213323	0.3885795	0.807486
Tes3-ps	-0.3159655	0.3524191	0.3887077	0.807486
Dnajc2	0.0920315	5.9906361	0.388791	0.807486
Pigg	-0.1526035	3.633915	0.3887922	0.807486
Col9a3	-0.6069829	3.912532	0.3887963	0.807486
Rtel1	0.1283084	4.7516112	0.3888306	0.807486
Cdkal1	0.1691501	3.3039318	0.3888761	0.807486
Rhot2	0.1152163	5.1866001	0.388985	0.807486
Gm7862	0.2909293	0.7793077	0.3889896	0.807486
Pdia6	-0.1049542	7.6023417	0.3891377	0.807486
Rcn2	0.1330916	4.5278737	0.3892826	0.807486
Rnpc3	0.1299465	5.4055749	0.3892848	0.807486
Fam91a1	-0.0956226	6.5903891	0.3893597	0.807486
Ddx51	0.1377167	4.3300202	0.3893728	0.807486
3000002C10Rik	0.3297127	0.6242393	0.3894255	0.807486
Npc1	-0.1173678	5.2658286	0.3895068	0.807486
LOC118567856	-0.4852224	-0.7207194	0.3895527	0.807486
Nme3	0.1542765	4.7668746	0.3895867	0.807486
Rpl34	0.1810743	8.474368	0.3896226	0.807486
Clasp1	0.1244401	6.0211652	0.3896326	0.807486
Nr2c2	-0.1066967	6.8738328	0.3897093	0.807486
4930563E22Rik	-0.4726958	-0.0278349	0.3897132	0.807486
Trim12a	-0.2366942	5.3686379	0.3897427	0.807486
Rpl13	0.1269587	9.8029674	0.3897488	0.807486
LOC118568753	0.4962131	-1.0033032	0.3897491	0.807486
Pdzd3	0.4313634	-0.2774528	0.3897779	0.807486
Zpbp	-0.37773	-0.216416	0.3900137	0.807696
Cdc37l1	-0.1016212	6.2887589	0.3900311	0.807696
Gm30233	0.2256586	2.5939766	0.390035	0.807696
Adk	-0.1109815	5.5627906	0.3900965	0.8077157
Tulp3	0.1855817	2.9625537	0.3901661	0.8077523
Copz2	0.2729161	4.0715271	0.3902668	0.8078534
1700113A16Rik	0.350967	0.1519436	0.3904656	0.8081402
LOC118567379	0.2870842	0.5640876	0.3905263	0.8081402
Gm10419	-0.2966305	1.6008274	0.3905613	0.8081402
4933407K13Rik	-0.2971971	0.7814881	0.3906873	0.8082934
Foxp3	0.5266014	-0.4310244	0.3908404	0.8085026
Acsm3	-0.5544827	-0.8594564	0.3909961	0.8087158
Procr	0.3245371	0.1083062	0.3910474	0.8087158
Rps27-ps2	0.4722759	-1.1204049	0.3911967	0.8089169

Dpm3	0.1445405	4.7058984	0.3913871	0.8092031
Hoxd8	0.4735405	1.0547759	0.3914823	0.8092924
Glo1	0.2446397	6.6438365	0.3915778	0.8093135
Cdc42bpa	-0.1072531	5.4186928	0.3916253	0.8093135
Tmem120b	0.1799655	4.5927555	0.3916486	0.8093135
Gm13192	-0.527586	-0.9465012	0.3917757	0.8094686
Pkhd1l1	0.1699491	6.753954	0.3918516	0.809487
Tmem263	0.1554999	5.3781022	0.3919085	0.809487
Twist1	0.3745046	3.0723332	0.3919832	0.809487
Chmp6	-0.1471971	3.9673854	0.3920321	0.809487
Gm4149	0.1786433	3.3597577	0.3920802	0.809487
Cenpi	0.1628864	4.8572456	0.3921106	0.809487
Zfp397	-0.118744	5.7870547	0.392153	0.809487
Carnmt1	-0.1623205	4.2489374	0.3923104	0.809487
Gm20658	-0.3021836	1.8859267	0.3924023	0.809487
Ncbp1	0.1019601	6.7152413	0.3924103	0.809487
Zfp287	0.3382941	1.3352904	0.3924143	0.809487
Zfp787	0.1719186	3.7834411	0.3924411	0.809487
Med14	-0.1222302	6.4531077	0.3924612	0.809487
Ppp3ca	-0.1110033	7.2200608	0.3928449	0.8101061
Ccne1	0.1579469	6.4848378	0.3928655	0.8101061
Sec63	0.1171446	6.8928822	0.392982	0.8102311
Mreg	-0.2351097	2.1829276	0.3930875	0.8102311
Egfl7	0.1465589	4.1372874	0.3930941	0.8102311
Gm31520	0.5119952	0.8527004	0.3932046	0.8102311
Tmem143	-0.1825387	2.8151873	0.3932686	0.8102311
Neurl1b	0.2361663	2.4509724	0.3933504	0.8102311
Cnot4	0.1139974	5.6027282	0.3933529	0.8102311
Zfp853	0.5604786	-0.8527949	0.3934081	0.8102311
Ttc14	-0.1046868	7.0794195	0.39346	0.8102311
Hagh	0.181068	7.0221553	0.3934604	0.8102311
BC147527	0.2777962	1.7553653	0.39352	0.8102311
Man1b1	-0.0901927	5.9750735	0.3935615	0.8102311
Ccdc25	0.101638	5.2164307	0.3936033	0.8102311
Epb41	0.1824185	9.4606181	0.393688	0.8102982
Ank1	0.1809625	8.8154615	0.3937779	0.810376
Gm3362	0.4564203	-0.7410369	0.3939733	0.8106653
Prtn3	-0.182523	8.9746956	0.3940228	0.8106653
Ripor1	0.113889	5.7594178	0.394165	0.8107592
LOC118567909	-0.5526158	-0.5705661	0.3941726	0.8107592
Tmem242	0.1427287	5.0730604	0.3943975	0.8108148
Gm46162	-0.1853129	3.3666933	0.3944696	0.8108148
Gabbr1	-0.2441992	3.9940222	0.3945474	0.8108148

Clip4	-0.4252925	0.1735174	0.3945542	0.8108148
Pars2	0.2415072	2.0055382	0.3945681	0.8108148
Cdkn2aip	-0.0989612	5.0553056	0.3945901	0.8108148
Cyb5r4	-0.2142858	6.5160745	0.3945951	0.8108148
Phrf1	0.1061121	6.0177327	0.3946167	0.8108148
Mtbp	0.1182388	4.4708496	0.3948088	0.8111024
Top3b	0.1156849	5.4928502	0.3950135	0.8113625
Lppos	0.4273329	0.6005837	0.3950684	0.8113625
Ppp1r11	-0.1206249	4.7983773	0.3950919	0.8113625
Tmppe	-0.1449569	4.2412902	0.3953177	0.8116591
Scn2a	-0.4277299	0.0952011	0.3953407	0.8116591
Ccdc71	0.1361055	4.6367317	0.3954335	0.8117268
Ankhd1	-0.1224738	6.8134888	0.3955121	0.8117268
Lin54	-0.0959342	5.5162313	0.3955302	0.8117268
Dapk2	0.1675932	5.7908379	0.3957188	0.8118842
Tbc1d22b	-0.1288544	4.1127121	0.3958008	0.8118842
N4bp2	-0.1621747	6.1833853	0.3958085	0.8118842
Vps35l	-0.1056302	5.5172992	0.3958914	0.8118842
Alkbh2	0.3029351	1.5451173	0.3959125	0.8118842
B230217O12Rik	-0.351874	0.1099908	0.3959271	0.8118842
Gng2	-0.1365428	5.6351799	0.3960976	0.8118842
Btf3-ps1	0.217993	1.7899616	0.3961269	0.8118842
Rab2b	0.1925267	3.3380918	0.3961962	0.8118842
Cd3e	-0.2810828	1.6388943	0.3962004	0.8118842
Atp5b	0.1045601	9.4816217	0.3962282	0.8118842
Dll4	0.2325281	1.5627128	0.3963024	0.8118842
Chic2	-0.1077458	5.3640818	0.3963189	0.8118842
Lpar6	0.1370726	4.7549627	0.3963377	0.8118842
Tmtc4	0.1949604	2.3197254	0.3967345	0.81259
Gm9512	0.449889	-0.9507819	0.3969916	0.8127747
Gm34305	0.5203679	-0.2251681	0.3970302	0.8127747
Ppp1r3f	0.2944663	0.7455157	0.3970309	0.8127747
Zc3h3	0.1512141	3.3141741	0.3970396	0.8127747
Cwc27	0.1177231	4.3483659	0.397086	0.8127747
Nr2c1	0.1690407	3.2797597	0.397255	0.8128401
Cachd1	0.2346614	3.2311997	0.3973047	0.8128401
Tpi1	-0.0873576	7.2000355	0.3973205	0.8128401
Fv1	0.2114246	2.3179424	0.3973743	0.8128401
Ccdc92	-0.2714957	2.2567226	0.3974272	0.8128401
Psmd11	0.0936304	6.6602768	0.3974315	0.8128401
Prkcd	-0.1261737	7.3603162	0.3977436	0.8133282
Fam118b	0.1477189	3.9196423	0.3977747	0.8133282
A430018G15Rik	-0.4246834	0.2618782	0.3978911	0.8133683

Ndufaf3	0.1298986	3.6696862	0.3978989	0.8133683
Sfn	0.2461999	2.6385607	0.398075	0.8134544
Paqr9	0.1636977	7.1912954	0.3981593	0.8134544
Slc35a2	0.1577443	3.9811128	0.3981668	0.8134544
Nav3	-0.2819746	2.7766231	0.3982353	0.8134544
Snx6	-0.1029387	5.8434098	0.3982463	0.8134544
Dtnbp1	-0.1082603	5.0940673	0.3982579	0.8134544
Gtf3c3	0.1319077	4.3197949	0.3983071	0.8134544
Gm31532	0.4547201	-0.6329793	0.3983762	0.8134778
Nudt14	0.185774	2.4654007	0.3984544	0.8134778
Mapkapk2	-0.1385919	7.4825007	0.3984755	0.8134778
Usf2	0.0910512	6.5877277	0.3986006	0.8135278
Tmem237	-0.1897053	2.5123179	0.3986569	0.8135278
Elmo2	-0.1483789	5.4147291	0.3987092	0.8135278
Rrbp1	-0.1205949	7.9393956	0.3987268	0.8135278
Pbk	0.1032358	6.3483445	0.3988032	0.8135278
Slc6a15	0.7391177	-0.3028569	0.398841	0.8135278
Timm10b	0.1140004	5.2984836	0.3988943	0.8135278
Gm16740	0.345494	0.3461112	0.3989394	0.8135278
Mvd	-0.2069707	2.5976094	0.3989707	0.8135278
Cox20	0.1506021	5.7156933	0.399092	0.8135812
Dimt1	0.1664762	3.2638912	0.399127	0.8135812
Dleu2	0.1940611	3.9705808	0.3991568	0.8135812
Dpm1	0.2721485	1.1675435	0.3992674	0.8135812
Prdx2-ps1	0.3030627	0.5273818	0.3992892	0.8135812
Ftl1-ps1	-0.2674485	3.2939308	0.3993679	0.8135812
Sh3rf3	0.4110637	0.0507814	0.3993702	0.8135812
Tcp11l2	-0.0956091	6.5051341	0.3994432	0.8135812
Ptprk	-0.2191625	3.6629856	0.3994676	0.8135812
Rps13-ps1	0.3250756	-0.0838024	0.3995447	0.8136092
Shkbp1	-0.0969429	5.0434543	0.399586	0.8136092
Ppfia2	-0.2846484	3.2414277	0.3997517	0.8136345
Wdyhv1	0.1496642	4.2335375	0.3997955	0.8136345
Zfp341	0.2205354	2.5906166	0.3998178	0.8136345
Nutf2-ps2	0.5374837	-0.0377042	0.3998838	0.8136345
Hddc2	0.2227121	2.9874445	0.3999636	0.8136345
Tasor	-0.0990602	6.2978311	0.3999671	0.8136345
Spns1	0.1280241	3.824127	0.4000954	0.8136345
Mtcp1	0.1983458	2.5654972	0.4001331	0.8136345
Otud4	-0.0979316	6.9164057	0.4001747	0.8136345
Ccdc28b	-0.1851618	3.6889463	0.4002606	0.8136345
Zadh2	0.1138839	5.2398826	0.4002725	0.8136345
Phb	0.1219749	5.6242431	0.40033	0.8136345

Oas1c	0.2638437	2.0560909	0.400373	0.8136345
Aamdc	0.2512079	2.8679459	0.4003739	0.8136345
Ube2h	0.097302	7.2485966	0.4003831	0.8136345
Zfp953	-0.1263581	3.8656086	0.4006815	0.8138074
Slc46a3	0.1500925	4.2508047	0.4007626	0.8138074
Klhl4	-0.3983131	0.0848036	0.4008183	0.8138074
Gm20605	-0.2101917	3.0655472	0.4008503	0.8138074
Pum2	-0.1020448	8.2217282	0.4008867	0.8138074
Gm6685	-0.4311605	-0.6932995	0.4009412	0.8138074
Arhgef19	0.3446834	1.7097257	0.4009836	0.8138074
Wwc2	-0.1397	4.6600394	0.4009991	0.8138074
Gm17709	0.5237857	0.5432909	0.4010772	0.8138074
Igkv4-61	-0.495478	2.3558624	0.4011097	0.8138074
Filip1	-0.2258803	1.2514439	0.4011372	0.8138074
Ubb	0.1118657	9.3993069	0.4011477	0.8138074
Pagr1a	0.1090407	5.2494124	0.4011483	0.8138074
Armcx3	-0.1278396	4.7776989	0.4012935	0.8139957
Tmod3	-0.0942767	7.0540142	0.401444	0.8141335
Ndufc2	0.1597573	6.2838135	0.4014942	0.8141335
Sgms2	-0.2041543	8.0252396	0.4015185	0.8141335
Gm10039	-0.6375493	4.0582762	0.4016933	0.8143819
Hemgn	0.1838214	9.2800258	0.401764	0.814412
Ngrn	0.1091131	4.6767298	0.4018129	0.814412
Sh3rf1	0.2171759	3.2639732	0.4020006	0.8145515
Pdxdc1	-0.0956285	5.823235	0.4020333	0.8145515
Gpatch11	0.1410682	4.343346	0.4020892	0.8145515
Hhex	-0.1228316	4.9937457	0.4020912	0.8145515
Coro7	-0.1572235	6.2190689	0.4021928	0.8145537
H1f0	-0.1501236	6.1748965	0.402197	0.8145537
Igk	-0.138274	8.2959378	0.4023333	0.8146504
Fancb	-0.1752617	3.120272	0.4023495	0.8146504
Gmfg	-0.14945	7.4759015	0.4024382	0.814724
Gm31326	-0.5176352	-0.7439536	0.4025992	0.8147562
Mrpl18	0.1188772	6.6712779	0.4026222	0.8147562
Ralgapa2	-0.1421574	5.0302665	0.4026563	0.8147562
2210408F21Rik	-0.4177987	-0.1996169	0.4026637	0.8147562
Dr1	0.1016302	6.5716138	0.4027529	0.8148229
Dnal1	0.3072008	1.6342097	0.4028158	0.8148229
Bag4	0.1195415	4.3963964	0.4028538	0.8148229
Erp44	-0.0888502	6.178622	0.4031183	0.8150928
Wipi2	0.1166223	6.618144	0.4032607	0.8150928
Ly6e	0.1159207	8.6753193	0.4032871	0.8150928
Cdc42	-0.1003211	9.0376981	0.403297	0.8150928

Lrmp	0.1432858	7.787627	0.4033431	0.8150928
Cd59a	0.1903577	5.2796857	0.4033612	0.8150928
Gstt3	0.26256	1.6542949	0.4034432	0.8150928
Rsad2	0.2814327	7.9249514	0.403454	0.8150928
Ranbp2	-0.1139333	8.3296301	0.4034589	0.8150928
Casp2	0.104997	6.4074448	0.4035426	0.815109
Nadk2	0.1443276	7.0065538	0.4035717	0.815109
Dph7	0.1785793	3.134395	0.4036583	0.8151779
Esr1	0.1930255	3.4927562	0.4037983	0.8153549
Nxph3	0.5370252	-0.8295917	0.4039871	0.8156302
Riok1	0.1197278	5.3947564	0.4040968	0.8156537
Tmc4	-0.2567346	2.3584441	0.4041492	0.8156537
Zdhhc3	-0.1107124	6.627002	0.4041598	0.8156537
Nt5c2	-0.1167695	6.4860845	0.4042085	0.8156537
Tagln2	-0.1257852	7.828234	0.4043971	0.8159285
Scml2	-0.2306869	2.0585229	0.4045039	0.8160183
Ctdnep1	0.104619	5.7755729	0.4045465	0.8160183
Dtx2	0.2426741	3.6851327	0.4047222	0.8162668
Rtn4rl1	0.2036601	3.0212021	0.4048468	0.8163252
Creb3l3	-0.3541004	1.3716764	0.4049233	0.8163252
3300005D01Rik	0.5329098	-0.5893386	0.4049574	0.8163252
Nkrf	0.1354656	4.045707	0.4050003	0.8163252
Slc35c2	-0.1125828	5.3572804	0.4050532	0.8163252
Rabl6	0.094142	5.7276521	0.4050661	0.8163252
Gm15459	0.1414936	8.7080913	0.4052456	0.8164334
Mtg2	0.1601532	2.8605216	0.4052843	0.8164334
Scrib	0.0955226	5.7176	0.4055406	0.8164334
Gm10177	0.2860919	1.259163	0.4055418	0.8164334
Mdfic	-0.1321606	4.7345928	0.4056235	0.8164334
Slc39a10	-0.1055192	4.8413522	0.4056378	0.8164334
Fgf1	-0.4691375	2.9610781	0.4056582	0.8164334
Fancc	0.1553906	3.6735426	0.4056646	0.8164334
Gm41291	-0.2860644	0.9585244	0.4056659	0.8164334
Ube2d3	-0.0929369	8.4878578	0.4056916	0.8164334
Trim25	-0.1014006	6.410128	0.4057108	0.8164334
Tmem135	0.1170673	4.7581779	0.4057497	0.8164334
Gm34181	-0.3951427	0.2099659	0.4059081	0.8165698
Gm12960	-0.3880599	-1.0437049	0.4060065	0.8165698
Mamdc4	0.4475071	-0.2036248	0.4060335	0.8165698
Usp16	-0.0953599	5.5579918	0.4060874	0.8165698
Epb41l3	-0.2061403	4.0877379	0.4061013	0.8165698
Dcps	0.1217488	5.0424244	0.4061767	0.8165698
Abhd14b	-0.2019981	2.0792	0.4061849	0.8165698

Clptm1	0.0923711	6.3459412	0.4063668	0.8167141
Anln	-0.1206419	7.0290098	0.4064345	0.8167141
Zfp825	0.1506989	3.425633	0.4064419	0.8167141
Plekhm3	-0.1765529	4.5224815	0.4064667	0.8167141
Prkra	0.1650802	4.3418821	0.406561	0.8167606
Cdc42se2	-0.093137	6.0777959	0.4065949	0.8167606
Psmd12	0.0980016	6.4838019	0.4067787	0.8168997
Kdm8	0.1672174	2.7091473	0.4067907	0.8168997
Osbpl6	0.3821862	0.9390888	0.4068217	0.8168997
Frs3	-0.4860684	-0.5099477	0.4070263	0.817205
Pcyt1b	0.1447686	5.0541611	0.4071241	0.8172185
Tnks1bp1	-0.1454988	4.7903605	0.4071381	0.8172185
Gm19696	-0.3529707	0.3695243	0.4072337	0.8173049
2810454H06Rik	0.4028412	0.7615026	0.4073463	0.817389
Luc7l	0.1072585	6.1457661	0.4075063	0.817389
Tsr1	0.1209954	5.3033078	0.4076286	0.817389
LOC118568031	0.3537398	0.2579274	0.4076667	0.817389
Cope	0.0942463	6.1655483	0.4077242	0.817389
Ipo11	-0.1377374	4.4966892	0.4077326	0.817389
Ccdc71l	0.1332322	6.5043646	0.4077552	0.817389
Snta1	0.2395608	2.5662378	0.4077818	0.817389
Amotl1	-0.1874714	5.0526481	0.4077948	0.817389
Mnd1	0.1891667	3.0523938	0.407847	0.817389
Lcor	-0.100374	5.1230201	0.4078537	0.817389
Pigo	-0.1399774	4.002878	0.4079531	0.8174829
Gkap1	-0.1754248	2.7102499	0.4081566	0.8176247
Ninl	0.172595	4.3020146	0.408202	0.8176247
Yipf4	0.1393347	6.5241072	0.4082315	0.8176247
Gm30732	0.2376338	1.0548012	0.4082341	0.8176247
Trappc13	-0.1273712	4.6883847	0.4083537	0.8177588
Carmil3	0.4865543	-0.990168	0.4084642	0.8178058
Atp5a1	0.0965048	9.1340675	0.4084966	0.8178058
Atraid	0.1239497	4.6758775	0.4085905	0.8178058
Gm9794	0.1533258	4.3183789	0.4086331	0.8178058
Cryzl2	-0.1781331	3.3696085	0.40864	0.8178058
Tal1	0.1655674	8.1791219	0.4088182	0.8180572
Klhl33	0.5131081	1.7408355	0.4089446	0.8181268
Rom1	0.2737437	3.086728	0.4089655	0.8181268
Pard6g	0.2818376	4.1893782	0.4090169	0.8181268
Kcmf1	0.0867068	6.2605205	0.409094	0.8181268
Gm20498	-0.4497919	-0.6568571	0.409116	0.8181268
Il12rb2	-0.2807383	1.1265767	0.4092383	0.8181826
Dglucy	0.1840896	3.2939167	0.4092491	0.8181826

Twistnb	0.0965547	5.2227596	0.4094603	0.8183068
Gm41644	0.4013438	-0.8666884	0.4094699	0.8183068
2010315B03Rik	0.1673679	3.2722659	0.4094845	0.8183068
Rbm4	-0.3153503	1.0496416	0.4095345	0.8183068
Lactb2	-0.1297096	4.3937167	0.4095811	0.8183068
Scoc	0.1565971	5.6338281	0.4097817	0.8183068
Zfp777	0.1462384	3.9269956	0.4097875	0.8183068
Pm20d1	-0.6676549	-0.6431352	0.4099826	0.8183068
Pdcd5	0.1560974	5.9735763	0.409985	0.8183068
Borcs7	0.152997	3.161991	0.4100502	0.8183068
Gmds	-0.1527122	3.2842488	0.4101284	0.8183068
Gba	-0.1048101	5.330212	0.4101374	0.8183068
Rps13-ps2	0.2404839	1.3195425	0.4101465	0.8183068
Azi2	-0.0932785	6.1906941	0.4101798	0.8183068
Nipsnap2	-0.105762	5.328584	0.4102188	0.8183068
Dctn3	0.1211511	5.1833057	0.4102565	0.8183068
Trp53i13	0.2437402	2.5192244	0.4103005	0.8183068
Zfp84	-0.127571	4.7623873	0.4103262	0.8183068
Gm14269	0.426142	-0.711743	0.4103561	0.8183068
Exoc3l2	-0.1245805	4.0493558	0.4103634	0.8183068
Acadvl	0.1163972	5.7204165	0.4104258	0.8183261
Tmc6	0.1056027	5.9784309	0.4104986	0.8183665
Ccser2	-0.1250068	5.2511728	0.41057	0.8183765
Pnpla3	0.8565248	-0.5703251	0.4106088	0.8183765
Gm5526	-0.2473147	2.2061083	0.4106925	0.8184384
Cyp2f2	-0.6141655	1.1363233	0.4108266	0.8186006
Tmem65	-0.1076207	5.6346522	0.4109786	0.8186942
Ola1	0.1052576	6.0284027	0.4109855	0.8186942
Rab3a	0.2322572	2.1670553	0.4110357	0.8186942
Sbf2	-0.1512713	4.3560155	0.411126	0.8186942
Usp3	-0.1065469	5.4862855	0.4112004	0.8186942
Spns2	-0.2046374	4.8326919	0.4112492	0.8186942
Rbm15b	0.1064001	6.1138226	0.4112993	0.8186942
Mecom	-0.299595	0.6596606	0.4113875	0.8186942
Phf3	-0.1036914	7.079191	0.4114383	0.8186942
Gm52704	0.3473673	0.6426304	0.4114717	0.8186942
Alpk1	-0.1828476	3.9910538	0.4115023	0.8186942
Dtwd1	0.1793742	3.7701024	0.4115584	0.8186942
Gpcpd1	0.1847632	7.3682471	0.4116382	0.8186942
Zwint	0.1112676	6.3934993	0.4116489	0.8186942
Nif3l1	0.1390319	3.7692507	0.4116631	0.8186942
Fcsk	-0.1666567	3.0008256	0.4118521	0.8188968
A230072C01Rik	0.2880578	1.7375126	0.4118702	0.8188968

Usp19	0.0926479	6.7537906	0.4119388	0.8189284
Akr1a1	0.0923659	7.9234432	0.4121092	0.8190367
Nedd4l	-0.1990157	4.1787514	0.4121749	0.8190367
Mob3c	0.1401716	4.147426	0.4121807	0.8190367
Ttc30b	-0.4140935	2.636677	0.4122039	0.8190367
Smim10l1	0.1259087	5.5603013	0.4122899	0.819103
Rpl9	0.120183	9.5034442	0.4123887	0.8191321
LOC115489981	0.3095859	2.0321187	0.4124099	0.8191321
Il31ra	-0.251739	1.3797337	0.4127111	0.8195437
Tm9sf2	-0.0889807	7.101322	0.4127336	0.8195437
Gna12	-0.1683751	4.915673	0.4128092	0.8195437
Acaca	-0.1103265	4.9187914	0.4128279	0.8195437
Zfp53	0.1819136	4.0132468	0.4130385	0.8195935
Gal	0.4348287	-0.8584389	0.4131745	0.8195935
Noct	0.156238	5.1289425	0.4133903	0.8195935
Gal3st1	0.5980373	-1.0726913	0.4134001	0.8195935
Jag1	0.1931602	3.4994362	0.413436	0.8195935
Zfp598	0.0928683	5.5410496	0.4134904	0.8195935
Ndrg3	0.0983537	5.1405769	0.4135759	0.8195935
Lrrc39	0.3373384	2.4054642	0.4135793	0.8195935
Gm18720	0.483035	-0.0384025	0.4136196	0.8195935
Deup1	-0.4882817	-1.0106697	0.4136435	0.8195935
Lfng	0.1110354	5.2669459	0.413664	0.8195935
Rpl36a-ps3	-0.3758061	-0.70733	0.413706	0.8195935
P2rx5	0.599213	-0.4811023	0.413718	0.8195935
Itgax	-0.2042509	3.2395882	0.4137277	0.8195935
Sfrp4	-0.1599024	4.6795734	0.4137783	0.8195935
Ccr3	-0.2178398	4.6618119	0.4137813	0.8195935
Lgi4	0.4475561	0.2228703	0.4137962	0.8195935
Ttf1	-0.1152476	4.7907979	0.4138014	0.8195935
Gng4	-0.500392	0.2797198	0.413921	0.8197128
Ecm1	0.1411275	4.8804257	0.4140751	0.8197128
Gm46967	0.3139592	0.1797274	0.4142774	0.8197128
Trmt2b	-0.1073908	4.7217336	0.4143107	0.8197128
Egfl8	0.261557	0.7214705	0.4143404	0.8197128
Haus1	0.1690787	4.5362145	0.4144283	0.8197128
Nipa1	0.1987949	4.3821692	0.4145329	0.8197128
Slc16a4	0.5491648	0.7026855	0.4145444	0.8197128
Dek	0.114562	9.6747155	0.4145491	0.8197128
Srsf11	0.0924906	7.5917234	0.4145664	0.8197128
Ptrh2	0.1433562	4.7960866	0.4145665	0.8197128
Gm15421	0.4604258	0.538161	0.4145678	0.8197128
Cyp26b1	-0.4193894	0.7012171	0.4145968	0.8197128

Hr	-0.2962922	1.3111215	0.4145995	0.8197128
Ihh	-0.6757048	0.749407	0.4149429	0.820207
Atp9a	0.2020676	1.9267244	0.4150408	0.820207
Pet100	-0.1613132	5.1642785	0.4151169	0.820207
Msh2	0.1107665	5.2950192	0.415225	0.820207
Tnfrsf11a	0.2067413	3.9734829	0.4153345	0.820207
Coq3	-0.1620301	3.10429	0.4153699	0.820207
Gm8251	-0.4339844	-0.0650954	0.4154105	0.820207
Tomm6os	-0.3458339	-0.0443572	0.4154121	0.820207
Rad54l2	-0.138291	4.8810535	0.4154584	0.820207
Ccl22	-0.5296014	-0.9188799	0.4154675	0.820207
Nol8	0.1057867	5.146689	0.4155148	0.820207
Fdxacb1	0.161256	2.848394	0.4155497	0.820207
Bop1	0.1268619	4.810639	0.4155909	0.820207
Ccdc97	0.1017807	5.4544951	0.4156251	0.820207
Gm7435	-0.4465	-0.2726807	0.4157132	0.820207
Rbm4b	0.1664462	3.4176416	0.4157373	0.820207
Asap1	-0.1231251	7.8467412	0.4158922	0.820207
Setd4	0.3062757	1.4629907	0.4158928	0.820207
Msrb3	-0.1852141	4.5325688	0.4159183	0.820207
Dnajc14	-0.081948	6.2510136	0.4159355	0.820207
Nsd1	-0.1002171	7.513843	0.4159803	0.820207
Rab11a	-0.0926938	6.6724599	0.4160096	0.820207
Dnajb5	0.1950312	2.3575953	0.4162014	0.8204811
Tpm1	-0.1492277	8.4201771	0.416389	0.8205338
Enpp6	-0.2843446	4.6112456	0.4164198	0.8205338
Evi5l	-0.1630339	3.4748483	0.4164271	0.8205338
Tk1	0.1416026	6.8860229	0.4164391	0.8205338
Phpt1	0.1192655	4.0666103	0.4165136	0.8205685
Clk3	0.1264173	6.7535481	0.4165622	0.8205685
Rnf34	-0.0942558	5.1661519	0.4166569	0.820626
Mfsd3	0.3271353	0.9449909	0.4166969	0.820626
Rbpms2	-0.1496296	5.0103019	0.4167536	0.8206337
Adam3	-0.4853008	-0.984907	0.4168511	0.8206442
Plce1	-0.2666076	1.6922023	0.4168644	0.8206442
Sec24c	0.0879254	6.7203522	0.4170322	0.8208265
Gnb1	0.0922805	7.9692583	0.4171427	0.8208265
Siva1	0.1579428	5.518231	0.4172065	0.8208265
Lims2	-0.3104387	0.069501	0.4172115	0.8208265
Mark2	-0.1223848	6.7923193	0.4172209	0.8208265
Napg	0.1074396	4.8977526	0.4173827	0.8209372
Irgm2	0.2403562	5.8246667	0.4173828	0.8209372
Sgo2a	-0.1303235	5.976043	0.4176175	0.8212951

Trmu	0.2028374	2.7185261	0.4177867	0.8214267
Nbea	-0.1890521	2.5946808	0.4177901	0.8214267
Ift46	-0.1386958	4.1415915	0.4179062	0.8214499
Wipf1	-0.1358114	7.3810384	0.4179075	0.8214499
Tram1	0.0931502	7.8675168	0.4180231	0.8215043
Tmem68	0.1148976	4.5915737	0.4180547	0.8215043
Trp53inp2	-0.117019	6.278202	0.4180936	0.8215043
Tfb2m	0.1288616	4.046932	0.4182226	0.8216539
Guf1	0.1090758	4.8552351	0.4183002	0.8217027
Mttp	-0.3046371	1.3714802	0.4185506	0.8219072
Phf12	0.0843641	6.4140911	0.4185544	0.8219072
Prkar2a	-0.1152822	5.0466904	0.4185902	0.8219072
Snhg20	0.252833	3.287902	0.4186357	0.8219072
Rnf40	0.0931345	6.5021969	0.4186685	0.8219072
Gdpgp1	-0.1956751	2.1497465	0.418831	0.8221223
Tcf7	0.2026493	3.5774709	0.4189377	0.822224
Eif3k	0.0953606	7.1489604	0.4189885	0.822224
Hap1	0.3799182	0.8944161	0.419449	0.822717
Zfp513	0.1241739	4.3148036	0.4194765	0.822717
Pemt	-0.3405118	0.9767875	0.4196651	0.822717
Mtrex	-0.1083107	6.632829	0.4197029	0.822717
Retreg3	0.0857348	6.0158625	0.4197162	0.822717
Proser1	0.123322	4.7260165	0.4197303	0.822717
Khdrbs1	0.1047468	7.2431588	0.4197575	0.822717
Gm2061	0.5717937	-0.5336483	0.4198082	0.822717
4930579G24Rik	0.1247744	4.2804508	0.4199292	0.822717
Cacnb2	-0.3211843	0.6218963	0.4199415	0.822717
Gm5134	-0.3543806	-0.1174593	0.4199803	0.822717
Glmn	0.1731543	2.732828	0.4200148	0.822717
Sepsecs	0.1222059	4.5680852	0.4200416	0.822717
E2f8	0.121091	7.705682	0.4200427	0.822717
Epyc	-0.675732	0.4308495	0.4201046	0.822717
Saysd1	0.2186645	1.9817956	0.4201392	0.822717
Mycl	-0.2937004	0.9494532	0.4201399	0.822717
U2af1	0.1036244	6.6173219	0.4201951	0.822717
Nt5dc3	0.1572123	3.342264	0.4202447	0.822717
Ticam2	-0.1764356	3.7836239	0.4203209	0.8227625
Zc3hc1	0.12368	3.9878593	0.4205039	0.8229482
Smpd1	0.1125512	4.767002	0.4205216	0.8229482
Paip2	0.0921401	7.5172138	0.4206542	0.8231041
Eaf1	0.0889714	6.3193463	0.4207649	0.8232172
Rassf2	-0.1213297	6.8766701	0.4208546	0.8232891
Pvt1	0.2568677	1.780693	0.4209479	0.8233682

Vps33b	0.1244144	3.9875302	0.421066	0.8233863
Ndufb7	0.1145025	6.9924473	0.421124	0.8233863
Gm38834	0.5560565	-0.4428529	0.4212423	0.8233863
Pcdhb4	-0.5273214	0.0280538	0.4212874	0.8233863
Zfp982	0.2105395	2.4935194	0.4212997	0.8233863
Dctn1	-0.1206378	6.8104151	0.4213274	0.8233863
Agmo	-0.2189168	1.7484305	0.4213277	0.8233863
Rdh13	0.1591953	3.0221696	0.4215392	0.8236435
Metrn	0.1460125	3.8030143	0.4215653	0.8236435
Mtrr	-0.1405193	3.5516634	0.4216461	0.8236868
Ddx19a	0.1019383	5.8372907	0.4217761	0.8236868
Bod1	0.1694533	3.3980548	0.4217938	0.8236868
Zfp280b	0.1249887	4.4187326	0.4217993	0.8236868
Ramp3	0.5459943	-0.3514841	0.421958	0.8238933
Nol4l	-0.143157	4.1713151	0.4220365	0.8239347
Rnf185	-0.1201851	4.3515214	0.4221218	0.8239347
Fyco1	-0.116537	4.9782183	0.4221381	0.8239347
C130046K22Rik	-0.2202157	1.9411634	0.4221955	0.8239434
Ric8a	-0.099877	5.0813862	0.4222621	0.82397
Nemp2	0.1441105	3.9221645	0.4223384	0.8240155
Slc25a43	0.3153812	0.1558113	0.4224176	0.8240666
Arid3b	-0.1358636	4.2436842	0.4224952	0.8241116
Naaa	0.1530892	4.9611616	0.4225466	0.8241116
Aamp	0.0848539	6.1925804	0.4226054	0.8241229
Gm8355	0.2036791	2.8542148	0.4228243	0.8242006
Lpcat3	0.1078935	6.6290842	0.4229107	0.8242006
Aldoa	-0.0908841	8.7373969	0.4229399	0.8242006
Fbl-ps2	0.296334	1.0533076	0.4229402	0.8242006
Gm6485	-0.3209106	-0.3014626	0.4229688	0.8242006
Sdhd	0.1274927	6.7248585	0.4232388	0.8242006
Fbxo9	0.1225684	6.4263554	0.4232547	0.8242006
Sp3os	0.2047351	2.7008447	0.423278	0.8242006
Slc26a7	0.2365172	2.5947531	0.423282	0.8242006
Ufsp1	0.1448825	3.8458634	0.4232843	0.8242006
Csnk2a1	0.0862622	7.0411621	0.423318	0.8242006
Hnrnph2	0.0886857	7.4575488	0.4233797	0.8242006
Eola1	-0.180683	2.9439675	0.4233799	0.8242006
Mettl26	0.1509979	3.2796808	0.4235485	0.8242006
Man2a2	0.1725795	6.1308539	0.4236736	0.8242006
Rps7	-0.1262485	9.6632425	0.4237031	0.8242006
Ebpl	0.2426014	2.6421791	0.4237928	0.8242006
Dclre1a	0.125619	4.531608	0.4238741	0.8242006
Smcr8	-0.1071318	5.9719445	0.4239076	0.8242006

2310034O05Rik	-0.4580884	-0.9039692	0.4239167	0.8242006
Vcpkmt	0.1651028	3.3118993	0.4239895	0.8242006
Parp10	0.1253633	4.3920605	0.4240228	0.8242006
Appbp2os	-0.5198257	-0.6608967	0.4241038	0.8242006
Rpusd3	0.2985724	0.5571373	0.4241217	0.8242006
Coa5	-0.0883423	7.0836931	0.4241332	0.8242006
Fcrl1	0.1861374	3.3840157	0.4243502	0.8242006
Arf3	-0.1128288	6.6748109	0.4244594	0.8242006
Gm10156	0.2794361	0.2226022	0.4244611	0.8242006
Fam216a	0.1233444	3.9411291	0.4244669	0.8242006
Gm13748	0.4192821	-0.0569345	0.4244725	0.8242006
4833417C18Rik	0.297032	1.1834999	0.4245121	0.8242006
Xab2	0.0862785	5.6683287	0.4245359	0.8242006
C2	-0.2744349	1.5707877	0.4246186	0.8242006
Rcl1	0.1482321	4.4132583	0.4246847	0.8242006
Tgif1	0.1707559	3.5704694	0.4246994	0.8242006
Mrpl54	0.1318422	4.3969069	0.4247248	0.8242006
Zfp14	0.2630517	1.5805107	0.4247398	0.8242006
Ercc8	0.2021285	2.7603027	0.4247969	0.8242006
Ptpn2	0.1003194	5.7305124	0.4247983	0.8242006
Polq	0.1214947	5.7944648	0.4248145	0.8242006
Gm46891	0.3313263	0.6274905	0.4248269	0.8242006
Igkv12-38	0.8973314	0.4622178	0.4248954	0.8242006
Fkbp4	0.0882088	7.1463831	0.4249238	0.8242006
Gm16973	0.2124219	3.1558336	0.4250422	0.8243276
Tbc1d9	0.14985	3.749901	0.425138	0.8244104
Zfp2	-0.3343734	0.8924659	0.4253661	0.8246315
Fbxl16	-0.4044406	0.6329086	0.4254688	0.8246315
Olfr558	0.3903443	-0.122466	0.4255333	0.8246315
Cadm3	-0.2017797	2.5032841	0.4256291	0.8246315
Pskh1	0.1157471	4.4611249	0.4256331	0.8246315
Nap1l4	0.1005078	7.2802438	0.4256915	0.8246315
Gm52380	0.4655175	-1.0321835	0.425713	0.8246315
Gm3375	-0.5212417	4.1830639	0.4257154	0.8246315
Myh10	0.1741656	6.6806061	0.4257291	0.8246315
Farsa	0.1150209	5.4883973	0.4258067	0.824679
Oaz1-ps	0.148649	3.2552127	0.4259175	0.8247909
Cobll1	0.2033143	3.11178	0.4261214	0.8250441
4933431E20Rik	0.1937754	2.2809098	0.4262105	0.8250441
Ccdc170	-0.3657748	1.4779731	0.4262438	0.8250441
Gm8013	0.4575654	-0.5688469	0.4264179	0.8250441
Ajm1	-0.3353515	-0.0319301	0.4264444	0.8250441
Gm34402	-0.3532456	0.6052059	0.4264473	0.8250441

Thumpd3	0.1237716	5.1881253	0.4264732	0.8250441
Gm5782	-0.332848	0.5566928	0.4264849	0.8250441
Samd3	-0.3510065	0.5296976	0.4265761	0.8250441
Ech1	-0.0923204	5.9438906	0.4265786	0.8250441
Zscan25	-0.1766694	3.5546686	0.4266383	0.8250568
Nfatc4	0.3575402	1.9408153	0.4268616	0.8253861
Umad1	-0.1191714	4.1665695	0.4269637	0.825425
Zfp944	0.109405	4.6852895	0.4269879	0.825425
Rpl15-ps6	-0.3399984	0.3769383	0.4270437	0.8254303
Rem2	-0.415832	-0.7744774	0.4271682	0.8255684
Coq4	0.1867056	2.8323418	0.4272362	0.8255973
Thap7	0.121022	3.8577172	0.4274367	0.8258297
Exd2	-0.1408479	3.431618	0.4274627	0.8258297
Herc3	-0.1262777	3.8364616	0.427594	0.8258846
Mboat1	-0.2284219	2.1604388	0.4275973	0.8258846
Mrps28	0.1345974	4.2741796	0.4277229	0.8260164
4833438C02Rik	-0.1623133	3.3998225	0.4278028	0.8260164
Igkv4-63	-0.449571	1.0035886	0.4278383	0.8260164
Il1bos	-0.4220112	-0.9613292	0.427949	0.8260164
N6amt1	-0.1863206	3.0903153	0.4279712	0.8260164
Lrrc14	0.1432998	3.8419956	0.4280458	0.8260164
Hacl1	-0.1878871	3.1519351	0.4281176	0.8260164
Gm3650	0.3034005	1.0173571	0.4282161	0.8260164
Psap	-0.1052203	10.106507	0.4282188	0.8260164
Rps15a-ps6	0.3132631	0.751752	0.4282918	0.8260164
Ucp3	-0.4478271	0.1271454	0.428321	0.8260164
Tmem63b	-0.1311937	3.9954086	0.4283817	0.8260164
Mcm3ap	-0.1107856	5.841344	0.4284943	0.8260164
Fjx1	0.3933398	0.066228	0.4285527	0.8260164
Sfxn2	0.2100906	2.5972254	0.4285574	0.8260164
Gm19135	0.4812738	-0.6529382	0.4286244	0.8260164
Tnfaip8l1	0.2190366	2.0002977	0.4286449	0.8260164
Cdk18	-0.2588452	2.2729099	0.4287023	0.8260164
Klrc1	-0.3851116	0.121184	0.4287677	0.8260164
Repin1	-0.1593485	3.1604823	0.4287806	0.8260164
Eif2ak3	-0.1293738	5.6185032	0.4287872	0.8260164
Ssna1	-0.0962939	5.2777088	0.4288339	0.8260164
Helz	-0.160705	5.7575945	0.4289527	0.826042
Tubg2	0.413017	-0.2395782	0.4289534	0.826042
Cpne1	-0.0903012	6.3984447	0.4291136	0.8260839
Cys1	0.4587132	0.120191	0.4291964	0.8260839
Ing3	0.105079	4.9612115	0.4292235	0.8260839
Smim3	-0.0990697	5.463346	0.4292368	0.8260839

Slc39a14	-0.2151177	4.2038319	0.4293911	0.8260839
AK157302	0.4102144	0.686862	0.4293933	0.8260839
Sh3bp5	-0.1114746	6.2635877	0.429407	0.8260839
Icosl	-0.1423838	4.2378859	0.4294655	0.8260839
Herpud2	-0.0867858	5.8928137	0.4296801	0.8260839
Cenpu	-0.144332	4.4410783	0.4298216	0.8260839
Cdc5l	0.0871334	6.9454058	0.4299385	0.8260839
Hectd1	-0.1078556	8.0068355	0.4301346	0.8260839
Urb1	0.1379312	4.0565917	0.4301983	0.8260839
Ikzf4	-0.2546036	0.4379866	0.4302241	0.8260839
Ubd	-0.170199	3.3942112	0.4302314	0.8260839
Asic3	0.6082227	-0.4319405	0.4302547	0.8260839
Zdhhc15	0.1627384	3.1655798	0.430296	0.8260839
Reck	-0.1319241	4.158159	0.4303587	0.8260839
Smg8	-0.1972402	4.4459438	0.4303997	0.8260839
Ezh2	0.1066589	7.3738709	0.430405	0.8260839
Btbd8	0.1890248	3.2188238	0.4304086	0.8260839
0610009L18Rik	-0.3297224	-0.3555003	0.4304092	0.8260839
Kantr	-0.1605259	3.1132688	0.4304204	0.8260839
Eipr1	0.119228	4.3121606	0.4304589	0.8260839
Dock9	-0.1533971	5.6200599	0.4305495	0.8260839
Srcap	0.1038187	6.8159223	0.430565	0.8260839
H2-Q4	0.1625338	6.1706937	0.4306085	0.8260839
Zfp945	-0.1465611	3.1980035	0.4306235	0.8260839
Lap3	-0.1143165	5.0811089	0.4306288	0.8260839
Tmbim1	0.1248829	4.4066027	0.4306552	0.8260839
Ahdc1	0.155851	4.0862971	0.4306552	0.8260839
Sash3	-0.1049678	6.4532763	0.430789	0.8260839
Rps12-ps4	0.2779346	1.0404895	0.4307919	0.8260839
Fcgr2b	-0.1152828	5.7022248	0.4308387	0.8260839
Ccdc85a	-0.5589023	-0.6789003	0.4309789	0.8260839
Stx6	0.1094821	4.4979131	0.4310525	0.8260839
Cldnd1	-0.1084864	5.0706054	0.4310917	0.8260839
Zfp532	-0.2155118	2.7600717	0.4311182	0.8260839
Ldhb-ps	0.5427387	-0.4716437	0.4311985	0.8260839
Uevld	-0.1332155	4.5305821	0.4311986	0.8260839
Pde7a	0.1455814	4.9774265	0.4312496	0.8260839
Madd	-0.1338355	5.7506123	0.4312667	0.8260839
2810403D21Rik	0.4774748	-0.1858565	0.4313232	0.8260839
Gm9726	-0.5769523	-0.6961738	0.4313237	0.8260839
Map1lc3a	0.1320826	3.852762	0.4313651	0.8260839
Ccr5	-0.2528111	2.3868984	0.4315183	0.826213
Atg12	-0.1046404	5.7954518	0.4315388	0.826213

Ppp3r1	-0.09499	7.0206441	0.4316488	0.8262752
Xrcc2	-0.1598473	3.4272153	0.4316857	0.8262752
Eef1akmt2	0.1569504	3.5188534	0.431791	0.8262752
Scfd1	-0.1225687	5.3382411	0.4318236	0.8262752
F730311O21Rik	-0.2077291	2.0662262	0.4318369	0.8262752
Xlr4c	-0.3239413	-0.2385849	0.4319809	0.8264491
Fmod	0.3426523	4.1992415	0.432069	0.826516
Kif7	0.2639393	1.9066677	0.4321346	0.8265398
Hmgb1-ps1	0.4827615	1.3405606	0.4323346	0.8267515
Gm15432	-0.4707878	-1.127619	0.4324021	0.8267515
Zfp459	-0.292985	0.4684277	0.4324292	0.8267515
Gm45871	-0.2314321	1.9332179	0.4324579	0.8267515
Zfand5	0.1092553	7.0657622	0.432713	0.8271375
Col15a1	0.2595578	4.4239336	0.4327912	0.8271853
Ctdsp1	-0.0960086	7.12774	0.4328958	0.8272775
Trmo	0.1807932	5.3846979	0.4329686	0.8272775
Cc2d1a	0.1346657	3.9524722	0.4330472	0.8272775
Hsf4	0.4064004	0.0857795	0.4330522	0.8272775
Rsl1d1	0.1076283	6.4895188	0.4331803	0.8273315
Dnajc19-ps	0.4247252	-0.7812009	0.4332151	0.8273315
Rhbdd3	0.1595194	2.9216142	0.43324	0.8273315
Pwwp3b	-0.3945757	0.7250889	0.4333516	0.8274429
Vangl2	0.2100248	3.3388779	0.4338004	0.8281146
Ece1	0.1707944	4.9679828	0.4338528	0.8281146
Mcmdc2	-0.4480903	-1.0226681	0.4338631	0.8281146
Zfp362	0.0960143	4.9514321	0.433929	0.8281388
Gm7879	0.3851658	0.3913906	0.4342891	0.8287245
Zfyve9	-0.1889771	3.1064845	0.4343506	0.8287401
Tnip2	-0.154224	3.4170397	0.4346273	0.8290263
CYTB	0.1374278	12.442338	0.4346413	0.8290263
Taf12	0.1070798	5.7035598	0.4346605	0.8290263
Dnaja1	-0.1102882	7.6010991	0.434857	0.8292252
E2f6	0.1066525	4.8251316	0.4349024	0.8292252
Epb42	0.1800265	8.0999739	0.4349247	0.8292252
Ints6l	-0.0945887	6.7995955	0.4350798	0.8293408
Polr2b	-0.1122909	6.8613576	0.4351743	0.8293408
Ccdc157	0.1707099	2.7486008	0.4351876	0.8293408
Adam23	0.3011767	0.4836771	0.435217	0.8293408
Ephx2	0.2241914	2.3194174	0.4353078	0.8293408
Rps9	0.1082838	9.507824	0.4353095	0.8293408
Nubp2	0.106471	5.1045907	0.4353969	0.8293408
Mia3	-0.0966496	6.6036372	0.4354119	0.8293408
Gm19705	-0.2200943	2.1596341	0.4354829	0.8293744

Mcm10	0.1104034	5.9486343	0.4355603	0.8294116
Yipf5	0.1021882	5.6194506	0.4356091	0.8294116
Gpr68	-0.26058	2.5386892	0.4358661	0.8297993
Ndufb4	0.1574065	5.3673236	0.4361048	0.8301357
Gm26637	-0.302605	1.4675283	0.4361722	0.8301357
Hnrnpll	-0.0887985	6.6355202	0.4362464	0.8301357
Adap2	0.1520649	4.2959209	0.4362894	0.8301357
Cep70	0.1287932	5.3709651	0.4363162	0.8301357
Nsfl1c	0.0878377	5.9304617	0.4364493	0.8301357
Pgpep1	0.1431362	3.7217491	0.4364684	0.8301357
Mesd	0.0902851	5.722286	0.4365491	0.8301357
Praf2	0.1909085	3.2190829	0.4365653	0.8301357
Dnajc8	0.0917725	7.2639263	0.4366208	0.8301357
Emp3	0.1058837	6.2189052	0.4366298	0.8301357
Gm7353	0.4956321	0.3524719	0.4367056	0.8301783
Plpp6	-0.1325938	3.5517961	0.4368316	0.8302062
Igf1	-0.1014046	5.7776094	0.4368385	0.8302062
Tmem67	-0.173827	2.133059	0.4368836	0.8302062
Srfbp1	0.1275111	3.8672711	0.4369338	0.8302062
Extl3	0.0928658	5.4348201	0.4370462	0.8303184
Atxn7l3	-0.1120358	6.2289476	0.4371258	0.8303681
H1f10	0.2117094	2.4726054	0.4372388	0.8304368
Rpl7	0.1292799	10.130043	0.4372687	0.8304368
Plppr2	0.3062204	0.7528965	0.4374243	0.8305841
Snx22	0.1710734	5.4401636	0.4374531	0.8305841
Podnl1	0.265768	2.5634567	0.4378768	0.8312871
Gm36738	-0.2949804	0.455139	0.4379571	0.8313381
Utp25	0.1296397	4.0199785	0.4380734	0.8314574
Ampd2	-0.1046146	5.5192291	0.4382118	0.8314785
Gbf1	-0.1024751	5.809087	0.4382315	0.8314785
Spg7	0.0899783	5.2825433	0.4382449	0.8314785
Rmnd5a	-0.0850107	7.3822519	0.4383413	0.8315599
Nup50	0.1065928	7.0340611	0.4384325	0.8316317
Sec16a	-0.0955491	5.9824463	0.4385349	0.8317244
Hoxa7	-0.2140949	1.4748947	0.4387006	0.8318105
Gng7	-0.3618929	0.422893	0.4387772	0.8318105
Utp23	0.1244751	4.1355571	0.4387807	0.8318105
Csnk1d	0.0834504	6.8476696	0.4387942	0.8318105
Arih1	-0.0926801	7.2975339	0.438884	0.8318133
Adck1	0.1551746	2.7739843	0.4390157	0.8318133
Tmem191c	-0.2498947	0.8504836	0.4391395	0.8318133
Arid4b	-0.0906962	6.0668186	0.4391953	0.8318133
Cluh	0.099146	5.6780433	0.4392222	0.8318133

Pot1b	0.1352977	4.4062332	0.439233	0.8318133
Gm20947	0.4427735	-0.4507833	0.4393117	0.8318133
Ufm1	0.0930629	5.4746648	0.4393414	0.8318133
Rbbp9	-0.1471883	3.2127284	0.4394377	0.8318133
Igkv4-55	-0.3826878	3.1782693	0.4394422	0.8318133
Gm15564	0.3642408	1.1381326	0.4394619	0.8318133
Gm4737	0.4064298	-0.101845	0.4395279	0.8318133
Exosc7	0.1171896	4.6911811	0.4395506	0.8318133
9930014A18Rik	0.4540511	-0.0219314	0.4396098	0.8318133
Gli1	0.5287711	-0.2226462	0.4397219	0.8318133
Pcdhb9	-0.2923058	1.9240705	0.439728	0.8318133
Fbxl8	0.1848799	2.334193	0.4397311	0.8318133
Cst3	-0.1655306	9.0427484	0.4397583	0.8318133
Gpr65	-0.1298541	4.7882725	0.4398332	0.8318539
Ppp4r3a	0.087662	7.1244778	0.43989	0.83186
Cox19	-0.1248812	3.9573529	0.4399618	0.8318948
Slc25a25	-0.1299873	3.8625548	0.4400582	0.8319758
Orc1	0.163381	5.1434526	0.4402134	0.8320593
Smchd1	-0.1160669	7.2160381	0.4402397	0.8320593
4930581F22Rik	-0.1618929	2.9269454	0.4403009	0.8320593
Brd3	-0.1019723	6.8021839	0.4403531	0.8320593
Rab23	0.173231	3.3841817	0.4404994	0.8320593
Pitx1	0.3211407	0.9503157	0.4406332	0.8320593
Pdik1l	-0.0913351	5.4616816	0.4407675	0.8320593
Ncoa4-ps	-0.2242448	2.1650824	0.4407785	0.8320593
Chmp4c	-0.6848366	-0.9083015	0.4407816	0.8320593
Pin1	0.0941533	4.8727385	0.4407843	0.8320593
Ckap5	-0.1161993	7.6779438	0.4408004	0.8320593
Tspo	-0.1056042	7.38041	0.44081	0.8320593
Acap1	-0.1072552	6.0364699	0.4408676	0.8320593
Gm38565	0.4054636	0.5436918	0.4408805	0.8320593
Gadd45g	0.158075	4.153746	0.4409662	0.8320593
Ehhadh	-0.1829883	2.2924812	0.4410379	0.8320593
Ddx4	0.3179579	0.8994306	0.4410389	0.8320593
Fubp3	0.099692	4.870575	0.4410864	0.8320593
Ppp2r3c	-0.1103265	4.5963505	0.4411188	0.8320593
Gm46172	0.4415722	-0.3466207	0.4411959	0.832104
Tmem248	-0.0848963	6.4082174	0.4414254	0.8321852
Slc45a3	-0.2234786	2.6956915	0.4414311	0.8321852
Dcaf5	0.0909685	5.2678381	0.4415451	0.8321852
Mylk	-0.0960167	6.3632853	0.4416696	0.8321852
Cd4	-0.1439801	3.8047631	0.4417068	0.8321852
Fam171b	0.3931087	0.8078241	0.4417146	0.8321852

Rac3	0.2838428	-0.0152594	0.4417257	0.8321852
Rabggta	-0.1219214	3.5040192	0.4418886	0.8321852
Rps6ka5	-0.1530821	4.9107003	0.4419578	0.8321852
Smurf1	-0.1414798	4.3023098	0.4419736	0.8321852
Gm5831	0.3370382	-0.0119389	0.4419841	0.8321852
Tnfaip3	0.122819	4.4811927	0.4420042	0.8321852
Gm7240	0.2505809	1.0562849	0.4420122	0.8321852
Rnf135	0.1913706	2.5087823	0.4420211	0.8321852
4930426I24Rik	0.5078024	-0.8185963	0.4420416	0.8321852
Ccdc124	0.0900101	5.9330574	0.4421004	0.8321953
1700003F12Rik	-0.3742974	-0.1521127	0.442403	0.8326487
Snhg4	0.1674023	3.7935455	0.442492	0.8326487
Vhl	-0.0973325	4.7393084	0.4425103	0.8326487
Dsel	0.2492408	2.8102887	0.442727	0.8326487
Car15	-0.360244	-0.0566123	0.4427313	0.8326487
Mrps5	0.1305127	4.7253703	0.442749	0.8326487
Immp1l	-0.1266375	4.3328567	0.4427983	0.8326487
Bcl2	-0.1135738	4.4808415	0.442811	0.8326487
A630034I12Rik	0.155687	4.9515262	0.4428231	0.8326487
Dedd2	-0.0909828	6.1704278	0.4428792	0.8326535
Fam76b	0.1014058	6.5574231	0.4430891	0.8329475
Gm16794	0.3872073	-0.4312246	0.4432779	0.8332018
Dcstamp	-0.2653308	3.2545461	0.4433393	0.8332031
B4galnt3	-0.2783084	2.323382	0.4433858	0.8332031
Gm5559	0.2365442	0.8675998	0.4434672	0.8332554
Gm13012	0.1854698	2.7561744	0.4435502	0.8333108
Rnf8	0.1995052	2.6965065	0.443632	0.8333638
Pno1	0.1326152	5.1044477	0.4437461	0.8334621
Immt	0.0854985	6.9323034	0.443798	0.8334621
Gpm6b	-0.1290208	3.9475247	0.4439382	0.8334621
LOC108167518	-0.1891117	3.3259887	0.4439428	0.8334621
Cpsf3	-0.0981723	5.7746396	0.4439632	0.8334621
Crtap	0.2092819	5.2208843	0.4440058	0.8334621
Wdr37	-0.0932893	5.1924157	0.4440684	0.833479
Mmp13	-0.2494428	8.8705085	0.444147	0.8335259
Rgcc	0.1140268	6.2714523	0.4442122	0.8335477
Upp2	-0.3397055	0.2266967	0.4444478	0.8338891
Kazald1	0.3273775	4.7775442	0.4445703	0.8339384
Tspan12	-0.1969135	2.6244557	0.4445813	0.8339384
Gps1	0.0862531	5.2398007	0.4447684	0.8341888
Ccdc167	0.1096309	4.4575351	0.4449483	0.8344255
Cfd	0.4269681	6.0608496	0.4452614	0.8347635
Kpnb1	0.0902279	8.663639	0.4453559	0.8347635

Gm15466	0.4520675	-0.2968561	0.4454028	0.8347635
Lrp12	-0.150691	3.2183125	0.4454641	0.8347635
Fto	0.1011977	5.3827102	0.445521	0.8347635
Rras	0.159601	3.6893798	0.4455349	0.8347635
Cdkn1c	0.2394423	3.1229186	0.4456051	0.8347635
Cog5	-0.0880257	5.5514393	0.4456608	0.8347635
Fancm	-0.1276638	4.9217255	0.4456802	0.8347635
Htra3	-0.1950033	4.140794	0.4457417	0.8347635
Rabep2	0.1293669	3.982983	0.4457502	0.8347635
Igfbp5	0.1169462	9.1468995	0.445887	0.8347635
Ctbs	-0.1243731	3.7306073	0.4459066	0.8347635
Kpna1	0.0925465	6.585567	0.445964	0.8347635
Kif26a	0.1915233	2.0284761	0.4459666	0.8347635
Cpsf2	0.0947789	6.9422811	0.4459884	0.8347635
Dnajc17	0.1931653	2.6165344	0.4460409	0.8347635
Cyb561a3	0.0903344	5.9549065	0.4461389	0.8348408
Rnf182	0.4529789	0.5322685	0.4461895	0.8348408
Ythdf3	-0.0959527	6.7519589	0.4465141	0.83527
Kcna4	0.3264575	0.3528041	0.4465263	0.83527
Stx1b	-0.4056578	-0.8052902	0.4466982	0.8354911
Hip1	0.1189417	5.0078885	0.4467727	0.83553
Hoxc8	0.4183528	0.7471987	0.4468756	0.8356219
Tgfbr2	-0.1504725	6.80841	0.4469474	0.8356558
Pold2	0.1120372	4.7851845	0.4470773	0.8357698
Gm16372	0.3133647	-0.1332036	0.4471159	0.8357698
Kbtbd7	-0.0859022	5.8397511	0.4472465	0.835899
Gm8203	0.1501459	3.2110555	0.4472925	0.835899
Phc1	0.1489279	3.6328653	0.4473865	0.8359742
Trip13	0.1318709	4.4683973	0.4475654	0.8359962
Cnr1	-0.3176263	1.1819474	0.4475675	0.8359962
Atf7	-0.1186903	5.8336739	0.4475714	0.8359962
Gm36556	0.1806556	2.5581202	0.4477952	0.8359962
Slc22a5	-0.1936323	2.8290914	0.4478152	0.8359962
Gm30431	0.255502	0.964864	0.4478315	0.8359962
Mgmt	-0.1593377	2.8126479	0.4479112	0.8359962
Taf1b	-0.1148619	4.1009166	0.4479841	0.8359962
Arhgef9	0.2352304	0.9988444	0.448037	0.8359962
Pan3	0.094581	6.7032272	0.4480427	0.8359962
Zc3h7a	0.1049966	6.670001	0.4480858	0.8359962
Olfml2b	0.1307747	6.6085192	0.4480927	0.8359962
Prdm15	0.138437	3.7890923	0.448097	0.8359962
Rps15a	0.1289431	8.6551249	0.4482941	0.8362637
Zfp558	-0.2676845	0.2527843	0.4483818	0.836327

Trim72	-0.6212895	-0.8622793	0.4485255	0.8363522
Ahnak	-0.0954777	7.2287426	0.4486587	0.8363522
Slc38a2	-0.1041037	7.7091066	0.448697	0.8363522
Acsl6	0.224039	2.0035304	0.4487021	0.8363522
Fam168b	-0.0994287	7.5946668	0.4488233	0.8363522
Dcbld1	0.3062256	1.6090149	0.4488537	0.8363522
Ado	0.1316351	4.4572892	0.4488749	0.8363522
Fbxw9	-0.2233592	1.7303018	0.4488885	0.8363522
Parm1	-0.3824391	0.1992784	0.4489603	0.8363522
Smc6	-0.0952861	7.4875348	0.4490096	0.8363522
Cep85	0.1452405	5.1582402	0.4491245	0.8363522
Zfp788	0.1404335	3.5764754	0.4492659	0.8363522
Ptprs	-0.1813683	6.6621962	0.449304	0.8363522
Cdh24	0.3356513	1.3290428	0.449358	0.8363522
Kcnn1	0.4311302	0.2214791	0.449391	0.8363522
Hyal1	-0.1660666	3.6181224	0.449414	0.8363522
Nsun6	-0.1404151	3.2282363	0.4494282	0.8363522
Reps1	-0.1016851	5.202074	0.4494582	0.8363522
Mknk2	0.0861249	7.7355646	0.449508	0.8363522
Sft2d1	-0.1051454	4.9343203	0.449525	0.8363522
B4galt1	0.0916899	7.186926	0.4495543	0.8363522
Ipo7	-0.0877966	7.4820635	0.4496505	0.8363522
Osgepl1	0.1459604	4.0803866	0.4496889	0.8363522
Stau1	0.0823507	5.9056446	0.4497265	0.8363522
Clec11a	0.2352996	4.9008987	0.4497396	0.8363522
Flywch1	0.136614	3.7861472	0.4499402	0.8366253
Jagn1	0.1219213	3.833313	0.4501399	0.8368345
Jdp2	-0.1317536	5.9420188	0.4501967	0.8368345
Slc16a7	-0.1285837	4.3310422	0.4502142	0.8368345
Golph3	-0.0846375	7.323225	0.4503035	0.8369006
2610037D02Rik	0.3457965	0.0136043	0.4504108	0.8369794
Ppig	0.0916703	7.1721331	0.4504667	0.8369794
Msi2	0.1379484	6.1615143	0.4505074	0.8369794
Pigt	0.0943353	5.9392007	0.450607	0.8370645
Atpif1	0.158837	9.0146007	0.4507147	0.8371646
Gadd45b	-0.1165851	3.9778213	0.4508225	0.8372435
Rin2	-0.1054214	5.4074991	0.4509389	0.8372435
Inpp5d	0.0973508	7.17647	0.4509602	0.8372435
Selenos	0.1181671	5.5944684	0.451151	0.8372435
2010110K18Rik	0.290965	1.3258752	0.4511526	0.8372435
Gm4285	0.2429617	1.4260133	0.4511667	0.8372435
Gm33280	0.1635181	3.3814008	0.4512185	0.8372435
A930024E05Rik	0.3388624	0.2696849	0.4513784	0.8372435

Gm14403	0.3263741	1.0635718	0.4513801	0.8372435
Zbtb18	0.1203518	6.4517703	0.4513876	0.8372435
LOC118568450	0.4256344	0.1448153	0.4515532	0.8372435
Atp6v1c1	-0.0993033	6.370438	0.4515987	0.8372435
Xrcc5	0.1156484	4.3530594	0.4517222	0.8372435
Fkbp14	0.2278734	3.7740219	0.4517323	0.8372435
Hsp90aa1	-0.1008925	9.41281	0.4517457	0.8372435
Cnksr3	0.1286318	3.4543567	0.4517693	0.8372435
Tbccd1	0.113552	4.9740967	0.4517733	0.8372435
Gm30189	0.1756643	2.5830193	0.4518244	0.8372435
Gnpnat1	-0.1488431	3.6242486	0.4518326	0.8372435
Rbm47	-0.1241269	3.8082416	0.4519347	0.8372435
Rpl19	0.0992434	9.6445358	0.4519524	0.8372435
Rtraf-ps	0.3811989	-0.6352627	0.451969	0.8372435
A530088E08Rik	-0.4550191	-0.7668422	0.4519952	0.8372435
Plekhh3	-0.2176539	2.0824825	0.4520603	0.8372448
Spin4	-0.3175367	0.0462267	0.4521036	0.8372448
Tm2d3	0.1415443	3.7184447	0.4522458	0.8373631
Creb1	-0.0913328	6.9692322	0.452312	0.8373631
2610002M06Rik	0.0941778	5.1585973	0.4523556	0.8373631
Taf5l	0.0925464	5.1913637	0.4523891	0.8373631
BC005537	-0.1011122	7.9568484	0.452485	0.8373631
Zfp330	0.0986781	4.7848677	0.4525362	0.8373631
Ppm1e	0.2091531	4.4908837	0.4526487	0.8373631
Rps10-ps2	0.175649	2.7149719	0.4526937	0.8373631
2700049A03Rik	-0.1320206	4.2682066	0.4527911	0.8373631
Xxylt1	0.1599711	2.9337958	0.4528107	0.8373631
Slc26a8	-0.3584673	0.419327	0.4528133	0.8373631
BC034090	-0.2833573	0.8542055	0.4529706	0.8373631
Pitpnm1	-0.0862553	6.083918	0.4530164	0.8373631
Etv1	0.2103613	1.7046525	0.4530392	0.8373631
Gm7143	0.4162117	-0.3692082	0.4530706	0.8373631
Nupr1l	0.4892579	-1.2074796	0.4530708	0.8373631
G6pc3	0.1073195	4.3552062	0.4530827	0.8373631
Eif4h	0.0835148	8.1451604	0.4532452	0.8373651
Ndrg2	-0.1750397	3.61748	0.4532577	0.8373651
Numa1	-0.090028	7.6221561	0.4533122	0.8373651
Lrrk1	0.1083662	4.6817443	0.4533865	0.8373651
Vkorc1	0.1755742	4.4496741	0.4534583	0.8373651
Helz2	-0.1254237	5.7503251	0.4534768	0.8373651
Ranbp1	0.089616	7.575964	0.453478	0.8373651
Sap30	0.0927393	5.3900059	0.4535145	0.8373651
3110082I17Rik	0.1685238	3.8002065	0.4536708	0.8375543

Dpagt1	-0.1095184	4.7105029	0.4538172	0.8377252
Slc44a1	-0.0827406	6.4997185	0.453922	0.8377968
Cyp2d22	0.3671625	0.8652622	0.4539637	0.8377968
Gm40841	0.3050468	1.5848417	0.4540918	0.8379339
Tsc22d3	0.2898044	6.968833	0.4542408	0.8380267
Knl1	-0.1145552	7.6512462	0.4542536	0.8380267
Gm9118	0.251613	1.5903152	0.4543038	0.8380267
Alyref	0.0896231	7.072807	0.4546387	0.8383648
B4galt3	-0.1068853	4.6548223	0.4546443	0.8383648
Fam120b	-0.1004083	4.5495648	0.4546488	0.8383648
Gm5500	0.4175728	-0.5454764	0.4548863	0.8384102
Smim5	-0.1260624	4.8409321	0.4549647	0.8384102
Vps37c	-0.1053576	4.4884855	0.4549968	0.8384102
Chmp7	0.0997825	4.716637	0.4550083	0.8384102
Gm30286	-0.2165007	2.4712313	0.4550257	0.8384102
Ier2	-0.1201908	5.6116728	0.4550648	0.8384102
Map1b	-0.2334989	2.7676113	0.4551141	0.8384102
Acot7	-0.0990811	4.69157	0.4551438	0.8384102
Adnp2	-0.0943902	4.6991334	0.45519	0.8384102
Pgap3	-0.2289373	2.2458242	0.4553526	0.8384102
Ift57	0.1231157	4.069708	0.4553827	0.8384102
Bmp4	0.2640956	2.8834156	0.4553944	0.8384102
Pkd1	-0.1030359	5.2841912	0.4554	0.8384102
Cdk8	0.0994679	5.8916616	0.4554598	0.8384102
Gigyf2	-0.1004342	6.3295975	0.455482	0.8384102
Gm14410	0.1608212	2.5500084	0.4556691	0.8386378
Aagab	-0.0889362	5.8015176	0.4557281	0.8386378
Ptp4a2	0.083644	8.4644677	0.4559373	0.8386378
Tsc22d2	-0.0905241	5.3116808	0.455954	0.8386378
Tial1	0.0859677	6.7901787	0.4559661	0.8386378
Tagln	-0.3255882	1.7159999	0.4561019	0.8386378
Tnip1	-0.1068137	5.5286572	0.4561048	0.8386378
Ahcy	0.1115236	5.7195305	0.4562697	0.8386378
Camkk2	-0.1042868	5.6867854	0.4562783	0.8386378
Myo1b	-0.1920239	5.802874	0.4563576	0.8386378
Morc4	0.2403694	1.5774505	0.4563706	0.8386378
Rbm34	0.1101976	4.3026244	0.4564515	0.8386378
Ptprf	-0.1935628	3.8007725	0.4564579	0.8386378
Cdc73	-0.0904641	5.8850803	0.4565183	0.8386378
Gm15706	0.3267667	0.5384679	0.4565623	0.8386378
LTO1	-0.1218696	4.1692635	0.4566585	0.8386378
Gcnt1	0.1404731	6.7201502	0.4566778	0.8386378
Zfp619	-0.1966062	2.541958	0.45673	0.8386378

Ppic	0.2124818	6.072609	0.4567486	0.8386378
Abi2	-0.1084632	4.47139	0.4567769	0.8386378
Plcd1	-0.2730344	3.3267991	0.4567901	0.8386378
Cep126	-0.3574153	1.2572581	0.4567918	0.8386378
Nasp	0.1131294	7.5741357	0.4568917	0.838649
Ccnb1ip1	-0.6231158	2.6554408	0.4569057	0.838649
Itsn1	0.1200119	7.0745054	0.4570191	0.838722
Fam72a	0.183194	2.060486	0.4570533	0.838722
Cfap161	0.3736755	1.3816328	0.4571306	0.8387648
Ciart	0.5051614	-0.1062387	0.4572166	0.8387846
Gmnn	0.1255078	6.2809607	0.4572492	0.8387846
Endov	0.1530906	3.3724443	0.4574849	0.83909
Mxi1	-0.0952673	6.9172646	0.4575236	0.83909
Lrrc59	0.0911131	6.8414355	0.4577418	0.8393912
Med31	-0.1304146	3.5354884	0.4579163	0.8395457
Capza2	-0.0889836	7.8487163	0.4579973	0.8395457
Gtf3c4	0.1130793	5.33557	0.4580613	0.8395457
Csnk2a2	0.0837015	5.783845	0.4581023	0.8395457
Gm9923	-0.5092702	-0.6429706	0.4581777	0.8395457
Trak2	0.1379881	8.9714657	0.4581888	0.8395457
Plcg2	-0.13117	7.3623646	0.458271	0.8395457
Rpl6	0.1005114	9.7266122	0.4583156	0.8395457
D11Wsu47e	-0.163361	3.4597766	0.4583983	0.8395457
Gm5548	-0.6905983	1.4393962	0.4584335	0.8395457
Mrpl44	-0.0945768	4.7576088	0.4584449	0.8395457
Src	-0.1234782	5.062903	0.4584738	0.8395457
Coa8	0.1111394	3.9215739	0.4586035	0.8395664
Cavin3	0.2008502	3.2382373	0.4586668	0.8395664
Parvb	-0.1210865	7.7450333	0.4586771	0.8395664
Abcg2	0.1727106	6.7606538	0.4587523	0.8395664
Uap1l1	0.1188471	4.3808691	0.458755	0.8395664
Erf	0.1392504	4.2025298	0.4588286	0.8395942
Tmed10-ps	-0.3036051	0.4639981	0.4588781	0.8395942
Ccdc162	-0.3879746	-0.6673945	0.4590451	0.8396517
Mrpl17	0.1170907	4.757692	0.4590595	0.8396517
Pltp	-0.1587908	4.5182611	0.4591113	0.8396517
Usp1	0.0951788	7.6882663	0.4591255	0.8396517
Hnrnpu	0.0877121	10.089436	0.4592655	0.8397188
Tapbp	0.0845799	7.3725277	0.4592819	0.8397188
Elp4	-0.125603	3.6932673	0.4593241	0.8397188
Agap3	0.1078009	4.8546853	0.4594445	0.8398401
Dok5	0.5639903	-0.0550711	0.4595809	0.8399908
Prkdc	-0.1004206	5.2886691	0.4597892	0.8401551

Cdk19	-0.1085222	6.5389627	0.4597943	0.8401551
Adsl	0.1212773	4.9296066	0.4598329	0.8401551
Spag5	0.1134962	6.8169726	0.4599785	0.8402794
Btd	0.1232357	3.7414553	0.460009	0.8402794
Slc38a10	-0.0901354	6.8283603	0.4601172	0.8403785
Mfsd13a	0.2489267	3.5241965	0.4602248	0.8404762
Zbtb25	0.1045595	4.0127755	0.460324	0.840536
Naip5	-0.1628899	3.7361423	0.4603656	0.840536
Ppp1cb	0.100686	9.3335541	0.4605934	0.8408533
Cdkn2aipnl	0.0853694	6.1574882	0.4606808	0.840914
Ngly1	-0.1219794	5.4490259	0.4607679	0.8409744
Cbx3	0.0776901	6.8812591	0.4608335	0.8409954
Xpot	0.0805391	6.2436094	0.4610711	0.8412343
Kcnc3	-0.306596	1.1156076	0.4612372	0.8412343
Gm29417	-0.377225	-0.2884837	0.4612446	0.8412343
Tubb4b-ps1	0.3276405	0.3767515	0.4612459	0.8412343
Gpatch1	0.1284183	3.6951758	0.4612761	0.8412343
Zbed3	0.1580074	3.485188	0.4613228	0.8412343
Ccm2l	0.2250907	1.7471342	0.461343	0.8412343
Dnajc30	-0.1267494	3.780503	0.4614537	0.8413376
Wdr41	-0.1146832	4.6275282	0.4617562	0.8416652
Aplnr	-0.1770729	3.7527882	0.4617624	0.8416652
Stx12	-0.0785606	5.722144	0.4618191	0.8416652
Maml2	0.175392	3.3873206	0.4618498	0.8416652
G430095P16Rik	0.1981332	2.2617542	0.4621305	0.842078
2610035D17Rik	0.621531	0.1904402	0.4622169	0.8421367
Plxna3	0.2766818	1.3638662	0.4622755	0.8421449
Napb	-0.3887683	-0.2670147	0.462408	0.8421708
Gm8091	0.290245	2.9435129	0.4624207	0.8421708
Mettl21a	-0.175125	3.0093805	0.4624522	0.8421708
Cog7	0.1208953	4.3079171	0.462658	0.8423692
Ss18l1	-0.1722451	2.6684667	0.4627106	0.8423692
Stimate	0.1270558	3.5829721	0.4627236	0.8423692
Ccr10	-0.4223869	0.0110016	0.4627925	0.8423806
Wdfy1	-0.1089592	4.4573524	0.4628381	0.8423806
Pgap4	-0.2020273	2.117782	0.4630201	0.8424749
Atl1	0.2049921	1.7126053	0.4630437	0.8424749
Btbd9	-0.1688374	4.6606946	0.463104	0.8424749
Gm12185	0.4481802	-0.7145628	0.463137	0.8424749
Cd28	-0.2686911	1.842218	0.4631608	0.8424749
Gm5763	-0.5059627	-1.1591256	0.46333	0.8425691
Pfdn5	0.1172272	7.1587717	0.4634443	0.8425691
Pex10	0.1762865	2.5885406	0.4634776	0.8425691

Pip5k1b	-0.1110208	5.4163931	0.4634859	0.8425691
Ptpru	-0.2249628	2.0016303	0.4635196	0.8425691
Rsrc2	-0.0842472	6.7969873	0.4635376	0.8425691
Rmi1	0.0916556	5.0169559	0.463743	0.8428423
Scap	0.1116307	5.2308119	0.4637963	0.8428423
Cnmd	-0.4624911	3.2728188	0.4639653	0.843051
Sobp	-0.2580868	1.9777524	0.4640962	0.8431903
Kctd18	-0.1381264	3.1264116	0.4642438	0.8433265
Cep128	-0.1163818	6.0226591	0.4643227	0.8433265
Sfi1	0.1398832	3.7994696	0.4643428	0.8433265
Zfx	-0.0812125	6.0492461	0.4644041	0.8433265
Igkv5-45	-0.5374876	-0.2317193	0.4644422	0.8433265
Ttc17	-0.1113129	4.9570567	0.464533	0.8433314
Hsph1	-0.1561509	6.3700386	0.4647825	0.8433314
Penk	0.3797937	0.7362443	0.4647982	0.8433314
Timm44	0.0846708	5.2741919	0.4648015	0.8433314
Zfp644	-0.0969482	5.9693487	0.4648308	0.8433314
Rian	-0.4548373	-0.4102956	0.4649905	0.8433314
Olfm2	0.3984784	0.5638338	0.4649908	0.8433314
Sf3a3	0.0853203	6.1371283	0.4650041	0.8433314
Etfa	0.0870073	6.3422538	0.465005	0.8433314
Zfp523	-0.1361845	3.7269428	0.4650509	0.8433314
Farsb	0.1069223	5.9304633	0.4651239	0.8433314
Aplp2	0.0875967	7.9459952	0.4651451	0.8433314
Pcsk6	-0.2840113	5.5761723	0.4651619	0.8433314
Vrk1	0.1080287	6.7847655	0.465204	0.8433314
Zfp606	0.1287486	3.7408918	0.4654253	0.84338
Gnl2	0.1038807	5.5606004	0.465446	0.84338
Egln3	-0.1470007	3.4980836	0.4654831	0.84338
Ppp4c	0.081586	6.292631	0.4655351	0.84338
Ssr2	0.0853006	6.4651814	0.4655615	0.84338
Ppp4r3b	-0.0870438	7.3015582	0.4655878	0.84338
Lrrc1	-0.1267157	3.6723736	0.4656676	0.84338
Kri1	0.0887889	5.3909508	0.4657163	0.84338
Dipk1b	-0.2600927	1.3417759	0.4657188	0.84338
Gm34225	0.5606346	-0.4324302	0.4657837	0.8433992
Zbtb24	0.1061667	3.9054135	0.4658659	0.8434269
Ecpas	0.0756669	7.0299697	0.4659344	0.8434269
Il2rg	0.1018605	5.1571795	0.4659616	0.8434269
Nr1h3	0.1492112	2.8831078	0.4661057	0.8435895
Dnajb11	0.0796962	6.7183577	0.4663303	0.8438242
Ptger4	0.1110454	4.0767078	0.4664387	0.8438242
Slc26a10	0.3299666	-0.0908717	0.4665387	0.8438242

Lgals4	0.2439299	2.0855336	0.4665614	0.8438242
Bcl2a1a	0.3004278	0.9615625	0.466601	0.8438242
Gm39000	-0.3501368	-0.4804549	0.4666235	0.8438242
Rbm22	0.0861257	5.6376321	0.4666806	0.8438242
4933412E12Rik	0.2882481	0.9122815	0.4666948	0.8438242
Dcaf12	0.1133714	7.6271002	0.4667439	0.8438242
Capns1	-0.0848791	7.5917791	0.4667779	0.8438242
Tmem219	-0.1322734	3.9943158	0.4668403	0.8438389
Cct6a	0.0920656	7.6156389	0.4670797	0.8439
Pnpt1	0.0957759	5.5514307	0.4671051	0.8439
Cd6	0.3321944	0.3530842	0.4671833	0.8439
Ccser1	-0.3634668	0.0918168	0.467335	0.8439
Gm8325	0.2346447	1.2429851	0.4673369	0.8439
Gm20939	0.2020558	1.4423614	0.4673393	0.8439
S100a13	-0.1221361	4.9342758	0.4673648	0.8439
Ifi47	-0.1166629	5.2948616	0.4673951	0.8439
Rbm25	0.087461	7.715594	0.4674164	0.8439
Accs	0.1976958	2.2978145	0.4674167	0.8439
Polr3b	0.1128855	5.6695091	0.4676053	0.8441426
Larp1b	0.0938347	6.0050152	0.4679046	0.8444811
Zfp976	-0.1836013	2.3783316	0.4680754	0.8444811
Cspp1	-0.1014632	5.1250189	0.4681282	0.8444811
Gm26797	0.244101	0.5836888	0.4682283	0.8444811
Ndufs1	0.0959631	6.6306826	0.4682298	0.8444811
Acsl1	0.0988948	7.4609976	0.4682334	0.8444811
Hyal3	0.3207034	0.6872533	0.4682962	0.8444811
Klhl26	0.18854	3.1355003	0.468297	0.8444811
Optn	0.1160392	6.4038404	0.4683138	0.8444811
Ankrd37	-0.2380999	0.7373095	0.4683357	0.8444811
Npm3-ps1	0.4663447	-0.9039609	0.4685351	0.8447426
Frzb	-0.3441192	2.0625554	0.4686299	0.8447659
Fam98a	0.0892464	5.4924393	0.4686566	0.8447659
Fabp5	-0.1158358	4.4008789	0.4687785	0.8447708
Sdf4	-0.0804728	7.4525798	0.4688549	0.8447708
Carf	0.1645334	2.7670532	0.4689053	0.8447708
Mcc	-0.2638006	1.1473838	0.4689484	0.8447708
Itgb8	0.5831368	-0.4304927	0.4689786	0.8447708
Pcm1	-0.0869019	6.6224384	0.4690073	0.8447708
Tep1	0.089904	4.5382847	0.4690395	0.8447708
Adcy2	-0.17112	2.6345574	0.4691878	0.8448302
Rapgef3	0.1509429	3.2800013	0.4692678	0.8448302
LOC118568201	0.192754	2.2499426	0.4695043	0.8448302
Gm7224	-0.5067388	-0.8976079	0.4696061	0.8448302

Tnpo1	-0.0887468	7.6242404	0.4696141	0.8448302
Mysm1	-0.0941024	5.3702186	0.4697662	0.8448302
Clcn4	0.0794515	5.7237822	0.4698411	0.8448302
H2-DMb1	0.1639296	4.1581285	0.4698964	0.8448302
Prps1l3	0.0898974	6.2481214	0.4699057	0.8448302
Thoc1	0.1010047	5.562714	0.4699243	0.8448302
Tssk4	0.2424169	0.7362399	0.4699254	0.8448302
Fam207a	0.0884671	4.9304289	0.4699427	0.8448302
Pcdh19	-0.340011	1.0025741	0.4699513	0.8448302
Macir	-0.087138	6.8984971	0.469972	0.8448302
Kif21a	-0.2873148	0.9226651	0.4700993	0.8448302
Zdhhc7	0.0924617	5.0509404	0.4701349	0.8448302
Ticam1	0.1314746	3.4245427	0.4701679	0.8448302
Prss34	-0.17715	5.1826792	0.4702921	0.8448302
Gm9755	-0.3536771	-0.1834166	0.4703726	0.8448302
Dmwd	0.1252428	4.0251569	0.4704226	0.8448302
Tmem170	0.1562677	3.1194665	0.4704439	0.8448302
Irak2	-0.0947112	4.9323776	0.4704827	0.8448302
Gm14539	0.2628893	0.4461021	0.4704994	0.8448302
Tbx21	-0.3358604	0.4550141	0.4705219	0.8448302
Mrpl57	0.1076363	5.5928026	0.4705684	0.8448302
Nrg2	-0.2164039	2.9296845	0.4705802	0.8448302
Pld3	-0.1191888	6.2495123	0.4705938	0.8448302
Pfn1	-0.0959598	9.7682549	0.4706018	0.8448302
Btbd19	0.1667611	2.4800659	0.4706595	0.8448302
Ptcd1	0.0993186	4.204575	0.4707395	0.8448302
Fancl	0.1506213	4.5606179	0.4708614	0.8448302
Cntd1	0.2143974	1.4126965	0.4708852	0.8448302
Vps37a	-0.0825347	5.4713849	0.470908	0.8448302
Gm6627	0.2930525	0.0461605	0.4709193	0.8448302
Kifc5b	0.1277661	4.381762	0.4710547	0.8448358
Cdh15	0.294532	3.155664	0.4710721	0.8448358
Clip2	-0.1351019	4.3059196	0.4710853	0.8448358
Cenpw	-0.1691997	3.5431709	0.4712344	0.8448849
Phtf2	0.1073084	4.8491402	0.4712406	0.8448849
Mr1	-0.1672434	2.8129079	0.4712756	0.8448849
Gm36417	0.278227	-0.2385873	0.4716082	0.8453735
Tnfrsf12a	-0.3096399	0.9867546	0.4716569	0.8453735
Slitrk6	0.257059	1.9002893	0.4717433	0.8454186
Gm38484	0.4784889	0.310849	0.4718586	0.8454186
Dctpp1	0.1192495	4.6861968	0.4719011	0.8454186
AI115009	-0.4585569	-1.0398072	0.4719356	0.8454186
Nradd	0.3186542	1.2890757	0.4720003	0.8454186

Epc2	-0.0976551	5.805643	0.4720082	0.8454186
Acot11	0.2395882	2.6065567	0.4721864	0.8456404
Tor2a	0.0993789	4.3099395	0.4722895	0.8457161
Cnot2	0.0833621	6.6679032	0.4725306	0.8457161
Ppp1r13b	0.1239188	4.3255566	0.4726439	0.8457161
Fam118a	0.1251019	3.422368	0.4726685	0.8457161
Ddx19b	0.1054491	4.1064049	0.4726698	0.8457161
Nfatc3	-0.0893873	6.8889648	0.4726979	0.8457161
Chst7	0.1992214	1.5499407	0.4727655	0.8457161
Prorp	0.1430315	3.0969785	0.4727874	0.8457161
Gclm	0.1245621	7.4569484	0.4728218	0.8457161
Cep76	0.1296187	6.3733701	0.4728264	0.8457161
Aurkaip1	0.1207117	6.1496026	0.4728268	0.8457161
Nsun4	0.1428847	3.1109034	0.4730117	0.8459495
Pnrc1	0.1135521	6.1716471	0.4732724	0.8463186
Zfp235	-0.1605525	2.311615	0.4733515	0.8463626
Tmco3	-0.1259037	4.1952837	0.4734917	0.8465162
Smad1	-0.1152358	4.0018052	0.4735802	0.846577
Crybg2	-0.3828081	-1.0018951	0.4737367	0.8467595
Atg2a	-0.0940754	5.3990438	0.4738079	0.8467893
Ppp1r21	-0.1008005	5.5916856	0.4738801	0.8468211
Bik	0.3963713	-0.1998783	0.4739814	0.8468724
9530082P21Rik	0.2146759	1.1027554	0.4741053	0.8468724
Txndc9	0.0904716	6.2294431	0.4741211	0.8468724
Nos3	0.2555876	1.062371	0.4741266	0.8468724
E130308A19Rik	0.126886	4.308079	0.474226	0.8469527
C030016D13Rik	-0.3141854	0.1560618	0.4743389	0.8469706
Phf19	0.1967528	3.0611362	0.4743449	0.8469706
Hnrnpl	0.0784879	8.3321494	0.474426	0.847018
Kctd6	0.1277308	3.511928	0.474533	0.847023
Igfbp2	-0.2133344	1.7851334	0.4745418	0.847023
Kif17	0.2781957	1.1871465	0.4746171	0.847023
Nln	0.1091732	4.461022	0.4748015	0.847023
Rpgrip1l	-0.1153071	3.9521195	0.4748125	0.847023
Siah1a	0.1224235	4.4667208	0.4748186	0.847023
Fads1	0.0891449	5.8276067	0.4748645	0.847023
Bmp2k	-0.0951115	6.626133	0.4748681	0.847023
Klhl25	0.1337779	4.7707612	0.474931	0.847023
Commd8	0.0998275	5.8201724	0.4751366	0.847023
Ncln	-0.076771	5.9220263	0.4751569	0.847023
Pdzk1ip1	0.1369406	6.1222038	0.4751585	0.847023
Trim47	-0.1753583	2.7645228	0.4751971	0.847023
Wdr66	-0.3725635	-0.5962413	0.4752061	0.847023

Ift27	0.1101272	3.7465213	0.4752692	0.847023
Fam20c	-0.2008514	4.1261243	0.4753	0.847023
Endod1	0.1229089	6.8560384	0.4754556	0.8472032
ND4	0.1396085	11.447147	0.4755198	0.8472205
Rtl6	0.2106908	2.5546769	0.4756125	0.847251
Cmtm4	-0.2197655	4.3862612	0.4756459	0.847251
Phc2	0.0853967	5.8561338	0.475897	0.8476013
Lpar2	0.1654589	2.6923837	0.4760483	0.8477736
Mrpl46	0.1118838	4.1953941	0.4761448	0.8478348
Amacr	0.2553008	1.6419585	0.4761917	0.8478348
Adm	0.3967602	0.162529	0.4762606	0.8478605
Tyms	0.1148354	6.6126881	0.4763842	0.8479275
Tmed10	-0.081982	7.5483454	0.4764418	0.8479275
Mea1	-0.0874284	5.1093969	0.4765274	0.8479275
Gpx1	0.1295661	10.68578	0.4765681	0.8479275
Ggps1	0.0822319	5.2654675	0.4765708	0.8479275
Cd24a	0.1267481	11.533446	0.4767216	0.8480592
Sesn2	-0.136883	3.5514236	0.4767539	0.8480592
Lax1	0.2150077	3.1279184	0.4768784	0.8481838
Maff	-0.5108482	0.6175822	0.4769953	0.8481912
Tns4	-0.2880405	0.8454852	0.4771635	0.8481912
Gm46485	0.6349163	-0.7890243	0.4772151	0.8481912
Dtnb	0.1265942	4.2154776	0.477223	0.8481912
Ormdl1	-0.0913632	5.0619134	0.4772587	0.8481912
Gnal	-0.3157233	0.9591322	0.477274	0.8481912
Bmp6	-0.1242265	4.1056052	0.4773128	0.8481912
Brf2	-0.1222475	3.0389464	0.477345	0.8481912
LOC118567921	0.2979505	5.3135589	0.4773734	0.8481912
LOC118567636	-0.2065726	1.8773615	0.4774536	0.8482368
Slc25a28	0.1234271	4.5346162	0.4775117	0.8482431
Tatdn3	-0.208939	2.022307	0.4776594	0.8483057
Mbd1	0.0819001	6.0212516	0.4777014	0.8483057
Ptgis	-0.2623085	4.8865079	0.4777105	0.8483057
Sclt1	-0.1262011	3.9135016	0.4778788	0.8484362
Gm19391	-0.3799292	0.3474247	0.4778931	0.8484362
Myg1	0.1151662	4.2924997	0.4779893	0.8485102
Lmf1	0.1428954	3.1552599	0.4780452	0.8485125
Trim34a	-0.15093	4.0246444	0.4782798	0.848696
Enah	-0.1876951	4.826191	0.4783682	0.848696
Ccsap	0.1465756	3.4119736	0.4784109	0.848696
Nbr1	-0.0763944	7.0798275	0.4784277	0.848696
Tra2b	0.0792653	7.8937713	0.4784477	0.848696
Dhx35	0.1390934	3.4159124	0.478476	0.848696

Nat9	-0.1347501	3.2082617	0.4786776	0.8488868
Ube2k	0.0795732	6.82048	0.4787469	0.8488868
Osmr	-0.2009119	3.7386275	0.4787473	0.8488868
Fbxl22	0.2425294	0.4311433	0.4788458	0.8489646
Grip1	0.4118484	0.4602994	0.4789684	0.8490854
Lox	-0.2207167	7.7362761	0.4791839	0.8493706
Gem	0.2606205	1.2609761	0.4793267	0.8495268
Zcchc2	-0.1143093	5.49192	0.4795254	0.8497056
Ska3	0.1043727	4.7565303	0.4795368	0.8497056
4930481A15Rik	-0.4868845	0.9017542	0.4796367	0.8497551
Pced1b	0.1480224	3.5745336	0.4797279	0.8497551
Phf21a	-0.1013636	5.3396219	0.4797287	0.8497551
Oosp1	-0.264899	0.6101599	0.4799119	0.8499829
Ptges3l	-0.3263203	0.9471995	0.4799721	0.8499927
Gm33205	-0.6080526	-0.8813476	0.4800779	0.8500834
Nxpe4	0.1778783	4.1481947	0.4801587	0.8501297
Ptcd2	0.102707	4.631303	0.4804311	0.850515
Osbpl1a	-0.1191552	4.4714481	0.4806915	0.8506148
Mpc1-ps	-0.1654695	2.4159617	0.4807443	0.8506148
Fxr2	0.077311	6.1266074	0.4808241	0.8506148
Pbx3	0.1868805	3.2711219	0.4808292	0.8506148
Resf1	-0.0848201	6.8547096	0.4808384	0.8506148
Mpp2	0.1250368	4.8693028	0.480921	0.8506148
Cysltr1	-0.1902854	2.1966557	0.4809344	0.8506148
Qrich1	0.0853097	6.4951067	0.4810318	0.8506148
Arl13b	-0.1370184	3.4820689	0.4810602	0.8506148
Mmp28	-0.2304551	1.3803194	0.4811253	0.8506148
Dnah12	-0.3769345	-0.6954788	0.481134	0.8506148
Tpcn1	0.1080863	6.9988161	0.4811437	0.8506148
Ralgds	0.1421702	3.8972847	0.4813164	0.85071
Sbf1	0.0781986	6.3291132	0.481325	0.85071
Fbxo44	-0.2834032	0.2300856	0.4814138	0.85071
Zfp433	-0.2482914	0.7002547	0.4814411	0.85071
Ehmt1	0.1036864	5.7652609	0.481471	0.85071
Pak4	0.108967	3.7553913	0.4816062	0.8508523
Arl6ip5	-0.0886281	6.158578	0.4819408	0.8512472
Anapc13	-0.1114177	5.4185831	0.4820651	0.8512472
Clec4a1	-0.168693	3.8508728	0.482075	0.8512472
Nup188	0.0880459	5.9672568	0.4820883	0.8512472
Zbtb12	0.1782209	2.9182218	0.4821034	0.8512472
Lrsam1	-0.1399702	3.1621152	0.4822809	0.8514639
Slc25a30	-0.1867578	2.9718541	0.4827011	0.8521091
Cox8a	0.0842233	7.1311867	0.4829499	0.8523414

Cib1	0.0929719	5.1729134	0.4829777	0.8523414
Specc1	0.1366507	7.7908868	0.4829971	0.8523414
Cnnm1	0.2219964	1.3312237	0.4831825	0.8525718
Igkv5-43	-0.2565453	3.4306075	0.4834764	0.8528773
Calml4	-0.2795802	0.8589845	0.4834768	0.8528773
Dynlt1-ps1	0.2843447	0.7961588	0.4835289	0.8528773
Gm16118	-0.3058849	0.4668915	0.483575	0.8528773
Gm5648	0.4169561	-0.0846701	0.4836331	0.852881
8030431J09Rik	0.5058365	-0.9586368	0.4838242	0.852881
Cdk13	-0.0957491	6.8165927	0.4838297	0.852881
Rab8a	-0.074542	7.1064665	0.4838372	0.852881
Slc22a4	0.1577372	5.1551946	0.4838596	0.852881
Efs	0.2813973	2.2925679	0.483946	0.852881
Rab26os	0.2241883	2.6767931	0.4839609	0.852881
Tecrl	0.3857251	-0.8630915	0.4841014	0.8529273
Rpl35	0.124306	9.41156	0.4841355	0.8529273
Plscr3	0.0959226	5.3375512	0.4842443	0.8529273
Zbtb45	0.125681	4.2739403	0.4843421	0.8529273
Baz2a	-0.085199	7.1401795	0.4844739	0.8529273
Gm6158	0.289625	-0.2516834	0.4845258	0.8529273
Mpp1	0.1037213	7.6749368	0.4845369	0.8529273
Lmbr1l	-0.1264741	3.5443039	0.4845608	0.8529273
Cd82	0.103171	7.7273268	0.4845698	0.8529273
Kcnj8	-0.1662502	3.1845357	0.4845744	0.8529273
Gm36195	-0.3391359	-0.2022841	0.4845904	0.8529273
Ifi214	0.3286355	0.1340595	0.4849086	0.8532732
Cd300lg	0.1543001	4.0204994	0.484942	0.8532732
Stk36	0.3305041	-0.4762955	0.4849515	0.8532732
Zfp622	0.0850919	5.2922682	0.4852259	0.8536116
Tigd2	0.1129222	4.0113392	0.4853076	0.8536116
Rnasek	0.0964649	6.5127665	0.4853461	0.8536116
Sema7a	-0.171425	5.2100152	0.4854469	0.8536116
Cir1	-0.1088273	5.3331281	0.4854605	0.8536116
Tmem39b	0.1772567	3.0919006	0.4854842	0.8536116
BC005624	0.081349	5.8372663	0.4855716	0.8536116
4930558J18Rik	-0.2378486	0.6423013	0.4855828	0.8536116
Epm2a	-0.2497234	2.0471312	0.4857432	0.8537682
Snrpd3	0.0888371	6.4395558	0.4857878	0.8537682
Trp53rka	0.1534737	3.14185	0.4858509	0.8537682
Pdgfc	-0.2107365	3.4067733	0.4859562	0.8537682
Gm46845	0.456756	-0.7519395	0.4859884	0.8537682
Noc3l	-0.114234	4.3491811	0.4860924	0.8537682
Bglap3	0.420775	-0.7217571	0.4860979	0.8537682

Gm46058	0.5550162	-1.0532747	0.4861111	0.8537682
Lyrm7	0.25108	1.056434	0.4862516	0.8539186
Il13ra2	-0.1669225	2.6973953	0.4863673	0.8540254
Tbc1d25	-0.1592411	3.1100932	0.4864613	0.854092
Yy2	0.1871523	2.1605973	0.486515	0.854092
Pfkl	0.08324	5.3799582	0.4866599	0.8542463
Sphk2	-0.0894091	5.0228597	0.4867357	0.8542463
Fign	-0.2648896	2.4725439	0.4867677	0.8542463
Zbp1	-0.200533	2.8612013	0.4868354	0.8542608
Kdm4a	0.0712597	6.2106945	0.486896	0.8542608
Jam2	-0.2421778	3.3048121	0.4869421	0.8542608
Magee1	0.1573351	2.3834113	0.4870216	0.8542608
Pyroxd2	-0.1345222	3.5184368	0.4870901	0.8542608
Tspoap1	-0.1715502	4.446768	0.4871423	0.8542608
Gm11808	0.134445	2.7647218	0.4872195	0.8542608
Gm9625	-0.2164781	2.2110666	0.4873199	0.8542608
Gm6988	0.2304197	1.0562455	0.4873265	0.8542608
Anp32a	0.0921596	8.2816639	0.4873761	0.8542608
Aox1	0.2708378	0.3372754	0.4873861	0.8542608
Selenop	-0.0886645	9.0834194	0.4874981	0.8542608
Ccdc181	-0.1304516	3.0220178	0.4875097	0.8542608
Kifc1	0.1103733	6.0873024	0.4875449	0.8542608
Runx3	-0.1405813	4.6608202	0.4876436	0.8543376
Lrmda	-0.1665956	2.030149	0.4877072	0.8543528
C030006K11Rik	0.1349378	3.2205276	0.4878068	0.8543909
Kmt5a	0.0905649	7.6977336	0.4878714	0.8543909
Rpl36a-ps1	0.3553041	0.5476395	0.4878938	0.8543909
Daxx	0.0796297	4.9504271	0.4879594	0.8544096
Hivep3	-0.147895	3.2785471	0.4880582	0.8544865
Hspa12a	0.2975132	0.9256432	0.4881403	0.8545214
Prrc2b	-0.1031701	6.9565843	0.4882359	0.8545214
Rps6-ps4	0.1063245	4.1667103	0.488243	0.8545214
Tmem150b	0.2452744	1.8594698	0.4885962	0.8549589
Arl4a	0.1550412	6.3157049	0.4887535	0.8549589
Crry-ps	-0.4218297	-1.008812	0.4887939	0.8549589
Nup88	0.0775376	6.0928901	0.4888788	0.8549589
Rab18	0.0821028	6.7052375	0.4888887	0.8549589
Nog	0.373278	0.2182857	0.4889542	0.8549589
Gm11772	0.3250402	0.2729489	0.4890124	0.8549589
Bnip3	0.1361916	3.7154751	0.4890269	0.8549589
Gosr1	-0.0800243	5.4863764	0.489036	0.8549589
Vac14	0.0969949	5.2103961	0.4890466	0.8549589
Tub	-0.3007355	0.8748019	0.489157	0.8549589

Swap70	0.0895104	6.4232768	0.4892116	0.8549589
Bbs4	-0.1998148	2.1584699	0.489226	0.8549589
Gm6091	0.4389165	-0.7491195	0.4892625	0.8549589
Psip1	0.0870585	7.6173286	0.489332	0.8549843
Scrn3	0.1243112	5.925621	0.489449	0.8550882
A130077B15Rik	-0.2558693	0.3975094	0.4895014	0.8550882
Rap1a	-0.0877673	7.793082	0.4897368	0.8552837
Enpep	0.1234774	3.8680907	0.4897774	0.8552837
Mrpl20	0.1236091	5.5458771	0.4898298	0.8552837
Tcf19	0.0974398	5.7030657	0.4898333	0.8552837
Baz1a	-0.1260036	7.6583674	0.4898946	0.8552947
Lrrc27	-0.2336109	1.534045	0.4899643	0.8553204
Nfu1	0.1237985	4.9133102	0.4901225	0.8554266
Bcl2l2	-0.115452	4.4945179	0.4901927	0.8554266
Sppl2a	-0.0865503	7.221205	0.4902003	0.8554266
Slc16a9	-0.1872468	2.3702098	0.4903157	0.8554266
Zmym2	-0.077025	6.1036675	0.4903848	0.8554266
Gm51701	0.2998062	-0.1349564	0.4903871	0.8554266
Tusc2	0.097285	4.4961837	0.4904373	0.8554266
Pced1a	-0.116509	3.7988722	0.4905153	0.8554266
Cbr1	0.1197983	3.8708811	0.4905201	0.8554266
Pole3	0.1186044	5.5816319	0.4905927	0.8554572
Ttc38	0.1968586	2.483442	0.4908164	0.8557514
Rnf121	-0.1434535	2.6570135	0.4909973	0.8559708
Gm14966	-0.3003132	-0.0332001	0.4912267	0.8561649
Prickle4	-0.4168016	-0.8810728	0.4912877	0.8561649
Commd2	0.1094968	3.948092	0.4913759	0.8561649
Slc30a6	0.1082449	3.5947756	0.4913804	0.8561649
Cops7b	-0.1005641	4.1406029	0.4913838	0.8561649
Gm3756	-0.3347033	-0.3598194	0.4914917	0.8561994
Gm52568	-0.4432732	-1.1813078	0.4915407	0.8561994
Rilp	-0.2486034	0.8479051	0.4915688	0.8561994
Anks3	0.1179218	4.2791922	0.4919141	0.8565272
Rora	-0.2067112	3.3980161	0.4919277	0.8565272
Rnf111	-0.0770912	5.8573001	0.4919466	0.8565272
Arhgef7	-0.0945906	5.6492957	0.4919772	0.8565272
E230029C05Rik	-0.2868326	0.9681396	0.4920998	0.8566447
Maco1	0.0778707	5.8287053	0.4922359	0.8567858
Birc6	-0.0934179	7.3006486	0.4924954	0.8570934
Cd59b	-0.1985485	1.4422026	0.4926785	0.8570934
Mkrn2	-0.0957823	4.2070387	0.492695	0.8570934
Lama5	-0.2289346	2.0678481	0.4927122	0.8570934
Kansl2	0.0823002	5.4065257	0.492791	0.8570934

Tsc22d1	0.1556778	8.0551844	0.4928144	0.8570934
Dop1a	-0.1011225	4.8844655	0.4929624	0.8570934
Phf2	0.1130501	6.4405053	0.4929824	0.8570934
Bscl2	0.0809141	5.1168683	0.4930269	0.8570934
Socs6	-0.0992956	4.4743109	0.493053	0.8570934
Sh3yl1	0.1485264	3.3751492	0.4931438	0.8570934
Fgfbp3	0.2775405	0.7088022	0.4932413	0.8570934
Lgals3bp	0.1082633	6.3018574	0.4933045	0.8570934
Tecr	-0.0822841	7.0931232	0.4933284	0.8570934
Rhoa	-0.082617	8.7505199	0.4934276	0.8570934
Rbms1	-0.0857924	6.9330872	0.4935161	0.8570934
Tmed9	0.0776934	7.0494055	0.4935762	0.8570934
Cks2	0.1030044	6.9030202	0.4937095	0.8570934
Mrps33	0.1155931	4.4004825	0.4937267	0.8570934
Prkar1a	-0.0750934	8.0759405	0.493767	0.8570934
1300002E11Rik	0.1317351	4.0106116	0.4938193	0.8570934
Tmco6	0.1395497	3.8023228	0.4938788	0.8570934
Aunip	-0.1551076	3.8775183	0.4939547	0.8570934
Eif5	0.0987308	8.587483	0.4939625	0.8570934
Asb10	-0.4208991	-0.9098989	0.4940393	0.8570934
Rybp	-0.1444374	4.6076885	0.4940416	0.8570934
Safb	0.0914559	6.9714049	0.4940771	0.8570934
Arhgef5	-0.1580689	2.6313725	0.4941326	0.8570934
Cd93	0.1354368	6.8370814	0.4941364	0.8570934
Srpr	-0.0749536	6.4875416	0.4941439	0.8570934
Spg21	-0.1000426	5.4562297	0.4942308	0.8570934
Mbd2	0.0896936	8.3635637	0.4942574	0.8570934
Prickle2	-0.2019608	2.1069609	0.4942859	0.8570934
Atad2b	-0.0950343	6.8169795	0.4942862	0.8570934
AW112010	-0.1462126	3.8406465	0.4944213	0.8572321
Tmem45b	0.6149334	-1.2484353	0.4946936	0.8573727
Agtpbp1	-0.0834978	5.5677295	0.4947706	0.8573727
Slc38a11	-0.4866509	-1.056761	0.4948493	0.8573727
Qpct	0.2555198	1.7516858	0.4949349	0.8573727
1190007I07Rik	0.1502673	2.2918931	0.4949377	0.8573727
Gm32276	0.275276	0.6943042	0.4950397	0.8573727
Gm28043	-0.207331	1.6090475	0.4950537	0.8573727
Bahcc1	-0.13961	3.5091821	0.4950646	0.8573727
B230303A05Rik	-0.2234271	1.2440757	0.495116	0.8573727
Rps27a-ps2	-0.2659402	0.1836055	0.4951509	0.8573727
Poln	0.3174744	-0.1435116	0.4951579	0.8573727
Lag3	0.274491	1.2409958	0.4951639	0.8573727
Samd5	-0.4032784	-0.7773073	0.4954453	0.8577646

Usp46	-0.0807635	6.3999473	0.4955024	0.8577679
Morc2a	0.0911327	5.2862199	0.4956828	0.8577937
Tacstd2	0.1534011	2.9431988	0.4957572	0.8577937
Ndufs5-ps	0.8145005	2.193428	0.4958523	0.8577937
Pus1	0.1125623	4.1129496	0.4958619	0.8577937
Arf1	-0.0794824	8.5837232	0.4958666	0.8577937
4933427D14Rik	-0.1373709	3.3732664	0.4959457	0.8577937
Gpr19	0.2545939	1.4711642	0.4959494	0.8577937
Ndufa12	0.0911616	6.1310114	0.4959585	0.8577937
Mau2	0.0791228	6.5809633	0.4961729	0.8578403
Gm38825	0.2279353	2.4242778	0.4962055	0.8578403
Trim68	0.1940894	1.6493376	0.4962304	0.8578403
Dusp10	0.1405483	3.4552206	0.4963011	0.8578403
Ltb4r2	-0.2725115	1.4298213	0.4963191	0.8578403
Zfp946	0.1481136	2.4328859	0.4963824	0.8578403
Klhl24	-0.122891	7.0994362	0.4964297	0.8578403
Zmiz1	0.0962505	6.947249	0.4964397	0.8578403
Acadm	-0.0774418	5.8841412	0.4964818	0.8578403
Fbxw7	0.090536	4.9645138	0.4965608	0.8578814
Slc25a47	0.3871468	-0.313671	0.496728	0.8580252
Atm	-0.1094889	5.2645306	0.4967946	0.8580252
Atp5e	0.1151895	6.3300014	0.4968716	0.8580252
Sipa1l2	-0.1238916	4.5429213	0.4969145	0.8580252
Ccdc112	-0.2903212	1.0722929	0.4969699	0.8580252
Ptdss1	-0.0768525	5.3837619	0.496975	0.8580252
Cadps2	-0.2772071	1.6986485	0.4971437	0.8582212
Coro2b	0.2376391	1.7585488	0.4972286	0.8582582
Med15	0.0804877	5.7756807	0.4972755	0.8582582
Clip3	0.3367736	1.3373159	0.4974538	0.858291
Renbp	0.1591554	3.6140596	0.4974874	0.858291
Pea15a	-0.1018497	5.0405768	0.4975271	0.858291
Lss	-0.1334665	2.7922398	0.4975739	0.858291
Hnrnpul1	0.0952295	8.6091675	0.4976657	0.858291
Mthfsl	0.2857086	2.7643584	0.4976797	0.858291
Itpa	0.1131691	5.0993858	0.4976807	0.858291
Gstm5	0.1388183	5.2137826	0.4977845	0.8583747
Gm46869	-0.5328794	-0.9177836	0.4978454	0.8583847
Ppil2	0.0703097	5.9913145	0.498007	0.8585314
Zc3h8	0.1792724	2.0254014	0.4980409	0.8585314
Yeats2	-0.0923195	4.9380041	0.4983859	0.8586254
Ints8	0.0799187	6.0472827	0.4984143	0.8586254
Dennd6a	0.0823614	5.5424632	0.4984172	0.8586254
Myo9b	-0.0715323	6.9139598	0.498607	0.8586254

Zfp62	-0.0756839	6.1965605	0.4986139	0.8586254
Tpx2	0.0953148	7.8681556	0.498631	0.8586254
Capn2	-0.0871178	6.4967865	0.4986367	0.8586254
Fbxo21	0.1213957	3.3386408	0.4986851	0.8586254
Tmem181c-ps	-0.223748	3.3386838	0.4986923	0.8586254
Zmym6	-0.1194668	3.9747524	0.4987471	0.8586254
Wdr4	0.1096634	3.9601164	0.4987495	0.8586254
Slc43a3	0.1036816	7.6866087	0.4987579	0.8586254
Med28	0.0931529	5.1152471	0.4988305	0.8586553
Pstk	0.1865706	2.3382862	0.4990504	0.8589388
Adcy6	0.0958451	6.457824	0.4991842	0.859074
Id2	-0.0965681	5.8886387	0.4993945	0.8592542
Msl1	-0.0892911	7.7212834	0.4993994	0.8592542
Patl1	-0.1182046	5.4251414	0.4995028	0.859337
Atr	-0.089681	4.9682565	0.4995714	0.8593601
Rpl11	0.0989506	9.6193848	0.4997286	0.8595355
Cox18	-0.1550239	3.2363516	0.4997856	0.8595385
Fiz1	0.0823092	4.9758817	0.4998481	0.8595509
Nxn	-0.1116296	4.4732684	0.4999624	0.8596225
Rabepk	0.1246589	3.8281892	0.5001086	0.8596225
Nyx	0.3957185	-0.7991215	0.5002286	0.8596225
Unc5c	0.3452709	1.9808145	0.5002723	0.8596225
Dgcr2	0.0878899	5.682994	0.5003137	0.8596225
Tug1	0.0762234	6.8975465	0.5003246	0.8596225
Ndrg4	0.4136837	2.3074366	0.5003919	0.8596225
Arf4	0.0971562	6.9225832	0.5004165	0.8596225
Robo3	-0.2934951	1.7825865	0.5006968	0.8596225
Gm15987	-0.1894325	1.5808901	0.5007051	0.8596225
Khdrbs3	0.1339964	3.3257572	0.5007424	0.8596225
Prex1	-0.0883092	7.3971291	0.5007754	0.8596225
Trip12	-0.0821993	8.0919437	0.5007853	0.8596225
Trim41	-0.09209	5.492968	0.5007914	0.8596225
Gm40476	-0.2938155	0.7021038	0.5007989	0.8596225
Ip6k3	-0.3940856	0.8133034	0.500809	0.8596225
Gm12678	0.6432972	-0.5737868	0.5008293	0.8596225
Mfng	0.112245	3.5690785	0.5009271	0.8596306
Rab13	0.1443553	3.4272474	0.5009949	0.8596306
Exoc2	-0.0962027	5.9381402	0.5009998	0.8596306
Uqcr10	0.1019867	6.3233311	0.5011587	0.8597785
Atp6v0a1	0.0874963	6.5733632	0.5012296	0.8597785
Mafb	0.0841828	5.9711169	0.5012519	0.8597785
Cst7	-0.127718	4.9303513	0.5013499	0.8598519
Hcst	-0.2090336	2.8386165	0.5015127	0.8599906

Zbtb33	-0.0986304	5.3711452	0.5015414	0.8599906
Slc38a6	0.1453394	3.9292046	0.5018749	0.8601231
Ccp110	0.1153608	6.0024911	0.5019099	0.8601231
Gm31504	0.4051984	-0.9818028	0.5019322	0.8601231
Morf4l1-ps1	0.1971218	1.9363351	0.5020051	0.8601231
1700123O20Rik	-0.0970011	5.3159363	0.5020287	0.8601231
Gm34081	-0.5234194	-0.958765	0.5020495	0.8601231
Cox7a2l	0.0973723	6.8332673	0.5020685	0.8601231
Ndufv2	0.1288211	6.9383347	0.5020726	0.8601231
Srp14	0.0921676	6.2425604	0.5021527	0.8601231
D630045J12Rik	-0.2468034	1.7058938	0.5021717	0.8601231
Snapc1	0.106691	4.0071163	0.5023199	0.8602823
Ighmbp2	0.1649433	2.7746279	0.5024136	0.860348
Gm52824	0.3311542	-0.4883111	0.5025343	0.86046
Cmbl	-0.1944407	2.1008473	0.5027813	0.8607577
Pdzd11	0.0959327	5.3578246	0.5028189	0.8607577
Rangrf	0.1653013	2.9276833	0.5030146	0.8609908
Mrps27	0.122726	3.5414602	0.50313	0.8609908
Snhg18	0.2763403	3.7281187	0.5032092	0.8609908
Dgka	0.0953705	5.5219015	0.5032738	0.8609908
Etfdh	-0.0901781	5.5563383	0.5032911	0.8609908
Nsrp1	-0.0829016	5.0786528	0.5033582	0.8609908
Rpl22	0.1249606	9.0610157	0.5034224	0.8609908
Spag9	-0.0764455	6.8975929	0.5034495	0.8609908
Reep2	-0.1525465	2.8299883	0.5035339	0.8609908
Fmr1	-0.0753966	6.9266488	0.5035457	0.8609908
Micu3	-0.2225026	1.5813872	0.5036232	0.8609908
Zbtb11	-0.0782196	5.4114133	0.5036348	0.8609908
Spryd4	0.1451207	3.169313	0.5036746	0.8609908
Gm30914	-0.4648118	-1.1443987	0.5038521	0.8611994
Mfsd2b	0.1057433	7.1494532	0.5039975	0.8613534
Shisa4	0.3123425	1.313601	0.5040902	0.8614172
Ccdc158	0.3710237	-0.9270384	0.5041889	0.8614912
Engase	0.1058468	3.8929159	0.5043036	0.8615926
4632415L05Rik	-0.1073592	3.6137922	0.5045312	0.8618867
Wdhd1	0.1148034	7.0679302	0.5046433	0.8619096
Luc7l2	0.0759073	8.6230209	0.5046668	0.8619096
Rbmx2	0.1334987	3.9836769	0.5047109	0.8619096
Msl3	-0.0711569	6.1985379	0.5049027	0.8621426
Gdf3	-0.3278016	1.585954	0.5049895	0.8621962
Ptn	0.4225572	2.2024803	0.505114	0.8622167
Mfsd5	0.0973193	4.5309286	0.5052226	0.8622167
Macrod2	-0.2267833	1.564175	0.5052355	0.8622167

Pxdn	-0.1377179	3.4807729	0.505275	0.8622167
Cercam	0.113221	4.7609144	0.5052791	0.8622167
Egln1	0.0796974	5.2435258	0.5053342	0.8622167
Enox1	0.3705169	0.0544743	0.5054137	0.8622579
Rsrc1	-0.0844208	5.1215617	0.5054822	0.8622801
Vcam1	-0.1107937	8.4457928	0.5056101	0.8624037
Paqr8	-0.1821544	1.8377571	0.5057777	0.862595
Slc2a1	-0.1268172	3.8752708	0.505894	0.8626987
Ube2z	0.0695597	6.2963009	0.5059989	0.8626987
Ube4a	-0.0722789	6.577211	0.5060985	0.8626987
Cracr2b	-0.1366696	2.687948	0.5062021	0.8626987
0610040J01Rik	0.2009624	1.250567	0.5062068	0.8626987
Gm8130	-0.4481494	0.6529312	0.5062796	0.8626987
Ly6c1	-0.1814386	2.445827	0.5063078	0.8626987
4930445K14Rik	0.2190272	1.2764263	0.5063154	0.8626987
Mrpl32	0.1261966	4.8826115	0.5064451	0.8626987
Tmem45a	0.2973961	2.422439	0.5064472	0.8626987
Vps4b	-0.0712229	6.715423	0.5064486	0.8626987
Pik3ca	-0.080003	6.4383031	0.50656	0.8627464
Uba52	0.0862338	9.4751424	0.5066783	0.8627464
Rslcan18	-0.2647462	0.8525207	0.5066907	0.8627464
Coq10a	0.119633	3.202253	0.506713	0.8627464
Bcan	0.4544871	-0.4033752	0.506754	0.8627464
Eva1a	0.2170915	1.2859314	0.5068896	0.8628829
BC064078	-0.1919731	2.1828888	0.5069774	0.8629378
Gpr137b-ps	-0.1192402	4.9336713	0.5070978	0.862939
Dyrk2	0.1091309	5.8280477	0.5073154	0.862939
Aldh7a1	0.1038738	3.9191755	0.5073252	0.862939
Slc24a5	-0.1443167	4.0184072	0.5073825	0.862939
C030034L19Rik	-0.2985318	-0.3501919	0.5074153	0.862939
Ssr3	-0.078935	8.1423553	0.5074753	0.862939
Gm7331	0.2011129	1.8002674	0.5074855	0.862939
Gm20008	0.1623463	2.071193	0.5074905	0.862939
Plekhm1	-0.0910248	5.9916489	0.5075503	0.862939
Chst10	0.1803958	3.8606169	0.507584	0.862939
Foxc1	-0.1391101	4.8317412	0.5075883	0.862939
Ctsf	0.1485594	4.1427944	0.5076508	0.8629508
Mapk3	-0.0785525	7.0287879	0.5078111	0.863129
Mepce	-0.0806755	5.765919	0.5080635	0.8632593
Gbp5	0.2155576	1.955939	0.5080688	0.8632593
Jmjd8	0.1072768	4.0326018	0.5081566	0.8632593
Cacna1i	0.3144944	2.257629	0.5081643	0.8632593
Micos13	0.1008081	5.2299334	0.5081652	0.8632593

Zfp128	-0.1499993	2.1102463	0.5082625	0.8632756
Armcx4	-0.1476931	3.8034133	0.5082859	0.8632756
B3gnt9	0.1781026	3.6448494	0.5087284	0.863933
Mocs2	-0.1138452	4.7299535	0.5089388	0.8640756
Hbb-bs	0.2252277	14.559227	0.5089528	0.8640756
St7	0.1110978	4.4844389	0.5089934	0.8640756
2300009A05Rik	-0.1880262	1.9874825	0.5090544	0.8640756
Vmp1	-0.0874554	6.1338435	0.5092011	0.8640756
COX1	0.1109225	13.18063	0.5092141	0.8640756
Inafm2	-0.1027286	6.1815236	0.509321	0.8640756
Cdk5rap1	-0.2668402	3.7579181	0.5093575	0.8640756
Tmem107	0.1710708	2.4139342	0.5094394	0.8640756
Dipk1c	-0.3940722	-0.0726806	0.5094414	0.8640756
Scamp2	-0.0962073	6.1670607	0.5095259	0.8640756
Gm9833	-0.2080013	2.6360172	0.509589	0.8640756
Zfp691	0.1378606	2.9080737	0.5095902	0.8640756
Csnk2a3	-0.2711633	1.0341103	0.5095952	0.8640756
Cct4	0.0754061	7.5396342	0.5096457	0.8640756
Igkv7-33	-0.58512	-0.2922738	0.5097337	0.8641306
Scarf2	0.2154259	4.2874447	0.5097936	0.864138
Man2b2	-0.086997	5.7959787	0.5100514	0.8643772
Fam219b	0.1114767	4.2441484	0.510089	0.8643772
Fam83a	-0.4093738	0.1682251	0.5101015	0.8643772
Gm3230	0.4813119	-0.8756866	0.5103324	0.8646077
Rpl5	0.0931119	9.7893427	0.5104285	0.8646077
Psmd13	0.0886797	6.3765122	0.5104965	0.8646077
Rfwd3	0.0789675	7.2901041	0.5105007	0.8646077
Pisd-ps2	0.4181512	-0.3804487	0.5105462	0.8646077
Tmem42	0.1665013	2.1103366	0.5105887	0.8646077
C2cd3	0.110322	5.745667	0.5107142	0.8646077
Washc5	-0.0985899	5.5345776	0.5108147	0.8646077
Skp2	0.0901233	5.1044386	0.5108433	0.8646077
Bicra	0.1244687	5.1453591	0.5108823	0.8646077
Col22a1	-0.2174939	6.7199491	0.5109009	0.8646077
Lpar1	-0.2130156	3.4982319	0.511	0.8646077
Clba1	-0.2580692	1.9644486	0.5110491	0.8646077
Mycbp	0.0981782	4.4364021	0.5111139	0.8646077
Galnt4	-0.087023	4.5669908	0.5111737	0.8646077
Nfkbid	-0.1489098	3.7122003	0.5112277	0.8646077
Gm15910	-0.4320141	-0.6621722	0.5112787	0.8646077
Mphosph10	-0.0845962	5.7080502	0.5114027	0.8646077
Hbb-bt	0.2028503	11.242328	0.5114578	0.8646077
Mki67	0.1135332	11.1396	0.5114711	0.8646077

Cdca7l	-0.1035076	5.2407911	0.5115566	0.8646077
Zfp65	-0.0994258	4.0298261	0.5116517	0.8646077
Apex1	0.0835285	5.1189668	0.5116664	0.8646077
H1f2	-0.1174213	4.8934818	0.5117123	0.8646077
Septin6	0.1022961	5.6707784	0.5117295	0.8646077
Lemd3	-0.0884388	5.0976913	0.5117631	0.8646077
Bmp1	0.2039324	6.5543734	0.5118459	0.8646077
Cr1l	-0.0701038	6.1488027	0.5120245	0.8646077
Pola1	-0.0976958	6.7884838	0.5120347	0.8646077
Vps13d	-0.0831576	6.3740505	0.512049	0.8646077
Ing5	-0.0887957	5.2607778	0.5120581	0.8646077
LOC118568202	-0.336808	-0.3375322	0.5121032	0.8646077
Hsf2	0.1590146	3.2659285	0.5121105	0.8646077
Col4a5	-0.4001916	-0.9473517	0.5121274	0.8646077
Fblim1	-0.1582622	4.1227937	0.5121944	0.8646268
Sh3glb1	0.0900856	8.2740327	0.5123531	0.8647668
Relch	-0.0895635	5.2957276	0.512427	0.8647668
Fkbp10	0.2018963	5.7129146	0.5125424	0.8647668
Gm16116	-0.2801443	0.3575547	0.5125547	0.8647668
Ffar1	-0.4430769	-0.9940045	0.5125822	0.8647668
Fam189a2	0.2768736	0.8094745	0.5126109	0.8647668
Cnot8	0.0679651	6.0310607	0.5128764	0.8650774
Yod1	-0.1391061	4.4912567	0.5129356	0.8650774
Spta1	0.1491455	9.0074002	0.5129693	0.8650774
Hmcn1	-0.2969789	0.9830768	0.5130175	0.8650774
Spice1	-0.1052546	3.8220832	0.5132303	0.86525
Dgkz	-0.0869833	7.2509504	0.5133613	0.86525
Gm32633	0.2688116	0.5012362	0.513365	0.86525
Med29	0.107868	3.5573897	0.5133659	0.86525
Asns	0.1254205	6.200207	0.513398	0.86525
Sec61a2	0.1237418	3.7750474	0.5134933	0.8653169
Strap	0.0770706	7.0043746	0.5136148	0.8653232
Susd2	0.2175306	2.3560991	0.5136391	0.8653232
Phka2	-0.1016711	5.602634	0.51376	0.8653232
Mmut	-0.0878456	5.0020967	0.5137669	0.8653232
Ogn	-0.167314	4.744484	0.5137752	0.8653232
Arid5a	0.1341388	4.9794862	0.5138911	0.8654238
Glyctk	0.2021399	1.2400482	0.5139632	0.8654238
Fubp1	0.0767048	7.378565	0.5140018	0.8654238
Klhl22	0.1290821	3.9922707	0.5142165	0.8656915
Qk	-0.0718508	7.8190848	0.5143842	0.8656952
Leng1	0.1168544	3.6258295	0.514422	0.8656952
Cyb561d2	0.1431945	2.6793682	0.5145127	0.8656952

Cibar1	0.1454549	3.8189943	0.5145452	0.8656952
Wdpcp	-0.2128002	1.7946507	0.5145472	0.8656952
Pnma1	0.3109638	1.3456908	0.5145526	0.8656952
Slc39a13	0.1564859	4.7838613	0.5147149	0.865814
Cox10	0.126473	3.2831342	0.514781	0.865814
5830418P13Rik	-0.311983	-0.4087722	0.5147903	0.865814
4931408C20Rik	-0.5442487	1.3633582	0.5148962	0.8658985
3300002I08Rik	0.1457732	3.0167892	0.5151006	0.8659706
Ttc32	0.1376381	3.7917204	0.5151043	0.8659706
Capn7	0.0685946	6.1160744	0.5151233	0.8659706
Cenpb	0.0779974	6.6922632	0.515236	0.8659706
Klrb1c	0.2012082	0.9304848	0.5152445	0.8659706
Spaar	-0.3133688	-0.0347706	0.5152731	0.8659706
Zfp560	0.1749908	2.0858102	0.5154556	0.8661237
Sf3b4	0.1142418	5.4019589	0.5154756	0.8661237
Cdpf1	0.2005549	2.3331965	0.5156127	0.8662605
Foxred1	-0.0883088	4.3486108	0.5157781	0.8664448
Mrps18b	0.1371336	3.5045386	0.5159753	0.8665474
Fgfr3-ps	0.308501	-0.9397119	0.5161742	0.8665474
Mid2	0.1418326	3.1831579	0.5162052	0.8665474
Serpine1	0.3184909	2.8172321	0.5162388	0.8665474
1700055D18Rik	0.4290471	-0.750885	0.5163116	0.8665474
Ogdh	0.0845534	7.8127742	0.5164196	0.8665474
Gm32401	-0.2791016	-0.5163943	0.5164219	0.8665474
Mapre1	-0.0715331	7.8475161	0.5164353	0.8665474
Ythdc1	0.0793924	6.9884167	0.5164886	0.8665474
Tbc1d4	0.1078654	3.8473451	0.5165208	0.8665474
Lypla2	0.0826085	5.793738	0.5165783	0.8665474
Ccng2	-0.1014141	6.1809809	0.516588	0.8665474
Ang	0.4958763	0.4552372	0.5165993	0.8665474
Xist	-0.1038122	10.318453	0.5166191	0.8665474
Cenpc1	-0.0760924	5.8264753	0.5166894	0.8665719
Itga3	-0.2726722	1.1496924	0.5168136	0.8666259
Tecpr1	-0.1017689	4.0189136	0.5168331	0.8666259
Sptb	0.1430862	8.8154106	0.5171619	0.8670002
Rtl5	0.1802579	2.9779233	0.5171699	0.8670002
Sys1	0.0888019	4.7194224	0.5172235	0.8670002
Ubxn6	0.0840543	5.1746534	0.5173212	0.8670704
Elmod2	-0.0874085	4.8751216	0.5174231	0.8671478
Gm44705	0.2927718	0.3528001	0.5174942	0.8671672
Tmem223	0.1131467	4.0236043	0.5175462	0.8671672
Mrpl23	0.10596	3.9356595	0.5177458	0.8673732
Tmem126a	0.1348311	3.3647014	0.5178279	0.8673732

Primpol	0.1426211	4.7321916	0.5178364	0.8673732
Gtf2h5	0.1161606	5.3348735	0.5179227	0.8674103
Osbpl10	0.3268368	-0.3398175	0.5179701	0.8674103
Aspn	-0.4821048	2.8186952	0.5180652	0.8674762
Ccar2	0.0847471	5.6100655	0.5181566	0.8675358
Jpt2	-0.0906457	5.4528998	0.5182721	0.8676206
Slc12a9	0.1344178	3.6435677	0.5183605	0.8676206
Atp5o	0.0778133	7.3760766	0.5183746	0.8676206
Gm5503	-0.3729373	0.7615693	0.5184435	0.8676426
Sestd1	-0.1051641	3.5070645	0.5186214	0.8678469
Lrp10	0.0665878	6.5686926	0.518735	0.867856
Zfp607b	0.2645554	0.539998	0.5187384	0.867856
Nlrc3	0.2164624	2.0714574	0.5188003	0.8678656
Lin9	0.1079007	4.8939826	0.5189083	0.8678656
Creg1	-0.1015862	8.0159399	0.5189116	0.8678656
Ahcyl2	-0.0903694	5.0014252	0.5190971	0.8679255
Zfp809	-0.0939992	3.8623881	0.5191324	0.8679255
Herc6	-0.1258291	4.875722	0.5191647	0.8679255
Rai2	-0.1586231	1.7275527	0.5191705	0.8679255
Kif1a	-0.271576	1.1907544	0.5192924	0.8679368
Atg4a	0.1407541	6.2501551	0.5193126	0.8679368
Atf1	0.0741237	6.9366	0.5194863	0.8679368
Uba1	-0.0973751	8.0781858	0.5195191	0.8679368
Atp5h	0.0939561	7.8357118	0.5195643	0.8679368
Kat8	0.1062307	3.9555675	0.5195836	0.8679368
Hspb8	-0.3603768	-0.1507686	0.5196169	0.8679368
Borcs8	0.0852302	4.8793177	0.5196544	0.8679368
Slc35b2	0.0849402	5.0833763	0.5196966	0.8679368
Slc10a7	0.0830449	4.6555284	0.5197353	0.8679368
Ankrd26	0.0950313	4.5040909	0.519872	0.8680719
Tnfrsf9	0.2675276	1.1662955	0.5200222	0.8681791
Gm52009	0.3211658	-0.6042743	0.5200629	0.8681791
Rad21	0.085184	8.8795825	0.5201487	0.8681791
Nudt5	0.0777845	5.1970318	0.5201595	0.8681791
Cyp39a1	-0.1457732	2.5224109	0.5202782	0.8682693
Pdgfd	-0.1925343	4.1115513	0.5203252	0.8682693
Ctsl	-0.0847541	6.6533819	0.5204529	0.8683046
Myadm	-0.0875238	6.4980875	0.5205046	0.8683046
Nt5c3	0.1333528	7.5415712	0.5205138	0.8683046
Gm40892	-0.2656321	2.4870995	0.5206578	0.8684517
Mettl6	0.0793988	5.2738191	0.520718	0.8684589
Recql5	0.0901839	4.2468366	0.5208304	0.8685321
Nrde2	0.1239259	3.9227039	0.5208736	0.8685321

Psma1	0.091902	7.1592035	0.5209968	0.8686445
Gm46901	-0.3337133	0.3627282	0.5211904	0.8688574
Atg4d	0.1106933	5.4699007	0.5212803	0.8688574
Card9	-0.1044754	5.2896676	0.5213507	0.8688574
Trmt9b	-0.2495657	0.6492528	0.5213931	0.8688574
Klhl8	-0.214182	1.5761319	0.5214113	0.8688574
Ppp4r1	-0.0934157	5.9931773	0.5214733	0.8688574
4930520E11Rik	-0.3195289	-0.9000721	0.5215236	0.8688574
Smarcc1	0.0891098	7.0586427	0.5215747	0.8688574
Psmd10	0.0843736	4.8491679	0.5217031	0.8688574
Zer1	-0.0974061	4.699273	0.5217705	0.8688574
Speg	-0.1884485	3.0339342	0.5217768	0.8688574
C2cd5	0.0895519	6.1473382	0.5218387	0.8688574
Coprs	0.2294882	1.2272515	0.521881	0.8688574
Tmem203	-0.1264075	2.8376523	0.5219989	0.8688574
Wbp1	0.0826675	5.1093657	0.5220098	0.8688574
Prkab2	-0.1284937	4.2710022	0.5220183	0.8688574
Rft1	-0.1443489	3.1282867	0.5220818	0.8688701
Fam174a	0.1151988	4.316532	0.5221452	0.8688826
Rspo2	-0.2050906	2.6295463	0.5223388	0.8689688
Abca3	0.0799173	6.2276951	0.5224357	0.8689688
Fkbp11	0.2174535	2.8520368	0.5224501	0.8689688
Kcng1	-0.3658555	-0.3726257	0.5225499	0.8689688
Txndc5	0.0841516	7.0954653	0.5225768	0.8689688
1110004F10Rik	0.0724469	6.2869939	0.5227462	0.8689688
Cd1d1	0.1333237	4.612586	0.5227661	0.8689688
Snx30	0.1034526	5.4099746	0.5227969	0.8689688
Gm20554	-0.3855779	-0.8537389	0.5228084	0.8689688
Acad9	-0.0983586	4.6840755	0.5228306	0.8689688
Zfp707	0.1686199	1.944222	0.522875	0.8689688
Aldh4a1	-0.1020728	4.0799503	0.5229196	0.8689688
Cwc15	0.0663446	6.4916247	0.5229232	0.8689688
Rfx2	0.105123	4.5534754	0.5230664	0.8691138
Gm47754	-0.2406333	0.7723576	0.5232384	0.8693068
Limk1	-0.10446	3.9219703	0.523355	0.8694077
Smim8	0.1722277	3.703309	0.5234201	0.869423
Zfyve26	-0.0900324	5.0402877	0.5235066	0.8694738
Zbtb5	0.0932266	4.2507144	0.5238317	0.8699084
Cep68	-0.1045441	4.6256709	0.5238802	0.8699084
Mllt3	0.1248926	5.6777673	0.5239674	0.8699603
Trp53rkb	0.184906	1.6490866	0.5240895	0.8700583
I730030J21Rik	-0.2333396	0.2057413	0.5241637	0.8700583
Zfp64	0.1172483	3.264729	0.5241942	0.8700583

Gdpd5	0.2699507	1.1014123	0.5243186	0.8701719
Nsun5	0.1129625	3.5314805	0.5246092	0.8703179
Dnajc6	-0.1179234	3.815601	0.5246269	0.8703179
Prx	-0.1896537	1.4332616	0.5246758	0.8703179
Bcorl1	-0.0996312	5.366668	0.5246818	0.8703179
2610300M13Rik	0.3487393	1.6272682	0.5246863	0.8703179
Farp2	-0.2330029	3.7865185	0.5247815	0.870383
Tceal9	0.1107104	6.2168386	0.5248962	0.8704331
Fibp	0.1004016	4.2407191	0.5249358	0.8704331
LOC118567423	0.2675474	0.9170022	0.5249796	0.8704331
Erich5	-0.5050022	-0.6617319	0.525057	0.8704686
Dbt	-0.0907035	3.9293349	0.5252857	0.8706967
Klhl21	0.1044374	3.9709222	0.5253119	0.8706967
Tpp2	-0.0730196	6.453792	0.5254042	0.8706967
Xpnpep1	0.0947683	6.5778566	0.5254185	0.8706967
Pias3	-0.0999789	4.4873031	0.5255134	0.8707029
Plpp7	0.1881272	1.1840065	0.5255342	0.8707029
Gm13414	0.398454	-0.9255373	0.5256721	0.8707784
Rsph3b	-0.1920618	2.5971628	0.5256917	0.8707784
Oas1e	0.3130533	-0.8691236	0.5258555	0.8708815
Hcfc1r1	0.1002912	4.933661	0.5258723	0.8708815
Rftn2	-0.1969916	3.7603303	0.5260028	0.8708815
Cd86	-0.1241844	3.6692293	0.526015	0.8708815
Lif	-0.3028189	1.1516769	0.5260339	0.8708815
Polr2j	0.0763325	5.8361922	0.5261754	0.871023
Ptpmt1	0.1349709	3.6810304	0.5262393	0.8710361
Trim3	-0.1505399	3.0035986	0.5262959	0.8710371
Lamb2	-0.1807522	4.8838252	0.526467	0.8712275
Agbl3	0.1769286	1.761092	0.5266427	0.8713543
Btnl7-ps	-0.2727221	-0.3049454	0.5267422	0.8713543
Map1s	0.0997151	4.5024779	0.5267776	0.8713543
1700056E22Rik	-0.2625315	-0.3821399	0.5267903	0.8713543
Gm14438	0.2614321	-0.5770456	0.5268666	0.8713543
Fbxo10	-0.1077432	4.4271545	0.5268797	0.8713543
Fkrp	0.0954175	4.530516	0.5270161	0.8713574
Glis2	0.207426	2.9989765	0.5270472	0.8713574
Kctd1	-0.161562	3.2200407	0.5270497	0.8713574
Serac1	0.1392411	2.628625	0.5272156	0.8713852
Tmem167	-0.0675904	6.2530983	0.527228	0.8713852
Fam214a	0.1198526	4.5058963	0.5272346	0.8713852
Exoc5	0.0673717	6.2977481	0.5273986	0.8713852
Fam122b	0.0977811	4.4206512	0.527412	0.8713852
Mipol1	0.308133	0.5355909	0.5274474	0.8713852

Oat	0.065749	6.5405118	0.5274586	0.8713852
Sema3g	0.1650503	2.8050829	0.5275198	0.8713936
Cyp2ab1	-0.4491254	-0.5131822	0.5276812	0.8715677
Mtarc1	0.2667211	1.9475496	0.5278219	0.8717075
Zfp563	-0.1632583	2.5553154	0.5278829	0.8717158
Efnb2	-0.1936818	2.8318472	0.528044	0.8717791
Kdm2b	0.1032759	5.3629863	0.5280986	0.8717791
Tufm	0.0905218	5.2610146	0.5281947	0.8717791
Sap130	0.0870221	5.4538555	0.528245	0.8717791
Ppp1r9b	0.0712908	7.7740151	0.5283301	0.8717791
Galnt11	-0.1074374	3.3748495	0.528385	0.8717791
Bbs9	-0.1484571	3.4050901	0.5284515	0.8717791
Rassf7	0.1749028	2.6379739	0.5284656	0.8717791
Pqbp1	0.0778596	5.6526325	0.5285012	0.8717791
Cwc25	-0.0808699	4.6298393	0.5285341	0.8717791
Ubox5	0.2076284	2.3349302	0.5285378	0.8717791
Men1	0.0865619	5.1369532	0.5286512	0.8718736
Dcun1d1	0.0934287	6.8538522	0.5287245	0.8719021
Aasdhppt	0.0960554	4.7569927	0.5289668	0.8721713
Armcx5	-0.13552	2.7797286	0.5289999	0.8721713
Gm7592	-0.288198	-0.0135729	0.529094	0.872234
Wasf2	-0.1107167	7.7923088	0.5291731	0.872272
Smim13	0.0843269	4.6589955	0.5293441	0.8724615
Chaf1a	0.0835584	6.5008208	0.5295075	0.8726382
F7	-0.2875718	-0.8466386	0.5298798	0.8729749
Mterf1a	0.1659114	2.8535502	0.529925	0.8729749
Lipc	-0.2176261	4.1664732	0.5299271	0.8729749
Prune1	-0.089236	4.9298607	0.5300033	0.8729749
Cenpj	-0.0935549	4.7277399	0.5300685	0.8729749
Sacm1l	-0.0691954	6.2860614	0.5301438	0.8729749
Myorg	-0.2528293	1.4358005	0.530295	0.8729749
Spopl	-0.0865706	4.9727182	0.5303084	0.8729749
Gspt2	0.2267127	1.1170953	0.5304503	0.8729749
Erap1	-0.0850921	6.0111427	0.5305017	0.8729749
Zhx1	-0.0810425	6.3383316	0.5305182	0.8729749
Pdgfb	-0.1272546	4.167473	0.5305564	0.8729749
Cryba4	0.3496293	-0.5170661	0.5305643	0.8729749
Kansl2-ps	-0.2878177	-0.7438081	0.5306029	0.8729749
Nsmce2	0.0835883	4.6864985	0.5306276	0.8729749
Jade3	0.0985977	4.195554	0.5306467	0.8729749
Tns3	-0.0943194	6.9661194	0.5306659	0.8729749
Ltbp3	-0.1384865	5.2529648	0.5308075	0.8730514
Fh1	0.0692493	5.8950922	0.5308247	0.8730514

Hexa	-0.0984482	7.7544275	0.530963	0.8731866
Ss18l2	0.1482675	3.4332231	0.5312617	0.8733954
Exosc10	0.0866054	5.6791693	0.531329	0.8733954
Blmh	0.0714393	5.9286669	0.5314328	0.8733954
Slc17a9	-0.1335669	3.4330931	0.5314815	0.8733954
Srek1	0.068388	6.2691736	0.5315112	0.8733954
Cbwd1	-0.1203587	3.4671939	0.5316103	0.8733954
Jade2	-0.1080748	4.5066448	0.5316672	0.8733954
A330094K24Rik	0.7001386	-0.9247548	0.5317815	0.8733954
Pimreg	0.134281	3.369418	0.5318465	0.8733954
Hp1bp3	-0.0728255	7.9200415	0.5318531	0.8733954
Asl	-0.1060186	3.6891963	0.5319161	0.8733954
Pak1	-0.0989422	5.4071314	0.5319454	0.8733954
Ociad2	-0.2438502	0.6112978	0.5320026	0.8733954
Tesc	-0.1988263	3.2273212	0.5320949	0.8733954
Ypel2	-0.0981471	4.1778625	0.5321473	0.8733954
Fkbpl	-0.1346514	2.6619469	0.5321765	0.8733954
Copb2	-0.0719505	7.5227858	0.5321858	0.8733954
Ndufaf5	-0.1329205	2.6217477	0.5322133	0.8733954
Mcoln1	0.0930352	4.2861032	0.5323435	0.8733954
Cdk5	0.1068434	3.8457835	0.5323439	0.8733954
Polr1e	0.1056531	3.3700052	0.5323694	0.8733954
Atxn10	-0.0829899	6.7001522	0.5324379	0.8733954
Ptchd1	0.3474858	0.3671602	0.5324796	0.8733954
Aff1	-0.0978875	7.6406848	0.5325699	0.8733954
LOC118568438	-0.2547247	0.6086858	0.5325737	0.8733954
Rmnd1	0.1041743	4.1845183	0.5325987	0.8733954
Mrps12	0.0919566	4.2692058	0.5326061	0.8733954
Rab14	-0.0713458	7.504111	0.5327169	0.873485
Gpr88	0.1921936	2.3784233	0.5329179	0.8735379
Hspe1	0.1205854	6.5882502	0.5330208	0.8735379
Gpi-ps	0.187124	1.5755087	0.5330553	0.8735379
Calm1	-0.0760302	8.7933218	0.533121	0.8735379
1700007L15Rik	-0.2348056	0.0381821	0.5331283	0.8735379
Col4a2	-0.1120053	6.248467	0.5331708	0.8735379
Med17	0.0714068	5.486377	0.533174	0.8735379
Hscb	0.1285715	4.4780593	0.5331984	0.8735379
Bcap31	-0.0672855	6.4716496	0.5332997	0.8735968
Psd4	-0.0964053	6.5150645	0.5333762	0.8735968
Ncf2-rs	-0.4983962	2.8340623	0.5336084	0.8735968
Dand5	0.1682106	1.9253748	0.5336418	0.8735968
Mvk	-0.1417036	2.4209602	0.5338219	0.8735968
Pcbp1	0.0763601	8.2849658	0.5338288	0.8735968

Trappc2l	-0.0946099	4.5997497	0.5338947	0.8735968
Med30	0.1139953	4.9498146	0.5339723	0.8735968
Supt3	-0.1020625	3.3115057	0.5341356	0.8735968
Mfsd9	-0.1555257	1.9469412	0.5342228	0.8735968
Klf13	0.0954954	7.0221358	0.5342811	0.8735968
Katna1	0.0715881	5.291068	0.5343197	0.8735968
Gm11451	0.1823484	1.228516	0.5344306	0.8735968
6330415G19Rik	0.3361617	-0.6204611	0.5344646	0.8735968
Nkiras1	0.1400237	2.5984664	0.5344655	0.8735968
Mlip	-0.2249058	3.6422746	0.5344721	0.8735968
Il16	-0.1011007	6.757145	0.5345511	0.8735968
Zfp385c	-0.305344	-0.6503245	0.5346091	0.8735968
Ranbp9	-0.0787073	6.3216496	0.5346746	0.8735968
Atp6v1e1	0.0830429	6.9648928	0.5347398	0.8735968
Rnf115	-0.0690507	6.2589527	0.5347661	0.8735968
Ppfibp2	0.1143097	3.8311801	0.5348998	0.8735968
Gm12350	-0.2544267	-0.0130659	0.534919	0.8735968
Zdhhc5	-0.0815948	6.6327439	0.5349322	0.8735968
Coq9	0.093556	4.5526971	0.5350154	0.8735968
Ubl5	0.0731744	6.9123375	0.5351174	0.8735968
Gm6012	0.3278208	0.7625319	0.5351236	0.8735968
Sec14l5	-0.1942093	2.5276204	0.5351434	0.8735968
Zfp512	0.0874398	4.6384295	0.5351639	0.8735968
Efna2	0.3059821	0.3688966	0.5351721	0.8735968
Snx10	-0.1087469	6.4476877	0.5352169	0.8735968
Timmdc1	0.0972048	3.8746554	0.53524	0.8735968
Gm9333	-0.2127544	0.6847049	0.5352598	0.8735968
Klf4	0.1045349	5.1275598	0.5352917	0.8735968
Cd96	-0.2503592	2.4086432	0.5353061	0.8735968
Abca8a	-0.1436796	3.6599003	0.5353157	0.8735968
Rps10-ps1	0.1804031	0.9421145	0.5354095	0.8735968
Lipt2	0.2037574	1.3111544	0.5354825	0.8735968
Slc25a23	0.1360181	3.3792286	0.5355182	0.8735968
Slc6a12	-0.2101817	1.144364	0.5355354	0.8735968
Wbp2	-0.0668694	6.7324029	0.5355372	0.8735968
Kif14	0.1011544	6.1433655	0.5358028	0.8736701
Gm10221	0.2415163	0.8107947	0.5358232	0.8736701
Cryzl1	-0.0841986	4.7265954	0.5358421	0.8736701
Ptpn6	-0.0825431	7.7402976	0.5358845	0.8736701
Rwdd3	0.3047316	0.9573122	0.5358929	0.8736701
Tctex1d2	-0.1189906	4.6772689	0.5359192	0.8736701
Ppp2r2a	0.0792024	5.7428245	0.5360296	0.8737014
Mbtps1	0.0645242	6.5123399	0.5361001	0.8737014

Dars2	0.1050104	4.7745295	0.5361068	0.8737014
Trdmt1	-0.1143282	3.3470814	0.5362543	0.8737841
Ndufs6	0.0823203	5.9569712	0.5362776	0.8737841
Taf4b	-0.1957467	2.8546932	0.5364051	0.8737841
Zfp9	-0.2034171	2.713933	0.5364265	0.8737841
Adora2b	-0.2614467	1.2558428	0.5364385	0.8737841
Zfp870	-0.136403	2.609635	0.5367663	0.874221
Tbp	0.0882199	4.7351286	0.5368191	0.874221
Nxf2	0.3331801	-0.809649	0.5369701	0.8743753
Eif5a2	0.1412618	3.6750835	0.5371228	0.8744082
Rab1a	-0.0737087	7.579571	0.5371831	0.8744082
Spag7	0.0740443	5.6602531	0.5371999	0.8744082
Ddr1	0.1866316	2.5710157	0.5372152	0.8744082
Rnf25	0.0949175	4.0095638	0.5372847	0.8744298
Gm11423	0.2436889	0.7385717	0.5373688	0.8744753
Ttc30a1	-0.1851688	1.6088378	0.5375161	0.8746234
Mmab	0.1540428	2.9728327	0.5378366	0.8750534
Stk38	-0.0749218	7.153075	0.5379188	0.8750957
Adamts15	-0.2166606	1.537601	0.5380646	0.8752413
Pgf	0.2823573	1.3812235	0.5381301	0.8752563
Tsix	-0.2249407	1.6833042	0.5383259	0.875353
Cdr2	0.1478446	7.844483	0.5383323	0.875353
Rdh14	0.1223756	3.3665469	0.5383584	0.875353
Mmgt1	-0.0690536	5.3621165	0.5384996	0.875491
Gm14325	-0.1150757	3.6269629	0.5385686	0.8755118
Hat1	0.1052844	6.6309078	0.5387192	0.8756649
Camkmt	0.135009	2.8350788	0.5388214	0.8757396
Tpst1	0.112346	3.32545	0.5390302	0.8759874
Rnf20	-0.0623622	6.6418201	0.5391899	0.8760821
Haspin	0.097292	4.560986	0.5394713	0.8760821
Glrx3	0.0882646	6.2104571	0.5395274	0.8760821
Napepld	-0.1737586	2.254205	0.539537	0.8760821
Gm38250	-0.2990551	-0.0506151	0.5395673	0.8760821
Smarca1	0.2272425	2.4091352	0.5395818	0.8760821
Npepl1	0.074325	5.002431	0.5395957	0.8760821
Gm41569	0.3106108	-0.6574204	0.5396057	0.8760821
Appl2	0.1184399	4.6555558	0.5396207	0.8760821
Gm42354	0.3777215	-0.9521509	0.5396517	0.8760821
Dennd2a	-0.1339424	2.6520094	0.540027	0.8765999
Gm38639	0.2511682	0.410257	0.5401426	0.876696
Fam161b	0.243096	0.9910469	0.5402184	0.8767275
Mafg	-0.0753919	7.0377721	0.540361	0.8767737
Fut4	-0.1352458	3.2790894	0.5403766	0.8767737

Gm12481	-0.1933439	1.4816359	0.5404233	0.8767737
Polr3k	0.0882521	5.3881726	0.5404723	0.8767737
Flt1	-0.0962714	4.3293963	0.5410072	0.8775498
Snrnp25	0.1505454	4.3166567	0.5412001	0.8775884
Akr1c14	-0.2407495	1.6565199	0.541249	0.8775884
Vps54	-0.0675325	6.5128026	0.5412712	0.8775884
Tbc1d17	0.0792879	4.4735499	0.5412835	0.8775884
Slx4	0.1314367	5.2390952	0.5413558	0.8775884
C630043F03Rik	-0.2301872	0.209602	0.5413695	0.8775884
1500009L16Rik	0.155321	1.7590843	0.541457	0.8775997
Nudcd2	0.1057193	5.6175822	0.5414893	0.8775997
Shld3	-0.1517968	2.2199746	0.5416817	0.8777823
Os9	-0.0690035	7.4768067	0.5417148	0.8777823
Slc12a6	-0.0996339	7.3313653	0.5418186	0.8778061
Gss	0.1317828	3.6479943	0.5418424	0.8778061
Mnt	-0.0772484	4.9579746	0.5419848	0.8779453
Mdn1	-0.0850656	5.4794716	0.5421617	0.8781404
Pecr	-0.1488755	2.7113026	0.5422265	0.878154
Pcdh9	-0.2887952	1.0640574	0.5423805	0.878214
Ing4	0.0715677	5.3426436	0.5424189	0.878214
Upf3b	0.0821548	6.0666681	0.5425295	0.878214
Thrb	-0.1835522	1.9115167	0.5426682	0.878214
Fas	-0.1821986	2.1604682	0.5426732	0.878214
Ankrd50	-0.1369436	4.0287278	0.542693	0.878214
Bhlhb9	-0.0939827	3.6828626	0.5426966	0.878214
Sema4d	-0.0912912	7.0650717	0.5427477	0.878214
Tfpt	0.0928178	3.9315059	0.5428116	0.878214
Nit2	-0.1175026	3.3136036	0.5428282	0.878214
Hdgfl3	-0.1815598	1.766965	0.5428891	0.8782213
Siah2	0.1392342	3.0334538	0.5430322	0.8783614
Adck5	0.1381379	3.1866532	0.5432427	0.8786105
Rev1	-0.0735151	5.6556534	0.5434662	0.8788087
C6	0.1696479	2.0002774	0.5434782	0.8788087
Ccdc166	0.1605377	1.7072701	0.5436116	0.878933
Zfp105	0.2238807	0.960557	0.5437093	0.8789995
Card6	-0.1211408	4.4375492	0.5437898	0.8790382
Amt	-0.3203269	-0.3683807	0.5441765	0.8794817
Acbd3	-0.0797425	5.886757	0.5442145	0.8794817
Zfp783	-0.1721561	2.0686147	0.5443388	0.8794817
Napsa	-0.1176841	5.9981206	0.5443831	0.8794817
Senp6	-0.0631021	6.9983369	0.5444668	0.8794817
Slc2a9	-0.1478323	2.7194414	0.5445157	0.8794817
Zfp955b	0.0945614	3.7816168	0.5445159	0.8794817

Rdh11	0.0902044	4.5896042	0.544564	0.8794817
Rpl35a-ps2	0.2887581	1.2716292	0.5446559	0.8794817
Cd99l2	0.0852287	4.279896	0.5446853	0.8794817
Rpl19-ps11	0.1079672	4.1301282	0.5446939	0.8794817
Tubgcp2	0.0955007	5.3727027	0.5447426	0.8794817
Rhebl1	0.2904774	1.0713242	0.5449026	0.8795418
Tut4	0.1294515	5.7866709	0.5449126	0.8795418
Ercc3	-0.0891862	4.9136112	0.5449495	0.8795418
Mtif3	0.1007517	3.8158672	0.5450129	0.8795529
Bpnt2	-0.0658047	6.367696	0.5450958	0.8795955
Gm7901	0.3838825	-0.0796418	0.5454241	0.8797106
Enpp3	0.1388344	2.2542317	0.5454399	0.8797106
Naa38	-0.094874	4.7417823	0.5454954	0.8797106
Kat2b	-0.0746185	6.1807185	0.5455301	0.8797106
Ddx1	0.0796187	6.680008	0.5455526	0.8797106
Zfp628	0.0965974	3.8352345	0.545699	0.8797106
Abca7	-0.0749255	6.8645261	0.5457922	0.8797106
Pcmtd1	-0.0795903	5.8496759	0.5458262	0.8797106
Uap1	0.0797258	5.6557164	0.5458719	0.8797106
Tmtc2	-0.2069617	3.9871732	0.5458912	0.8797106
Fhl2	0.1431433	3.4996292	0.545936	0.8797106
Ephx4	0.532192	-0.9646166	0.5460041	0.8797106
Btn1a1	0.2353017	1.3401685	0.5460218	0.8797106
Sumf2	0.1188773	3.407426	0.5461186	0.8797106
Gpr15	-0.3054078	-0.3201972	0.5462114	0.8797106
Cyren	-0.0928095	4.2107478	0.5462202	0.8797106
Glg1	-0.0706562	7.0941724	0.546251	0.8797106
BC065397	-0.2874335	-0.1637943	0.5462798	0.8797106
Traf3ip1	0.1156005	3.2486344	0.5463512	0.8797106
Kcnab2	-0.0906546	6.1134283	0.5463673	0.8797106
LOC114841036	0.1683826	1.948277	0.5464008	0.8797106
Gabpa	-0.083634	6.5716846	0.5464912	0.8797106
1810030O07Rik	0.0742133	5.3197486	0.5465336	0.8797106
Rps27	0.1063466	9.3568579	0.5465782	0.8797106
Csf1	0.1084319	5.6263446	0.5465812	0.8797106
Patz1	0.0864209	5.1060646	0.5467812	0.8798351
Gata3	0.2740913	0.0044999	0.5468258	0.8798351
Pcdhb18	-0.3120369	-0.6877327	0.5468282	0.8798351
Cdc26	0.0884303	4.7716445	0.5469946	0.8798608
Etaa1os	-0.2221127	0.1580079	0.5470845	0.8798608
Pard6a	0.158046	1.9880651	0.5471928	0.8798608
Cdc16	-0.0753359	5.5304046	0.547232	0.8798608
Myo1g	-0.0996976	6.401893	0.547399	0.8798608

Nle1	0.1138383	3.2193183	0.5474285	0.8798608
Zbtb6	-0.0802951	5.3101	0.5474299	0.8798608
Traf7	-0.0737599	5.9894396	0.5474827	0.8798608
Ndufa6	-0.0909294	6.6764116	0.5475173	0.8798608
Serpinb8	0.1871221	1.6910312	0.5475334	0.8798608
Cd109	-0.2173281	6.1070888	0.547603	0.8798608
Gm2614	0.1715246	1.3829033	0.5476057	0.8798608
Sema4g	-0.2953079	0.5169813	0.5477923	0.8798608
Gapdh	-0.0788432	8.9597038	0.5478333	0.8798608
Gm8283	-0.300378	-0.9261775	0.5479555	0.8798608
D3Ertd751e	0.1089662	3.4873879	0.5479905	0.8798608
Gak	-0.0760379	6.6869765	0.5479923	0.8798608
Trabd	0.0748467	5.626492	0.5479938	0.8798608
Nuak1	-0.14359	4.717817	0.548006	0.8798608
Ogfod2	0.1112683	3.4430777	0.5480263	0.8798608
Ptpra	0.0608753	6.5885964	0.5480321	0.8798608
Pask	-0.0948634	4.4416712	0.5481831	0.8799931
Lyn	-0.0873319	7.7279681	0.5482277	0.8799931
Gm37201	0.2243895	1.2112122	0.5483624	0.8801186
Ubash3a	0.1119807	4.0850425	0.5486102	0.8801922
Arhgef12	-0.0928733	7.2078747	0.5486354	0.8801922
Gm33272	-0.1965768	0.8968757	0.5486671	0.8801922
Ccnt2	-0.0731833	5.9620569	0.5486759	0.8801922
Tango6	0.1330235	2.9382866	0.5486912	0.8801922
Crtac1	-0.2686461	0.8839271	0.5488821	0.880267
Frmd4a	0.0863345	6.5799613	0.5489414	0.880267
Pde8b	0.253465	0.5413102	0.5489462	0.880267
Rnase6	0.1303964	4.4240607	0.5489684	0.880267
Tbc1d16	-0.1441284	2.6485066	0.5492003	0.880267
Fkbp7	0.1917751	4.6930515	0.5492161	0.880267
Tfe3	-0.0699195	5.5021652	0.5492234	0.880267
Nup43	0.1207481	3.8371551	0.5492245	0.880267
Dph2	0.15673	2.7852151	0.5492472	0.880267
Ehf	-0.2967876	1.1678821	0.5493259	0.8802914
Ywhab	0.0694946	7.8163649	0.5494032	0.8802914
Gm17655	-0.1679957	1.242055	0.5494322	0.8802914
Zfp326	0.096713	4.167722	0.5495307	0.8803584
Vti1a	-0.0753747	5.2030437	0.5497901	0.8805444
Pus3	0.1089169	3.5595893	0.5498135	0.8805444
Gm33176	-0.2958669	-0.2878832	0.5498166	0.8805444
Ccdc68	0.2361426	1.3611449	0.5499214	0.8805577
Ppp1r1a	-0.4377147	-0.9998717	0.5500157	0.8805577
Gm6682	0.2260244	2.0826478	0.5500171	0.8805577

Asap3	0.3502213	-0.4084248	0.5500916	0.8805577
Hsp90ab1	0.0799785	10.274137	0.550108	0.8805577
Arhgap19	-0.0815298	7.2363004	0.5502409	0.8806046
Sh2b3	-0.0987883	6.4658078	0.5502825	0.8806046
Gm12854	-0.1966259	1.2422746	0.5503071	0.8806046
Gm2830	-0.2803002	1.2315122	0.550438	0.8806645
D7Ertd443e	-0.356971	-0.3939851	0.5504578	0.8806645
Rab34	0.1699227	2.5076992	0.5506498	0.8807793
Pfkfb2	-0.0762035	5.4523444	0.5506929	0.8807793
Gm14173	0.2128298	-0.2324812	0.5506994	0.8807793
Ky	0.3974927	-0.6443754	0.5508603	0.880946
Gm42059	-0.2860452	-0.5085273	0.5511332	0.8811833
Zfp316	0.1364054	2.1263187	0.5511363	0.8811833
Nme4	0.1410038	3.4839808	0.5511786	0.8811833
4833418N02Rik	0.2553563	0.2141955	0.5512369	0.8811859
Lama4	-0.14729	4.05204	0.5513099	0.8812069
Gimap3	-0.2083673	3.5850372	0.5514472	0.8812069
Gm9104	0.687999	0.7892113	0.5514537	0.8812069
Zcchc8	0.0666628	6.1322501	0.5514924	0.8812069
Dnaaf5	0.0974072	3.8094444	0.5515333	0.8812069
Cdkl3	0.1538624	1.664794	0.5516526	0.8812529
Zswim9	0.148007	2.134431	0.5516754	0.8812529
Qpctl	-0.1512379	2.4063126	0.5518462	0.8813285
Phf11d	-0.191766	1.525686	0.5518911	0.8813285
2700097O09Rik	0.1177557	3.4118976	0.5518927	0.8813285
Zfp810	0.1351773	3.3887033	0.5519591	0.881333
Marchf8	0.1110927	7.8014097	0.5520491	0.881333
Anapc15	-0.0868269	5.0704788	0.5520655	0.881333
Rab2a	-0.0744694	7.181367	0.552251	0.8814548
Ubqln1	0.0646394	6.9847127	0.5522552	0.8814548
Ccdc28a	0.1487721	2.6845962	0.5525239	0.8817932
Mst1r	0.1830621	3.6898048	0.552606	0.8818148
Agk	-0.108813	3.3375401	0.5526508	0.8818148
Rpa3	0.1072889	5.5780113	0.5527793	0.8818666
Fam133b	0.080091	5.3377818	0.5528663	0.8818666
Zrsr2	0.0706908	4.9774429	0.5528811	0.8818666
Crlf3	-0.0666539	7.1836246	0.5529101	0.8818666
C330018D20Rik	0.1227861	3.9309563	0.5530174	0.8819434
Mknk1	-0.0744201	5.7909126	0.5530716	0.8819434
Adap1	-0.1135377	4.3885622	0.5531523	0.8819816
Gm13213	-0.2776129	-0.5504568	0.5534503	0.8823168
Gm9396	-0.2385034	0.6762695	0.5534805	0.8823168
Tma7	-0.0753405	7.2078782	0.5535327	0.8823168

Snapc3	0.0882279	5.1026106	0.5538693	0.8826866
Gpr4	0.2310083	1.004474	0.5538874	0.8826866
Sri	-0.0853579	7.5619861	0.5540076	0.8826866
Syncrip	0.0665965	7.9972404	0.5540295	0.8826866
Alkbh5	0.0715251	6.3752597	0.5540485	0.8826866
Gatc	0.0872073	4.5292408	0.5542041	0.882799
Rbm18	0.0819319	5.0048507	0.5542325	0.882799
Taz	0.0863996	4.839616	0.5543449	0.8828876
Bloc1s6	-0.072549	4.9924089	0.5544841	0.883019
2810004N23Rik	-0.0753534	4.509515	0.5545759	0.8830747
Rpl9-ps6	0.1771815	2.2795983	0.5546354	0.883079
Sfrp2	-0.2848721	2.3195299	0.5547355	0.8830994
Mis18a	0.089659	5.5975042	0.5547964	0.8830994
Lpin3	0.3571597	-0.2915739	0.5548185	0.8830994
Hspa8	0.1008676	9.8201385	0.5550087	0.8833117
Rad18	-0.1043071	4.810744	0.5552228	0.8835621
Ric1	0.0816373	4.6635479	0.5554641	0.8838556
Cd2ap	-0.0839569	6.1768307	0.5556654	0.8840854
A230056P14Rik	0.271995	-0.5026314	0.5558317	0.8841701
Fam120aos	0.115836	3.3706807	0.5558416	0.8841701
Ppat	0.0672619	6.002223	0.5558906	0.8841701
Irf2	0.0734521	6.2947678	0.5560144	0.8841701
Oxnad1	0.106894	3.6380674	0.5560441	0.8841701
Robo1	-0.1721761	3.2250405	0.5561411	0.8841701
Tpcn2	-0.1042255	3.7348625	0.5561633	0.8841701
Acot1	-0.2223128	2.6982632	0.5561734	0.8841701
Tnc	-0.1446436	8.9635918	0.5562384	0.8841831
Gtf3c6	0.0749618	5.0682495	0.5565037	0.8844485
Efhd2	-0.0879815	6.841258	0.5565192	0.8844485
Cetn2	-0.0829636	5.0297814	0.5565759	0.8844485
Map2k3	0.0731385	6.7780626	0.5567376	0.8844754
Usp38	-0.0679475	5.8300651	0.5567567	0.8844754
Zeb1	0.0689279	5.5396729	0.5567999	0.8844754
LOC118567533	0.3382939	0.3542958	0.5568203	0.8844754
Ints11	0.0754302	4.9554376	0.5570917	0.8846744
Gm8186	0.0832124	5.4321672	0.5571475	0.8846744
Mrpl50	0.0854122	5.3039335	0.5571571	0.8846744
Aftph	0.0630603	6.5518899	0.5572179	0.8846744
Tmem160	0.0892766	4.3596995	0.5572847	0.8846744
Cd68	-0.0910289	6.7853731	0.5572869	0.8846744
Dpm2	-0.0807602	4.5684167	0.5574562	0.884699
Lime1	0.1258053	3.7428797	0.5575186	0.884699
Eef2-ps2	-0.3153892	-0.3120768	0.557576	0.884699

1110025M09Rik	0.1387042	2.3868229	0.5576032	0.884699
Txlna	0.0756447	6.514956	0.5576311	0.884699
Crppa	-0.2042283	1.221111	0.5576512	0.884699
Csrnp2	-0.1673911	1.822045	0.5577005	0.884699
Eif3m	0.0871346	7.0318096	0.5578253	0.8847458
Cyth4	-0.0610943	6.6741312	0.5578438	0.8847458
Sybu	0.2998052	0.3568824	0.5579527	0.8848283
Zfp963	0.1100535	2.7548431	0.5581469	0.8850256
Xpo5	-0.0806867	5.9718871	0.5581909	0.8850256
Tex30	0.0865617	4.1925615	0.558262	0.885032
Naf1	0.0975907	4.2104099	0.5583224	0.885032
Recql	-0.1016455	5.0556362	0.5583852	0.885032
Dnaaf2	0.0761726	4.6785822	0.5584787	0.885032
Sass6	-0.0731142	5.3973881	0.5584794	0.885032
Thop1	0.1053412	4.2882616	0.5585532	0.8850578
Styx	-0.1021481	3.5191905	0.5586326	0.8850578
1110028F11Rik	-0.2844572	-0.7925596	0.55878	0.8850578
Rab12	0.0879364	4.7485833	0.5588206	0.8850578
Trim16	-0.11746	3.67179	0.558837	0.8850578
A330035P11Rik	0.3650848	-0.8377265	0.5588371	0.8850578
Gm34703	-0.229342	0.8547484	0.5590079	0.8851546
Dact1	0.1929597	2.0358798	0.5590121	0.8851546
Gpx8	0.1999432	4.9981955	0.5590784	0.8851695
Gm51821	0.2545583	-0.0071885	0.5592493	0.88535
Rps4x	0.0771846	9.9157133	0.5593083	0.8853533
Impa1	0.0690046	6.0842065	0.5593726	0.8853569
Mst1	0.2378572	1.1766105	0.5594244	0.8853569
Itpr3	0.1315625	4.9732582	0.5595005	0.8853621
Snx11	-0.0762641	4.785576	0.5596334	0.8853621
Ing1	-0.0627922	5.8903837	0.5596643	0.8853621
Ppard	-0.0925424	4.1915109	0.5597531	0.8853621
St6galnac6	0.0968495	4.5218282	0.5598775	0.8853621
LOC118567383	-0.171908	1.9866756	0.5599923	0.8853621
Bex4	-0.1640735	3.8838225	0.5600076	0.8853621
Casc3	0.0691896	6.3057695	0.5600115	0.8853621
Gm14048	-0.2709642	-0.8541988	0.5600196	0.8853621
Agpat3	0.0718984	5.5698592	0.5600562	0.8853621
Myo19	0.1188435	3.6466616	0.5601573	0.8853621
Prmt2	0.1415682	2.7841895	0.5601882	0.8853621
Zfp322a	0.067105	5.3948437	0.5602135	0.8853621
Nus1	0.0632997	6.085328	0.5602828	0.8853621
Pfn4	0.330157	-0.3479872	0.5603185	0.8853621
Znrf1	-0.0838886	6.1378949	0.5603384	0.8853621

Mfsd4b4	-0.1549292	2.5719154	0.5605452	0.8855987
Mmp19	0.1287472	3.419265	0.5607701	0.8858641
Hepacam2	0.2961928	0.402492	0.5610221	0.8860682
Fryl	0.1265423	7.5801126	0.5610505	0.8860682
Fbxw2	0.0799074	6.1977074	0.5611527	0.8860682
Dnase2a	0.1078685	5.0032838	0.5611685	0.8860682
Nisch	-0.0717753	7.6652363	0.561194	0.8860682
Pdpr	-0.0862783	5.3930256	0.5612457	0.8860682
Taok2	-0.0808129	6.8346407	0.5613729	0.8860682
2610020C07Rik	0.1975514	1.8024266	0.5614094	0.8860682
Zfp688	0.1358839	2.5710793	0.561412	0.8860682
Pigl	0.1210595	3.444527	0.5617009	0.8862568
F630048H11Rik	-0.248493	1.375693	0.5617397	0.8862568
Phf14	0.0802069	5.7855069	0.5617408	0.8862568
Cpped1	0.0892989	4.3039442	0.5617594	0.8862568
Itga7	-0.3152106	0.3806005	0.5618708	0.8862832
Gm51737	0.3526615	-1.0472851	0.5619189	0.8862832
Wnk1	-0.0772835	8.7796887	0.5620422	0.8862832
Shroom4	-0.1579919	2.7317866	0.5621058	0.8862832
Manba	-0.0796507	4.7976215	0.562159	0.8862832
Ache	0.0968006	6.468724	0.5621981	0.8862832
Sox7	0.3277317	-0.0034018	0.5622184	0.8862832
mt-Rnr2	-0.1384209	9.9652456	0.5622714	0.8862832
Pik3ip1	0.15106	4.2275768	0.562289	0.8862832
Drp2	0.2199868	0.7787404	0.5624113	0.8863308
Spaca6	0.2886185	1.6356717	0.5624332	0.8863308
S1pr3	0.1342051	5.1049247	0.5630897	0.8872756
Fam124a	0.234287	0.9447932	0.5633618	0.8876144
Ankrd65	0.4103769	-0.7108584	0.563462	0.8876824
Clpb	0.085518	4.1716003	0.5636954	0.8879601
Hlf	0.1792693	2.4155566	0.5638485	0.8881113
Gm6278	-0.3261001	-0.7165855	0.564113	0.8883964
Gm31223	-0.360157	-1.0433337	0.5641437	0.8883964
Ifit1bl1	-0.2036928	-0.0326186	0.564308	0.8885651
Dcaf4	0.1048494	3.8009697	0.5645387	0.888786
Sar1a	0.0719858	7.1131334	0.5646023	0.888786
Vps51	0.0746634	4.5686721	0.5646197	0.888786
Nupl2	0.1595683	2.693522	0.5648957	0.8891305
Wdr24	0.0951953	4.1724549	0.5649749	0.8891652
Gm20585	0.4419517	-0.7143712	0.5650545	0.8892006
Zscan26	0.0987342	4.929656	0.5653863	0.8896257
Rab32	-0.0687315	6.0512887	0.5654469	0.8896257
Fgfr3	-0.1938744	3.225925	0.5655511	0.8896257

Efr3a	-0.0702459	6.3483421	0.5655535	0.8896257
Camk2b	-0.291705	1.6286879	0.5656813	0.8897367
Pla2g6	0.1284152	2.8959882	0.5658941	0.8898973
Tmem104	0.1117407	4.132314	0.5658978	0.8898973
Cpe	0.2155968	7.4685649	0.5659943	0.8899591
Gm15506	-0.2685355	0.748643	0.5660907	0.8900206
Gpr160	-0.1093979	4.0360132	0.5662028	0.890107
Gpc3	0.2952437	0.077548	0.5664039	0.8901538
Sgpp1	0.0753197	6.6981559	0.5664725	0.8901538
Lyrm9	0.1644393	3.4893335	0.5665049	0.8901538
Qtrt2	0.1192862	3.1850927	0.566508	0.8901538
Sdsl	0.146328	2.6502433	0.5666295	0.8901538
Naalad2	0.2368909	1.5346503	0.566662	0.8901538
Gm26535	-0.4477704	-1.0594755	0.5667667	0.8901538
Rala	0.0863929	5.2787125	0.5668108	0.8901538
Sh3bgrl3	0.0799215	8.4028803	0.5668436	0.8901538
Dzip1l	-0.2256453	1.00624	0.5669095	0.8901538
Tyro3	0.2562376	0.0067025	0.5669656	0.8901538
Rttn	0.0951514	3.871939	0.5669697	0.8901538
LOC102636360	-0.2544125	-0.3012101	0.5670424	0.8901538
Itpr2	-0.120172	5.7560181	0.5670765	0.8901538
Bbc3	-0.1383038	3.067152	0.567091	0.8901538
Mettl7a1	0.077201	5.5065778	0.5673387	0.8904125
Tfdp2	0.1111381	8.294547	0.5673703	0.8904125
Dmd	-0.1702887	1.1534381	0.5674976	0.8904388
Tomm5	0.1011723	5.5078305	0.567515	0.8904388
Cd80	-0.1679868	2.1099225	0.5675588	0.8904388
Gm12312	-0.3198401	1.4077536	0.5676719	0.8905264
Taf2	0.0711634	5.9202868	0.567912	0.8907938
Gm5776	-0.4468043	-0.9424412	0.5680829	0.8907938
Pcdhgb1	0.2630341	-0.381051	0.5681764	0.8907938
Zfp975	0.1535594	2.0884801	0.5681858	0.8907938
Sf1	0.0692882	7.9968796	0.5682477	0.8907938
Sumf1	0.0758047	5.0261151	0.5682939	0.8907938
Urgcp	-0.0829785	4.2661299	0.5682948	0.8907938
Pcx	0.1201252	4.8881474	0.5683005	0.8907938
Ankrd13a	0.0783032	8.2097429	0.5684649	0.8908294
Fbln2	-0.1972591	1.7973917	0.5685049	0.8908294
Art3	-0.2446356	0.9473226	0.5685398	0.8908294
Bnip3l-ps	-0.2067121	1.1377213	0.568646	0.8908294
Galm	-0.1089807	3.1330404	0.5686487	0.8908294
Sdhaf4	0.1595616	3.1821447	0.5686987	0.8908294
Gm10222	-0.2536757	-0.5018386	0.5687242	0.8908294

Sdhaf3	0.1618074	1.479629	0.568954	0.8910123
Zmat1	-0.1261201	2.7057091	0.5690117	0.8910123
Tespa1	-0.1814495	2.1150885	0.5691035	0.8910123
Ccdc69	0.1299396	2.8307856	0.5691684	0.8910123
Araf	0.0676281	5.3342331	0.5691725	0.8910123
Gm1943	0.1308453	2.5584534	0.5691847	0.8910123
Ifi44	0.4056087	2.5954256	0.5693865	0.8912385
Trafd1	-0.0746774	5.4495117	0.5695682	0.8913413
Yae1d1	-0.1163621	3.3667148	0.5695784	0.8913413
Ccdc163	0.1400592	3.0799261	0.5696241	0.8913413
AI662270	0.0836042	7.8872891	0.5697649	0.8914236
Gm15663	0.4015422	0.5034784	0.5697913	0.8914236
Gdap2	-0.078314	4.56571	0.5699325	0.8914479
Trmt112	0.0665039	5.797694	0.5699524	0.8914479
Gm15417	0.2295161	0.9113424	0.5700038	0.8914479
Thyn1	0.1040001	4.2150808	0.5700745	0.8914479
Srbd1	-0.0737413	4.9445514	0.570116	0.8914479
Fah	-0.17653	2.8821826	0.5701615	0.8914479
Decr1	-0.0868069	4.5027972	0.5702177	0.8914479
P3h3	0.1626497	3.6931998	0.570292	0.8914479
Afg3l2	0.0793538	6.4128945	0.5703226	0.8914479
Pdcd6ip	-0.070787	7.2845421	0.5704492	0.8915562
Mybpc2	-0.2763815	1.653218	0.5705759	0.8916208
Spin1	-0.0894717	6.0057485	0.5706052	0.8916208
Erfe	-0.332166	1.128149	0.5708821	0.8918406
Rab7b	-0.1362211	2.6104795	0.5709622	0.8918406
Gm38486	-0.3642756	-0.4558823	0.5709697	0.8918406
Mthfd1	0.097441	6.6277428	0.5710261	0.8918406
Gm4864	0.2235746	1.0689623	0.5710617	0.8918406
Mcoln3	0.4958341	-0.9548818	0.5710899	0.8918406
Atp6v1g1	-0.0995744	7.1577894	0.5713201	0.8921105
Ash2l	0.0712719	5.5005658	0.5715169	0.8922061
Tmem87b	0.0824631	4.597826	0.5715998	0.8922061
Cpxm2	0.3162409	1.4476986	0.5716312	0.8922061
Rragd	-0.1111361	2.9844327	0.5716476	0.8922061
Zfp142	0.1175666	4.4929144	0.5716681	0.8922061
Ccdc62	-0.199347	1.0183358	0.5717786	0.892289
Gm32059	-0.1377385	3.4071881	0.5720368	0.8926023
Cd34	-0.0976148	4.6745013	0.5721308	0.8926595
Gm9115	0.3386562	-0.7252184	0.5722634	0.8927768
Pecam1	0.0784069	6.0787752	0.5725455	0.8929168
Edem2	-0.0901013	5.3343736	0.5725481	0.8929168
Pigz	0.3141896	0.0171735	0.5726209	0.8929168

Pdk4	0.2107927	4.1387653	0.5726283	0.8929168
Commd4	0.0908861	4.5961317	0.5727603	0.8929168
Taf9	0.0699786	6.4542839	0.5728132	0.8929168
Sin3a	-0.0721584	6.6164812	0.5729333	0.8929168
Spint2	0.0951884	4.6841069	0.5729462	0.8929168
Ube2w	-0.0945732	5.1332898	0.5729469	0.8929168
Hip1r	-0.0933842	5.6287379	0.5729545	0.8929168
Gm6180	-0.1242505	2.1386635	0.5730353	0.8929168
Ccdc120	0.3436539	-0.4975904	0.573042	0.8929168
Rpp30	-0.0939397	4.1626596	0.5731307	0.8929656
Gm12174	0.1278765	2.6220813	0.5732188	0.8930133
LOC118568760	-0.3136146	-0.7558486	0.5733485	0.8930542
Piga	0.091217	5.3442084	0.5733791	0.8930542
Ttc27	0.0805939	4.0597764	0.5734571	0.8930542
Ikbkg	-0.0695532	5.3341338	0.5734747	0.8930542
Slc35d1	-0.0954432	3.9901566	0.5736123	0.893179
Adig	0.5152114	-0.7192017	0.5737338	0.8932788
Cma1	0.4713507	-0.22369	0.5739783	0.89357
Dcaf11	0.071948	5.6466865	0.5741174	0.8936129
Ankfy1	-0.0686021	6.9009341	0.5741207	0.8936129
Gm13464	-0.3280129	-0.5876852	0.5742704	0.8937564
Lamc1	0.095589	5.5234467	0.5743314	0.893762
Igkv4-58	-0.3624097	0.4957902	0.5743891	0.8937622
Fth1-ps	0.3451698	-0.9239524	0.5745015	0.893792
Arl6	-0.1678825	1.8415478	0.5745231	0.893792
Mettl1	0.1150747	3.1501983	0.5746256	0.8937936
Kctd17	0.1052755	3.6818902	0.574639	0.8937936
Pxmp2	0.1610523	2.848456	0.5747307	0.8938087
Gm13408	0.2810525	0.4132044	0.5747637	0.8938087
Gm6451	-0.3944663	-0.808139	0.5748788	0.8938983
Dmp1	-0.2699741	7.8090227	0.5749599	0.8939351
Tbc1d20	-0.0715283	6.2662115	0.5750895	0.8940168
Cad	0.0845548	5.8070418	0.5751274	0.8940168
Scaf8	0.0807108	6.0230126	0.5752152	0.8940638
Gng10	0.0923054	6.1286217	0.5752944	0.8940976
Zfp369	-0.0856255	5.0293659	0.5755527	0.8942356
Adrm1	0.0850534	5.5415511	0.5756189	0.8942356
Cox15	0.0784745	4.5652237	0.5757246	0.8942356
Slc9a3r1	-0.0740893	6.2300411	0.5757795	0.8942356
1810055G02Rik	-0.095084	4.4195783	0.5757962	0.8942356
Ctse	0.0882151	9.7259016	0.5758042	0.8942356
Slc26a3	0.5198098	-1.1342564	0.5758372	0.8942356
Gli2	0.2158327	0.771748	0.5758632	0.8942356

Ptprg	-0.1392307	4.0783084	0.5759455	0.8942356
Chd8	0.0669868	6.710695	0.5760017	0.8942356
M6pr	-0.0612423	7.5457866	0.5760156	0.8942356
Cacfd1	0.0943738	3.9778304	0.5761917	0.8944198
Wdr59	0.1601729	3.2359913	0.576442	0.8945175
Akip1	0.0974067	4.6000671	0.57649	0.8945175
Gm24175	-0.2476225	0.6740491	0.5765068	0.8945175
Derl2	0.0628387	5.6184931	0.5765348	0.8945175
Adam9	-0.0661925	5.5025455	0.5765832	0.8945175
Zfp212	-0.0851213	4.3508499	0.5767455	0.8945175
Meg3	-0.3956743	-0.010117	0.5768547	0.8945175
Rhoc	-0.1250296	5.0028652	0.5769081	0.8945175
Kdm6a	-0.06815	6.8051679	0.576966	0.8945175
Efcc1	0.3169808	-0.0895682	0.5770545	0.8945175
Vamp4	-0.0692122	4.9381812	0.5770694	0.8945175
Plekhh1	0.213815	0.2973019	0.5771044	0.8945175
Bckdk	0.0698491	5.6434943	0.577108	0.8945175
Selenof	-0.0815134	8.1194291	0.5772132	0.8945175
Icmt	0.0723798	5.1491144	0.5773535	0.8945175
Ifi27l2a	-0.1873546	4.6535066	0.5773745	0.8945175
Rab7	-0.0648619	8.0694988	0.5773779	0.8945175
Tmem183a	0.0841041	6.0280084	0.577391	0.8945175
Kcnj16	-0.3772846	-0.0455214	0.577445	0.8945175
Armcx6	-0.1380423	1.911457	0.5774493	0.8945175
Gpr182	0.1507404	3.2714706	0.5774624	0.8945175
Rfx7	-0.0917163	5.8454634	0.5776963	0.8947907
Ppp4r1l-ps	-0.1498899	2.0526598	0.5778818	0.8949888
Golph3l	-0.0780116	6.078723	0.577965	0.8950286
Ccdc173	0.2287135	0.7475479	0.5780649	0.8950592
Oscar	0.2218861	3.4031744	0.5780998	0.8950592
Gm39286	-0.2190107	0.6564471	0.5781834	0.8950779
Ppp1r2	-0.0658528	6.5767322	0.578227	0.8950779
Lrrc51	0.3175058	-0.6790621	0.5783693	0.8951343
Pttg1	0.0988446	6.3438533	0.5784235	0.8951343
Gatd3a	0.0690807	5.0013285	0.5784361	0.8951343
Itga10	-0.1864785	4.6037601	0.578575	0.8951487
Ist1	-0.0596116	6.5328459	0.5786026	0.8951487
Arhgdia	0.0754339	8.206193	0.578618	0.8951487
Kcnj9	-0.1985944	0.1762207	0.5787734	0.8952038
Adamts18	0.2579202	0.617738	0.5788216	0.8952038
Bace1	-0.0884808	4.7551897	0.5788263	0.8952038
Aldh16a1	0.0689184	5.1255396	0.5789038	0.8952346
Usp11	0.106828	4.0331043	0.5790466	0.8953664

Gm15427	-0.0995046	4.4035125	0.5791638	0.8954569
Hecw2	-0.1877182	1.9571538	0.5792203	0.8954569
Hspb2	0.3819156	-0.0721213	0.5793472	0.8954796
Tbcb	0.0921607	4.904917	0.5793605	0.8954796
Inf2	-0.0644573	5.9907029	0.57946	0.8954796
Mfsd11	-0.0880402	4.1886672	0.5794652	0.8954796
Numbl	-0.1372495	2.5610438	0.5796088	0.8955224
Gm9056	-0.2740724	-0.5089801	0.5796821	0.8955224
Mfap1b	-0.1007831	3.4644912	0.5797485	0.8955224
Tnfaip6	-0.3401755	-0.0966439	0.5797777	0.8955224
Sap30l	0.0855757	4.4493467	0.5797808	0.8955224
Cyth3	0.0974729	6.0797176	0.5800206	0.8957034
Adck2	0.1080889	3.5231327	0.580035	0.8957034
Acaa1a	0.1528875	4.4932502	0.5800707	0.8957034
Zfp24	-0.0591075	5.4120606	0.5802524	0.895786
Gm11767	-0.2818027	-0.5847531	0.5803847	0.895786
Ptov1	0.1185892	4.9347903	0.5804045	0.895786
Ankrd16	0.0942045	4.7264305	0.5804225	0.895786
Sgcb	-0.1021098	3.8351739	0.5804777	0.895786
Msrb2	-0.0993156	3.3520548	0.580508	0.895786
L2hgdh	-0.1241522	3.5792242	0.5805285	0.895786
Sgsm3	0.1114927	3.6032776	0.5806028	0.895786
Ears2	0.1315417	2.2473683	0.580742	0.895786
Def8	-0.0821107	5.5975198	0.5808867	0.895786
Cbarp	-0.2354674	0.6518956	0.580912	0.895786
Cplane1	-0.1092816	3.4399069	0.5809604	0.895786
Bcl9l	-0.1147106	4.4712845	0.581028	0.895786
Ffar4	-0.318465	-0.4608184	0.5810485	0.895786
Tmem128	-0.0821905	5.0547708	0.5810977	0.895786
Klhl13	-0.1932603	2.6688053	0.5812467	0.895786
Rb1	0.0958894	7.8455223	0.581252	0.895786
Cnpy3	-0.0718075	5.2145318	0.5813149	0.895786
Arntl2	0.4286576	-0.8889034	0.581333	0.895786
Fam120a	0.064284	8.291352	0.5813617	0.895786
Slc4a1ap	0.0655694	4.9930112	0.5814394	0.895786
Zbtb9	0.0858802	4.2067986	0.5815115	0.895786
Zfp609	-0.0921591	5.3106928	0.5815334	0.895786
Brdt	0.3005511	0.3980628	0.581615	0.895786
Norad	-0.0566627	7.1406367	0.5816516	0.895786
Pcdh12	-0.1541648	2.1204713	0.5817435	0.895786
Il12rb1	0.2038541	0.5963709	0.5818271	0.895786
Tspan3	0.1125732	5.0569112	0.5818613	0.895786
A530040E14Rik	0.3476179	-0.0732642	0.5818941	0.895786

Slco2a1	-0.1209698	3.162388	0.5819561	0.895786
Cgrrf1	0.1005355	3.9688202	0.582216	0.895786
Cdh23	-0.271773	0.4973008	0.5822554	0.895786
Ddx41	0.0732061	5.2083172	0.5823012	0.895786
Gtf2h3	0.0870294	4.1069549	0.5823109	0.895786
Slc27a1	0.136293	3.68027	0.5824063	0.895786
Map3k4	0.0963472	4.8331438	0.582626	0.895786
Gm51875	0.2384827	-0.1397747	0.5827366	0.895786
Tubb3	0.3050682	-0.3104966	0.5827424	0.895786
Slc35e3	0.1070882	3.4781554	0.5827607	0.895786
Gabpb1	0.0618069	5.6433091	0.5828138	0.895786
Ndfip2	0.0747322	4.9210272	0.5828434	0.895786
Cggbp1	-0.0669064	7.4846356	0.5828615	0.895786
Igtp	-0.1131013	5.6525328	0.5829795	0.895786
Srp9	0.1000119	6.8063288	0.5830159	0.895786
Adprh	-0.0758148	5.4810496	0.5830276	0.895786
Glrp1	0.2802651	-0.0939677	0.5830353	0.895786
Rmnd5b	-0.0709663	4.6915488	0.5831066	0.895786
Frmd6	-0.087544	5.0102586	0.5831499	0.895786
LOC118568402	-0.1291608	2.546342	0.5831587	0.895786
Il1rl2	0.0889944	4.4594267	0.5831729	0.895786
Uqcrc2	-0.0656235	7.0470297	0.5831921	0.895786
Gm7666	-0.1985083	0.4317842	0.5832186	0.895786
2810013P06Rik	0.0954566	3.8909943	0.5832955	0.895786
Wnt10b	-0.2241653	0.9244006	0.5833391	0.895786
Gm7332	-0.2681466	-0.2041516	0.5833431	0.895786
Rin1	-0.2578121	-0.1515242	0.5833878	0.895786
Adgrl3	0.2421991	-0.3215745	0.583407	0.895786
Rnf146	-0.0624364	5.7436137	0.5835241	0.8958774
Enox2	-0.0936618	3.8328619	0.5836593	0.8959533
1810037I17Rik	-0.0992125	6.6880834	0.5836947	0.8959533
Kif11	-0.0790669	8.1635616	0.5837463	0.8959533
LOC118567621	0.230162	1.3606598	0.5838825	0.8960552
Oxsr1	0.0635612	6.0036941	0.583928	0.8960552
Rps17	0.0898771	9.4710927	0.5840836	0.8960818
Alkbh8	0.0742613	5.51768	0.5841203	0.8960818
Cops2	0.0857494	7.1130435	0.5842073	0.8960818
Dpf2	0.076849	6.1586183	0.5842178	0.8960818
2500002B13Rik	0.1782055	2.8263741	0.5842334	0.8960818
Rpl22l1	0.147372	6.1084236	0.5843178	0.896123
Supt6	-0.0678667	6.8966667	0.5843953	0.8961534
Ccny	0.0572833	6.5175715	0.584786	0.8966343
Nsun3	-0.1087337	4.1579161	0.5848242	0.8966343

Birc2	0.0782697	5.7661861	0.5849402	0.8967238
Gm42031	0.2682392	0.8457594	0.5851386	0.8969395
Gm8806	0.2050479	0.3371045	0.5854075	0.8972634
Vdac3	0.0649608	7.1007168	0.5855746	0.8973369
Slc25a4	0.0894146	6.5659579	0.5855838	0.8973369
Crls1	0.0784852	4.5990643	0.585672	0.8973369
Rad51ap1	0.0828017	5.495992	0.5857396	0.8973369
Neu3	0.1817292	3.458075	0.585744	0.8973369
Cdr2l	0.1642755	3.8159253	0.5859081	0.8973564
Gm20522	0.302345	-0.815519	0.5859187	0.8973564
Celf5	0.3184169	0.1308602	0.5860143	0.8973564
Dpy19l3	-0.1212246	3.6090474	0.5860876	0.8973564
Acsf3	0.1241002	2.9891479	0.5861276	0.8973564
Rxra	0.0797499	4.9605006	0.5861499	0.8973564
Cpsf1	0.0698445	5.6935819	0.5861653	0.8973564
Cfap97	-0.0724075	4.7907771	0.5862182	0.8973564
2410004B18Rik	0.077198	4.132523	0.5864459	0.8975304
Cfdp1	0.071017	6.3472889	0.5864929	0.8975304
Ndufa9	0.0702078	5.9567591	0.586505	0.8975304
Tomm7	0.0959377	6.1960659	0.5867157	0.8977644
Angel2	0.0571306	5.999649	0.5868476	0.8978182
Mtmr9	-0.0702435	5.353317	0.5869233	0.8978182
Nyap1	-0.1842253	0.945741	0.586924	0.8978182
Alg2	0.0785493	4.4834492	0.5871012	0.8979728
Megf10	-0.1884261	2.3871781	0.5871405	0.8979728
Cdyl2	-0.1274174	3.0723927	0.5872408	0.8980235
Grcc10	0.0920072	5.5621006	0.5872891	0.8980235
Nhlrc2	0.0693378	5.8851684	0.5873784	0.8980717
Gpr52	0.3532712	-0.5672012	0.5874565	0.8981028
Ckb	0.1546424	6.7484715	0.5877049	0.8981675
Tpbg	-0.2172779	2.5200545	0.5877581	0.8981675
Kif2c	0.0837966	5.9049221	0.587848	0.8981675
Zfp983	-0.1092258	3.2564656	0.5879432	0.8981675
Casp6	0.0911312	4.3835264	0.5880094	0.8981675
Sptbn4	-0.260145	-0.3495681	0.5880615	0.8981675
Chchd1	-0.0871582	5.2409502	0.5880859	0.8981675
Ak1	0.1381307	4.0937177	0.5880936	0.8981675
Wipf3	-0.2917276	-0.4890386	0.5880992	0.8981675
Mtpap	0.0683118	5.0318437	0.5881327	0.8981675
Cox7c	0.1063524	7.2526716	0.5881826	0.8981675
Mtr	-0.087316	5.4966329	0.5882136	0.8981675
Hopx	-0.131906	3.9809007	0.5882621	0.8981675
Zfp174	-0.2244449	0.9905576	0.5883072	0.8981675

Fzd5	-0.0903054	4.7703457	0.5884426	0.8982265
Znrd1	0.1101897	4.2000947	0.5884613	0.8982265
Tsga10	-0.1359407	2.2729339	0.5886299	0.8983955
Anp32b-ps1	0.2518152	-0.1872786	0.5887671	0.8984321
Ei24	0.060045	5.6353865	0.5887693	0.8984321
Actg2	0.2322023	-0.1546513	0.5888629	0.8984464
Rec8	0.3217759	0.6895351	0.5888942	0.8984464
Cyp4f17	-0.2221067	0.913607	0.5889681	0.8984552
Alms1	-0.07874	4.7011946	0.5890155	0.8984552
Shprh	-0.0706677	5.4694657	0.5892464	0.898636
Syt7	-0.293882	-0.5325012	0.5893505	0.898636
Cdkn3	0.1372982	5.4454745	0.5893791	0.898636
Gm46137	0.3157356	-0.3309908	0.5894407	0.898636
Gm34039	0.2868021	0.357	0.5895396	0.898636
Cdh6	0.332394	0.012023	0.5895597	0.898636
Limk2	0.0698339	4.9692655	0.589569	0.898636
Trim36	0.1800157	1.6322419	0.5896978	0.898636
D330050I16Rik	0.2112674	0.2461082	0.5897094	0.898636
Sema3d	0.2373766	4.9207245	0.5897718	0.898636
Ctsb	-0.070441	9.8321195	0.5898054	0.898636
Tnfsf11	-0.1588591	2.577559	0.5898274	0.898636
Zfp217	-0.0675579	6.5396801	0.5899066	0.8986549
Sox6	0.107463	7.1243839	0.5899553	0.8986549
Smc1a	-0.0758459	8.382236	0.5900828	0.8987307
Lpcat4	-0.1205559	3.8570501	0.5901206	0.8987307
Msantd1	0.2212233	0.4217578	0.5903833	0.8990426
Sav1	0.0605442	6.6460564	0.5905296	0.8991668
Trarg1	0.3287595	1.5388417	0.5905804	0.8991668
Ublcp1	0.0879992	4.7984329	0.5906551	0.8991925
Gse1	0.0903484	7.3965071	0.5908439	0.8992431
Zfp236	-0.0757432	5.6453898	0.5908754	0.8992431
Fndc4	0.2365372	1.0061227	0.5908963	0.8992431
Eprs	-0.0653693	7.1999512	0.5909196	0.8992431
Kyat3	0.1388339	1.8882054	0.5909909	0.8992635
Rps15-ps2	-0.2627236	-0.5656395	0.5910631	0.8992855
Arfip1	0.0760774	5.4629747	0.5913999	0.8994778
U2surp	0.0665951	7.092282	0.5914254	0.8994778
Gm28523	-0.4429993	-0.3326792	0.5914868	0.8994778
Cep95	0.0769209	4.6297427	0.5915234	0.8994778
Nrbp1	0.0616665	5.9800623	0.5915597	0.8994778
Cmas	0.0913611	7.3355087	0.5915692	0.8994778
Gm6712	0.1147734	2.6789451	0.5918449	0.8994778
Spry4	0.16169	1.7508415	0.5919543	0.8994778

Tspan14	-0.0642674	6.4824304	0.5919628	0.8994778
Car5b	0.2467731	1.3648483	0.5919832	0.8994778
Pphln1	-0.0573525	5.6206165	0.5920018	0.8994778
Gm34894	0.2074959	0.0069946	0.5920033	0.8994778
Gm12708	-0.2570898	-0.7524367	0.592043	0.8994778
Fchsd2	-0.0915889	5.0220925	0.5921265	0.8994778
Ankrd17	-0.0904557	7.2395082	0.592202	0.8994778
Krt222	-0.3162608	-0.6263195	0.5922067	0.8994778
Crybb3	-0.3226087	-0.1668786	0.5922262	0.8994778
Ppp1r7	0.0715509	5.750735	0.5922305	0.8994778
Tubb6	0.1272723	4.1224002	0.5923556	0.8995801
Coa3	0.0753718	4.9988679	0.5926093	0.8996154
Mest	0.1343372	2.6449582	0.5926646	0.8996154
Ggnbp2os	0.1956954	1.9945513	0.5926784	0.8996154
Pigw	0.1225346	3.053696	0.5927058	0.8996154
Rsbn1l	-0.0581427	6.5159844	0.5927096	0.8996154
Psmd1	0.0622155	7.120531	0.5928237	0.8996154
Gm10167	-0.2715268	0.287589	0.5929777	0.8996154
A430057M04Rik	0.3789908	-0.3856397	0.5930007	0.8996154
Eif4enif1	0.0717393	6.7446552	0.5930182	0.8996154
Vamp5	0.0863135	4.9307746	0.5930739	0.8996154
Mark1	0.1902861	2.7225112	0.593101	0.8996154
Aaas	0.0611274	5.1119487	0.593146	0.8996154
Psma2	0.0726484	7.1881424	0.5931464	0.8996154
Eif3b	0.0769539	7.5690752	0.5932557	0.8996154
Tab2	0.0602877	7.653143	0.5933317	0.8996154
Gm15834	0.3060172	0.4402926	0.5933538	0.8996154
Kat7	-0.0658986	6.3189457	0.5934577	0.8996154
Ankrd10	0.0795414	5.7348439	0.593538	0.8996154
Ide	0.0696594	6.1348724	0.5936078	0.8996154
Cand1	0.0564327	6.4268785	0.5936744	0.8996154
Invs	0.0992496	3.1394799	0.5936954	0.8996154
Hlcs	-0.1230921	3.6437295	0.5936962	0.8996154
Rab21	-0.0789077	6.4872482	0.5937092	0.8996154
Entr1	0.0584239	5.7761076	0.5939588	0.899906
Ipmk	-0.0647809	6.194641	0.594058	0.8999687
Cep162	0.1025521	3.9756878	0.5941456	0.8999782
Rbis	0.112529	5.9992091	0.59418	0.8999782
Sos1	-0.0697789	5.1812773	0.5942718	0.899988
Slirp	0.109899	5.1512448	0.5943982	0.899988
Fut10	0.133906	2.3841295	0.5944195	0.899988
Adgre1	-0.0962961	5.0392844	0.5944975	0.899988
Taok3	-0.083868	6.9301814	0.5945003	0.899988

2610027K06Rik	0.3170758	-0.2053433	0.594602	0.899988
Jcad	-0.0819032	4.419249	0.594643	0.899988
LOC115488002	0.452576	0.0193241	0.5946494	0.899988
Pwwp2b	-0.1492271	2.7656502	0.5948424	0.9001925
Golgb1	-0.0710603	6.3353298	0.5950936	0.9004379
Eid2	0.1374455	1.9502199	0.5951203	0.9004379
Atf7ip	-0.0680978	7.2489963	0.5951888	0.900454
Usp27x	0.2901632	-0.3133157	0.5955005	0.90079
Rad54b	0.1042712	3.5516996	0.5956108	0.90079
Crybg1	0.1058561	4.0033581	0.5956471	0.90079
Trappc10	-0.0675943	6.7901827	0.5956593	0.90079
Dtd2	0.1131313	2.953143	0.5957005	0.90079
Gm15163	-0.2940493	-1.1653468	0.5960273	0.9011965
Wdr20	-0.0848738	4.5277809	0.5962313	0.9014174
Naaladl2	-0.2353343	1.2035459	0.5963072	0.9014444
Rabl3	0.1096293	3.4071034	0.5964199	0.9015272
Spop	0.0578631	7.4355824	0.5966711	0.9016539
Magi2	-0.1666197	3.7559441	0.5967104	0.9016539
Gpn3	0.0916332	4.4482864	0.5967135	0.9016539
Wdr61	-0.0718253	5.161054	0.5967356	0.9016539
Gm11263	0.2167223	0.4633548	0.5969024	0.9018184
Arc	-0.293707	-0.7007095	0.5970409	0.9019052
Smarca5	0.0680125	8.3520457	0.5971601	0.9019052
Rnf31	0.0678336	4.619404	0.5972089	0.9019052
Tmem70	-0.0720229	4.3572517	0.5973506	0.9019052
Zmym5	0.0651605	5.6088059	0.5973557	0.9019052
1300017J02Rik	0.1139521	5.6329269	0.5974473	0.9019052
Pex5	-0.0618278	4.9599196	0.5974604	0.9019052
Tincr	0.2863721	-0.6490817	0.5974888	0.9019052
Klf11	-0.0962829	5.015353	0.5975224	0.9019052
H4c3	0.3225285	-0.7440916	0.5975736	0.9019052
Fzd3	-0.1798672	1.8673122	0.5976289	0.9019052
Rpl36-ps12	-0.2415103	0.4593977	0.5976557	0.9019052
Slc31a1	0.0630235	5.7048133	0.5978413	0.9020908
Tbcc	0.1015386	3.6912857	0.5979585	0.9020908
Gm39859	-0.2876676	0.3965744	0.5979771	0.9020908
Pla2r1	-0.1862166	1.5161613	0.5981069	0.9020908
Taf9b	-0.0945232	3.8460993	0.5981156	0.9020908
Gm8319	-0.2672972	-0.3090156	0.5981267	0.9020908
Fancd2	0.0716288	5.4481611	0.5983721	0.9021207
Sbsn	-0.1589426	1.1551054	0.598535	0.9021207
Zfp385b	-0.2237968	1.3893745	0.598667	0.9021207
Rnps1	0.1559449	5.5564795	0.5987324	0.9021207

Syn3	-0.3284246	-1.1609883	0.5989143	0.9021207
Adcy3	-0.1447138	4.5813749	0.5989438	0.9021207
Espn	0.3619258	-0.2640703	0.5990308	0.9021207
Gm16332	-0.3219874	0.3424515	0.599031	0.9021207
Cxcl10	0.2694338	0.5081569	0.5990388	0.9021207
Gm31560	0.3023738	-0.3959414	0.5990551	0.9021207
Mybl2	0.098816	6.1144329	0.5991932	0.9021207
Pkn1	-0.0717589	7.24222	0.5992001	0.9021207
Stard8	-0.0828674	4.4228902	0.5992803	0.9021207
Pitpnb	-0.0625275	5.935653	0.5993494	0.9021207
Gm10241	-0.1943655	0.1110191	0.59937	0.9021207
Gm30716	-0.136499	1.9388461	0.5994147	0.9021207
Bivm	-0.0933764	3.5479034	0.5994173	0.9021207
Gm20544	0.1274583	2.6895022	0.5994357	0.9021207
Ppargc1b	-0.1185016	3.030846	0.5995185	0.9021207
Tfdp1	-0.0646894	7.9240433	0.5995675	0.9021207
Nfkbib	0.0715035	4.4947394	0.5995848	0.9021207
B930095G15Rik	0.2575576	-0.1135107	0.5996273	0.9021207
Prdx4	-0.0843	5.2862362	0.5996308	0.9021207
Uqcc2	0.0931666	5.1357438	0.5996889	0.9021207
Dipk2a	0.0693165	5.6574675	0.5997952	0.9021207
Abcb7	-0.0636025	6.5474347	0.5998995	0.9021207
Zfp994	-0.1230136	3.5640208	0.600014	0.9021207
Ccdc149	0.1877322	1.6910812	0.600015	0.9021207
Ppp1r10	0.0727298	6.0890664	0.6001038	0.9021207
Slc25a33	0.2072358	1.9121421	0.6002651	0.9021207
Rtn4r	0.1873546	0.7195374	0.6004243	0.9021207
Sdc3	-0.1155522	7.2487985	0.6005195	0.9021207
Zswim8	-0.0609432	5.7615637	0.6005435	0.9021207
Mff	-0.068894	6.6590983	0.600639	0.9021207
Efna4	0.2672094	-0.4100245	0.6006394	0.9021207
Scamp5	-0.0821844	3.617201	0.6006742	0.9021207
Camsap2	0.0762795	6.9563884	0.6007156	0.9021207
Timm50	0.0833024	4.5966805	0.6007219	0.9021207
Lta	0.4451032	-0.6038404	0.6007582	0.9021207
Phldb2	0.1215763	3.4618944	0.6007825	0.9021207
Rab30	0.104372	3.7697861	0.6008167	0.9021207
Gm33248	-0.2957129	-0.7928646	0.6008635	0.9021207
Rnf11	0.1099152	7.1940657	0.6009148	0.9021207
Foxp2	0.2832487	-0.5875924	0.6009488	0.9021207
Trim17	0.1413953	1.3753791	0.6009595	0.9021207
Alkbh3	0.0810593	4.0011724	0.6010461	0.9021207
Stx8	-0.0829851	4.304245	0.6010598	0.9021207

Zfp318	0.1030517	4.8543236	0.6011436	0.9021207
Gm32796	0.3494596	-0.8274865	0.6011507	0.9021207
Ccdc191	0.1183744	2.6003872	0.6012344	0.9021207
Ndufs4	0.0914588	5.9101975	0.6012762	0.9021207
Igkv4-80	-0.4000437	2.4273681	0.6013483	0.9021207
4930431P03Rik	0.2542415	0.6558286	0.601385	0.9021207
Adam12	0.1367946	5.258744	0.6014333	0.9021207
Prrc1	-0.0797461	5.5789713	0.6014589	0.9021207
Plekhg5	0.1108166	3.413181	0.60148	0.9021207
Rhbdl2	-0.2117138	2.0629977	0.6014824	0.9021207
Ctss	0.0662501	7.6406793	0.6015409	0.9021207
Casp8	0.052538	5.9668013	0.6015685	0.9021207
Erg28	0.0692546	5.0314377	0.6016359	0.9021256
Scly	0.0738734	5.239209	0.6016877	0.9021256
Hdac1	0.0670286	7.0383042	0.6019987	0.9022885
Col24a1	-0.2052017	5.3321729	0.6021434	0.9022885
Coro6	-0.3719063	-0.9202261	0.6021912	0.9022885
Pofut1	0.0611431	5.3046153	0.6022296	0.9022885
Gm13778	0.2399007	0.0910613	0.6022389	0.9022885
Slc24a3	-0.0948033	3.5184643	0.6022607	0.9022885
Baiap3	-0.2749925	0.4151579	0.6022745	0.9022885
Igkv8-27	-0.2246608	1.8085707	0.6023616	0.9022885
Dcp1b	-0.1248627	2.8872502	0.6023738	0.9022885
Nck2	0.0984667	4.3639963	0.6024193	0.9022885
Pcdhga1	-0.3628703	-1.1684825	0.6024345	0.9022885
Gpx4	0.0961664	7.8075114	0.6025667	0.9023216
Gm9824	-0.2455599	-0.7415717	0.602613	0.9023216
Vsig10	-0.1395788	1.9505365	0.6026741	0.9023216
Alox12e	-0.3222436	-0.1609983	0.602737	0.9023216
Nnt	-0.1193715	5.5160082	0.6027467	0.9023216
Pdzd4	-0.1719473	2.1775351	0.6029094	0.9023572
Ndufaf1	0.0879011	3.5893115	0.602936	0.9023572
Dap	0.0604538	6.8855439	0.6029739	0.9023572
Med9os	0.3543418	-0.6761188	0.6030506	0.9023572
Smc2	-0.0790596	8.4806547	0.6030605	0.9023572
LOC118568331	0.1399207	1.3350814	0.603168	0.9024312
Dym	0.0620556	5.2793453	0.6033527	0.9026207
M1ap	-0.1218105	2.0640917	0.6036871	0.9030341
Pik3c3	0.1740906	5.6470844	0.60395	0.9033406
Eif3l	0.0623771	6.7739238	0.604067	0.9033622
Dhx37	0.1049584	4.2106192	0.6040806	0.9033622
Mettl7a3	-0.2941972	-0.6767095	0.6042329	0.903382
Gm5564	0.16968	0.8524449	0.6043173	0.903382

Mcm9	0.0812027	4.3439296	0.6043176	0.903382
Slc16a6	-0.0743487	6.5601439	0.6043532	0.903382
Pisd-ps1	-0.0964787	2.9830563	0.6044737	0.903382
Adcyap1r1	0.334287	1.8282771	0.6044776	0.903382
Plcl2	-0.0745009	6.9170481	0.6045005	0.903382
4930430F08Rik	0.0993746	4.4005195	0.6046681	0.9035429
Zscan18	0.2290026	0.1778177	0.6047929	0.9035429
Krtcap3	0.1544262	0.9664092	0.6048049	0.9035429
Trim24	0.0694156	5.3388283	0.6048405	0.9035429
Cdkl5	0.2100336	-0.0164105	0.6050794	0.903813
Zmiz1os1	0.3426804	-0.5660021	0.6052318	0.9038616
Gm40799	-0.2350966	-0.6787232	0.605263	0.9038616
Cdh4	0.2287844	0.1157058	0.6053439	0.9038616
Ggact	0.1313896	2.5812426	0.6053443	0.9038616
Rbks	0.0897539	3.4743126	0.6055146	0.9039478
Zfp574	0.0723743	4.4144701	0.6056339	0.9039478
Alg11	-0.0722641	3.9389996	0.6056974	0.9039478
Ap3b1	-0.0720672	6.382194	0.6057103	0.9039478
Lrrc75b	0.2042559	1.3389388	0.6057147	0.9039478
Tubgcp3	0.0682372	6.181391	0.6057676	0.9039478
Vash2	0.3291252	0.2490822	0.6058208	0.9039478
Lamtor2	-0.0653955	5.7972582	0.605912	0.9039478
Cox5b	0.0569206	7.5119081	0.6059251	0.9039478
Ulk2	0.0764043	5.3298843	0.606067	0.9040171
Amn1	-0.1585178	2.0220411	0.6061079	0.9040171
Naa50	0.0573767	7.7024552	0.6062165	0.9040171
Mink1	-0.072429	5.6173086	0.6062593	0.9040171
Nrgn	-0.0723114	6.2058872	0.6063228	0.9040171
Sdc2	0.1424503	5.4135543	0.606427	0.9040171
Cebpz	-0.0590027	5.9875727	0.606466	0.9040171
LOC115488130	0.2150927	0.5992175	0.6064789	0.9040171
Gm38983	0.1221869	2.8550845	0.6064947	0.9040171
Fcrls	0.2541971	0.9833435	0.606723	0.9042708
Ergic3	0.0650692	5.7950697	0.6072689	0.904494
Wdr76	0.0670614	6.1311683	0.607281	0.904494
Scaf11	0.0683931	7.6613797	0.6073262	0.904494
Zfp493	-0.1770453	1.28944	0.60736	0.904494
Cmtm8	-0.1617614	0.8312281	0.6073784	0.904494
Parn	0.0823491	4.5991567	0.607384	0.904494
Cdc7	0.0710431	5.3691494	0.607406	0.904494
Cdk7	-0.0755177	5.2759265	0.6074423	0.904494
Gm41127	0.3147852	0.0153696	0.607502	0.904494
Slc3a1	0.3254411	-1.0040311	0.607583	0.904494

Vbp1	0.0709687	6.6530313	0.6076168	0.904494
Tceanc	-0.1081614	3.1720268	0.6077166	0.904494
Slc43a2	-0.0786912	5.58479	0.6077564	0.904494
Gm16638	-0.1597522	1.1969254	0.6079026	0.904494
9430091E24Rik	0.2350244	0.7258926	0.6079168	0.904494
Serpinh1	0.1732846	8.3696297	0.6079368	0.904494
Hk1os	-0.2459628	0.7492566	0.60797	0.904494
Dnajc25	0.0803126	3.8616335	0.6080574	0.904494
Pygo2	0.0812881	5.3924338	0.6080918	0.904494
Basp1	-0.0847151	5.0575626	0.60828	0.904494
Arpc3	-0.0697328	7.932703	0.6082983	0.904494
Sox11	-0.2514479	-0.3783014	0.6083205	0.904494
Rpl39	0.1060862	8.84224	0.6083407	0.904494
Igsf10	-0.1714445	3.1219224	0.6084321	0.904494
Cstf2	0.0601553	6.2681113	0.6084834	0.904494
Hsdl1	-0.0736538	5.5941922	0.6085154	0.904494
Bola1	0.1014683	3.8644922	0.6085662	0.904494
Uck1	0.067775	4.7103041	0.6085911	0.904494
Loxl1	-0.1234994	5.292026	0.60863	0.904494
Hexb	0.0785144	7.6817091	0.608638	0.904494
Pianp	0.506459	1.6054843	0.6086755	0.904494
Exoc8	0.0761298	4.8805018	0.6089933	0.9045956
Upk3bl	0.8660088	-0.6236913	0.6089946	0.9045956
Fdx1	0.1180671	2.9571965	0.6090112	0.9045956
Tmem258	-0.0632419	6.1690928	0.6090279	0.9045956
Galnt15	0.1107915	3.0687585	0.6091506	0.9045956
Emd	0.0660139	5.3433033	0.6093488	0.9045956
Igkv8-16	-0.3511241	-0.7723595	0.6093558	0.9045956
Rsl1	0.1216695	2.430511	0.60937	0.9045956
Gm38843	-0.2055145	0.8789918	0.6094298	0.9045956
Stox1	-0.3013627	-0.7503018	0.6094405	0.9045956
2310075K07Rik	-0.2390855	-0.2675524	0.609475	0.9045956
Bola2	0.0950524	5.0280227	0.6094914	0.9045956
Cnpy4	0.0703312	4.787526	0.6095	0.9045956
Bex3	0.0780452	5.7325433	0.6095812	0.9046299
Antxr1	0.0992334	5.8536046	0.6096768	0.9046854
Acsbg1	0.3341657	-0.7642423	0.6097842	0.9047586
Mtmr11	-0.1907694	0.9393487	0.6100538	0.9048577
Miat	-0.3462128	-0.120333	0.6101719	0.9048577
Ciapin1	0.0819301	5.0166485	0.6101751	0.9048577
Tnni3	0.2923483	-0.8829824	0.6102717	0.9048577
Mrps21	0.0678046	5.6358532	0.6103545	0.9048577
Idua	0.1033984	3.6162485	0.6103925	0.9048577

Med22	0.0648131	5.2167003	0.61041	0.9048577
Arglu1	-0.0610676	6.6068485	0.6104344	0.9048577
Pycard	-0.0779869	5.0421621	0.6104426	0.9048577
Zfp78	-0.1747834	0.6421205	0.6105235	0.9048577
Taco1os	0.26943	-0.7260081	0.6105571	0.9048577
Gm16845	-0.1670919	1.2898747	0.6105724	0.9048577
Brcc3	-0.0765553	5.3454322	0.6107881	0.9048577
Bean1	-0.2040569	0.6239004	0.6108555	0.9048577
Hdac9	0.1349447	3.794361	0.6109366	0.9048577
Spindoc	-0.0913986	3.5954504	0.6109754	0.9048577
Arel1	-0.0670799	6.8080298	0.6110205	0.9048577
Pdcd7	0.0734441	4.4099045	0.6110745	0.9048577
Mcrip2	0.1450282	2.3567121	0.61112	0.9048577
Slc4a2	-0.0881925	5.6716329	0.6111266	0.9048577
Gm35959	-0.2102891	0.3981905	0.6112613	0.9048577
Dnaaf4	0.2925242	-0.6211413	0.6112735	0.9048577
Lrch1	-0.0698328	5.1019333	0.6113597	0.9048577
Babam2	-0.0754908	4.8524232	0.6113774	0.9048577
Tatdn2	-0.0632424	5.8697054	0.6115211	0.9048577
G3bp2	-0.0535965	7.1790037	0.6115225	0.9048577
Map3k11	-0.0683301	6.0310936	0.6115331	0.9048577
Gm9616	-0.1086404	2.3842558	0.6115495	0.9048577
Golga1	-0.0893477	4.5654064	0.6116448	0.9048577
Dpp3	0.0621655	5.7121794	0.6116705	0.9048577
Dcdc2b	-0.2871497	-0.4629923	0.611755	0.9048577
2700062C07Rik	0.0984566	3.0701039	0.6117608	0.9048577
Papola	0.0593694	8.1072263	0.6117708	0.9048577
Tinagl1	-0.0660895	5.5226803	0.611968	0.904994
Ndufb2	-0.0952351	4.8764074	0.6119833	0.904994
Zfp248	0.201987	0.7148777	0.6120515	0.904994
Itga5	0.0840891	4.8034993	0.6120957	0.904994
Cbr4	0.1135364	2.8785792	0.6122973	0.9050568
4933440N22Rik	-0.2484103	0.6262082	0.6123071	0.9050568
Gdf9	0.2930539	-0.3208075	0.6125588	0.9050568
Bbs7	0.2584899	1.4897926	0.6126246	0.9050568
Atg16l1	0.069436	4.9836244	0.6126278	0.9050568
Ergic1	-0.0610839	5.2563793	0.6126781	0.9050568
Ly6d	0.2210698	4.6495816	0.612691	0.9050568
Rbfox2	-0.1588401	4.3863	0.6126968	0.9050568
Gm15429	0.2569152	-0.9799296	0.6127984	0.9050568
Rpl14-ps1	0.083793	3.9816866	0.6127985	0.9050568
Chrna1os	-0.3418149	-1.0512387	0.6129426	0.9050568
Heatr3	0.0750569	5.1850003	0.6129955	0.9050568

Pex3	-0.1056781	4.0085351	0.6130322	0.9050568
Slc39a8	0.089953	5.294993	0.6130674	0.9050568
Dclre1c	-0.0636297	4.8562175	0.6130925	0.9050568
Med27	0.0944584	3.1412593	0.613169	0.9050568
Abl2	-0.0774246	5.2224352	0.6131777	0.9050568
Pik3cd	0.0778893	6.8912411	0.6131856	0.9050568
Zfp282	0.0843518	4.1400336	0.6133567	0.9052024
Pcdhga12	-0.3767142	-1.1197389	0.6134436	0.9052024
Cldn5	-0.0944777	3.6702703	0.6135665	0.9052024
Galnt1	0.0623419	7.7047202	0.6135749	0.9052024
Dad1	0.0616718	6.2687876	0.6135752	0.9052024
Phospho2	-0.0723938	4.5013994	0.6139302	0.9055865
Gm36338	-0.3742647	-1.1273323	0.6140183	0.9055865
Auts2	-0.1807291	2.1091336	0.6140513	0.9055865
Commd10	0.0861014	3.7901695	0.6141098	0.9055865
Gm12411	-0.3948126	-0.454819	0.6141267	0.9055865
Chpf2	-0.0636332	5.1188808	0.6142967	0.9057267
Atp8a2	-0.2313655	-0.1824742	0.6143454	0.9057267
Cplane2	-0.3636725	-0.7367489	0.6144963	0.9057267
Dlg4	-0.1474983	2.6972381	0.6145093	0.9057267
Phf2os1	0.14455	2.053015	0.6145408	0.9057267
Cpt2	-0.072446	4.542562	0.6145716	0.9057267
Sts	-0.1198197	2.5844621	0.6146294	0.9057267
Gstm4	-0.113913	2.4964009	0.6147012	0.9057467
Timm23	0.0658263	5.7020813	0.6148577	0.9058916
Bend6	0.2081459	1.1781426	0.6151695	0.9060101
Clstn3	-0.1552074	2.2618516	0.6152774	0.9060101
Galnt7	0.065261	4.8512255	0.6152834	0.9060101
Itgb3bp	0.1124006	2.9919757	0.6153833	0.9060101
Uba7	0.0960448	5.8163468	0.6154138	0.9060101
Scin	-0.1609674	2.1962491	0.615418	0.9060101
Nlrp1b	0.1841692	2.9017904	0.6155291	0.9060101
Ssr4	-0.059936	6.8342646	0.6155635	0.9060101
Phactr1	0.1510064	0.8772278	0.6155676	0.9060101
Zscan20	0.1245418	2.0235263	0.6155688	0.9060101
Amz2	0.073419	4.8350776	0.6156301	0.9060101
Nol9	0.072365	4.8591298	0.6156372	0.9060101
P2ry10	0.1485344	3.5857564	0.6157384	0.9060733
Ldlrad3	-0.1311513	3.678868	0.6159594	0.9062601
L3hypdh	0.2128838	1.5098802	0.6159859	0.9062601
Il17d	0.3019341	0.3672402	0.6165671	0.9062601
Dkk3	-0.1721513	2.3906975	0.6165992	0.9062601
Zfp882	0.108888	2.8570674	0.6165992	0.9062601

Gm40696	0.2017761	1.191395	0.6166493	0.9062601
Vps9d1	-0.0898939	3.71197	0.6167823	0.9062601
Npdc1	0.1455916	3.3877736	0.6168662	0.9062601
Pik3c2b	-0.0878573	4.9163009	0.6169417	0.9062601
Tlk2	-0.0533124	5.8769219	0.6169423	0.9062601
Klc4	-0.0789802	4.1690436	0.6169822	0.9062601
Particl	0.2062232	1.6039	0.6169846	0.9062601
Pi4k2b	0.0866372	6.124666	0.6169968	0.9062601
Slc29a3	-0.0715306	4.9038016	0.6170167	0.9062601
Gsta4	0.1836061	2.4686354	0.6170337	0.9062601
Rpl27-ps3	-0.2127589	-0.3600886	0.617068	0.9062601
Poglut2	-0.0823991	4.3315811	0.6170994	0.9062601
Fam160a1	-0.1731802	1.3997795	0.617111	0.9062601
Rps27a	0.1012807	9.6018084	0.6171389	0.9062601
Gm5820	-0.3020162	-0.4203517	0.6172369	0.9062601
Fam151b	0.139138	1.7041771	0.6172967	0.9062601
Stc1	0.1785591	1.7761125	0.6173036	0.9062601
Gm7972	-0.3090308	0.1563126	0.6173765	0.9062601
Emc3	0.0531602	5.965055	0.6174071	0.9062601
Zfp131	0.0781988	6.2433921	0.6174503	0.9062601
Chek1	0.0679984	5.3584077	0.6174801	0.9062601
Pard6b	0.3093549	-0.3288626	0.6175553	0.9062601
Pithd1	0.074296	5.5831974	0.617658	0.9062601
Zfp398	-0.0829027	4.2256415	0.6176742	0.9062601
Opa3	-0.0960098	4.0093147	0.6177192	0.9062601
Larp4	-0.0618793	6.5684925	0.6177473	0.9062601
Pum3	0.0633057	5.8642104	0.6177603	0.9062601
Dnase1l2	0.1945296	1.5749653	0.6177963	0.9062601
Ppp1r13l	0.1790936	1.6777908	0.6178797	0.9062601
Sdcbp	-0.0677779	8.6856123	0.6179281	0.9062601
Dennd6b	0.1690194	1.6299282	0.6179629	0.9062601
Atg4a-ps	-0.2392898	-0.2728815	0.6180778	0.9063432
Fam221a	-0.2181611	0.7155935	0.6181786	0.9064055
Gm4202	-0.2074479	1.2801277	0.6183418	0.9064307
Matn2	0.2248529	1.0633669	0.6183663	0.9064307
Selenoh	-0.0719946	4.8784201	0.6184193	0.9064307
Fis1	0.0745002	7.1053459	0.6184397	0.9064307
Mafk	0.0754008	5.7630477	0.6185291	0.9064307
Wsb2	-0.0617201	4.9129702	0.6185668	0.9064307
Max	-0.0541576	7.1744373	0.6186037	0.9064307
Ckap2l	0.0664988	7.2414966	0.6188373	0.9066239
Gm39008	0.2524094	-0.8801177	0.6188521	0.9066239
LOC118568302	0.1847991	2.3445253	0.6189509	0.9066833

Uba2	0.0596424	7.0659523	0.6190393	0.9067193
Sall2	0.1490342	1.9199645	0.6191246	0.9067193
Tsen54	0.1103012	3.0387405	0.6193039	0.9067193
Brd3os	-0.4272403	-0.2847954	0.619487	0.9067193
Cyp11a1	-0.1611529	0.727212	0.6194984	0.9067193
Amd1	0.0736831	7.1748701	0.6195322	0.9067193
Cmtr2	-0.1155657	2.7874548	0.6195608	0.9067193
Flii	-0.0721407	6.9964895	0.6196691	0.9067193
Bcr	-0.1043111	5.3770065	0.6197764	0.9067193
Dipk1a	0.0745736	4.8156609	0.6198489	0.9067193
Tnfrsf18	-0.123952	1.7930611	0.6198875	0.9067193
Gm6969	-0.0639203	5.5495952	0.6199347	0.9067193
Arrdc1	0.0764113	4.9467027	0.6199355	0.9067193
Rbbp4	0.0580553	8.4337818	0.6199452	0.9067193
Zfp595	-0.0975416	3.611278	0.6199673	0.9067193
Elovl5	-0.0691403	6.4092767	0.6200461	0.9067193
Mydgf	0.0861599	5.3028457	0.6201093	0.9067193
Abce1	0.0646242	7.2995135	0.6201219	0.9067193
Cenpe	-0.0664648	7.5800044	0.6202143	0.9067193
Tmem251	0.0950987	4.0078586	0.6202507	0.9067193
Cdh13	0.2827804	0.9843254	0.6202515	0.9067193
Zkscan14	0.0903214	3.3522753	0.620258	0.9067193
Gm12183	0.1189678	2.1292586	0.6203623	0.9067866
Bcl3	-0.1218178	4.1051904	0.6205913	0.9068947
Rnmt	0.0701369	5.5560874	0.6206092	0.9068947
Ikbkb	0.0613156	5.9990026	0.6206911	0.9068947
Zfp454	0.5246134	-1.3436599	0.6207148	0.9068947
Nsmf	0.1013982	3.377981	0.620767	0.9068947
Rps16	0.0688055	9.1653334	0.6207862	0.9068947
Kdm5c	0.0625752	7.2904842	0.6208762	0.9069105
Gm33171	0.237248	-0.1313396	0.6209467	0.9069105
Efl1	0.0845802	4.0614878	0.6209719	0.9069105
Ap1s1	0.0599804	5.5555762	0.6210476	0.9069127
Arrdc4	-0.0749989	5.6504216	0.62109	0.9069127
Fbxo30	0.0993811	6.8480985	0.6213974	0.9072094
Firre	-0.1093771	4.1858821	0.621424	0.9072094
Eci2	0.0650656	5.5774921	0.6214682	0.9072094
Rap1gds1	-0.0670015	5.9530415	0.6217392	0.9075078
Tac2	0.1899225	2.6977237	0.6217893	0.9075078
Tle5	-0.0599905	7.7623426	0.6218946	0.9075763
Ap2m1	-0.0615644	7.4602798	0.6220558	0.9076407
Zfp775	0.1704149	0.7158007	0.6220812	0.9076407
Fbxw17	-0.1369351	2.2981233	0.6222913	0.9076407

Atp13a3	-0.0624589	7.571407	0.6223161	0.9076407
Polr3d	0.0950339	3.8786433	0.6223305	0.9076407
Hmgxb4	-0.060555	5.32919	0.6223651	0.9076407
Stam	-0.061302	4.7916738	0.6223829	0.9076407
Ddx46	-0.05698	7.2376092	0.6224347	0.9076407
Odf2l	-0.0802968	3.9421676	0.6224639	0.9076407
Rdh10	0.0972167	3.2895183	0.622852	0.9080868
COX3	0.1094824	4.668953	0.6228902	0.9080868
Tasl	-0.1045113	3.0967146	0.6229985	0.9080868
Gm6545	-0.2180044	0.4060258	0.6230428	0.9080868
Rpl36al	0.079864	6.3286583	0.623104	0.9080868
Rbm12	0.0761943	5.8599833	0.6231201	0.9080868
Yju2	0.082212	3.3411429	0.6231933	0.9081083
Ptges2	0.0847025	3.6293951	0.623308	0.9081905
Nup37	0.0787586	3.92475	0.6234276	0.9082795
Gm4130	-0.3472544	-0.3160154	0.6235221	0.9082808
Usp4	0.0586792	6.897782	0.6235486	0.9082808
Socs3	-0.1292129	4.4032263	0.6236036	0.9082808
Gdi1	0.0641889	7.1133687	0.6238314	0.9085275
Irf2bp2	-0.0587007	7.3787473	0.6240094	0.9087016
St14	0.1453179	2.1155346	0.6243498	0.9088281
Mcl1	-0.0662068	8.8726324	0.6243521	0.9088281
Rab40b	0.2596443	0.0526181	0.6244048	0.9088281
Zfp266	-0.0580855	5.2191463	0.6244475	0.9088281
Ddx59	0.1284605	3.3532526	0.6244708	0.9088281
Ccng1	-0.0602846	6.4253381	0.6246168	0.9088281
Nedd8	0.0663242	6.8022025	0.624661	0.9088281
Gm51557	-0.1958651	0.3603222	0.624711	0.9088281
Gm7308	-0.1889356	-0.0299223	0.6247448	0.9088281
Vdac3-ps1	0.1642096	1.0394137	0.6248011	0.9088281
Prelid1	0.0576238	7.0614234	0.6248061	0.9088281
Zbtb44	0.0682742	5.5653428	0.624923	0.9088281
Gucd1	0.0845368	6.5622554	0.624947	0.9088281
Tmc3	-0.2285623	0.0262643	0.6250698	0.9088281
Ldb2	0.1080431	3.0319002	0.6251541	0.9088281
Pcmtd2	-0.0538359	6.3563168	0.6252045	0.9088281
Tchp	0.1044682	3.335164	0.6253185	0.9088281
Chmp3	-0.0605714	6.7370953	0.6253446	0.9088281
Tmem176a	0.0913748	5.9471416	0.6253714	0.9088281
Dusp8	0.1026884	3.961011	0.6254324	0.9088281
Kpna3	-0.0570673	6.4885459	0.6254523	0.9088281
Fgd6	-0.0802504	4.0860031	0.6254582	0.9088281
Epb41l5	0.1736107	1.6763429	0.6255036	0.9088281

H2-Eb2	0.2077619	2.2443257	0.6255115	0.9088281
Gm5421	0.2857418	-0.6634924	0.6255863	0.9088281
Aqp7	0.2741688	1.4491125	0.6256154	0.9088281
Gimap6	0.0980201	5.5965613	0.6259062	0.9091655
Zbtb7c	-0.2139495	2.9136065	0.6260053	0.9092246
Ppm1k	0.0933065	3.5980163	0.6261623	0.9092507
Spsb2	-0.0947633	3.1720005	0.6262985	0.9092507
Gm51535	0.2557675	0.4584674	0.6263414	0.9092507
Gm13421	-0.161759	1.7086421	0.626354	0.9092507
Tspyl1	-0.049587	6.0606011	0.6264211	0.9092507
Col11a1	-0.1840436	9.4851299	0.626506	0.9092507
Ubap1l	0.3847136	-1.0403427	0.6265999	0.9092507
Zkscan4	-0.227015	-0.1778832	0.6266576	0.9092507
Cnep1r1	0.0756003	5.1743441	0.6267102	0.9092507
Rps12-ps3	-0.12864	1.6172216	0.6267274	0.9092507
Ubr4	-0.0697967	7.386068	0.6268262	0.9092507
Mpzl1	-0.0878555	4.4067974	0.6268296	0.9092507
Gm17745	0.3070599	-0.3076314	0.6268747	0.9092507
Lrrc29	0.1621581	1.8071916	0.6268777	0.9092507
Clic4	0.0616225	8.1138865	0.6269001	0.9092507
Ccl9	-0.0967442	6.1469195	0.6270465	0.9093063
Zfp593	0.1055515	2.9182328	0.6270999	0.9093063
Zfp518b	-0.1209986	3.2985822	0.6271139	0.9093063
Rdx	-0.0536628	7.5201475	0.6273138	0.9094515
Tmem131	-0.080688	7.2794776	0.6273309	0.9094515
Paxx	-0.1157368	3.364654	0.6274234	0.9095008
Gm26947	0.2324397	0.2043388	0.6276021	0.9095413
Tgfbr3	-0.0709383	5.4492349	0.6276164	0.9095413
Ssc5d	0.301645	-0.6357082	0.6276676	0.9095413
Idh3b	0.0547698	6.5587628	0.6276852	0.9095413
Fgf2	-0.1906905	0.1679722	0.627822	0.9096547
Ttyh3	-0.0706132	6.9114306	0.6279794	0.9097672
Il4ra	0.0713016	5.6675933	0.6280166	0.9097672
Slc39a1	0.0920266	5.8238526	0.6281376	0.909795
Iws1	-0.0506601	6.1967818	0.6281628	0.909795
Fbxl4	-0.1132253	2.7288637	0.6282113	0.909795
Prr15	0.2712501	1.1125194	0.628363	0.9098525
Ddx18	0.0613345	5.382276	0.628368	0.9098525
Dnajc10	-0.0600831	6.7906106	0.6284716	0.9098699
Galns	-0.0708629	5.2874501	0.6287042	0.9098699
Spef2	0.2229837	0.635512	0.6287622	0.9098699
Zfp764	0.1470976	2.3381315	0.6288056	0.9098699
Ubxn11	-0.1636934	1.3524437	0.6288686	0.9098699

Zfp768	0.1065172	3.2490001	0.6288721	0.9098699
Entpd7	0.0846392	4.0561312	0.6289525	0.9098699
Zranb1	0.0636946	5.9081506	0.6290725	0.9098699
2610301B20Rik	0.0795331	3.7192069	0.629189	0.9098699
Rwdd1	-0.0663587	4.7538246	0.6293341	0.9098699
Creld1	0.0964766	3.250945	0.6293714	0.9098699
Igflr1	0.1955315	1.4904684	0.6294143	0.9098699
4930426L09Rik	0.2232009	0.0488234	0.6294946	0.9098699
Ctdsp2	-0.0716205	6.9103741	0.6295716	0.9098699
Zfp119b	-0.1817906	2.2185987	0.6295752	0.9098699
Cep164	0.0628571	5.1328032	0.6296264	0.9098699
Rpl21	0.1413553	9.5493145	0.629741	0.9098699
Mapkapk5	0.0587871	5.10226	0.6297783	0.9098699
Acot2	0.1077909	2.3990148	0.6298497	0.9098699
Akt1	0.0619986	7.2929164	0.6298745	0.9098699
Atad1	-0.0534414	6.1208723	0.6298868	0.9098699
Elf1	0.0746715	6.999277	0.6299431	0.9098699
Tnrc6a	-0.0501357	6.7765936	0.6300609	0.9098699
Mmd	0.0702677	5.6430351	0.6300904	0.9098699
Ifngr1	-0.0595034	7.0741559	0.6300921	0.9098699
Acvr1b	-0.1064305	4.2020186	0.6301011	0.9098699
Pinx1	-0.0801466	3.2608367	0.6301406	0.9098699
Tex261	0.0610753	5.6642271	0.6301696	0.9098699
Mettl2	0.0804105	3.8121259	0.630292	0.9098699
Malsu1	-0.0999169	3.4382269	0.6303507	0.9098699
Septin1	0.0694523	5.6129502	0.6303628	0.9098699
Fgf11	0.1759371	1.2227866	0.6304103	0.9098699
Phf20	-0.0673968	4.6391481	0.6304305	0.9098699
Klhl6	0.0940656	6.5446016	0.6304588	0.9098699
Gm15772	-0.3429589	8.2431477	0.6304603	0.9098699
Wdr31	-0.2768021	-0.9707066	0.6304859	0.9098699
Crnkl1	0.0566071	6.2109333	0.6306927	0.9099814
Echdc3	0.2731391	-0.8082852	0.630709	0.9099814
Kiss1r	-0.123768	1.3281266	0.6308095	0.9099814
Mrps18c	0.0818461	5.558463	0.6308235	0.9099814
Eldr	0.1121569	2.9466533	0.6308557	0.9099814
2810408A11Rik	0.136729	1.4894152	0.6310755	0.9100375
Ndc80	0.0763884	6.0782173	0.6310982	0.9100375
Pcdhgc4	-0.298171	-0.6159396	0.6311156	0.9100375
Tex15	-0.1691012	4.0701483	0.6312038	0.9100375
Zfp41	-0.0902817	3.5105317	0.6312114	0.9100375
Pdcd4	0.0585929	6.9599522	0.6312457	0.9100375
Kdm3b	-0.0598962	6.8245329	0.6313511	0.9100839

Ttc12	-0.2095239	0.8556726	0.6313949	0.9100839
Wdsub1	0.0783459	3.6778831	0.6315468	0.9102178
Polr3g	0.1079824	3.4432293	0.6316048	0.9102178
Smim26	0.1244927	2.8012209	0.6316648	0.91022
Fam162a	0.078209	4.9165369	0.631803	0.910265
Adat2	-0.1228713	2.0174221	0.63188	0.910265
Gm36774	-0.164302	1.1743257	0.6319456	0.910265
H3f3a-ps2	0.1865151	1.1729966	0.6320244	0.910265
Mrpl3	-0.0674831	5.0341496	0.6321665	0.910265
Sephs2	0.0597424	6.4249208	0.6324307	0.910265
Gm15523	-0.2739997	-0.1062888	0.6324554	0.910265
Mettl22	0.1142657	1.948972	0.6325262	0.910265
Gm9762	-0.1103037	2.4629033	0.6325599	0.910265
Syngap1	0.1095355	3.2597053	0.6325808	0.910265
Wdr47	-0.0857589	2.917719	0.6325876	0.910265
Cog6	-0.0608597	5.0037908	0.6326577	0.910265
Slc6a2	0.2245545	0.9303952	0.6326793	0.910265
H6pd	-0.0704431	5.7462146	0.6328581	0.910265
8430429K09Rik	-0.135239	1.8291589	0.6328719	0.910265
Tmem59	0.0676998	7.4459974	0.6328934	0.910265
Stx2	0.0774527	6.7635356	0.6329876	0.910265
Usp18	-0.1271158	3.1834251	0.6331329	0.910265
N4bp2l1	-0.0916864	4.1986461	0.6331958	0.910265
Zfp626	0.0796862	4.0470358	0.6332404	0.910265
Map10	0.1718551	1.0374788	0.6332577	0.910265
Klra9	0.2302179	-0.0218517	0.6333234	0.910265
Zfp866	-0.0568472	5.3617864	0.6333375	0.910265
Gaa	-0.0613377	5.5092036	0.6333856	0.910265
Cdip1	0.0837249	4.3231412	0.6334118	0.910265
Tmem120a	-0.0996318	3.5086521	0.6334434	0.910265
Ppp4r2	-0.0533538	6.2792951	0.6335093	0.910265
Zfp566	0.1668459	1.523783	0.6335744	0.910265
Slc35f5	-0.0968152	3.8864432	0.6336417	0.910265
Tfg	-0.0634597	6.0600937	0.6336876	0.910265
Cox7b	-0.0723028	7.1619033	0.6337061	0.910265
Gm46800	-0.295573	-0.4716484	0.63371	0.910265
Star	0.219992	-0.164672	0.6337297	0.910265
Magohb	-0.1058572	3.6927385	0.6337898	0.910265
Gm46731	0.3191153	-0.1283633	0.6338458	0.910265
Ccnd1	0.1245636	4.3999353	0.6338959	0.910265
Hccs	0.0689871	4.6894928	0.6340262	0.910265
Taf1c	-0.0812397	3.709492	0.6340312	0.910265
Pms1	-0.0952981	3.5519309	0.6341516	0.910265

Cenpk	-0.1033701	5.1596214	0.6341597	0.910265
Btbd1	-0.0488193	6.2338789	0.6342043	0.910265
Wdr25	0.1563307	1.4959021	0.6342109	0.910265
Nkapd1	-0.0610664	4.6570703	0.6342296	0.910265
Smarcal1	0.0625244	4.868016	0.6342711	0.910265
Bbs10	0.2060179	0.9485291	0.6343316	0.9102679
Zfp771	0.0867478	3.4969614	0.6344103	0.9102969
5730409E04Rik	0.1871276	2.2230774	0.634556	0.9103457
Dmac1	0.0765466	4.7656412	0.6345614	0.9103457
Chd1	-0.0672374	7.1226307	0.6348827	0.9107226
Ncs1	0.2538851	-0.3567796	0.6349829	0.9107824
Klhl23	-0.0734875	4.7691955	0.6350673	0.9107836
Spaca1	-0.4601808	-0.6606422	0.6351533	0.9107836
Sun1	-0.0643239	5.8681955	0.6351594	0.9107836
Myof	-0.0839338	4.4385929	0.6352885	0.9108577
Cptp	-0.0826676	3.7695044	0.6353441	0.9108577
Scn1b	-0.0995188	3.042369	0.6354397	0.9108577
Gm14326	-0.075923	3.9977195	0.6354453	0.9108577
Adam17	-0.0546197	6.3236361	0.6358562	0.9111037
Brd4	0.0664547	7.4072399	0.6359732	0.9111037
Golm1	0.0629961	6.1725625	0.6359766	0.9111037
Phldb1	-0.1051215	4.250361	0.6359796	0.9111037
Chac1	-0.1872711	0.5877823	0.6360262	0.9111037
Them4	0.2233477	0.9586607	0.6360849	0.9111037
Mfsd10	-0.0648943	5.2231152	0.6361098	0.9111037
Sdha	0.0560873	7.7906866	0.6361428	0.9111037
Senp7	-0.0529014	5.7326499	0.6362665	0.9111037
Zfp37	-0.2126707	0.2419931	0.6363179	0.9111037
Calcoco1	-0.0527145	5.3126933	0.6363221	0.9111037
Ptk2	-0.0692994	4.5335935	0.6363925	0.9111037
Card11	0.1065532	3.5385198	0.6364593	0.9111037
Zw10	0.0637514	5.5471708	0.6365909	0.9111037
Btbd10	-0.072729	4.7772062	0.6367056	0.9111037
Mrpl45	0.0641409	4.7446913	0.6367214	0.9111037
LOC118567850	-0.2527294	-0.7646579	0.6367527	0.9111037
Glb1l2	-0.3086518	-0.460941	0.6368505	0.9111037
Ubb-ps	-0.0708267	6.1445726	0.6368532	0.9111037
Map2k6	-0.0821951	4.1630216	0.6369176	0.9111037
Ankrd63	-0.2176834	-0.0739836	0.6369594	0.9111037
Soat1	-0.0898709	5.9454741	0.6369746	0.9111037
Pcdhb3	-0.2225716	-0.6893225	0.6370004	0.9111037
Smyd3	0.0879296	3.4091611	0.6370507	0.9111037
Ddb1	0.0647758	8.1525038	0.6371052	0.9111037

Thoc2l	-0.0827408	4.722104	0.6372785	0.9111037
Guca1b	0.348204	-0.4801671	0.6373006	0.9111037
Enoph1	0.0930831	3.5806774	0.6373423	0.9111037
Dip2b	0.0682167	5.1249523	0.6374159	0.9111037
Lrrc28	-0.1103658	3.0115772	0.6374323	0.9111037
Cars2	0.0728531	3.8000282	0.6374765	0.9111037
Gltp	-0.0552531	6.1943425	0.6375537	0.9111037
Rabep1	-0.0553266	5.4956931	0.6376271	0.9111037
Trmt10a	0.1044147	3.2209434	0.6376571	0.9111037
Orc3	-0.055797	5.7233466	0.6376935	0.9111037
4921531C22Rik	-0.1572472	1.2385309	0.6377257	0.9111037
Gm7609	-0.3428959	-0.5509802	0.6383476	0.9118177
C1s1	0.1256156	4.756703	0.6383676	0.9118177
Nop58	0.0678151	7.257583	0.6384428	0.9118177
Gm26510	-0.4021085	-1.4226192	0.63846	0.9118177
Gm50357	-0.2776839	-0.1034744	0.6385281	0.9118178
Socs1	0.1780017	1.879254	0.6385773	0.9118178
Tspan31	0.0643147	5.3043845	0.6386422	0.9118267
Gm4924	0.1457534	1.3679883	0.6387452	0.9118901
Dlg3	0.0843031	3.138041	0.6388688	0.9119711
Sec24d	-0.0985109	5.5250951	0.6390101	0.9119711
Stk32c	-0.2399434	0.2212057	0.639029	0.9119711
Zfp213	0.0949931	2.8531666	0.6390365	0.9119711
Srp68	0.0634689	6.3542957	0.6391555	0.9119973
Rpl15	0.0623395	8.226825	0.6391721	0.9119973
Virma	-0.0622658	6.4000247	0.6394021	0.9120454
Lrrc61	0.1059557	3.3389402	0.6395346	0.9120454
Ebna1bp2	0.0629587	5.945642	0.6395615	0.9120454
Ank2	-0.1328031	1.8821111	0.6395642	0.9120454
9330020H09Rik	-0.1097332	1.9809681	0.6396044	0.9120454
Txnrd1	-0.05318	6.5253525	0.639682	0.9120454
Crcp	0.0803169	4.8326508	0.639738	0.9120454
Hrh2	-0.0784698	3.7580298	0.6398054	0.9120454
Rundc3a	0.3118801	-0.9509207	0.6398271	0.9120454
Tex264	0.0859909	3.9439798	0.6398473	0.9120454
Rrm1	0.0737546	8.9201801	0.639854	0.9120454
Gm11769	0.2781328	-0.1355117	0.6399117	0.9120454
Dhx15	-0.0654884	7.8157421	0.6399681	0.9120454
Zfp786	-0.2728203	-0.5043816	0.6401583	0.9121078
Gm41804	0.1980698	-0.5029706	0.6401773	0.9121078
Stx16	0.0604047	5.8436923	0.6401878	0.9121078
Cd244a	-0.0970533	3.1757348	0.6403783	0.9122741
Szt2	0.0584167	5.5737263	0.6404699	0.9122741

Akt1s1	-0.0617345	4.6673164	0.6404805	0.9122741
Parp16	0.0813801	3.6858383	0.6409611	0.912558
Sp110	-0.0660429	5.6965028	0.6410316	0.912558
Tmem117	0.2261737	1.6198111	0.6410388	0.912558
A930007A09Rik	-0.2506495	-0.1236384	0.6410427	0.912558
Nsmce1	0.0668162	5.4157316	0.6410852	0.912558
Coq5	0.0717217	4.3063298	0.6411017	0.912558
Gm46775	0.1934135	0.3268189	0.6411276	0.912558
Bach2os	0.3229544	-0.8191051	0.6412015	0.912558
Phf6	-0.0561318	5.6356855	0.641253	0.912558
Gm9830	0.5638321	-0.5144005	0.6412665	0.912558
Aknad1	0.16128	2.1870802	0.6414087	0.9126296
Phf13	-0.1167182	2.9302773	0.6415989	0.9126296
Uri1	0.0584912	5.4100619	0.6416344	0.9126296
Phf8	0.0572001	5.591071	0.6416346	0.9126296
Fam83h	-0.30128	-0.1870475	0.641666	0.9126296
Comtd1	0.0837054	3.314942	0.6416689	0.9126296
D8Ertd738e	-0.0518637	6.4232916	0.64182	0.9127611
Atp1a1	0.0583988	8.1849153	0.641905	0.9127986
Skil	0.0857124	6.0429399	0.6420709	0.9129276
Strn4	0.0500997	6.0188629	0.6421132	0.9129276
Dbnl	-0.0650315	6.8234407	0.6422137	0.912987
Adh6b	-0.1947713	1.7587962	0.6422913	0.913014
Zfp93	-0.1582939	0.5435381	0.642436	0.9131362
Synpo2	0.198835	2.360834	0.6426299	0.9133038
Snx21	0.1342392	2.3224356	0.6426714	0.9133038
Sycp2	-0.1379149	2.6088796	0.6428541	0.9134737
Fastkd5	0.070241	3.9630728	0.6430681	0.9134737
Exoc4	-0.0680869	5.6387174	0.6430805	0.9134737
Ndufs8	0.0618093	6.0560154	0.6431036	0.9134737
F2r	-0.0849845	5.244536	0.6431308	0.9134737
Ccnk	0.0597248	5.7331935	0.6431433	0.9134737
Yars2	0.0947176	3.9291851	0.6433558	0.9136893
Rhbdl1	0.1983454	0.9730569	0.6434126	0.9136893
Rpl32	0.0635679	9.0694442	0.6435002	0.9137303
Tspan6	0.1256457	3.1099079	0.6435617	0.9137343
Slc25a32	0.0670646	4.5082602	0.6437513	0.9138806
Smpd3	-0.1493875	6.2050678	0.6437823	0.9138806
Ptpn7	-0.0647819	6.2754865	0.6439052	0.9139491
Trip10	-0.1041249	3.4553986	0.6439481	0.9139491
Lamtor3	0.0602191	5.3247103	0.644128	0.9140714
Gas2	0.1050469	2.4579705	0.6442531	0.9140714
Cep192	-0.0739129	6.3447855	0.6442632	0.9140714

Csf2ra	-0.0664787	6.921936	0.6443205	0.9140714
St3gal3	-0.0813939	3.6044236	0.6443395	0.9140714
Tspan32	-0.0591683	5.5107006	0.6444093	0.9140714
Rita1	-0.1449135	1.4664386	0.6444565	0.9140714
Prkar1b	-0.1484848	1.112516	0.6445044	0.9140714
Zfp773	-0.1569525	1.0621258	0.6445678	0.914078
Cnpy2	0.0778475	5.7374142	0.6446749	0.9141466
Zfp655	-0.0580449	5.2222825	0.6449374	0.9144233
Pls3	-0.1367909	5.2610408	0.6449876	0.9144233
Gm31513	0.2799769	-0.4115358	0.6450746	0.9144483
Gm36109	0.2830518	-0.5637108	0.6451229	0.9144483
5730460C07Rik	-0.2361096	0.0293748	0.6452239	0.9144817
9130023H24Rik	0.0910935	2.815988	0.645264	0.9144817
Marcks	0.0670673	7.9750387	0.6454369	0.9146414
Sipa1l3	-0.078757	4.545571	0.64551	0.9146414
Uaca	-0.1096631	4.1429572	0.6455531	0.9146414
Armcx1	0.0961591	3.253612	0.645805	0.9148038
9130002K18Rik	-0.3103532	-0.0994178	0.645838	0.9148038
Nectin4	-0.1537905	1.0516368	0.6458565	0.9148038
Timp1	0.1863154	3.3411212	0.645925	0.9148038
Psd3	-0.0869736	5.1327644	0.6459618	0.9148038
Zfp324	-0.0999884	2.7302871	0.6460558	0.9148535
Nacc1	0.0610402	6.4857519	0.6462111	0.914972
Ifnar1	0.0476455	6.0795181	0.6462611	0.914972
Lrif1	-0.0643334	4.7443792	0.6463616	0.914972
E030042O20Rik	-0.2665668	-0.860223	0.6463747	0.914972
Casp12	-0.171992	2.1586534	0.6465162	0.915089
Odr4	-0.061296	5.2706055	0.6466406	0.9151818
Tcea1-ps1	-0.1738623	1.1069548	0.6468464	0.9152487
Vav1	0.0702362	6.9622543	0.6469391	0.9152487
ATP8	0.1040953	2.3981195	0.6469823	0.9152487
Nfya	0.0664277	6.7287278	0.6470572	0.9152487
Hnrnpa3	0.0560505	9.9268975	0.6470801	0.9152487
Zrsr1	-0.1026946	5.3526436	0.6472023	0.9152487
Gm15353	-0.3730093	-0.6574578	0.6472509	0.9152487
Psme4	-0.0514909	7.1455096	0.6472967	0.9152487
Ifi213	0.1529429	2.5596649	0.6475225	0.9152487
Gm10658	-0.2096297	0.4302036	0.6475594	0.9152487
Slc13a5	0.2054541	5.755708	0.6475631	0.9152487
Sumo3	0.0499881	6.8942401	0.6476402	0.9152487
Ncbp3	0.0509182	5.6597143	0.6477024	0.9152487
Nepro	-0.0761551	4.0901785	0.6477721	0.9152487
Xpnpep3	-0.0642653	4.1837075	0.6478412	0.9152487

Stx5a	0.0538856	5.8794031	0.6478945	0.9152487
Zfp942	0.0687454	4.6368878	0.6479081	0.9152487
Poc1a	0.0805334	4.1851888	0.6479115	0.9152487
Gm6325	0.4061147	-0.3928857	0.6479569	0.9152487
Snx16	-0.1373443	1.794936	0.6479823	0.9152487
Mynn	0.0598617	5.0926861	0.648021	0.9152487
Gabpb2	0.0678708	7.4948792	0.6480304	0.9152487
Tap2	-0.0530703	5.8438168	0.6480413	0.9152487
Drg1	0.0532403	5.9063441	0.648173	0.9153048
Slc39a3	-0.0787259	3.6945884	0.6482433	0.9153048
Chek2	0.0706796	4.957365	0.6482575	0.9153048
Timm8a1	0.1118211	2.9885599	0.6484205	0.9154518
S100a10	0.0692577	6.5451707	0.6486407	0.9155712
Cobl	-0.304662	-0.6570476	0.6486653	0.9155712
Atp1a2	-0.2058292	2.0386481	0.6487875	0.9155712
Selenok	-0.0701871	6.020055	0.6488229	0.9155712
Car8	0.2200921	1.052581	0.6488334	0.9155712
Zfp568	-0.0781303	4.619196	0.648904	0.9155712
Psmb8	0.0610331	6.4099775	0.6489456	0.9155712
Ccdc88c	-0.0652739	7.3701396	0.648976	0.9155712
Arid5b	-0.0648702	4.6488317	0.6492554	0.9158823
Mrpl22	0.0701803	4.5345683	0.6493578	0.9159309
Serbp1	0.0625795	9.400856	0.6494076	0.9159309
Slc31a2	-0.06158	5.353851	0.6495797	0.9159443
Slc38a1	0.077995	6.39633	0.6495883	0.9159443
Mbtd1	0.0588793	5.5500269	0.6496533	0.9159443
Mirt2	0.1392025	2.8595697	0.6498571	0.9159443
Nhs	0.1664404	1.1670478	0.6498618	0.9159443
LOC118567920	-0.2177732	-0.2631084	0.6498678	0.9159443
Mex3b	-0.1173469	2.8810093	0.6498969	0.9159443
Gm33937	0.206272	-0.1343578	0.649963	0.9159443
Pus7l	0.0941356	2.89356	0.6499738	0.9159443
Mbd6	0.059528	6.4520373	0.650006	0.9159443
Tmem106a	0.0806693	3.734472	0.6500886	0.9159777
Retreg1	-0.0703372	5.2894947	0.6502158	0.9160229
Ttl	-0.101741	2.5705647	0.650277	0.9160229
C1d	-0.0763518	5.6321926	0.6502974	0.9160229
Dynlt1c	0.1457923	1.6990413	0.6504344	0.9161329
Tstd2	0.0586261	4.8603884	0.6505969	0.9162789
Tsc22d4	-0.0552016	7.1739118	0.6507242	0.9163003
Aoc2	-0.0886045	3.6749568	0.6507633	0.9163003
Fbxl3	-0.0605444	6.3871909	0.6508148	0.9163003
Kctd21	0.1328445	1.9051585	0.6509064	0.9163003

Maged1	-0.1198946	6.2802159	0.6509066	0.9163003
Gm12966	0.157197	1.391615	0.6510539	0.9164247
Abhd8	0.0943006	3.4726122	0.6511262	0.9164435
Fgf7	-0.1028239	2.9156519	0.6512702	0.9165633
Apex2	-0.0796578	3.706087	0.6514441	0.9166901
Tbc1d22a	-0.0785891	4.9603797	0.6515263	0.9166901
Mlxip	0.0846563	6.0997701	0.6515815	0.9166901
Gm41056	-0.1959595	1.1235633	0.6516024	0.9166901
Tsc2	0.0620287	5.3058982	0.6517692	0.9166901
Bzw1	0.050592	8.2581543	0.6517873	0.9166901
Mmaa	-0.0969815	3.1376307	0.6518073	0.9166901
Tgfb1i1	-0.0672217	4.9112546	0.6519091	0.9166901
Gm6407	-0.5780773	-0.3010332	0.6519115	0.9166901
Aebp1	0.1833308	3.9761831	0.6519497	0.9166901
Slc35a4	0.0534436	5.9112626	0.652256	0.9169723
Tox	0.1818244	0.5062229	0.6522683	0.9169723
Washc3	0.0921362	4.0772003	0.6524796	0.91712
Kif15	-0.0590597	7.0752725	0.652584	0.91712
Tmem202	-0.1982747	-0.141656	0.6527736	0.91712
Ppp1r26	-0.2035334	0.692283	0.6527823	0.91712
Hal	-0.1587526	1.8296459	0.6527884	0.91712
Mgat2	-0.053101	6.2104852	0.6528918	0.91712
Gm14270	-0.2733632	-0.7938499	0.6529369	0.91712
Btnl6	-0.2382598	0.4071997	0.6529626	0.91712
Gatad2a	0.0522192	6.6704443	0.6529864	0.91712
Slx4ip	0.0821053	3.7046946	0.6530281	0.91712
Tmem98	0.1535674	2.2467145	0.6530309	0.91712
Ska2	-0.0622313	5.4524169	0.653081	0.91712
Kdm1a	0.0616996	5.8083162	0.6532634	0.9172934
Gabarapl2	0.0805005	5.7825007	0.6535628	0.9176292
Uxs1	0.0660575	4.5750397	0.6536205	0.9176292
Tmem238	0.1110653	3.0584104	0.6537857	0.9176485
Pcdhgb2	-0.2204212	0.9018433	0.6538035	0.9176485
2810030D12Rik	0.2198039	0.7665917	0.6538312	0.9176485
Ncl	0.0570154	9.7031469	0.6539775	0.9176485
Dgkq	0.0928644	3.366992	0.6540101	0.9176485
Ppp2r5c	-0.0562653	7.458445	0.6540107	0.9176485
Ufsp2	-0.0683061	5.1185668	0.6540472	0.9176485
A630001G21Rik	0.0811895	4.3632095	0.6541231	0.9176581
Apoo	0.090246	3.7344261	0.6542049	0.9176581
Vps52	-0.0596548	5.7378179	0.6542895	0.9176581
Ska1	0.0848904	4.9472277	0.6542901	0.9176581
Gm29948	0.2726765	-0.6890898	0.6543528	0.9176633

Ociad1	0.0475669	7.0356288	0.654458	0.917675
Dmtf1	-0.05205	5.6166575	0.6544811	0.917675
Cript	0.0599265	5.4589726	0.6545612	0.917675
Polr3a	0.084264	4.0868463	0.6545972	0.917675
Tmem51	0.0942268	2.8900701	0.6546646	0.9176869
Fxr1	-0.0534552	6.9616063	0.654876	0.9179005
Fadd	0.0801272	4.0186581	0.6549998	0.9179007
Slc37a3	-0.0808829	4.2702326	0.6550075	0.9179007
Mrpl33	-0.0891222	6.0733686	0.6550533	0.9179007
Etaa1	-0.0763071	4.8407601	0.6551924	0.918013
Gm33273	0.2602655	-0.7279998	0.6555217	0.9181569
Gm7964	0.0602474	5.4467905	0.6555649	0.9181569
Foxd2os	0.1845676	1.5313932	0.6555952	0.9181569
Zfp862-ps	0.0948771	3.7089752	0.6558624	0.9181569
Plp1	-0.130683	1.6049899	0.6559135	0.9181569
Bmp7	0.2037404	1.7754113	0.6559516	0.9181569
Hirip3	0.061904	5.8128731	0.6560144	0.9181569
Stard5	-0.0813487	3.8468538	0.656029	0.9181569
Yipf1	-0.0745061	4.6435208	0.656099	0.9181569
Slc4a7	0.0664739	5.0482251	0.656185	0.9181569
Cyb5d1	0.0572547	4.8388392	0.6562095	0.9181569
Timm8b	-0.0812087	5.238624	0.6562625	0.9181569
Usp54	-0.0810005	3.6423764	0.656306	0.9181569
Abi3bp	-0.0989066	4.2756647	0.656345	0.9181569
Shld2	0.1078807	3.0656068	0.656361	0.9181569
Gm9347	0.5835795	-1.0595556	0.6564292	0.9181569
Map9	-0.2525582	0.2758761	0.6564293	0.9181569
Thtpa	0.0771886	3.4509972	0.6564362	0.9181569
Arsa	-0.0837116	3.8282214	0.6564747	0.9181569
Vps28	-0.0516095	5.6414818	0.6564757	0.9181569
Insyn1	0.266059	0.2033894	0.6566082	0.9182597
Tnnt2	0.2119789	-0.2344987	0.6567107	0.9183204
Lrrc8a	0.0612818	4.4707656	0.6568162	0.9183854
Gm10029	-0.1728859	1.416641	0.6569524	0.9184932
Mob3b	0.0893778	2.9747363	0.6571825	0.9187324
Itk	-0.1494728	1.9455988	0.6572783	0.9187838
E130112N10Rik	-0.2654917	-0.4885606	0.6573527	0.9188051
Ikzf5	0.058293	5.7683129	0.6577136	0.919227
Cdc42ep5	-0.1051917	2.1796392	0.6581398	0.91974
Lrrc40	0.0706334	4.9065535	0.6583906	0.9200079
Polh	0.0601821	5.6929247	0.6584748	0.9200429
Exoc3	-0.0491117	5.4860546	0.6585601	0.9200562
Gm40582	0.345715	-0.355043	0.6586026	0.9200562

Lzts1	0.2074477	0.0482391	0.6587187	0.9201357
Syt14	0.203181	3.0160109	0.6588916	0.9202946
Spa17	0.1992047	0.9098753	0.6591249	0.9205377
Dpp8	-0.0555786	7.476339	0.6593112	0.9206529
Mbtps2	-0.0686295	4.2725088	0.6593697	0.9206529
Atp9b	-0.0608374	5.0947413	0.6595112	0.9206529
4930550C14Rik	-0.2059134	0.4694209	0.6595765	0.9206529
Plk4	-0.0569107	6.4356662	0.659624	0.9206529
Dsp	-0.2431211	0.176686	0.6596324	0.9206529
2510009E07Rik	-0.0782217	4.3950129	0.6596506	0.9206529
Zfyve28	-0.184421	0.8115622	0.6597044	0.9206529
Mtmr3	-0.057029	7.5958248	0.6597704	0.9206529
Rph3a	-0.2706996	-0.8745294	0.6597993	0.9206529
Gm30934	0.2720555	-0.394208	0.6599241	0.9206562
Pdzd9	0.1684442	0.9366933	0.6599722	0.9206562
Dbi	-0.0710823	6.1299322	0.6601722	0.9206562
Scarb2	-0.0525511	6.4169658	0.6602113	0.9206562
Tsr2	0.0767171	3.804282	0.6602453	0.9206562
Stambp	0.0686286	4.0726406	0.6602608	0.9206562
Slc9a2	-0.1541292	2.4138906	0.6602676	0.9206562
Ywhah	0.0538602	8.3840923	0.6602752	0.9206562
Srp54a	0.3922637	1.1107852	0.6604546	0.9207982
Klc1	0.0570125	5.7135985	0.6605484	0.9207982
Myo7a	0.0868597	4.8572041	0.6605546	0.9207982
Cnot10	0.0575511	5.3148529	0.6608284	0.920877
Gm13446	0.2251284	-0.172788	0.6608331	0.920877
Tpp1	-0.0482473	6.6052262	0.6608636	0.920877
Cyp4x1	-0.2394769	-0.6353937	0.6608703	0.920877
Foxo4	0.0575114	5.9802909	0.6609072	0.920877
Efnb3	0.1506414	0.8591527	0.6609794	0.9208951
Kars	0.0567877	6.5440497	0.6610952	0.9209741
Slc35e2	0.0626498	4.926336	0.6611952	0.9210308
Zfp85	0.1016668	2.5096384	0.6614505	0.9213039
Kif2a	-0.058493	6.8751141	0.6616515	0.9215014
Scd2	0.089226	6.8571967	0.6617145	0.9215066
Dcakd	-0.0721541	3.7139369	0.6618109	0.9215584
Siglece	0.1262669	5.3040897	0.6618808	0.9215731
Nbas	-0.0776775	4.5583754	0.6621061	0.9218044
Grik5	-0.0867745	2.9280789	0.6621672	0.9218069
Rcan2	0.1215701	3.4001415	0.6622381	0.9218231
H2ac8	0.2232339	0.1459364	0.6624105	0.9219806
H2-Q9	-0.2517503	0.2080735	0.6625226	0.9219999
Sec23b	-0.0459784	5.5957523	0.662543	0.9219999

Atp5pb	0.0652781	8.2692748	0.6627679	0.9220982
Tppp3	0.1608073	2.3518716	0.662828	0.9220982
Slc22a23	0.087443	5.6964869	0.6628356	0.9220982
Cln6	0.0675825	4.7809757	0.6628507	0.9220982
Arhgap39	-0.0700053	3.8974106	0.6629431	0.9221288
Tnn	0.346221	2.1624861	0.6630389	0.9221288
Tnrc6c	-0.055249	5.9731985	0.6630506	0.9221288
Shq1	0.1150447	2.8238495	0.6631463	0.9221451
Dzip3	-0.0614955	4.8995952	0.663209	0.9221451
Pex2	-0.0604775	4.6594426	0.6632401	0.9221451
Gm15232	0.176043	0.5347912	0.6633817	0.9222252
Ect2	-0.072525	6.5768415	0.6634163	0.9222252
Inpp5e	0.0613131	4.2174264	0.6635561	0.922337
Aacs	-0.1026421	4.3286244	0.6638358	0.9226434
Arfgap1	0.0544821	5.2948402	0.6640449	0.9228515
Kras	-0.0495175	6.7654474	0.6643773	0.9229767
Etfrf1	-0.084255	4.2678406	0.6643972	0.9229767
Ccn4	-0.1413764	6.4842779	0.6644562	0.9229767
Sf3b2	0.0554876	8.0958637	0.6644923	0.9229767
Capn10	0.077961	3.4751488	0.6645053	0.9229767
Tnfsf10	-0.0894094	3.2284527	0.6645611	0.9229767
Fbxl17	0.0647837	4.9989671	0.6646238	0.9229767
Sh3kbp1	-0.0604823	6.938409	0.6646586	0.9229767
Gt(ROSA)26Sor	0.1179997	3.9513353	0.6647198	0.9229767
Rgs1	0.219318	1.5825707	0.6647284	0.9229767
Maea	0.0467592	6.2456523	0.6648315	0.9230375
Tnfaip2	0.0678801	7.4301459	0.6650679	0.9232438
Mrpl1	0.0817906	4.2256272	0.6651301	0.9232438
Dapk1	-0.0812387	4.8854689	0.6651582	0.9232438
Slc50a1	0.07176	4.0864493	0.6652468	0.9232757
Eif2s3x	-0.0535529	6.6876871	0.6653301	0.9232757
Khk	-0.0712346	3.946001	0.6653592	0.9232757
Rnf214	-0.0570588	5.5954849	0.6654847	0.9233675
Cul1	0.0493735	7.0352259	0.6655887	0.9233753
Slc33a1	0.0653062	4.7788792	0.6656091	0.9233753
Gm17828	0.1963177	0.3501743	0.665928	0.9237354
Htatip2	0.0849137	4.6553617	0.6662069	0.9240397
Apba1	-0.1039542	3.6639148	0.6663835	0.924088
Creb3l1	0.1413207	5.1809701	0.6663936	0.924088
Rad9a	0.0670844	4.6017015	0.6665314	0.924088
Tlcd3a	0.1208721	1.4229693	0.6665614	0.924088
Gm5141	0.124383	2.295514	0.666614	0.924088
Stard3nl	0.0615086	4.0652714	0.6666148	0.924088

Prim2	0.0621812	5.9188231	0.6666575	0.924088
Noxred1	-0.1854281	0.50929	0.6667565	0.9241393
Ciao3	-0.0559169	4.181483	0.6668525	0.9241393
Gm29679	0.1848547	-0.7175458	0.6668728	0.9241393
Trmt5	-0.0770132	3.5430325	0.6669536	0.9241689
Mtss2	0.0865129	5.0759047	0.6671934	0.9242224
Ero1b	-0.0720171	4.7704879	0.6672869	0.9242224
LOC115488789	-0.1435682	0.3879733	0.6673	0.9242224
Peli3	0.2285773	-0.3830702	0.6673135	0.9242224
Pou6f1	-0.1034174	3.2898676	0.6674823	0.9242224
BC051226	0.1532434	1.0986557	0.6675808	0.9242224
Pik3r4	0.059768	4.5619069	0.6676196	0.9242224
Mrps22	-0.0836594	4.2856927	0.6676721	0.9242224
Oip5	0.0752172	4.6215703	0.6676727	0.9242224
Nipbl	-0.0632363	7.825858	0.6677571	0.9242224
Gm4013	0.1504868	0.868983	0.6679171	0.9242224
Ribc1	0.1923402	0.8350371	0.6679436	0.9242224
Mpp6	0.052594	5.5905019	0.6679594	0.9242224
Pfdn2	0.0603157	5.4344936	0.6679974	0.9242224
Uck2	0.0565838	6.3966502	0.6681176	0.9242224
Fam89b	0.0506084	5.1695118	0.668143	0.9242224
Dot1l	0.0677611	6.695644	0.6681634	0.9242224
Gm12663	0.0869306	3.6308573	0.6682298	0.9242224
Rmi2	0.0733147	3.71884	0.6682514	0.9242224
Cpeb3	0.0913942	3.9590756	0.6682799	0.9242224
Kdm2a	-0.0534444	7.3119667	0.6683217	0.9242224
Wls	-0.0523246	6.0807738	0.6683392	0.9242224
Spc25	0.0739453	6.4651653	0.6683777	0.9242224
Desi2	-0.0591219	4.8009589	0.6684183	0.9242224
Ppp1r42	-0.1472261	2.0805115	0.6686757	0.9244962
Nsmaf	0.0507145	5.6425569	0.6688009	0.9245774
Srrm2	-0.0591255	8.9346611	0.6688533	0.9245774
Gm34484	0.3048201	-0.0001961	0.6689835	0.9246751
Cep43	0.0687131	4.9685709	0.6691815	0.9248666
Vps39	0.056906	5.0249306	0.6693781	0.9249736
Dpysl2	0.1286948	4.2201033	0.6694644	0.9249736
Gm15246	-0.1287926	1.7184234	0.6695208	0.9249736
Gm12435	-0.2811091	-0.3454376	0.6695412	0.9249736
Copg1	0.0488606	6.7000328	0.6696606	0.9249736
Gpc1	-0.0764651	6.7522091	0.6697384	0.9249736
Pcdhgb6	-0.1797919	0.5433019	0.6697832	0.9249736
Asf1a	0.0577967	5.8524981	0.6698272	0.9249736
Cd14	-0.1795797	3.3245186	0.6699163	0.9249736

Zc3hav1l	0.0841383	3.5697354	0.6701345	0.9249736
Tmem69	0.0832569	4.3643067	0.6702178	0.9249736
Gm30369	-0.2295506	-0.8013928	0.6702347	0.9249736
1700052K11Rik	0.1054994	2.8271307	0.6702502	0.9249736
Nt5c3b	0.0643297	3.8983551	0.6702996	0.9249736
Cep104	0.057016	4.4310016	0.6704167	0.9249736
Nfib	-0.1090506	4.4213061	0.6704575	0.9249736
Rufy1	-0.0654798	4.852117	0.6705413	0.9249736
Nrbp2	0.1283616	3.2186655	0.6705561	0.9249736
Pdss1	0.0967587	3.1985472	0.6706075	0.9249736
Zfp420	-0.1286897	1.8962247	0.6706201	0.9249736
Cacna1d	0.1179934	1.4757656	0.6706338	0.9249736
AI450353	0.1345978	1.6995732	0.6706379	0.9249736
Zfand2b	-0.0634458	4.5890933	0.670666	0.9249736
Rnf216	-0.0570164	5.982349	0.6706861	0.9249736
Clmp	0.0825144	4.6113391	0.6708152	0.924994
Clpx	0.0719107	5.5105923	0.6708199	0.924994
Bag6	0.0502941	6.835301	0.6708969	0.9250029
Ilvbl	0.0805724	3.7036074	0.6709453	0.9250029
Pcdh18	-0.1262312	3.7556435	0.6711419	0.9251396
Zfp790	-0.0730828	4.090586	0.6711634	0.9251396
Rprd1a	-0.0762518	3.8239202	0.6713014	0.9252479
B2m	-0.0560411	10.113777	0.6714707	0.9253991
Gtf2a1	0.070463	6.0463185	0.6715361	0.9254073
Cd247	-0.1410266	1.1525805	0.6716481	0.9254581
Car3	0.1065409	7.6298799	0.6716919	0.9254581
Zfp865	0.0602519	4.4066999	0.6717809	0.9254711
Arid1a	-0.071251	7.8476845	0.6718204	0.9254711
Ints14	0.0532334	5.2146411	0.6719423	0.9254863
Irf7	-0.1087009	4.4429716	0.6719504	0.9254863
Pcsk5	-0.1307771	3.7355113	0.6720841	0.9255247
Noc2l	0.0504796	6.5384998	0.6721522	0.9255247
Med20	-0.0564258	5.255743	0.6721568	0.9255247
Rnf139	0.068977	6.6278705	0.6724488	0.9256903
Nmd3	0.0721848	4.7556489	0.6724631	0.9256903
Nup155	0.0539344	6.1703272	0.6725048	0.9256903
Lin52	0.0839558	3.7794775	0.6725744	0.9256903
Gm3604	0.1398583	2.3504561	0.6725867	0.9256903
Tmem17	0.2292832	0.2289572	0.6726906	0.9256903
Maoa	-0.1027439	2.8320095	0.6727852	0.9256903
Rpn1	0.0459477	7.6777453	0.6728296	0.9256903
Zfp747	0.1262189	2.1615237	0.6728313	0.9256903
Plod3	-0.0570079	5.7159404	0.6728722	0.9256903

Mrap	0.1397978	1.5857823	0.672976	0.9257512
Rcn3	0.1374638	6.4913219	0.673059	0.9257834
Slc26a11	-0.1077899	2.3602327	0.6732588	0.9259734
Gm18588	0.2331042	0.321569	0.6733534	0.9259734
Map3k20	0.0583115	5.5749218	0.6734187	0.9259734
Gm10123	-0.0614536	4.5143	0.6734546	0.9259734
Fbxo3	0.0573106	7.1034612	0.6734947	0.9259734
Kpna2	0.053441	7.8874371	0.6735787	0.9259758
Rogdi	-0.0755493	5.0143626	0.6736155	0.9259758
Sting1	0.0692491	4.5770431	0.6737195	0.9260369
LOC118567753	0.2196203	0.0978417	0.6738407	0.9261217
Tmem189	-0.0541563	5.2160271	0.6741334	0.9262165
Kcnq5	0.1566827	1.7346233	0.6741868	0.9262165
Csnk2b	-0.0599512	5.1314103	0.6742092	0.9262165
Rpl18-ps1	0.2818381	-0.5886159	0.6742605	0.9262165
Morf4l2	0.0521566	7.3433646	0.6742734	0.9262165
Dido1	-0.0622485	6.9867917	0.6744564	0.9262165
1810034E14Rik	-0.1368729	0.6711023	0.6745385	0.9262165
Adipor1	0.0588889	8.4017894	0.674566	0.9262165
Fads2	0.0573541	5.3384743	0.6746765	0.9262165
Btnl2	-0.280436	0.656957	0.6746801	0.9262165
Macrod1	0.0983953	2.4476685	0.6746983	0.9262165
Hcfc2	-0.0513435	4.9967357	0.6748137	0.9262165
Ndufa3	0.0610606	6.60778	0.6748175	0.9262165
Ccdc9b	0.164842	1.6332379	0.6748222	0.9262165
Surf1	-0.0576815	4.4687987	0.6748311	0.9262165
Zfp383	0.1167196	1.9913053	0.674886	0.9262165
Mfsd12	0.0614427	5.3814327	0.6749814	0.9262165
Lama2	-0.1592517	1.4592474	0.6749998	0.9262165
0610040B10Rik	0.3256205	-0.8425513	0.6750822	0.9262165
Rere	0.0809202	6.9129586	0.6751007	0.9262165
Klra5	-0.1358449	0.6765175	0.6753238	0.9264408
Baz1b	0.059481	8.1486146	0.6754597	0.9265396
Lsm10	-0.0774789	3.2276794	0.6755468	0.9265396
Gm10240	0.4104307	-0.6958587	0.6755745	0.9265396
Csdc2	0.2967688	0.4756666	0.6757113	0.9266455
Npm3	0.0665689	5.5023795	0.6758002	0.9266858
Atf2	-0.0454138	6.1801537	0.6759182	0.9267659
Gm31253	-0.1988052	-0.1008648	0.6762548	0.9268471
Casp3	-0.0559778	6.8448021	0.6762808	0.9268471
Hid1	-0.1288194	2.4046672	0.6762994	0.9268471
Preb	-0.0511734	6.2199984	0.6763152	0.9268471
Jrk	-0.1449346	1.1102811	0.6763205	0.9268471

Mavs	0.0734498	5.3734298	0.676335	0.9268471
Thra	0.0478273	5.5785292	0.6765647	0.9270387
LOC118568495	0.208906	0.2661561	0.676594	0.9270387
Trim46	0.205664	0.033652	0.6767076	0.9271126
Ftl1	0.0567326	10.950811	0.6769944	0.9273545
Gm3625	0.2566487	-0.2225401	0.677109	0.9273545
Gm26588	-0.1840313	0.0620679	0.6771579	0.9273545
Cep44	0.0780748	3.9123337	0.6772138	0.9273545
Ankrd13c	0.0499927	6.1030674	0.6772269	0.9273545
Tmlhe	-0.1022406	2.157835	0.6773967	0.9273545
Gm6501	-0.1618289	0.8029417	0.6774321	0.9273545
Stat2	0.0532071	5.2508081	0.6774597	0.9273545
Ilrun	0.0574969	7.4209227	0.6774636	0.9273545
Mettl18	0.1076849	2.2531219	0.6776085	0.9273545
Wac	-0.0528893	7.1186985	0.6776609	0.9273545
Ube2t	0.0857668	3.4126971	0.6776806	0.9273545
Pgs1	0.0712834	5.1643902	0.6776978	0.9273545
Dhx34	0.0757531	3.9422179	0.6777188	0.9273545
Nxpe1-ps	0.1408787	1.4589615	0.6778456	0.9274464
Rnf225	0.3459547	-0.619558	0.678007	0.9275856
9030607J07Rik	0.259794	-0.6046959	0.6781254	0.927666
Sike1	-0.0642676	4.6528569	0.6781968	0.9276752
Mettl14	0.0571089	4.7976999	0.678285	0.9276752
Id1	-0.0731273	4.0242885	0.6783218	0.9276752
Nprl2	0.0854676	2.9963866	0.6783952	0.9276752
Plat	-0.284241	0.8699002	0.6784974	0.9276752
Hmg20a	0.0516962	4.9716926	0.6785799	0.9276752
Adprm	-0.0823769	3.8096788	0.6785954	0.9276752
Gm5069	0.2787371	0.067183	0.678718	0.9276752
Ncdn	0.0684831	4.24532	0.6788018	0.9276752
Gm10350	-0.4019344	-1.265597	0.6788213	0.9276752
Purb	-0.0488558	7.9185649	0.678868	0.9276752
Gm9282	0.3962674	-1.1172065	0.6788704	0.9276752
LOC118568796	0.1308945	2.1708363	0.6789397	0.9276752
Ap4e1	0.0551033	4.725239	0.6789767	0.9276752
Syngr1	0.0852678	4.1014034	0.6790302	0.9276752
Gm39608	-0.1962235	1.5255491	0.6791842	0.9276752
Katnbl1	0.0616355	4.7440403	0.6791871	0.9276752
AV099323	0.1211968	2.0545235	0.6793748	0.9276752
Nfkb2	0.084647	5.171894	0.6794059	0.9276752
C330007P06Rik	0.0446057	5.7736647	0.6794598	0.9276752
LOC118567770	0.3401766	-0.6249379	0.679492	0.9276752
Gemin5	0.0622704	4.7496816	0.6795201	0.9276752

Rnf181	0.0470905	5.7539551	0.6795603	0.9276752
Gm40180	0.1402891	2.8999672	0.6796159	0.9276752
Clcc1	-0.0513903	4.9991682	0.6796321	0.9276752
Fam181b	-0.2802414	-0.5163847	0.6796828	0.9276752
Gm21811	0.1262073	1.2157351	0.6797832	0.9277308
Oxsm	-0.1014169	3.7854145	0.6799764	0.927844
Gm33938	-0.2035465	-0.1059355	0.6801226	0.927844
9630028I04Rik	-0.2210747	-0.4329877	0.6801687	0.927844
Nucks1	0.0540267	8.7838277	0.680323	0.927844
Acvr2b	-0.155594	1.7764644	0.6803477	0.927844
Slc8a3	-0.1440949	4.3134141	0.6803891	0.927844
Zfp335	-0.0555092	5.1077689	0.6804485	0.927844
Gm30352	-0.1373851	0.7145643	0.6804544	0.927844
Mtor	0.0642754	5.3226793	0.6804842	0.927844
Abcf3	0.0514531	5.0531304	0.6805368	0.927844
Kpna6	0.0579439	6.0315641	0.6805748	0.927844
Cmc4	0.1055251	2.7359576	0.6806415	0.927844
Caprin1	-0.0518774	8.635227	0.6806417	0.927844
Rnaset2b	-0.3503213	3.1774048	0.6807175	0.9278661
Gm12184	-0.1968534	-0.2119994	0.6810075	0.9280748
Rims1	0.2846224	-1.0350011	0.6810239	0.9280748
Kat5	0.0488572	5.2284831	0.6810496	0.9280748
AI225912	-0.1408277	2.3073923	0.6813607	0.9281823
Ppm1b	0.0472581	6.6346529	0.6813678	0.9281823
Zfp658	0.1307323	1.7566209	0.6813964	0.9281823
Ldha	0.0507297	8.46634	0.6814404	0.9281823
Ano6	-0.0515691	6.691708	0.6814712	0.9281823
Zfp426	-0.0539938	4.6483644	0.6814865	0.9281823
Chaf1b	0.0619779	5.3512169	0.681693	0.9282164
A130051J06Rik	0.2110089	-0.2762504	0.6817048	0.9282164
Pcgf2	0.1456675	1.9838162	0.681832	0.9282164
Trim35	-0.0519658	5.5534571	0.6818426	0.9282164
Hnrnpa1	0.0502825	9.28242	0.6818884	0.9282164
Rbbp6	0.0496163	7.1529291	0.6819661	0.9282164
Dcaf10	0.0526038	5.2191426	0.6821414	0.9282164
Gmps	0.0492625	6.3999362	0.682217	0.9282164
Stac2	0.1619659	1.5063559	0.6823042	0.9282164
Ccz1	0.0484409	5.8576996	0.6823494	0.9282164
Mon2	-0.0504996	6.1319436	0.6824825	0.9282164
Sgk1	0.0594396	5.1149689	0.6825242	0.9282164
2410022M11Rik	0.1855931	0.5066248	0.6826232	0.9282164
Parp2	0.0552377	5.5356739	0.6826397	0.9282164
Gm6297	0.2784839	-0.7643031	0.6827052	0.9282164

Ptpro	-0.0877476	3.8010608	0.6827478	0.9282164
Ccdc18	0.076782	4.4292941	0.6827619	0.9282164
Lipt1	-0.1310127	0.8461911	0.6827888	0.9282164
Gm8494	0.228323	-0.1783868	0.6829719	0.9282164
Gm12359	0.2529128	-0.650467	0.6829842	0.9282164
Gm9222	0.0909832	3.028669	0.6830301	0.9282164
Pbx2	-0.0518565	6.2961126	0.6831042	0.9282164
Fbxl18	0.0734405	3.5968325	0.6831449	0.9282164
Acbd6	0.0521227	4.8339414	0.6831704	0.9282164
Edem3	0.0641361	6.4330408	0.6832236	0.9282164
Dvl2	0.0684661	3.7767897	0.6832277	0.9282164
Pcca	-0.0817029	4.0839241	0.6832308	0.9282164
Atad2	-0.0587817	7.8208308	0.6832757	0.9282164
Tsg101-ps	-0.2427801	-0.9074421	0.6834864	0.9282164
Ccdc17	0.1409593	0.5248784	0.6835021	0.9282164
Srpx2	0.1227341	4.1181242	0.6835038	0.9282164
Spred1	0.0572007	5.7185057	0.6835363	0.9282164
Pex11g	-0.1247935	1.4340799	0.6835372	0.9282164
Bckdhb	0.1220607	3.1604904	0.6835696	0.9282164
Spon2	-0.139762	2.0063506	0.6836836	0.9282164
Rpl37	0.0592329	8.9445963	0.6837014	0.9282164
Hsd3b7	-0.0727791	4.3750204	0.6837255	0.9282164
LOC118567814	0.2252961	-1.1052203	0.683872	0.9282164
Gale	-0.0762598	3.1791994	0.6839197	0.9282164
Hspa4	0.0464444	7.6603977	0.6839585	0.9282164
Pdk1	0.065567	4.9760901	0.6839637	0.9282164
Mapre3	-0.1368343	1.9986704	0.684018	0.9282164
Mdh2	-0.0489061	7.6191869	0.6840798	0.9282192
Haus8	0.0549529	5.1655904	0.6841735	0.9282284
Zfp367	0.0687084	6.5839489	0.6842226	0.9282284
Trim37	0.053496	5.9834415	0.6842809	0.9282284
Gm5841	0.3518871	0.3152874	0.6843734	0.9282284
Zdhhc24	-0.0696491	4.1737945	0.6844493	0.9282284
Tpm3-rs7	-0.4020128	5.9185925	0.6844889	0.9282284
Malt1	-0.0651865	5.5128597	0.6845085	0.9282284
Pawr	0.0837315	3.4053636	0.6846091	0.9282284
Fance	0.0859183	3.3507314	0.6846237	0.9282284
Ywhag	-0.0560602	7.2722758	0.6847229	0.928282
Gimap8	0.1155297	4.1732967	0.6848601	0.9283872
A330069E16Rik	0.2716412	-0.766359	0.6849843	0.9283963
Tmed7	-0.0481559	6.882946	0.6850804	0.9283963
Btbd11	-0.1346875	0.9317137	0.6851	0.9283963
Yif1b	-0.0514669	4.6283934	0.6851056	0.9283963

Gm3325	0.0560251	4.4034727	0.6852023	0.9284464
Eif3a	-0.0528801	8.9574772	0.6853745	0.9285711
Gm39181	0.1464365	0.9327806	0.6854137	0.9285711
Dab2	-0.0705008	4.9816289	0.6854854	0.9285873
3110053B16Rik	-0.1984612	2.2077773	0.685572	0.9286238
Wtip	0.1560375	2.1453649	0.6856907	0.9287037
Rerg	-0.1591073	3.4326183	0.6859746	0.9289734
Mcmbp	-0.0513138	6.2833397	0.6860345	0.9289734
Vgll4	-0.0590958	5.1614326	0.686069	0.9289734
Ofd1	0.061017	4.1803037	0.686138	0.928986
Tmem164	-0.0536827	6.689114	0.6863519	0.9291947
Rexo1	0.0555597	5.9881057	0.6864394	0.9292322
9330159M07Rik	0.3613822	0.7881318	0.6866735	0.9293622
Eif3j1	-0.0515716	5.7798734	0.6867641	0.9293622
Kif24	0.0771937	4.5567646	0.6867718	0.9293622
Gm39321	-0.1341935	1.8782867	0.6868625	0.9293622
Fbxw10	-0.2206958	-0.0674187	0.6869188	0.9293622
Ptgr2	-0.0464352	5.4785397	0.6869509	0.9293622
Pcna	0.0588187	8.9481636	0.6869537	0.9293622
Sfxn3	0.0762021	4.0443351	0.6870209	0.9293723
Crtc1	0.0749249	3.5782488	0.6871811	0.9295083
Cdc6	0.0645052	7.1430687	0.6875142	0.9298025
Elf2	0.0435618	6.6875158	0.6875619	0.9298025
Armc6	0.0800358	3.0305044	0.6876535	0.9298025
Gm5905	-0.1070915	2.1697587	0.6876912	0.9298025
Cab39l	-0.0510997	5.5878729	0.6877455	0.9298025
Cep57	0.0612026	5.8592648	0.6877955	0.9298025
Sgk3	-0.0536419	6.8189358	0.6878756	0.9298025
Eya4	-0.1301528	3.3187736	0.6878769	0.9298025
Gm20234	0.2554801	-0.584344	0.6881516	0.930019
Wnt11	0.2352511	-0.8115806	0.6881756	0.930019
Snapc2	0.0907821	3.3145802	0.6882165	0.930019
B3gnt7	-0.1718919	0.5364621	0.6883597	0.9300493
Junos	0.2660134	2.9455118	0.6883631	0.9300493
Zfp59	0.0996844	2.37984	0.6884358	0.9300493
Nova2	-0.1857646	1.0933749	0.6884781	0.9300493
Ago1	0.0674909	5.6021604	0.688557	0.9300751
Gm6900	-0.1682874	-0.3012765	0.68865	0.9301
Gm14253	-0.1426783	-0.1619913	0.688695	0.9301
Akr1b10	-0.0780189	3.5648188	0.6889115	0.9303117
Ptgs2	-0.2007227	-0.6423157	0.6892003	0.9306208
Gmcl1	0.0573121	4.9186149	0.6892899	0.930661
Cdk1	0.0706919	7.4013746	0.6894006	0.9306857

Septin10	-0.0844558	3.1636528	0.6894278	0.9306857
Cchcr1	0.0652511	4.3554124	0.6898295	0.9310839
Zfp939	-0.1518334	0.4099246	0.6898822	0.9310839
Cutc	-0.0723642	3.2282019	0.6899522	0.9310839
Gigyf1	0.0519641	5.8055962	0.6899623	0.9310839
Trappc9	-0.06565	4.7855736	0.6900848	0.9311159
Col8a1	-0.1266811	6.8080356	0.6901057	0.9311159
Acap3	0.0553489	4.8426141	0.6902605	0.9311317
Sec61b	0.0686393	6.5728543	0.6903936	0.9311317
Rpn2	0.0430086	7.6234413	0.6904439	0.9311317
Mia	-0.2170327	0.9258832	0.6904669	0.9311317
Smoc2	0.1838368	3.613599	0.6905749	0.9311317
Actr1b	-0.0519767	5.6461462	0.6905977	0.9311317
Dennd2c	0.0883225	4.4455861	0.6905989	0.9311317
Acat3	-0.242693	-0.4050209	0.6906401	0.9311317
Gfod2	0.1462775	1.3647302	0.6906708	0.9311317
Kxd1	0.0532179	4.8299804	0.6907789	0.9311317
Cxxc1	0.0524723	6.1022442	0.6908182	0.9311317
Klhl36	0.0715015	3.3417375	0.6908358	0.9311317
Mid1	-0.2021629	1.0792	0.691143	0.9314651
Daam2	0.0904923	4.1520738	0.6913109	0.9316106
Ssx2ip	0.0739749	6.2000722	0.6914846	0.9316876
Eya1	-0.1395928	1.6338731	0.6914878	0.9316876
Malat1	-0.0720272	9.2591717	0.6916426	0.9317797
Sidt1	-0.1981139	0.3049344	0.6916787	0.9317797
Nol11	-0.0529449	5.02208	0.6917359	0.9317797
Ylpm1	-0.0625894	6.2579207	0.6917999	0.9317838
Efnb1	-0.0893126	4.4531046	0.6919125	0.9317838
Pcdhb5	-0.1989988	-0.2522418	0.6919187	0.9317838
Gm6136	0.0653414	3.7571203	0.6919954	0.9318064
Fhod3	0.1571781	3.3971363	0.6921419	0.9319231
Ube2l3	0.0451851	7.3813222	0.6922161	0.9319422
Raly	0.0479329	7.9222922	0.6924774	0.9322134
Prrc2c	0.0520979	7.7619599	0.6927146	0.9324521
Cc2d1b	0.0594071	4.957738	0.6927971	0.9324824
Msln	-0.2271179	-0.5397522	0.6930154	0.9326761
Mrpl40	0.0587411	4.8892509	0.6930609	0.9326761
Atg16l2	-0.0898176	4.238074	0.693235	0.9327234
Snx32	-0.0985467	2.8485106	0.6932487	0.9327234
Man2c1os	-0.1960753	-0.3810854	0.693276	0.9327234
Tmem101	0.0800805	3.5414675	0.6934802	0.9328493
Tbc1d1	-0.0556557	5.3910625	0.693502	0.9328493
Insc	-0.1629076	3.1451506	0.6936157	0.9328493

Stt3a	-0.0438712	7.565554	0.6936367	0.9328493
Fam110c	0.1179582	1.685842	0.6936695	0.9328493
Phf23	-0.0445643	5.6343115	0.6938265	0.9329798
Actn4	-0.0534327	8.1716376	0.6939577	0.9330755
Bbs1	0.1076409	1.5826477	0.6941462	0.9332331
Hars2	-0.0576431	4.2219639	0.694222	0.9332331
Nmnat2	0.2172325	0.1583447	0.694283	0.9332331
Cyb5b	-0.0481075	6.0708737	0.6943149	0.9332331
Ptch2	-0.169013	1.1618771	0.6944569	0.9332509
Mirt1	-0.1593282	2.6742701	0.6944978	0.9332509
Uspl1	0.0549845	5.1218139	0.6945758	0.9332509
Synj2bp	-0.0461034	5.6515218	0.6945823	0.9332509
Cbx3-ps7	0.213949	-0.2175649	0.6946281	0.9332509
Oga	0.0451887	7.2717126	0.6948312	0.9334432
Gm41098	0.1957645	-0.1942778	0.6949099	0.9334682
Plxnb1	-0.1237595	3.0387352	0.6949754	0.9334756
Sptssa	-0.059681	6.2414465	0.6951915	0.9336853
Eif1ax	0.0465739	6.8592947	0.6952561	0.9336913
Colca2	0.2088513	-0.997309	0.6953843	0.9336922
Pold3	0.0542497	6.4402598	0.6954224	0.9336922
Aga	-0.0779026	3.7043816	0.6954452	0.9336922
Mad2l1	0.0592032	7.0629099	0.6954968	0.9336922
Irx5	-0.1476862	3.1376607	0.695687	0.9338669
Akt2-ps	0.1429969	0.0439992	0.6958001	0.9339381
Tprkb	0.0574338	4.4214009	0.6959675	0.9340822
Lmnb2	0.0645996	5.3462482	0.6962492	0.9341556
Mgat1	0.047988	6.3069403	0.6962893	0.9341556
Ttc9c	-0.0403134	6.1426825	0.6963031	0.9341556
Gins1	0.0584849	4.9179129	0.6964314	0.9341556
Bcl2a1d	-0.1884855	0.6807763	0.6964673	0.9341556
Mgme1	0.0629083	3.88608	0.6965639	0.9341556
Spo11	0.2708986	-0.9300867	0.696574	0.9341556
Rufy3	0.0648287	4.1772592	0.6966208	0.9341556
A130010J15Rik	-0.0889706	2.925924	0.6966364	0.9341556
Efemp1	-0.1677511	2.7850255	0.6966415	0.9341556
Gm20517	-0.2139599	-1.0092152	0.6967726	0.9341556
Erbb2	-0.1310935	1.8986226	0.696946	0.9341556
Scn2b	0.2234968	0.897477	0.6969593	0.9341556
Calm3	-0.0466297	8.4608408	0.6969604	0.9341556
Esam	-0.0565386	4.0959257	0.6970082	0.9341556
A930005H10Rik	0.1283305	2.6461814	0.6970339	0.9341556
Ndufa13	0.0543978	6.6769416	0.6970432	0.9341556
Tma16	-0.0813012	3.4712589	0.6971738	0.9341586

Mast4	-0.086784	4.6375181	0.6972503	0.9341586
Lmtk2	-0.059038	6.1087787	0.6972685	0.9341586
Rbmx	0.0497353	5.9768012	0.6973191	0.9341586
Snapc5	0.1258316	4.7767191	0.6973917	0.9341586
Fam219a	-0.0904143	3.1783184	0.6974631	0.9341586
Ephb3	-0.2083194	0.2989298	0.6975841	0.9341586
Nudt12	-0.109473	2.4161133	0.6976454	0.9341586
Cep170b	0.1265294	2.4761773	0.6976784	0.9341586
Atg9b	0.1800291	-0.1749682	0.6978363	0.9341586
Eif2ak4	0.0705584	4.2735444	0.6978381	0.9341586
Eme2	0.0924872	2.4292593	0.6978546	0.9341586
Ak6	0.0633808	4.6139834	0.6979363	0.9341586
Gm5619	-0.2167485	-0.0295598	0.6980724	0.9341586
Rnf38	-0.0490564	7.1557481	0.6980792	0.9341586
Ndufb5	0.0472823	5.6414285	0.6983082	0.9341586
Rusc1	0.0831988	3.1537121	0.6983227	0.9341586
Swt1	0.0623367	4.9840984	0.6985413	0.9341586
Dcxr	0.100129	2.6531759	0.6985415	0.9341586
LOC118568713	-0.1908443	0.5936587	0.6987268	0.9341586
Ppp1r12c	0.0418937	6.1193585	0.6987329	0.9341586
Eef1akmt1	0.0813056	3.1811977	0.6988076	0.9341586
Mllt10	0.0477331	6.3875183	0.6988685	0.9341586
Tomm70a	-0.045315	6.8803682	0.6990505	0.9341586
Klra1	-0.1781187	1.8481658	0.699059	0.9341586
Sec11a	0.0433552	5.866133	0.6990592	0.9341586
Wdr62	0.068838	4.2492167	0.6991117	0.9341586
Rhob	-0.0656423	6.1748907	0.6992146	0.9341586
Denr	0.0479656	5.6362706	0.6992256	0.9341586
Adamts9	0.0883535	4.5720226	0.699276	0.9341586
Cep41	0.0867506	3.3741329	0.6993107	0.9341586
Fam171a2	0.1478556	1.3573963	0.699383	0.9341586
Pkib	0.1130154	2.5223706	0.6993885	0.9341586
Gm10145	-0.1321433	0.2068913	0.6994028	0.9341586
Gm46626	0.2965985	-0.4981085	0.6994744	0.9341586
Mppe1	-0.0552724	6.2258024	0.6994781	0.9341586
Nol12	0.0628717	4.4039317	0.6994968	0.9341586
Fam126a	0.0592747	6.8443379	0.6995417	0.9341586
Sirt2	0.0448701	5.5204144	0.6996763	0.9341586
Rgs5	-0.1087294	5.8548927	0.699678	0.9341586
Larp1	-0.0489071	7.1538343	0.6996942	0.9341586
Abhd12	0.0523542	5.4704921	0.6997291	0.9341586
Muc3a	-0.1553504	1.5375106	0.6997319	0.9341586
Eral1	0.073599	3.6238468	0.6997439	0.9341586

Commd5	0.0773762	3.8847961	0.6998591	0.9341586
Gnpda1	-0.1176671	5.2003928	0.6998868	0.9341586
Nid1	0.07064	5.6336152	0.6999879	0.9341586
Arl2bp	-0.0405254	6.2350054	0.6999963	0.9341586
Caap1	-0.0641561	4.2371795	0.7001478	0.9341586
Inha	0.3064909	-0.4890619	0.7002048	0.9341586
Gphn	-0.0693329	3.7855829	0.7002629	0.9341586
Lrr1	0.0925337	3.8119037	0.700284	0.9341586
Atg9a	-0.0452014	5.9280282	0.7003645	0.9341586
Itm2a	-0.1270701	3.1855989	0.7004709	0.9341586
Gstm1	-0.0819913	6.8420659	0.7005419	0.9341586
Vma21	0.0476749	5.398558	0.7007553	0.9341586
Zfp759	0.1124703	2.1849	0.7007893	0.9341586
Spryd3	-0.0632529	4.3275772	0.7009793	0.9341586
Plxnb2	0.0538458	6.0628658	0.7009824	0.9341586
Irf2bpl	0.079769	3.7328382	0.7010105	0.9341586
LOC118568020	-0.1886327	0.1359373	0.7011681	0.9341586
Ly6a	-0.1466401	2.5163859	0.7011933	0.9341586
Entpd5	-0.0505125	5.5852559	0.701296	0.9341586
Oxr1	-0.0514663	6.3289284	0.701296	0.9341586
Gm14150	-0.1252639	2.0621092	0.7012967	0.9341586
Ehd3	-0.0581737	5.0844919	0.701332	0.9341586
Dchs1	-0.1250449	2.3617309	0.7013572	0.9341586
Gm10451	0.2738633	-0.655631	0.701366	0.9341586
Thap4	0.0568487	4.0232541	0.7014046	0.9341586
Nucb1	0.0495198	7.6780514	0.7014152	0.9341586
Tmbim4	-0.0475426	5.3562533	0.7015882	0.9341586
Cd7	0.0969642	2.6283683	0.7017938	0.9341586
Dda1	0.0529333	5.2941302	0.7018597	0.9341586
Sorbs3	0.0849224	3.0776548	0.7019205	0.9341586
Ap3d1	0.0460642	6.5634435	0.7019603	0.9341586
Peli1	-0.0459226	5.6252378	0.7019639	0.9341586
Gm36591	-0.2240448	-0.9753139	0.7020047	0.9341586
D330023K18Rik	0.2603855	-0.1779258	0.7022024	0.9341586
Ralbp1	-0.0441239	6.8255264	0.7022096	0.9341586
Rab5c	-0.0444625	7.7545278	0.7023745	0.9341586
Tle2	0.1122308	3.0182558	0.7023779	0.9341586
Shpk	0.1134592	1.5672621	0.7024186	0.9341586
Pisd	-0.0540725	4.6356761	0.7025245	0.9341586
Zeb2	-0.0482126	7.544336	0.7026102	0.9341586
Btk	-0.0577349	6.3571245	0.7026554	0.9341586
Ltbp1	-0.051469	6.2343723	0.7027074	0.9341586
Oas1b	0.0954141	2.2685833	0.7027332	0.9341586

Lefty1	0.1397738	1.7348576	0.702741	0.9341586
Sned1	0.0787	4.0674917	0.7027533	0.9341586
4930577N17Rik	-0.13254	0.9421065	0.7029119	0.9341586
Gm6807	0.1968958	-1.0543523	0.7029479	0.9341586
Man1a	-0.0472423	6.6496687	0.7029598	0.9341586
Polb	-0.0558327	4.3548884	0.7031227	0.9341586
Prkar2b	-0.0476215	8.0881635	0.7031699	0.9341586
Clic1	-0.0479389	7.7019184	0.7031931	0.9341586
Kcnj14	-0.296702	-0.8601285	0.7033104	0.9341586
Exo1	0.0628021	4.9005832	0.7033663	0.9341586
Zup1	0.0618756	4.3350399	0.7033688	0.9341586
Fam83f	-0.1442317	-0.1002553	0.7033728	0.9341586
Dusp5	0.145304	1.431539	0.7034133	0.9341586
Focad	0.077359	3.8430964	0.7034703	0.9341586
Gm7107	-0.3030368	-0.9833721	0.7035039	0.9341586
Gtpbp10	0.0743523	3.6216818	0.7035353	0.9341586
Mtmr10	0.0829934	3.422721	0.7035756	0.9341586
Morf4l1	-0.0484953	8.1860683	0.703596	0.9341586
Zfp54	0.0888908	2.1631418	0.7036891	0.9341586
2700012I20Rik	-0.1623093	0.2148623	0.7037656	0.9341586
Cbln1	-0.146124	1.5159984	0.703983	0.9341586
Timm21	-0.0636178	4.4197108	0.7040192	0.9341586
Esd	-0.0405847	6.8886472	0.7040672	0.9341586
Slc25a46	-0.0456256	5.8255011	0.7040871	0.9341586
Ap3s2	0.0516595	4.6417542	0.7041153	0.9341586
Snw1	0.0417012	6.5115252	0.7041735	0.9341586
Rps6kb1	-0.0378809	6.2482073	0.7042744	0.9341586
Slc5a2	-0.1828783	-0.4508206	0.7042756	0.9341586
Senp2	0.0454317	6.8170214	0.7043002	0.9341586
Rcor2	0.0943354	3.5828525	0.7044678	0.9341586
Pip4k2a	0.0414253	6.9955383	0.7044733	0.9341586
Topbp1	0.050044	7.6261328	0.7044743	0.9341586
Gfod1	-0.0822934	4.8086586	0.7044819	0.9341586
Ampd3	0.0497995	6.7641789	0.7045	0.9341586
Adamts14	0.1359018	2.8686642	0.7045176	0.9341586
Chsy3	-0.1081744	2.2290182	0.7045464	0.9341586
Cdc42ep3	-0.0394038	6.3063195	0.7045869	0.9341586
Ifitm5	0.1653428	4.0181184	0.7046444	0.9341586
Grrp1	0.1204989	2.7045935	0.7046487	0.9341586
Pdzd2	-0.0956991	4.6173864	0.704673	0.9341586
Ttc9	-0.1898891	0.0532533	0.7053191	0.9346543
Zfp239	0.1319086	1.4801228	0.70538	0.9346543
Dip2a	-0.0779034	3.3097678	0.7053958	0.9346543

Igkv8-19	-0.1793129	2.3808426	0.7055479	0.9346543
Gm12568	0.2806077	-0.8665133	0.7055917	0.9346543
Zc3hav1	0.0470249	7.2225878	0.7056056	0.9346543
Ppp1r15b	-0.0462359	8.2953311	0.7056366	0.9346543
Gm6542	-0.1770032	-0.2263014	0.705646	0.9346543
Il4i1	0.2716365	-1.1189121	0.705669	0.9346543
Tle4	-0.059017	5.204456	0.7057085	0.9346543
Kbtbd11	-0.0736688	4.744509	0.7057143	0.9346543
Neurl2	0.154299	0.0688672	0.7058552	0.9346543
Cd276	0.1302692	3.9034537	0.7059505	0.9346543
Cdk17	-0.044832	5.9288081	0.7059651	0.9346543
Snrnp200	-0.0555937	7.2597847	0.7059819	0.9346543
Tbc1d14	-0.045069	5.5895122	0.7060825	0.9346543
Col5a2	0.1506773	9.2817621	0.706146	0.9346543
Fuca2	-0.0457027	6.342444	0.7061575	0.9346543
Rgl2	0.062627	5.6602414	0.7061886	0.9346543
Fam241a	0.0611523	6.2412848	0.7063231	0.9346981
Exo5	0.065932	3.4011757	0.7063419	0.9346981
Rragc	-0.0453473	6.0066624	0.7064048	0.9347018
Tmem185a	0.0579327	3.8546876	0.7065413	0.93478
Fgd5	-0.0940786	2.6669107	0.7065953	0.93478
Msl2	0.0458358	7.1390597	0.7067346	0.93478
Morf4l1b	0.0778574	2.8861558	0.7067552	0.93478
Grb10	0.0950798	5.3436816	0.7067878	0.93478
Nudt16	0.0913718	2.7045326	0.7068244	0.93478
Med21	-0.0683578	5.2000949	0.7069446	0.9348594
Mapkap1	0.0488185	5.3293977	0.7070466	0.9349148
Smim20	0.0664435	3.7808294	0.7073307	0.9350761
Eda	0.0933149	1.6394827	0.707431	0.9350761
Gm14586	0.0885887	3.6544997	0.707431	0.9350761
B3galt4	-0.0953047	1.8502627	0.7074902	0.9350761
Gli3	-0.1526812	1.9716818	0.7075429	0.9350761
Nab2	0.0867365	3.4616922	0.7077934	0.9350761
Tle1	-0.0768178	3.5218624	0.7078876	0.9350761
Gm9027	-0.1623321	-0.8482437	0.7079228	0.9350761
Clec2d	0.0663786	5.3688166	0.7079813	0.9350761
Ccdc58	-0.0826982	3.5211795	0.7079837	0.9350761
Zfp599	0.1182654	1.6840016	0.7080171	0.9350761
Mdh1	0.0464132	7.0197988	0.7080408	0.9350761
Dync2h1	-0.0994618	3.4198596	0.7081471	0.9350761
Tmub1	-0.086335	2.7986816	0.708166	0.9350761
Arpc1a	0.0430903	6.1194018	0.7082838	0.9350761
Gm29771	-0.2532323	-0.7148652	0.7084242	0.9350761

Cd209a	-0.1620356	1.0349984	0.7084932	0.9350761
Yes1	-0.0870927	3.4086783	0.7084991	0.9350761
Zcchc4	0.0632256	4.0357772	0.7085788	0.9350761
Chmp2a	0.0435514	6.5695951	0.7085921	0.9350761
Mx1	0.1741934	1.6402865	0.7087114	0.9350761
Arhgef18	-0.0626504	5.9517356	0.7087667	0.9350761
Tkfc	-0.0774702	3.0838441	0.7087794	0.9350761
Qdpr	0.0692293	4.8374567	0.7088446	0.9350761
Pdha1	-0.0439109	7.0621475	0.7088472	0.9350761
Prdx1	0.0583932	7.6111462	0.7088704	0.9350761
Lhfp	0.0780785	5.3683955	0.708942	0.9350761
Foxh1	0.2170191	0.4956759	0.709016	0.9350761
Trub1	-0.0776965	3.0999304	0.7091836	0.9350761
Nusap1	0.0565121	8.1778031	0.7092168	0.9350761
Atp5mpl	0.0708102	6.539144	0.7092567	0.9350761
Zfp219	0.0636927	4.7446775	0.7092913	0.9350761
Hells	0.0553853	6.4962932	0.7093232	0.9350761
Epg5	-0.0496177	5.4923297	0.7093553	0.9350761
Gm10036	0.1019352	1.6482525	0.7094252	0.9350761
Sap30bp	-0.0662998	4.8826552	0.7095732	0.9350761
Rnf168	-0.0409757	6.0225558	0.7096979	0.9350761
Mettl4	-0.0440615	4.8901214	0.7097307	0.9350761
Smim7	-0.0446415	5.602186	0.7097402	0.9350761
Cdin1	0.0654701	3.5932209	0.7097531	0.9350761
Cltb	0.0578276	4.3728946	0.7097555	0.9350761
Tagap1	0.0703876	3.9934866	0.7097761	0.9350761
Gm13331	-0.163698	-0.2715269	0.7097841	0.9350761
Hexdc	-0.0902495	2.4110891	0.7098536	0.9350761
Ncapg2	-0.054781	7.7996425	0.7100246	0.9350761
Brox	-0.0424082	5.845804	0.710026	0.9350761
Med6	0.0609231	4.1285422	0.7100577	0.9350761
Rbm15	0.0528085	6.0532751	0.7100879	0.9350761
Ggt7	-0.1755568	-0.6076131	0.7101687	0.9350761
Trnau1ap	0.0652548	4.2393303	0.7101745	0.9350761
Eml6	-0.177183	1.0396591	0.7105837	0.9355357
Palmd	0.1743446	1.8363061	0.7106775	0.9355801
Slbp	0.0542964	7.9640424	0.7108295	0.9355921
Hif1an	-0.0411485	5.3840569	0.7108309	0.9355921
Mag	-0.1600731	0.5297963	0.7109495	0.9355921
Poc1b	0.0552418	4.5174848	0.7109827	0.9355921
L3mbtl3	-0.0677501	4.360548	0.7109875	0.9355921
Mccc1	0.0893138	3.3365907	0.7111873	0.9357666
Prr12	0.0675928	5.1799187	0.7112832	0.9357666

Nrf1	0.04726	5.788604	0.7113005	0.9357666
Nudt22	0.0795684	3.3813369	0.7115042	0.9359554
Lactb	0.0524022	4.7834082	0.7117154	0.9360555
Adgrd1	-0.1231979	4.0978463	0.7117573	0.9360555
Gm9828	-0.2017099	-0.8989512	0.7117609	0.9360555
Pex7	-0.0649854	4.232138	0.7119543	0.9362307
Arhgap23	0.0521355	7.0941073	0.7120283	0.9362488
Gm32404	0.2901786	-0.2868911	0.7122085	0.9363361
5031425E22Rik	-0.0533693	4.1399304	0.7122345	0.9363361
Gm8451	-0.2032641	-0.8254304	0.7123066	0.9363361
Gm41677	-0.1685906	0.0586753	0.7123437	0.9363361
Cdc25c	-0.0768151	4.1906735	0.7123956	0.9363361
Tns1	-0.0443663	6.0254568	0.7125291	0.9364324
1600012H06Rik	-0.0507932	4.8219412	0.7127524	0.9364578
Zkscan6	-0.0595329	3.7852244	0.7128938	0.9364578
Ucma	-0.3798449	-0.7292701	0.7130458	0.9364578
Rpp21	0.0644047	3.8625091	0.713128	0.9364578
Fan1	-0.0820369	2.702772	0.7131969	0.9364578
Bex6	-0.1373164	1.471461	0.7132	0.9364578
Ttc8	0.1670926	1.5309398	0.713248	0.9364578
Ostc	0.0575559	6.2509294	0.7132617	0.9364578
Tbx15	-0.1133067	3.0791996	0.713271	0.9364578
Ahctf1	0.0468543	7.1281614	0.7132887	0.9364578
Dnmt3b	0.0934736	2.7826142	0.7132939	0.9364578
Plekha5	0.0884059	4.0542422	0.7134341	0.9364578
Ppme1	0.0439783	4.9305875	0.713452	0.9364578
Mrpl27	0.0620435	4.4911502	0.7134525	0.9364578
Dgke	0.083834	3.0605757	0.7135196	0.9364578
Rfx3	-0.0906169	3.6170848	0.7135258	0.9364578
Alg10b	-0.05419	5.2875456	0.7136183	0.9364578
H13	0.0448858	6.8846936	0.7136321	0.9364578
Rnaset2a	-0.0957849	5.6111839	0.7139452	0.9367838
Psmb7	0.0446565	6.7683455	0.7140264	0.9367838
Mphosph8	0.0493915	5.0324275	0.7140612	0.9367838
Thap11	0.04584	5.1021376	0.7143367	0.9370518
Zfp780b	0.0751274	3.147545	0.7145232	0.9370518
Pigh	-0.0661264	3.3366012	0.7145705	0.9370518
Ippk	-0.0631352	3.6150748	0.7147312	0.9370518
Serpina3b	-0.1593324	3.0329003	0.714792	0.9370518
Bad	0.0556506	4.4761757	0.7148024	0.9370518
Dnajc27	0.07342	3.0916729	0.7148041	0.9370518
Rexo4	0.0507557	4.9396637	0.7148641	0.9370518
Peg3	0.1495436	2.2814332	0.7148822	0.9370518

Lancl1	-0.067851	3.8955027	0.714909	0.9370518
Acvr1	0.0947476	3.5416165	0.7149404	0.9370518
Zhx3	-0.0887994	2.7248351	0.7149885	0.9370518
Wdr60	-0.0926743	3.2868457	0.7152059	0.9370619
Uqcc1	0.0547163	4.3749745	0.7152542	0.9370619
Gga3	0.0519825	5.0402561	0.715259	0.9370619
Pes1	0.0458052	6.086244	0.715326	0.9370619
Foxf2	-0.2841414	-0.6353326	0.7153928	0.9370619
Anxa4	-0.0617076	4.9620947	0.7154507	0.9370619
Gm12216	-0.190418	-1.0479196	0.7154553	0.9370619
Tbc1d7	-0.100167	2.2329229	0.7155015	0.9370619
Gnptab	-0.0524701	6.3696748	0.7156199	0.9370619
Rusc2	-0.0973917	2.5034701	0.7156289	0.9370619
Dnmt3l	-0.2400536	-0.5221887	0.715702	0.9370619
Ncapg	0.0587859	7.187474	0.7157191	0.9370619
Cers2	0.0437219	6.7941803	0.7159679	0.9373087
Ebp	-0.0450981	5.1529237	0.7161583	0.9373546
Rpusd2	0.0829397	3.0430816	0.71648	0.9373546
Cdkl1	0.1088731	3.627046	0.7165177	0.9373546
Unc5cl	0.1326717	2.4208475	0.716529	0.9373546
Ints3	0.0561078	5.529862	0.716581	0.9373546
Psmc1	0.0463446	6.8333926	0.7166829	0.9373546
Fsd1l	-0.0868802	2.5528958	0.7166853	0.9373546
Pcdh1	-0.1291543	1.5150652	0.7167512	0.9373546
Ube2i	0.0409108	6.8501356	0.7167685	0.9373546
Smu1	0.0421875	6.2952859	0.7167956	0.9373546
Sec31a	0.0565701	6.7660697	0.7168707	0.9373546
Pds5a	-0.0455232	8.111352	0.7168831	0.9373546
Vis1	0.1760204	0.8528383	0.7169114	0.9373546
Vps36	-0.0440322	5.6856725	0.7169115	0.9373546
Gm10335	0.0991559	1.4275382	0.716913	0.9373546
Orai3	0.051628	4.4688526	0.7169672	0.9373546
1700102P08Rik	0.1220906	0.8543566	0.7171336	0.937466
Acyp1	-0.0628474	4.0203754	0.7172072	0.937466
Tuba1a	-0.0526283	6.3684057	0.7172845	0.937466
Rac1	-0.0437483	8.5011504	0.7174406	0.937466
Bard1	0.0541057	5.2840066	0.717553	0.937466
Pomk	0.0744879	3.0319115	0.7175573	0.937466
Robo4	-0.066909	3.7401948	0.7175628	0.937466
Slc49a4	-0.0464289	4.675198	0.7176407	0.937466
Pias2	-0.0446306	5.2671704	0.7177556	0.937466
Rpl23a	0.0540637	10.126292	0.7178003	0.937466
Gm24105	0.2387459	-0.7313376	0.7178252	0.937466

Gm13835	0.1141466	1.6171182	0.7179017	0.937466
Gcn1	-0.0501828	5.6076915	0.7180725	0.937466
Gm4739	-0.0640538	4.0652752	0.718091	0.937466
Ramac	-0.0465782	5.2421377	0.7181038	0.937466
Acsl4	-0.0444725	6.0014587	0.7181583	0.937466
Zfhx2	0.0967687	2.3844002	0.7181601	0.937466
Atat1	0.0883147	2.6831224	0.7182355	0.937466
Prmt3	0.0496078	4.6712521	0.7183318	0.937466
Alx4	0.1388986	1.1126658	0.7184033	0.937466
Zfp263	0.0536181	5.0946737	0.7184283	0.937466
Mtfmt	0.0833835	2.912188	0.7184864	0.937466
Ssh3	0.0641674	3.5764825	0.7186225	0.937466
Ehd1	-0.0413032	6.9544066	0.7187049	0.937466
Tox4	0.0461001	5.8002622	0.718735	0.937466
Fuz	-0.0765273	2.9138729	0.718791	0.937466
Epb41l1	-0.0894488	2.770619	0.7187955	0.937466
Ssbp2	0.0562316	4.9590287	0.7188098	0.937466
Igbp1	0.050207	5.4697955	0.7189669	0.937466
Mcm5	0.0558908	8.1647141	0.719011	0.937466
Zfp319	-0.0824348	3.5636477	0.719244	0.937466
Fam120c	-0.0686485	3.4003288	0.7192474	0.937466
Mex3a	0.0910349	2.7985938	0.7194488	0.937466
Prkd1	0.2036243	0.0328426	0.7194516	0.937466
Cmss1	-0.0684891	2.9916533	0.7194967	0.937466
Atad3aos	-0.2108574	-0.2446022	0.7195212	0.937466
Arl5a	-0.0447086	6.4269482	0.7195633	0.937466
1110065P20Rik	-0.0987615	2.578004	0.7196458	0.937466
Enkd1	0.0790838	2.989839	0.7196547	0.937466
Gm6249	0.1835664	0.3138496	0.7196983	0.937466
Runx1t1	0.1142166	1.7593348	0.7197209	0.937466
Emcn	0.0798893	3.616184	0.7197546	0.937466
Hsd17b4	-0.0436791	5.7521919	0.7199197	0.937466
1500004A13Rik	0.241602	-1.0566081	0.7199222	0.937466
Alpl	0.1268902	7.5905789	0.7199417	0.937466
Gm5817	0.1362293	1.2515458	0.7199893	0.937466
Utp20	-0.0611082	5.0636156	0.7200525	0.937466
Katnal1	0.0866051	4.4075893	0.7200572	0.937466
Stk35os1	-0.1034131	1.9828128	0.7200597	0.937466
Gm50340	-0.1236104	0.8830661	0.720066	0.937466
Tmem29	0.0688649	3.7883547	0.7202575	0.9374787
Cstf3	0.0412947	5.3453168	0.7202636	0.9374787
Smad5	-0.0418855	5.7696047	0.7202873	0.9374787
Rapgef4	-0.1238432	3.3637587	0.7203608	0.9374787

Arhgap31	0.0617162	5.3389313	0.7203771	0.9374787
Zfp449	0.1588782	1.3196664	0.7205241	0.9375915
Mcm6	0.0499407	8.4521778	0.7208737	0.9379679
LOC118568309	-0.1098033	1.0771511	0.7211162	0.9382051
Rnf2	0.0436214	5.5763529	0.7211805	0.9382102
Snapin	-0.0429676	4.9024086	0.7213406	0.9383219
Camsap1	0.0559692	4.6672907	0.721427	0.9383219
Slc25a26	0.0807344	1.9984848	0.7214473	0.9383219
Entpd3	-0.136315	5.3656381	0.721675	0.9383989
Rasgrf2	0.1050932	1.7701003	0.7217085	0.9383989
1700008J07Rik	0.0953773	1.5182005	0.7217354	0.9383989
Btnl4	-0.2787476	-0.1649633	0.7219142	0.9383989
Snap47	0.0775897	2.9692641	0.7219218	0.9383989
Lcmt1	-0.0606861	4.0849596	0.721931	0.9383989
Shtn1	-0.0799513	4.2929861	0.7221232	0.9383989
Tet3	-0.0420057	7.0236812	0.7221411	0.9383989
Nhlrc1	0.2898072	-0.864016	0.7222138	0.9383989
Ift88	-0.0844319	2.24491	0.7222372	0.9383989
Wdr35	-0.0723471	2.5108465	0.7222764	0.9383989
Gatm	-0.0587874	6.7694584	0.722285	0.9383989
Drap1	0.0381862	6.3406452	0.7222908	0.9383989
Neat1	-0.0777098	8.5770919	0.7224977	0.9385893
Smarcc2	-0.0486674	7.4271446	0.7226382	0.9386934
Vsig10l	-0.0890023	2.4646479	0.7230098	0.9388454
Gm52041	-0.1772267	-0.1378244	0.7230264	0.9388454
Il15ra	0.0742634	2.9872541	0.723145	0.9388454
Snx3	0.0398181	7.1282707	0.7232445	0.9388454
2610524H06Rik	0.0831069	3.4788697	0.7233226	0.9388454
Clstn1	-0.0519441	4.5378088	0.7233271	0.9388454
Ttc13	0.0478944	5.5547807	0.7233445	0.9388454
Riox2	-0.0510646	4.592942	0.7233782	0.9388454
Errfi1	-0.1102834	4.949922	0.7233789	0.9388454
Vkorc1l1	-0.0402174	6.2941738	0.7234513	0.9388454
Plagl2	0.055499	6.2546903	0.7234647	0.9388454
A4galt	0.1617838	0.537433	0.7236373	0.9388454
Rnf113a2	-0.0706377	4.1865309	0.7236453	0.9388454
Ap3m1-ps	0.2035868	-1.0428146	0.7236591	0.9388454
Rtn3	-0.0429092	7.9280637	0.7236665	0.9388454
Limd2	-0.0513383	7.1256888	0.723721	0.9388454
Zdhhc23	0.1269545	1.3000835	0.7239084	0.9390102
Pgghg	0.0619821	3.9695218	0.7240157	0.9390404
Nat2	0.0632354	4.3534943	0.7241535	0.9390404
Synrg	-0.054124	5.5651784	0.7241731	0.9390404

Helb	0.0496005	5.7762053	0.7242587	0.9390404
Nup160	0.0473365	6.3893713	0.7243875	0.9390404
Lamtor1	-0.0383814	5.9508746	0.7244085	0.9390404
Rif1	-0.0474328	7.0685079	0.7244132	0.9390404
Slc25a13	-0.0742207	3.8947322	0.7244147	0.9390404
Rpl17-ps3	0.1196529	1.4456911	0.7245715	0.9390689
Coq10b	0.0552339	4.6048419	0.7245828	0.9390689
Lbr	-0.0447756	8.9184051	0.7246244	0.9390689
Cspg4	-0.1126052	3.2004961	0.7246781	0.9390689
Traf3ip2	0.0982926	2.5665589	0.7247887	0.9391339
Hyal2	0.0665297	3.5760909	0.724887	0.939183
Rfx1	-0.0583798	4.5384307	0.7249707	0.9392133
Sqstm1	-0.0378486	7.3690561	0.7250876	0.9392482
Gm6254	-0.1406709	0.0671853	0.7251729	0.9392482
Avpi1	-0.1650772	1.25824	0.7251932	0.9392482
Slc2a10	-0.1392594	1.7461568	0.7252393	0.9392482
Gimap4	0.0585624	5.2055982	0.7253774	0.9393079
Sf3b6	0.0610078	6.1905091	0.7254524	0.9393079
Gm19810	0.1590741	0.3270629	0.7254665	0.9393079
Eef1a1-ps1	0.1077775	2.003473	0.7255733	0.939368
Rxylt1	-0.0609497	3.7141185	0.726043	0.9398979
Tnfrsf11b	-0.1493078	2.0564075	0.7261801	0.9399972
Ctla2b	-0.0714062	3.9863621	0.7262824	0.9400514
Atxn2l	0.043353	7.5158625	0.7265462	0.9403145
Tlr7	-0.0700145	4.5477693	0.7268734	0.9405485
Rgp1	0.040629	6.2295753	0.7268737	0.9405485
Sox18	-0.0602556	3.6806762	0.7269084	0.9405485
Fbxw8	-0.0451812	5.2369897	0.7270846	0.9406982
Rchy1	0.0397345	5.6453378	0.7271481	0.9407021
Gtf2f2	0.0498891	4.6783554	0.7274071	0.9408573
Tbck	0.0615754	3.9790537	0.7274203	0.9408573
Mllt11	0.0918723	2.6468682	0.7274957	0.9408573
Ttc41	0.1815278	0.7130764	0.7275807	0.9408573
Dnttip2	0.0386975	5.8487254	0.7276143	0.9408573
Zfp280c	-0.0522353	4.3019856	0.7276588	0.9408573
Slc9a8	0.055235	4.8540227	0.7276915	0.9408573
Traf6	0.0472064	5.4637083	0.7278124	0.9409354
Acyp2	0.2062828	2.5970286	0.7279819	0.9409418
Apold1	-0.0830979	2.951338	0.7280681	0.9409418
Ndufb1	-0.0566834	6.4504029	0.7280922	0.9409418
Zfp472	-0.0520872	3.6182807	0.7281025	0.9409418
Cldn12	-0.0984559	2.6265153	0.7281198	0.9409418
Acp5	0.1045137	9.9440327	0.7282222	0.9409959

Nans	0.0432811	5.7477329	0.7282976	0.9410152
Pid1	0.0554323	4.1984899	0.7285336	0.9412419
Gm12992	0.0966673	1.4418943	0.7286196	0.9412749
Asnsd1	-0.0501862	6.4989674	0.7288299	0.9412856
Gm51436	0.1518198	0.6071064	0.7289218	0.9412856
Wapl	-0.0478103	8.1918775	0.7289604	0.9412856
Zfp553	0.0756825	3.6939753	0.7289639	0.9412856
Shc2	-0.1342867	3.5256793	0.7289747	0.9412856
Cttnbp2nl	-0.0597873	3.830825	0.728991	0.9412856
Rbl1	-0.0494247	6.8980961	0.7291295	0.9413565
Txn1	-0.0457233	8.4139322	0.7292161	0.9413565
Rasgef1a	-0.2278923	-0.3424294	0.7292275	0.9413565
Chrnb2	0.2355955	-0.5507877	0.7293209	0.9413989
Dhx32	-0.0662478	3.6861123	0.7294541	0.9414928
Lrrc57	0.0576328	3.879621	0.7295722	0.9415237
Gm38914	-0.1835669	-0.4696196	0.7296175	0.9415237
5033421B08Rik	-0.0992363	1.4335366	0.7296597	0.9415237
Thap1	0.0748854	3.4342317	0.7298074	0.9415759
Zcwpw1	0.1131009	2.2251808	0.7298212	0.9415759
Ube2e2	0.1369657	1.9475218	0.7299717	0.9416919
Atp5pb-ps	-0.1379681	0.5250984	0.7301813	0.9418571
C2cd2	-0.0470204	4.4895	0.7302599	0.9418571
Luc7l3	0.0496074	7.8961204	0.7302814	0.9418571
Stau2	0.0998349	3.2715163	0.7304441	0.9419202
Cers5	0.0495161	4.6089194	0.7304514	0.9419202
Chml	-0.0612289	4.3323995	0.7307154	0.9421824
Gnao1	0.1181187	1.5860595	0.7308406	0.9422659
Apbb1	0.1162731	2.7098264	0.7309623	0.9422837
Miip	0.0661425	4.8124104	0.7310691	0.9422837
Mdfi	0.115166	2.7596145	0.731115	0.9422837
Gm1673	0.1788552	1.4329753	0.7311406	0.9422837
Zfp458	-0.1360885	1.1085727	0.7311574	0.9422837
Ncoa7	-0.061928	6.2280084	0.731278	0.9423443
Ccdc80	-0.0988004	6.0508961	0.7313825	0.9423443
Six1	0.0997673	3.035201	0.7314377	0.9423443
Usp45	0.0565992	6.2367242	0.7314467	0.9423443
Cul5	-0.0376092	5.6008705	0.7315699	0.9424249
Ubr7	0.0418197	5.8813624	0.7316899	0.9424285
Ggt5	-0.0643652	4.196063	0.7316938	0.9424285
Smg6	0.0501693	5.7546526	0.7320042	0.9424712
Ndufa1	0.0509276	6.0681733	0.7320126	0.9424712
Tpt1-ps6	0.1501254	-0.1011668	0.7320142	0.9424712
Wdr5	0.0393051	6.1138338	0.732022	0.9424712

Evc	0.1160964	2.0169059	0.7320576	0.9424712
Gm9761	-0.1226437	1.9437969	0.7320972	0.9424712
Ap4s1	0.0582559	4.2810868	0.7321825	0.9424712
Rasal2	-0.0718056	3.629342	0.7322626	0.9424712
Dusp27	0.207109	0.3292135	0.7322723	0.9424712
Cdk20	0.1189645	1.7125951	0.732649	0.9427328
Katnip	-0.0581644	3.7310601	0.7326817	0.9427328
Ankrd7	-0.1497167	0.5524493	0.7327367	0.9427328
Asph	0.0512241	5.8630526	0.73275	0.9427328
Gspt1	0.0367763	7.3881825	0.7327787	0.9427328
Vmac	0.0694141	2.8355343	0.7328604	0.94276
Tmsb15b1	-0.1957869	-0.6083337	0.7330918	0.9429796
Pigp	-0.0695736	4.1447162	0.7333999	0.9432531
Ammecr1	0.0418973	6.2394704	0.733444	0.9432531
Gng5-ps	-0.1978946	-0.4823794	0.7337515	0.9432531
Dctn2	0.0372915	6.5099539	0.733779	0.9432531
Dhx9	-0.0497158	8.2455963	0.7338102	0.9432531
Fem1b	-0.0380188	6.2273168	0.7338283	0.9432531
Tgfa	-0.1590262	-0.2420494	0.7338878	0.9432531
Pla2g15	-0.0437046	5.2656284	0.7339286	0.9432531
Trim34b	0.4127099	1.7850517	0.734014	0.9432531
Fbxo22	0.0422632	5.2513913	0.7340457	0.9432531
Lrig2	-0.0445025	4.4728938	0.7340865	0.9432531
Nras	-0.0386629	6.555959	0.7342575	0.9432531
Psma7	-0.0440525	7.0859559	0.7342889	0.9432531
Mms19	0.0446632	5.1032968	0.7342994	0.9432531
Lekr1	0.1631544	0.5269953	0.7343339	0.9432531
Mitf	-0.0754607	3.3671388	0.7343375	0.9432531
Phactr4	0.0503272	5.6608198	0.7343866	0.9432531
Aldh3a1	0.148435	0.0336994	0.734396	0.9432531
Mtmr2	0.0402881	5.4295323	0.734536	0.9433551
Trappc1	0.0490543	5.1330774	0.7346416	0.9434127
Gm15787	-0.20289	-0.8805324	0.7349934	0.9435542
Ehd4	-0.0470385	6.385236	0.7350369	0.9435542
Vamp1	0.0722038	3.9689537	0.7350383	0.9435542
Gm11613	-0.13894	1.3148702	0.7350464	0.9435542
Emsy	0.0445804	5.61267	0.7351515	0.9435542
Snd1	0.0385424	6.8673813	0.7351611	0.9435542
Zbtb7a	0.0438197	7.2816542	0.7351764	0.9435542
Prpf38a	-0.0516632	5.6376348	0.7353031	0.943639
Haao	0.0920438	2.3829874	0.7353777	0.9436569
Pdxk	-0.0589641	4.4098675	0.7354495	0.9436711
Rpl7l1	0.0345802	6.2973969	0.7355278	0.9436938

1500015A07Rik	0.1080323	2.2130814	0.7357145	0.9438554
Gm38561	-0.0452285	4.7244291	0.7357792	0.9438606
Nup210l	0.1477065	0.8794669	0.7358921	0.9439275
Gm17344	-0.0988301	2.189443	0.7360249	0.9439504
Gm5424	-0.1829818	-0.465017	0.7360792	0.9439504
Osbpl5	-0.0760897	3.374874	0.736092	0.9439504
Abcd3	-0.0417965	5.2513446	0.7361616	0.9439619
Angpt4	-0.1021633	3.3074462	0.7363178	0.9440843
Mterf4	0.0729186	3.334103	0.7366171	0.9441878
Ppif	-0.0412666	5.5257479	0.736669	0.9441878
Cep63	0.0460723	4.7367865	0.7366877	0.9441878
Gfm2	0.0436026	4.6760971	0.7367452	0.9441878
Bcl9	-0.0698476	4.0016799	0.7367603	0.9441878
Zfp869	0.0466146	5.2580621	0.7367627	0.9441878
Psma6	0.0376475	6.9882605	0.736826	0.9441912
Tax1bp3	0.0713724	3.1115752	0.736903	0.944212
2610044O15Rik8	0.1065767	2.3096628	0.7370337	0.9443017
Zap70	-0.1552549	1.3153053	0.7372282	0.9444295
Mlxipl	-0.1849951	-0.2448426	0.7372549	0.9444295
Eny2	0.0547371	6.0925204	0.7375833	0.9446183
Nemp1	-0.0481824	5.5129093	0.7376213	0.9446183
Mios	0.0425208	4.7770941	0.7376354	0.9446183
Lratd2	-0.0733429	3.4583763	0.7377635	0.9446183
C1galt1	-0.0566871	6.276595	0.7378067	0.9446183
Atl2	0.0406977	6.4567964	0.7378608	0.9446183
Rab11b	0.0370779	6.947772	0.7378893	0.9446183
Celf1	0.0423857	7.8595363	0.7379176	0.9446183
Asb3	-0.0481322	3.9570234	0.7379489	0.9446183
Gm8292	-0.2131557	-0.1227962	0.7381464	0.9447236
Axin1	-0.0490008	5.4026273	0.73819	0.9447236
2900076A07Rik	-0.1191948	1.1435458	0.7383321	0.9447236
BC002059	0.0807421	3.6182187	0.738349	0.9447236
Rrp15	0.0601927	4.1937872	0.7384055	0.9447236
Smim24	0.1380354	1.3731879	0.7384204	0.9447236
Fig4	-0.0557683	5.008721	0.7384563	0.9447236
Acmsd	-0.086786	2.9153633	0.7387964	0.9450251
Zfand1	0.0833429	3.6179392	0.7388135	0.9450251
Atp5c1	-0.0392042	7.6508721	0.7389939	0.9451271
Chmp1a	0.0414852	5.8466929	0.7390276	0.9451271
Stap2	-0.1017521	2.4154425	0.7391089	0.9451271
Synj2	-0.0769003	3.5074008	0.7391363	0.9451271
Mthfr	0.0555512	3.8525209	0.7392504	0.9451312
Atp5md	-0.0635831	6.3371854	0.739274	0.9451312

Surf4	-0.0431793	7.3534585	0.7393218	0.9451312
Gas2l3	0.0503163	5.036113	0.7394072	0.9451627
Prpf8	-0.0607833	8.1554091	0.7395734	0.9452974
Serhl	0.0770717	3.1710479	0.7396492	0.9453167
Hsbp1	0.0397983	6.2188377	0.7398701	0.9454481
Cdk12	-0.0465562	6.7272122	0.739926	0.9454481
Stard7	0.0356578	6.5578591	0.7399528	0.9454481
Gm10615	-0.1258915	1.0915704	0.7400157	0.9454481
Scamp3	-0.0710402	3.7265117	0.740056	0.9454481
Gpr161	-0.1689973	0.2804645	0.7401599	0.9455032
Arcn1	-0.0416304	7.6864862	0.7402465	0.9455361
Gm46965	0.2064957	-0.819365	0.7403266	0.9455608
Rpl34-ps1	0.220587	4.3252808	0.7403877	0.9455612
Mc5r	0.1062741	1.5465639	0.7406056	0.9457082
Plcd3	0.1480408	0.4726529	0.7406244	0.9457082
Nek6	-0.0717012	3.1884259	0.7412743	0.9464315
Mier3	0.0489655	6.66734	0.7413125	0.9464315
Rpp38	0.0981324	2.3615482	0.7418329	0.9467348
Macroh2a1	0.0514939	8.3003908	0.741913	0.9467348
Rnaseh2c	0.0556835	6.5336181	0.7419501	0.9467348
Egln2	0.0388192	5.4412705	0.7420522	0.9467348
Ltn1	-0.0382603	6.0994492	0.7421117	0.9467348
Zfp551	0.1248043	0.9928379	0.7421197	0.9467348
4930430E12Rik	0.2063928	0.5280037	0.7421455	0.9467348
Atp5l	-0.0599597	6.8774407	0.7422777	0.9467348
Gm2518	0.240131	-0.6425662	0.7422891	0.9467348
Slc52a3	0.1872652	0.3158663	0.7423088	0.9467348
Nudt16l1	0.043933	4.5570356	0.7423285	0.9467348
Sptlc1	0.0475926	5.0264841	0.7423626	0.9467348
Coro1b	0.035521	7.1468337	0.7424241	0.9467348
Lpar4	-0.13435	0.3366369	0.7424344	0.9467348
Haus2	-0.0480195	4.3608572	0.7425145	0.9467348
Zbtb21	-0.0603996	3.4805603	0.742524	0.9467348
Mat2a	0.0451147	7.8593704	0.7426375	0.9468019
Spata2	-0.0568383	4.7474901	0.7427647	0.9468865
2310047D07Rik	0.1154377	0.3488854	0.7431751	0.9473321
Hk1	0.0521235	8.0753484	0.7433428	0.9473624
Gm8439	-0.2485391	-1.0773127	0.743366	0.9473624
Zfp930	0.0556563	4.3213018	0.7434442	0.9473624
Lrp11	-0.1235501	0.5981696	0.743458	0.9473624
Snx12	-0.0434842	5.2396914	0.7435035	0.9473624
Atp6v0a2	-0.0391849	5.8272708	0.7435658	0.9473642
Git2	-0.0469922	6.8138306	0.7437677	0.94738

Ppp1ca	-0.0418396	8.415995	0.7439141	0.94738
Gm5785	-0.0624217	3.4411538	0.7439168	0.94738
Rps6ka1	-0.0470487	7.5557192	0.7439209	0.94738
Adamts17	-0.1562611	0.4015684	0.7439347	0.94738
Derl3	-0.119436	1.5763757	0.7439869	0.94738
Icos	-0.2630414	-0.5096362	0.7442029	0.94738
Ubl3	-0.051381	6.380894	0.7443279	0.94738
Rapgef5	0.0696359	3.6382664	0.7444034	0.94738
Pgrmc1	0.0397685	5.6602035	0.7444057	0.94738
Slc66a2	0.0485315	6.1219073	0.7445325	0.94738
Fmc1	0.0587524	3.2879118	0.7445739	0.94738
Rsrp1	0.060981	8.3882355	0.7446056	0.94738
Oxld1	-0.0891588	1.7241399	0.7446301	0.94738
Syf2	0.0423134	6.4724312	0.7446427	0.94738
Gpr84	0.0633404	3.7939101	0.7446883	0.94738
Med13	-0.0447263	7.3855995	0.7447459	0.94738
Tesmin	-0.1358031	0.4472158	0.744761	0.94738
Gm31619	-0.0828221	3.0075071	0.7447787	0.94738
3110045C21Rik	-0.2223537	-0.7783288	0.7448462	0.94738
Noxo1	-0.1554902	0.0901772	0.7449059	0.94738
Tpt1-ps5	0.1786033	-0.1799804	0.7449182	0.94738
Thrap3	0.0401503	7.936411	0.7450402	0.9474578
Gm10250	0.0978098	1.5702094	0.7451289	0.947493
Gm15801	0.1101135	0.8782744	0.7453028	0.94758
Rtca	0.0454518	4.7242756	0.7453191	0.94758
Dusp9	0.1763878	-0.4070329	0.7454294	0.9476427
Cux1	-0.0505757	7.0534888	0.7455579	0.9477287
Gm16124	-0.1207469	0.7978464	0.7456871	0.9477928
Sec24b	-0.0417202	5.5245738	0.7457303	0.9477928
Kif9	-0.1333115	0.183351	0.7458832	0.94786
Ppp1r2-ps4	-0.1686604	-0.9814206	0.745905	0.94786
Glrx2	0.0522603	4.7220735	0.746047	0.9478881
Rnf212	0.1024134	2.1445727	0.7460794	0.9478881
Lins1	-0.0579643	3.2491401	0.7461099	0.9478881
Il3ra	-0.1082291	3.3007911	0.7461997	0.9479248
Arpc4	-0.0399123	7.6980661	0.7462629	0.9479276
Psmd6	0.0417594	6.3096949	0.7463416	0.9479502
Lars	0.0564503	6.1932358	0.7464631	0.9480187
Sh2b2	-0.0792809	3.4822508	0.7465809	0.9480187
Zfp770	0.0512106	4.1077146	0.7466266	0.9480187
Mbd4	0.051131	5.1061211	0.7466394	0.9480187
Gm28809	-0.1757966	0.7434269	0.7469384	0.9483026
Cebpg	0.0403081	6.4649893	0.746985	0.9483026

Tm9sf1	-0.0339308	5.9316484	0.7471844	0.9483527
Cdk6	-0.0421124	6.5901967	0.7472504	0.9483527
Ccdc91	0.0576938	3.6068925	0.7472637	0.9483527
Tcf4	-0.0519472	6.4329321	0.7472683	0.9483527
Gm5165	-0.0770362	3.1203822	0.7475276	0.948369
Tnks2	-0.0350132	7.2737256	0.7475784	0.948369
Zmym4	-0.041298	5.0308586	0.7476172	0.948369
Pum1	0.04157	6.5671022	0.7476313	0.948369
Fbxo48	-0.0681965	4.8845623	0.7476339	0.948369
Eif4g1	0.0453559	8.6389194	0.7476469	0.948369
Psmb3	0.0402751	6.2604117	0.7479683	0.9485378
Sla2	-0.0739044	2.4367856	0.7480519	0.9485378
Serpina12	0.1284487	0.2360345	0.7481436	0.9485378
Atp8b2	0.0552103	5.1228875	0.7482394	0.9485378
Tarsl2	0.0656034	5.0083572	0.748361	0.9485378
Gnat2	0.315003	-1.0203925	0.7483676	0.9485378
Mical3	0.0419447	6.1703755	0.7484345	0.9485378
Tor1b	0.0372487	5.4943062	0.7485304	0.9485378
Cebpb	-0.0915061	6.3644774	0.7486328	0.9485378
Letm1	-0.0368763	5.2467979	0.7486637	0.9485378
Aph1a	0.0477328	4.6364966	0.7486729	0.9485378
Uhrf2	-0.0347784	6.2372179	0.7487517	0.9485378
Spata5	0.0498213	3.8225968	0.7487622	0.9485378
Uba5	0.0390536	5.3142801	0.7488989	0.9485378
Agt	0.0536492	4.83573	0.7490234	0.9485378
1110032A03Rik	0.0676695	3.532086	0.7490897	0.9485378
Kcnip3	0.1379714	1.0218525	0.7490954	0.9485378
Arl3	-0.0716958	3.3389306	0.7491308	0.9485378
Cntn1	-0.1851345	-0.0057452	0.7491413	0.9485378
Lztfl1	0.0426135	4.5323368	0.7491441	0.9485378
Sec22a	-0.0703401	3.0507798	0.7492396	0.9485378
Cep290	0.0532482	4.7924692	0.7492737	0.9485378
Hsd17b10	-0.0361784	5.2789724	0.7492789	0.9485378
1700037C18Rik	0.1824069	0.346769	0.7492911	0.9485378
Cops6	0.0381292	6.0449537	0.7493046	0.9485378
Cdc45	0.0420057	5.840753	0.7495667	0.9487923
Gm7327	-0.167234	-0.5798946	0.7498607	0.9490872
H2-T23	0.0557316	6.2678697	0.7500005	0.9491869
Zfp943	-0.0467129	4.6398679	0.7501664	0.9492552
Tpsb2	-0.235639	-1.2112635	0.7501765	0.9492552
Unkl	-0.1037218	3.1333278	0.7503255	0.9493666
Fam172a	0.0418758	5.3717985	0.7505008	0.9494224
Chic1	-0.1400866	0.6126938	0.7505862	0.9494224

Rsph3a	0.0612018	3.3957453	0.750613	0.9494224
H2bu2	0.1069699	1.268484	0.7506138	0.9494224
Gm11274	0.2385059	-0.6826675	0.7507824	0.9494379
Dlgap5	0.0547248	6.865405	0.7507875	0.9494379
Calr4	-0.1671708	-0.5109868	0.750866	0.9494379
Ccdc127	0.0371589	5.5014208	0.7509678	0.9494379
Ell2	0.0477648	6.8071553	0.7509977	0.9494379
Gm10269	0.1188333	0.7795884	0.7509981	0.9494379
LOC118567504	-0.1090606	1.4970392	0.7510636	0.9494379
Tmem230	0.0524513	3.9950715	0.7512713	0.9494379
Sik1	-0.0965868	5.3092182	0.7512904	0.9494379
Cast	-0.0356532	7.514279	0.7513152	0.9494379
LOC118568050	0.2026754	-0.6350734	0.7513281	0.9494379
Sart3	-0.037972	5.9202874	0.7513625	0.9494379
Psmg4	0.0602885	3.3945922	0.7514196	0.9494379
Tmod2	-0.1579466	-0.8572638	0.751509	0.9494422
Gm51953	-0.1481073	-0.5902354	0.7515451	0.9494422
Tlcd3b	-0.163805	-0.3388127	0.751615	0.9494533
Shfl	0.0646964	3.2003087	0.7517281	0.9494868
Gm38872	-0.1260077	0.4428228	0.7517635	0.9494868
Txlng	-0.047973	4.327232	0.7519221	0.9494961
Zhx2	0.0718177	3.9927456	0.7519808	0.9494961
Tmem94	-0.040985	5.1417193	0.7520006	0.9494961
5730480H06Rik	0.1553922	0.0393092	0.752015	0.9494961
Cbx7	-0.0661867	3.4393079	0.7521147	0.9495448
Prorsd1	-0.047545	4.2360066	0.7522123	0.9495706
Tgtp2	0.1347125	1.753149	0.7522572	0.9495706
Slc19a2	-0.0604405	3.0433929	0.7525328	0.9497703
Scara5	0.1089517	1.7203443	0.7526437	0.9497703
4933434E20Rik	-0.042297	5.1660855	0.7526575	0.9497703
Dvl1	0.0453079	5.1201926	0.7526685	0.9497703
Tmem175	0.0650238	3.5149673	0.7527566	0.9497703
Hs1bp3	-0.0568814	3.1909419	0.7527818	0.9497703
Thoc7	0.0449297	5.9620079	0.7529323	0.9498831
Cep120	-0.0360977	5.6626976	0.753038	0.9499394
Snhg3	-0.0746155	4.5277217	0.7531123	0.9499454
D130020L05Rik	-0.1115607	1.0445054	0.7531867	0.9499454
Dop1b	0.0403211	5.5785631	0.7533255	0.9499454
Rexo2	0.0399787	6.5573265	0.7534502	0.9499454
Pptc7	-0.0491261	5.1256935	0.7534801	0.9499454
Mageh1	0.1141946	2.1361437	0.7535228	0.9499454
Rcor3	0.0492348	4.4698314	0.7536057	0.9499454
Liph	0.1741113	0.0500337	0.7536063	0.9499454

Ntpcr	0.0661414	3.2327511	0.7538281	0.9499454
Wdr11	-0.0426908	4.8096251	0.7538296	0.9499454
Zfp275	0.0549429	4.2332554	0.7538644	0.9499454
Maged2	0.0618556	4.8861484	0.753877	0.9499454
Zscan22	-0.0621805	3.4473294	0.7539242	0.9499454
Nfxl1	0.0547933	4.4945439	0.7539835	0.9499454
Amd-ps3	-0.1129399	1.2334285	0.7540237	0.9499454
Gm10053	0.1263337	0.7460579	0.754066	0.9499454
Tsen34	-0.0523275	4.2724197	0.754081	0.9499454
Amdhd2	0.0574183	4.1539763	0.7541467	0.9499512
Mtpn	-0.041991	8.0968402	0.7542492	0.9499709
Tsen2	-0.0782115	2.1991999	0.754289	0.9499709
Tmco4	0.0705061	3.3436526	0.7543706	0.9499709
Cdipt	-0.0342848	5.6734956	0.754446	0.9499709
Epb41l2	0.059041	6.816164	0.7544677	0.9499709
Smim27	-0.063947	3.0987484	0.7546012	0.9500583
Ppp6r1	0.0407348	7.8420181	0.7546737	0.9500583
Fez1	0.2715284	-0.7127645	0.7547203	0.9500583
LOC118567393	0.2126067	1.0227016	0.7548493	0.9501437
Fam149b	0.0447933	4.2225478	0.7549613	0.9501801
Slc6a13	-0.1056178	2.2614817	0.7550003	0.9501801
Flywch2	0.169423	-0.0266756	0.7550981	0.9502261
Arrb1	0.0562082	8.4867845	0.755344	0.9503545
Cnnm3	-0.0446279	4.5662255	0.7554132	0.9503545
Foxo3	0.0489309	7.774462	0.7554537	0.9503545
1500011B03Rik	-0.0812384	2.9390407	0.7554775	0.9503545
LOC118567381	0.2248742	-0.5348731	0.7555585	0.9503545
B630019K06Rik	-0.1064388	0.7888919	0.7555667	0.9503545
Ercc6l	-0.052876	5.5376866	0.7559541	0.9507639
D17H6S53E	0.060172	3.1512056	0.7560144	0.9507639
Gm38664	-0.1772434	-0.1921436	0.756166	0.9507705
Kank4	0.1263473	0.977313	0.7562005	0.9507705
Ssr1	0.0363307	8.1331922	0.756203	0.9507705
Itpk1	-0.0524114	3.9838308	0.7564427	0.9509431
Mlycd	-0.0689133	3.1049455	0.7564626	0.9509431
Tmem8b	-0.113945	0.9770973	0.7565323	0.9509539
Zfp617	-0.0508947	4.4324983	0.7566046	0.950968
Rnf167	-0.0349929	6.6367065	0.7568295	0.9511008
Scaf1	0.0378801	6.3671655	0.7568326	0.9511008
Nars	0.0385217	7.4044748	0.7570374	0.951136
Mief1	0.0461743	4.8080218	0.7571177	0.951136
Mitd1	0.0517742	4.1066538	0.7571237	0.951136
Gm14098	0.2152997	-0.7160593	0.757172	0.951136

Ap3s1-ps2	-0.1838112	-0.74004	0.7573231	0.951136
Stxbp4	0.0559308	3.8515425	0.7573403	0.951136
Sh3pxd2b	-0.0849186	5.6030245	0.7573669	0.951136
Map4k4	-0.0488727	7.252095	0.7573835	0.951136
Zmym3	-0.05028	4.6074986	0.757411	0.951136
LOC118568194	0.1741866	0.4156391	0.757556	0.9512329
Cd69	0.0810782	3.0979531	0.7576104	0.9512329
Pdia3	0.0366081	9.0246376	0.7577236	0.9512359
Fam168a	-0.0360862	5.37334	0.7577352	0.9512359
Gga1	-0.0342478	5.5863149	0.7580767	0.9514924
Gm5835	-0.0975909	1.3231359	0.7580959	0.9514924
Igha	0.1948165	0.1393969	0.7581393	0.9514924
Wsb1	-0.0420797	6.6990435	0.7582056	0.9514924
Traip	0.0567042	4.4139567	0.7582454	0.9514924
Prkcsh	0.0369435	6.2319844	0.758344	0.9515395
Pttg1ip	-0.0328414	6.7194287	0.7584283	0.9515684
Fam204a	0.0564074	4.6180656	0.7585047	0.9515875
Aprt	-0.0443753	5.6346877	0.7587391	0.9517372
Gm6421	0.0653669	2.6366634	0.7588759	0.9517372
Dbn1	0.0943889	2.744828	0.758902	0.9517372
Smim15	0.036312	5.9676102	0.7589231	0.9517372
Ciao2b	-0.0498139	3.9593569	0.7589421	0.9517372
LOC115490409	0.333351	-1.3134122	0.7589912	0.9517372
Hmga2	0.233926	0.2726072	0.7591569	0.9518096
2310010J17Rik	0.1241019	1.1465774	0.7591713	0.9518096
Nudcd1	-0.0467897	4.4969045	0.7592962	0.9518895
Rad54l	0.0510378	5.7720149	0.7594584	0.9520161
Per3	0.0702292	3.9810394	0.7597712	0.9523315
Tcf7l2	0.0583376	3.7373614	0.7599491	0.9524087
Itga4	0.0794214	7.8766336	0.7599552	0.9524087
Laptm4b	-0.0667671	2.8136287	0.7600209	0.9524143
Slc35b3	0.0515757	4.1321755	0.7602542	0.9526298
Bri3bp	0.044625	5.581931	0.7605142	0.9527662
Parp9	-0.039171	4.9659853	0.7606374	0.9527662
Gm10052	-0.0689388	2.8600516	0.7606856	0.9527662
Hace1	0.0521434	6.2544279	0.7608851	0.9527662
Vash1	0.08212	3.5076342	0.7608972	0.9527662
Zfp260	-0.044913	5.2281468	0.7608975	0.9527662
Dennd1a	0.0454403	4.901	0.7609418	0.9527662
Fkbp1b	0.0797734	2.0515275	0.7609486	0.9527662
Usp6nl	-0.0381959	5.8707647	0.7610578	0.9527662
Lmo4	-0.0507914	6.5573951	0.7610714	0.9527662
Dusp19	-0.076801	2.2264201	0.7610744	0.9527662

Cdv3	0.031711	7.458968	0.7610981	0.9527662
Edf1	0.0322275	6.5341032	0.7615873	0.9532324
Yipf6	-0.0350984	5.7339047	0.761645	0.9532324
Zxda	-0.0864514	2.2097302	0.7616715	0.9532324
Mocs3	0.0667374	3.1002851	0.7617157	0.9532324
Mpc2	0.0476265	5.8340638	0.7619266	0.9534196
Tmem186	0.044278	4.4525602	0.7621924	0.9536756
Mmel1	0.2306001	0.0991969	0.7622807	0.9537093
Zfp661	0.0951113	1.7246635	0.7624017	0.9537765
Bcl2l11	-0.0385844	5.1426488	0.7624571	0.9537765
Pon3	-0.0429458	4.8586906	0.7625402	0.9537869
Gm42127	0.080907	3.1104116	0.762588	0.9537869
Prkag1	0.0346559	6.3754731	0.7627883	0.9538265
Hoxa10	0.0787401	2.5554329	0.7627973	0.9538265
Ccdc32	0.0530723	3.9246217	0.7628136	0.9538265
Dnmt1	-0.058881	8.3872237	0.762865	0.9538265
Aldh9a1	0.0425568	6.2043253	0.7630034	0.9538636
Cox6c	0.0644652	7.8943672	0.7630173	0.9538636
Rbm7	-0.0380523	6.0525252	0.7630911	0.9538792
Prg4	0.2100983	-0.8402591	0.763168	0.9538987
Tyw1	0.049132	4.2711954	0.7633351	0.9539701
Iqsec2	-0.0758492	3.2028452	0.7633478	0.9539701
Smarcd3	-0.1234487	1.4840008	0.7636288	0.9542394
Asb1	-0.0584404	6.8452692	0.7637407	0.9542394
Arfrp1	0.0431319	4.3655578	0.7637473	0.9542394
Trim13	-0.0925426	1.9148905	0.763882	0.954331
Btaf1	-0.0414675	6.9316751	0.764066	0.9544804
Rab7-ps1	-0.1514947	0.0400797	0.7642413	0.9544804
Ephb2	-0.1172595	0.9859099	0.7642999	0.9544804
Papss2	0.0452048	4.8928284	0.7643186	0.9544804
Cybrd1	0.1777228	0.7103783	0.7644012	0.9544804
Abhd18	-0.058511	2.8203837	0.7644364	0.9544804
AA388235	0.1210724	0.9866853	0.7645028	0.9544804
Lsm5	-0.0566217	4.7935278	0.7645563	0.9544804
Ms4a8a	-0.1218979	1.2419154	0.7646434	0.9544804
Pknox1	0.0404098	5.2992265	0.7646787	0.9544804
Brk1	-0.0352528	6.6490875	0.7646903	0.9544804
Ephx1	0.0576648	3.8546657	0.7648359	0.9544804
Dnajb12	0.036712	5.4460813	0.7649071	0.9544804
Atp6ap2	-0.0347886	7.0943386	0.7649302	0.9544804
AI661453	0.1352325	0.0573599	0.7649742	0.9544804
Nelfb	0.03483	5.6357842	0.7649834	0.9544804
Rasgef1b	-0.0748531	2.9398703	0.7652292	0.9544818

Cd99	0.0558654	3.3426257	0.7652369	0.9544818
Cd5	-0.1040024	1.0657127	0.7652943	0.9544818
Fth1	0.042847	10.602246	0.7653615	0.9544818
Cilk1	-0.0494946	4.0396705	0.7653723	0.9544818
Ube2g2	-0.0323066	5.6881825	0.7654268	0.9544818
Slc6a6	0.0445112	7.2727669	0.7654828	0.9544818
Lzts3	-0.0966087	1.2922861	0.7655471	0.9544818
Cdh2	0.0871659	6.2054149	0.7655685	0.9544818
Trim44	0.0305458	6.3771866	0.7655982	0.9544818
Tsc1	0.0416179	4.9167397	0.7657274	0.9545663
Tln2	-0.0634536	3.3910849	0.7658839	0.954584
Gm38618	0.1843542	0.0786984	0.7659969	0.954584
Pus10	0.0484136	4.9418011	0.7660626	0.954584
Unc119b	-0.0407503	4.8160065	0.7661172	0.954584
Supt16	-0.0352357	8.2153675	0.7661459	0.954584
Gm14399	-0.085333	2.1931344	0.7663113	0.954584
Cdk2	-0.034366	6.5044488	0.7663506	0.954584
Ggnbp2	0.0375367	7.1719632	0.7663618	0.954584
Cacnb3	0.1368645	3.6204404	0.7664144	0.954584
Wdr89	0.088555	1.2626468	0.766506	0.954584
Hdhd2	0.0420108	4.4762009	0.7665086	0.954584
Carmil1	0.0959983	3.3737004	0.766535	0.954584
Ube4b	0.0376321	6.3428384	0.7665394	0.954584
Dock6	-0.0675762	3.6022252	0.7666041	0.9545881
Parva	0.0658913	4.1404442	0.7668414	0.95473
Aebp2	0.0357376	6.3787402	0.7668494	0.95473
Enpp2	0.0994599	3.0454554	0.7669022	0.95473
Slc36a3os	0.1953271	-0.7811093	0.767039	0.954799
Trappc12	-0.0453183	4.654	0.7670804	0.954799
Chuk	0.0315498	5.7354944	0.7671704	0.9548346
Tmem229b	0.0564672	5.5991909	0.7674836	0.9551156
Mmp15	-0.1109865	1.0035236	0.7675411	0.9551156
Pja1	0.0475607	4.8237327	0.7675803	0.9551156
Col3a1	0.1447935	6.6230934	0.767694	0.9551806
Wdr46	0.0577237	4.6911194	0.7678579	0.9553081
Sp7	0.1090573	5.6420148	0.7679221	0.9553115
Polr1c	-0.0396443	4.7894982	0.768083	0.9554353
LOC118568072	-0.1974221	-0.8700745	0.7681538	0.955447
Uchl5	0.0433545	6.1629758	0.768431	0.9555024
Vsig4	-0.1608083	2.0123486	0.7684311	0.9555024
Pfkfb1	0.0829321	1.6296272	0.7684675	0.9555024
Adamtsl5	0.1421761	1.1014997	0.7685193	0.9555024
Rps3a3	0.1057282	1.4133719	0.7685937	0.9555024

Morn1	-0.1209537	1.1038564	0.768718	0.9555024
Peg12	0.1680018	-0.1820516	0.7687374	0.9555024
St3gal5	0.0505901	7.8523538	0.7687571	0.9555024
Bnip2	-0.0318642	7.0065559	0.7688532	0.9555024
Glyr1	-0.0362449	7.1457589	0.7688781	0.9555024
Vps53	-0.0445743	4.8190624	0.7689821	0.9555024
Gas7	0.0678086	5.8591028	0.7691326	0.9555024
Cdkn1a	-0.0514554	4.6538338	0.7691443	0.9555024
Gm20604	-0.1215158	0.1483031	0.769175	0.9555024
Mrpl30	0.0444342	5.6266094	0.7691755	0.9555024
Gm6210	-0.1700926	-0.5317387	0.7691813	0.9555024
Stk16	0.033121	6.1691721	0.7693116	0.955588
Ndufa4	0.0489998	7.0953867	0.7694131	0.9556377
Wtap	0.0306267	6.787098	0.7697319	0.955871
Sco1	0.0586914	3.3991125	0.7698083	0.955871
Dnm2	-0.0372645	7.3578829	0.769827	0.955871
Tsku	-0.0918592	3.7876798	0.7698686	0.955871
Slc35g2	-0.2310716	-0.9686834	0.7700558	0.955871
Polr1b	0.0484407	4.2796776	0.7701727	0.955871
Scyl1	0.0355518	5.6126631	0.7702049	0.955871
Cxcr4	0.0508655	8.0480412	0.770214	0.955871
Wwtr1	-0.0677873	4.1187527	0.7702392	0.955871
Dync1i1	0.278506	0.0531644	0.7703074	0.955871
Pmpca	0.0339986	6.2394698	0.7704009	0.955871
Gm4951	-0.0805919	2.771772	0.7704387	0.955871
Gm5819	0.1204212	2.3779394	0.7704982	0.955871
Gm7285	0.0874868	2.095923	0.7705216	0.955871
Zfp846	0.0583349	2.8045766	0.7705228	0.955871
Ggnbp1	0.1282706	0.0219059	0.7706017	0.9558823
5430405H02Rik	-0.0846413	1.227004	0.7707087	0.9558823
Col4a1	-0.0546521	6.8038335	0.7707162	0.9558823
Klhl5	-0.0337054	5.5335439	0.7708697	0.9559245
C1qtnf1	-0.0547029	4.4728066	0.7708964	0.9559245
Eogt	0.0604144	3.4574977	0.7709437	0.9559245
Pdzrn4	0.1408162	0.1331116	0.7709961	0.9559245
Pnpla2	-0.0450895	5.7411254	0.7710854	0.955959
Hic1	0.0814905	2.0661233	0.7711566	0.9559712
Hectd3	-0.0387612	5.3246648	0.7713237	0.956102
Khdc4	-0.0362597	6.459143	0.7713853	0.9561022
Zbtb2	-0.0465834	4.3594999	0.7715562	0.9561669
Ddb2	0.0593359	3.3307148	0.7715736	0.9561669
Arl14ep	0.0486987	4.6176579	0.7716797	0.9561669
F9	-0.2303077	-0.5982416	0.7717153	0.9561669

Zfp52	-0.0433564	4.0594372	0.7718551	0.9561669
Gm32745	-0.1623664	0.0632386	0.7719239	0.9561669
Gmeb1	-0.0365091	5.1880244	0.7719699	0.9561669
0610010K14Rik	0.0387142	4.887301	0.7719869	0.9561669
Sgsh	-0.0413959	4.232041	0.7720467	0.9561669
Arhgap21	-0.0381163	6.2157165	0.7721333	0.9561669
Pard3	0.099724	2.94955	0.7721571	0.9561669
Arl6ip6	0.034503	5.7584964	0.7721751	0.9561669
Ctns	0.0425566	4.2167577	0.7722882	0.9562192
Tspan9	-0.0510999	4.2447657	0.7723403	0.9562192
Bcap29	0.0548198	5.2888737	0.7724139	0.9562342
Bicdl1	-0.112761	0.9870725	0.7726045	0.9563579
Gorab	0.0645095	2.6776992	0.7726817	0.9563579
Rps29	0.0435809	9.7288616	0.7726983	0.9563579
Ndfip1	-0.0311579	6.8076081	0.7728204	0.9563989
Parg	0.0312761	5.7761226	0.7728947	0.9563989
Snx33	0.0724221	3.3153635	0.7729623	0.9563989
3110040N11Rik	0.0607883	3.6679921	0.7730984	0.9563989
Rab40c	0.0515118	3.3858665	0.7731862	0.9563989
Gper1	0.2055723	-0.0693169	0.773286	0.9563989
Gm32184	0.1843621	-0.9334083	0.7733855	0.9563989
Col27a1	0.0839163	4.4364576	0.7734201	0.9563989
Cep20	0.0460894	5.3648828	0.7734811	0.9563989
Ldlrad4	-0.0567674	4.1293408	0.7734986	0.9563989
Trnt1	0.0370526	5.3861377	0.7735033	0.9563989
LOC118568781	-0.1137015	0.7821941	0.7735069	0.9563989
Hint3	0.0578586	3.3505612	0.7735308	0.9563989
Egfr	-0.0634332	3.1148845	0.7736496	0.9564698
Gm26799	-0.1150027	0.734809	0.7737222	0.9564835
Zfp46	-0.0449077	4.1229836	0.7738432	0.9564966
Gm17383	0.1126573	0.048538	0.7739237	0.9564966
Cox7a2	-0.0548106	6.6053222	0.7739742	0.9564966
Mrpl42	0.0404068	5.7522799	0.7739788	0.9564966
Disp1	0.0584224	2.9466688	0.7741677	0.956654
Capn6	-0.1609569	1.5979142	0.7743333	0.956689
Tgm1	-0.0712367	2.2650347	0.7743437	0.956689
Get3	0.0357894	5.4643005	0.7743805	0.956689
Gm36459	-0.1542578	0.0776379	0.7744922	0.9567509
Smim40	0.1959612	-0.6753985	0.7747061	0.9569041
Gm41538	0.1215276	1.2408576	0.7748446	0.9569041
Trim5	-0.1029045	3.2200108	0.774969	0.9569041
Rtf2	-0.03071	6.2767852	0.7750142	0.9569041
Cox16	-0.0494992	3.1079283	0.7750356	0.9569041

Zfp11	-0.0937247	1.5683223	0.7750587	0.9569041
Ror2	0.2120466	-0.4986003	0.7751063	0.9569041
Fastkd3	0.0591541	2.8721089	0.7751083	0.9569041
Btbd3	-0.0720234	3.4727531	0.7753631	0.9571427
2010320M18Rik	0.0972972	2.3478591	0.7756894	0.9573597
Gm31718	0.0803878	1.7614831	0.7756977	0.9573597
Ahcyl1	0.0315129	6.8070726	0.7757236	0.9573597
Hmox2	0.0364487	5.8053147	0.7758837	0.9574813
Kdelr2	-0.0352102	6.5139268	0.776085	0.957608
Wdr36	0.0438748	5.0772623	0.7761566	0.957608
Cnppd1	0.0322077	6.4699628	0.7762236	0.957608
Atxn2	-0.045515	5.633319	0.7762894	0.957608
Lrch3	-0.0372373	5.512175	0.7763905	0.957608
Prdm4	0.0407757	4.7505284	0.7763908	0.957608
Derl1	-0.0294485	6.9609035	0.7764407	0.957608
Rnf10	0.0479003	8.8015133	0.7764788	0.957608
Jak3	0.0470642	4.8492582	0.7765455	0.9576142
Rpl31-ps8	0.061287	3.9486309	0.7766561	0.9576462
Gnptg	-0.0454167	3.9283326	0.7767126	0.9576462
Inka1	-0.0502672	4.1957714	0.7767961	0.9576462
Dvl3	0.0428552	5.1880688	0.776874	0.9576462
Gm9294	0.1250636	-0.368298	0.7768792	0.9576462
Dennd4a	-0.0473139	8.3475422	0.7770725	0.9577656
Ap2b1	-0.0364391	6.8571838	0.777125	0.9577656
Srp54c	-0.0414986	4.2882699	0.7772775	0.9577656
Tbc1d13	0.0332918	5.5940677	0.7773256	0.9577656
Clcn2	0.0900597	4.0071323	0.777377	0.9577656
Ift172	0.0583707	3.5704764	0.7774158	0.9577656
Tle3	-0.0419623	6.564404	0.777435	0.9577656
Kifc3	-0.0691246	3.1299523	0.7774737	0.9577656
Cenpf	-0.0453427	7.7688881	0.7776438	0.9577656
Reep1	-0.0931884	1.3512218	0.7777224	0.9577656
Rgs9	0.1502895	-0.033516	0.7778037	0.9577656
2010001A14Rik	0.101441	1.2956047	0.7778429	0.9577656
Cbx2	0.0756042	2.6522072	0.7780066	0.9577656
C1qb	-0.0457619	6.5874532	0.7780173	0.9577656
Pacsin1	-0.0935377	2.5475872	0.7781044	0.9577656
Mnat1	0.0589853	3.9339588	0.7782071	0.9577656
Ankrd24	-0.0817638	1.3126401	0.7783414	0.9577656
Cradd	-0.0595872	2.6261453	0.778482	0.9577656
Eps15	-0.0353471	7.7634697	0.7786344	0.9577656
Sacs	0.0445704	5.3833251	0.778651	0.9577656
Ythdc2	-0.0473884	5.0384167	0.7786643	0.9577656

Col11a2	-0.098009	9.3832112	0.7786782	0.9577656
Akr1c19	-0.1489172	-0.0627321	0.7787636	0.9577656
Uggt2	-0.0989121	2.4377734	0.7787878	0.9577656
Pcdhgc5	-0.2213859	-0.5606784	0.7788376	0.9577656
Npr1	0.0550995	3.3526638	0.7788962	0.9577656
Spcs1	0.0355144	6.4910606	0.7790989	0.9577656
Gm12263	-0.1440887	-1.0371529	0.779159	0.9577656
Cox14	0.0484542	4.986212	0.7792366	0.9577656
Tm7sf3	-0.0392751	6.3274205	0.7792417	0.9577656
Lmbr1	0.0814784	2.0603394	0.7792543	0.9577656
Klk8	0.1124299	0.7386136	0.7793373	0.9577656
Plcl1	-0.0975892	1.1792458	0.7793587	0.9577656
Eif1a	0.0343642	5.7361566	0.7794078	0.9577656
Rpusd1	0.0598873	2.8296608	0.7794242	0.9577656
Zfpl1	0.0509762	3.6808905	0.7794325	0.9577656
Zbtb14	0.0343014	5.2656216	0.7794541	0.9577656
Asxl2	-0.0373351	6.4190826	0.7795121	0.9577656
Rhpn2	0.1463365	1.4519939	0.7795592	0.9577656
Gm34907	-0.208652	-0.5351739	0.7796395	0.9577656
Tead3	-0.0986735	1.2839208	0.7797532	0.9577656
Zfp874a	-0.0617432	3.9146239	0.7798724	0.9577656
Klf15	-0.0990083	0.9031111	0.7798763	0.9577656
Tmem177	0.0666425	2.5457021	0.7799768	0.9577656
Depdc7	-0.0882227	1.7076262	0.780021	0.9577656
Ogfod1	-0.0433023	4.194776	0.780033	0.9577656
Mras	-0.0787179	3.3163031	0.7800669	0.9577656
Yipf2	-0.0453901	3.6273527	0.7804238	0.9577656
Ifrd1	0.0397877	5.6344742	0.7804542	0.9577656
Wdcp	-0.0595701	2.674628	0.780522	0.9577656
Mtfr2	-0.0387908	4.8365535	0.7805401	0.9577656
Rhof	-0.0437444	5.7494188	0.7805983	0.9577656
Fam98c	0.0516995	3.2771084	0.7806697	0.9577656
Stbd1	0.1186258	0.3387066	0.7806893	0.9577656
1700047M11Rik	-0.0832746	2.4111746	0.7806932	0.9577656
Cdk5rap2	0.037426	5.5545239	0.7806981	0.9577656
Hdac6	-0.0482677	3.4637	0.780715	0.9577656
Acacb	-0.1177263	1.0881695	0.7807188	0.9577656
Pi4kb	0.0424534	4.8660136	0.7807904	0.9577656
Zfp87	0.0427778	4.150055	0.7808319	0.9577656
Akap10	-0.0324905	5.7480755	0.7808426	0.9577656
Tmem184b	-0.0435311	5.4659971	0.7808616	0.9577656
Cd83	-0.077337	3.53113	0.7808649	0.9577656
Cog4	-0.033212	5.3563305	0.7810067	0.9577656

Dscc1	0.0665116	3.8346503	0.7810758	0.9577656
Dock4	0.0576866	3.6335001	0.7810791	0.9577656
Klhdc4	-0.0539339	4.2552667	0.7811017	0.9577656
Surf2	0.0463895	3.8962514	0.7811945	0.9578038
Ip6k2	-0.0374493	4.6832783	0.7814277	0.9579687
Wdr26	0.0360672	8.8962375	0.7815133	0.9579687
Leprot	0.0363086	6.6327828	0.7815674	0.9579687
Rbp4	-0.1441303	2.5827266	0.7815922	0.9579687
Eme1	-0.0481289	4.0977838	0.7816623	0.9579687
Smpdl3b	0.2002751	-0.3407135	0.7817338	0.9579687
Med24	-0.0365441	5.5013284	0.7817934	0.9579687
Marchf9	-0.1384598	0.6815481	0.7819736	0.9579687
Tmem209	-0.0330278	5.1980386	0.7819871	0.9579687
Zfp935	-0.0638456	3.5711246	0.7819908	0.9579687
Gm8116	0.0879432	1.9661337	0.7820065	0.9579687
Xlr4b	-0.0689491	1.6317019	0.7821928	0.9580139
Isca2	0.0498882	5.3736811	0.7822893	0.9580139
Wdtc1	0.0334864	5.9175504	0.7823074	0.9580139
Shank3	-0.0557647	4.2206455	0.7823643	0.9580139
Heyl	-0.0933779	3.3677875	0.7823992	0.9580139
Eif3e	0.0397595	7.7211445	0.7825644	0.9580139
Nanp	-0.086258	1.5027438	0.7825802	0.9580139
Kif23	0.0381833	6.9429014	0.7825845	0.9580139
Ypel1	0.0966339	0.9278367	0.7826951	0.9580139
Gm52482	-0.1974449	-0.142496	0.78282	0.9580139
Glod4	0.0342409	5.3319333	0.7828385	0.9580139
8030462N17Rik	-0.0368362	5.5858314	0.7828592	0.9580139
Stat5a	0.0354661	5.3707963	0.7829049	0.9580139
Gm12346	-0.0714927	3.0755868	0.7829056	0.9580139
Arl2	-0.0480041	3.6012144	0.7830385	0.958068
Dnah8	0.0989077	4.8528879	0.7830731	0.958068
Pacs2	0.0461685	6.0963519	0.7833283	0.9582699
Zfp30	0.1002969	1.1560233	0.783385	0.9582699
Gm10182	-0.1015376	0.1353113	0.7834229	0.9582699
Lipo3	0.1093252	2.3193836	0.7836758	0.9584518
Gm5898	0.1158575	0.8625232	0.7838452	0.9584518
Ubr1	-0.0313221	6.1694614	0.7838558	0.9584518
1600014C10Rik	-0.066169	6.1034723	0.7839758	0.9584518
Kif3a	0.0466595	3.8319972	0.7841237	0.9584518
Mrps14	0.0357846	5.324963	0.7841517	0.9584518
Tm9sf4	-0.0354194	5.5758527	0.7841668	0.9584518
Gm8493	-0.1562844	-0.1576356	0.7842597	0.9584518
Cacybp	0.0365778	6.9774013	0.784271	0.9584518

LOC118567473	0.1388907	0.5234747	0.7842784	0.9584518
Usp32	-0.0474319	7.833642	0.7844033	0.9584518
Snx1	-0.0357263	6.8664965	0.7844163	0.9584518
Rpl17-ps7	0.1706299	-0.1739939	0.7846021	0.9584518
Cd36	-0.0430689	7.0399872	0.7846242	0.9584518
Lsm1	0.0449461	4.0055078	0.7846654	0.9584518
Zfp677	-0.0749321	2.9713868	0.7847026	0.9584518
St3gal1	0.0426922	7.0030001	0.784753	0.9584518
Lmcd1	0.0889945	2.0203052	0.7847575	0.9584518
Coro1c	0.0314651	6.9982332	0.7848793	0.9584518
Ap2a2	0.0340164	7.2037502	0.784899	0.9584518
Cyth2	0.0386701	5.2912931	0.7849141	0.9584518
1700029J07Rik	0.1505731	0.2768399	0.7849876	0.9584518
Ccpg1os	-0.0995242	0.9946022	0.7849889	0.9584518
Tgfbi	0.040164	7.1577573	0.7851263	0.9585328
Nme7	-0.0461009	3.3205798	0.7851785	0.9585328
Efcab2	0.0763328	2.5452573	0.7853552	0.9585998
Taf11	0.0349412	4.7582321	0.7853566	0.9585998
Kif18a	-0.0437944	5.7822263	0.7855011	0.9586362
Ypel5	-0.0380214	6.7333957	0.7855097	0.9586362
Jmjd4	0.062449	3.7200425	0.7856468	0.9586556
Hmgb1-ps3	-0.1796359	-0.968735	0.7856824	0.9586556
Rec114	0.064178	4.3633507	0.7857105	0.9586556
Cpsf4	-0.0351934	5.1943815	0.7858769	0.9587219
Crem	0.0577954	3.2429487	0.7859969	0.9587219
Suclg2	0.031938	5.306572	0.7860027	0.9587219
Gm52232	-0.1035219	0.1603146	0.7860329	0.9587219
Snap29	-0.030414	5.9037479	0.7860731	0.9587219
Tcea2	-0.0749276	0.9150078	0.7863008	0.9588772
Ube2cbp	-0.117781	0.6456021	0.7863237	0.9588772
Lsm3	0.0438503	5.9759666	0.7864217	0.9589072
Pdss2	0.0637772	2.4383521	0.7865271	0.9589072
Eif3c	-0.0367047	7.7474337	0.7865722	0.9589072
Pex14	0.0466679	3.848564	0.7865949	0.9589072
Zfp429	0.0594945	3.3226643	0.7867013	0.9589091
Cyp4v3	-0.052127	4.5473237	0.7867198	0.9589091
Gm12338	0.0924007	1.5677528	0.7869017	0.9589177
Twf1	-0.0269931	6.0585235	0.7869623	0.9589177
Gipc3	-0.1847282	-0.5980918	0.7869718	0.9589177
Mrpl14	0.0518185	4.3177729	0.7871574	0.9589177
Gm19426	-0.1183094	0.4599066	0.7871963	0.9589177
Socs5	0.0487819	4.327761	0.7872116	0.9589177
Ptk2b	-0.0357102	6.7095178	0.7872728	0.9589177

9130024F11Rik	-0.1041848	2.7370836	0.7874876	0.9589177
Chmp2b	0.0385746	4.9771694	0.7875432	0.9589177
Mettl8	0.0533652	3.747782	0.787544	0.9589177
Erbb3	-0.0827397	1.3831522	0.7875817	0.9589177
Septin7	-0.0303308	7.8978062	0.7876164	0.9589177
Fndc10	0.1309014	1.6598101	0.7876199	0.9589177
Herc2	-0.0360576	6.9090842	0.7876566	0.9589177
Mutyh	0.0882511	1.5244966	0.7877061	0.9589177
Fam122a	0.0418296	4.5881592	0.7877133	0.9589177
Slc25a5	-0.0359668	8.2547599	0.7878012	0.958931
Foxd2	-0.1405938	0.0988663	0.7878832	0.958931
Rasl11a	0.0985598	0.7799717	0.7879091	0.958931
Gdpd3	-0.0834322	1.9122153	0.787996	0.9589618
Commd3	0.0416529	5.772124	0.7881139	0.9590301
Wfdc3	0.1218883	0.3026295	0.7883072	0.9590937
Faf1	0.0321833	5.5688843	0.7884654	0.9590937
Zscan2	0.0730192	2.3305003	0.7885649	0.9590937
Fgd1	-0.1018144	2.1907367	0.7885676	0.9590937
Add1	-0.0396486	8.358039	0.7887188	0.9590937
Gas8	0.0799859	2.2450048	0.7887497	0.9590937
Gm42517	-0.0739636	1.9938199	0.7888446	0.9590937
Gprc5c	0.1030777	3.8442235	0.7888837	0.9590937
4632404H12Rik	-0.0737622	1.8371151	0.7890316	0.9590937
Gm15915	-0.0923631	1.278456	0.7890389	0.9590937
Commd6	-0.0411515	3.6965641	0.7891724	0.9590937
Bcl2l15	-0.0783241	1.7479707	0.7892181	0.9590937
Ttc7	-0.033146	6.7366601	0.789305	0.9590937
Tmem138	0.0572378	3.4769174	0.7893234	0.9590937
Usp48	0.0297033	6.5059897	0.7894676	0.9590937
Cdc42se1	-0.0331688	7.1274969	0.7895496	0.9590937
Zmynd19	0.0352196	5.0942385	0.7896881	0.9590937
Gm10814	0.3182726	-1.2471536	0.7897174	0.9590937
Lamtor5	-0.0391561	4.9676535	0.7897854	0.9590937
Ifi208	-0.0951979	1.8342776	0.7897928	0.9590937
Fbxw5	-0.0390977	4.7887792	0.7899442	0.9590937
LOC118567840	0.2059011	-0.8946048	0.7900786	0.9590937
Tmem204	0.0795482	2.7652819	0.7901205	0.9590937
Nostrin	-0.0965753	1.1690526	0.7901223	0.9590937
Npat	0.0383202	5.6665645	0.7901551	0.9590937
Pelp1	0.0403117	4.9858084	0.7901892	0.9590937
Poldip3	0.0341174	6.9287768	0.7901893	0.9590937
Tpk1	-0.0600942	2.9054724	0.7902341	0.9590937
Zfp605	0.0592502	2.8286661	0.7902576	0.9590937

Ubald1	-0.0411995	4.9740006	0.7902743	0.9590937
Ydjc	0.0682476	1.9347734	0.7902848	0.9590937
Tfcp2	-0.0542487	3.2577764	0.7902994	0.9590937
Gm41235	-0.1186394	1.4955746	0.7903427	0.9590937
Gm5113	-0.0698251	1.9837392	0.7905223	0.9590937
Tctn2	-0.1079454	0.6125015	0.7905344	0.9590937
Lyl1	0.0331667	6.742543	0.7905816	0.9590937
Akirin1	-0.0287295	6.3697936	0.7905909	0.9590937
Ndufa12-ps	0.0593354	2.5632352	0.7907133	0.9590937
Gm5446	0.1872297	-0.9486964	0.7907335	0.9590937
Ankrd29	-0.1123225	0.7773015	0.7908153	0.9590937
Coa4	-0.0628942	3.0485661	0.7909007	0.9590937
Cenpl	-0.0433195	5.4984577	0.7909766	0.9590937
Rhbdl3	-0.101921	1.5464071	0.791017	0.9590937
Naxd	0.0370196	4.4500747	0.7910871	0.9590937
Hmgxb3	-0.0447506	4.417762	0.7911076	0.9590937
Rab39b	0.1555639	-0.5708448	0.7912063	0.9590937
Slc48a1	0.0443014	7.281581	0.7912354	0.9590937
Snx24	0.1001395	1.3976579	0.7912435	0.9590937
Itgb7	-0.0380975	5.4984231	0.791382	0.9590937
Gm8399	-0.0710297	1.5223746	0.7914041	0.9590937
Marco	0.1738004	-0.1569224	0.7914703	0.9590937
Slf2	0.0386415	6.0972974	0.7914749	0.9590937
Piezo1	-0.0288293	6.3869568	0.7915558	0.9590937
Ankrd40	0.0390325	5.6886273	0.7915626	0.9590937
Znhit6	-0.0442618	3.8859912	0.7916669	0.9590937
Slc39a6	0.035659	4.3711412	0.7917172	0.9590937
Trpa1	0.1724093	-0.3423315	0.7917258	0.9590937
Haus7	0.0447785	3.9562238	0.7917639	0.9590937
Mrpl53	0.0474296	4.719076	0.7918042	0.9590937
Ctla2a	-0.0428321	6.4241036	0.7919775	0.9591302
Lrrc71	-0.149842	-0.6099992	0.7920456	0.9591302
Pmvk	0.043791	3.8806922	0.7921035	0.9591302
Hnrnpf	0.0349892	8.7111691	0.7921299	0.9591302
Ubxn2b	0.0472654	4.9200977	0.7921427	0.9591302
Foxs1	-0.1513636	-0.4371139	0.7923273	0.9592367
Bbs2	-0.0591646	2.4229977	0.792354	0.9592367
Cblb	0.044047	5.4636418	0.792445	0.9592723
Ifi211	-0.0604287	3.2766099	0.7929223	0.9594409
Bcl11b	-0.1210339	0.7645168	0.7929882	0.9594409
B9d1	0.1471263	0.247011	0.793001	0.9594409
Ocrl	-0.0329649	4.8836685	0.7930803	0.9594409
Adam22	0.0970035	0.6834241	0.7930965	0.9594409

Tubgcp5	-0.0364429	4.4995985	0.7931217	0.9594409
Mrpl36	-0.0369664	4.6150933	0.7932062	0.9594409
Tex10	0.0419948	4.9674945	0.7932793	0.9594409
Senp5	-0.0423279	5.2899408	0.7936057	0.9594409
Necap1	-0.047535	4.4011896	0.793743	0.9594409
Eci1	0.0409815	4.5392277	0.793781	0.9594409
Trappc3	-0.0342927	5.0959538	0.7937906	0.9594409
Fyn	-0.03171	5.9663145	0.7938615	0.9594409
Smyd4	0.0635925	2.281762	0.7939055	0.9594409
Rasl12	0.1860281	-0.4727123	0.7939647	0.9594409
Mmp27	0.156175	-0.5432672	0.7939652	0.9594409
Ankrd52	-0.047393	6.1843436	0.7939741	0.9594409
Mrps15	-0.0490002	5.4149171	0.7940438	0.9594409
Mgll	0.0353737	6.6237658	0.7940604	0.9594409
Pgp	0.0349101	5.9737957	0.794067	0.9594409
Canx	-0.0323368	8.9705466	0.7940852	0.9594409
Bicral	-0.0302331	5.7980285	0.7941198	0.9594409
Ecscr	-0.0937311	1.7852809	0.794164	0.9594409
Cnot6	0.0300353	7.6544722	0.7941823	0.9594409
Lzts2	0.048686	3.9466596	0.794227	0.9594409
Grk2	-0.0392049	8.444977	0.7942982	0.9594409
Fkbp3	-0.0321516	5.9111983	0.7942983	0.9594409
LOC118567342	-0.0950471	1.1000681	0.7943114	0.9594409
Mplkip	0.0378059	4.6360661	0.7944204	0.9594452
Lix1l	0.0632977	3.723324	0.7945586	0.9594452
1110008P14Rik	-0.0423481	4.145544	0.7945656	0.9594452
Ubxn2a	-0.0347758	6.3406193	0.7946231	0.9594452
Fbxo46	0.0465586	3.7947845	0.7947576	0.9594452
Gpatch3	0.0565569	2.7938062	0.794818	0.9594452
Gm46224	0.144582	-0.0459795	0.7948443	0.9594452
Acot13	-0.044342	4.4523443	0.7948792	0.9594452
Brca2	-0.0443588	5.7602548	0.7949296	0.9594452
Erc1	-0.0370276	4.6867025	0.7949976	0.9594452
Osbp	-0.0327666	6.4741185	0.7951753	0.9594452
Mtg1	-0.0571573	3.0049351	0.7951784	0.9594452
Fbxo33	-0.0343603	5.4822152	0.7952127	0.9594452
Maf1	-0.0288597	5.9747107	0.7952495	0.9594452
Ptpn9	-0.0343596	4.6004244	0.7953011	0.9594452
Ccdc115	0.0368097	5.1971594	0.7953468	0.9594452
Spg20	0.0445549	4.3646169	0.7954873	0.9594452
Nanos3	-0.1664922	-1.2540816	0.7955364	0.9594452
Spire2	-0.1148496	0.1003519	0.7955413	0.9594452
Rgs10	0.0309628	6.0254547	0.7956092	0.9594452

Coil	-0.0520409	3.1351484	0.7956565	0.9594452
Pnisr	0.0410918	7.4761988	0.7956721	0.9594452
Zfp521	-0.0627678	2.7910604	0.7958913	0.9596351
5430425K12Rik	0.2127269	-0.8229573	0.795956	0.9596388
Cfap410	0.0709728	2.291179	0.7961769	0.9597481
Gm35486	-0.0701154	1.8545144	0.7961842	0.9597481
Lrch2	-0.1324544	1.0038136	0.7962318	0.9597481
Cop1	-0.0272501	6.8455864	0.796408	0.9598861
Tusc1	0.0470772	4.6820998	0.7966101	0.9599911
Fermt2	0.0618133	4.4951385	0.7966186	0.9599911
Xk	-0.040127	6.3124893	0.7968283	0.9601197
Hyou1	-0.0352074	6.5025758	0.7968487	0.9601197
Nedd1	-0.0343509	5.3010241	0.7969377	0.9601525
Rgs9bp	0.0911823	0.4463268	0.7970506	0.9601612
Gm34865	0.1359867	-0.268614	0.7970684	0.9601612
Sft2d3	-0.0522364	3.039428	0.7972895	0.9603532
Ppp2r2d	-0.0309243	5.3318641	0.7974138	0.9604057
5430431A17Rik	0.2729499	-0.063577	0.7974566	0.9604057
Acot8	0.0448872	3.8735913	0.7976481	0.9605462
Ccdc141	-0.1162087	0.6366604	0.7976968	0.9605462
Elovl1	-0.0273036	6.0882275	0.7978616	0.9606703
Akr1c13	-0.0624336	2.4030059	0.7979244	0.9606716
Ccdc96	0.0938143	-0.3698261	0.7981725	0.9608179
Ghitm	0.0446934	8.1629265	0.7982285	0.9608179
Cd248	-0.1438915	0.1398525	0.7982953	0.9608179
Eps15l1	0.0313749	6.1571288	0.7983366	0.9608179
Acaa1b	-0.2099584	-0.1618225	0.7984088	0.9608179
Gm6169	0.1661323	-0.6320668	0.7984166	0.9608179
Col6a1	-0.064117	6.8642702	0.7985434	0.9608817
Bbip1	-0.0355145	5.1678208	0.7985932	0.9608817
Ap1m2	-0.2124428	-1.0216647	0.7988077	0.9609753
Tnfrsf8	0.1249723	-0.6282177	0.7988509	0.9609753
Ncoa2	-0.0438455	6.3474716	0.7989042	0.9609753
Sirt7	-0.0302325	6.0515447	0.798918	0.9609753
Cdca2	0.034935	5.8887568	0.7990812	0.9610972
Map3k13	-0.1308497	-0.036874	0.799425	0.9613891
Pcdhb14	0.1510295	-0.2933481	0.7994475	0.9613891
Pcdhgb4	-0.1573996	-0.1186247	0.7995186	0.9614002
Hjurp	-0.0645209	7.4627258	0.7996474	0.9614236
Gm51891	0.092504	1.5008762	0.7997169	0.9614236
Gatb	0.0567833	2.9364616	0.7997235	0.9614236
Tmem181b-ps	-0.0482677	4.2544546	0.7999572	0.9616303
Eif4g3	0.0320689	7.2688232	0.8000814	0.9617053

Gm13842	-0.1626599	-1.0135994	0.8004946	0.9619391
Cdc25a	-0.0340606	6.0485219	0.8005913	0.9619391
Ubap1	0.0329257	5.9160314	0.8006169	0.9619391
Abcd1	-0.0399126	5.7292497	0.8006902	0.9619391
Cacna2d3	0.1200963	0.8684369	0.8007038	0.9619391
Rundc3b	-0.1561406	-0.6795843	0.8007617	0.9619391
Chd4	-0.0365853	8.574497	0.8008109	0.9619391
Anxa6	-0.0380509	6.366372	0.8008255	0.9619391
Map3k10	0.0454266	3.3808368	0.8008737	0.9619391
Rab5if	-0.0310752	6.8811329	0.8009679	0.9619391
Naa60	-0.0356172	6.2093727	0.8010175	0.9619391
Dusp18	-0.1166366	0.4049647	0.8010661	0.9619391
Ficd	0.0586029	2.5043084	0.8010799	0.9619391
Timm17b	-0.034119	4.620011	0.8015249	0.9622459
Spred3	-0.0955125	0.5699373	0.8016076	0.9622459
Nphp4	0.1379986	0.3716088	0.8016778	0.9622459
Shcbp1	0.0447201	5.5783097	0.8017163	0.9622459
Bud31	0.0419504	6.555849	0.8018613	0.9622459
Zbtb26	0.0464123	4.0276251	0.8019032	0.9622459
Camta2	-0.0346382	5.1807919	0.8019362	0.9622459
9330117O12Rik	-0.1628409	-0.5997518	0.8020326	0.9622459
Rps27rt	0.0473644	3.6625228	0.8020987	0.9622459
Ecd	0.0368927	5.2460743	0.8021035	0.9622459
Gpr137b	-0.0617521	4.8145564	0.8021093	0.9622459
Gm31583	0.1714755	-0.6819472	0.8021094	0.9622459
Mindy4	0.0988146	0.727733	0.8021596	0.9622459
Ptprv	-0.0372809	4.1875013	0.8022016	0.9622459
Dlx5	0.0874701	3.4423341	0.8023036	0.9622942
Dsn1	-0.03332	4.793812	0.8024593	0.9622956
Atp10a	-0.0523138	3.6968934	0.8025607	0.9622956
Mphosph9	-0.0381379	5.0543274	0.8025986	0.9622956
Dnajc24	-0.0502036	3.0135288	0.802608	0.9622956
Atp5j2	0.0470011	7.8166282	0.8026443	0.9622956
Rrad	0.1114252	0.9535526	0.8028132	0.9622956
Abhd17b	0.0356407	5.2785733	0.8028314	0.9622956
Gm41640	-0.0988491	0.6636272	0.8029372	0.9622956
Zfp422	0.0323946	5.6447009	0.8029811	0.9622956
Eef1akmt4	0.0756955	1.4639095	0.8030034	0.9622956
Fxyd1	0.0805661	4.3099419	0.8030152	0.9622956
Rpsa-ps2	0.1050222	0.6990387	0.8030472	0.9622956
Rpl21-ps8	0.1952249	0.1365293	0.8032227	0.9624318
Etfbkmt	0.0603232	2.5153919	0.8034544	0.9625159
Sms-ps	0.1421522	-0.2477324	0.803461	0.9625159

Zfp408	-0.038919	3.6579021	0.8035496	0.9625159
Ap3s1-ps1	-0.1418308	-0.7134881	0.8035575	0.9625159
Cep250	0.0328257	5.9968518	0.8036024	0.9625159
Pigs	-0.0281675	5.3486195	0.8037801	0.9625361
Hmgb1-ps4	0.1537422	2.1201841	0.803796	0.9625361
Cyp7b1	-0.1499877	-0.5761183	0.8038049	0.9625361
Syt12	0.1216528	0.6590096	0.8039013	0.9625774
Mtx3	0.042247	4.2520007	0.8040952	0.9627005
Rars	-0.0326076	6.1364016	0.8041279	0.9627005
Bub1b	0.038122	7.0761295	0.8041965	0.9627086
Slc46a1	0.0876595	1.3314086	0.8042673	0.9627121
Ptpn18	0.0494414	5.6973987	0.8043232	0.9627121
Nrip1	0.0317412	5.9260879	0.8046502	0.9628382
Cycs	-0.0343755	6.6149475	0.8046822	0.9628382
Eed	-0.028883	6.0698103	0.8047019	0.9628382
Rgl3	0.078848	1.8732038	0.8047734	0.9628382
Plekhf2	0.0283563	6.5508194	0.8048065	0.9628382
Vasn	-0.0807369	4.5297753	0.8048892	0.9628382
Tefm	-0.0631644	2.2827357	0.8049089	0.9628382
Tcaf2	-0.1219383	0.7484318	0.8051115	0.9628382
Gm15384	-0.1709764	-0.759273	0.805192	0.9628382
Naxe	0.0360587	4.5134465	0.8053601	0.9628382
LOC118567396	0.1263956	-0.053525	0.8053929	0.9628382
Radx	-0.1245443	-0.3480573	0.8054031	0.9628382
Clk4	-0.0406646	6.1639288	0.8054477	0.9628382
Ttc37	-0.0351461	4.7651825	0.8055046	0.9628382
Pdcd6	0.0301964	5.484574	0.8055672	0.9628382
BC055324	0.0359816	4.0461707	0.8055833	0.9628382
Zfp868	-0.0403747	4.0323503	0.8055957	0.9628382
Rps23-ps1	0.0418618	4.2690986	0.805715	0.9628382
Bcs1l	0.0553968	3.1271272	0.8058709	0.9628382
Clybl	0.0541237	2.9597693	0.8058731	0.9628382
Ap2m1-ps	-0.068985	2.3224106	0.8058852	0.9628382
Gm32031	-0.1234536	0.5243685	0.8059722	0.9628382
Rab10	0.028956	7.47213	0.8060339	0.9628382
Csrp1	-0.0374794	6.942166	0.8060463	0.9628382
Tab3	0.0370553	6.1071981	0.8060716	0.9628382
Klhl20	-0.0302807	5.0159333	0.8061245	0.9628382
Hs2st1	0.0350149	4.8735808	0.8061719	0.9628382
Pcdhb22	-0.1142718	0.0940657	0.8062875	0.9628382
Cul3	-0.028965	7.596781	0.8062882	0.9628382
Anapc11	0.0359583	5.0422418	0.8063101	0.9628382
Gramd1a	0.0408369	6.166171	0.8063804	0.9628382

Sltm	0.0269329	6.4117192	0.8064095	0.9628382
Zgrf1	-0.0477174	5.0336154	0.8066154	0.9629437
Nlrx1	0.0386977	5.064213	0.8067412	0.9629437
Ttc1	0.0299642	5.2048911	0.8068775	0.9629437
LOC115488500	0.149573	-0.125046	0.806934	0.9629437
Cog3	0.0284959	5.2623142	0.8070288	0.9629437
Platr7	-0.1013059	0.2118104	0.8070523	0.9629437
Zbtb49	-0.0748437	2.4916886	0.8070581	0.9629437
Serping1	0.0686459	7.9234249	0.8070996	0.9629437
Hbq1a	0.1268696	1.360119	0.8071006	0.9629437
Mphosph6	-0.0392104	4.5414769	0.8071486	0.9629437
H3f3a	0.0338661	9.9390875	0.8072942	0.9629437
LOC118567916	0.0890086	2.0703495	0.8073224	0.9629437
Mecr	0.0410987	3.609216	0.8073819	0.9629437
Zfp354a	0.1474498	0.1284787	0.807689	0.9629437
Fzd7	-0.0533836	3.2258698	0.8077772	0.9629437
Fetub	0.2395834	0.1474358	0.8078348	0.9629437
Slc35e4	-0.0738024	2.6423999	0.8079165	0.9629437
Mrc2	-0.0710488	5.6664394	0.8081647	0.9629437
Slc35c1	0.0612823	5.1807863	0.8082773	0.9629437
ND4L	0.0791351	1.0399823	0.8083724	0.9629437
Plekhm2	0.0345608	4.5559661	0.8083958	0.9629437
Lrpap1	-0.0266997	6.0118916	0.8084157	0.9629437
Larp6	0.1278861	1.1570317	0.8084397	0.9629437
Mettl15	-0.062097	2.4220133	0.8085032	0.9629437
Blzf1	0.0363546	4.4591711	0.8085878	0.9629437
Sh3glb2	0.0344671	4.7141115	0.8086145	0.9629437
Mtdh	-0.0282515	7.3296178	0.8086242	0.9629437
Fkbp9	0.0624902	6.4038359	0.8086556	0.9629437
Mdm4	-0.0295756	7.4153353	0.8086774	0.9629437
Zfp395	-0.0364311	4.1639164	0.8087798	0.9629437
Zranb3	0.0424994	4.5437023	0.80878	0.9629437
Rassf8	0.0506955	3.7391823	0.8088188	0.9629437
Smyd5	0.0521492	3.5167666	0.808918	0.9629437
Spcs2	0.0288592	7.1789967	0.8090227	0.9629437
Sec13	0.03198	6.0565986	0.8090633	0.9629437
Hoxa1	-0.1526416	-0.823533	0.8090708	0.9629437
Negr1	0.0732157	2.0799592	0.809099	0.9629437
Fbln5	0.0610815	6.0131146	0.8091017	0.9629437
Arf2	0.0334073	5.1984911	0.8091826	0.9629437
Sigirr	-0.0501996	2.5444711	0.8092461	0.9629437
4930481B07Rik	-0.070634	1.5209907	0.8092624	0.9629437
Ctnna1	-0.0252054	7.1328932	0.8092747	0.9629437

Ppfia4	0.1086157	0.7943056	0.8094211	0.9629437
LOC115489130	-0.0913293	0.8511474	0.8094311	0.9629437
Nupl1	0.0292362	6.5976353	0.8095033	0.9629437
Fam110b	-0.0550668	2.2049661	0.8095686	0.9629437
Mlkl	-0.0467577	4.1708704	0.8096055	0.9629437
Kazn	0.0795438	2.9499136	0.8096148	0.9629437
Ubl7	0.0301985	5.2161856	0.8096699	0.9629437
Lnx2	-0.0460773	4.0885894	0.8097077	0.9629437
Hoxb8	-0.1274009	0.2122065	0.8098245	0.9629437
Iffo2	-0.0589555	3.2815871	0.809963	0.9629437
Paics	0.0325786	7.6824683	0.8099973	0.9629437
B430319G15Rik	-0.1993698	-0.7343422	0.810017	0.9629437
Mkln1os	-0.1270317	-0.5869154	0.8100708	0.9629437
Xpo1	0.0300267	8.1791579	0.8100906	0.9629437
Zfp81	-0.038191	3.9944971	0.8100922	0.9629437
Pbrm1	-0.0305849	7.652914	0.810179	0.9629437
Rabgef1	-0.0327801	5.4455352	0.810188	0.9629437
5031425F14Rik	-0.1869877	-1.0846723	0.8103788	0.9629437
Gm4540	-0.1419155	0.0536304	0.8104002	0.9629437
Trmt6	0.0441311	4.7099017	0.8104801	0.9629437
Wdr13	-0.0359909	5.2891985	0.8105067	0.9629437
Fbxo34	0.0293733	5.5641342	0.8105635	0.9629437
Wwox	0.0597594	2.415142	0.8105814	0.9629437
Mcm8	-0.0443207	3.7016199	0.8105839	0.9629437
Sgce	0.0427011	3.522166	0.8108326	0.9631328
Hsdl2	0.0291056	5.5853981	0.8110319	0.9631328
Skida1	0.1785135	0.0944814	0.8111112	0.9631328
Map4	-0.0330626	6.3586132	0.811165	0.9631328
Arpin	-0.0765741	1.8220932	0.8112319	0.9631328
Tcf7l1	-0.0608615	3.1508698	0.8113154	0.9631328
Nmrk1	-0.0657644	2.1395122	0.8113581	0.9631328
Lamb1	-0.056794	4.6231495	0.8114628	0.9631328
Zfp143	-0.0293099	4.8648052	0.8114863	0.9631328
Irx6	0.2090763	-0.5685522	0.8115414	0.9631328
Kat2a	0.0354447	4.8137963	0.8116436	0.9631328
Zfp850	-0.0498602	2.8863265	0.8116749	0.9631328
B3galt6	0.0516855	3.1552054	0.8117424	0.9631328
Otulin	0.0387563	5.0736063	0.8118149	0.9631328
Mroh6	-0.0874672	1.6126892	0.8118188	0.9631328
Pigyl	0.0473797	4.2754142	0.8118616	0.9631328
Pip5k1c	0.0295932	6.2204036	0.8119161	0.9631328
Copa	-0.0282285	8.0029051	0.8119208	0.9631328
A2m	-0.1810585	-0.2451106	0.8119468	0.9631328

Gfpt2	0.1664549	-0.4005225	0.8120399	0.9631328
Gna14	0.1034877	0.835187	0.8121036	0.9631328
Lrba	-0.0339876	5.4950586	0.8122151	0.9631328
Gm16283	0.239396	-1.2085685	0.8122793	0.9631328
Gm11703	0.1255977	-0.7648866	0.8123968	0.9631328
Cetn3	-0.0387561	6.4826972	0.8124033	0.9631328
Zcchc14	0.0503567	4.8081852	0.8124807	0.9631328
St6galnac2	-0.0603479	2.0547651	0.8126326	0.9631328
Map3k7	0.0277875	6.4904879	0.8126748	0.9631328
Rbm39	0.0292653	8.8460319	0.8127227	0.9631328
Pgap6	0.0399642	5.2328702	0.812735	0.9631328
Mir22hg	0.0605435	3.6650998	0.8127441	0.9631328
Xlr3b	0.2016763	-0.1940369	0.8127986	0.9631328
Zfp13	0.1194331	0.0609426	0.8130581	0.9631328
Ccdc14	0.0591698	3.3078438	0.8130594	0.9631328
Pkn2	-0.0319499	7.0598534	0.813067	0.9631328
Soat2	0.0881353	0.9684902	0.8131106	0.9631328
Lrrc49	0.061421	2.0966159	0.8131573	0.9631328
Slc37a2	0.0611879	5.7040915	0.8131802	0.9631328
Ano1	0.0949681	4.3506993	0.813183	0.9631328
Nectin3	0.0941723	3.1648711	0.8133206	0.9631328
Cmklr1	0.0565676	2.2694531	0.8133341	0.9631328
Igkv4-50	0.1526937	1.7294487	0.8133805	0.9631328
Mob4	-0.0290587	5.4815457	0.8134057	0.9631328
Ccdc171	0.0890274	1.6622101	0.813639	0.963283
Gm35254	-0.1496543	-0.1733549	0.8137014	0.963283
Ap1m1	-0.0292356	6.0691513	0.8137215	0.963283
LOC118567330	0.2186335	-0.5739342	0.8138002	0.963283
Unc45a	-0.0381348	4.2656952	0.8138422	0.963283
Nup214	-0.0326141	5.8619992	0.8139597	0.9633488
Sap18	0.0252045	6.6877836	0.8141112	0.9634547
Mok	-0.1321813	-0.9475489	0.8143491	0.9635401
Gm15452	-0.1259037	-0.1674297	0.8143653	0.9635401
Gm36496	0.1481019	0.1164275	0.8143692	0.9635401
Polk	-0.0376287	3.6049417	0.814457	0.9635707
D930048N14Rik	0.0981503	0.1910944	0.8146533	0.9636597
Tifab	0.046171	6.1724956	0.8146814	0.9636597
Ston1	0.0662361	3.5133709	0.8147505	0.9636597
Ephb4	0.0442314	3.8992447	0.8148065	0.9636597
Top1	-0.0283572	8.2095371	0.814842	0.9636597
Fam8a1	-0.0351936	5.431022	0.815022	0.9637993
Mast2	0.0300518	6.1833461	0.8152348	0.9639776
Ly9	-0.0331618	4.5763771	0.8154354	0.9640741

Zbtb22	0.0331463	5.0316192	0.8154404	0.9640741
Ftsj1	0.0432631	4.7836538	0.8157177	0.9642225
Pkn3	0.0704056	2.3098606	0.8158638	0.9642225
Klhl15	-0.0457985	3.0364788	0.8158725	0.9642225
Arhgef10l	-0.0381669	3.7938266	0.8158885	0.9642225
ND6	-0.0482647	6.7775167	0.8158935	0.9642225
Phf11b	0.0916505	1.8524941	0.8159378	0.9642225
Ppp1ccb	0.0617085	2.4852157	0.8161081	0.9643505
Camk2n1	0.0881577	2.3035715	0.8162644	0.964462
Mroh1	0.0325941	4.7100692	0.8163334	0.9644701
Mmp17	0.0886159	0.7047627	0.8163976	0.9644728
Lin7c	-0.0254453	6.5087649	0.8165756	0.9645817
Bdh2	-0.1140752	1.2535057	0.8166138	0.9645817
Lsm11	-0.0445959	3.4780551	0.8166798	0.9645864
Gbx2	-0.1022429	0.4561755	0.8167985	0.9646533
Ap5z1	0.042508	3.7199628	0.8169986	0.9647248
Intu	-0.063843	2.6135184	0.8170133	0.9647248
Galnt10	0.0341088	5.8986865	0.8170451	0.9647248
Ppia	-0.0300987	9.7259765	0.8171714	0.9647567
Il11ra1	0.0563441	3.1542912	0.8171962	0.9647567
Scarb1	-0.0365763	5.9093975	0.8173924	0.9648235
Zfp958	-0.0439046	3.8454968	0.817402	0.9648235
Rpap1	0.0374341	4.1947822	0.8175303	0.9648235
Mprip	0.0275716	7.1709399	0.8175594	0.9648235
Taf6	0.0339968	4.9529438	0.8175629	0.9648235
ATP6	0.0492809	4.8089889	0.8176973	0.9648599
Rpl10	0.0293465	9.1633698	0.8177178	0.9648599
Papss1	-0.0329216	4.9501589	0.8181482	0.9651714
Sin3b	-0.0260758	5.9905098	0.8181955	0.9651714
Mpnd	-0.0416478	4.6788297	0.8182176	0.9651714
Fbxo4	0.0575959	2.8520131	0.8182301	0.9651714
Plcxd2	0.0622374	1.9004958	0.818327	0.9652126
Etf1	-0.0279002	7.6176863	0.8186086	0.9654715
Fktn	0.0353625	3.79497	0.8187278	0.9654922
Ptpn23	-0.0360739	4.4019824	0.8187851	0.9654922
Got1	-0.0356115	4.6412368	0.8188787	0.9654922
Polr2a	0.0389271	7.9663391	0.8189714	0.9654922
Gm5822	-0.1433934	-0.668154	0.8190048	0.9654922
Gm10910	0.1153699	0.036352	0.8190255	0.9654922
Tmem159	0.0604635	2.7620845	0.819239	0.9654922
Tbrg1	0.0288692	5.599402	0.8192528	0.9654922
Lrig3	-0.1077437	1.3750109	0.8192604	0.9654922
Ltv1	0.0290973	5.1858526	0.8192873	0.9654922

Psenen	-0.0270359	5.2561879	0.8193092	0.9654922
5730455P16Rik	-0.0392539	4.1925179	0.819371	0.9654922
Mtf2	0.0241398	6.3970139	0.8194591	0.965502
Pxk	-0.0311145	5.7969355	0.8195035	0.965502
Rasa4	0.0384138	4.5741168	0.8197802	0.9657127
Esyt1	0.0472113	7.0403876	0.8198413	0.9657127
Dpp9	-0.0362312	5.5425406	0.8198686	0.9657127
Gstcd	0.0394313	4.3060612	0.8199311	0.9657132
Gm35558	0.1002253	0.1922457	0.8200795	0.9657267
Hspd1	0.030592	7.9807892	0.8200934	0.9657267
Mad1l1	0.0374297	5.2160417	0.8202486	0.9657267
Fxyd2	-0.1192232	0.9068636	0.820366	0.9657267
Hspe1-rs1	-0.0526753	2.39918	0.8204271	0.9657267
Gm10131	0.1328784	-0.648941	0.8204542	0.9657267
Frk	0.0971716	2.2456033	0.8204958	0.9657267
Iffo1	0.0404875	4.6317319	0.820501	0.9657267
Rrnad1	0.0426856	4.128076	0.8205052	0.9657267
Slc35b4	0.035569	4.5652482	0.8206094	0.9657267
Zfp583	0.0809773	1.1774252	0.8206256	0.9657267
Hoxc6	0.076377	1.5833019	0.820739	0.9657871
Igdcc4	0.1136035	2.1361585	0.8209094	0.9659098
Pgam1-ps2	-0.092808	0.2532458	0.8212532	0.9659098
Cfap126	0.0962573	1.0150232	0.8212737	0.9659098
Gm2225	-0.0955805	0.1813453	0.8213076	0.9659098
Chchd3	0.0322566	6.3323481	0.8214042	0.9659098
Gm17202	-0.1763589	-0.5043378	0.8214234	0.9659098
Dnase1l1	0.0357774	4.3482499	0.8214564	0.9659098
Gls	-0.0245308	6.8497491	0.8215256	0.9659098
Lbh	0.0388714	6.574267	0.8215886	0.9659098
Hoxc9	-0.1006083	1.1033894	0.8215931	0.9659098
Kifbp	0.0273859	4.8438828	0.8216071	0.9659098
Lmo7	-0.0808338	4.2837801	0.8216268	0.9659098
Ctsa	0.0290852	7.3656503	0.8216505	0.9659098
Rad52	0.0363972	3.8567742	0.8217806	0.9659538
Tspan13	0.0259082	6.1314811	0.8218568	0.9659538
Ywhaz	-0.0288063	8.7906158	0.8220015	0.9659538
Mtfr1l	0.0285023	5.3201148	0.8221016	0.9659538
Pyroxd1	-0.0464933	3.3153119	0.8222032	0.9659538
Acat2	-0.0374376	4.2615881	0.8222354	0.9659538
Pheta1	-0.0679553	3.4796044	0.8222716	0.9659538
Mgat4a	0.0333671	4.725416	0.82234	0.9659538
Nup153	-0.0317283	7.1987454	0.8223868	0.9659538
Tmem144	-0.1022926	0.0715153	0.8223905	0.9659538

Golt1b	0.0411283	5.3757672	0.8224295	0.9659538
LOC118567721	-0.0551994	2.1634908	0.8225251	0.9659538
Tti1	-0.0266517	5.2341765	0.822545	0.9659538
Yy1	-0.0258756	7.4082344	0.8226211	0.9659538
Arfgef2	-0.0284776	6.3110143	0.8226244	0.9659538
Tmbim6	-0.0276009	8.9295564	0.8227246	0.9659538
Clstn2	-0.13498	-0.8524663	0.8228303	0.9659538
Nploc4	0.0338466	6.3038907	0.8228311	0.9659538
C1qc	0.0404828	6.7377309	0.8228693	0.9659538
Tollip	-0.0245467	6.3925704	0.82306	0.9659538
Wdr3	0.0276196	5.4653272	0.8231156	0.9659538
Pias1	-0.0239615	6.112753	0.8232023	0.9659538
Ranbp3l	0.1221565	0.8324263	0.8232373	0.9659538
Zfp687	-0.0306242	5.4253174	0.8233264	0.9659538
Zfp952	0.0407858	3.460661	0.8233276	0.9659538
Nuf2	0.0353552	6.766974	0.8233516	0.9659538
Ddx52	-0.0288743	5.5402962	0.8234483	0.9659538
Ctnnal1	-0.0376389	3.6050707	0.8234626	0.9659538
Lypd1	-0.1707689	-1.3631579	0.823489	0.9659538
Nfatc2	-0.0454074	2.7652452	0.8235616	0.9659662
Gm10778	0.1071407	-0.7036913	0.8238983	0.9662724
Chrna1	0.2326525	-0.7643936	0.8239469	0.9662724
Cacul1	0.0316267	5.6089072	0.8241136	0.9663545
Tmem132a	0.0779434	2.3720991	0.8241412	0.9663545
Ceacam16	0.108382	0.3957737	0.8243356	0.9664372
Oser1	-0.0303782	4.2723942	0.824336	0.9664372
Stk24	-0.0267405	7.6158708	0.8244987	0.9664858
Antkmt	0.0329566	4.7980038	0.8245367	0.9664858
Jrkl	-0.0404595	4.2709971	0.8246488	0.9664858
Gnrh1	0.170072	-0.0682936	0.8246636	0.9664858
Ccnc	0.0324147	5.1468083	0.8247439	0.9664858
Xkrx	-0.1012796	0.8815222	0.8247503	0.9664858
5031439G07Rik	-0.0323372	6.4531464	0.8250478	0.9667616
Rnf180	-0.05345	1.924828	0.8251162	0.9667689
Blm	0.0438078	6.4428248	0.825281	0.9668193
Gm8290	0.133509	-0.8416233	0.8252835	0.9668193
Togaram1	-0.0317416	5.0987858	0.8253587	0.9668346
Rnf217	-0.0675261	1.6274666	0.82548	0.9668843
Clec2g	-0.1277764	-0.129677	0.8255306	0.9668843
Nrd1	-0.0272513	7.3893828	0.8255877	0.9668843
P2rx7	-0.0449358	3.7574667	0.8257806	0.9670375
Snrnp27	-0.0317068	5.7129745	0.8260349	0.9671711
Sdhb	0.026867	6.1153944	0.8261164	0.9671711

Tmem39a	0.0333909	4.5593275	0.8261345	0.9671711
Slc41a1	-0.0464973	3.7670518	0.8261899	0.9671711
Ift122	-0.0568763	2.3663362	0.8262056	0.9671711
Tvp23a	0.1725559	-0.8216141	0.8264317	0.9673629
Clec2i	0.0528889	2.6519245	0.826606	0.9674942
Mpv17l	-0.0736325	1.5864613	0.8267108	0.9675441
Sirt1	-0.0265777	5.7745122	0.8268798	0.967669
Myl6b	0.0983998	0.4994131	0.8270541	0.9678001
Dhx40	0.0314251	5.5845516	0.8272427	0.9679331
Armh3	0.0413258	3.6639538	0.8273406	0.9679331
Suds3	0.0262529	6.3729186	0.8273544	0.9679331
Zfp709	0.0479296	2.9324668	0.8277431	0.968315
Zfp646	-0.0295184	5.6154192	0.8279202	0.9683496
Abcd4	0.0748165	1.8550961	0.8279932	0.9683496
Calr	-0.0294449	9.5057202	0.8280108	0.9683496
Bhlha15	0.0850605	1.6919505	0.8281192	0.9683496
Timm22	0.0302392	4.2403051	0.828173	0.9683496
ND1	0.0432917	11.921781	0.8282223	0.9683496
Zfp27	0.050442	2.5875218	0.828243	0.9683496
4933421O10Rik	-0.0635535	2.6399256	0.8283035	0.9683496
Prrg1	-0.0680051	2.3210717	0.8284073	0.9683496
Zfp507	0.0339914	4.4713395	0.8284281	0.9683496
Hilpda	-0.061058	2.1370944	0.8284575	0.9683496
Gm12474	-0.1201986	0.1898183	0.8287303	0.9685956
1700066M21Rik	0.0386098	4.4733238	0.8289281	0.9687541
B230307C23Rik	-0.0391801	3.6615325	0.8290225	0.9687916
Gpkow	0.0248767	5.4879373	0.8291467	0.9688421
Clec1a	0.1052569	0.7920901	0.8291903	0.9688421
Scpep1	0.0287682	5.6861012	0.8294002	0.9690146
Pcyt1a	-0.0348128	7.1117268	0.829593	0.969167
1700067K01Rik	0.1515422	-0.7313409	0.8296633	0.9691764
Aldh1b1	0.0644326	2.840172	0.8298961	0.9692045
Cd46	-0.0928049	1.9846078	0.8299117	0.9692045
Cass4	-0.0460159	3.3177903	0.8299612	0.9692045
Fkbp15	-0.0225455	6.0229816	0.8299739	0.9692045
Susd1	-0.0319019	6.1252278	0.829999	0.9692045
Zmat5	-0.0477837	3.4316959	0.8300793	0.9692256
Npr3	-0.1262424	0.7312026	0.8304531	0.9695197
Prss46	-0.146385	-0.407453	0.8304941	0.9695197
Anapc2	-0.0236155	6.050856	0.8305182	0.9695197
Mettl25	-0.047225	2.6419339	0.8306817	0.9695317
Ssh1	-0.0366766	5.3031391	0.8307602	0.9695317
Stip1	0.0289465	7.1711004	0.830797	0.9695317

Ube2ql1	-0.1125829	-0.3319607	0.8308078	0.9695317
Pcmt1	0.0251215	6.5737864	0.8308401	0.9695317
Dnajc19	0.0451559	5.4582971	0.83126	0.9699488
Ccdc174	-0.031583	4.44732	0.8313694	0.9700038
Cramp1l	0.0384688	5.536313	0.8315426	0.9701071
Grtp1	0.065042	2.0183287	0.8316149	0.9701071
Sh3bp1	-0.0273404	5.5495526	0.8316783	0.9701071
Gm6457	-0.1290036	-0.3272782	0.8317075	0.9701071
Plscr4	0.0408312	3.134573	0.8318411	0.9701902
Cdc42ep4	-0.0266253	5.2456758	0.8320993	0.9702862
Cfap36	-0.0375301	4.3410053	0.8321602	0.9702862
Arhgef1	-0.0258057	7.7177063	0.8321661	0.9702862
Pla2g2d	-0.0792029	2.359386	0.832173	0.9702862
Nek1	-0.0290477	4.727031	0.8323978	0.9703658
Cntnap1	-0.0726277	1.508294	0.8324005	0.9703658
Bpnt1	0.0406686	3.4450943	0.8324535	0.9703658
Ezh1	-0.0255341	5.3287358	0.8325797	0.9703658
Rpl15-ps2	-0.1142302	-1.0969555	0.832602	0.9703658
Vps37d	-0.0974419	-0.5431202	0.8326738	0.9703658
Rad50	0.0312839	5.9822889	0.8327621	0.9703658
Klhl7	-0.0658586	4.3165193	0.8328611	0.9703658
Lynx1	0.0901624	2.2480067	0.8328679	0.9703658
Gm51536	-0.1238251	0.018216	0.8329333	0.9703658
Adhfe1	-0.1034008	0.2048172	0.8329652	0.9703658
Extl1	-0.1370761	-0.7403966	0.8330261	0.9703658
Gpatch8	-0.0289778	6.1427567	0.8332844	0.9703658
Actr10	0.0277971	5.7701889	0.8332979	0.9703658
Gm52251	0.2064725	-0.9164198	0.8333347	0.9703658
Strada	0.0308474	4.229407	0.8334181	0.9703658
Gtf2e2	-0.0256076	5.8773781	0.8335935	0.9703658
Supt4b	-0.121659	-0.748098	0.8336797	0.9703658
Umps	0.0280178	5.7895147	0.8337898	0.9703658
Samd4b	0.0346662	5.2031034	0.8339134	0.9703658
Ms4a4c	-0.0443417	4.4024033	0.8339731	0.9703658
Auh	-0.0278475	4.7068938	0.8341498	0.9703658
ND2	0.0383245	11.169444	0.834163	0.9703658
Zfp58	0.0537598	2.9913106	0.8342915	0.9703658
Stt3b	0.0237941	7.6855284	0.8343884	0.9703658
Gm16083	-0.1035429	-0.4630277	0.8344643	0.9703658
2900093K20Rik	-0.0886686	0.3146544	0.834492	0.9703658
Igfbp6	0.1069051	0.232267	0.8344973	0.9703658
Bcar1	0.0479588	3.3419968	0.8345007	0.9703658
Gm2788	-0.0796848	0.7080431	0.8345485	0.9703658

Vill	-0.2770425	3.4418657	0.8346374	0.9703658
Lysmd2	0.085177	1.127872	0.8346506	0.9703658
Metap1	-0.0242762	5.5698976	0.834658	0.9703658
Cltc	-0.0245385	9.186598	0.83473	0.9703658
Ttll5	0.0362082	4.0848137	0.8347866	0.9703658
Tiprl	0.0262067	5.4326404	0.8348071	0.9703658
Mmgt2	0.0378196	3.3426402	0.8348131	0.9703658
Trpv4	-0.0953744	2.305401	0.834873	0.9703658
Epha4	0.0931777	0.3053449	0.8349321	0.9703658
Gbp7	-0.0352823	5.0267399	0.8349424	0.9703658
Trim14	0.0377065	4.1666985	0.8349756	0.9703658
Mastl	-0.0428627	4.4264477	0.8349905	0.9703658
Klrb1a	0.1048013	-0.7730286	0.835015	0.9703658
Fdft1	0.048777	4.8050613	0.8350741	0.9703658
Camk2a	-0.0522512	2.3688063	0.8351109	0.9703658
Nubpl	-0.0658204	1.5483471	0.8351649	0.9703658
Ttc21b	-0.053165	2.8264069	0.8352118	0.9703658
Pygo1	-0.0887676	1.8927788	0.8352358	0.9703658
Acsf2	-0.0392709	3.9727936	0.8353052	0.970374
Abhd17c	0.0311074	4.6341274	0.8355059	0.9705346
Irak1	-0.0223872	6.2705108	0.8358094	0.9708147
Usp20	-0.0407054	3.0459078	0.8361236	0.9710463
Wrn	-0.0284471	6.859967	0.8361337	0.9710463
Slc25a15	0.0452127	2.9901752	0.8362338	0.9710901
Jarid2	-0.0286749	6.3597167	0.8363371	0.9711044
Dbr1	0.0295625	4.5814463	0.836371	0.9711044
Gm14303	-0.0467106	3.3812332	0.8365347	0.9712219
Rbm27	-0.0231644	6.3833336	0.8366111	0.9712382
Zfp740	-0.0217502	6.5756889	0.836901	0.9715023
Fam83d	0.0464417	3.7280812	0.837111	0.9715464
Nfkb1	0.0330113	6.7544321	0.837137	0.9715464
Aif1l	0.2278435	-0.9300973	0.837144	0.9715464
Gpbp1l1	-0.0219801	6.5604762	0.8372238	0.9715464
Smg7	0.0260864	6.4172302	0.8372514	0.9715464
4833415N18Rik	-0.1033581	0.4383834	0.8375295	0.9716372
Tmem106b	-0.0242694	6.0628061	0.8375402	0.9716372
Adrb3	-0.1140856	-0.4032998	0.8376063	0.9716372
Ice2	0.0320641	4.184906	0.8376351	0.9716372
Sphkap	0.1391358	0.1491527	0.8378846	0.9716372
Ube2v2	0.0352487	4.5842296	0.8379462	0.9716372
Dync1i2	0.0225523	7.0373543	0.8379557	0.9716372
C1rb	-0.1080611	1.526533	0.8381218	0.9716372
Sec61a1	0.0231368	7.3064045	0.8382104	0.9716372

Efna1	0.0559669	2.6641549	0.838286	0.9716372
Jpx	0.078352	1.209687	0.8382942	0.9716372
Tada2b	-0.0277288	5.2546098	0.838303	0.9716372
Zfp820	-0.0668584	1.8091332	0.8383475	0.9716372
2310057M21Rik	0.0483785	3.1794989	0.8384243	0.9716372
Gm16580	0.0603226	1.6006402	0.8384356	0.9716372
Cul7	0.0421936	3.9998374	0.838448	0.9716372
Gm5532	0.1720351	0.2681747	0.8384987	0.9716372
1110059G10Rik	0.035461	4.672701	0.8385165	0.9716372
Ptms	0.0385735	6.3837257	0.8385166	0.9716372
Eml4	0.0290647	5.8461505	0.8386529	0.9717045
Ccdc6	-0.0259352	5.9535066	0.8386995	0.9717045
Mgat3	-0.1003167	0.7994733	0.8388418	0.9717969
Erich1	-0.0374841	3.0547643	0.8389614	0.9718631
Samd12	-0.1013623	0.8394008	0.8390715	0.9719182
Ubr5	-0.0237003	7.4128793	0.8392929	0.9719925
Ncoa5	-0.0262964	5.3713191	0.8393094	0.9719925
Ccdc51	0.0567666	2.8156893	0.8393775	0.9719925
Chpt1	0.0339103	4.6954394	0.8394144	0.9719925
Apool	0.038645	4.1337472	0.8394612	0.9719925
Scyl3	0.0256452	5.2492322	0.8395428	0.9719925
Dlg1	-0.0209605	6.2629788	0.8395802	0.9719925
Golga5	-0.0229348	5.3298246	0.8396355	0.9719925
Pms2	0.035865	4.7513347	0.839782	0.9720567
Fcgr1	-0.0372682	3.6769497	0.8398841	0.9720567
Vnn1	-0.1310238	-0.4679362	0.83991	0.9720567
Gm7816	-0.1007121	-0.2770795	0.839941	0.9720567
Dhx8	-0.0255064	5.5861439	0.8401615	0.9722352
Cdnf	0.1309064	-0.1011784	0.8402887	0.9722352
Metrnl	-0.0670993	4.8706714	0.8403925	0.9722352
Ttc26	-0.0988877	0.4834056	0.8404747	0.9722352
Fam131a	-0.0512397	2.4653589	0.8405157	0.9722352
Syp	-0.0593062	1.1605974	0.8405629	0.9722352
Socs7	0.0358933	4.8022502	0.8406718	0.9722352
Gtf2e1	-0.0295843	4.3870253	0.8407252	0.9722352
Klhdc1	-0.0553762	1.8218635	0.8407508	0.9722352
Gm45927	0.0654622	0.7459831	0.840759	0.9722352
Vstm4	-0.0465141	3.6796665	0.8408234	0.9722352
Slc23a3	0.1260312	-0.3310805	0.8408649	0.9722352
Ino80dos	-0.0636573	1.263062	0.8409078	0.9722352
Hexim1	0.0261589	6.2013318	0.8410269	0.9723006
Ubn1	-0.0229515	6.8386366	0.8412575	0.9724078
Gcsh	-0.0351613	3.028753	0.8413915	0.9724078

Gcc1	0.0264674	4.926496	0.8413951	0.9724078
Slc39a9	-0.0221786	5.5948114	0.8414787	0.9724078
Alg13	0.0352545	3.4284503	0.8415331	0.9724078
Slc25a17	0.0251713	5.4110644	0.8416555	0.9724078
Irs1	0.0635624	4.6073373	0.8417029	0.9724078
Zfp33b	-0.0384909	3.5803731	0.8417147	0.9724078
Dzip1	0.0615816	2.3246226	0.8418683	0.9724078
Cfl1	0.0243225	10.053163	0.8418732	0.9724078
Gm41829	0.0790526	0.6335806	0.8419097	0.9724078
Smad2	-0.0299329	5.5840659	0.8420114	0.9724078
Commd7	-0.0243494	4.9632415	0.8420531	0.9724078
Plod2	0.0745533	6.8777461	0.8421395	0.9724078
Cav1	0.0600792	3.5281158	0.8421642	0.9724078
Gm10536	-0.1614856	-0.250054	0.8421931	0.9724078
Vmn2r-ps23	-0.1067172	-0.1710656	0.842242	0.9724078
1110002L01Rik	-0.0448478	3.0397652	0.842245	0.9724078
Alg14	0.0423884	3.3069494	0.8424449	0.972433
Taf1a	0.0552986	2.915096	0.8424713	0.972433
Foxc2	-0.0988857	1.0802888	0.84254	0.972433
Mzt2	0.0391169	3.163769	0.8426409	0.972433
Rab24	-0.0250363	5.6134701	0.8426477	0.972433
Stom	0.0293061	8.5725158	0.8426541	0.972433
Trpm4	-0.076928	2.0333396	0.8427045	0.972433
Dusp28	0.0582054	2.3861722	0.8429085	0.9725964
Gm16201	0.1077566	-0.1562869	0.8430888	0.9727323
Birc3	-0.0301454	5.7740581	0.8432343	0.9727964
Gm15975	0.1954843	0.3672826	0.8432695	0.9727964
Mxra8	0.0527924	5.1705211	0.8433632	0.9728324
Cops4	0.0260918	5.8326024	0.8434521	0.9728379
Atp6v0d1	-0.0270569	6.5325438	0.8435409	0.9728379
Gm11478	-0.0658081	1.8408958	0.8435887	0.9728379
Cnih4	-0.0291203	5.13147	0.8436407	0.9728379
Slc37a4	-0.040829	3.4533441	0.8437881	0.9728379
Serpina3n	-0.1781418	-0.2776262	0.8438459	0.9728379
Eya2	-0.0625741	1.5228405	0.8439499	0.9728379
Armcx2	0.0445818	4.1484407	0.8439516	0.9728379
Klhdc7a	0.216443	-0.86394	0.8441017	0.9728379
Trim65	0.0516631	3.4645937	0.8442203	0.9728379
Bicd2	0.0224463	6.3686051	0.8442733	0.9728379
Gpr183	-0.0441345	3.2875274	0.8443442	0.9728379
Vps26a	-0.0220194	6.4859844	0.8443606	0.9728379
Rassf4	0.0303751	6.4408027	0.8443868	0.9728379
Psmc3ip	-0.038705	4.2241531	0.8444375	0.9728379

Dnah2	-0.1016523	-0.3385843	0.8445305	0.9728379
Plin4	0.1423942	2.7320721	0.8445665	0.9728379
Mcat	0.0431716	3.0614722	0.8446592	0.9728379
Fbxo42	0.0252686	5.947034	0.8447056	0.9728379
Rufy4	-0.0570093	3.2830085	0.8447804	0.9728379
Gm550	-0.1074865	-0.8552264	0.8448665	0.9728379
Thbs2	0.0646388	3.8172678	0.8449243	0.9728379
Gcnt2	0.0354274	5.349323	0.8449681	0.9728379
Zfp738	-0.0353597	3.7912826	0.8449792	0.9728379
Sh3pxd2a	-0.0460456	6.0537194	0.84499	0.9728379
1700001L05Rik	-0.0457935	3.5073565	0.8449942	0.9728379
Cyfip2	-0.0354621	8.0671608	0.8451024	0.9728904
Mfsd7a	0.0719211	2.0237941	0.8454751	0.9730264
Adam5	-0.186065	-1.1368337	0.8455136	0.9730264
Cmpk1	-0.0215197	6.7553763	0.84554	0.9730264
Usp39	-0.0237388	6.1028913	0.8457552	0.9730264
Mpped2	0.1059231	0.8608085	0.845794	0.9730264
Zfp456	0.0535738	2.7279874	0.8458576	0.9730264
Wdr77	0.0238769	5.3982972	0.8459128	0.9730264
Cops3	-0.0245321	6.2382993	0.8460187	0.9730264
Kdelr3	0.0749653	3.9400443	0.8461975	0.9730264
4833419F23Rik	-0.1394526	-0.7849897	0.8462305	0.9730264
Mlh1	0.0329697	4.3519616	0.8463576	0.9730264
Gm8991	0.1375553	-0.7157994	0.8463984	0.9730264
Anapc10	0.0306094	4.0390137	0.8465185	0.9730264
Jam3	-0.0758848	1.9747368	0.8465896	0.9730264
Slc30a9	-0.0331252	7.0318352	0.8465915	0.9730264
Nudt3	-0.0226917	6.5984779	0.8466038	0.9730264
Ap4m1	0.0255224	4.8191696	0.8466205	0.9730264
Secisbp2	-0.0270459	4.8743084	0.8466234	0.9730264
4833439L19Rik	0.0255467	5.7654657	0.8467403	0.9730264
Glo1-ps	-0.133974	1.2562642	0.8468098	0.9730264
Pramel12	-0.0376198	3.3949981	0.8468579	0.9730264
Ptrhd1	0.0412561	3.0058922	0.8469528	0.9730264
Cyb5a	-0.0331944	7.0823231	0.8469998	0.9730264
Trpt1	0.0589745	1.7899837	0.8470525	0.9730264
Gm10244	0.1203268	-0.4752425	0.8470728	0.9730264
Nae1	0.0377875	4.6821347	0.8471333	0.9730264
3110056K07Rik	0.0406319	3.3012956	0.8471494	0.9730264
Cmtm7	-0.0292278	6.35495	0.8471974	0.9730264
Gm16938	0.0992589	0.2581117	0.84721	0.9730264
Ecm2	-0.0611067	2.0352612	0.8472307	0.9730264
Actr1a	0.0207613	6.7195023	0.847332	0.9730264

LOC118567734	0.1067538	-0.3584493	0.8474151	0.9730264
Akr1c12	-0.0863973	0.7852544	0.8474328	0.9730264
Palb2	-0.0347123	4.2041068	0.8474378	0.9730264
Tuba4a	-0.0273609	8.9588453	0.8474599	0.9730264
Pde12	0.0256802	4.8468993	0.8474983	0.9730264
Glt1d1	-0.0810232	1.4320063	0.8475352	0.9730264
Vim	0.026116	8.8837431	0.8477218	0.9731688
Slit3	0.0447959	4.7808886	0.8478939	0.9732858
Rrp7a	0.0228152	5.192108	0.8479999	0.9732858
Tshz1	0.0280624	4.9546179	0.8480114	0.9732858
Tbc1d12	-0.0413705	3.5263562	0.8481847	0.9734129
Vps11	0.0249987	5.1536992	0.8484969	0.9736952
Crispld2	-0.0529997	5.2276189	0.8485559	0.9736952
Hoxc10	-0.0831077	2.1468941	0.8486915	0.9737789
Gm32511	0.148881	-1.1370954	0.8489059	0.9739531
Hes1	0.0443448	3.0555168	0.8489708	0.9739558
Zfp101	0.0388128	4.8973355	0.8491412	0.9740794
Gm14292	-0.0568581	3.2154646	0.8493351	0.9741627
Metap1d	0.043418	4.045858	0.8493391	0.9741627
Stk38l	0.0396257	3.4383226	0.8494291	0.9741826
Cul9	-0.0414183	2.7032189	0.8494817	0.9741826
Gm13135	-0.0805114	-0.3997776	0.8497306	0.9742754
Cfap45	0.0816925	0.700443	0.8498942	0.9742754
Dcp1a	-0.0242474	5.3487877	0.8499801	0.9742754
Psma8	-0.0828448	1.5390767	0.850246	0.9742754
Stk3	-0.0237508	5.1335925	0.8502501	0.9742754
Shank1	0.1055641	1.0826705	0.8502658	0.9742754
Gm14305	0.0569808	1.3721872	0.850273	0.9742754
Gm10076	0.0292047	6.1574026	0.8503491	0.9742754
Dlx3	0.0747136	3.7418433	0.850352	0.9742754
Sil1	-0.0387382	3.7946811	0.8503756	0.9742754
Map2k4	-0.0217381	6.2628186	0.8505185	0.9742754
Ccdc66	-0.0319131	3.6215233	0.8505389	0.9742754
Arhgap27os3	-0.0705075	2.9878098	0.8505436	0.9742754
Akt2	0.0199308	6.5580724	0.8506587	0.9742754
Pds5b	0.0247887	6.6961582	0.8506751	0.9742754
Aplp1	0.1189425	0.4104354	0.8507227	0.9742754
Sgip1	-0.0728386	1.3670851	0.8508651	0.9742754
Dhcr7	0.0436749	3.6676404	0.8508721	0.9742754
Gabarapl1	0.0225042	5.960876	0.8508749	0.9742754
Pi4ka	-0.0263802	6.0569481	0.8508999	0.9742754
9930104L06Rik	-0.0517709	2.9537913	0.8509078	0.9742754
Mars2	-0.0378809	3.1565809	0.8509407	0.9742754

Rpl5-ps1	0.0800992	-0.8572022	0.8511292	0.9743098
Gm6851	-0.1227934	-0.3527944	0.8511886	0.9743098
Amz1	0.0562042	2.3640318	0.8512066	0.9743098
Ppm1f	0.0276447	4.7137193	0.8512213	0.9743098
Fdx2	0.0937172	0.0755813	0.8513153	0.9743457
Gm39523	-0.1020402	-0.7090736	0.8515793	0.9745761
Fbxo6	-0.0289018	3.7789036	0.8517922	0.974748
Dennd4b	0.0329148	5.4925795	0.851943	0.9748489
Cryz	-0.0592828	2.2255272	0.8524432	0.9753496
Ttll11	-0.0500684	2.6654041	0.8525405	0.9753891
Pogk	-0.0392644	3.6817732	0.852843	0.9756265
Plekhn1	0.0439762	2.7519212	0.8529229	0.9756265
Borcs6	-0.0292094	4.4094281	0.8529362	0.9756265
Top2a	-0.0259258	9.7381774	0.853078	0.975717
Gm3222	0.1797279	-1.4898192	0.8532203	0.975755
Josd2	0.0503305	6.0932725	0.8532367	0.975755
Fnip2	0.0304786	5.9011495	0.8533472	0.9758096
Rnps1-ps	-0.0375589	4.5581724	0.8536109	0.9760393
Npnt	0.0528945	4.209176	0.8540698	0.9763411
Jmy	-0.0231955	5.7572475	0.8540907	0.9763411
Ccl27a	0.068234	1.3479172	0.8541059	0.9763411
Fundc2	-0.026391	6.1200517	0.854126	0.9763411
Lypd11	-0.1059306	-0.2783924	0.8542442	0.9763411
Cdc37	-0.0243055	7.2079651	0.8542514	0.9763411
1700084E18Rik	-0.1206163	-1.2559728	0.8543579	0.9763911
Camkk1	0.0568641	2.0142406	0.8545367	0.9765236
Loxl2	-0.0709122	6.0988087	0.8547349	0.9766784
9330133O14Rik	-0.0342803	3.1919998	0.8548826	0.9767341
Smim19	0.030839	4.0175467	0.8549431	0.9767341
Dusp11	0.0214801	7.2151423	0.854972	0.9767341
Prkd2	-0.0299512	5.6590878	0.8552035	0.9769267
Trpc4ap	0.0214591	6.780669	0.8553989	0.9770563
Pcif1	0.0228481	5.9308599	0.8554425	0.9770563
Unc45b	0.0906969	0.5284385	0.8557503	0.9770604
Zkscan7	-0.0651905	1.5540572	0.855977	0.9770604
Cpq	-0.0346222	4.94825	0.8560133	0.9770604
Polr2h	0.039957	3.6960893	0.8560431	0.9770604
Gm31656	0.1407443	0.0734319	0.8560637	0.9770604
Fam114a1	-0.0458891	4.7122405	0.8560756	0.9770604
Ints13	-0.0229116	6.1442984	0.8560877	0.9770604
Pigk	0.0217894	5.2277938	0.8561422	0.9770604
Klra7	0.1224563	-0.3896207	0.8561816	0.9770604
Gm5960	-0.0736299	0.4155857	0.8561976	0.9770604

1110019D14Rik	0.0582644	1.2140392	0.856222	0.9770604
Zfp874b	0.0300572	4.2592066	0.8562639	0.9770604
Gm39210	-0.1448449	-0.827565	0.8562886	0.9770604
Gabarap	0.0246026	8.0981543	0.8563255	0.9770604
Vmn2r-ps16	-0.0750273	2.9939046	0.856633	0.9773395
Eapp	-0.0228081	5.3070262	0.8571285	0.9775939
Dcaf17	-0.0276277	4.4107724	0.8571333	0.9775939
Osbpl7	0.0332559	4.0374561	0.8572083	0.9775939
Mis18bp1	0.0247764	6.1587963	0.8573142	0.9775939
Col16a1	0.0655909	3.8792946	0.8575071	0.9775939
Eya3	-0.0213601	5.8804826	0.8575082	0.9775939
Cbx4	-0.0228743	5.7308908	0.857522	0.9775939
Agfg1	-0.0200289	6.5820383	0.8575812	0.9775939
Afg3l1	-0.0228847	5.699765	0.8576353	0.9775939
Gls2	-0.0840976	0.6337697	0.8577173	0.9775939
Tpbgl	-0.1159911	-0.6344061	0.8577987	0.9775939
Mrps36	-0.0270781	4.8733728	0.8580236	0.9775939
Pkd1l1	0.0895042	0.4668092	0.8580686	0.9775939
Macroh2a2	-0.0703299	2.2896044	0.8581801	0.9775939
2610001J05Rik	0.0281641	4.5331252	0.8582088	0.9775939
Gm35343	-0.1126611	-0.832923	0.8583746	0.9775939
Shroom2	-0.0500663	2.67945	0.8583769	0.9775939
Lsm8	-0.025674	5.4133868	0.8584556	0.9775939
Ppa2	-0.0274565	4.3261195	0.8585064	0.9775939
Tatdn1	0.0411203	4.0013369	0.8585608	0.9775939
Taf13	0.0310976	4.3893631	0.8585785	0.9775939
2500004C02Rik	-0.0439837	2.124545	0.8586068	0.9775939
Trp53bp1	-0.02683	4.5676023	0.858625	0.9775939
Usp22	0.0259576	5.2352049	0.8587297	0.9775939
Slc41a3	-0.0334688	3.5388903	0.8587813	0.9775939
H3f3c	0.0685278	1.3366406	0.8587878	0.9775939
Gtpbp1	-0.0258749	6.9088705	0.8588664	0.9775939
Pfn2	0.0683693	2.2411515	0.8589922	0.9775939
Grn	0.0229776	7.8398824	0.8589923	0.9775939
Tor3a	-0.024338	4.6571651	0.8589934	0.9775939
Fastkd2	0.0440491	3.720596	0.859006	0.9775939
Pm20d2	-0.0819033	0.9700076	0.8590329	0.9775939
Dpp7	-0.0403986	3.3661782	0.8590721	0.9775939
1110020A21Rik	0.0741246	0.850998	0.8591206	0.9775939
Kntc1	-0.0287629	6.253826	0.859127	0.9775939
Rpf1	0.0219972	5.6177144	0.8591605	0.9775939
Haghl	0.0453231	3.2032301	0.8592623	0.9775939
Laptm5	-0.0260338	8.7109915	0.859338	0.9775939

Cxcr3	-0.0578209	1.4469858	0.8593962	0.9775939
Tmem179b	-0.0221245	5.1481512	0.8594814	0.9775939
Upb1	-0.0766029	1.1955489	0.8595191	0.9775939
Ufl1	-0.0250946	4.9058341	0.8595412	0.9775939
Gm4294	-0.1064167	-0.3792241	0.8595586	0.9775939
Gm16062	0.1651065	-0.6384105	0.8598224	0.977785
Las1l	-0.0205822	5.292779	0.8599986	0.977785
Igkv1-88	-0.0916521	1.2564291	0.8600354	0.977785
2310022B05Rik	-0.0308147	5.9531804	0.860047	0.977785
Pcbp3	-0.0487816	1.4939228	0.8601816	0.977785
Cip2a	-0.0211135	6.6969366	0.8603557	0.977785
Rnf43	0.0688812	1.2457166	0.860395	0.977785
A530016L24Rik	0.1293506	0.4011959	0.8604171	0.977785
Arfgap3	0.0284659	4.7852196	0.8604842	0.977785
Rnf19a	-0.0262204	6.263534	0.8605356	0.977785
Ddr2	0.0486915	5.7755453	0.8605627	0.977785
Tmem169	0.1315623	-0.3293141	0.8606137	0.977785
Mex3d	-0.0430267	3.1259367	0.8608555	0.977785
Nav2	-0.029571	4.1017659	0.8609508	0.977785
Depdc1a	0.0359991	5.91815	0.8610528	0.977785
Tle6	-0.0571837	1.1445148	0.8610719	0.977785
Lypd6b	0.0862366	0.8344673	0.8611263	0.977785
Nbn	-0.0251685	5.1166941	0.8612015	0.977785
Smc3	-0.0201268	7.8562529	0.8613013	0.977785
Tgfbr3l	0.1375405	-0.152553	0.8613202	0.977785
Rpl36-ps2	-0.0879698	0.3735213	0.8613495	0.977785
Whamm	0.030205	4.6306338	0.8613849	0.977785
Mrps11	0.0271264	4.1099121	0.8614174	0.977785
Atp6v1d	-0.020633	6.6114729	0.8614932	0.977785
Rps25	0.0266457	9.4080483	0.8615359	0.977785
Shisa5	-0.0230703	6.8249442	0.8615624	0.977785
Pigf	-0.0445639	2.4809085	0.8615774	0.977785
Supt5	0.0223577	6.7518766	0.8615938	0.977785
Zscan12	0.0443643	2.8195619	0.8617557	0.977785
Gm2921	-0.1702806	-1.1528963	0.8617586	0.977785
Vdac2	0.020078	7.5855366	0.8618529	0.977785
Pla2g10	0.1489556	-1.2581718	0.861933	0.977785
Polr2k	-0.0339689	4.9674932	0.8619677	0.977785
Ino80c	0.0209454	5.578666	0.8619787	0.977785
Miga1	0.0293267	4.0484942	0.8619918	0.977785
Abcb9	0.0487307	2.7491853	0.8620775	0.977785
Gipc2	-0.0961533	-0.5324149	0.8621069	0.977785
Kptn	0.0343415	3.0653124	0.8624008	0.977785

Yipf3	-0.0183631	5.3668401	0.8624036	0.977785
Mmrn2	0.0365106	4.2238437	0.8624613	0.977785
Prdm9	0.0609882	3.0092071	0.8625236	0.977785
Get1	-0.0402552	2.7268499	0.8625431	0.977785
Gtf2h1	0.0197831	5.7624761	0.862558	0.977785
Ints1	0.0295435	5.7467958	0.8627283	0.977785
Pcid2	0.0262467	4.6723581	0.8628946	0.977785
Usp25	0.0259455	7.9790732	0.8629073	0.977785
Kbtbd2	-0.0182412	6.0456098	0.8629259	0.977785
Atf6	-0.0203628	6.0444868	0.8629897	0.977785
Cd180	0.0413896	4.2530752	0.863011	0.977785
BC049762	0.1460218	-0.9911647	0.8630336	0.977785
Gm8520	-0.0850591	-0.3452167	0.8631508	0.977785
Ttc33	0.0236147	4.5035506	0.8631732	0.977785
Tmem260	0.0388556	4.1260587	0.863174	0.977785
Nudt2	-0.0506938	2.7292861	0.8631857	0.977785
Zfp28	-0.0567529	1.7501517	0.8632851	0.977785
Sat2	0.1204531	0.3898525	0.8633072	0.977785
Daglb	-0.0243717	4.8824944	0.8634127	0.977785
Padi3	-0.0769741	-0.5502047	0.8634256	0.977785
Zfp36l2	0.0333855	7.886324	0.8636393	0.977785
2610008E11Rik	-0.0260659	4.1622035	0.8637254	0.977785
Sec22b	0.0201445	5.9427367	0.8638577	0.977785
2510039O18Rik	-0.0183882	5.8640482	0.8638796	0.977785
Dguok	-0.029096	3.7752689	0.8640046	0.977785
Psmc2	0.0208167	6.7592665	0.8640074	0.977785
Fbxo31	-0.0235826	4.3143603	0.8640933	0.977785
2610507B11Rik	-0.0198284	7.439526	0.8640959	0.977785
1700034H15Rik	-0.0976822	-0.2314326	0.864179	0.977785
Tshz2	-0.0428743	3.2395025	0.8641862	0.977785
Ccar1	0.0222062	7.3302328	0.8642489	0.977785
Uvssa	0.0291775	4.7559171	0.8642507	0.977785
Nipa2	-0.0188159	5.6823353	0.8642836	0.977785
Clmn	-0.0934119	-0.0855246	0.8643209	0.977785
Msx1	0.1185638	0.1426365	0.8644619	0.977785
Gin1	0.0346517	3.89808	0.8644765	0.977785
Zfp68	-0.0193749	5.3364135	0.8644909	0.977785
Zc3h14	-0.0177143	6.5602986	0.8645411	0.977785
Ccdc148	-0.0891529	0.6640728	0.8647269	0.977785
Ldah	-0.0200888	5.2472749	0.8648153	0.977785
Pdcd2l	0.0343714	3.3797355	0.864885	0.977785
Tmx2	-0.0225536	5.114545	0.8649528	0.977785
Fhod1	-0.0227649	5.600919	0.8650318	0.977785

Adamtsl2	0.0832599	0.5208015	0.8651262	0.977785
Bbs5	0.065059	0.875183	0.8651375	0.977785
Tut7	0.0214428	8.0991779	0.865201	0.977785
Map1lc3b	-0.0190083	7.3680772	0.8652223	0.977785
C2cd2l	-0.0265848	4.7170732	0.8652603	0.977785
Gm7045	0.1320147	-0.1888478	0.8652724	0.977785
Cables1	0.047161	2.0590213	0.865297	0.977785
Gm45986	0.0895742	0.072278	0.8653215	0.977785
Depdc5	-0.0265263	4.7941503	0.8655078	0.9779245
Dhrs7b	0.0338081	3.8259046	0.8657097	0.9779698
Fez2	-0.023635	4.147435	0.8658102	0.9779698
Alkbh4	0.0322777	3.457817	0.8659141	0.9779698
Wdr44	-0.0246943	4.9782546	0.865969	0.9779698
Mapk7	-0.0264554	4.3471016	0.8660385	0.9779698
Zfyve19	0.0312095	3.5784302	0.8660861	0.9779698
Ms4a6b	-0.0331236	4.1215715	0.8660916	0.9779698
Tom1l1	0.0431332	5.4754132	0.8661713	0.9779698
Mybl1	-0.0319401	3.1550562	0.8662641	0.9779698
LOC108167553	-0.0507365	2.2500766	0.8663362	0.9779698
2310001H17Rik	0.0683693	1.1590319	0.8664079	0.9779698
Wdr12	0.0236936	5.1117998	0.8664243	0.9779698
Siglecf	0.0499399	3.6872319	0.8664371	0.9779698
Rps3	0.025202	10.001915	0.8665095	0.9779698
4933430I17Rik	-0.1530715	-0.6253654	0.8665133	0.9779698
Zbtb3	0.0675025	1.1169369	0.8665566	0.9779698
Gm11868	-0.0966763	3.0975195	0.8666494	0.9779698
Eid1	0.0224656	7.292492	0.866683	0.9779698
Akap7	-0.0295175	4.6561481	0.8667865	0.9779698
Ccnb1	0.028343	7.2288458	0.8668055	0.9779698
Ddx23	0.0236446	6.4163065	0.8670405	0.9780819
Smn1	0.0209269	4.7205138	0.8670703	0.9780819
Snrnp35	-0.0261788	3.9535341	0.8673744	0.9780819
Gm11663	0.1086127	-0.5723906	0.8674199	0.9780819
Gen1	0.022049	5.4725086	0.8674353	0.9780819
Vrk2	0.0509206	4.7542343	0.8674911	0.9780819
Ppp1cc	0.024305	7.5601397	0.8675146	0.9780819
Tbc1d19	-0.0335345	2.9396518	0.8675537	0.9780819
Gnai1	0.0633199	4.3211439	0.8676317	0.9780819
Gpt2	-0.0539191	1.989321	0.8676357	0.9780819
Gm4353	0.1085256	0.6894521	0.8676759	0.9780819
Qsox2	-0.0364218	3.3339424	0.8677967	0.9780819
Gtf2b	-0.0210006	5.4802219	0.8678976	0.9780819
Sox5	0.0819002	0.6584092	0.8679151	0.9780819

Smndc1	0.0192145	5.728374	0.8681432	0.9780819
Bsn	0.0425946	2.7871029	0.8681744	0.9780819
Zfp157	-0.0303788	3.8728234	0.8682153	0.9780819
LOC118568377	-0.0605949	1.5349872	0.8682469	0.9780819
Mthfsd	-0.0290347	3.7077445	0.8682531	0.9780819
Slc25a29	-0.0631017	0.5400556	0.868294	0.9780819
A930017M01Rik	-0.1206577	-0.2413142	0.8683085	0.9780819
Zfp207	0.0190798	8.1651296	0.8683133	0.9780819
Gm5296	0.0591695	2.4555406	0.8684605	0.9780819
Tbc1d31	0.0285245	4.9370195	0.8684708	0.9780819
Hoxa9	-0.0351989	3.1038709	0.8684769	0.9780819
Atad5	-0.0253918	6.5991734	0.8687086	0.9781602
Nudt6	0.0528028	1.4742576	0.8687589	0.9781602
Sh3d19	0.0234696	4.6777768	0.8687817	0.9781602
Wnt4	0.059072	2.8725373	0.8687979	0.9781602
Nudt4	-0.021511	8.536585	0.8689185	0.9782251
Casp7	0.0254976	5.0558667	0.8690896	0.9782603
Batf	-0.0391839	2.7020868	0.8691213	0.9782603
9530052C20Rik	-0.0602361	-0.2663907	0.8691384	0.9782603
Pgm2l1	-0.0217824	6.0203238	0.8694318	0.9785196
Dtl	0.0255241	6.8093799	0.8695795	0.9785651
Gm5171	0.0893912	-0.4269326	0.869598	0.9785651
Trim6	-0.092399	-0.0730288	0.869898	0.9787382
Ankrd49	-0.0268823	4.9317027	0.8699429	0.9787382
Ss18	0.0207681	6.2892343	0.8701421	0.9787382
Sirt5	0.0529787	1.9630769	0.8701735	0.9787382
Snx20	0.0274458	5.357943	0.8702416	0.9787382
Tnfrsf25	-0.103714	-0.1789663	0.8702789	0.9787382
Ier5	-0.0233023	5.5772484	0.8703043	0.9787382
Fam53c	-0.0182771	5.4258903	0.8703052	0.9787382
Ebag9	0.0241119	4.1146266	0.8703182	0.9787382
A530032D15Rik	0.0788289	1.1133448	0.8704514	0.9788173
Usp44	0.0762957	0.5963653	0.8705437	0.9788504
Osm	0.0573906	3.1626689	0.8706614	0.9788724
AU022252	-0.0307981	3.5630302	0.8706892	0.9788724
Plvap	-0.0210119	5.6614171	0.8709039	0.9789242
Bfar	-0.0191947	5.2545125	0.8709285	0.9789242
Ppil4	-0.0174427	6.2422506	0.8709793	0.9789242
Gnpat	-0.0193804	6.1295588	0.8711075	0.9789242
Stard9	0.0255289	3.9167307	0.8712003	0.9789242
Thbs3	-0.0808967	1.0901469	0.8712921	0.9789242
C330013E15Rik	0.0895057	-0.6505679	0.8713035	0.9789242
Plpp5	-0.0242718	4.4813026	0.8713548	0.9789242

Six5	0.0471636	2.7907768	0.8713729	0.9789242
Pxmp4	-0.0189519	4.8162382	0.8714554	0.9789242
Xrn2	-0.020316	6.8063061	0.8714778	0.9789242
Slf1	-0.0262392	4.6373357	0.8715558	0.9789242
Sdad1	0.0186233	5.5530815	0.8715778	0.9789242
D5Ertd605e	-0.0876478	0.5431339	0.8717136	0.9789242
Nup98	-0.021991	6.2493999	0.8719362	0.9789242
Fbn2	-0.0505757	3.9799582	0.8719377	0.9789242
Gm2a	-0.0205193	7.092231	0.8720031	0.9789242
D830050J10Rik	-0.0703834	0.7924807	0.8720109	0.9789242
Dmrtb1	-0.1051315	-0.4805951	0.8720536	0.9789242
Sord	-0.0190831	5.7977769	0.8721102	0.9789242
Slc26a1	-0.0625827	2.4530492	0.8722201	0.9789242
E2f5	0.0419658	2.3091948	0.8722484	0.9789242
5033417F24Rik	-0.1102933	-0.7040259	0.8722653	0.9789242
Tbx18	-0.0977491	0.8808558	0.8722833	0.9789242
Cul2	0.0207071	5.2394725	0.8723942	0.9789242
2310009B15Rik	0.0418835	2.4039887	0.8724345	0.9789242
B3galt5	0.121923	-0.9174696	0.8724346	0.9789242
Fam169a	0.0734935	-0.1770325	0.8725236	0.978951
Coa7	0.0405773	2.7591274	0.8725843	0.978951
Mtmr4	0.0232112	5.5349319	0.8727384	0.9789678
Krba1	-0.045012	2.4192306	0.8727764	0.9789678
Abhd11	-0.0273593	4.0857756	0.8727881	0.9789678
Cacna2d1	-0.0390626	3.4113987	0.873068	0.9790242
Elp1	0.018717	5.1950955	0.8730883	0.9790242
Slc6a4	-0.0273903	5.6200202	0.8731176	0.9790242
Lman1	-0.0191766	6.8533799	0.8732248	0.9790242
Vti1b	-0.0185158	5.6137555	0.8733093	0.9790242
AA386476	0.0488457	1.8813697	0.8733138	0.9790242
Jmjd6	0.0268939	4.9648518	0.8733329	0.9790242
Itpripl2	-0.0329785	6.5246598	0.8734326	0.9790242
Sub1	0.0218862	7.3919041	0.873568	0.9790242
Rab15	-0.0672315	-0.6940501	0.8735746	0.9790242
Gm6206	0.0587698	0.6662516	0.8736092	0.9790242
Tcra	0.0497848	2.3022304	0.8736474	0.9790242
Cdk11b	0.0181882	6.6955086	0.8736567	0.9790242
Xaf1	0.0411032	3.0722183	0.8740861	0.9794349
Leo1	0.0227127	5.1250371	0.8742669	0.9795669
Gm38521	0.0562369	1.8652686	0.8746886	0.9799687
Ndufaf2	-0.0328144	3.3584195	0.8747725	0.97997
Pgap2	0.0271023	4.4825218	0.8748344	0.97997
Lcmt2	-0.0395658	2.9672816	0.8749406	0.97997

Zfp867	0.0746464	1.0176999	0.8749635	0.97997
Rnf169	-0.0310953	5.4721159	0.8750636	0.97997
Gm2223	0.0548589	1.8241925	0.8751057	0.97997
Spns3	0.0506779	3.3674891	0.8751866	0.97997
Atg10	-0.036149	2.2619772	0.8751937	0.97997
Nlrc4	-0.0316647	2.9372464	0.8753187	0.9799934
Rcan1	-0.0413829	4.6570866	0.8753407	0.9799934
BC024978	-0.04043	2.203754	0.8755123	0.9801151
Tada3	0.0234107	4.3715275	0.8757336	0.9801485
Mapk8	-0.0213247	4.5188664	0.8757735	0.9801485
Gm15518	-0.0548777	0.9032723	0.875776	0.9801485
Cep152	-0.0225279	5.0489735	0.8757943	0.9801485
Gm6104	0.0547624	0.502988	0.8762363	0.9804497
Cops5	-0.0240421	5.9456159	0.87624	0.9804497
Rbm42	-0.0166056	5.865507	0.8762525	0.9804497
Rasd1	-0.1019034	-0.3918024	0.8766042	0.9807727
Ptgfr	-0.0524742	2.5439992	0.8767541	0.9807959
Ticrr	-0.023795	5.3278523	0.8768906	0.9807959
Hoxc4	-0.0964237	0.3024938	0.8769209	0.9807959
Arv1	0.0353698	3.0672962	0.8769507	0.9807959
Slc22a21	0.0715005	1.171507	0.8770065	0.9807959
Nuggc	0.0928377	-0.3318028	0.8770271	0.9807959
Zfp629	-0.0246169	3.9548708	0.8770695	0.9807959
Crygn	0.1017573	-0.277423	0.8771975	0.9807959
Vps4a	0.0213863	5.3075452	0.877206	0.9807959
Mkln1	-0.0175324	6.4803641	0.8772555	0.9807959
Gm26614	-0.066565	-0.3589761	0.8773773	0.9808289
Pdia4	-0.0180383	6.5134948	0.8774111	0.9808289
Tagap	-0.0283598	4.3692493	0.8775229	0.9808702
4930503L19Rik	0.0393574	5.0880473	0.8775743	0.9808702
Phf1	-0.0265925	4.7983484	0.8777375	0.9809453
Ak4	0.0807929	1.2418813	0.877935	0.9809453
Ipp	-0.0269971	4.4766673	0.8779542	0.9809453
Pter	0.0534813	2.6835591	0.8780632	0.9809453
BC031181	-0.0204664	5.8230647	0.8780722	0.9809453
C430049B03Rik	-0.0807516	0.5666975	0.8781623	0.9809453
Pfdn1	0.0239782	5.2414935	0.8781686	0.9809453
Elac2	-0.0225245	4.3762111	0.878183	0.9809453
Ednrb	0.0875748	1.2213973	0.8782412	0.9809453
Adam19	0.0372413	5.7156819	0.8783189	0.9809453
Frat1	0.0320884	3.1642434	0.8783645	0.9809453
Shisa2	-0.0843082	0.2601791	0.8783983	0.9809453
Fkbp5	0.0334334	5.8839117	0.8786713	0.9810846

Snrpert	-0.0482596	2.382541	0.8787112	0.9810846
Nadsyn1	0.0350339	2.5477196	0.8787122	0.9810846
Megf8	0.0349913	4.3956517	0.8788732	0.9811939
Tfip11	0.0208295	5.2722853	0.8791244	0.9813965
Nthl1	0.0444505	1.484191	0.8791808	0.9813965
Mtx2	0.0264461	5.0900772	0.8792755	0.9814317
Inpp5a	0.018373	5.1800925	0.8795702	0.9815345
Dmac2l	-0.031918	2.9026486	0.8796895	0.9815345
Rgmb	-0.0453912	2.4988533	0.8797054	0.9815345
Tango2	0.0286055	4.6130246	0.8797091	0.9815345
Ppip5k1	0.0234497	4.1358007	0.8799074	0.9815345
Vps33a	0.0207445	5.3203277	0.8799325	0.9815345
AW011738	-0.0377726	2.7178491	0.8799717	0.9815345
Gusb	-0.0205309	6.2830336	0.8799717	0.9815345
Gm14321	-0.0720156	0.561169	0.8799807	0.9815345
Hdhd3	0.1054859	0.2736921	0.880089	0.9815345
Gm8539	-0.1041474	-0.9361432	0.8801233	0.9815345
Huwe1	-0.01942	7.7594541	0.8801728	0.9815345
Gata2	-0.0282627	4.0458031	0.8801879	0.9815345
Trim32	0.0422504	2.9197524	0.8803558	0.9816019
Gm4799	0.082691	-0.6878461	0.8803746	0.9816019
Arl4d	0.0578988	2.118834	0.8804844	0.981654
Nf2	-0.018653	5.6810098	0.8807229	0.9818495
Cx3cl1	-0.0438555	2.055846	0.8811274	0.9819662
Rpl35a-ps5	0.1078626	-0.9186324	0.881181	0.9819662
Lsmem2	-0.075258	0.2205342	0.8811834	0.9819662
Rbbp8	-0.018551	6.1514597	0.8811941	0.9819662
Prpf18	0.0172899	6.2981181	0.8812021	0.9819662
Zcchc3	-0.0306703	3.2969566	0.8812631	0.9819662
Kank2	-0.0388801	4.1321367	0.8812695	0.9819662
Gm52419	0.0658367	0.3058715	0.8814416	0.9820876
Ubac2	0.023651	4.6817626	0.8816278	0.9822247
Arpp19	0.01849	6.8642282	0.881708	0.9822437
Ivd	0.0220383	4.6268865	0.8821912	0.9825272
D030028A08Rik	-0.0455804	2.1389102	0.8821965	0.9825272
Suv39h2	-0.0281866	4.0443031	0.8822411	0.9825272
Prkab1	0.0214686	6.4845099	0.8822471	0.9825272
Gm34861	0.1040315	-0.2874516	0.8823831	0.9825272
Vwa1	0.0746216	0.5030474	0.8823946	0.9825272
Ms4a4b	0.0559779	3.1097588	0.8824687	0.9825272
Fap	-0.0410805	6.8874183	0.8825599	0.9825272
Eps8l1	0.2433032	1.7814225	0.8825898	0.9825272
Prc1	0.022387	7.9009765	0.8825942	0.9825272

Marcksl1	-0.0169513	6.3066623	0.8827541	0.9826281
Glb1	0.0272704	5.5816435	0.8828112	0.9826281
Ccdc47	-0.0157729	6.7012348	0.8828957	0.9826467
Klf3	-0.0259027	7.871217	0.8829542	0.9826467
Btbd2	0.0242429	4.0604095	0.8830221	0.9826519
Gm8566	0.0706334	0.5942854	0.8832857	0.9828255
Vps18	-0.0228401	4.4785735	0.8833045	0.9828255
Ak2	-0.0165128	7.020478	0.8834553	0.9828935
Col7a1	0.0691198	0.2776226	0.8835537	0.9828935
Stoml2	0.021026	5.6853564	0.8835552	0.9828935
Sirt3	0.0335544	3.1992791	0.8837934	0.9829951
Zmynd11	0.018249	6.6427359	0.8838862	0.9829951
Dennd10	0.0220462	4.8367098	0.8838964	0.9829951
Igkv4-72	0.0432804	2.9865407	0.8838993	0.9829951
Gm7658	0.0638115	2.0970217	0.884035	0.9830189
Gm36266	0.0528489	1.6754929	0.8840471	0.9830189
Ctsw	0.0553726	2.0000786	0.8842631	0.983048
Gm13375	-0.0487768	1.4901944	0.8842653	0.983048
Rfc1	0.0202841	6.9367111	0.8842807	0.983048
Tbc1d10b	-0.016925	6.8060083	0.8845338	0.983048
Reep4	0.0289189	5.1306873	0.8845461	0.983048
Nr2f1	-0.0966321	-0.1421117	0.8845607	0.983048
Blvra	-0.0233895	4.8458257	0.8846138	0.983048
Clec14a	-0.0337012	4.1363078	0.8846435	0.983048
Josd1	-0.0182164	5.9553048	0.8848133	0.983048
Pole2	0.020988	4.7097416	0.8848878	0.983048
Gm37292	-0.0710035	0.917771	0.8849265	0.983048
Nkap	-0.0174067	4.7834473	0.8850091	0.983048
Fbxl14	-0.0187917	6.7238156	0.8851182	0.983048
Tdrd3	0.0257295	5.1142971	0.8851499	0.983048
Prpf6	0.0190105	5.8324934	0.885175	0.983048
Grm4	0.1814671	-0.1373631	0.8852065	0.983048
St18	0.0710644	0.4867114	0.8852108	0.983048
Actr8	0.0195616	4.7863101	0.8852799	0.983048
Gemin8	0.0380722	2.1522717	0.8853068	0.983048
H2-K2	-0.0461469	2.4305273	0.8853452	0.983048
Septin2	-0.0153183	7.6073911	0.8854005	0.983048
Rnf19b	-0.0167875	6.1637436	0.8856548	0.9831059
Lcat	0.0737434	0.9001447	0.8857635	0.9831059
Sh2d1b1	-0.0412504	2.1931124	0.8858546	0.9831059
Acbd5	0.0175005	5.609168	0.8859148	0.9831059
Pdp1	-0.0291562	2.7989405	0.8859727	0.9831059
Tnfrsf22	-0.0455973	1.4138554	0.8860148	0.9831059

Slc26a6	0.0497672	1.7266494	0.8860996	0.9831059
Gnb5	-0.0304314	2.6106554	0.8862231	0.9831059
Ctif	-0.0284439	3.5857774	0.8863032	0.9831059
Dnm1l	-0.0151543	6.4727243	0.8863507	0.9831059
Arl8b	-0.0154002	6.5261436	0.8863797	0.9831059
C030034I22Rik	-0.0268802	2.7785975	0.8864233	0.9831059
Qprt	-0.0827909	-0.0381813	0.8864603	0.9831059
Cd5l	-0.0459895	4.875035	0.8864814	0.9831059
5830433I10Rik	-0.0596624	0.0237267	0.8864968	0.9831059
Sdccag8	-0.0279195	3.8095417	0.8865217	0.9831059
Mir100hg	-0.0775451	1.5402931	0.8865454	0.9831059
Gm15387	0.0618213	1.9253229	0.8866368	0.9831059
Cox5b-ps	0.0427001	1.0274664	0.8866535	0.9831059
Itpkc	0.042716	2.1055476	0.8869035	0.9832377
Fat4	0.0414014	2.8248881	0.8869868	0.9832377
Zbtb17	0.0224751	4.0348356	0.8870774	0.9832377
Gm15545	0.0606058	0.1907986	0.887181	0.9832377
Ctnnb1	-0.0181747	8.7024973	0.8871948	0.9832377
Mre11a	0.0209321	5.3816409	0.8872103	0.9832377
Traf3ip3	-0.0269396	5.1139987	0.8872149	0.9832377
A930004D18Rik	-0.1044906	-0.5218308	0.8873886	0.9833392
Cnot3	0.0196054	6.3479906	0.8875001	0.9833392
Alg5	-0.0250001	4.2960867	0.887556	0.9833392
Krt86	-0.0657378	0.5863915	0.8875681	0.9833392
Tmx1	0.0181855	7.4434686	0.8876226	0.9833392
Calcr	-0.0654605	1.710861	0.8879544	0.9836367
Nynrin	-0.0294734	3.1455392	0.8880575	0.9836808
Crlf2	0.0305079	4.7547642	0.8881676	0.9837328
Clcn5	0.033836	3.612633	0.8882552	0.9837597
Pafah1b2	-0.0150509	6.6269658	0.8885075	0.9837923
Tcea1	-0.0163244	7.25023	0.8885752	0.9837923
Oas1g	0.1273468	0.0190304	0.8886081	0.9837923
Ifi203-ps	0.0371618	2.8154644	0.888691	0.9837923
4930486L24Rik	-0.0911772	-0.424446	0.8887208	0.9837923
Cul4b	-0.0150618	6.2170836	0.8888431	0.9837923
Rpsa-ps7	-0.0378311	2.0476904	0.8889301	0.9837923
Vapb	-0.0162644	5.9146094	0.88908	0.9837923
Pax8	0.0890714	1.1655812	0.8891174	0.9837923
Pou2f1	-0.0208613	5.7151105	0.8891582	0.9837923
Evc2	0.0551603	1.8666235	0.8891666	0.9837923
Morn2	-0.0371344	1.9045741	0.8891845	0.9837923
Erp27	0.09687	-0.2840216	0.8891872	0.9837923
Snhg10	0.0503478	1.4998376	0.8892293	0.9837923

Rgl1	-0.0182149	5.1666241	0.8892334	0.9837923
Car1	-0.0360606	8.2557138	0.8894119	0.9838821
Pigb	0.027506	3.723289	0.8894411	0.9838821
Top2b	-0.0167159	8.1065162	0.8897316	0.9840525
Dnm1	0.0686502	2.3881288	0.8897748	0.9840525
Nol3	0.0447134	1.1475697	0.8900542	0.9840525
A930037H05Rik	0.1894454	-1.1033211	0.8900693	0.9840525
Reps2	-0.0246257	3.5809955	0.8900813	0.9840525
Cebpzos	-0.0324504	3.2406392	0.8900901	0.9840525
Gvin-ps6	-0.0625444	2.9155686	0.8902362	0.9840525
Zfp72	0.0502477	1.6374694	0.8902633	0.9840525
Mt3	-0.0542926	3.5746298	0.890308	0.9840525
Lrfn3	-0.1184173	-0.7966663	0.8903459	0.9840525
Dph6	0.0230557	4.1530494	0.8903549	0.9840525
Actr6	0.0285097	3.6717909	0.8903896	0.9840525
Cyp4f13	-0.037008	2.6919565	0.890438	0.9840525
Rprd1b	-0.0187962	6.1567912	0.890605	0.9840525
Rcbtb2	-0.0182968	5.2170641	0.8907907	0.9840525
Gm15542	-0.054048	1.5642971	0.8908422	0.9840525
Shisa8	0.1221753	-0.2229551	0.8908547	0.9840525
Ube2b	0.0235897	6.8524075	0.8908937	0.9840525
Tmc1	0.1161488	-0.1331279	0.8909236	0.9840525
Tubd1	0.0249866	3.8622535	0.8910715	0.9840525
Rpsa-ps9	0.1307105	-0.672287	0.8911683	0.9840525
Spata7	0.0603099	1.314743	0.8911815	0.9840525
Guca1a	-0.0536998	0.7622614	0.8911842	0.9840525
Gm20045	-0.0386885	2.1866677	0.8912132	0.9840525
Plekhh2	-0.0348829	3.4570972	0.891273	0.9840525
Nhp2	0.0223709	5.9622415	0.8913897	0.9840525
Ppp2r5e	0.0173109	6.1616474	0.891419	0.9840525
Ifngr2	-0.0184036	5.9403646	0.8914468	0.9840525
Sema6a	-0.0284651	2.6827446	0.8915453	0.9840525
Tmem126b	-0.037175	2.8480697	0.8916074	0.9840525
Gm13736	-0.1047249	-0.8321061	0.8916125	0.9840525
Kif20a	0.021374	6.7773682	0.8916414	0.9840525
Kcnj15	0.0587468	1.2381874	0.8916829	0.9840525
Gxylt2	-0.0492825	4.8063036	0.8918734	0.9841723
Usp40	0.0258165	3.802865	0.891918	0.9841723
Abcc1	0.0190396	4.8665765	0.8923512	0.9845805
Gm17251	-0.0333323	1.7640307	0.8925817	0.9847516
Tpd52l2	0.0187384	5.813055	0.892656	0.9847516
Rxrb	0.0196171	5.4909398	0.892708	0.9847516
Pias4	0.0211705	4.0891861	0.8927921	0.9847516

Hmgb1-ps2	0.0894371	-0.4452703	0.8928229	0.9847516
Ccdc57	-0.0319946	2.2628899	0.8930022	0.9848629
Med23	0.0191803	4.9036016	0.8931385	0.9848629
Kif19a	-0.0500187	0.7411624	0.8931572	0.9848629
Dnajb6	-0.016226	6.8606609	0.893177	0.9848629
Zfp933	-0.02489	3.5950458	0.8934081	0.9850478
LOC118568180	-0.0470055	-0.1636726	0.893752	0.9853572
Fcgrt	-0.0186638	6.0371999	0.8942008	0.985493
Saal1	0.0273561	3.4369977	0.8942395	0.985493
Nrxn2	-0.0678915	-0.0528099	0.8942735	0.985493
Taf1	-0.0139855	6.7586193	0.8942951	0.985493
Bak1	0.0190367	6.5468041	0.8943063	0.985493
Zfp3	-0.0313321	1.5140778	0.8943185	0.985493
Aatf	0.0170922	4.8184185	0.8943601	0.985493
Inhbb	-0.0492579	2.3028517	0.8943821	0.985493
Wars	-0.0186955	4.9239637	0.8944584	0.9855073
Ntan1	0.018368	4.7448151	0.8945685	0.9855243
Mvb12a	0.0220523	4.2449453	0.8946006	0.9855243
Dcaf7	0.0176645	6.9297002	0.8949715	0.9858381
Sbk2	-0.0722288	0.2122084	0.8951482	0.9858381
Ggcx	-0.0322913	3.7548215	0.8951707	0.9858381
Fbxo8	0.0254189	4.5355984	0.8951768	0.9858381
Alad	-0.0263764	7.8997649	0.8952023	0.9858381
Rarres1	0.0470024	0.3583311	0.8953968	0.9859825
LOC118568652	-0.0495266	1.9593351	0.8956196	0.9861579
Unc50	0.018283	5.4299768	0.8957363	0.9861831
Ckmt1	0.0565533	0.2473476	0.8957812	0.9861831
4930453N24Rik	0.0143904	5.4319764	0.8959227	0.9861831
Rnpep	0.020571	5.6213258	0.8959773	0.9861831
AI506816	0.1351819	3.9413582	0.8959827	0.9861831
Garem1	-0.0313886	4.1648037	0.8960452	0.9861831
Dag1	-0.0200274	5.7342057	0.8961038	0.9861831
Prob1	-0.0448925	1.7543644	0.8961925	0.9861831
Gm13534	0.2702732	-1.1773662	0.8962131	0.9861831
Sstr1	-0.104398	0.3522132	0.8966052	0.9865448
Ext2	0.0183567	5.3013248	0.8966875	0.9865655
Creld2	-0.020846	4.6462335	0.8969114	0.9867421
Adamts12	0.0372646	3.1597349	0.8971863	0.9867643
Gm6055	-0.0532901	1.112978	0.8972456	0.9867643
Cpn1	-0.102928	-0.3731458	0.8972612	0.9867643
Hira	0.0204416	5.6045096	0.8973275	0.9867643
Trmt44	0.0325935	2.7275311	0.8973442	0.9867643
Ajuba	-0.0542077	0.9496971	0.8973524	0.9867643

Gtdc1	0.0213051	3.9203754	0.8974011	0.9867643
Lyrm2	-0.0177194	4.6292711	0.8974391	0.9867643
Scfd2	-0.0270811	3.2827987	0.8975499	0.9868164
C4b	-0.0188272	6.196306	0.8977441	0.9868719
Aimp2	0.020959	4.0573681	0.8977907	0.9868719
Mrps24	-0.0169386	5.1369726	0.8978335	0.9868719
Herc1	-0.0199982	8.0527565	0.8979399	0.9868719
Mrtfb	0.0230081	3.9405432	0.8979518	0.9868719
0610042G04Rik	0.0406286	1.6365392	0.8980661	0.9868719
Slfn5	0.0185517	5.8461794	0.898111	0.9868719
Tnfaip1	0.0253479	4.2091398	0.898156	0.9868719
Brip1os	0.0143451	6.5833618	0.8982505	0.9868719
E030030I06Rik	-0.0535939	0.9572639	0.8982566	0.9868719
Dpcd	-0.0230828	3.5491861	0.8983188	0.9868719
St3gal2	-0.0191489	6.6405002	0.8983618	0.9868719
Hacd1	0.022734	4.5605973	0.8985338	0.9869247
Nagpa	0.0243893	4.2729493	0.8987695	0.9869247
Otud6b	-0.0175141	5.5201177	0.898874	0.9869247
Faxc	0.0685642	0.5577978	0.8989302	0.9869247
Zfp672	0.0160251	5.1331429	0.8989302	0.9869247
Tfpi2	-0.0374129	1.5805947	0.8989962	0.9869247
Itgb1bp1	-0.0202295	4.2765542	0.8990351	0.9869247
Sec62	-0.0135424	7.5189335	0.8991033	0.9869247
Homer3	0.0247004	4.192361	0.8991276	0.9869247
4833420G17Rik	0.0173817	5.6080078	0.8992148	0.9869247
Il5ra	0.1133169	1.0980452	0.8992453	0.9869247
Grhl1	0.0745283	0.1984647	0.8993239	0.9869247
Tmem115	0.0228414	3.7938382	0.8993264	0.9869247
Gm32313	-0.0854297	0.0167337	0.8993554	0.9869247
Folr2	-0.0632154	0.4486797	0.8995101	0.9869247
Ext1	-0.0203072	5.431996	0.899536	0.9869247
Pcbd2	-0.0208592	4.0623403	0.8995625	0.9869247
Tnpo3	-0.0132091	6.6019858	0.8995767	0.9869247
Abcb8	-0.0195565	3.7584131	0.8996361	0.9869247
Haus6	-0.0147009	5.466229	0.8997008	0.9869247
LOC118568784	0.1513917	-0.7514971	0.899756	0.9869247
Fbxo28	0.0153995	5.2475523	0.899885	0.9869247
Dctn4	-0.0135822	6.0675143	0.8999814	0.9869247
Mx2	0.0520882	1.0657933	0.9000159	0.9869247
Kif1c	0.0193356	5.8593671	0.9000573	0.9869247
Ptcd3	0.018903	5.2157297	0.900075	0.9869247
Ptpn13	0.0290535	4.3810724	0.9001842	0.9869247
P3h1	0.0388161	5.0289487	0.9002255	0.9869247

Luzp1	-0.0230648	4.5212531	0.9003256	0.9869247
Ranbp6	0.0151942	5.0839665	0.9004074	0.9869247
Slc25a40	0.0254106	3.8733878	0.900474	0.9869247
Lsm14a	0.0135145	7.3529385	0.9005373	0.9869247
Gpr18	-0.035002	2.700074	0.9006826	0.9869247
Habp4	-0.0266054	3.3603499	0.9006997	0.9869247
Rbm28	0.0160713	5.3158919	0.9007861	0.9869247
Fnta	-0.0142252	5.6625762	0.9009046	0.9869247
Phf7	0.030398	3.0788788	0.9009156	0.9869247
Csf1r	0.0192101	7.4770606	0.9009164	0.9869247
Arid2	-0.0141894	7.0365661	0.9009456	0.9869247
Tlk1	0.0163823	7.4577618	0.9010055	0.9869247
Fbf1	-0.017258	4.7955202	0.9010743	0.9869247
Scmh1	0.0170323	4.87338	0.9010811	0.9869247
Igh	-0.0207699	9.8231956	0.9011383	0.9869247
Cdh11	0.0284113	7.0152061	0.9012525	0.9869803
9130008F23Rik	-0.0759416	-0.3070613	0.9014931	0.9871181
Ankrd35	0.0352729	2.6705882	0.9015052	0.9871181
Azin1	0.0196013	8.5310441	0.901641	0.9871711
Det1	-0.0229951	3.3760284	0.901702	0.9871711
Cdc27	-0.014426	7.0595984	0.9018031	0.9871711
Gorasp1	-0.0231287	3.7339198	0.9018075	0.9871711
Cxcl5	0.0489318	1.5372366	0.9019486	0.9872216
Gpr89	-0.0185483	4.1921626	0.9019989	0.9872216
Prokr1	0.0467511	3.1125271	0.9021241	0.9872216
Irx3os	0.1044958	-0.6428795	0.9021799	0.9872216
Gm36112	0.0677865	0.0042548	0.9022594	0.9872216
Ift43	-0.0440054	2.1280701	0.9022805	0.9872216
Neil3	0.0175422	6.438237	0.902298	0.9872216
Hps6	0.0277101	2.6511751	0.9023997	0.9872635
Cd300c2	-0.0230401	3.9519536	0.9027031	0.9872686
Rint1	0.0174784	4.7104009	0.9029654	0.9872686
Knop1	-0.0149016	5.5834877	0.9031303	0.9872686
Gbp9	0.0246266	4.7057478	0.9031522	0.9872686
Sgo1	0.0176129	5.9528296	0.9031892	0.9872686
Nfe2	0.0169374	7.7288851	0.9032312	0.9872686
Gpbp1	0.0137479	7.6847062	0.9032412	0.9872686
Zfp524	0.0267008	3.1264808	0.9032441	0.9872686
Me1	0.0269139	3.6055621	0.9032487	0.9872686
3010003L21Rik	0.1326733	-0.177192	0.9032702	0.9872686
Gm3320	0.083939	0.1765899	0.903271	0.9872686
Ercc6l2	-0.0155476	4.7197442	0.9032852	0.9872686
Junb	0.0241117	5.7592215	0.9033056	0.9872686

P2rx4	0.0210525	4.2869572	0.903308	0.9872686
Slc25a27	-0.0458072	1.6417692	0.9033824	0.9872686
Plppr3	0.0247584	4.5021667	0.90342	0.9872686
N4bp2l2	0.0137915	6.4387054	0.9036434	0.9873901
Stac3	0.0822945	-0.4687686	0.9038108	0.9873901
Strip2	0.0434654	2.0561118	0.9038132	0.9873901
Kremen1	-0.029046	3.5387266	0.9038289	0.9873901
Slc6a8	0.0381041	4.3011803	0.9038535	0.9873901
Met	-0.0228851	3.8999546	0.9039121	0.9873901
Ddx21	0.0180782	7.23547	0.9040632	0.9873922
Zfp618	-0.0331118	2.1602374	0.9040969	0.9873922
A430046D13Rik	-0.0288681	2.5859656	0.9041166	0.9873922
Arhgap12	-0.01967	4.644689	0.9041679	0.9873922
Ints4	0.0166481	5.1217253	0.9043784	0.9875001
Plekhf1	-0.0330858	3.1057307	0.9044355	0.9875001
2810002D19Rik	0.0366529	2.0929767	0.9046574	0.9875001
Atp6v0d2	-0.0324985	6.2255	0.904708	0.9875001
Clec4n	-0.0452866	2.0539318	0.9047115	0.9875001
Serpini1	-0.0448015	1.7145083	0.904776	0.9875001
Chrnb1	-0.0543734	0.4800929	0.9048031	0.9875001
Rpsa-ps12	0.0765111	0.0013978	0.9048723	0.9875001
Ddx5	0.0144452	9.9695425	0.9048844	0.9875001
Tmem127	-0.0125306	6.6987754	0.9051052	0.9875001
Cnga1	-0.1378617	-1.1666734	0.9051277	0.9875001
Plekhb2	-0.0146778	5.2581385	0.9051297	0.9875001
Snx9	-0.0130504	5.7856799	0.905205	0.9875001
Nsa2	-0.0142486	6.8613524	0.9052168	0.9875001
Fuom	-0.0442355	1.6453907	0.905219	0.9875001
Ifit3	-0.0351269	2.7784024	0.9054581	0.9876917
Rfc4	0.0220285	5.7944407	0.9056367	0.9877958
Dgkd	0.0222346	7.2249602	0.9058166	0.9877958
Ccdc43	0.0176411	4.2177276	0.9058522	0.9877958
Slc25a36	0.014643	5.6587125	0.9058839	0.9877958
Cyp51	-0.0276283	4.4240961	0.9058936	0.9877958
Map4k2	-0.0169322	6.7254371	0.9059739	0.9877958
Chrd	0.0607019	-0.3125748	0.9060338	0.9877958
Naa25	-0.0159683	5.1784956	0.9060616	0.9877958
Elk1	-0.0225018	3.4678023	0.9064723	0.9881742
Apba3	-0.0222268	3.5716588	0.9066062	0.988251
Pdlim2	-0.0243982	4.296828	0.9068219	0.9883686
Ccnjl	0.0357552	1.6945788	0.9068875	0.9883686
Zc3h10	-0.0210288	3.7950991	0.9069048	0.9883686
Kank3	-0.0260133	3.0988831	0.9071224	0.9885077

Naa20	0.0207159	4.8906606	0.9072073	0.9885077
Ppib	-0.0141317	7.7610132	0.9072381	0.9885077
Farp1	0.0362974	3.0702464	0.9072866	0.9885077
Gm34560	0.0898027	0.22391	0.9073926	0.9885533
Ciz1	0.0145611	5.607532	0.9074556	0.9885533
Lman2	0.0135308	7.3667607	0.9075664	0.9886047
Ret	-0.0484481	1.1101311	0.9077977	0.9887875
Brca1	-0.0169825	6.5417503	0.9080092	0.9889262
Col6a2	0.0346677	6.6023994	0.9081087	0.9889262
Nemf	0.0140123	6.0843755	0.9081213	0.9889262
Lin7a	-0.0624598	-0.1292831	0.9082083	0.9889262
Rnpepl1	0.0147228	5.2986864	0.9082429	0.9889262
Thsd7a	-0.042476	1.5973677	0.908416	0.9890453
Slc4a4	0.0496514	0.4044585	0.9085091	0.9890462
Rhbdd2	-0.0212856	3.092676	0.9085499	0.9890462
Kcnb1	0.052312	0.9813376	0.9086668	0.9890462
Rpl9-ps7	-0.0469951	0.3322619	0.9086711	0.9890462
H2bc6	0.0548731	0.7759656	0.9087497	0.9890626
Heph	-0.0269714	3.4478987	0.9090559	0.9892716
Pomt2	0.0244203	3.311592	0.909069	0.9892716
Gm6252	-0.0696755	-0.4253093	0.9091511	0.9892918
Myadml2	-0.0735683	-0.4750387	0.9093808	0.9893825
Pitpnm3	0.0659974	1.5094983	0.9093866	0.9893825
Ica1l	0.0838271	-0.5986478	0.9094253	0.9893825
Pde6d	0.0158981	4.6614955	0.9095926	0.9894952
Gm12166	0.0542659	1.5477188	0.9096943	0.9895367
Msn	-0.0141428	9.2534006	0.9099315	0.9897255
Gm18872	0.052436	0.6069691	0.9101611	0.9899011
Fzd9	0.098138	-0.5419482	0.910283	0.9899011
Pick1	0.0294796	2.5509804	0.9103384	0.9899011
Bclaf1	-0.0133401	7.9627106	0.9104932	0.9899011
Zc3h11a	-0.0124185	7.1713223	0.9104948	0.9899011
Evi5	-0.0177873	6.2022687	0.9107188	0.9899011
Arhgap11a	0.0146355	7.2179921	0.9107903	0.9899011
Wnt5b	0.0485031	1.7212963	0.9108509	0.9899011
Dip2c	-0.025335	3.4306369	0.9110513	0.9899011
Setmar	0.0510169	1.2529446	0.9112233	0.9899011
Ktn1	-0.0132641	5.6760961	0.9112427	0.9899011
Wdr92	0.0201457	3.8663367	0.9113399	0.9899011
Gm26533	0.1101403	-0.9427638	0.9113674	0.9899011
Zbtb11os1	-0.0399669	1.9400303	0.911435	0.9899011
Dna2	0.0165586	6.0982789	0.9114602	0.9899011
Mfap1a	0.0166363	4.980655	0.9114845	0.9899011

Irgq	0.0276037	4.0112809	0.9115131	0.9899011
Pmp22	0.0248065	3.4798155	0.9115322	0.9899011
Tmub2	0.0144715	5.2383223	0.9115609	0.9899011
Tyk2	-0.0139266	5.3129264	0.9117228	0.9899011
Slc41a2	0.0311734	3.1480008	0.91183	0.9899011
Ints2	-0.0135156	5.0428708	0.9118666	0.9899011
Lpar5	0.0336472	3.0572785	0.9119569	0.9899011
Lgals8	0.0153307	5.4098203	0.9119712	0.9899011
Zfp950	0.0227089	4.6458347	0.9120384	0.9899011
Pex13	-0.0153174	4.6356747	0.9120409	0.9899011
Gm41688	-0.0723902	-0.3966485	0.9120812	0.9899011
Xpo6	0.013605	6.3083497	0.912097	0.9899011
Prdm10	-0.01427	4.8499085	0.9121263	0.9899011
Nutf2-ps1	-0.0688556	-0.6519055	0.9122157	0.9899011
Prpf4b	0.0152137	7.7122804	0.9122334	0.9899011
Igkv4-57	0.0399235	2.3151732	0.9122598	0.9899011
D130007C19Rik	-0.0607411	-0.5715087	0.9122866	0.9899011
AI182371	0.1007849	0.0163954	0.9123056	0.9899011
Mapk1	0.014036	8.0503972	0.9123595	0.9899011
Adcy4	-0.0307236	2.4824569	0.9124098	0.9899011
Gpld1	-0.0414752	0.8323086	0.9124774	0.9899011
Phtf1os	-0.0290208	1.9786026	0.9126388	0.9899011
2900009J06Rik	0.1508949	-1.17195	0.9126855	0.9899011
Ppp6c	0.0128746	6.6957179	0.9127161	0.9899011
Ccnj	-0.0199078	4.0699056	0.9127538	0.9899011
Stab1	0.0207994	5.3554702	0.9128022	0.9899011
Zc4h2	0.0416321	1.8431967	0.9128296	0.9899011
Efna5	-0.0383903	1.6949984	0.9129333	0.9899445
Safb2	0.0161613	6.8867561	0.9130188	0.9899683
Gm17586	0.028707	2.3138076	0.9131881	0.9900828
Gm12696	0.0509081	0.0998558	0.9133766	0.9902181
Atg2b	-0.012666	5.6509222	0.9134732	0.990244
Cdk5rap3	0.0189538	5.0718925	0.9135278	0.990244
Gm38592	-0.0356255	1.8979263	0.9137209	0.9903386
Col12a1	-0.0335134	6.5586035	0.9137424	0.9903386
Arnt2	-0.0610623	0.488708	0.9140088	0.9904506
Map2k7	-0.012894	5.6931598	0.9140313	0.9904506
3830406C13Rik	0.0197602	4.7010801	0.9140368	0.9904506
Sac3d1	0.0169907	3.8576866	0.9141375	0.9904523
Bmp2	-0.0293919	2.8250989	0.9142001	0.9904523
Fgfr1	-0.0315075	6.0869506	0.9142294	0.9904523
Gm11914	0.0344836	2.3135949	0.9144281	0.9905986
Aktip	-0.0164863	4.2198082	0.9145382	0.9906489

Actb	-0.0158105	12.188603	0.9148632	0.9906793
Rtl8c	-0.027867	2.4286612	0.9149147	0.9906793
Sh3bp4	0.0345244	2.4346719	0.9150071	0.9906793
Atp5k	-0.0151915	6.9424026	0.9150448	0.9906793
Pde1b	0.0265089	4.2002834	0.9150532	0.9906793
P4hb	-0.0122677	8.9242812	0.9150754	0.9906793
Gm2065	-0.0611725	0.0978568	0.915325	0.9906793
H2az1	-0.0149584	9.3097864	0.915332	0.9906793
Zfp277	0.0232645	3.7549099	0.9154343	0.9906793
Ppih	0.0189656	5.0800112	0.9154514	0.9906793
Smap2	0.0147865	7.1402325	0.9155119	0.9906793
Gm19378	-0.045339	1.1085652	0.9155382	0.9906793
Cd55	0.0197807	6.4781131	0.9155886	0.9906793
Lcorl	-0.0171666	4.4136593	0.915609	0.9906793
Wbp11	0.0172959	6.7255055	0.9156219	0.9906793
Phldb3	0.0352634	3.0303939	0.9156487	0.9906793
Sgca	-0.0671041	0.4819756	0.9156599	0.9906793
Pcdhb7	-0.0645091	-0.0449294	0.9157127	0.9906793
Batf2	-0.0757296	-0.8758755	0.9158638	0.9907738
Lzic	0.0186794	4.0569598	0.9160381	0.9908223
Ccdc85c	-0.0333657	1.294152	0.916122	0.9908223
Bcl6b	0.0414492	2.357722	0.916196	0.9908223
Pole4	-0.0135733	5.7714008	0.9162107	0.9908223
Dis3	-0.0182393	5.5767061	0.9162272	0.9908223
Gm20036	0.0837912	-0.3591566	0.9162915	0.990823
P3h4	0.0455436	3.9044337	0.9163918	0.9908626
Yif1a	0.0183047	3.8893554	0.9164662	0.9908741
Ccdc194	-0.0448625	3.8311653	0.9165907	0.9908949
Zfp512b	-0.0144654	4.2291995	0.9166567	0.9908949
Magi3	0.0217052	3.8364505	0.9167692	0.9908949
Hikeshi	0.0165117	4.2675739	0.9167907	0.9908949
Pef1	-0.0143618	5.284014	0.9170719	0.9908949
Cyp20a1	-0.0162448	4.3186318	0.9171172	0.9908949
Ogfr	0.0128239	5.6743708	0.9174937	0.9908949
Gamt	-0.036894	1.8616848	0.9175003	0.9908949
Mab21l2	-0.0504498	1.7260522	0.9176197	0.9908949
Rpl39l	0.0449314	1.6544058	0.9176415	0.9908949
Slc6a3	0.0377716	1.4546377	0.9177111	0.9908949
Slc45a4	0.015339	5.4117933	0.9177176	0.9908949
Amy1	-0.0261584	2.7466546	0.9177933	0.9908949
Arhgap42	-0.0363247	2.8780361	0.9178012	0.9908949
E430018J23Rik	-0.0394069	0.852943	0.9178208	0.9908949
Ppp2ca	-0.0108904	8.115948	0.9178243	0.9908949

Cdc42bpg	-0.0151504	4.0214687	0.917878	0.9908949
Spdl1	0.0167565	5.0232756	0.9179851	0.9908949
Selenot	-0.011555	7.8239563	0.9180507	0.9908949
Fbxo36	-0.0360352	1.4716755	0.9182127	0.9908949
Zfp110	0.0127197	5.2897861	0.9182238	0.9908949
Trappc5	-0.0124618	4.9545147	0.9182289	0.9908949
Rap2b	0.0136854	5.5391055	0.9182407	0.9908949
Fggy	-0.0387582	1.3872014	0.9182568	0.9908949
Scarf1	-0.0200189	3.3899375	0.9182606	0.9908949
Aifm1	0.013135	5.5936419	0.9182769	0.9908949
Rbm12b2	0.0198121	4.5146095	0.9183012	0.9908949
Gm14057	-0.0517386	0.2649853	0.9183496	0.9908949
Atmin	0.0156957	4.445252	0.9184298	0.9908949
Trim8	0.0116601	6.1405536	0.9184372	0.9908949
Ppid	0.0142866	6.5077227	0.9185184	0.9908949
Glt8d2	-0.1141303	-0.68921	0.918524	0.9908949
Ammecr1l	0.0163063	5.4944052	0.9186089	0.9909177
Zfp51	-0.0167387	4.0444083	0.9187467	0.9909977
Kctd2	0.0209367	4.3700561	0.9188302	0.991019
Fyttd1	-0.0126395	5.2234695	0.9188982	0.9910236
Abhd14a	0.0338789	2.3506311	0.9190295	0.9910774
Ube2j1	-0.0117521	6.5597582	0.9190755	0.9910774
Trim58	0.0292178	4.9531146	0.9192911	0.9912245
Neo1	0.0331809	4.8550025	0.9193917	0.9912245
Tmem19	-0.0139346	4.983765	0.9194089	0.9912245
Slc6a20a	0.030312	3.1514214	0.919505	0.9912245
A730017L22Rik	0.0498989	0.7045447	0.9195305	0.9912245
Dcun1d5	0.0117546	6.0028818	0.9195987	0.9912293
Bud13	-0.0221886	3.7699446	0.9197076	0.9912299
Ccdc130	-0.0191103	3.2686022	0.9197468	0.9912299
Zbtb10	0.0301183	3.2844001	0.919826	0.9912299
Sdr39u1	0.0299325	1.9633647	0.919943	0.9912299
Etohd2	-0.031159	1.0970672	0.9201492	0.9912299
Ocstamp	-0.0428843	2.6558365	0.9201693	0.9912299
Atp2a2	0.0137445	7.7774531	0.9201779	0.9912299
Matn1	0.1220377	0.2091684	0.9203075	0.9912299
Smo	0.0160903	4.8104082	0.9203546	0.9912299
Adat1	-0.0313749	2.3361958	0.9203637	0.9912299
Ddx50	0.0129794	6.2433468	0.9204115	0.9912299
Xkr8	-0.0264305	2.4558276	0.9204695	0.9912299
Ttc39c	-0.0292948	2.5615668	0.9204722	0.9912299
Pir	0.0283616	2.6669305	0.9204961	0.9912299
Map4k5	0.0173255	6.995088	0.9205552	0.9912299

Mosmo	0.014447	4.6518408	0.9206542	0.9912678
Cic	0.0151974	6.950536	0.9207594	0.9913125
Aldh1l2	0.042192	3.7887743	0.9211112	0.9916227
Gm29674	0.0727547	-0.0615714	0.9211763	0.991624
Arhgef15	0.0366612	2.1747442	0.9216137	0.9919309
Ndufv1	-0.0128374	6.1035539	0.9216626	0.9919309
Serp1	-0.0115916	8.2023531	0.9217229	0.9919309
Atxn7l3b	-0.0100896	7.0325546	0.9217988	0.9919309
Map11	-0.0149833	6.1560022	0.9218955	0.9919309
Supt4a	0.0124108	5.9272371	0.9219061	0.9919309
Decr2	-0.0217506	2.8501894	0.9219563	0.9919309
Gm4890	0.0852044	-0.2014329	0.9219803	0.9919309
Rps27l	0.0206046	6.7298548	0.9220412	0.9919309
Pdhx	-0.0131591	4.823942	0.9220991	0.9919309
Anxa7	0.0108098	6.5230883	0.9222033	0.9919744
Sh2d4b	0.0311884	2.3801452	0.9223965	0.9920905
Tspyl4	0.0356976	2.1862077	0.9224388	0.9920905
Dicer1	0.0144537	6.1030662	0.9227359	0.9921355
Dld	-0.0118106	6.9710116	0.9227809	0.9921355
Cenpo	0.0173108	4.3499432	0.922791	0.9921355
Ogt	0.0135342	8.1553674	0.9228125	0.9921355
Thap2	-0.0176383	4.4913971	0.922846	0.9921355
Nmb	0.0383721	2.464355	0.9228633	0.9921355
Poldip2	0.0122345	5.1921129	0.9229835	0.9921531
Zc3h12b	-0.0445728	0.4731662	0.9231307	0.9921531
Efhc1	-0.0371824	0.2158362	0.923132	0.9921531
H2-Q6	-0.0339229	1.4606731	0.9231873	0.9921531
Ptch1	-0.0143902	4.7754056	0.9231986	0.9921531
Ccdc114	-0.0445913	1.047865	0.9232808	0.9921728
Nim1k	0.045955	0.3486595	0.9235295	0.9922956
Fbxo45	-0.0146759	5.0835796	0.923577	0.9922956
7530428D23Rik	0.0684188	0.1462189	0.9235865	0.9922956
Zfp964	0.0543282	-0.2412979	0.9236588	0.9923048
4930520O04Rik	-0.0504396	-0.0648757	0.9238023	0.9923078
Sema6b	0.0267683	3.4576324	0.9238482	0.9923078
Sde2	0.0140584	5.6250371	0.9238979	0.9923078
Synpo	-0.0255511	3.7988853	0.9239168	0.9923078
Hps4	0.0196188	3.7964139	0.9240776	0.992412
Zscan30	-0.0334331	1.3257924	0.924296	0.992578
Pigm	0.0124679	5.7525455	0.9244684	0.9926946
D5Ertd579e	0.0133559	6.459649	0.9248441	0.9929058
Catsperg1	-0.0623715	-0.8794136	0.9249465	0.9929058
Mmp23	-0.0297596	3.0863849	0.9250581	0.9929058

Mxd4	-0.020656	4.9878844	0.9251061	0.9929058
Igfbp7	0.0225969	6.4249399	0.9252046	0.9929058
Lyplal1	0.0377181	0.2572008	0.9252576	0.9929058
Gm46093	-0.0561249	-0.1160707	0.9252583	0.9929058
Rars2	0.0165263	4.4252648	0.9252886	0.9929058
Trp53bp2	0.0162059	4.1455501	0.9253249	0.9929058
Nelfe	0.0122671	4.9929018	0.9253424	0.9929058
Sfxn4	-0.0384229	1.0799183	0.9255631	0.9929058
Mtrf1l	0.0171848	4.1258591	0.9255758	0.9929058
Pdcl	-0.0114544	5.6112718	0.9255896	0.9929058
Eif5b	-0.0121187	6.9089706	0.9257116	0.9929058
Zfp273	0.0321433	2.1673735	0.9257416	0.9929058
Fbrs	0.014755	6.4540259	0.9258158	0.9929058
Ppp1r9a	-0.0208963	2.6181692	0.9258471	0.9929058
Btg3	-0.0219118	3.4675093	0.9258991	0.9929058
Nop10	-0.0127377	6.0751417	0.9259017	0.9929058
Prrc2a	0.0141878	8.1581897	0.9259773	0.9929058
Dcbld2	-0.0226439	3.3284524	0.9260113	0.9929058
Mc2r	-0.0768347	-0.7600773	0.9260747	0.9929058
Dusp1	-0.0271871	5.2943984	0.9261333	0.9929058
Tm2d2	0.014528	4.4983268	0.9264401	0.9931662
Hhat	-0.0707723	0.0568568	0.9265686	0.9932356
Psmd14	-0.0101367	6.4866512	0.92694	0.9935513
Gm16008	0.0964035	-0.3093896	0.9269909	0.9935513
Rabl2	0.027088	2.062083	0.9271468	0.993629
Nup133	-0.0122703	5.4862833	0.9272161	0.993629
Ubald2	-0.0105655	6.0461444	0.9272551	0.993629
C9orf72	-0.0185421	3.9637943	0.9273685	0.9936821
Plgrkt	-0.0160457	4.4441585	0.9275098	0.9936871
Kif5a	-0.0362718	0.2768786	0.9275559	0.9936871
Syk	0.0207263	8.4331609	0.9275648	0.9936871
Ddt	-0.021552	3.2903153	0.9276359	0.9936948
Mindy3	-0.0120674	4.7400406	0.9278521	0.9938579
Lipe	0.0208342	4.39961	0.9279696	0.9938979
Rhobtb2	0.021648	4.1987966	0.9280171	0.9938979
Zfp169	-0.012824	3.9601477	0.9282084	0.9939283
Skp1	0.0113655	7.0400854	0.928269	0.9939283
Gm6483	-0.034833	0.6356667	0.9282972	0.9939283
Gm32510	0.0281568	2.3617537	0.9283012	0.9939283
Zfp60	-0.0167543	3.7873128	0.928504	0.9939309
Oit3	0.0548646	-0.0875657	0.9285113	0.9939309
Fanci	-0.0131091	5.0951899	0.9285895	0.9939309
Stil	0.0157665	5.3855168	0.9286729	0.9939309

Zfp74	-0.0161552	3.1278032	0.9286958	0.9939309
Gm38560	0.1530433	-1.2184556	0.9287126	0.9939309
Kcnma1	-0.035624	3.5319321	0.9287509	0.9939309
Dhx36	0.0116607	6.0412677	0.9288554	0.9939743
Mrpl55	0.016465	3.6494056	0.9289217	0.9939769
Fads6	-0.0208152	1.6881357	0.9290517	0.9940476
Rad51c	-0.0165724	3.3957355	0.9292955	0.9941477
Tmem87a	-0.0104874	5.716978	0.9293085	0.9941477
Gm30445	-0.0288608	1.244785	0.929337	0.9941477
Muc20	0.0739198	0.1569149	0.9295979	0.9941542
Plbd2	0.0162783	4.7359866	0.9297605	0.9941542
Cnot7	-0.0095702	6.1802234	0.929919	0.9941542
Eml3	-0.0105228	5.4661664	0.9299556	0.9941542
Smc5	0.0119524	5.9851095	0.930028	0.9941542
Cinp	0.0119396	4.7496119	0.9300417	0.9941542
Plxdc1	-0.0202962	2.52267	0.9300873	0.9941542
Marf1	-0.0105902	6.4359994	0.9301128	0.9941542
Il10ra	0.0180792	5.0338468	0.9301258	0.9941542
Gm43464	-0.091022	-0.880938	0.9301307	0.9941542
Hoxa4	-0.056923	-0.0059664	0.9301486	0.9941542
Celf3	-0.0737769	-0.8689946	0.9303471	0.9941542
Gm36411	0.0679942	-0.4228483	0.9304015	0.9941542
Gm36462	-0.0409189	1.0034213	0.9304231	0.9941542
Ncapd3	-0.0119084	7.1404267	0.9305285	0.9941542
Gns	-0.0115903	6.9888192	0.930756	0.9941542
Hadhb	0.011592	6.3713693	0.93077	0.9941542
Depdc1b	0.0127545	5.749483	0.9308292	0.9941542
Svep1	-0.0213306	4.0216946	0.9308503	0.9941542
Pfkfb3	-0.0129329	6.0526763	0.9309616	0.9941542
Vps26c	-0.0107186	4.9877719	0.9310778	0.9941542
Exog	-0.0184928	3.1735232	0.9310779	0.9941542
Rad17	-0.0097517	5.4342271	0.9311264	0.9941542
Zfp948	-0.0185161	3.0652094	0.9313172	0.9941542
Tcrb	0.0291997	3.6855529	0.9313233	0.9941542
Prr11	0.012048	6.0930442	0.931427	0.9941542
Cand2	-0.02142	1.4803726	0.9314412	0.9941542
Tas1r1	-0.043017	-0.6853777	0.9314728	0.9941542
Cmc1	-0.0152092	3.7385587	0.9314823	0.9941542
Hgs	-0.0121208	5.2741414	0.9315529	0.9941542
Abcc10	0.0245926	2.2571272	0.9316449	0.9941542
Pgm3	0.0209695	3.6293541	0.9316491	0.9941542
Zfp607a	-0.0244114	2.6015864	0.9316586	0.9941542
Cited4	-0.0154577	4.6175424	0.931715	0.9941542

Zfp937	-0.0325194	0.8752673	0.931724	0.9941542
Gm5814	0.0439872	-0.0197796	0.9317464	0.9941542
Dusp23	0.023682	2.7172845	0.9318447	0.9941542
Usp33	-0.0092711	6.3874377	0.9318899	0.9941542
Relt	0.0111471	5.2398705	0.9318917	0.9941542
Slc16a14	-0.0593678	-0.4278964	0.9318997	0.9941542
Gnl3l	0.011284	5.5240216	0.9319679	0.9941588
Tmem41b	-0.0141682	4.7872125	0.9320449	0.9941727
Ms4a14	0.0402167	0.7210527	0.9321845	0.9942535
Airn	-0.0531663	0.5668957	0.9322577	0.9942588
Prrg3	0.0911276	-0.7468216	0.9323174	0.9942588
B3galnt1	0.0265901	2.0070469	0.9324276	0.9943081
Trgc1	-0.0735048	-0.8172751	0.9326298	0.9944342
E430024I08Rik	-0.0132584	3.4798017	0.9326737	0.9944342
Spata1	0.0352728	1.0890421	0.9328408	0.9945442
Akr7a5	0.0191407	2.9579094	0.9330637	0.9947137
Gm14567	0.0737968	-0.8832727	0.9335569	0.9951713
Ccdc102a	-0.0413717	1.4880808	0.9338506	0.9954161
Use1	0.0121174	5.8622144	0.9340171	0.9954727
Gon4l	0.0123695	5.4010701	0.9340316	0.9954727
Abitram	0.014685	4.2716404	0.9341413	0.9954873
Cmtm3	-0.0117307	4.7378191	0.934185	0.9954873
Gm6212	0.0526159	0.4446951	0.9342374	0.9954873
Kcnc1	-0.0485015	-0.2370728	0.9344061	0.9955989
Fabp7	0.0648802	0.3565925	0.9344946	0.9956219
Boc	-0.0235066	3.055459	0.9346181	0.9956219
Psmb9	0.0148941	4.8719207	0.9346197	0.9956219
Ube2e1	-0.0097412	5.6556155	0.9347795	0.9956262
Gm10785	-0.048295	0.3602463	0.9348813	0.9956262
Gm41173	-0.0655321	-0.5109611	0.93493	0.9956262
Adam10	0.0104493	8.7315155	0.9350284	0.9956262
Lsm12	-0.010802	5.7749643	0.9350987	0.9956262
Tlr3	-0.0261309	2.0919417	0.9351031	0.9956262
Acad12	0.0365494	0.4989515	0.9351546	0.9956262
Ercc6	0.015856	4.7850813	0.93535	0.9956262
Utp14a	0.0111054	5.5326642	0.9354543	0.9956262
Ninj1	-0.0108673	4.9044631	0.9354547	0.9956262
Smg9	-0.0107558	4.898068	0.9354591	0.9956262
Phlpp1	-0.0148621	3.5054594	0.935682	0.9956262
Zfp526	-0.0168471	4.8773055	0.9357065	0.9956262
9530018F02Rik	-0.0365755	0.8698543	0.935786	0.9956262
Casp9	0.0107561	4.9209431	0.9359977	0.9956262
Srd5a1	0.0239182	1.5988686	0.9360352	0.9956262

Sympk	0.0117142	6.2739181	0.936052	0.9956262
Chordc1	0.0098601	6.2894629	0.9360795	0.9956262
Map3k19	-0.0440697	0.4280793	0.9361185	0.9956262
Slc25a35	-0.0176617	1.9387372	0.9361191	0.9956262
Spats2	0.0294437	2.3538978	0.9362278	0.9956262
Cndp2	0.0130041	5.5849407	0.9362751	0.9956262
Eloc	-0.0125384	6.5225986	0.936288	0.9956262
Cnot6l	0.0135054	6.5177957	0.9363395	0.9956262
Rab22a	-0.0102468	5.0835681	0.9363421	0.9956262
Spats2l	-0.0441648	0.9942135	0.9364253	0.9956262
E2f3	0.0111705	5.2050312	0.9364924	0.9956262
Flt4	-0.0118865	5.042271	0.9365996	0.9956262
Btbd6	-0.0218619	2.5136419	0.9368285	0.9956262
A930015D03Rik	-0.0596139	-0.6927364	0.9369033	0.9956262
Ccr2	0.0143904	7.5867097	0.9369406	0.9956262
Ifi207	-0.017005	4.5709256	0.9369833	0.9956262
Zdhhc13	-0.010185	4.9592448	0.9369977	0.9956262
Brd2	0.0092757	7.7697782	0.9370127	0.9956262
Sra1	0.014004	4.6662706	0.9370457	0.9956262
Parp12	-0.015329	4.2133971	0.9371517	0.9956262
AW554918	-0.0136882	3.3892207	0.9372673	0.9956262
Slc25a44	-0.0116559	5.5742658	0.937319	0.9956262
Ahi1	0.0167908	4.1860076	0.9374872	0.9956262
Klhl29	0.0594313	-0.2237061	0.937492	0.9956262
Lbx2	-0.1026636	-1.0656615	0.9375453	0.9956262
Pus7	0.0123968	4.6734502	0.9375728	0.9956262
Myo18a	-0.0088115	6.3378364	0.9376515	0.9956262
Rab4b	0.0114412	5.120102	0.9377407	0.9956262
Kcnd1	0.0284805	1.461742	0.9377609	0.9956262
Zfp189	-0.0175429	2.9577929	0.9378424	0.9956262
Arpp21	-0.0203749	2.3587719	0.9378946	0.9956262
Eif2s2	0.0133004	7.5070685	0.9379512	0.9956262
Dpep2	-0.0305391	0.8161703	0.9379909	0.9956262
Fos	-0.0279376	3.9765056	0.9380772	0.9956262
Gm12657	0.0548425	-0.6847147	0.9381123	0.9956262
Gm15796	0.1229821	-0.9100092	0.9381491	0.9956262
Letm2	-0.0156553	2.3276952	0.9383449	0.9956262
Rtl8a	-0.0275308	1.563278	0.9383559	0.9956262
Rpl39-ps	0.0241315	1.8690768	0.9383614	0.9956262
Ifi27	0.017013	4.9677706	0.938363	0.9956262
Mrpl16	0.0156315	4.5455134	0.9384409	0.9956262
Ptges	0.0309357	1.6208111	0.9384538	0.9956262
Ncstn	0.0109504	6.1840099	0.9384553	0.9956262

Haus5	-0.0100677	4.92132	0.938574	0.9956262
Hnrnpul2	0.0100606	8.7575004	0.9385827	0.9956262
LOC115486436	0.0729628	-0.0946508	0.9385925	0.9956262
Prkaca	0.0094337	6.0257768	0.9386855	0.995657
Clptm1l	-0.0084586	6.4011595	0.9388408	0.9956715
BC051142	0.0615353	0.3820782	0.9389153	0.9956715
Gvin-ps1	-0.0629714	0.9000802	0.9389426	0.9956715
Zfp39	-0.0170702	4.5506779	0.9389552	0.9956715
Rab11fip5	-0.012335	3.7076143	0.9391068	0.9957046
Parp11	-0.0101044	4.2263614	0.9391145	0.9957046
Aar2	-0.0134078	3.7912664	0.9393145	0.9957472
Rab11fip2	0.0202429	3.3008007	0.9394019	0.9957472
Kcnh2	-0.0466664	0.4563818	0.9395109	0.9957472
Calr3	-0.1306085	-1.090861	0.9395672	0.9957472
Lrrc56	0.0301045	2.0012725	0.9395931	0.9957472
Tmtc3	-0.0110511	4.8355297	0.9396242	0.9957472
Gm46183	-0.031357	0.1586073	0.9396451	0.9957472
Mmp14	-0.0173331	7.0673094	0.9396668	0.9957472
Zfp442	-0.0328051	1.3233432	0.9397512	0.9957688
Il20ra	-0.0887623	-0.8338893	0.9399157	0.9958608
Gm38708	0.042063	0.4219554	0.9399661	0.9958608
Glmp	0.010412	5.5093423	0.9401617	0.9959904
Gpihbp1	0.0196216	2.4852871	0.9402165	0.9959904
Top1mt	0.0212501	2.1137137	0.9402812	0.9959911
Bckdha	0.0142869	4.8158787	0.940357	0.9960036
Gm13707	0.0408131	0.5147785	0.9404922	0.996079
Gm19951	0.0877643	1.1046671	0.9405811	0.9960842
Pacsin3	0.0401267	0.923683	0.9406252	0.9960842
Gm46801	-0.0336023	-0.51195	0.9407418	0.9961398
Triap1	-0.01073	4.4592654	0.9408152	0.9961497
Zfp455	0.0488654	0.9515546	0.9409388	0.9961529
Gm40448	-0.0591147	-0.5935705	0.9409501	0.9961529
Arl6ip1	0.0093645	8.3964304	0.9410103	0.9961529
Gatd1	-0.0122723	3.7542039	0.9411472	0.996212
Eps8l2	0.0470596	0.9823232	0.9412475	0.996212
Kcnk1	-0.0296628	3.1233027	0.9414493	0.996212
Tent4a	0.0113826	4.7978454	0.9414583	0.996212
Ube2g1	-0.0089687	5.6519065	0.9414852	0.996212
A930033H14Rik	-0.0278887	1.0395515	0.9414888	0.996212
Ltb	-0.0171882	5.1816086	0.9416775	0.996212
Mill2	-0.0426345	0.9656681	0.94173	0.996212
Pde4b	0.0163889	4.6053874	0.9417424	0.996212
Atg101	-0.0124375	4.682925	0.9418403	0.996212

Zfp35	0.0122479	5.2007948	0.94191	0.996212
Usp51	-0.0835782	-0.8301576	0.9419351	0.996212
Hoxb7	0.0489682	-0.2333014	0.9419454	0.996212
Armt1	0.0107057	4.7552386	0.9420136	0.996212
E130311K13Rik	0.0305769	1.4094159	0.9420269	0.996212
Gapvd1	0.0089032	7.3456827	0.9422934	0.9964261
Cenpp	0.0158998	4.3464987	0.9423739	0.9964435
Naa16	-0.0092332	4.9362674	0.9427007	0.9967212
AI837181	-0.0100093	4.609695	0.9429552	0.9968473
Cd27	0.0245758	2.2240764	0.9429703	0.9968473
Ipo8	0.0093517	5.4869564	0.943065	0.9968473
Zfp970	0.023888	3.0115911	0.9430762	0.9968473
Gfus	-0.0107884	4.1353239	0.9433107	0.9969841
Ino80	0.0142309	5.1852135	0.9433338	0.9969841
Nid2	-0.0151583	3.6847511	0.943433	0.9970211
Ost4	0.0106557	6.954276	0.9435731	0.9971014
Ntn4	0.0274832	2.1409147	0.9437094	0.9971777
Tubgcp4	0.0079678	5.5045122	0.9438457	0.9971869
Dse	0.0121158	4.639412	0.9438974	0.9971869
Mfsd4b3-ps	-0.0843277	-0.9619772	0.9439104	0.9971869
Snhg17	-0.0187218	2.6117127	0.9441552	0.9973156
Hdac5	0.0137532	5.8125319	0.9442346	0.9973156
Abhd4	-0.009117	5.1258784	0.9443561	0.9973156
Erlin2	-0.0075983	5.2726717	0.9443693	0.9973156
Cep55	0.0138259	5.195882	0.9443829	0.9973156
LOC118567382	-0.054919	-0.5112569	0.9444169	0.9973156
Yap1	0.0201746	4.1752103	0.944623	0.9973763
Fastkd1	-0.0128613	2.9652531	0.9446406	0.9973763
Kif4	0.010818	6.6162682	0.9446917	0.9973763
Smad9	-0.0315998	1.7299468	0.9447736	0.9973763
Gm4928	-0.0722829	-1.2234775	0.9448235	0.9973763
Zfp386	0.0136033	4.4163535	0.9448592	0.9973763
Plek2	-0.0253096	3.5181847	0.9449732	0.9973831
Cdc42ep1	-0.0156807	3.6610355	0.9449938	0.9973831
Ak5	-0.0245871	2.4170877	0.9457335	0.9979134
Gm35498	-0.0193749	1.7702403	0.9459999	0.9979134
Gna15	0.0127389	4.2457355	0.9460255	0.9979134
Colec12	-0.0130209	5.5035488	0.9462289	0.9979134
Ormdl2	-0.0108021	4.9439467	0.9462943	0.9979134
Dmkn	-0.0224297	2.6636938	0.9463124	0.9979134
Cdhr4	0.0840804	-0.6118893	0.9464258	0.9979134
Tmem88b	-0.0275402	-0.2656081	0.9465432	0.9979134
S100b	0.0227807	3.0516191	0.9465463	0.9979134

Tti2	0.0124816	3.6185883	0.9466059	0.9979134
Cbfb	0.0101129	7.1340112	0.9466922	0.9979134
Filip1l	-0.0115466	5.6267042	0.9467947	0.9979134
Gask1b	-0.0179567	5.4458615	0.9468361	0.9979134
Trappc6b	0.008312	5.6670416	0.9468756	0.9979134
Ppa1	0.009762	5.1279574	0.9468869	0.9979134
Ankmy2	-0.0095601	4.0554364	0.9470743	0.9979134
Gabrb2	0.0589298	0.1228529	0.9471358	0.9979134
Sorbs1	0.0123765	5.3477245	0.9472724	0.9979134
Naaladl1	-0.0400572	0.8712459	0.9473704	0.9979134
Elovl4	-0.0518489	-0.1223535	0.9474701	0.9979134
Brd9	0.0080381	5.7236384	0.9474876	0.9979134
Sertad1	-0.014681	3.7233211	0.9475053	0.9979134
Clk1	0.0117277	7.9299409	0.9475164	0.9979134
Adgrl1	0.0131497	4.3081633	0.9475291	0.9979134
Commd9	0.0109165	3.4905846	0.9475413	0.9979134
Arid1b	-0.0108873	6.3033199	0.9475681	0.9979134
Slc25a11	0.0077062	5.7051823	0.9475892	0.9979134
Gm29666	0.0745733	-1.0415202	0.9476515	0.9979134
Cyp2r1	-0.0650124	-0.3790647	0.9476911	0.9979134
Twsg1	0.007847	5.6216218	0.9477012	0.9979134
Dchs2	0.0284948	0.2546102	0.9477037	0.9979134
Nr1h2	-0.0062838	6.0333301	0.9477128	0.9979134
Eva1c	-0.0582214	-0.1205137	0.947753	0.9979134
Gm2058	-0.0220838	0.9680625	0.9478141	0.9979134
Psma5	-0.0089183	6.1297765	0.9478786	0.9979134
Klhdc9	0.1127952	-1.1395273	0.9478834	0.9979134
Pi4k2a	0.009844	4.7223058	0.9480902	0.9979134
Izumo4	0.0204276	2.2876083	0.9480931	0.9979134
Prdx6	0.0076669	6.8033771	0.9481062	0.9979134
Tprgl	-0.00743	6.7812353	0.9481535	0.9979134
Tead1	-0.0214697	3.0802499	0.9482501	0.9979134
Ykt6	0.0085083	6.3135851	0.9482795	0.9979134
Zfp760	0.0117298	4.3438226	0.9482898	0.9979134
Gm9320	-0.0636323	-0.7425055	0.9483597	0.9979134
Fam102a	-0.0154268	5.4661034	0.9483835	0.9979134
Pcgf3	0.0087911	5.5789972	0.9486787	0.9979405
Cpt1a	0.016249	7.1204782	0.9487469	0.9979405
LOC118568733	0.0895658	-0.8921753	0.9487476	0.9979405
Lpar3	-0.0271144	2.0924175	0.9487482	0.9979405
Gm13160	-0.0871035	-1.1476872	0.9488258	0.9979405
Eif4a1	0.0089695	8.9925856	0.948855	0.9979405
Pcbp4	0.02945	2.1544972	0.9489277	0.9979405

Phka1	-0.0111079	3.6280973	0.948956	0.9979405
Stag1	0.0074509	6.8219907	0.9489866	0.9979405
Gm34872	-0.0796027	-0.969472	0.9490798	0.997971
Mgrn1	-0.0070631	5.4153499	0.9492648	0.9980517
Rnf4	0.0073552	6.8235763	0.9493651	0.9980517
Gm52422	0.0611224	-0.3992172	0.9493831	0.9980517
Ly6g5b	-0.0167914	2.8762385	0.9496554	0.9980517
Tspan7	-0.0131819	4.3026741	0.9496715	0.9980517
Gbgt1	-0.037567	0.6856935	0.9496741	0.9980517
Rpl31	0.0109353	9.5344775	0.949851	0.9980517
Slc1a3	-0.0158062	2.8577805	0.9498617	0.9980517
Bcl2l13	0.010964	6.5069997	0.9499196	0.9980517
Gpc4	0.0117094	4.7222144	0.9499653	0.9980517
Fam13c	0.0296643	1.5794315	0.9500075	0.9980517
Cyp2b10	0.0317362	0.720259	0.9500667	0.9980517
St6galnac4	-0.0106168	4.659831	0.9500805	0.9980517
Dedd	-0.0085143	4.8620465	0.9501132	0.9980517
Zmat3	0.0122861	2.8688012	0.9501297	0.9980517
Hectd4	0.0129155	7.7629752	0.9501832	0.9980517
Sergef	-0.0184985	1.9473546	0.9503347	0.9980857
Cnnm2	-0.0114323	4.6676521	0.9503554	0.9980857
Nkiras2	-0.0095975	4.8060661	0.9504081	0.9980857
B4galnt1	-0.008146	6.552428	0.9505708	0.9981892
Ppfia1	-0.0075262	5.9689015	0.9506615	0.9982171
Acss3	-0.0282707	1.7667061	0.95077	0.9982635
Grm6	0.037469	-0.8848789	0.9509166	0.998314
Acss2	-0.011658	4.1369772	0.9509464	0.998314
Gm5921	0.0811655	-0.9578931	0.9511627	0.9984737
Ttc19	-0.0139582	2.9522707	0.9517022	0.9986777
Rcor1	-0.0088634	6.4311773	0.951715	0.9986777
A430033K04Rik	0.0224384	2.6928937	0.9518084	0.9986777
Prcp	-0.0088239	6.1952029	0.951847	0.9986777
Gm12596	0.0653748	-0.3278238	0.9519177	0.9986777
Fam234a	-0.0095171	4.5144322	0.9520446	0.9986777
Zfp839	0.0212228	2.765542	0.9521019	0.9986777
Cav2	0.0142344	3.6006121	0.9521022	0.9986777
Fitm2	0.0185009	1.5673316	0.952142	0.9986777
Zfp651	0.0209182	2.7774563	0.9522149	0.9986777
Slc2a13	-0.0223346	2.6794922	0.9522273	0.9986777
Lztr1	0.0080067	4.955562	0.9524203	0.9986777
Pcnt	-0.0084109	6.5054605	0.9524587	0.9986777
Tctn3	0.0164924	2.2894537	0.9524597	0.9986777
Lman2l	-0.0079322	4.6773192	0.95263	0.9986777

Ikbip	-0.0116963	4.8240473	0.9527204	0.9986777
Phf21b	0.0909015	-0.682647	0.9527775	0.9986777
Mrnip	0.021809	2.0879413	0.9528665	0.9986777
Stk35	-0.0085199	4.671572	0.9529525	0.9986777
Tmem106c	0.0135209	3.2767153	0.9530341	0.9986777
Kmt2d	0.0094481	6.9232181	0.9530473	0.9986777
Gm20257	0.0309878	1.7558498	0.9530749	0.9986777
Il9r	0.0614257	0.3789076	0.9531049	0.9986777
Bnip3l	-0.0094791	8.7811818	0.9531128	0.9986777
Zik1	-0.0144628	2.2647518	0.9531135	0.9986777
Pcnx3	0.0075702	5.7374454	0.9531508	0.9986777
Gtf2i	0.0083381	6.8768218	0.9531973	0.9986777
LOC118568783	0.0161781	2.8749236	0.9532104	0.9986777
Eif2b2	-0.0088886	4.7909444	0.9532191	0.9986777
Gm51877	0.0524246	-0.9608197	0.9534879	0.9987268
Epc1	0.00681	6.1243947	0.9537295	0.9987268
Grap2	0.0111469	6.3298309	0.9537915	0.9987268
Fam114a2	-0.006697	5.3936044	0.9538258	0.9987268
Ubap2l	0.0085324	7.6006258	0.9539787	0.9987268
Ube2q1	0.007385	6.4030181	0.9539891	0.9987268
Trim23	0.0135513	5.0548921	0.9540056	0.9987268
Parp6	0.0108554	3.9688081	0.954068	0.9987268
Adcy7	-0.0073701	6.4275327	0.9541123	0.9987268
B3gnt2	0.0078901	6.6078967	0.9542311	0.9987268
Uprt	-0.0072306	4.4591106	0.9542319	0.9987268
Cct7	0.0068628	7.5968896	0.9542446	0.9987268
Orc4	0.0082163	5.2744521	0.9542883	0.9987268
Sesn3	-0.0153764	2.9242048	0.9543282	0.9987268
Ccm2	-0.0075845	5.7859241	0.9543894	0.9987268
Aspm	-0.0075411	7.1748938	0.9543898	0.9987268
Podxl2	0.0212813	3.1109872	0.9544194	0.9987268
Wdr7	0.0087562	5.4738256	0.9544217	0.9987268
Tent4b	-0.007184	5.9238063	0.9545567	0.9988009
Sh2d5	0.0217594	3.3033186	0.9547376	0.9988713
Tor1aip2	-0.0060699	6.8503741	0.9547848	0.9988713
Npr2	-0.01452	3.6551567	0.9548167	0.9988713
Bphl	0.0145499	3.2270908	0.9549285	0.9989077
Pfdn4	-0.0125006	4.1658722	0.9550341	0.9989077
Ifi206	-0.0243445	2.2095997	0.9553409	0.9989077
Cep295	-0.0067031	6.1669044	0.9553753	0.9989077
Tmem18	-0.0073358	4.5331415	0.9554793	0.9989077
Uckl1	0.0106776	4.4803062	0.9554882	0.9989077
Gm9531	0.0178846	2.0464857	0.9555061	0.9989077

Fut11	-0.0097162	4.4885623	0.9555409	0.9989077
Ric8b	-0.0089608	4.1909368	0.9555738	0.9989077
Gm37915	-0.0615168	-0.7627188	0.9556248	0.9989077
Lrrc41	0.0094514	4.5159007	0.9556508	0.9989077
Klhl11	0.0082335	4.1865578	0.9556694	0.9989077
Gm14388	0.0376275	-0.1687188	0.9556864	0.9989077
Plxnb3	-0.052343	-0.4719545	0.955852	0.9990137
Gm18373	0.0464061	0.3346803	0.9559618	0.9990614
Zfp623	-0.016767	2.4363636	0.9560604	0.9990972
Pik3r1	0.0076328	7.8575959	0.9561855	0.9991116
Hoxa5	-0.0149631	1.0761157	0.9563538	0.9991116
Gmpr2	-0.0082311	4.1351226	0.956393	0.9991116
Thada	-0.0103711	4.2228587	0.9564609	0.9991116
Chmp1b	-0.007427	6.0054073	0.9565693	0.9991116
Naga	0.0139587	4.7037348	0.956582	0.9991116
1110059E24Rik	0.0123158	3.2444282	0.9567642	0.9991116
Capzb	-0.0063639	8.2988876	0.9567833	0.9991116
Gpr176	-0.0390755	0.4763329	0.9568482	0.9991116
Gopc	-0.0083594	4.9733903	0.9568653	0.9991116
Gm36182	0.0349007	-1.1122524	0.9568885	0.9991116
Cd302	0.0082803	4.6811619	0.9569118	0.9991116
Zfp592	-0.0071964	6.0972858	0.9570337	0.9991116
Hnrnpdl	0.0084883	7.86476	0.9570547	0.9991116
Sar1b	0.0087965	5.1556372	0.9571438	0.9991116
Galk2	0.009867	3.8358768	0.9572072	0.9991116
Gm11353	0.0257237	0.6315374	0.957256	0.9991116
B230398E01Rik	-0.0343277	-0.6481358	0.9573022	0.9991116
R3hcc1l	0.0083703	4.9310161	0.9574602	0.9991116
Spp1	0.0202438	9.2450271	0.9574996	0.9991116
Gripap1	0.0068773	6.2557317	0.957506	0.9991116
Atpsckmt	-0.0094379	3.731293	0.9575457	0.9991116
Ddx49	-0.0065147	4.5454607	0.9575515	0.9991116
P4ha3	0.0343943	0.5244407	0.9578325	0.9992632
Atp6v1h	-0.0068517	5.5761552	0.9578571	0.9992632
Gm5525	-0.0359254	-0.5463116	0.9579202	0.9992632
Adh5	-0.0071644	5.5082272	0.9579912	0.9992632
Tmem35a	0.0556269	-0.3341794	0.9580181	0.9992632
Prss35	-0.0186636	6.1827184	0.9582439	0.9993565
Tcaf1	0.0149757	3.2121267	0.9583482	0.9993565
Jkamp	-0.0084864	4.2544866	0.958405	0.9993565
Crkl	-0.0063493	6.2057938	0.9586445	0.9993565
Pkp2	-0.0324978	0.774377	0.9586958	0.9993565
Htr1b	-0.0366359	0.6433031	0.9588497	0.9993565

Cnrip1	0.021061	1.3969057	0.9588652	0.9993565
Ildr2	0.0292994	-0.499879	0.9589015	0.9993565
Nup54	-0.0071543	5.2299725	0.9589101	0.9993565
Ahsa2	0.0074732	5.1197109	0.9589121	0.9993565
Fahd2a	-0.0138014	1.7972999	0.9589868	0.9993565
Ndufc1	0.0080367	5.2329583	0.9589974	0.9993565
6230400D17Rik	0.0692597	-0.951388	0.9590414	0.9993565
Bclaf3	-0.0061387	4.7005189	0.9590899	0.9993565
Tgfbrap1	0.0087663	4.9969942	0.9594967	0.9993565
Rem1	-0.0393902	-0.5616336	0.9595724	0.9993565
Rngtt	0.0069651	5.4990814	0.9595853	0.9993565
Man1a2	0.0065144	6.296992	0.9596549	0.9993565
Slc18b1	-0.018205	1.6850861	0.9597266	0.9993565
LOC115490443	0.0351551	1.2894721	0.95975	0.9993565
Cyth1	0.0104306	5.9342163	0.9597642	0.9993565
Prpf4	-0.0071934	5.0301521	0.9597806	0.9993565
Nutf2	0.0094217	5.0954276	0.9598819	0.9993565
Cd63	-0.0087563	8.1203267	0.9598873	0.9993565
Pcdhb12	-0.0249127	-0.0547399	0.9599396	0.9993565
Ubn2	-0.0063231	6.3842982	0.9600052	0.9993565
Anpep	0.0194265	3.3120285	0.9600134	0.9993565
Ifi205	0.0254757	0.8018732	0.9600259	0.9993565
BC035044	0.010388	5.0415133	0.9600322	0.9993565
Atp6v1g2	-0.016891	1.9928311	0.960035	0.9993565
Gm20346	-0.0433839	0.1256056	0.9602596	0.9995023
Dazap2	0.0061632	8.269167	0.9603574	0.9995023
Prickle3	-0.0094491	3.649515	0.9603679	0.9995023
Gm17501	0.0384494	0.1663259	0.960773	0.9997062
Klhl42	-0.0064219	4.2801344	0.9607913	0.9997062
Rtf1	-0.0053909	6.3648407	0.9608181	0.9997062
Gm527	0.0414424	-0.0429405	0.9609593	0.9997062
Adgra3	-0.0110977	4.1071995	0.9610501	0.9997062
Cbx8	-0.0163816	2.5149365	0.9610797	0.9997062
2310011J03Rik	-0.0071945	5.0845737	0.9611422	0.9997062
Asb17	-0.0452172	-0.0906175	0.9613551	0.9997062
Hsbp1l1	0.0482366	-0.4471036	0.9613965	0.9997062
Gm41553	0.0525195	-0.0868401	0.9614123	0.9997062
mt-Rnr1	-0.0084044	9.5844864	0.9615424	0.9997062
Sucla2	-0.0050905	5.9045409	0.9616507	0.9997062
Cops9	-0.0091119	6.3897685	0.9616527	0.9997062
Card14	0.0469217	-0.0608216	0.961702	0.9997062
Nup205	-0.0063065	6.4802971	0.9617226	0.9997062
Cdkn2d	0.0073154	6.7969655	0.9617381	0.9997062

Aste1	0.0166801	2.5527825	0.9618824	0.9997062
Tbca	-0.0069167	6.0287657	0.9619026	0.9997062
Kbtbd4	-0.0084527	4.0683232	0.961909	0.9997062
Tigd3	0.0168069	2.018097	0.9620385	0.9997062
Gpr146	0.0069746	5.7648671	0.9620958	0.9997062
Fbxo25	0.0112147	3.2166444	0.9621026	0.9997062
Prrg2	0.0138271	2.6513688	0.9621223	0.9997062
Rrn3	-0.0063344	5.3239228	0.9621591	0.9997062
Lincred1	0.0260439	2.5784063	0.9621707	0.9997062
Cse1l	-0.005446	6.9158686	0.9623151	0.999709
Gm46359	0.0568545	-0.8422715	0.9623341	0.999709
Bag3	-0.0075003	4.4709857	0.9623749	0.999709
Aknaos	-0.0231562	-0.3817263	0.9624353	0.999709
R74862	0.0528845	-0.0700631	0.9625038	0.999709
Slc14a1	-0.0067139	7.5464915	0.962559	0.999709
Bco2	-0.037257	-0.4083071	0.9629862	0.9999081
Slc9b2	0.0219023	4.8976207	0.9630316	0.9999081
Echdc1	0.0081572	4.1265934	0.9631955	0.9999081
Plekha3	0.0058949	4.8813256	0.9632054	0.9999081
Odc1	-0.0068114	8.9235865	0.9632776	0.9999081
Riok3	0.0067064	7.5103814	0.9632871	0.9999081
Wdr45b	0.0072351	5.0451764	0.9633462	0.9999081
Gm5617	-0.0127953	2.465489	0.9633998	0.9999081
Psmc6	-0.006396	6.9583389	0.9634459	0.9999081
Faap20	0.0103855	3.6286419	0.9634664	0.9999081
Npc2	0.0065302	7.6792941	0.9635357	0.9999081
Strn3	-0.0046667	6.3655754	0.9637138	0.9999081
Prkch	-0.011208	4.2225745	0.9638177	0.9999081
Mns1	0.0119386	6.3049748	0.9638356	0.9999081
Fam228b	0.0503587	-0.1813836	0.9639162	0.9999081
Nek8	0.0113149	3.5078901	0.9639277	0.9999081
Gsto1	0.0060253	5.3825124	0.9640428	0.9999081
Rheb	0.0048113	5.9408515	0.9640746	0.9999081
Ugcg	-0.0047673	6.8663634	0.9640861	0.9999081
Gtf3c1	0.0070189	5.7855666	0.9640882	0.9999081
Esco2	0.0075262	6.7241196	0.9641935	0.9999081
Smc4	0.006775	9.3423459	0.9642176	0.9999081
Gm45162	-0.0206068	-0.2384302	0.9642292	0.9999081
Socs2	-0.0139105	3.8333169	0.9643364	0.9999168
Laptm4a	0.0066566	8.0075511	0.9644392	0.9999168
Tlcd5	-0.0144819	-0.0840902	0.964442	0.9999168
Hadha	0.006155	7.2701513	0.9646005	0.9999168
Fam13b	-0.0052297	6.7460824	0.9646581	0.9999168

Zfp329	-0.0067738	4.446519	0.9648597	0.9999168
1810014B01Rik	-0.0156825	2.0113012	0.9648717	0.9999168
Serpina3g	-0.0095065	4.801429	0.9648813	0.9999168
Srxn1	-0.0069147	4.4238465	0.964956	0.9999168
Bst2	-0.009154	4.986621	0.9649823	0.9999168
Tceal1	0.0156375	1.2513043	0.9651081	0.9999168
Tceal8	-0.0078825	5.8684376	0.96514	0.9999168
Syde1	0.0153408	2.8816152	0.9651593	0.9999168
Brd1	-0.0052512	6.5428292	0.9651933	0.9999168
Pamr1	0.0272099	1.0162762	0.9652269	0.9999168
Kmt2b	-0.005959	6.4563087	0.9652663	0.9999168
Nt5m	0.0095488	2.6362216	0.9654019	0.9999908
Mb21d2	0.0383797	0.1600941	0.9656107	1
Gm12751	0.05721	3.262063	0.9657981	1
Sox9	-0.0242355	1.4786193	0.9658774	1
Selenom	0.0107991	4.5650456	0.9660334	1
Galnt5	0.018181	2.8726206	0.9660819	1
Zfp354c	-0.0138842	3.3094835	0.9661383	1
Nlgn1	-0.0464603	-1.0296162	0.9662089	1
Hmga1b	-0.0143296	1.0732209	0.9663522	1
Rprd2	-0.0047804	5.8858635	0.9664119	1
Adgrg2	0.0505434	-0.9263245	0.96647	1
Ppil3	-0.0067159	4.5459808	0.966602	1
Gm32016	0.0369919	-0.7268989	0.9666151	1
Gm40462	-0.0251152	-0.6681447	0.96662	1
Grk4	0.0176523	1.4450696	0.9666829	1
E130215H24Rik	-0.0184818	0.0191293	0.9667875	1
Col5a1	0.012984	7.4643606	0.9668952	1
Psmd5	-0.005163	5.8829396	0.9669916	1
Slc15a2	0.037484	4.5460991	0.967045	1
Gm42226	0.1841636	4.9854664	0.9670489	1
Rpl3-ps2	0.0181922	3.6965656	0.9672124	1
Ctnnbl1	-0.0048828	5.0657934	0.9672973	1
Gpr107	-0.0059891	5.1293673	0.9673733	1
Lrrc58	0.0060166	5.5294049	0.967398	1
Prpf3	-0.0054371	5.6074082	0.9674087	1
Atp6v1f	0.006137	5.7427791	0.9677452	1
Mfn1	-0.0052125	5.0625438	0.967776	1
Ppp4r4	0.0687547	-0.9450661	0.9678248	1
Ogg1	0.012732	3.0338853	0.9678449	1
Fzd2	0.0576987	-0.0323419	0.9679026	1
Eif4a2	-0.0054601	7.9513436	0.9679722	1
Stn1	-0.0088483	3.1675648	0.9680818	1

Gpank1	0.0064191	4.2788891	0.9681165	1
Nvl	0.0063737	5.9583081	0.9682091	1
Rab35	-0.0046578	6.1928752	0.9682353	1
Rps2-ps13	0.0111027	8.571334	0.9682474	1
Catsper2	-0.013281	0.3269711	0.9683048	1
Chid1	0.0060951	4.1352671	0.968333	1
Alkbh6	0.014056	2.5877014	0.9684181	1
E330033B04Rik	-0.0135101	1.8599112	0.9685644	1
Mmachc	-0.013097	3.1282371	0.9686274	1
Ccdc9	0.0067014	5.2689316	0.968742	1
Gm10243	0.0369564	0.4787883	0.968806	1
B230217C12Rik	-0.0207389	0.8300046	0.9688699	1
Gnb2	-0.0042109	8.1691144	0.9689607	1
Zfp358	0.0104133	3.0828846	0.9689812	1
Rexo5	0.0107901	4.0171142	0.9690334	1
Gm40258	-0.0477483	-0.1660341	0.9690599	1
Ireb2	0.0047796	6.8714411	0.9691469	1
Bloc1s3	0.0095777	3.3803914	0.9692398	1
Zc3h18	-0.0051279	6.5318633	0.9692827	1
Top3a	-0.004682	4.1353729	0.9693819	1
Ccdc61	0.0121419	3.4296061	0.9694801	1
A630066F11Rik	0.0262514	0.61161	0.9695215	1
Gm10925	-0.0122946	2.8688408	0.9695226	1
Tgfb1	-0.0057211	7.6873604	0.9696443	1
Marchf7	-0.0042136	6.5382458	0.9697613	1
Ackr2	-0.0395059	-0.0391318	0.9698278	1
Fmnl1	-0.0063798	7.645227	0.9698284	1
Fam76a	-0.0038129	5.4885024	0.9698778	1
Psma3	0.0067235	7.1265681	0.9699964	1
Mms22l	0.0064958	5.463915	0.9701038	1
Ccn2	-0.0096048	5.0429769	0.9701379	1
Lipo2	0.0591449	-0.3651274	0.970188	1
Cxcr6	0.0295265	1.1787998	0.970208	1
Cbr3	0.0086261	2.0574601	0.9702924	1
Dyrk1a	-0.0040574	6.9966877	0.9703635	1
Tnfrsf17	-0.0331088	0.3519271	0.9704466	1
Ubap2	-0.0048818	6.368135	0.9705817	1
Arhgef4	0.0115275	2.7109806	0.9707974	1
Afg1l	0.0124234	2.0442381	0.9708456	1
Mdga1	0.0100216	2.7634601	0.9708803	1
Arhgap22	-0.0365988	-0.4797855	0.9710311	1
Nsf	-0.0036754	5.9163355	0.971133	1
Ankra2	0.0073232	4.2904219	0.9712835	1

Pnrc2	0.0043005	7.3419676	0.9714887	1
Tmem141	-0.0091922	2.6602397	0.9717113	1
Czib	-0.0048557	4.3706264	0.9718081	1
Syde2	-0.0124285	1.5449581	0.9718883	1
Fignl2	-0.0158123	1.5445336	0.9719148	1
Tapbpl	-0.0047196	3.8432003	0.9719765	1
Nckipsd	-0.0060504	2.8452546	0.9719963	1
Hmgcs1	0.0073782	5.4982013	0.9720135	1
Mfsd4a	-0.013789	1.407836	0.9720468	1
Gm12764	-0.0150894	1.3113952	0.9721877	1
Tbc1d32	-0.009348	1.6782981	0.9722135	1
Gm46218	0.0303743	-0.739941	0.9722582	1
Piezo2	0.0401216	-0.1070046	0.9723003	1
Gstz1	-0.0066529	4.3895223	0.9723285	1
Fndc1	-0.0202158	1.642881	0.972389	1
Mcts2	0.0162213	-0.614639	0.9723944	1
Samd10	-0.0076833	2.3248382	0.9724146	1
Mon1a	0.0072993	3.4894895	0.9724711	1
Nr5a2	-0.0401593	-0.391749	0.9725884	1
Tubb2a	0.006805	5.6722814	0.9726194	1
Mmp16	-0.0113496	3.4816769	0.97272	1
Prpsap2	0.0065169	4.1076531	0.9727912	1
Retsat	0.0116199	3.1949187	0.9728291	1
Rdh5	-0.0254669	0.3550818	0.972874	1
Pvr	0.0093746	2.2261023	0.9730343	1
Phlpp2	-0.0046453	5.6724305	0.9730431	1
Pex12	-0.0087628	2.9375653	0.9730658	1
Cap2	-0.0086359	2.0935328	0.9731178	1
Pole	-0.0047167	6.0523241	0.9731193	1
Ift52	0.0058683	4.6164562	0.9733073	1
Gm31719	0.0166159	0.9242268	0.9733388	1
Nxpe3	-0.004544	4.3525367	0.973575	1
Gm16001	0.0309977	1.0728446	0.9735921	1
Szrd1	-0.0033713	6.6275259	0.97369	1
Gjc1	0.0217169	1.2329909	0.973796	1
Katnb1	-0.0049587	3.8036939	0.9738651	1
4933404O12Rik	0.015146	3.9541679	0.9740699	1
Crocc	0.0082376	2.9381559	0.9742349	1
Ahsa1	0.004047	6.6822798	0.9742853	1
Ilf3	-0.0039578	6.1326366	0.9743206	1
Ube2v1	-0.0048633	5.7673837	0.974416	1
Wdr45	0.0066055	4.4025831	0.9745924	1
Pdk3	0.0039563	5.7365378	0.9746169	1

Ik	-0.0031891	7.185351	0.974747	1
Slc19a1	0.0069016	3.4534485	0.9747531	1
9130019O22Rik	0.0144381	1.8909421	0.9747556	1
Taf4	0.0048327	5.7409988	0.9750912	1
Gm46914	-0.0407414	-0.9311246	0.9751252	1
Tbce	0.0065522	5.4663554	0.9751316	1
Aldh5a1	-0.0098524	1.2206775	0.975243	1
Trmt112-ps2	0.0396848	-0.4387763	0.9755559	1
Atg13	-0.0036007	4.9588941	0.9755633	1
Tomm34	-0.003599	5.3072222	0.975574	1
Folr1	0.020958	-0.4287802	0.9755769	1
Hspa5	0.004019	9.2030082	0.9755992	1
Mzf1	-0.026888	-0.1068115	0.9757117	1
Rgs3	0.0071187	5.2399757	0.9758256	1
Dync1li2	-0.0031547	6.4380889	0.9760151	1
C730036E19Rik	0.0577041	-0.1940789	0.9760287	1
Tvp23b	0.0044113	4.5735051	0.9762182	1
Tbc1d10c	-0.0044352	5.0152672	0.9762278	1
Msantd4	-0.0037513	4.7270645	0.9762866	1
Grm1	-0.0290456	-0.8436169	0.9763887	1
Gm3226	-0.0099431	2.4447844	0.9764649	1
Ccdc116	-0.0232128	0.7364922	0.9765136	1
Mllt1	-0.0038851	5.2024725	0.9765688	1
Gbp3	0.0097647	4.1535096	0.9766727	1
Cyb5r3	0.0042834	6.1867052	0.9767603	1
LOC118567335	-0.0108548	1.7332449	0.9767628	1
Gm5451	-0.0278095	-0.7032729	0.9767919	1
Nicn1	-0.0176754	1.7726273	0.9768018	1
Gm10320	-0.060863	-0.6754753	0.9769304	1
Zfp647	0.0098958	1.1378489	0.9772601	1
Adgb	-0.0282715	0.0144894	0.977272	1
Krtcap2	-0.0035433	5.5185292	0.9774023	1
Gm10575	0.0568335	-0.9144018	0.9778367	1
Trappc6a	0.0090803	3.5242905	0.9778434	1
Ephb6	-0.0214988	0.0620311	0.9778469	1
Krcc1	-0.0031735	6.2052576	0.9778491	1
Zfhx4	0.0103017	4.0482213	0.9780349	1
Rfxank	-0.004767	3.815788	0.9781019	1
Il1r1	-0.0039168	4.5934947	0.9781083	1
Ndufa7	0.0045404	6.0064368	0.9781328	1
Fmnl3	-0.0038013	5.8040706	0.978145	1
Icam5	0.0201818	0.2991314	0.9781537	1
9030025P20Rik	-0.0162542	0.1135733	0.9781865	1

Rhot1	0.0038411	5.4029891	0.978194	1
Zfp40	0.0097674	3.118056	0.9782113	1
1810009A15Rik	-0.0093898	3.2457618	0.9783054	1
Nanos1	-0.0069144	3.5125276	0.9783467	1
Wdr82	0.003625	6.6572369	0.9784152	1
Cbfa2t3	0.0064413	5.9637784	0.9785674	1
Smug1	0.0111855	2.8046726	0.9786863	1
Unc5a	-0.0152603	0.2313867	0.9787536	1
Mrrf	0.0059234	3.9242409	0.9789206	1
Srpx	0.0081792	3.7415536	0.9790863	1
Nsd2	0.0040424	7.4831588	0.9791056	1
Spata24	0.0088317	3.5578791	0.9792127	1
Zfp947	-0.0172229	0.780881	0.9792502	1
C87436	0.0056679	3.6665383	0.9792736	1
Gdpd1	-0.0070883	3.1141293	0.9792933	1
Acox1	0.0039708	5.5512582	0.97932	1
Mis12	0.0040207	6.0574258	0.9794149	1
1810010H24Rik	-0.031933	-0.4221235	0.9795046	1
Cd200	0.0088062	5.0725078	0.979511	1
Tk2	0.0061502	4.3183054	0.9795901	1
Il20rb	0.044212	0.1152996	0.9796555	1
Slc66a1	-0.0056686	3.0090876	0.979755	1
Pank1	-0.0053802	3.1078043	0.9798138	1
Wnk4	-0.0083512	2.2630114	0.9798185	1
Igkv6-14	0.0157816	2.791576	0.979895	1
Eif4g2	0.0031818	9.8735139	0.9799174	1
Celsr1	0.0143556	2.3063635	0.9800184	1
Ankle2	0.0040618	6.1540481	0.9801352	1
Gm40733	-0.0298412	-1.3480669	0.9803405	1
Sorbs2	-0.006124	4.6904379	0.9805581	1
Nudt8	-0.0093132	2.5560131	0.9806572	1
Kctd12	0.0040772	8.1939079	0.9806912	1
Nagk	-0.0047618	3.6800624	0.9807872	1
Dynlrb1	0.0033773	6.2204421	0.9809226	1
Zgpat	-0.0025361	4.3553179	0.9811774	1
Naglu	0.0070695	4.3624609	0.9811813	1
Cyb561	0.0121109	1.3386571	0.9811878	1
Tob2	-0.0030504	5.5384603	0.9813027	1
Rbbp5	-0.0022474	5.808552	0.9813416	1
B4galt4	-0.0030151	3.2656858	0.9814766	1
Klc2	-0.0064964	3.0983904	0.9814877	1
Oaz2	-0.0033949	5.5932269	0.9817177	1
Syt8	-0.0154104	-0.5088944	0.9817488	1

Mpdz	0.004374	4.2183834	0.9818017	1
Sema3b	0.0141849	3.3041033	0.9818685	1
Srp72	-0.0022592	7.1609616	0.9820107	1
6530409C15Rik	-0.0181699	0.0155627	0.9820172	1
Gm13803	0.0219369	0.1722074	0.9822081	1
Fbxo47	-0.0262145	-0.0146069	0.9822705	1
Gm41724	0.0481111	0.2506208	0.9824719	1
Cdkn1b	-0.002474	7.2895054	0.9824867	1
Thap12	0.0030349	5.8003969	0.9825276	1
Ccdc150	-0.0068097	1.6498893	0.982784	1
H2-Q7	-0.0075834	0.4011723	0.9828426	1
Ass1	0.0072947	5.1615683	0.9829188	1
Ensa	0.0024288	6.6817368	0.9829687	1
Zfp414	0.0049545	4.751211	0.9829722	1
Creb3	0.0045155	4.2545567	0.9829787	1
Matr3	0.002657	7.7344013	0.9830172	1
Pim1	-0.0026608	7.0887441	0.9833131	1
Ep400	-0.0026493	7.237533	0.9833375	1
Aifm2	-0.0032347	3.4728439	0.9835452	1
Ctps2	-0.0024201	5.1962633	0.9835541	1
2700046G09Rik	0.0071745	-0.8228314	0.9836395	1
Gm807	0.0071118	0.2730422	0.9836412	1
Aqr	-0.002083	6.0797156	0.9838342	1
Tlcd2	0.0103309	2.8110468	0.9839213	1
Epn2	0.0071103	3.2971446	0.9841839	1
Gm13127	0.0285966	0.6880166	0.984338	1
Polr3f	-0.0023311	4.5454656	0.9845383	1
Cyp4x1os	-0.0426105	-0.971055	0.9846696	1
Ythdf2	0.0027087	5.8351199	0.9847335	1
Eaf2	-0.01053	1.1192258	0.984782	1
Hspg2	0.0054708	6.4088292	0.9848665	1
Vapa	-0.0017842	7.0100048	0.9852162	1
Ror1	0.0067905	-0.274305	0.9854014	1
Atp5j	0.003173	7.0347587	0.9855042	1
Atf6b	-0.0016403	5.5670173	0.9855128	1
Prr16	-0.0148771	0.8802289	0.985643	1
Zfp827	-0.0079493	2.4528136	0.9856551	1
Plod1	-0.0032859	5.216018	0.9856655	1
Zfp385a	-0.0022171	4.9439627	0.9858098	1
Gm13552	0.0827465	-1.1697765	0.9858184	1
Sdf2	0.0025074	4.9772124	0.9859695	1
Tmem50a	-0.0020371	7.1594733	0.986009	1
Ddias	0.0052945	4.8023135	0.9860275	1

Sec61g	0.004546	7.6687079	0.986104	1
Gmfg-ps	-0.004383	0.5960946	0.986257	1
Etfb	0.0027181	6.5263502	0.9863217	1
Imp4	-0.0020472	5.0152765	0.986379	1
Vps41	-0.0014603	5.7997047	0.9864143	1
Tmem221	0.0084848	-0.0516961	0.9866733	1
Caml	-0.0034214	4.2479516	0.9868692	1
4930513N10Rik	-0.0096381	-0.2859119	0.987047	1
Zfp423	-0.0071772	2.0994377	0.9871125	1
Cdiptos	-0.0312669	-0.722173	0.9872522	1
Rab43	0.0026323	7.4384739	0.9875403	1
Adss	-0.0013178	6.6534408	0.9876988	1
Vamp8	-0.002018	6.536894	0.9877157	1
Api5	-0.00154	7.663918	0.9877195	1
Rpgr	-0.00135	2.7157307	0.9877389	1
Fzd4	-0.0028129	4.1343863	0.9880107	1
Mat2b	0.0023214	6.4057138	0.9880418	1
Pmpcb	0.0036546	5.2033838	0.9880662	1
Hcfc1	0.003007	8.1894445	0.9880871	1
Cbfa2t2	0.0046955	4.2118283	0.9882183	1
Kcnk5	0.0040679	2.4155202	0.9882262	1
Dcun1d2	-0.0023174	4.471018	0.9882275	1
Dnase1l3	0.0081034	3.376236	0.988314	1
Gpr132	0.0112026	2.2689183	0.9883613	1
Pcna-ps2	0.0239809	4.8153726	0.9883778	1
Tecpr2	-0.0026508	3.8014406	0.9884018	1
Gm36470	0.023727	0.5174173	0.9884952	1
Fzd8	0.0101844	2.1059424	0.9884984	1
Sh3bgrl2	-0.0010122	4.0738541	0.9887032	1
Cmip	-0.0015977	7.2857053	0.9887791	1
5930403L14Rik	0.0482391	-0.7894079	0.9889906	1
Strip1	0.0037958	5.1007962	0.9891038	1
Chst1	-0.0258525	-0.0120588	0.9892328	1
Igkv8-28	-0.0006892	2.2228063	0.9893229	1
Bend7	0.0503161	-1.0274629	0.9896154	1
Idh3g	0.0024709	6.4086564	0.9897995	1
Dync1h1	-0.0012759	7.5321982	0.9898234	1
Ptgr1	-0.0012108	5.9197832	0.9898432	1
St6galnac3	-0.0019914	2.7311233	0.9898483	1
Sertad3	0.0048171	4.3089232	0.9899756	1
Gm8783	0.0539234	-0.7726865	0.9899765	1
Atxn7	0.0031733	5.4343108	0.9900056	1
Pdap1	0.0018629	6.8604291	0.9900346	1

Orc5	-0.0018294	5.2508669	0.9900549	1
Dera	0.0040693	5.0195752	0.9903583	1
Abl1	-0.0011495	5.9654643	0.9906114	1
Zfp800	0.0015697	6.2782783	0.9907562	1
Zfp746	-0.0021005	4.8214383	0.9907563	1
Ano8	0.0060526	3.0790565	0.9909656	1
Csde1	-0.0009876	8.1772077	0.9910667	1
Opa1	0.0025349	6.2808182	0.9911126	1
Ankrd42	0.0400737	-0.3308679	0.9911486	1
Hmgb2	0.0021793	10.580329	0.9912689	1
Sec11c	0.0023895	6.9706706	0.9912725	1
Esf1	-0.0012123	5.2413404	0.9914033	1
Ddx17	0.0018841	9.0387658	0.9914977	1
Sf3b1	0.0015963	9.0065443	0.9915298	1
Sugt1	-0.0015502	5.8477635	0.991576	1
Axin2	0.0104466	1.5470321	0.9916175	1
Gm46371	-0.0208419	-0.3724679	0.9916399	1
Col18a1	0.0056733	3.2335695	0.9916762	1
Ripk3	-0.001063	3.9505746	0.9916917	1
Pkd2	0.0033973	4.6360929	0.9917053	1
Usp15	0.0025378	8.1012347	0.9917233	1
Hps5	-0.0009671	4.6488699	0.9918529	1
Heatr1	-0.001778	5.3390499	0.9918882	1
Zfp712	-0.0001462	2.2465526	0.9920429	1
Cds1	0.0097742	2.0838879	0.9921171	1
Cbx5	-0.0009051	8.4420617	0.9921981	1
Zfp473	-0.0143695	1.1888586	0.9923664	1
Zfat	0.0056472	2.4079541	0.9923906	1
Hps1	0.0041928	4.4384321	0.9925655	1
Anapc1	0.0022609	7.0306517	0.9926028	1
Zc3h13	-0.0007015	6.0567719	0.9927827	1
Adam15	0.0018323	5.4763058	0.9928907	1
Kctd9	-0.0002657	5.1828101	0.9929488	1
Dnajb13	0.0311677	-0.4093731	0.9931269	1
Tdg	-0.0006613	4.7321812	0.9932029	1
Cep83	-0.0007682	5.5962419	0.9932418	1
Pdia5	0.0055836	3.3578558	0.9932489	1
Rad51d	0.0045604	3.9641527	0.9935578	1
Adamtsl3	-0.0173068	0.8161515	0.9935931	1
4930523C07Rik	-0.0006184	5.8679306	0.9938079	1
Thbd	-0.0006069	5.6729167	0.9938267	1
Zcchc17	0.0018068	5.2495245	0.9940537	1
Sgsm2	-0.0065256	2.281881	0.9941051	1

Ube3c	0.0020979	5.7354061	0.9942075	1
Ndc1	0.001707	5.7282821	0.994312	1
Vwa8	0.0026788	4.4877309	0.9943309	1
Hddc3	0.0001362	2.6135612	0.9943524	1
Obi1	0.0001603	3.8179186	0.9944199	1
Adam11	-0.0066481	0.2407032	0.9945044	1
H2aj	0.0015096	7.3510927	0.9945317	1
Fcf1	-0.0010933	5.2916968	0.994542	1
Tbl1x	0.0021323	5.9363695	0.9945697	1
2310022A10Rik	-0.0006815	4.285459	0.9945784	1
Ubxn4	-0.0004423	6.5375402	0.994693	1
Gm52877	7.16E-06	3.7995148	0.9947849	1
Ube2l6	0.0031375	8.5362708	0.9950475	1
Abcf2	-0.0006279	5.9185413	0.995085	1
Wdr33	0.0011324	6.1467638	0.9951024	1
Cfl2	0.0032245	4.6595489	0.9951431	1
Cct3	0.0012922	6.8471437	0.9951558	1
Eif3h	-0.0003593	7.7952857	0.9951931	1
Senp1	0.002014	6.1584988	0.9952352	1
Gm33690	0.0193064	0.5026325	0.9954083	1
Gm42162	0.0386513	-0.7723854	0.9954125	1
Rab33b	0.0016864	4.3976216	0.9954151	1
Sumo1	0.0011753	6.7695222	0.9954176	1
Srek1ip1	-0.0001489	4.4302496	0.995705	1
Fam193a	-0.0006137	5.5122095	0.9957258	1
Inppl1	0.0035167	4.5445888	0.9957265	1
BC004004	-0.0007103	5.4221044	0.9958047	1
Steap2	-0.0223722	0.3523895	0.9958213	1
Plekha4	7.17E-06	-0.4639601	0.9959239	1
Epha2	-5.70E-05	3.5657689	0.9961668	1
Pomgnt2	-0.0066387	1.6059323	0.9963473	1
Atp6v0c	0.000647	5.4493998	0.9963848	1
Gm5913	-0.0191478	-0.4599966	0.9964594	1
Cstf2t	0.0003991	5.5751563	0.9964722	1
Cirbp	0.0034324	5.0272022	0.9965385	1
Pnkd	0.0027366	3.4514209	0.9965612	1
Sh3tc1	0.0032553	3.7343674	0.9966225	1
A830082N09Rik	-0.023711	0.0917122	0.9966394	1
Gm19412	-0.0150692	0.2656586	0.9966495	1
Pdk2	0.0040574	3.554924	0.9967124	1
Arl1	0.0009555	6.3863054	0.9967378	1
Ercc1	0.0023602	3.2077429	0.9968118	1
Wfs1	-0.0037214	2.3883981	0.9968669	1

Camk2n2	-0.034581	-0.1631147	0.996896	1
Apon	-0.0355889	-0.3211241	0.9970048	1
Gpt	-0.0007747	1.5529685	0.9970189	1
Anks6	0.0065924	0.4795631	0.9970745	1
Tpm3	0.0002018	8.9239508	0.9971053	1
Zfp692	0.0034072	4.2512815	0.9971284	1
P4ha1	-0.0002106	5.5827614	0.9971766	1
Smim14	0.0011782	6.6575501	0.9973084	1
Cacna1c	-0.0088417	1.3861495	0.9973837	1
Tmem161b	-0.0002628	3.9282309	0.9974847	1
Bfsp1	0.0217586	0.7176362	0.9977215	1
LOC118568082	0.0453704	-0.2370782	0.9977714	1
Lrp1	4.91E-05	7.2769963	0.9977894	1
Rilpl1	0.0023049	2.9331233	0.9978328	1
Prelp	-0.0008686	5.0790376	0.9979997	1
Dcaf12l1	0.0315962	-0.879636	0.9980541	1
Parpbp	0.0004276	5.0804133	0.9980952	1
Zfp267	-0.0028301	2.8485141	0.9981044	1
Nek4	0.0043591	3.2892931	0.9981148	1
Dnajb4	0.0011987	6.6450453	0.9981888	1
Msl3l2	0.007117	1.8739514	0.998377	1
Ranbp17	0.00268	1.5564258	0.9984003	1
Hrct1	0.0066908	0.3317863	0.9984184	1
Ccdc189	-0.004532	2.9132847	0.9985947	1
Ercc4	0.0031797	3.8485131	0.9986783	1
Shc1	3.03E-05	7.1348812	0.9987428	1
Atox1	0.0011571	5.1624562	0.9987827	1
Polr2g	0.0016312	4.8536222	0.9988872	1
Taf3	0.0002391	4.5943962	0.9989085	1
Lysmd4	0.0033289	2.7489493	0.9989572	1
Zbtb48	0.0015857	3.1118439	0.9990868	1
Garnl3	0.0143252	0.5037105	0.9993693	1
Cd48	0.0003381	5.2548251	0.9994429	1
Ankzf1	-0.0003493	4.0924582	0.9995296	1
Gm10638	-0.0197325	0.0967823	0.999562	1
Tcf25	0.0004377	7.4268004	0.9996129	1
Brpf1	0.0009323	6.1972681	0.9996849	1
Rnf170	0.002624	4.1761855	0.9997993	1
Specc1l	0.0008576	5.6197755	0.9998034	1
Nipsnap3b	0.0006014	4.3628704	0.9998821	1
Cldn34c1	-0.0059455	1.2078236	0.9999432	1
Slc25a18	0.0551323	-0.4905835	1	1
LOC118567646	0.046218	-0.5540298	1	1

LOC118568080	0.0419264	0.0109798	1	1
4930590J08Rik	0.0374102	-1.0559001	1	1
Gm16553	0.0351738	-1.066517	1	1
Rps11-ps1	0.0337442	-0.8480021	1	1
Baalc	0.0334901	-1.0701267	1	1
Slco5a1	0.0324492	-0.2413728	1	1
Rgs16	0.0318502	-0.4351749	1	1
Gm40740	-0.0288671	-0.6903893	1	1
Ascl2	0.0283357	-0.54475	1	1
Gm6611	-0.0275465	-0.6883053	1	1
Gm51544	0.0253055	-0.7883549	1	1
0610009E02Rik	0.0242308	-0.5653512	1	1
Gm10045	-0.0238249	-1.11119	1	1
Pgpep1l	-0.0236171	-0.350821	1	1
Sytl5	-0.0235627	-0.9040948	1	1
1700086P04Rik	0.0232033	-0.5665466	1	1
Tmem151a	-0.0218637	-0.9988219	1	1
Sgtb	0.021209	-0.2742215	1	1
Nat14	0.0208129	0.2441291	1	1
Gm46404	0.0207912	-0.5758817	1	1
Vsig2	0.0195994	-1.008122	1	1
Ccdc39	-0.0194841	-0.393721	1	1
Rbm20	-0.0185163	-0.7081403	1	1
Gm51797	0.0172391	-0.2785189	1	1
Gm29050	0.0165406	-0.7624999	1	1
Slc32a1	-0.0164169	-0.7422875	1	1
Asgr2	0.0160264	-0.9042614	1	1
Gpr162	-0.0159698	-0.1695255	1	1
Gm14862	0.0156831	-0.9730929	1	1
Car9	0.0155072	0.8303629	1	1
Snx31	0.0153645	-0.2793142	1	1
Aldh1a3	0.0153301	-0.0066027	1	1
Mafa	-0.0140481	-0.8347615	1	1
Gm52456	-0.0140216	-0.9039103	1	1
Kcng2	0.0138697	0.6166582	1	1
Gm12669	0.0135868	0.0607241	1	1
Gm52310	-0.0131326	0.9748884	1	1
Lrrc2	-0.0126055	-0.8859704	1	1
Gm33046	-0.0123247	-0.8991205	1	1
Rhcg	-0.0119937	-1.0010561	1	1
Gm9892	0.0119486	0.9798131	1	1
Crip3	-0.0117803	-0.7813755	1	1
Esyt3	0.0116836	0.0243679	1	1

Ctxn1	0.0116444	0.3546342	1	1
Pcdhb10	0.0113052	0.2454615	1	1
Gm16023	0.0107828	-0.1749668	1	1
Ifi44l	0.0105576	0.6583798	1	1
Eda2r	0.0104049	-1.1990229	1	1
Zfp82	0.0103479	1.0389975	1	1
4921507P07Rik	0.0101956	0.3784838	1	1
Gm10777	0.010168	2.0215131	1	1
Carmn	0.010087	-0.3904401	1	1
Gm10420	-0.0098611	-0.1457418	1	1
Disc1	-0.0086485	0.5696953	1	1
Egf	0.0081694	-0.2930869	1	1
Gm38482	0.008148	-0.843381	1	1
Itpa-ps1	-0.0080516	0.0607063	1	1
Zfr2	0.008034	-0.3890518	1	1
Acot6	-0.0080062	1.2698131	1	1
Gm2981	-0.007865	-1.169137	1	1
AU015836	0.0077761	-0.6211344	1	1
Fam81a	-0.0076297	-0.9895549	1	1
Gm7846	0.0071281	1.9775228	1	1
Zfp334	0.0070138	0.8660822	1	1
Tril	-0.0069853	0.9099432	1	1
Gm11346	0.0067932	0.0846675	1	1
Gm38553	-0.0066087	-0.2339481	1	1
Zfp366	-0.0065086	0.6304665	1	1
Gm15590	-0.0064738	0.2045956	1	1
Polg2	-0.0063932	1.6753675	1	1
Gm33724	0.0063739	0.9308757	1	1
Acad10	0.0063582	1.4471894	1	1
Gm40573	-0.0050497	0.8413261	1	1
LOC118568526	-0.0049819	-1.000874	1	1
A530020G20Rik	-0.0049589	0.0489541	1	1
E230016M11Rik	0.0046316	0.0102911	1	1
Hykk	0.0045654	0.7029668	1	1
Gm17082	-0.004428	-0.2071091	1	1
Gm52832	-0.0043655	-0.6182521	1	1
C920009B18Rik	0.0041975	1.6672376	1	1
Tha1	-0.0039189	1.0845423	1	1
Hs3st1	0.0035993	0.9104752	1	1
Nudt18	0.0031833	3.8108418	1	1
Dnal4	0.0031451	3.1675099	1	1
Fkbp2	0.003123	1.5660133	1	1
Rab11fip3	0.0030252	3.0676543	1	1

Pcdhga10	-0.0028735	-0.6764543	1	1
Gm40193	0.0027074	-0.8211664	1	1
Tmem53	0.0026238	0.8155009	1	1
Mybph	-0.0026082	-0.3217802	1	1
LOC115486538	-0.0024587	0.1847814	1	1
Gm19605	0.0020994	1.487819	1	1
Kif3c	-0.0018242	2.4763055	1	1
Grem2	-0.0017382	-0.7418781	1	1
Ptbp2	0.0017314	4.7792492	1	1
Gm14698	-0.0017196	-0.5014557	1	1
Gm10132	0.0016956	-0.605418	1	1
Cep83os	0.0016113	3.3985805	1	1
Pradc1	0.0013963	3.3601671	1	1
Mtln	-0.0013735	3.4917672	1	1
9930012K11Rik	-0.0011907	1.4039767	1	1
Camsap3	0.0011781	0.2995447	1	1
Naa35	0.0011313	4.9009453	1	1
Prkag2	0.001108	4.4092899	1	1
Prmt9	0.000987	4.428933	1	1
Gm13372	-0.0008927	0.90799	1	1
Mpi	0.0008012	3.6102673	1	1
Ccnb1-ps	0.0006456	0.2462726	1	1
Fbxl7	0.0004688	1.4825784	1	1
Ambra1	0.0004544	5.0976942	1	1
Nuak2	0.0004092	4.2196417	1	1
Dhx38	0.0003995	6.3326479	1	1
Uqcrb	0.000383	6.9467971	1	1
Cyb5d2	-0.0003241	2.7051564	1	1
Gm5963	-0.0002598	0.9655156	1	1
Chka	0.0001659	4.3644026	1	1
Ubfd1	-0.000131	5.6032823	1	1
Igkv4-74	9.67E-05	1.9335231	1	1
Rad1	-7.94E-05	3.5935084	1	1
Ier3ip1	1.10E-05	5.7218307	1	1
